# Dissecting the effect of soil on plant phenology and berry transcriptional plasticity in two Italian grapevine varieties (*Vitis vinifera* L.).

**DOI:** 10.1093/hr/uhad056

**Published:** 2023-03-28

**Authors:** Alessandro Vannozzi, Corrado Perin, Fabio Palumbo, Marco Sandri, Paola Zuccolotto, Sara Zenoni, Silvia Farinati, Gianni Barcaccia, Massimo Pindo, Paolo Sonego, Alessandro Cestaro, Margherita Lucchin

**Affiliations:** Department of Agronomy, Food, Natural resources, Animals and Environment (DAFNAE), University of Padova Agripolis, 35020 Legnaro, Italy; Department of Agronomy, Food, Natural resources, Animals and Environment (DAFNAE), University of Padova Agripolis, 35020 Legnaro, Italy; Department of Agronomy, Food, Natural resources, Animals and Environment (DAFNAE), University of Padova Agripolis, 35020 Legnaro, Italy; Department of biotechnology, University of Verona, I-37034, Verona, Italy; Big&Open Data Innovation Laboratory, University of Brescia, 25122 Brescia, Italy; Research and Innovation Centre, Fondazione Edmund Mach, via E. Mach 1, 38010, San Michele all'Adige, Italy; Department of Agronomy, Food, Natural resources, Animals and Environment (DAFNAE), University of Padova Agripolis, 35020 Legnaro, Italy; Department of Agronomy, Food, Natural resources, Animals and Environment (DAFNAE), University of Padova Agripolis, 35020 Legnaro, Italy; Research and Innovation Centre, Fondazione Edmund Mach, via E. Mach 1, 38010, San Michele all'Adige, Italy; Research and Innovation Centre, Fondazione Edmund Mach, via E. Mach 1, 38010, San Michele all'Adige, Italy; Research and Innovation Centre, Fondazione Edmund Mach, via E. Mach 1, 38010, San Michele all'Adige, Italy; Department of Agronomy, Food, Natural resources, Animals and Environment (DAFNAE), University of Padova Agripolis, 35020 Legnaro, Italy

## Abstract

Grapevine embodies a fascinating species as regards phenotypic plasticity and genotype-per-environment interactions. The terroir, namely the set of agri-environmental factors to which a variety is subjected, can influence the phenotype at the physiological, molecular, and biochemical level, representing an important phenomenon connected to the typicality of productions. We investigated the determinants of plasticity by conducting a field-experiment where all terroir variables, except soil, were kept as constant as possible. We isolated the effect of soils collected from different areas, on phenology, physiology, and transcriptional responses of skin and flesh of a red and a white variety of great economic value: Corvina and Glera. Molecular results, together with physio-phenological parameters, suggest a specific effect of soil on grapevine plastic response, highlighting a higher transcriptional plasticity of Glera in respect to Corvina and a marked response of skin compared to flesh. Using a novel statistical approach, we identified clusters of plastic genes subjected to the specific influence of soil. These findings could represent an issue of applicative value, posing the basis for targeted agricultural practices to enhance the desired characteristics for any soil/cultivar combination, to improve vineyards management for a better resource usage and to valorize vineyards uniqueness maximizing the terroir-effect.

## Introduction

The ability of a given genotype to produce a variety of distinct phenotypes as a result of the environment is known as phenotypic plasticity (PP) and represents a particularly important feature for sessile organisms such as plants, especially in the agricultural field, where sudden and extreme climate changes are opposed to the need to obtain stable productions both in terms of quality and quantity [1]. PP is a significant ecological phenomenon, but our understanding of its underlying genetic and molecular bases is still lacking, given the complexity of genotypes and environmental components interactions [2]. With the introduction of omics technology, researchers have just lately begun to focus on plasticity at the transcriptome level. In model organisms, transcriptome plasticity has been described [3–8], however, few investigations have been undertaken in plants grown in the open field, where they are exposed to a variety of environmental stimuli that cause complex responses in terms of gene expression, metabolic activity, and epigenetic alterations [9].

Grapevine (*Vitis vinifera* L.) is one of the world’s oldest and most frequently grown perennial fruit crops. Its global socioeconomic importance is due to the high-quality attributes of berries, principally employed for wine production and fresh consumption. Grapevine is characterized by a pronounced PP, as different wines can be produced from the same genotype when cultivated in different environmental conditions [10]. This PP is observable as well as when different enology practices in producing wine are applied: also in this case typical peculiar products are obtained according to the specific vinification techniques adopted. The growing interest of the scientific community and wine producers on genotype *per* environment (GxE) interactions and the strictly related concept of *terroir*, which itself embodies one of the most evident examples of PP, led to a boost in studies on this issue. The term terroir was coined by French winemakers, and after various definitions, it is now widely accepted that it refers to the set of specific characteristics expressed in the final product by the geography, geology, and climate of a specific location, interacting with plant genomes and agricultural practices [11].

The soil and its physical, chemical, and microbiological features have been recognized as terroir variables that define the uniqueness of berry composition by vines growing in a certain environment [12,13]. The nutrient availability of soils has a fundamental influence on plant growth and affects the flavonoid composition tissues [14]. Similarly, soil physical–chemical characteristics such as the parent material, age, micronutrient pool, structure, and texture influence plant developmental processes and affect secondary metabolites accumulation [15]. Bokulich et al. revealed geographical patterns in grape and wine microbiota associated with wine chemical composition [16] and Pinto *et al.* studied the grapevine microbiome landscape in relation to the vegetative growth cycle of the plant, indicating that the grape microbiome may influence *terroir* [17]. Almost all these studies concern the determination of the link between certain *terroir* factors and the final product of the vine, be it the grape or the wine it produces, without investigating what are the molecular determinants that explain these interactions.

A pioneering study aimed to investigate transcriptional plasticity during berry ripening in vines grown under different environmental and agronomical conditions was performed on Corvina [18]. The analysis revealed that approximately 1500 genes (5% of the studied annotated coding genes) resulted “plastic”, showing different expression profiles during berry ripening in at least one of the 11 locations considered in the study. Afterwards, a metabolomic analysis was realized in same cultivar, allowing to identify several metabolites that represent a sort of “terroir signature” for each vineyard and to find a correlation between terroir-sensitive metabolites and changes in the expression of their biosynthetic genes [19]. Similar results were obtained for the cultivar Garganega grown in 4 different sites, where a clear correlation between gene expression and phenylpropanoids accumulation was detected [20]. Dal Santo et al. have attempted to investigate the foundation of grapevine GE interactions describing berry plasticity at the transcriptome, methylome, and allele specific expression (ASE) levels in two varieties grown in three distinct conditions across two vintages [21]. Using a novel approach, they identified genes with expression patterns that were independent of genotype or environment, dependent on genotype but independent of environment, dependent on environment regardless of genotype, and linked to G x E.

Even though all these studies have enormously contributed to extend the knowledge on the molecular determinants of PP and the terroir effect in grapevine, their irrefutable limit lies in the complexity of variables and co-variables that came into play in field comparison experiments. Very often the identification of “plastic” regulatory networks based on the comparison of the same clonal variety grown in different locations was inevitably the result of the contribution of multiple endogenous and exogenous factors, such as the age of the plants, the type of rootstock, the cultivation practices, the pedoclimatic characteristics of the areas in comparison, etc. To well define the thin boundaries between nature and nurture it is necessary to identify and isolate all the individual factors that drive the *terroir* and elucidate their possible interactions with the plant.

Here we propose an original approach aimed to dissect the weight of soil components in the grapevine plastic response, by keeping constant all the other terroir variables. The analysis was carried out on Corvina and Glera cultivars, grown in three different types of soils under the same environmental and agronomical conditions. Molecular results, combined with phenological and physiological parameters measured during the whole seasonal cycle, revealed a genotype and tissue-specific effect of soil factor on grapevine berry plasticity and indicated specific gene networks related to *terroir*. The use of an innovative statistical approach to estimate the weight of variables in gene expression patterns, the identification of soil-specific gene clusters, and the integration of such data with available genomic data has allowed to identify plasticity regulatory genes, most of which represent “switch genes” involved in the transition between the herbaceous and maturation phase in berry development. Moreover, promoter enrichment analyses on soil-modulated genes identified the R2R3-MYB TFs VvMYB13, VvMYB14 and VvMYB15 as key regulators of the grapevine plastic response to soil.

## Results

### Phenology and physiology are differently affected by soil in Corvina and Glera varieties.

**Figure 1:**
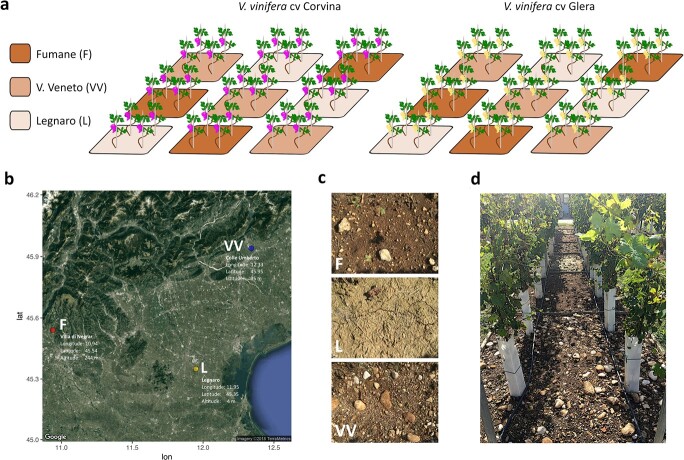
Experimental design. (a) Schematic representation of the experimental plan: three soil caissons per thesis: Fumane (F), Vittorio Veneto (VV) and Legnaro (L), four plants per caisson, two varieties (Glera and Corvina). All plants were grafted onto the same rootstock (Kober 5BB). (b) Satellite map of the Veneto region indicating the three localities where soils were collected, their latitude, longitude, and altitude: the locality of Fumane (F) within the Valpolicella DOCG area, the locality of Vittorio Veneto (VV) within the Prosecco Valdobbiadene-Conegliano area, and the L. Toniolo experimental farm of the University of Padua located in Legnaro (L) municipality, where the experiment took place. (c) Detail of the three different soils considered in the study. (d) Picture showing the experimental plan for the 2017 vintage.

Two-year-old certified clonal varieties of *V. vinifera* cv. Glera and Corvina were transplanted into concrete caissons filled with three soils taken from various Veneto districts associated to worldwide known wine production in 2015 (Fig. 1).

**Figure 2:**
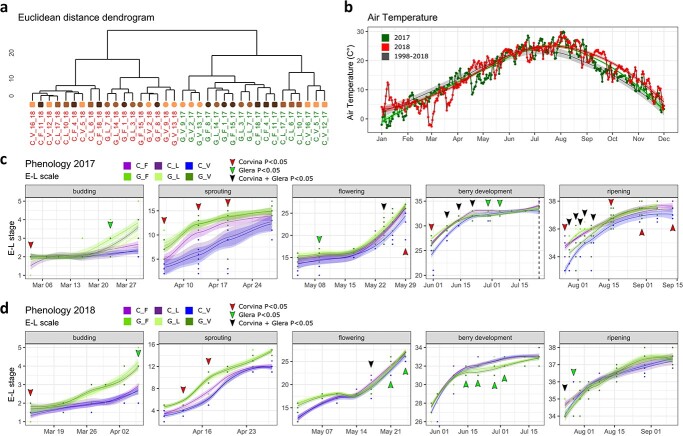
(a) Cluster dendrogram of plant phenology based on the average value of the E-L phenological scale in three biological replicates (n = 3). Data were normalized by the dataset median value. Pearson’s correlation values were converted into distance coefficients to define the height of the dendrogram. Sample names are composed by variety abbreviation (C, Corvina; G, Glera) followed by the indication of the type of soil (F, V, L), the caisson number (1–18) and the growth year (17, 18). Green and red indicate plant grown in 2017 and in 2018, respectively. Data are the average of the three biological replicates (n = 3) each one constituted of 4 plants (one caisson). (b) Daily mean air temperature (°C) related to the 2017 (green line), 2018 (red line), and the average trend of the period between 1994 and 2018 (grey line) based on ARPAV meteorological station located near the experimental field (Lat. 45.348, Lon. 11.953), 2 m from the ground level. (c,d) Phenological progression of Corvina (purple) and Glera (green) varieties in 2017 and 2018 growth seasons. For clarity, the growth curve was subdivided in distinct panels representing different developmental stages (budding, sprouting, flowering, berry development and ripening). Green lines represent Glera variety grown in different soils (light green, green and dark green) whereas purple lines represent Corvina one (purple, violet and blue). The colored arrows indicate the dates on which the Kruskall-Wallis test detected statistically significant differences (p < 0.05) between plants grown in different soils in Glera (green arrows), Corvina (red arrows) or in both varieties (black arrows) as reported in Tables S1 and S2.

In order to understand whether different soils affected the development of both varieties, the phenology of individual plants grown in concrete caissons filled with Fumane (F), Vittorio Veneto (VV), and Legnaro (L) soils was monitored weekly in both 2017/2018 years. Glera and Corvina phenological development was comparable throughout the whole growing season, with the exclusion of the early stages of shoot development, when Glera anticipated the budburst of about one week respect to Corvina. The distance dendrogram built from the average developmental value (E-L scale) of each biological replicate considered (n = 3), depicted a sharp separation between years and cultivars (Fig. 2A), suggesting that the vintage factor has the main impact on plant phenology, followed by the genotype one. As a matter of fact, climatic conditions in 2017 and 2018 seasons were quite different (Fig. 2B).

In 2017, the air temperature was always above the last 20-years average, especially during summer (July–September), and precipitations were insufficient for an adequate water supply (206 mm from March to August), making it necessary to occasionally water vineyard (Fig. S1). Although air temperature was still above the last 20-years average, the climatic conditions during summer 2018 were less extreme and precipitations were more constant and abundant (538.4 mm from March to August; Fig. S1). The soil effect was more evident in the 2017 than in 2018. In fact, while in 2017 samples were subclustered based on the soil, in 2018 this factor did not appear to be discriminant. Nonetheless, in both vintages, the Kruskal-Wallis test identified significant differences in phenology (Fig. 2C,D; Tables S1, S2), most of which observed during the reproductive phase, at flowering (19 E-L), during berry development (31–33 E-L), and at véraison (35–36 E-L), suggesting that soil factor exerts its strongest influence on the first phase of berry development, while when fruit ripening is in progress (i.e. during the second phase of berry ripening), the phenology might be less affected by soil itself (at least in 2018). The behavior of the two varieties in different vintages was however controversial: while in 2017 Corvina proved to be the most plastic variety, showing significant differences at numerous time points during berry development and ripening, in 2018 it showed a less marked response to the soil factor (Fig. 2C,D). On the contrary Glera maintained comparable levels of plasticity in both seasons.

**Figure 3:**
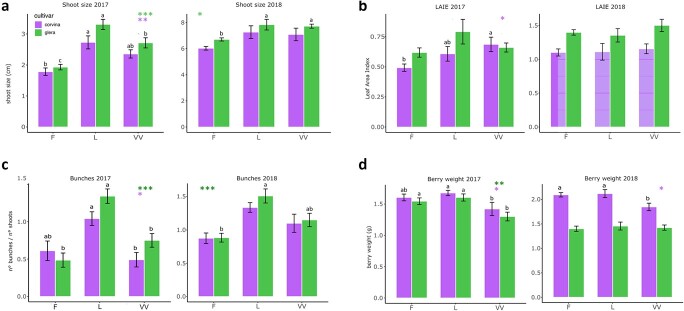
Physiological parameters registered over 2017 and 2018 vintages in Glera (green bars), and Corvina (green bars) cultivars grown in the three different soils (F, VV, and L). Bars indicate mean values ± SE (n = 12). Colored asterisks indicate the significance of soil factor on the parameter on each variety based on ANOVA (^*^ = p value <0.05; ^**^ = p value <0.01; ^***^ = p value >0.001). When ANOVA was significant Duncan’s test at P > 0.05 was performed. Different letters indicate significant differences among soils.

For what concerns physiological parameters throughout the vegetative phase, differences were reported from plants grown in the different soils (Fig. 3, Table S3, S4).

**Figure 4:**
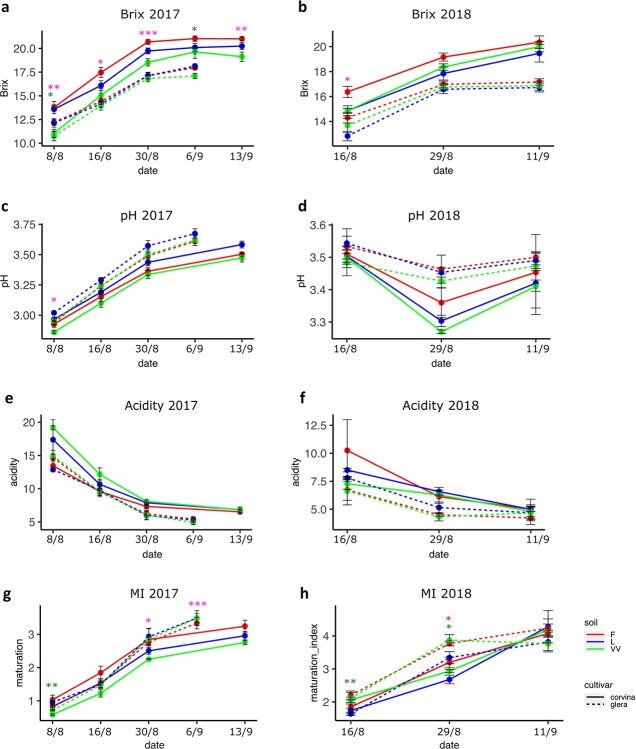
Berry maturation parameters registered over 2017 and 2018 vintages in Glera (dotted line), and Corvina (solid line) cultivars grown in the three different soils (F, VV, and L). Throughout berry maturation, total soluble solids (TSS) in °Brix were assessed from 4 berries each plant per time point, using a hand refractometer. Titratable acidity measurement (g/L tartaric acid equivalents) was performed for each biological replicate (caisson) according to the standard procedures. With the same juice, pH was determined by means of a pH-meter. For each sampling date, maturation index (MI) was also computed from the ratio between the TSS value and the titratable acidity value of each biological replicate. Dots indicate mean values ± SE. Colored asterisks indicate the significance of soil factor on a given parameter for each variety based on ANOVA; ^*^ = p value <0.05; ^**^ = p value <0.01; ^***^ = p value >0.001.

Again, 2017 season appeared to be more influent on plasticity, influencing several parameters including the shoot size at the curvature of the horizontal spurred cordon (F with lower values), the vegetative growth (LAIe) (in 2017) (Fig. 3), and the shoot length. Differences in growing development in 2018 were less appreciable. While the parameters related to the vegetative growth of the plant seem to be more influenced by the soil on Glera, those more closely related to the ripening of the berry and its quality showed greater plasticity in Corvina, at least in 2017 vintage (Fig. 4; Table S3, S4). pH and TA were significantly affected at the onset of ripening stage. On the contrary, in 2018, Glera appeared to be more plastic than Corvina. TSS value was higher in plants grown in F soil compared to those of VV and L, but such characteristic statistically faded toward the ripening stage. Nonetheless, maturation index (TSS-TA ratio) showed a statistical delay of berry maturation in VV soil and L soil in the first stages of ripening of 2017 and 2018, respectively (Fig. 4; Table S3, S4).

### Glera and Corvina showed different magnitude and specificity of transcriptional plasticity.

**Figure 5:**
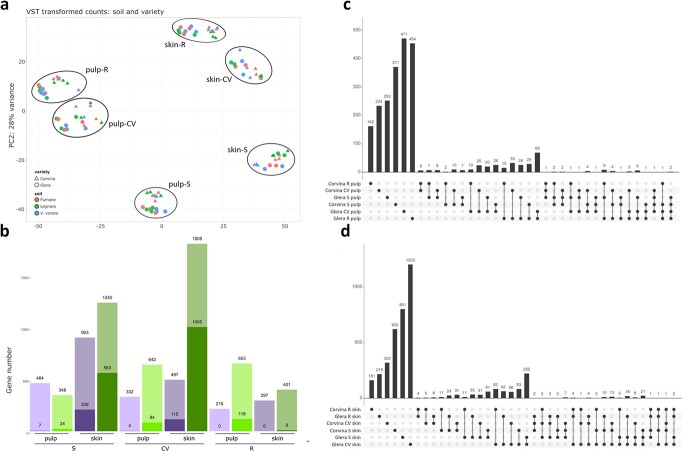
(a) Principal Component Analysis (PCA) based on VST normalized data obtained from 108 RNAseq sample constituted of skin and pulp tissues of Glera and Corvina varieties in softening (S), complete-véraison (CV) and ripening (R) phase. Colors indicate different soils considered, namely Fumane (F), Legnaro (L) and V. Veneto (VV). (b) Differentially expressed Genes (DEGs) identified by means of ANOVA-like analysis in both skin and flesh of Glera and Corvina varieties at S, CV, and R phenological stages. Colored histograms show the different numbers of significant genes in each condition considered (Purple bars refer to Corvina variety whereas green bars refer to Glera. Light colors represent the amount of DEGs according to a p-value<0.01 whereas darker colors refer to FDR < 0.05. The number of DEGs detected in both cases are reported on the bars. (c,d) Upset plots visualizing the intersections amongst different conditions considered by ANOVA analyses. Single points indicate private DEG identified in a given condition, whereas 2 to *n* dot plots indicate DEGs shared between 2 to *n* conditions.

With the aim of gaining a better comprehension of the molecular mechanisms underlying the plastic responses of Glera and Corvina to different soils considered, we performed an mRNA-seq analysis by means of the HiSeq 2500 platform (Illumina). The analysis was accomplished on a total number OF 108 samples corresponding to the two varieties grown in different soils (VV, F and L), at three developmental stages (softening - S, complete véraison - PS and ripening -R), in two tissues (skin and pulp) and in three biological replicates in the 2017 season. A description of samples names used in this study is reported in Table S5. Sequencing produced a total number of 852.988.367 paired end reads (100 bp length), with an average value of 8 mln reads/sample and a median of 7.4 million reads. On average 76% total reads passed the quality control test and were mapped onto the PN40024 12x V1 prediction of the grapevine reference genome [22] (Table S6). Summarized read count data were normalized using the Trimmed Mean of M-values (TMM) method and eventually transformed using a “variance stabilizing transformation” (VST), which leads different samples to have a comparable variance between them and used to build a Principal Components Analysis (PCA) (Table S7). As illustrated in Fig. 5A, PC1 explained 53% of the total variance and clearly divided samples based on tissue factor (flesh or skin). The PC2 explained 28% of the variance dividing the S, CV, and R stages. Moreover, in both tissues and in each singular stage considered, especially at R, the two cultivars were clearly separated. When looking at the soil effect on a whole transcriptome scale, the PCA did not sharply separate samples, suggesting that transcriptional rearrangements due to this terroir factor are limited to a reduced number of transcripts. Thus, in order to investigate the weight of this variable on the transcriptome rearrangements, successfully mapped read counts were used to identify plastic transcripts as a response to soil factor. An ANOVA-like technique was used to determine the number of transcripts that showed significant variation between soils in both tissues and genotypes at the S, CV, and R phases (ANOVA). When considering DEGs with FDR <0.05, the number of differentially expressed genes was extremely low, with only 11 DE genes in Corvina pulp and 324 in skin. Glera showed a more plastic transcriptional response with 226 genes modulated in pulp and 1577 in skin (Fig. 5B). Despite the more marked transcriptional response in Glera, GO enrichment analyses on the totality of DEGs detected in Corvina pulp, Corvina skin, Glera pulp and Glera skin, showed a more specific response in Corvina, with the pulp tissue modulating genes related to polysaccharide metabolism and cell wall organization and the skin modulating genes related to secondary metabolite biosynthesis and in particular poliketide biosynthesis. Regarding Glera, no GO categories were enriched for pulp tissue, whereas in skin many genes involved in transmembrane transport, response to hormones and endogenous stimulus were modulated (Fig. S2). Given the low number of DEGs with a FDR < 0.05, we preferred to perform an “explorative” analysis considering DEGs with a p-value <0.01.

We identified a cumulative number of 7801 differentially expressed genes (DEGs; p-value <0.01) (6810 excluding genes shared between more conditions) (Table S8), representing the sum of all those genes significantly affected by soil in each of the 12 variety/tissue/stage combinations considered. Again, glera resulted to be more responsive than Corvina to soil factor, modulating a total number of genes equal to 5092 (4674 excluding DEGs shared between the two tissues or different stages) compared to 2709 DEGs (2598 excluding DEGs shared between the two tissues or different stages) detected in Corvina (Fig. 5B). In both varieties the skin tissue was the most responsive one, being always a greater number of DEGs compared to pulp (1697 and 3449 DEGs in Corvina and Glera skin compared to 1012 and 1643 DEGs in Corvina and Glera pulp, respectively).

The only exception was Glera at ripening stage (R) where pulp showed a higher number of DEGs compared to flesh. Finally, moving on to the response of the plant to the soil factor based on berry developmental stage, data indicated that while in Corvina the most responsive phase in both skin and pulp tissues corresponds to softening (S) (903 DEGs in skin and 464 in pulp), in Glera the most responsive phase was CV for skin (1808 DEGs) and R for pulp (653 DEGs) (Fig 5B). Fig. 5C,D graphically illustrate the intersections between the DEGs identified in the different variety/time point combinations in pulp and skin respectively, i.e. those genes shared between multiple conditions and those whose expression was found to be genotype or stage specific.

Results suggest that the soil-response specificity depends on the variety and the time point considered, as there is a much higher number of “private” genes, compared to those shared between multiple conditions.

Despite the higher number of genes modulated in Glera, GO enrichment analyzes conducted on the total DEGs in the two tissues and in the two varieties showed a more specific response in Corvina, whose transcriptional modulation was canalized into specific pathways of secondary metabolism. In fact, GO enrichment analyses for Corvina skin DEGs at CV indicated enrichment ontologies related to secondary metabolic processes, and in detail polyketide biosynthetic processes, confirming what observed in looking at DEGs with FDR < 0,05. These categories encompassed 28 genes belonging to the stilbene synthase family (*VvSTSs*), and 7 genes encoding for phenylalanine ammonia lyase (*VviPAL*), involved in the general phenylpropanoid pathways and in the biosynthesis of precursors of stilbenes. For a detailed description of all enriched categories in different tissue/stage combination in the two variety see Fig. S3,S4.

### A novel statistical method uncovered grapevine GxS interactions.

**Figure 6:**
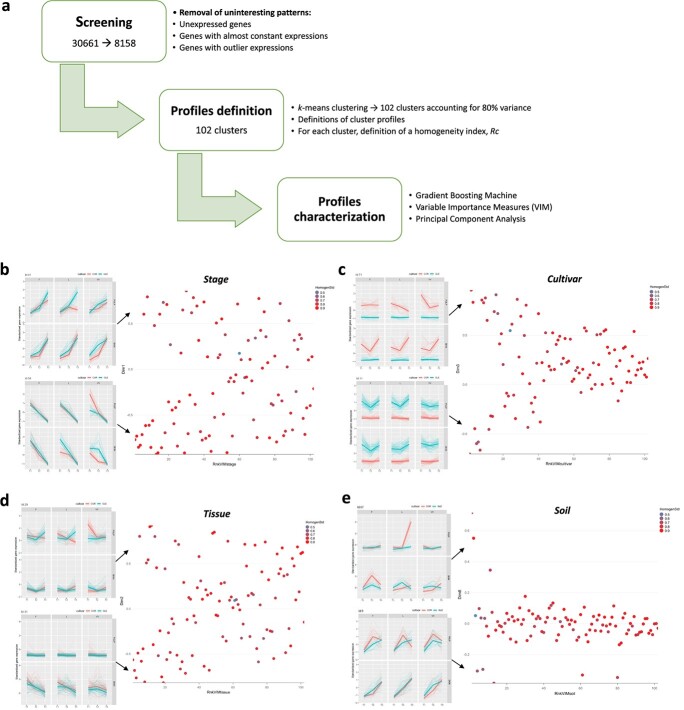
Statistical pipeline and variable hierarchies. (a) Schematic flowchart illustrating the three-step statistical pipeline. See Methods S2 for a detailed description of the pipeline. (b-e) Description of the genotypic (stage, cultivar, and tissue) and environmental (soil) variable-related expression clusters. Scatterplot of the 102 clusters according to the ranking in (b) VIM_Stage, (c) VIM_cultivar, (d) VIM_tissue and (e) VIM_soil (i.e. Rnk_VIM_stage = rank of clusters according to VIM_stage; low values denote high importance of the stage) and to the component loading in the first, third, second and eight principal components (Dim1, 3, 2, and 8), associated with the importance of the stage, cultivar, tissue, and soil variables, respectively. Each dot represents a single cluster, colored according to the homogeneity index, Rc. Relevant examples of variable-specific clusters are given at the side of each scatter plot. See Table S12 for a detailed description of the 102 clusters identified in the study.

We used a three-step data-mining strategy to find the most significant connections between the variables in the RNAseq dataset, concentrating on how stage, cultivar, tissue, and soil (and their interactions) affect gene expression. This method, recently proposed by Dal Santo et al (2018), is able to detect, other than strong evidence, subtle effects that are not easily revealed by common methods. The machine learning technique at the basis of the procedure is a tree-based learning ensemble with the main feature of discovering interactions among variables that are relevant for the analyzed outcome (in this case, the gene expression). The main difference between this tool and traditional methods such as, for example, linear models with interactions, is that the algorithmic procedure is able to reveal which (two-way, three-way, . . .) interactions are relevant, without the need to specify them a priori in the model. Moreover, a much larger sample size would be needed by traditional methods, with a minimum number of observations for each explored interaction. This is not requested by this method, as the gradient boosting machine iteratively performs the analysis on all the residuals, without splitting the sample. In a first screening step, starting from the whole gene set of grapevine genome corresponding to 30 661 genes, we discarded a subset of 22 503 genes with inadequate profiles, i.e. unexpressed genes, genes with constitutive expression, genes with outlier expression (Fig. 6A, Table S9). The remaining dataset, comprising 8158 genes (Table S10), was subjected to a k-means clustering, reducing the genes to 102 clusters containing transcripts with similar patterns amongst experimental conditions and globally accounting for approximately 80% of the total variance in gene expression (Fig. S8). The average profile was then used as a representative of each cluster and the variability of expression around it was used as an index of its representativeness (homogeneity index, *Rc*, Fig. S9). In the third step, the gradient boosting machine (GBM) [23] an advanced machine learning algorithm, was exploited in order to evaluate the extent to which each of the variables (stage, cultivar, tissue, and soil) affects gene patterns. To do that, we used Variable Importance Measures (VIMs), a machine learning statistical tool, able to extract information from black box algorithms, in order to describe the impact of predictors, also in interaction among them, on the outcome. The clusters were then characterized according to these features, by using the median VIMs within them, and this allowed us to describe the associations between clusters and experimental conditions (Fig. S10). Finally, a better interpretation of results was achieved by crossing the VIMs to a Principal Component Analysis (PCA), used to reduce the dimensionality of the matrix composed by 102 variables (columns) given by the average cluster profiles.

Principal components, defined by linear combinations of cluster profiles, were found to be able to discriminate with remarkable accuracy among the variables characterizing the 36 experimental conditions (stage, tissue, cultivar, Fig. S11-S13). The relevance of the stage, cultivar, tissue, and soil factors is shown by the component loadings of the clusters in the first, third, second, and eight principal components (Dim1, 3, 2, and 8), respectively (Fig. 6b-e). The least significant and weakest connection of loadings were seen for the soil variable, and there was little homogeneity within these clusters. In summary, thanks to the three-step statistical pipeline we were able to allocate each of the 8158 modulated genes to one of 102 clusters. Each cluster was described by an average profile, an index of representativeness of this profile and four VIMs, accounting for the impact (for the genes belonging to that cluster) of stage, cultivar, tissue, and soil on gene expression. As a result, we assigned each cluster a rank based on the VIM for each variable. For example, cluster no. 102 is particularly interesting since it has the highest VIMcultivar (1281,5) and the lowest one for all other variables (VIMstage: 6,34; VIMtissue: 5,6; VIMsoil: 1,84) being ranked first for the cultivar variable but only 102nd for all other variables (Table S11 and Table S12).

### Changes in transcriptional levels are influenced by soil in the context of GxS interactions.

**Figure 7:**
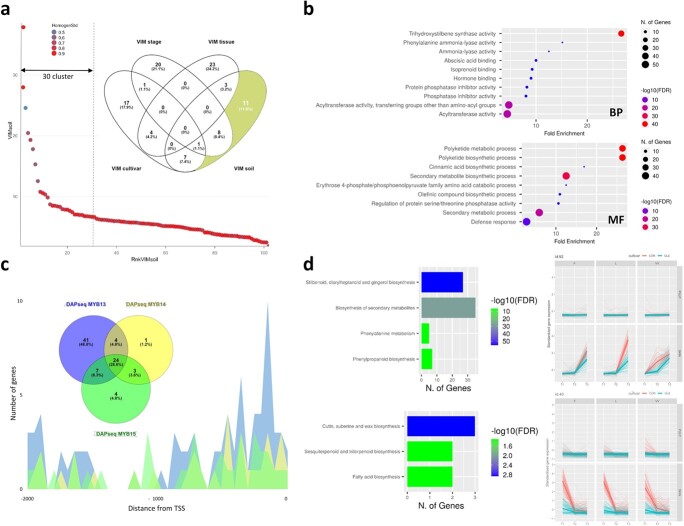
Transcriptional variation according to the soil variable. (a) Scatterplot of the 102 clusters according to the rank in VIM_soil_, with decreasing importance moving from left to right. Each dot represents a single cluster, colored according to the cluster homogeneity index, Rc. On the top right, a Venn diagram showing the number of variable-specific and variable-shared clusters, by taking into account only the 30 most impactful clusters for each of the four variables (soil, stage, tissue and cultivar). The 11 soil specific clusters are highlighted in yellow. (b) Dot plots ranking the enriched Biological Process (BP) and Molecular Function (MF) GO terms within the 11 soil specific cluster genes. The X axis represents the Fold Enrichment, the color is function of the –log10(FDR) whilst the size of each dot is proportional to the number of genes identified enriched for each GO term. (c) Spatial distribution of the promoter sequences belonging to 84 soil specific genes that - according to a recent DNA Affinity Purification Assay (DAPseq)-based study (Orduña et al., 2022) - resulted recognized by MYB13 (blue peaks), MYB14 (yellow peaks) and MYB15 (green peaks) TFs in a window comprised between −2000 and 0 bp upstream the TSS. As illustrated also by the Venn diagram 76 genes are recognized by MYB13, 32 by MYB14 and 38 by MYB15. Of these 24 gene are in common. (d) The bar plots on the left depict the KEGG enrichment analysis for the genes belonging to soil specific clusters 42 and 51. The X axis represents the number of genes enriched in each KEGG category whilst the color of each bar is function of the –log10(FDR). The temporal kinetics for the genes belonging to the two aforementioned clusters are illustrated on the right side of the panel.

Although the 102 clusters accounted for approximately 80% of the total variance in gene expression (Fig. S8), the top 30, alone, accounted for approximately 70% of the total variance. Therefore, we restricted the analysis to the top-30 clusters ranked by VIM for soil, stage, tissue, and cultivar variables to identify both the specific (*i.e.* top-30 ranking for a single variable) and shared ones (*i.e.* ranking in the top-30 for more variables) (Fig. 7A; Table S11).. Gene Set Enrichment Analysis revealed, overall, functional categories related to “polyketide biosynthetic process”, “cinnamic acid biosynthetic processes”, “erythrose 4-phosphate/phosphoenolpyruvate family amino acid catabolic process”, “regulation of protein serine/threonine phosphatase activity”, “olefinic compound biosynthetic process” (Fig. 7B). Except for clusters No. 55 and 47, all the soil-specific clusters included genes with higher expression in Corvina than in Glera (Fig. S132).

In order to identify *cis* elements involved in the soil-specific plastic response, we performed an enrichment analysis on the promoter sequences (600 bp) of the aforementioned 740 genes (Table S14). The analysis indicated the sequence GGTAGGTG as the best-ranked enriched motif (FDR 9.1E-12). Based on PlantDBTF, this element represents the target of the TF VvMYB03. This motif was also identified as the binding site of VvMYB13, VvMYB14 and VvMYB15 based on a recent study aimed at defining the cistrome of several secondary metabolism regulators through a DNA-Affinity Purification Assay (DAPseq) [24] To shed light on the regulation of these soil-responsive genes we therefore screened these DAPseq data to verify how many of the 740 genes were effective targets of MYB13–15. In total, the promoter regions of 101 genes for MYB14 (13.6%), 293 for MYB13 and 110 for MYB15 (75 in common - 25%), resulted effectively linked by the aforementioned TFs (Table S15). By further narrowing the analysis, we only considered those genes linked in the promoter region comprised between −2000 and 0 bp upstream the TSS (Fig. 7C).

Seventy-six genes were linked by MYB13, 32 by MYB14 and 38 by MYB15 (Fig. 7C). Of these 24 genes were in common. Most of the genes were found to belong to soil specific clusters 42 and 51. A large number of genes (32) belonging to cluster 42 was found to encode for *VvSTSs* and were poorly expressed in the pulp tissues of both cultivars, but highly accumulated, with some differences, in the skin of both cultivars passing from CV to R (Fig. 7D). In particular, Corvina skin accumulated more *VvSTS* transcripts than Glera and, most important, transcribed them in a soil-dependent manner. In fact, the number of transcripts detected at R in Corvina skin grown in the L soil was higher than that observed in the other two soils. Moreover, at CV, the STS mRNA accumulation in the Corvina skin was almost null in L and F soils, but clearly detectable in VV. From KEGG enrichment analysis cluster 42 was enriched for specific KEGG pathways such as, flavonoid, phenylpropanoid and stilbenoid biosynthesis and phenylalanine metabolism (Fig. 7D).

**Figure 8:**
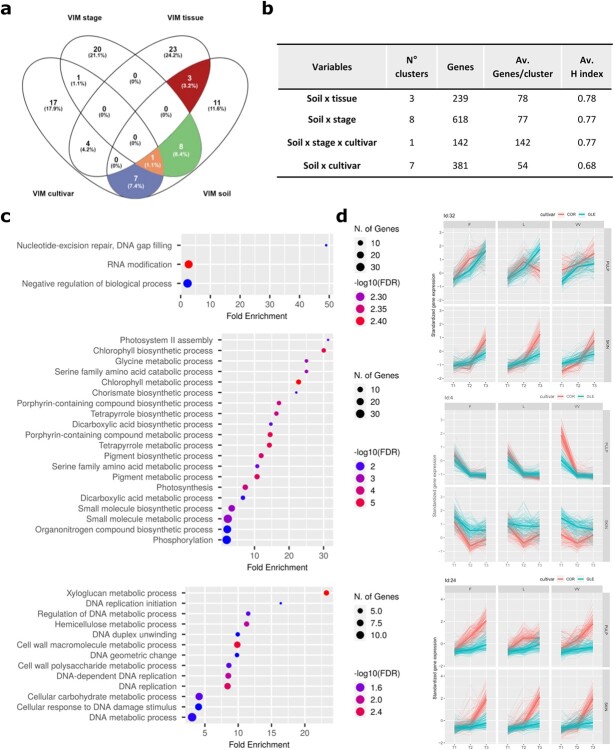
Interaction between soil and other variables. (a) Venn diagram showing the number of variable-specific and variable-shared clusters, by considering only the 30 most impactful clusters for each of the four variables (soil, stage, tissue, and cultivar). The clusters shared between soil and at least one of the other variables are highlighted with different colors. (b) Summary of the principal properties of each of the four categories highlighted in panel (a), including the number of shared clusters, the total number of genes, the average number of genes per cluster and the average homogeneity index are reported. (c) Dot plots ranking the enriched Biological Process (BP) GO terms for three representative clusters. Cluster 30 was chosen as representative of the clusters shared between the soil and stage factors, cluster 44 was chosen as the most abundant cluster (in terms of genes number) among the clusters shared between soil and tissue variables and finally cluster 28 is the only cluster shared among soil, stage and cultivar. The X axis represents the Fold Enrichment, the color is function of the –log10(FDR) whilst the size of each dot is proportional to the number of genes identified enriched for each GO term. (d) Temporal kinetics for the genes belonging to the three aforementioned clusters.

Interestingly, the expression pattern of cluster 51 (*R_c_* = 0.83) turned out to be the opposite of cluster 42. Again, the transcripts accumulation of the 102 cluster genes was almost meaningless in the pulp tissues, while a marked decrease was evident in the skin of both cultivars during the berry phenological progression. This decline was more pronounced in Corvina, where the initial expression (at S) of the cluster was higher. Slight soil-dependent differences were observed when comparing the expression profiles of Corvina skin in L and F soils with the ones in VV. In fact, at CV, in both L and F there was an almost complete transcriptional repression of all genes belonging to the cluster, which instead continued to be moderately expressed, albeit more attenuated, in VV. The KEGG pathways significantly more enriched in cluster 51 were cutin, suberin and wax biosynthesis, fatty acid biosynthesis and metabolism and sesquiterpenoid and triterpenoid biosynthesis (Fig. 7D).

### Transcriptional plasticity also relies on the interaction between soil and other variables.

When analyzing the top 30 clusters ranked by VIM for soil, stage, tissue, and cultivar variables, beyond the 11 soil-specific clusters (see previous section), we also considered those clusters that resulted in the top 30 of two or more variables, so as to highlight the transcriptional plasticity component deriving from the interaction between soil and other variables. The most important association in terms of number of clusters was observed between the soil and stage factors (8 clusters, 618 genes, Fig. 8**A,B**), suggesting that the interaction between phenological stage and soil composition consistently contribute to berry transcriptomic plasticity. As exemplified by Cluster No. 30 (Rc = 0.80), the most enriched functional categories were “Nucleotide-excision repair, DNA gap filling”, “RNA modification” and “Negative regulation of biological process” (Fig. 8C, uppermost panel) and the genes involved in these GO categories, showed, in the temporal kinetics, increasing expression levels in both cultivars except for L where a clear decrease was observed in R for Corvina Pulp (Fig. 8D, uppermost panel).

Soil and tissue variables shared instead three gene clusters (239 genes) that resulted particularly enriched for biological processes related to photosynthesis, chlorophyll metabolism and biosynthesis and pigment biosynthesis. Cluster 44 resulted the most abundant one in terms of genes (R_c_ = 0.82, 123 genes, Fig. 8C, intermediate panel) and clearly demonstrates a sharp decline in the expression of the genes involved in the photosynthetic processes in the pulp of Corvina and Glera from S to R. Also the soil variable seems to play some influence, especially looking at the differences in expression of the aforementioned genes in Corvina Pulp in VV at S, in relation to F and L (Fig. 8D, intermediate panel). The decrease observed in pulp, it’s less evident in the skin of both varieties, suggesting a persistent albeit decreased photosynthetic activity in the outer tissues of the berry. Despite soil and cultivar variables shared 7 clusters (for a total of 381 genes) no significant GO enrichment was observed. Finally, a single cluster (142 genes, R_c_ = 0.77) was shared among soil, stage and cultivar and was mainly enriched for functional categories related to xyloglucan metabolic process, DNA replication, cell wall macromolecule metabolic process and hemicellulose metabolic process (Fig. 8D, lowermost panel). Overall, these genes underwent a more consistent growth in the expression levels in Corvina than in Glera. Again, L soil seems to play a role in the expression pattern of these genes in the Corvina pulp (Fig. 8D, lowermost panel).

## Discussion

Climate, soil, and cultivar are among the major factors involved in terroir expression, with their effect being mediated through the vine. Dissecting their singular weight in the grapevine plastic response is a notable effort, given the difficulty of comparing the behavior of plants grown in different areas blocking all variables except one. To do this we conducted an experiment where all the terroir variables, except for the soil, were kept as constant as possible minimizing the effect due to the agronomical and climatic variability and allowing us to isolate and study in detail the singular effect that different soils exert on Glera and Corvina vegetative and reproductive phases and on berry molecular profile (Fig. 1).

Our study showed that the soil factor, can potentially affect the plant growth and grape quality, as confirmed by the differences found at the phenological, physiological and molecular level. Looking at the pheno-physiological behavior of plants, the comparison of two different seasons, 2017 and 2018, suggests that the soil effect depends on the interactions with other terroir factors including the genotype and vintage. In fact, grapevine phenological development was affected by soil differences in both seasons, but this effect took place with different magnitude over 2017 and 2018 years, without a sharp and stable distinction between theses (Fig. 2). Although the plastic response was more pronounced in 2017 than 2018 in both years soil exerted a more incisive effect in phases following flowering. However, while in 2017 Corvina proved to be strongly plastic especially in the berry developing and ripening phase, in 2018 the plastic response was greater in Glera. As an explanation, soil differences were too weak for determining stable differences, and the predominant influence is driven by other factors or by the interaction with them as observed by [25] and [26]. The latter, investigating the total variance attributable respectively to the climate, soil, and temperature over 37 variables measured in three varieties grown on different soils over five consecutive vintages, observed that vine development and phenology are predominantly driven by the climate, except total shoot length and ripening speed. *De facto*, climate can still be considered the most incisive terroir factor on phenology exerting its effect on the sum of temperature [25]. Another factor that must be taken into consideration, although its effect is not directly measurable, is the fact that, given the young age of the vineyard, the longer periods spent in the different soils have allowed the plants to adapt, reducing their impact on the plastic response. Something similar was observed for physiology: when analyzing the two cultivars separately, in 2017 Glera resulted more plastic than Corvina for many traits related to the vegetative growth (Fig. 3), whereas Corvina resulted more plastic than Glera for those traits related to berry development and maturation such as Brix, acidity, and maturation index (MI) (Fig. 4, Tables S3, S4) supporting the idea by which different cultivars differently respond to environmental changes as observed by [21] comparing two genotypes cultivated in three different environments over two vintages and by [27] comparing the stilbene composition of Merlot and Syrah cultivar in different terroirs. In 2018 the situation was different, with a higher plasticity detected in Glera berries, although in general the plastic response in this year was strongly reduced making it difficult to make reliable comparisons.

Pheno-physiological results were corroborated by transcriptomic analyses. At first glance, transcriptomic data confirmed that the soil, although exerting an effect at the transcriptional level, has a lower impact compared to other variables such as genotype, tissue or the phenological stage (Fig. 5A). Despite this, ANOVA analysis allowed to identify sets of genes showing statistically significant differences depending on the soil. The comparison of the two varieties showed that Glera moves a much higher number of genes (4674 DEGs) compared to Corvina (2598 DEGs). This observation agrees with the higher pheno-physiological plasticity observed in this variety in 2018.

The skin was found to be the most responsive tissue, showing a number of DEGs always (or almost) higher than pulp, while the most important stage in the plant response to the soil factor was softening (S) followed by the complete véraison (CV) (Fig. 5B). An interesting observation lies in the fact that the S-phase DEGs identified in both varieties contained 51 out of 131 (39%) switch genes identified by [28] analyzing the berry transcriptomes of 10 grapevine varieties (five red and five white) and potentially involved in the regulation of the developmental transition at véraison [29]. The majority of these genes were shown to be differently expressed in skin tissue, and several TF transcription factors may be recognized as master regulators of the transcriptome remodeling that occurs throughout the developmental transition from immature to mature growth. Amongst these are *MYBA1/A2* (VIT_02s0033g00410, VIT_02s0033g00390), already well characterized for their direct and crucial role during the transition to berry ripening [30,31], NAC33 (VIT_19s0027g00230), whose regulatory role in organ development has been proposed in many plant species [32–34], including grapevine [35,36], *AGL15a* [37,38], encoding the MADS box protein Agamous-like15a (VIT_13s0158g00100), and the transcription factor gene *WRKY19* (VIT_07s0005g01710) [39]*.* The fact that soil affects these important genes in the transition between the herbaceous and maturation phases supports the concept that this factor might profoundly influence the processes that lead to berry growth and maturity, as demonstrated in phenological investigations. The differential response in the technological maturation phase was much lower, probably because at that stage the plant has already adopted all the adaptive processes necessary to complete its biological cycle and to reach the formation and maturation of the seed. Afterall, with the exclusion of specific gene groups (such as those linked to the metabolism of phenylpropanoids), a decline in the transcriptional activity from véraison to ripening in Corvina and other varieties including Glera had already been observed by [29] and [28].

With the specific aim to target the most significant differences in gene expression due to the soil factor, we adopted a novel statistical approach already used by [21]. This approach comprises a three-step screening and filtering scheme to remove unwanted sources of variability in gene expression, the clustering of gene co-expression profiles based on three different soils, different phases, tissue and genotypes and an estimation of the inner representativeness of the clusters (i.e. the internal cohesion of each cluster). We would like to underline that, although this study was focused on determining the effects of the soil variable on the transcriptional response of vine, this analysis led to the identification of 102 gene clusters that also contain gene groups whose expression was exclusively conditioned by the other variables involved, such as the tissues, the genotype and the phenological phase, as well as their interaction. Due to space constrains, these data are not discussed here, but remain fully available to the grapevine scientific community, representing a treasure trove to be deeply explored.

We thus focused on 11 clusters considered “soil-specific” (Fig. 7A, Fig. S14), which grouped 740 genes (Table S13) enriched in functional categories related to “Polyketide biosynthetic process”, “cinnamic acid biosynthetic processes”, “erythrose 4-phosphate/phosphoenolpyruvate family amino acid catabolic process”, “regulation of protein serine/threonine phosphatase activity”, “olefinic compound biosynthetic process” (Fig. 7B). Although ANOVA analysis indicated Glera as the most plastic variety, with the except of two clusters (No. 55 and No. 47), all soil-specific clusters included genes with higher expression in Corvina (Fig. S14). Among the most intriguing, which significantly contributed to the enrichment study results, are Clusters 19 and 52, which are firmly related to secondary metabolic activities, namely the biosynthesis of stilbenes and other phenolic compounds, in addition to terpenes and sesquiterpenes. These clusters had opposing expression patterns, correlating with prior findings and indicating the rivalry between these two distinct branches of the phenylpropanoid metabolic pathway [40]. The cis elements found within these genes’ promoter sequences were shown to be possible targets of the transcription factors MYB13, MYB14, and MYB15. The control of *STS* and other biosynthetic genes of the shikimate pathway is now a known case [24,41–43]. More intriguing is the apparent involvement of the aforementioned TFs in the regulation of gene clusters linked to the biosynthesis of terpenes and other flavonoids, as well as their involvement in the regulation of genes that show divergent expression patterns. This observation suggests complex regulatory networks that would involve interaction with other specific TFs in the plastic response of these pathways of secondary metabolism. The fact remains that this study would indicate MYB13–14-15 as master regulators of the grapevine plastic response to soil.

To conclude, although the transcriptome plastic response observed in Glera was more pronounced than Corvina, suggesting a higher adaptative capacity of this variety as testified by its larger area of cultivation, Corvina showed a higher transcriptional canalization, meant as the modulation of genes belonging to specific pathways of secondary metabolism, such as the stilbene and, as a consequence the flavonoid one, that strongly affect berry composition and ultimately wine quality. This higher effect on specific compound of great importance on berry and wine quality could explain the higher sensitivity that contradistinguish this variety respect to Glera.

## Materials and methods

### Experimental design

Two-year-old certified clonal varieties of *V. vinifera* cv. Glera and Corvina grafted onto Kober 5BB rootstock were transplanted into concrete caissons (2 m x 2 m x 1.5 m deep) filled with three soils collected from different Veneto regions associated with internationally renowned wine production in 2015. The first soil, denoted by the letter “F,” was gathered in the municipality of Fumane (VR) (Lat. 45.54, Lon. 10.94, Alt. 244 m a.s.l.), which is part of the DOCG Valpolicella and is best known for producing “Amarone” wine. A second soil, denoted as “VV,” was gathered in the Vittorio Veneto (TV) piedmont region (Lat. 45.95, Lon. 12.33, Alt. 95 m asl), which is part of the Prosecco DOCG and recognized for producing the homonymous wine. Finally, a third soil, designated as “L”, was represented by the typical plain soil present in the L. Toniolo university experimental farm (University of Padua, Legnaro PD), where the experiment was conducted (Lat. 45.35, Lon. 11.95, Alt. 8 m asl). A detailed description of the experimental plan is reported in Methods S1 whereas soil physico-chemical characteristics are reported in [44]. F, VV and L soils considerably differed from each other in texture, physical properties and skeleton, micro-, and macro-elements content and based on the International Union of Soil Sciences (IUSS), are classified as “heavy clay” (F soil), or “clay loam” (VV and L soils), respectively (Methods S2 and Fig. S18).

### Phenological and physiological measurements

The phenological progression of the 72 plants previously described was monitored weekly during both 2017/2018 growing seasons, starting from bud-breaking till harvest (from March to September), using the modified E-L system [45]. It is worth mentioning that after véraison the E-L scale refers to the relative berry total soluble sugar (TSS) content, which changes based on the variety considered. Therefore, we defined post-véraison stages in the two varieties under study based on their potential sugar accumulation at harvest (18 and 23°Brix for Glera and Corvina, respectively). For what concerns biometric indicators related to the vegetative phase, the one-year-old shoots used for the horizontal spurred cordon framework were chosen for measuring the shoot size at the curvature based on their circumference. Other analyses, focusing on the period from flowering to véraison of 2018, included leaf gas exchanges, stomatal conductance and net photosynthesis rate using a newly released portable photosynthesis system (LI-6800, LICOR, USA). Other physiological parameters related to both vegetative and the reproductive phase of 2018 were recently described in Perin et al. (2020) and comprised the shoot length, the Leaf Area Index (LAIe), number of bunches and the evolution throughout berry ripening of the total soluble solid (TSS; mostly related to the sugar content), titratable acidity, pH, and berry weight at harvest. Here, they are reported and compared with the same parameters in 2017 season [44].

### Pheno-physiological statistical analyses

The Shapiro–Wilk test was adopted to assess data normality in phenological and physiological analyses. Before the analysis of variance, the variance homoscedasticity among treatments was calculated by means of Levene’s and Bartlett’s tests (ANOVA assumptions). The treatment effects, which included two varieties, three soils, and their interaction, was evaluated using multifactorial ANOVA. Kruskal-Wallis test was used for the phenological data analysis at each time point and when ANOVA assumptions were not met. Where ANOVA showed significant effects of treatments Tukey’s HSD test at the 95% confidence level (P < 0.05) was used as a *post-hoc* test.

### Berry sampling for RNA-seq analysis

For molecular analysis, three phases throughout berry maturation were considered: softening (34 E-L - “berries begin to soften”), complete véraison (36 E-L - “intermediate Brix value”), and harvest (38 E-L - “berries harvest-ripe”). Three berries were gathered from the median part of a cluster representative of each plant at the same day time (about 11 a.m.), avoiding those showing damages or pathogen infection symptoms. Berries collected from plants grown on the same soil-caisson were collected and combined to form a single biological copy (consisting of 12 berries), then instantly frozen in liquid nitrogen. Subsequently, berries were removed from the seeds whereas skin and pulp were thoroughly separated and grinded to fine powder by mortar and pestle in liquid nitrogen and stored in polypropylene tubes at −80°C until further processing.

### RNA purification, library preparation and sequencing.

Total RNA was extracted from 400 mg of skin and pulp tissues using the “Spectrum™ Plant Total RNA kit” (Sigma-Aldrich, St. Louis, MO, USA) according to manufacturer’s instructions with some modifications [46]. RNA quantity and quality were determined using a Nanodrop® 2000 spectrophotometer (Thermo Scientific, Wilmington, DE, USA) and agarose gel electrophoresis. A total number of 108 RNA samples (3 soils x 3 biological replicates x 2 varieties x 2 tissues x 3 time points) were obtained and processed for RNA-seq by Illumina technology. As described in Procedures S3, RNA-seq library preparation, quality and quantification of pooled libraries, and high throughput sequencing with Illumina technology were all carried out at the Fondazione Edmund Mach (FEM; San Michele all’Adige, Italy) Sequencing Platform.

### Identification of differentially expressed genes (DEGs) and gene set enrichment analyses.

The Trimmed Mean of M-values (TMM) approach was used to standardize the summed read count data, which allows for sample normalization while accounting for changes in sequencing depth and sample variance [47]. The normalized read count data were analyzed by one-way ANOVA-like test for the investigation of the soil effect on both cultivars taken together and separately by using EdgeR Version 3.26.5 [48]. Gene Set Enrichment Analyses (GSEA) were applied to the different lists of DEGs (depending on the variables chosen) using the on-line tool ShinyGO [49]. Venn diagrams and identification of common and specific DEGs were performed using the UpSetR online tool (https://gehlenborglab.shinyapps.io/upsetr/) [50].

### Variable importance measure analyses on gene expression data

The gene expressions were evaluated using data mining algorithms to summarize the most relevant associations in the data, with a focus on the extent to which the factors cultivar, stage, soil, and tissue impact gene expressions independently or in combination. With this aim we followed a statistical approach based on the estimation of the Variable Importance Measure [21]. Methods S4 contains a full explanation of the statistical workflow. Venn diagrams were created using Venny v2.1 and the top 30 scoring clusters for each variable’s VIM ranking (http://bioinfogp.cnb.csic.es/tools/venny/). Gene Set Enrichment Analysis for GO and KEGG terms, as well as promoter enrichment analysis were performed using the ShinyGO online tool [49].

Overrepresented GO and KEGG categories were found using a hypergeometric test with a significance level of 0.05. When feasible, bar graphs ranking the top five biological processes were created based on enrichment values [−log10 (p-value)]. Promoters were scanned using TF binding motifs available in all the databases supported by ShinyGO. Instead of defining a binary outcome of binding or not binding (which depends on arbitrary cutoffs), the best score for each TF in every 600 bp promoter sequences was recorded. The student’s t-test was then used to compare the scores observed in a group of genes against the rest of genes and finally the p-values were corrected for multiple testing using FDR.

## Acknowledgements

Authors would like to thank Dr Diego Tomasi (CREA-VE, Conegliano, Italy) for providing the contacts for collecting soils, Dr. Federica Gaiotti (CREA-VE, Conegliano, Italy), Dr Sara Sgubin, Dr Martina Pacifico, Dr Pierfrancesco Boldrini and Dr Enrico Carraro for their help in physiological measurements. The study was carried out within the Agritech National Research Center and received funding from the European Union Next-Generation EU (PIANO NAZIONALE DI RIPRESA E RESILIENZA (PNRR) – MISSIONE 4 COMPONENTE 2, INVESTIMENTO 1.4—D.D. 1032 17/06/2022, CN00000022). In particular, our study represents a research paper within the Task 4.1.1. (Spoke 4) titled: “Next-generation genotyping and -omics technologies for the molecular prediction of multiple resilient traits in crop plants”. This study was also supported by the Starting Grants 2015 CARIPARO project titled “Grapevine plasticity and terroir: a multidisciplinary approach for dissecting the single effect of soil and climate on berry and wine quality”.

## Author Contribution

A.V. and M.L conceived the study. C.P. and A.V performed phenological and physiological measurements and their statistical analysis. S.M., Z.P. S.Z. performed the VIM statistical analysis and helped in the interpretation of results. M.P., P.S. and A.C performed the RNAseq analyses and bioinformatic preprocessing of reads. A.V., C.P., F.P. and S.Z. analyzed the data. The manuscript was written by A.V., C.P., F.P., S.F., G.B. and M.L. and approved by all other authors.

## Data Availability

All raw reads were deposited in the NCBI SRA database with accession numbers PRJEB46163.

## Conflict of interests

The authors declare that the research was conducted in the absence of any commercial or financial relationships that could be construed as a potential conflict of interest.

## Supplementary Data

Supplementary data is available at Horticulture Research online.

## Supplementary Material

Web_Material_uhad056
