# Supplementary material for: Dissecting the effect of soil on plant phenology and berry transcriptional plasticity in two Italian grapevine varieties (Vitis vinifera L.)
Source: Hortic Res. 2023 Mar 28;10(5):uhad056. doi: 10.1093/hr/uhad056 (PMC10199706; doi:10.1093/hr/uhad056)
Supplement: Web_Material_uhad056 [file web_material_uhad056.zip › HORTRES_SI_revised-1.pdf]

Horticulture Research **Supporting Information**

Article title: Dissecting the effect of soil on berry transcriptional plasticity in two Italian grapevine varieties (*V. vinifera* L.).

Authors: Vannozzi Alessandro, Perin Corrado, Palumbo Fabio, Sandri Marco, Zuccolotto Paola, Zenoni Sara, Pindo Massimo, Sonego Paolo, Cestaro Alessandro, Lucchin Margherita

Article acceptance date: Click here to enter a date.

The following Supporting Information is available for this article:

Fig. S1 Ecological characterization of the experimental site.

Fig. S2 GO enrichment analysis in Corvina and Glera tissues.

Fig. S3 GO enrichment analyses in Corvina DEGs

Fig. S4 KEGG enrichment analyses in Glera DEGs

Fig. S5 Step 1, phase I; examples of removed genes.

Fig. S6 Step 1, phase II; examples of removed genes.

Fig. S7 Step 1, phase III; examples of removed genes.

Fig. S8 Variance accounted for (VAF) by clusterization vs number of clusters.

Fig. S9 Examples of clusters with high and low homogeneity index *Rc*.

Fig. S10 Expression profiles and boxplots of VIMs for cluster no. 58.

Fig. S11 Two-dimensional object scores plots of the 36 experimental conditions according to "stage" covariate.

Fig. S12 Two-dimensional object scores plots of the 36 experimental conditions according to "tissue" covariate.

Fig. S13 Two-dimensional object scores plots of the 36 experimental conditions according to "cultivar" covariate.

Fig. S14 Graphical representation of the eleven-soil specific cluster identified in the VIM analyses.

Fig. S15 Scatterplot of the 102 clusters according to Rank in VIM^{c}_{Stage} and loading in the first Principal Component

Fig. S16 Scatterplot of the 102 clusters according to Rank in VIM^{c}_{Tissue} and loading in the first

Principal Component.

Fig. S17 Scatterplot of the 102 clusters according to Rank in $VIM^{c}_{Cultivar}$ and loading in the first Principal Component.

Fig. S18 Soil map of the Veneto in scale 1: 250,000.

Table S1 Phenological parameters 2017.

Table S2 Phenological parameters 2018.

Table S3 ANOVA on physiological parameters 2017.

Table S4 ANOVA on physiological parameters 2018.

Table S5 Description of sample names used for the present study.

Table S6 RNAseq Mapping statistics.

Table S7 Matrix of raw, mean raw and VST normalized counts.

Table S8 Differentially expressed transcripts based on ANOVA analysis.

Table S9 Screening guidelines.

Table S10 Set of the 8158 modulated genes analyzed using the statistical pipeline.

Table S11 Characterization of the 102 clusters identified by the VIM analysis.

Table S12 Detailed description of all the 102 clusters of gene expression defined in the present study.

Table S13 List of 740 genes belonging to the 11 soil specific clusters.

Table S14 Enrichment analysis of the promoter regions of the 740 soil specific genes.

Methods S1 detailed description of the experimental plan

Methods S2 description of soils based on the Veneto soils map provided by the Regional Agency for Environmental Prevention and Protection of Veneto.

Methods S3 Variable Importance Measure (VIM) analysis.

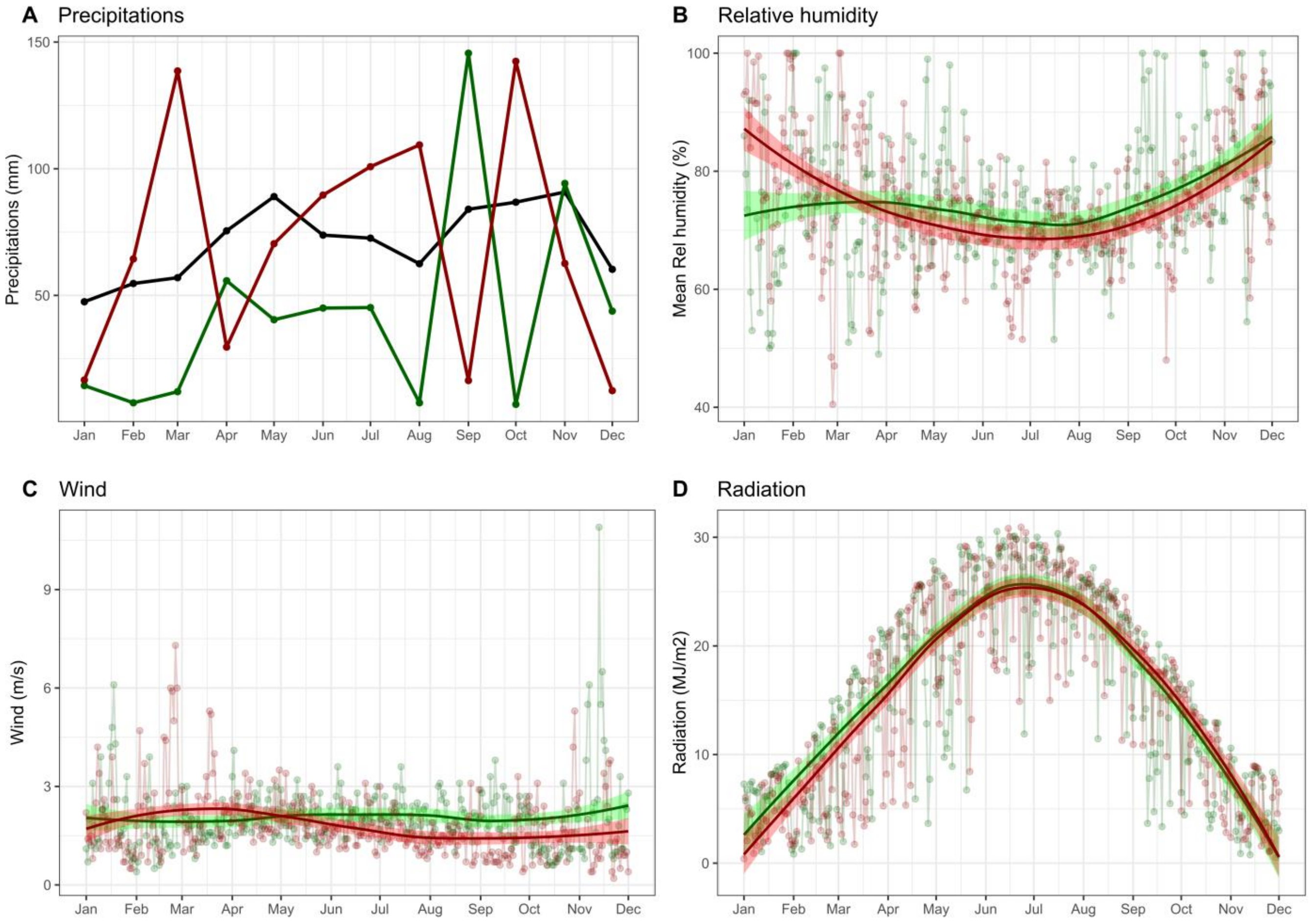

Fig. S1 Ecological characterization of the experimental site. Data recorded from an ARPAV meteorological station located in close proximity to the experimental field (Lat. 45.348, Lon. 11.953), 2 m from the ground level. (A)) Monthly precipitations in 2017 (green line), in 2018 (red line) and averaged in the period 1998-2018 (black line). Average daily relative humidity (B), wind (C) and radiation (D) are also reported. Green lines refer to 2017 whereas red lines refer to 2018.

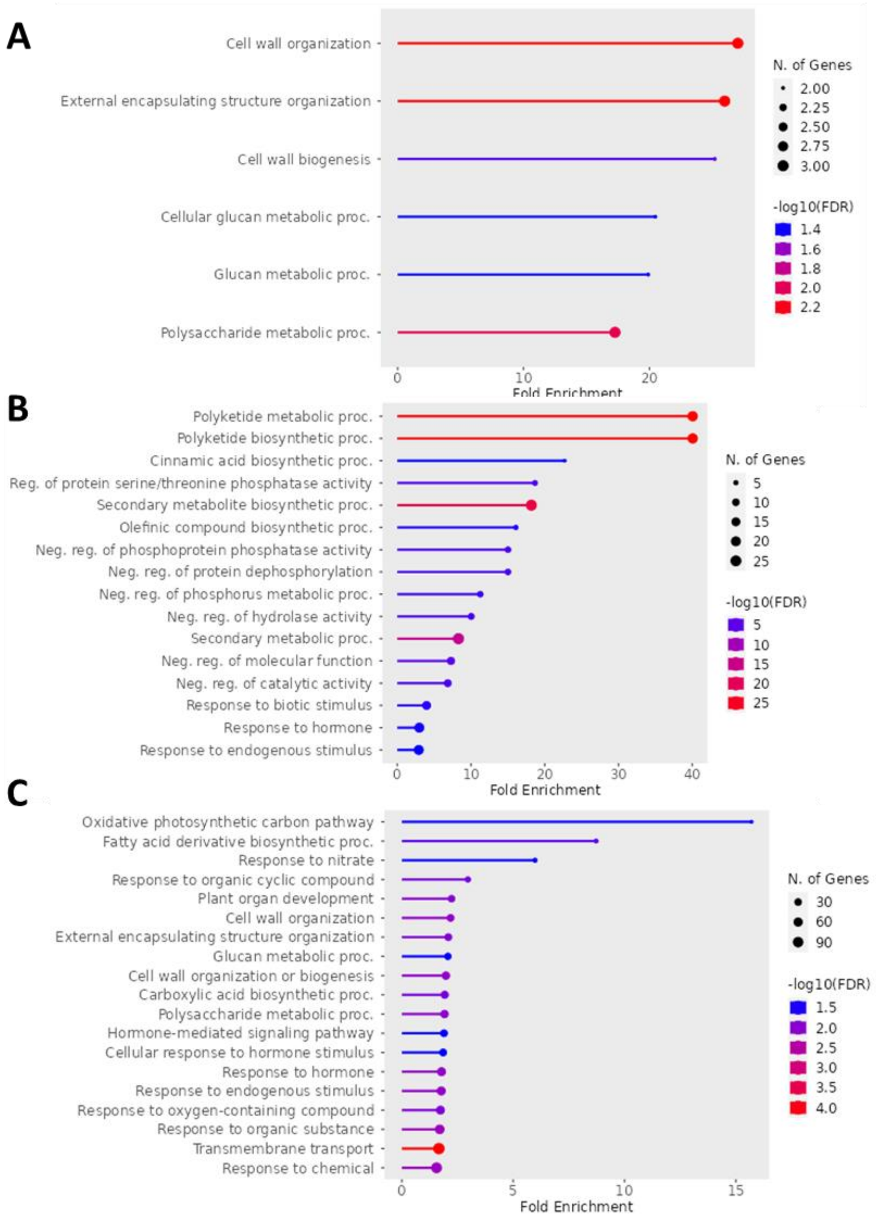

Fig. S2 GO enrichment analysis in Corvina and Glera tissues. Lolly plot ranking the enriched Biological Process (BP) GO terms within cumulative DEGs (not divided by stage) with FDR < 0,05 in the 4 cultivar/tissue combinations (Corvina pulp, Corvina skin, Glera pulp, Glera skin)**.** The *x* axis represents the Fold Enrichment, the color is function of the -log10(FDR), whilst the size of each dot is proportional to the number of enriched genes. (A) Enriched categories in Corvina pulp; (B) in Corvina skin; (C) and in Glera skin. Glera pulp did not give any enriched category.

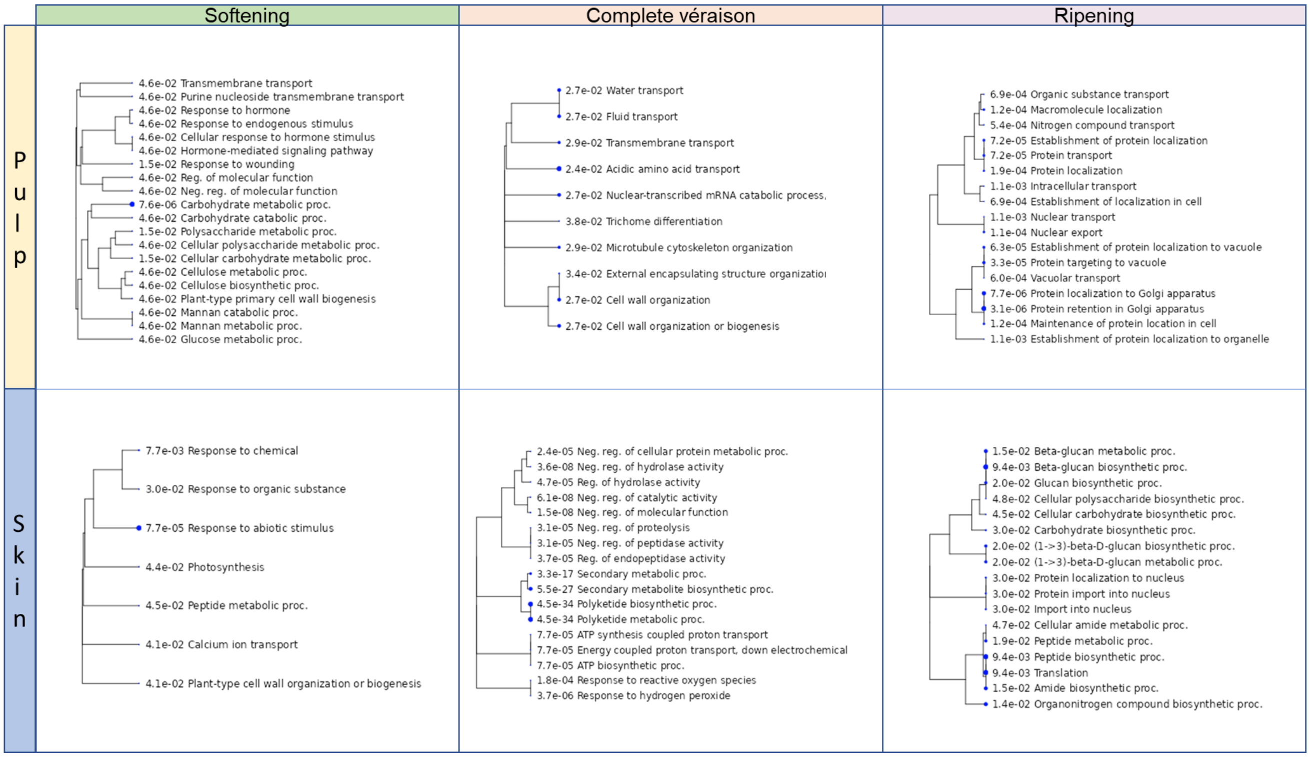

Fig. S3 GO enrichment analysis in Corvina DEGs. A hierarchical clustering tree summarizing the correlation among significant pathways obtained from the Enrichment analysis on DEGs identified in S, CV, and R stages in Corvina skin and flesh tissues with p-value < 0,01. Pathways with many shared genes are clustered together. Bigger dots indicate more significant P-values.

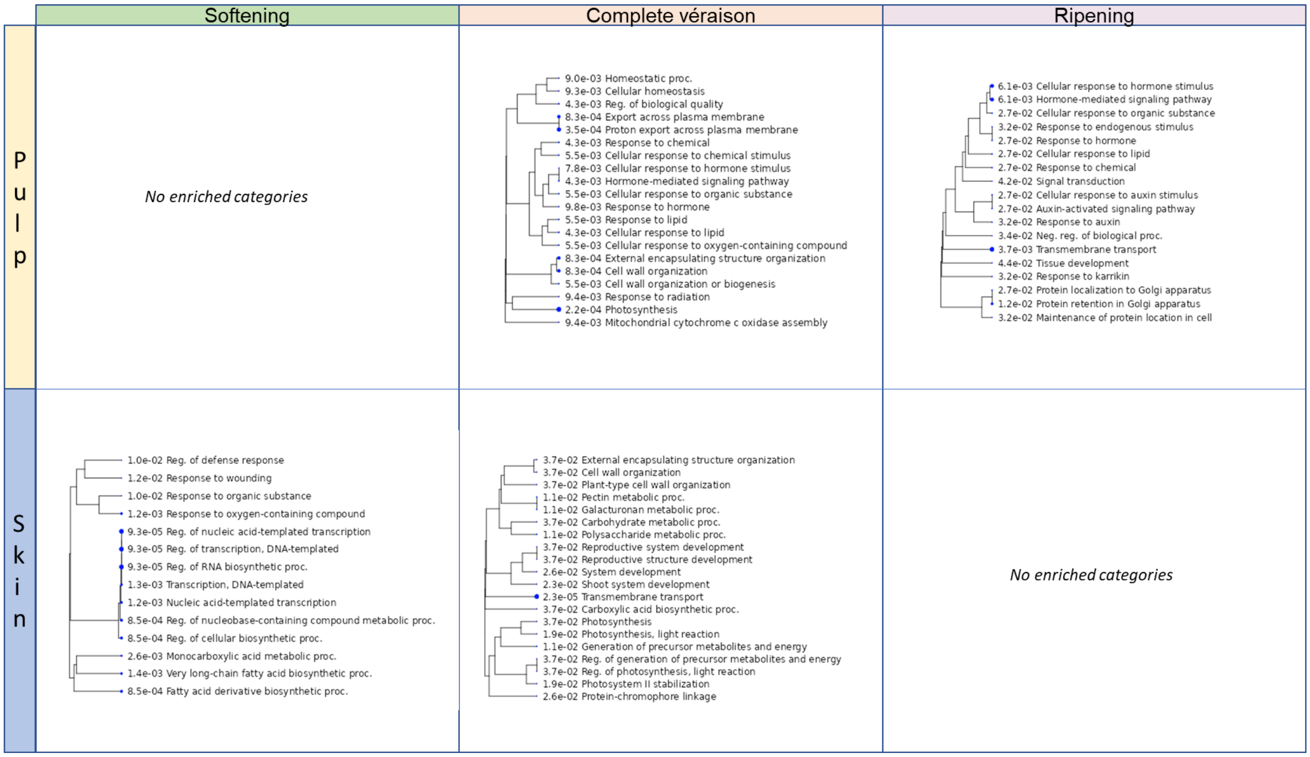

Fig. S4 GO enrichment analysis in Glera DEGs. A hierarchical clustering tree summarizing the correlation among significant pathways obtained from the Enrichment analysis on DEGs identified in S, CV, and R stages in Glera skin and flesh tissues with p-value < 0,01. Pathways with many shared genes are clustered together. Bigger dots indicate more significant P-values.

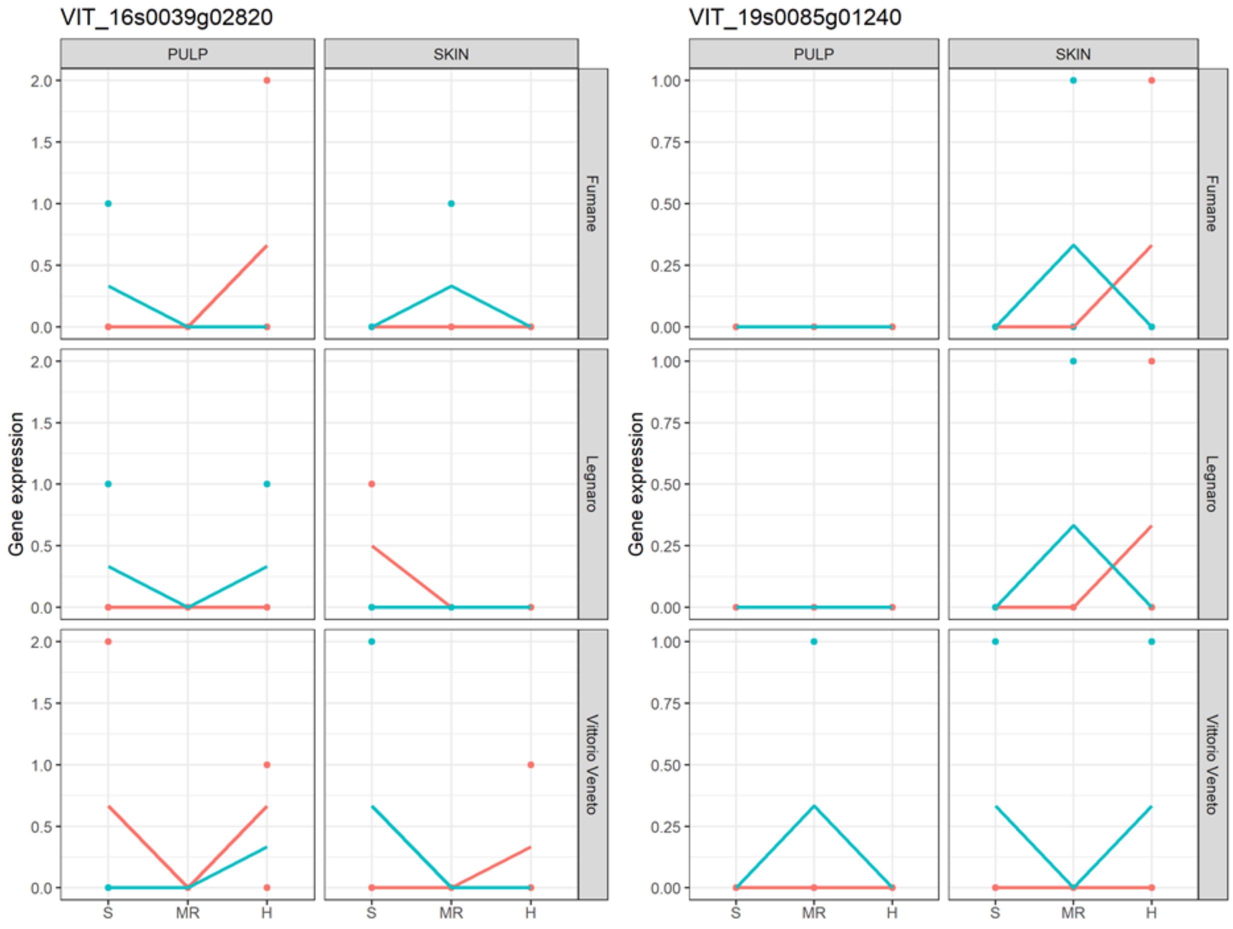

Fig. S5 Step 1, phase I; examples of removed genes. Examples of genes with uninteresting expression pattern screened out from statistical analysis according to the guidelines in Table S9, Step I, phase I.

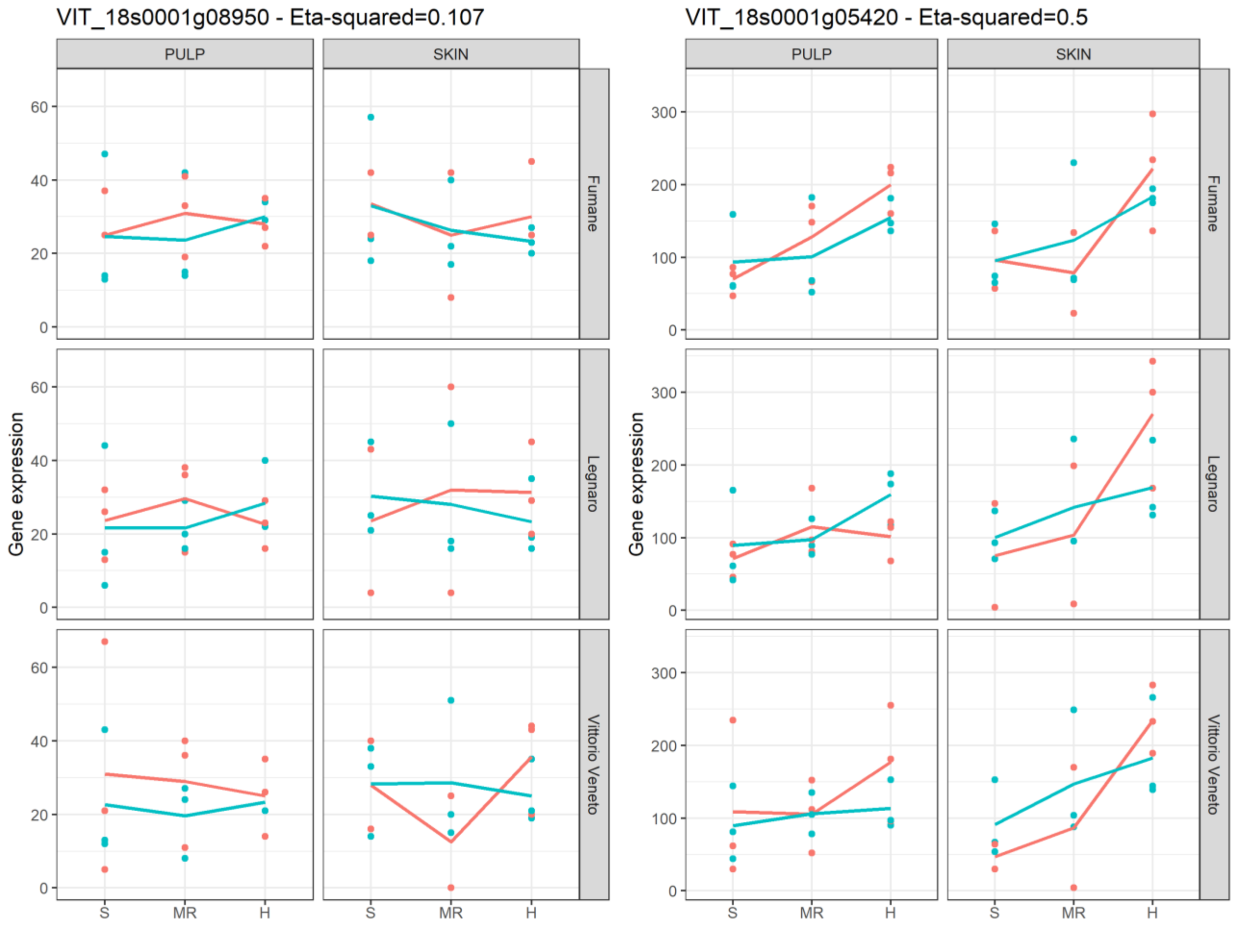

Fig. S6 Step 1, phase II; examples of removed genes. Examples of genes with uninteresting expression pattern screened out from statistical analysis according to the guidelines in Table S9, Step I, phase II.

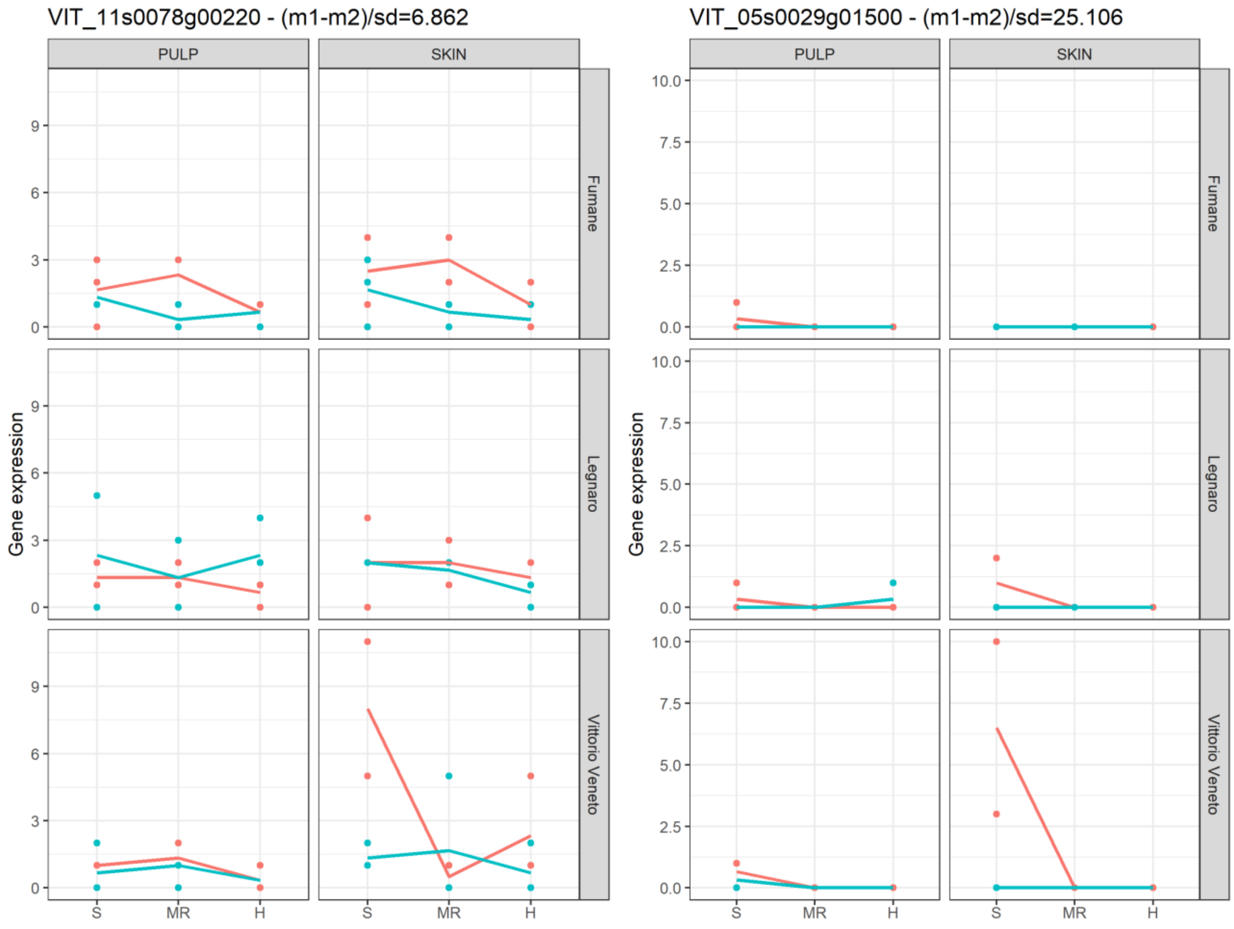

Fig. S7 Step 1, phase III; examples of removed genes. Examples of genes with uninteresting expression pattern screened out from statistical analysis according to the guidelines in Table S9, Step I, phase III.

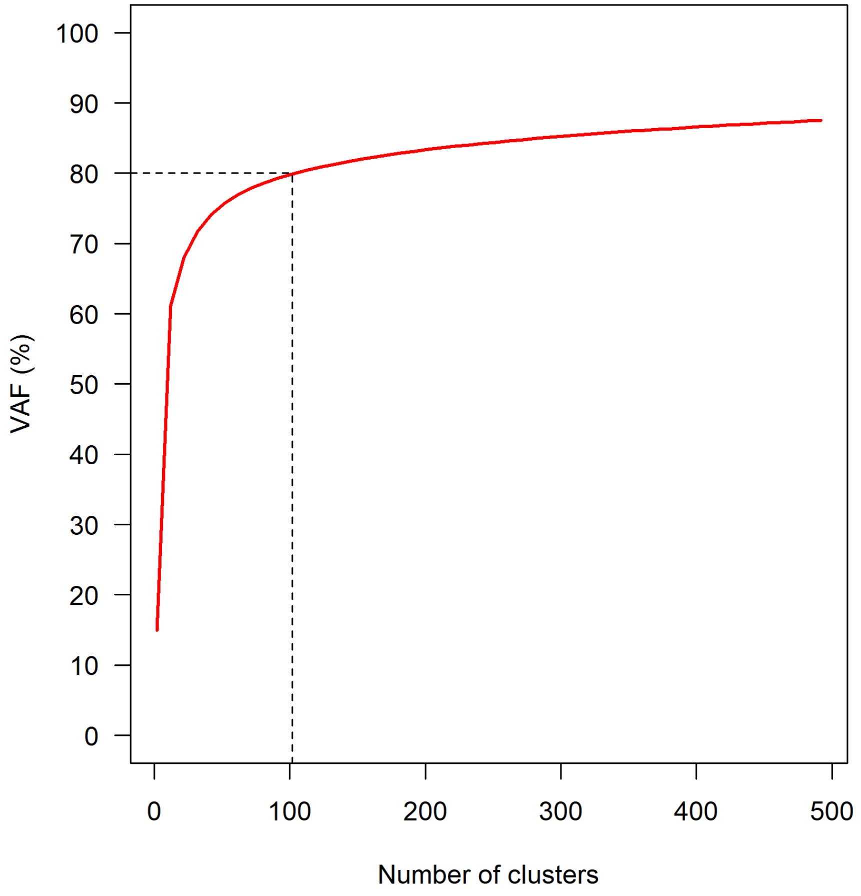

Fig. S8 Variance accounted for (VAF) by clusterization vs number of clusters.

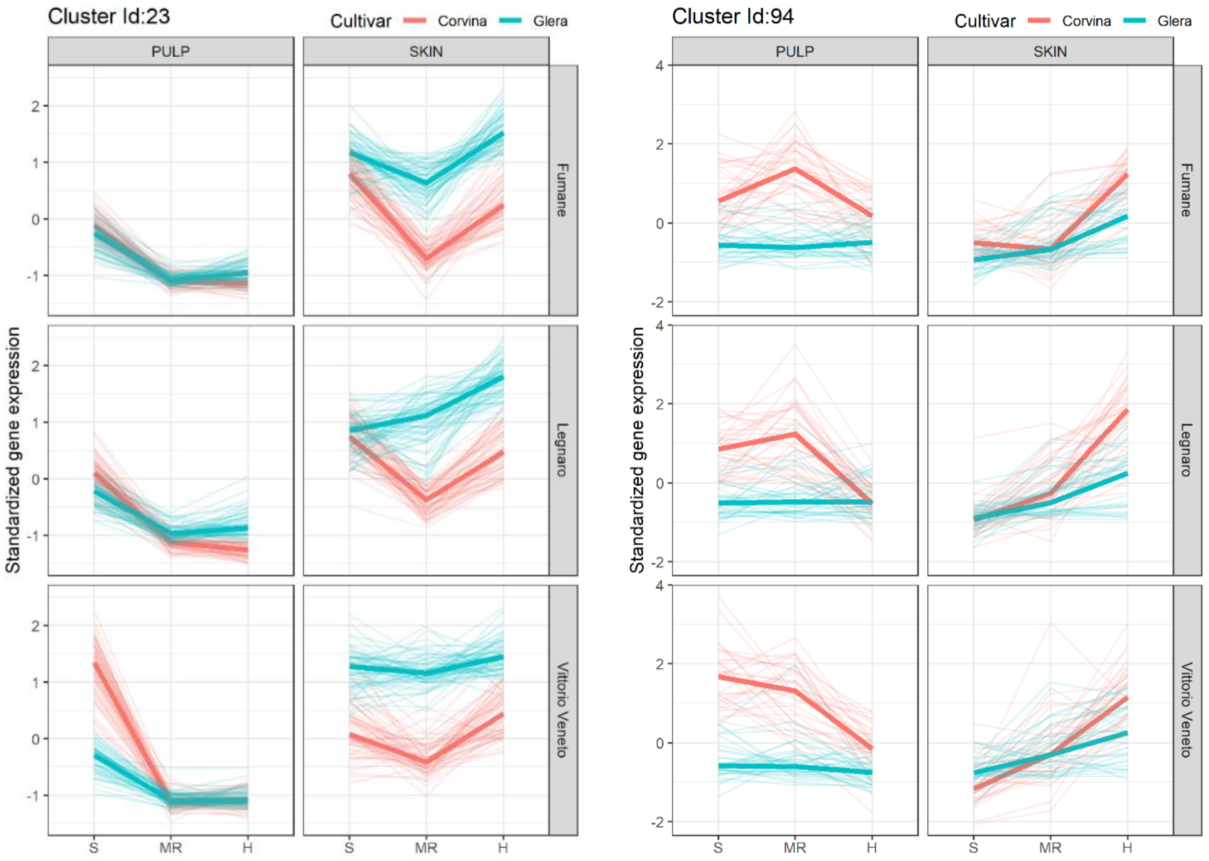

Fig. S9 Examples of clusters with high (left, cluster no. 23) and low (right, cluster no. 94) homogeneity index *Rc* (*Rc*=0.92 and *Rc*=0. 62, respectively). Thin lines represent the expressions of the genes belonging to each cluster; bold lines depict the median profiles Pc of clusters.

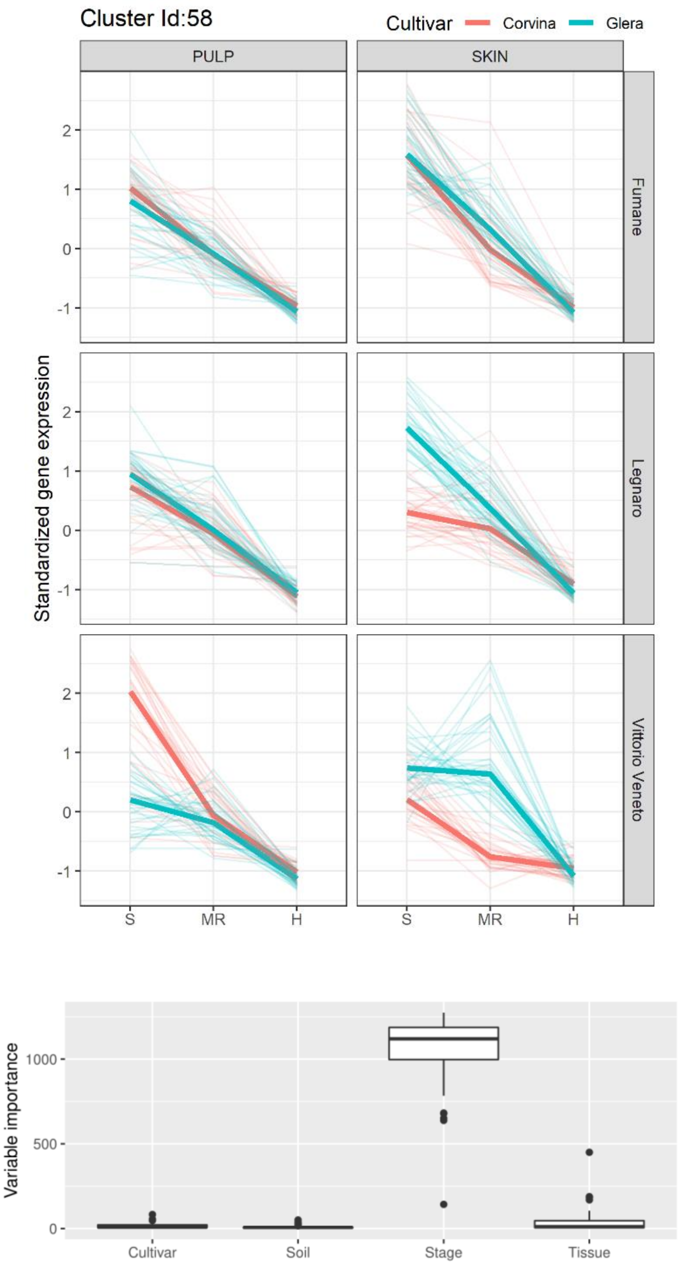

Fig. S10 Expression profiles (top) and boxplots of VIMs (bottom) for cluster no. 58.

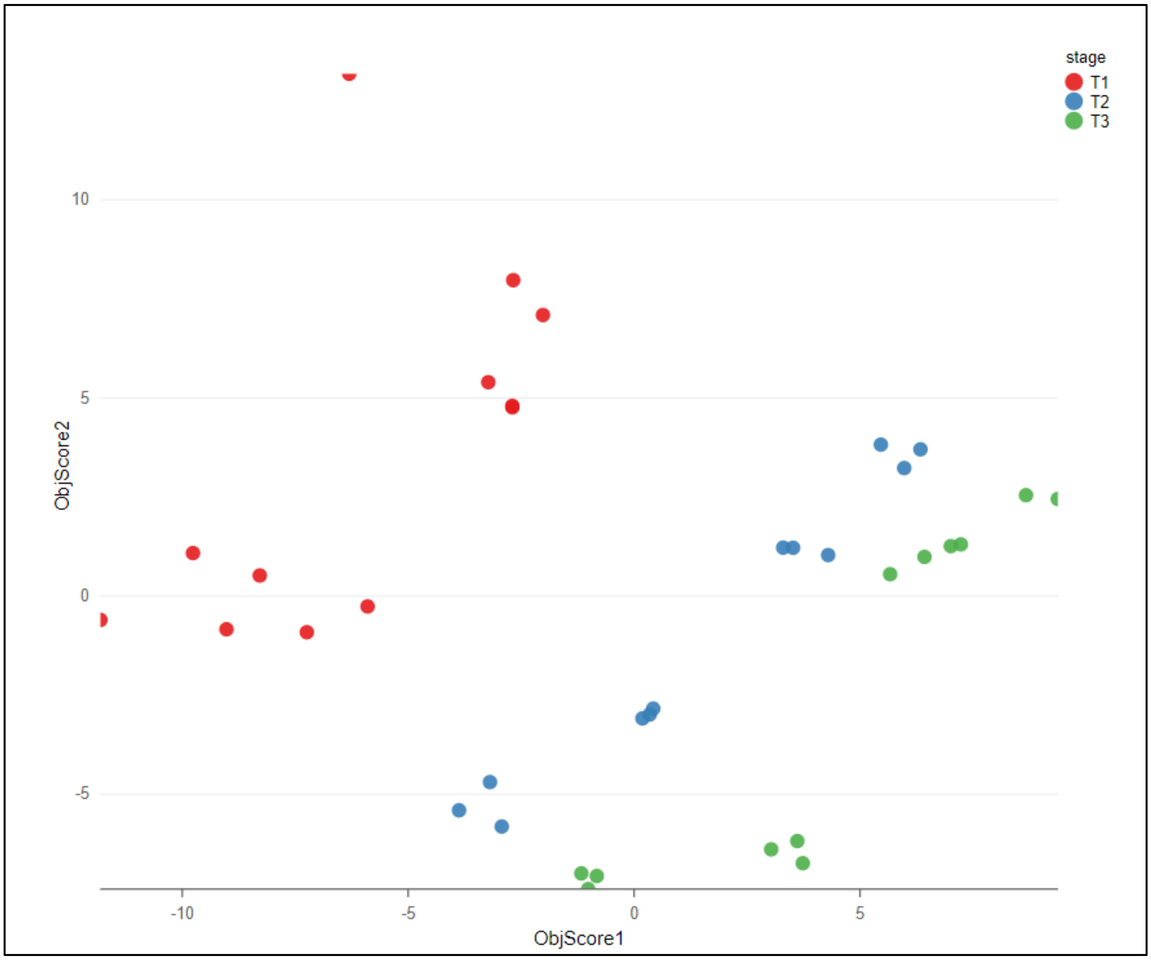

Fig. S11 Selected two-dimensional object scores plots of the 36 experimental conditions. Points are colored according to the covariates=stage. T1: softening; T2: Complete-véraison; T3: ripening)

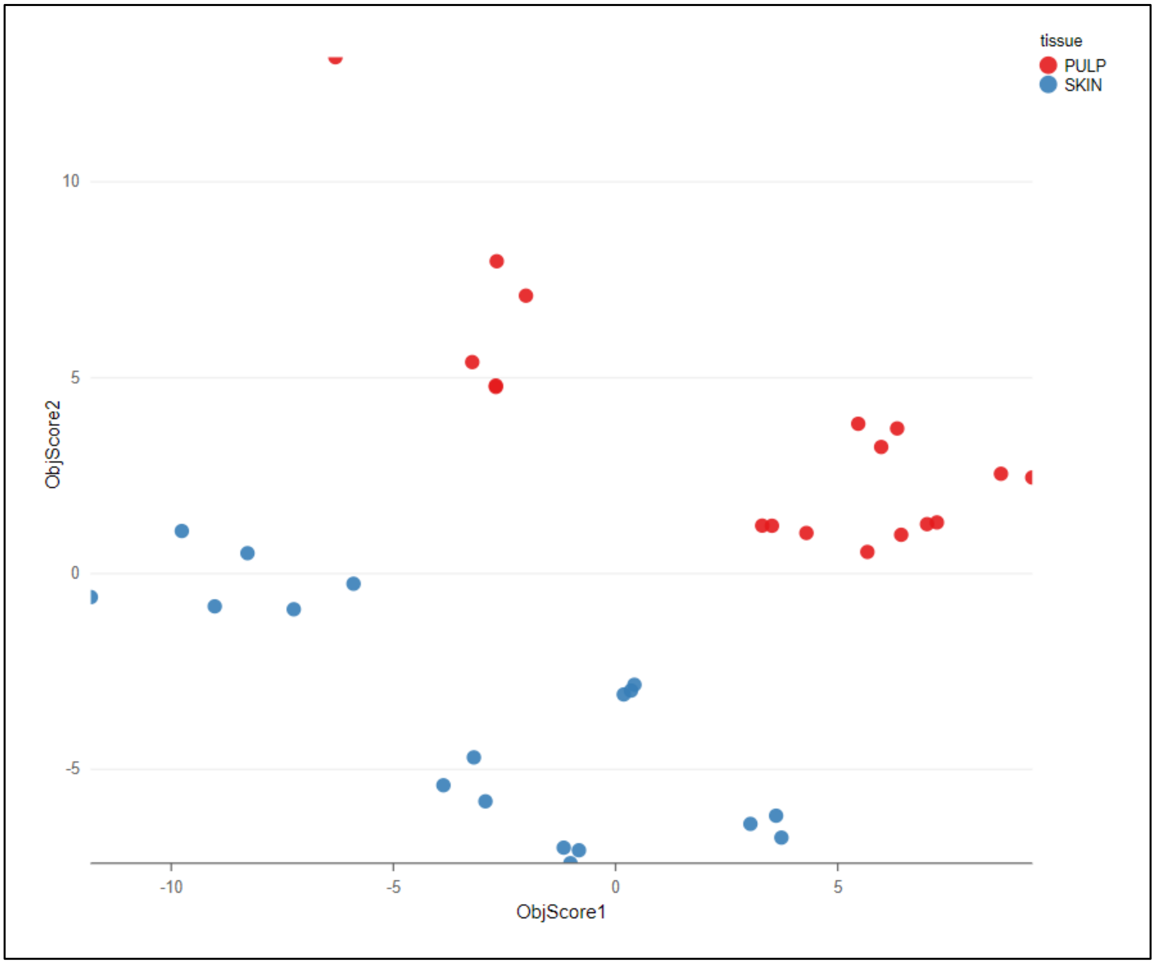

Fig. S12 Selected two-dimensional object scores plots of the 36 experimental conditions (points colored according to the covariates)

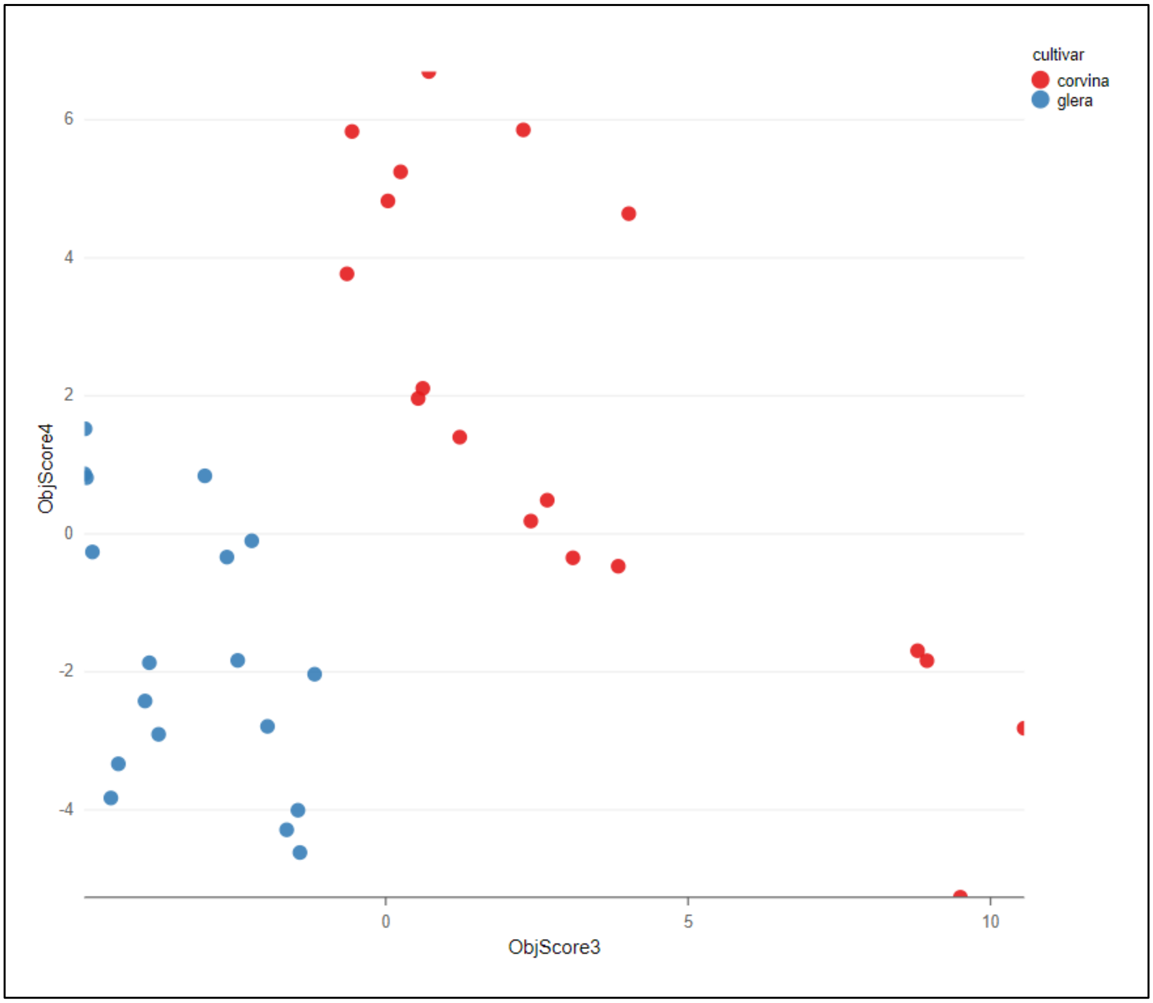

Fig. S13 Selected two-dimensional object scores plots of the 36 experimental conditions (points colored according to the covariates).

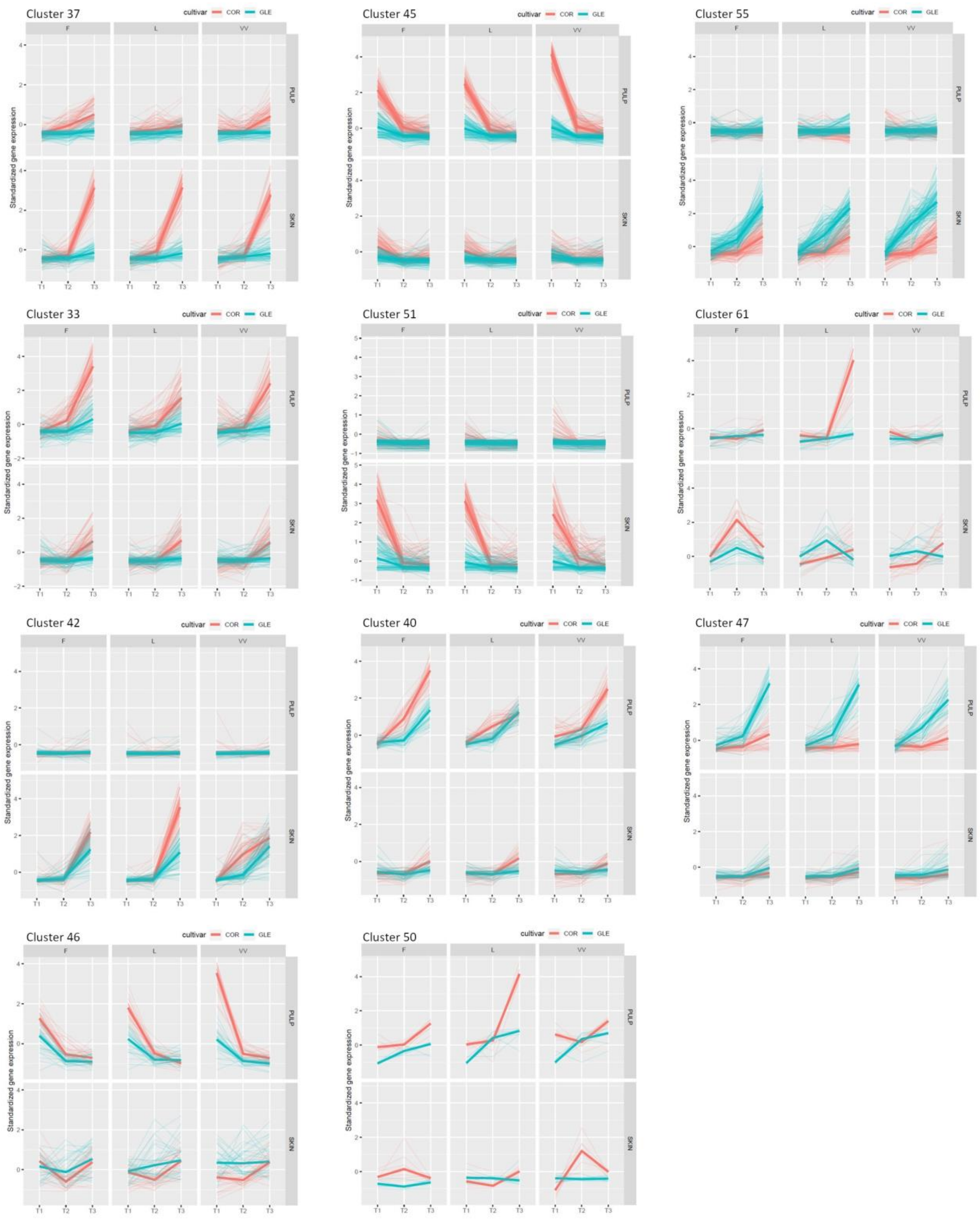

Fig. S15 Graphical representation of the 11 soil specific cluster identified in the VIM analyses.

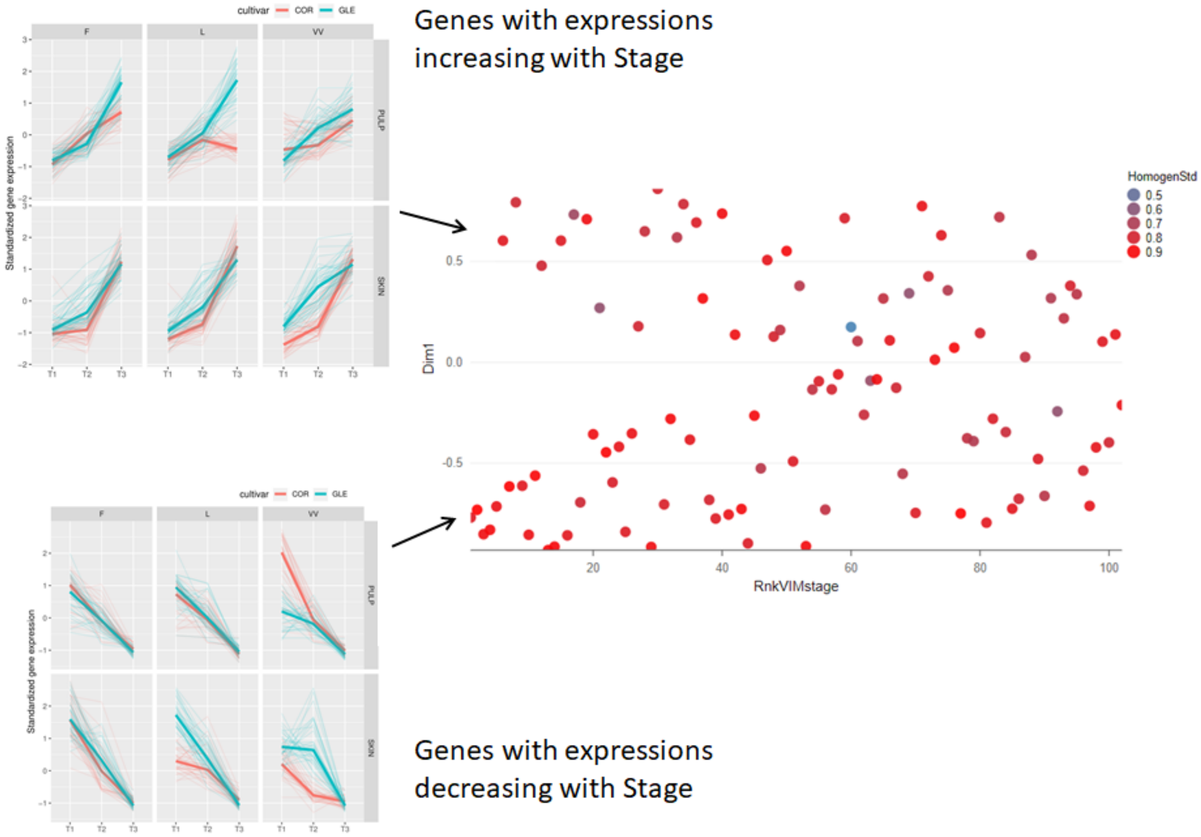

Figure S15 – Scatterplot of the 102 clusters according to Rank in VIM^{c}_{Stage} and loading in the first Principal Component (points coloured by cluster homogeneity index R_c).

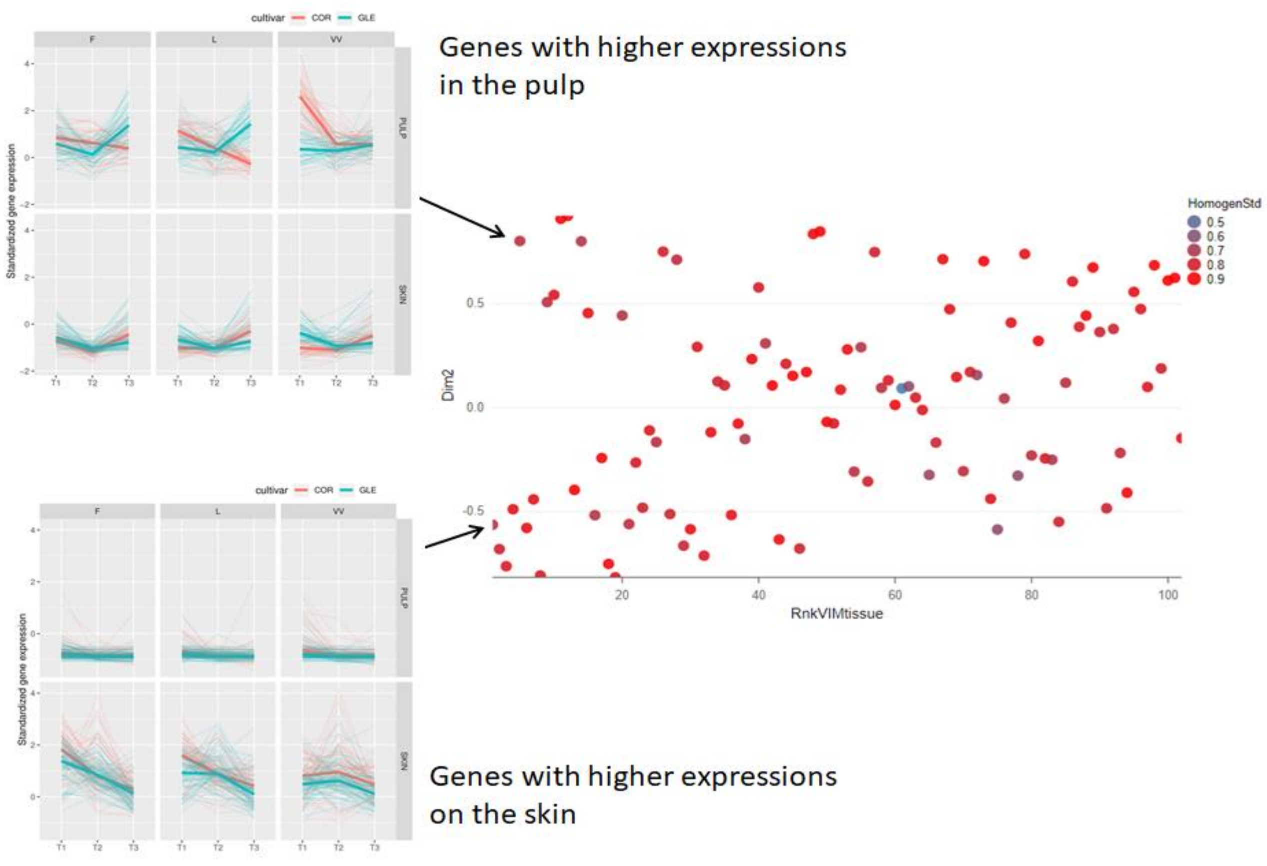

Figure S16 – Scatterplot of the 102 clusters according to Rank in VIM_{Tissue}^{c} and loading in the second Principal Component (points coloured by cluster homogeneity index R_c).

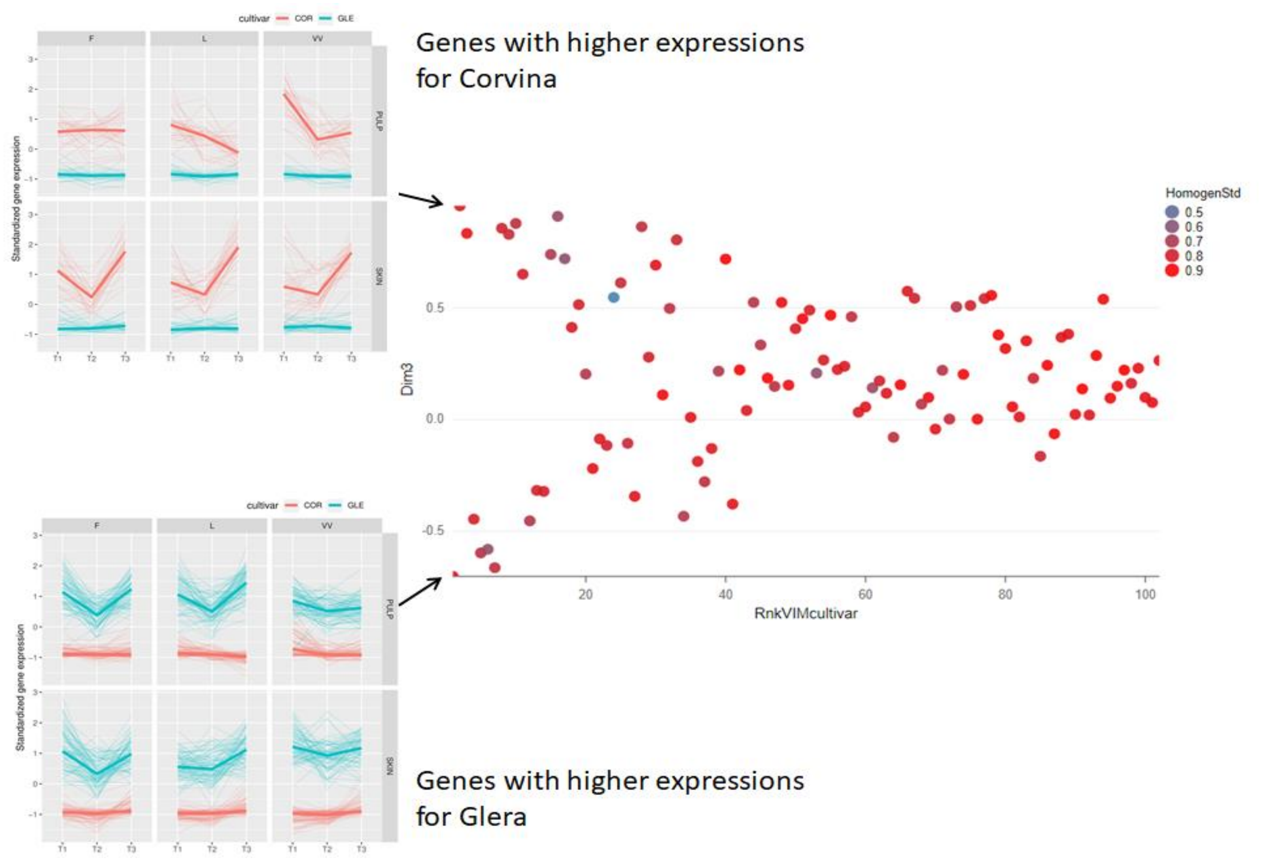

Figure S17 – Scatterplot of the 102 clusters according to Rank in $VIM^{c}_{Cultivar}$ and loading in the third Principal Component (points coloured by cluster homogeneity index R_c).

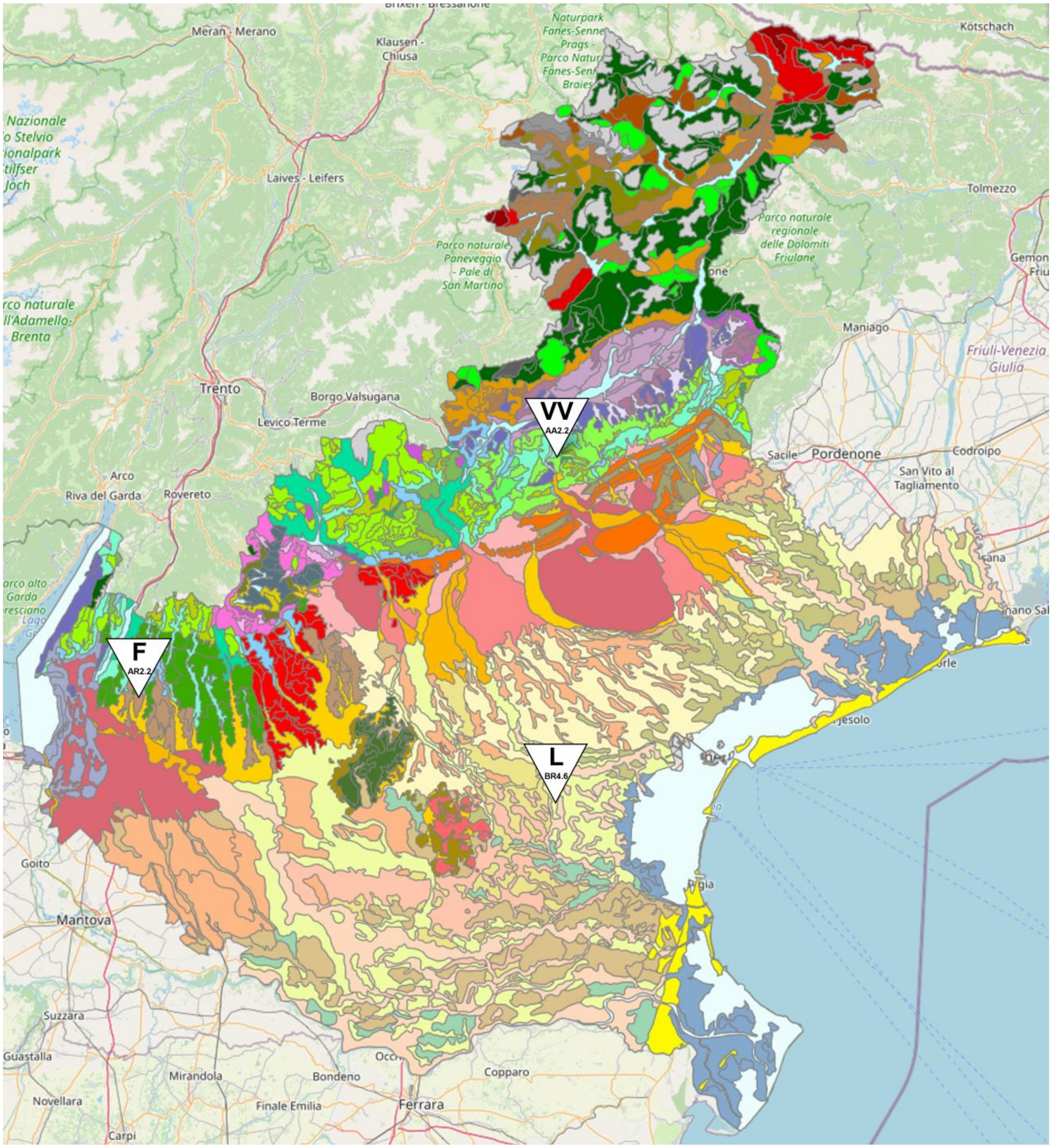

Fig. S18 Soil map of Veneto soils in scale 1:250.000. The figure, downloaded and modified from the ARPAV (Agenzia Regionale per la Prevenzione e Protezione Ambientale del Veneto) website (https://gaia.arpa.veneto.it/maps/271), shows the positions of the localities from which the soils were collected and the codes relating to the classification reported in Methods S1.

Table S1 Phenological development in 2017 vintage in both Glera and Corvina varieties. Statistical analyses were performed by means of non-parametrical Kruskall-Wallis test, P<0.05. Values represent the average of 12 plants (4 plants x 3 replicates).

Date	Significance	p value	F	VV	L	Significance	p value	F	VV	L
	Glera					Corvina				
03_03_2017	ns	0,264	2,0	2,0	1,9	**	0,002	2,0	2,0	1,5
07_03_2017	ns	1,000	2,0	2,0	2,0	ns	1,000	2,0	2,0	2,0
10_03_2017	ns	1,000	2,0	2,0	2,0	ns	1,000	2,0	2,0	2,0
16_03_2017	ns	0,164	2,2	2,0	2,0	ns	0,472	2,1	2,0	2,0
23_03_2017	*	0,016	3,0	2,6	2,3	ns	0,309	2,4	2,2	2,1
30_03_2017	ns	0,141	3,8	3,6	3,2	ns	0,202	2,7	2,3	2,4
06_04_2017	ns	0,193	8,3	7,2	7,0	*	0,022	5,0	3,3	3,8
13_04_2017	ns	0,486	12,3	12,3	11,9	**	0,001	10,5	5,8	6,5
19_04_2017	ns	0,853	13,9	13,8	13,6	**	0,006	12,7	8,9	9,5
28_04_2017	ns	0,877	14,9	14,9	14,7	ns	0,123	13,9	12,3	12,8
04_05_2017	ns	0,232	15,3	14,8	15,6	ns	0,271	14,5	13,6	13,4
09_05_2017	*	0,022	16,1	15,8	16,7	ns	0,118	15,7	14,6	15,0
17_05_2017	.	0,061	17,0	16,8	17,0	.	0,069	16,2	15,6	15,6
22_05_2017	ns	0,425	18,8	18,3	18,7	.	0,078	18,7	17,9	18,4
24_05_2017	*	0,044	21,6	21,0	22,9	.	0,081	20,4	18,6	20,4
26_05_2017	ns	0,300	25,0	24,3	25,0	ns	0,188	21,5	21,2	22,9
29_05_2017	ns	0,370	26,3	26,2	26,4	*	0,011	26,0	24,4	26,0
31_05_2017	ns	0,461	27,5	27,2	27,4	**	0,008	26,6	25,7	26,5
08_06_2017	*	0,014	29,3	29,4	29,9	**	0,001	29,3	27,7	28,8
14_06_2017	*	0,043	31,5	31,1	31,6	***	0,001	31,7	30,5	31,5
21_06_2017	*	0,026	32,1	32,0	32,6	**	0,005	32,4	32,1	33,0
29_06_2017	**	0,003	32,1	32,1	32,7	.	0,068	32,7	32,4	33,0
05_07_2017	***	0,000	32,4	32,1	33,0	ns	1,000	33,0	33,0	33,0
11_07_2017	ns	1,000	33,0	33,0	33,0	ns	1,000	33,0	33,0	33,0
18_07_2017	ns	1,000	33,0	33,0	33,0	ns	1,000	33,0	33,0	33,0
25_07_2017	**	0,006	33,8	33,3	33,9	***	0,001	34,3	33,0	34,1
26_07_2017	na	na	34,4	33,8	34,4	***	0,000	34,8	33,0	34,7
28_07_2017	***	0,000	35,1	34,3	35,0	***	0,000	35,0	33,6	34,8
31_07_2017	***	0,000	35,5	34,8	35,6	*	0,018	35,3	34,6	35,2
02_08_2017	***	0,000	35,8	35,3	35,9	***	0,000	35,5	34,7	35,6
04_08_2017	***	0,000	35,9	35,3	35,9	***	0,001	35,9	35,2	35,8
08_08_2017	***	0,000	36,0	35,5	36,0	***	0,000	35,9	35,4	35,9
16_08_2017	ns	0,651	36,8	36,7	36,7	*	0,017	36,8	36,4	36,5
30_08_2017	ns	0,587	37,4	37,3	37,4	**	0,004	37,5	36,9	37,3
06_09_2017	.	0,078	37,7	37,4	37,8	.	0,085	37,7	37,2	37,4
13_09_2017	na	na	na	na	na	*	0,021	37,7	37,1	37,4

Table S2 Phenological development in 2018 vintage in both Glera and Corvina varieties. Statistical analyses were performed by means of non-parametrical Kruskall-Wallis test, P<0.05. Values represent the average of 12 plants (4 plants x 3 replicates).

Date	Significance	p-value	F	VV	L	Significance	p-value	F	VV	L
	Glera					Corvina				
14_03_2018	.	0,082	1,5	1,8	1,8	*	0,026	1,3	1,5	1,7
21_03_2018	ns	0,598	2,0	2,0	2,0	ns	0,680	1,7	1,7	1,8
27_03_2018	ns	0,465	2,6	2,7	2,4	ns	0,314	2,0	2,0	2,2
30_03_2018	ns	0,368	3,0	3,0	2,9	ns	1,000	2,0	2,0	2,0
03_04_2018	ns	0,306	3,4	3,2	3,2	ns	0,899	2,5	2,5	2,4
06_04_2018	*	0,041	4,3	4,0	3,9	ns	0,268	2,9	2,6	2,7
10_04_2018	ns	1,000	5,0	5,0	5,0	ns	0,183	3,5	3,0	3,4
13_04_2018	ns	1,000	5,0	5,0	5,0	*	0,042	4,8	4,1	4,4
17_04_2018	ns	0,593	10,0	9,8	9,5	*	0,011	7,5	6,1	6,2
20_04_2018	ns	0,245	11,7	11,4	10,9	.	0,076	10,2	9,4	9,4
24_04_2018	ns	0,444	13,3	12,8	13,3	ns	0,801	11,8	11,6	11,6
27_04_2018	ns	0,346	15,0	14,8	14,9	ns	0,274	12,0	11,7	12,0
02_05_2018	ns	0,144	15,9	15,3	15,9	ns	0,970	12,6	12,7	12,6
07_05_2018	.	0,061	18,0	17,7	18,0	ns	0,188	16,6	16,2	16,5
10_05_2018	ns	0,417	18,0	17,9	18,0	ns	0,272	17,1	16,8	17,0
14_05_2018	ns	1,000	18,0	18,0	18,0	ns	0,427	18,2	18,0	18,1
17_05_2018	*	0,041	19,2	18,4	18,4	**	0,009	19,8	18,4	19,1
21_05_2018	**	0,004	25,7	25,1	25,0	ns	0,108	25,5	24,5	25,3
24_05_2018	*	0,020	26,4	26,3	26,9	.	0,064	26,3	25,7	26,4
28_05_2018	ns	0,361	27,6	27,3	27,4	.	0,065	27,5	26,9	27,6
31_05_2018	ns	0,279	29,0	29,0	28,9	ns	0,143	28,9	28,4	28,9
08_06_2018	ns	0,435	30,9	31,0	31,0	ns	0,799	31,1	31,0	30,9
14_06_2018	***	0,001	31,4	31,1	32,0	ns	0,216	32,0	31,8	31,8
20_06_2018	*	0,033	31,6	31,4	31,9	ns	0,261	31,9	31,7	32,0
28_06_2018	**	0,006	31,8	31,5	32,2	ns	0,748	32,3	32,5	32,4
03_07_2018	***	0,000	32,2	32,2	32,9	ns	0,279	33,0	33,0	32,9
13_07_2018	ns	0,243	32,7	32,6	32,9	ns	1,000	33,0	33,0	33,0
20_07_2018	ns	0,316	32,8	32,8	33,0	ns	0,279	33,0	33,0	33,1
23_07_2018	*	0,012	34,3	34,0	34,6	*	0,011	34,7	34,1	34,7
27_07_2018	***	0,000	34,2	34,3	34,9	ns	0,627	34,9	34,8	35,0
30_07_2018	ns	0,301	35,4	35,2	35,5	ns	0,143	35,5	35,2	35,3
03_08_2018	ns	0,251	35,7	35,5	35,7	ns	0,270	35,8	35,9	35,7
08_08_2018	ns	0,167	35,9	35,7	35,8	ns	0,426	36,1	36,0	36,1
16_08_2018	ns	0,161	36,7	36,6	36,5	ns	0,121	36,6	36,4	36,4
29_08_2018	ns	0,762	37,4	37,3	37,3	.	0,064	37,1	36,9	36,8
11_09_2018	ns	0,785	37,4	37,3	37,4	ns	0,320	37,5	37,4	37,2

Table S3 ANOVA on physiological parameters measured in 2017. *=p value <0.05; **=p value <0.01; ***=p value >0.001.

Date	*Soil (S)*	*p value*	*Cv*	*p value*	*S x Cv*	*p value*	*F*	*VV*	*L*	*Glera*	*p value*	*F*	*VV*	*L*	*Corvina*	*p value*	*F*	*VV*	*L*
Shoot size																			
03/03/17	***	1,0E-09	**	5,4E-03	ns	3,0E-01	c	b	a	***	3,5E-07	c	b	a	**	1,5E-03	b	ab	a
LAIE																			
09/06/17	*	2,3E-02	*	3,8E-02	ns	1,7E-01	b	ab	a	ns	1,6E-01				*	2,7E-02	b	a	ab
Leaves number																			
17/05/17	ns	9,0E-01	*	1,1E-02	ns	2,6E-01				ns	6,3E-01				ns	3,7E-01			
24/05/17	ns	6,5E-01	ns	8,1E-01	ns	2,2E-01				ns	2,5E-01				ns	7,0E-01			
31/05/17	ns	6,2E-01	ns	3,6E-01	ns	2,4E-01				ns	2,8E-01				ns	6,2E-01			
08/06/17	ns	8,6E-01	ns	2,5E-01	ns	3,0E-01				ns	4,9E-01				ns	5,2E-01			
14/06/17	ns	9,2E-01	ns	8,2E-02	ns	5,7E-01				ns	7,7E-01				ns	6,1E-01			
21/06/17	ns	5,5E-01	*	2,9E-02	ns	8,3E-01				ns	6,5E-01				ns	7,1E-01			
29/06/17	ns	1,7E-01	ns	7,6E-02	ns	7,4E-01				ns	5,8E-01				ns	2,2E-01			
05/07/17	ns	6,9E-02	ns	1,9E-01	ns	9,5E-01				ns	2,4E-01				ns	2,8E-01			
11/07/17	ns	5,9E-02	ns	7,6E-02	ns	6,3E-01				ns	5,3E-01				ns	5,5E-02			
18/07/17	*	1,5E-02	ns	5,6E-02	ns	7,2E-01	b	a	ab	ns	3,4E-01				*	3,4E-02	b	a	ab
25/07/17	*	1,6E-02	ns	9,5E-02	ns	4,2E-01	b	a	ab	ns	5,3E-01				*	1,4E-02	b	a	ab
01/08/17	ns	7,3E-02	*	3,7E-02	ns	7,4E-01				ns	4,5E-01				ns	5,9E-02			
Shoot lenght																			
13/04/17	**	3,4E-03	***	2,1E-12	ns	7,4E-01	a	b	b	ns	1,8E-01				**	6,1E-03	a	b	b
19/04/17	**	9,4E-03	***	2,2E-09	ns	5,0E-01	a	b	ab	ns	2,7E-01				*	1,1E-02	a	b	ab
28/04/17	*	3,4E-02	***	1,5E-07	ns	3,6E-01	a	b	ab	ns	2,2E-01				*	4,8E-02	a	b	b
04/05/17	ns	1,1E-01	***	1,5E-05	ns	3,2E-01				ns	3,9E-01				ns	6,5E-02			
12/05/17	ns	2,1E-01	***	9,5E-05	ns	4,8E-01				ns	3,3E-01				ns	3,0E-01			
17/05/17	ns	3,7E-01	**	2,3E-03	ns	3,9E-01				ns	3,4E-01				ns	4,5E-01			
24/05/17	ns	6,7E-01	ns	1,4E-01	ns	4,7E-01				ns	4,2E-01				ns	8,5E-01			
31/05/17	ns	8,3E-01	ns	9,9E-01	ns	3,0E-01				ns	3,5E-01				ns	7,5E-01			
08/06/17	ns	5,1E-01	ns	2,0E-01	ns	3,6E-01				ns	2,9E-01				ns	7,0E-01			
14/06/17	ns	4,5E-01	ns	1,2E-01	ns	8,6E-01				ns	5,4E-01				ns	7,4E-01			
21/06/17	ns	2,9E-01	*	2,3E-02	ns	7,7E-01				ns	5,6E-01				ns	3,9E-01			
29/06/17	ns	1,0E-01	ns	1,1E-01	ns	6,0E-01				ns	5,0E-01				ns	1,4E-01			
05/07/17	*	3,5E-02	*	4,8E-02	ns	6,1E-01	b	a	ab	ns	4,7E-01				ns	6,0E-02			
11/07/17	*	3,3E-02	*	1,8E-02	ns	3,5E-01	b	a	a	ns	4,2E-01				*	3,3E-02	b	a	ab
18/07/17	*	1,0E-02	*	3,0E-02	ns	2,9E-01	b	a	a	ns	5,6E-01				**	7,1E-03	b	a	a
25/07/17	**	8,0E-03	ns	7,2E-02	ns	2,1E-01	b	a	ab	ns	6,1E-01				**	3,8E-03	b	a	a
01/08/17	*	2,6E-02	ns	5,2E-02	ns	4,6E-01	b	a	ab	ns	6,0E-01				*	2,1E-02	b	a	ab
Brix																			
08/08/17	***	1,6E-04	**	7,0E-03	ns	3,7E-01	a	b	a	*	3,4E-02	a	b	ab	**	4,2E-03	a	b	a
16/08/17	*	3,5E-02	***	1,4E-06	ns	1,2E-01	a	b	ab	ns	6,9E-01				*	2,7E-02	a	b	ab
30/08/17	*	1,2E-02	***	2,9E-13	*	2,9E-02	a	b	a	ns	7,7E-01				***	3,6E-04	a	b	a
06/09/17	**	4,4E-03	***	7,4E-13	ns	2,4E-01	a	b	a	*	1,2E-02	a	b	a	ns	8,6E-02			
13/09/17															**	3,0E-03	a	b	ab
pH																			
08/08/17	**	3,3E-03	**	7,3E-03	ns	1,9E-01	b	b	a	ns	7,5E-02				*	2,2E-02	ab	b	a
16/08/17	ns	2,1E-01	**	3,4E-03	ns	7,8E-01				ns	6,5E-01				ns	2,2E-01			
30/08/17	ns	7,3E-02	***	5,5E-04	ns	8,8E-01				ns	3,8E-01				ns	1,4E-01			
Acidity																			
08/08/17	ns	8,3E-02	*	4,4E-02	ns	7,0E-02				ns	7,5E-02				ns	1,5E-01			
16/08/17	ns	2,2E-01	ns	6,8E-02	ns	2,4E-01				ns	9,6E-01				ns	1,4E-01			
30/08/17	ns	8,3E-01	***	9,9E-04	ns	5,8E-01				ns	9,5E-01				ns	3,3E-01			
06/09/17										ns	5,8E-01								
13/09/17															ns	6,8E-01			
MI																			
08/08/17	**	1,8E-03	ns	3,6E-01	ns	5,4E-02	a	b	a	**	3,9E-03	ab	b	a	ns	5,5E-02			
16/08/17	ns	5,9E-02	ns	6,8E-01	ns	1,1E-01				ns	9,2E-01				ns	7,4E-02			
30/08/17	ns	4,5E-01	ns	7,1E-02	ns	2,3E-01				ns	9,0E-01				*	2,3E-02	a	b	ab
06/09/17										ns	8,0E-01				***				
13/09/17															ns	9,9E-02			
Berry weight																			
29/09/17	***	1,5E-04	ns	1,4E-01	ns	8,9E-01	a	b	a	**	3,4E-03	a	b	a	*	4,3E-02	ab	b	a
Bunches																			
31/05/17	***	6,0E-08	ns	1,1E-01	ns	2,7E-02	b	b	a	***	1,8E-06	b	b	a	*	1,3E-02	ab	b	a

Table S4 ANOVA on physiological parameters measured in 2018. *=p value <0.05; **=p value <0.01; ***=p value >0.001.

Date	*Soil (S)*	*p value*	*Cv*	*p value*	*S x Cv*	*p value*	*F*	*VV*	*L*	*Glera*	*p value*	*F*	*VV*	*L*	*Corvina*	*p value*	*F*	*VV*	*L*
Shoot size																			
27/4/18	**	5,9E-03	*	4,5E-02	ns	5,9E-01	b	a	a	*	1,3E-02	b	a	a	*	2,6E-02	b	ab	a
LAIE																			
24/05/18	ns	9,2E-01	***	1,1E-06	ns	6,0E-01				ns	6,3E-01				ns	8,8E-01			
20/06/18	ns	4,5E-01	ns	3,0E+00	ns	8,3E-01				ns	4,4E-01				ns	8,7E-01			
28/06/18	ns	5,3E-01	***	4,1E-04	ns	9,1E-01				ns	6,6E-01				ns	7,3E-01			
03/07/18	ns	1,8E-01	ns	1,4E-01	ns	6,4E-01				ns	1,3E-01				ns	7,8E-01			
13/07/18	ns	8,2E-01	*	1,4E-02	ns	4,9E-01				ns	4,3E-01				ns	9,2E-01			
20/07/18	ns	8,8E-01	*	1,4E-02	ns	6,0E-01				ns	6,5E-01				ns	8,1E-01			
Leaves number																			
14/05/18	ns	7,3E-01	**	6,9E-03	ns	8,0E-01				ns	8,3E-01				ns	6,7E-01			
24/05/18	ns	4,0E-01	ns	8,1E-01	ns	8,0E-02				ns	2,7E-01				ns	1,5E-01			
31/05/18	ns	1,2E-01	ns	6,0E-01	ns	8,3E-01				ns	2,9E-01				ns	3,4E-01			
08/06/18	ns	2,0E-01	ns	8,9E-01	ns	1,1E-01				.	6,4E-02				ns	8,9E-01			
14/06/18	ns	8,9E-01	ns	1,0E+00	ns	8,0E-01				ns	8,5E-01				ns	8,2E-01			
Shoot lenght																			
27/04/18	ns	9,8E-02	***	9,1E-09	ns	8,8E-01				ns	4,2E-01				ns	2,3E-01			
02/05/18	ns	2,9E-01	***	3,0E-04	ns	9,5E-01				ns	4,7E-01				ns	5,9E-01			
07/05/18	ns	6,1E-01	*	2,9E-02	ns	8,5E-01				ns	6,4E-01				ns	8,0E-01			
14/05/18	ns	4,0E-01	ns	8,9E-01	ns	6,8E-01				ns	3,3E-01				ns	9,0E-01			
24/05/18	ns	2,5E-01	ns	2,7E-01	ns	6,0E-01				ns	2,2E-01				ns	7,4E-01			
31/05/18	*	1,3E-02	ns	1,2E-01	ns	7,7E-01	b	b	a	ns	2,2E-01				ns	2,2E-01			
08/06/18	ns	1,5E-01	*	3,1E-02	ns	6,4E-01				ns	2,8E-01				ns	3,6E-01			
14/06/18	ns	5,4E-01	**	2,7E-03	ns	9,5E-01				ns	8,3E-01				ns	4,0E-01			
Brix																			
16/08/18	**	2,6E-03	**	5,3E-03	ns	5,2E-01	a	b	b	.	9,1E-02				*	1,0E-02	a	b	b
29/08/18	*	4,9E-02	***	5,4E-05	ns	3,8E-01	a	ab	b	ns	6,7E-01				ns	5,2E-02			
11/09/18	ns	3,3E-01	***	8,8E-12	ns	8,6E-01				ns	6,2E-01				ns	5,1E-01			
pH																			
16/08/18	ns	5,9E-01	ns	5,8E-01	ns	7,0E-01				ns	5,8E-01				ns	9,6E-01			
29/08/18	ns	1,3E-01	***	8,8E-05	ns	6,2E-01				ns	6,8E-01				ns	1,6E-01			
11/09/18	ns	8,5E-01	ns	2,6E-01	ns	9,8E-01				ns	9,1E-01				ns	9,2E-01			
Acidity																			
16/08/18	ns	4,9E-01	ns	1,5E-01	ns	4,7E-01				ns	6,1E-01				ns	4,7E-01			
29/08/18	ns	2,8E-01	***	1,8E-04	ns	7,9E-01				ns	3,1E-01				ns	7,3E-01			
11/09/18	ns	9,3E-01	ns	4,3E-01	ns	8,6E-01				ns	8,4E-01				ns	9,7E-01			
MI																			
16/08/18	*	1,5E-02	ns	3,1E-01	ns	2,7E-01	a	a	b	**	5,5E-03	a	a	b	ns	3,8E-01			
29/08/18	**	2,9E-03	***	3,2E-05	ns	3,9E-01	a	a	b	*	4,0E-02	ab	a	b	*	3,1E-02	a	ab	b
11/09/18	ns	7,8E-01	ns	3,3E-01	ns	4,2E-01				ns	3,9E-01				ns	8,7E-01			
Berry weight																			
11/09/18	ns	6,6E-02	***	5,8E-12		8,2E-02	a	ab	a	ns	8,2E-01				*	1,7E-02	a	b	a
Bunches																			
14/05/18	***	1,1E-04	ns	2,2E-01	ns	3,4E-01	b	b	a	***	7,5E-04	b	ab	a	ns	9,8E-02			

Table S5 Description of sample names used for the present study. Sample IDs are composed by the variety abbreviation (Glera: G, Corvina: C), followed by the indication of the tissue considered (Pulp: P; Skin; S), by the indication of the developmental stage (Softening: T1; Complete-véraison: T2, and Ripening: T3) and by the concrete caisson number (b1-b18).

Sample #	Sample ID	Cultivar	Tissue	Stage	Soil	Box	Replicate	Vintage
1	GPFT1b1	Glera	Pulp	Softening	Fumane	1	A	2018
2	GSFT1b1	Glera	Skin	Softening	Fumane	1	A	2018
3	GPFT2b1	Glera	Pulp	Complete véraison	Fumane	1	A	2018
4	GSFT2b1	Glera	Skin	Complete véraison	Fumane	1	A	2018
5	GPFT3b1	Glera	Pulp	Ripening	Fumane	1	A	2018
6	GSFT3b1	Glera	Skin	Ripening	Fumane	1	A	2018
7	GPVVT1b2	Glera	Pulp	Softening	V. veneto	2	A	2018
8	GSVVT1b2	Glera	Skin	Softening	V. veneto	2	A	2018
9	GPVVT2b2	Glera	Pulp	Complete véraison	V. veneto	2	A	2018
10	GSVVT2b2	Glera	Skin	Complete véraison	V. veneto	2	A	2018
11	GPVVT3b2	Glera	Pulp	Ripening	V. veneto	2	A	2018
12	GSVVT3b2	Glera	Skin	Ripening	V. veneto	2	A	2018
13	GPLT1b3	Glera	Pulp	Softening	Legnaro	3	A	2018
14	GSLT1b3	Glera	Skin	Softening	Legnaro	3	A	2018
15	GPLT2b3	Glera	Pulp	Complete véraison	Legnaro	3	A	2018
16	GSLT2b3	Glera	Skin	Complete véraison	Legnaro	3	A	2018
17	GPLT3b3	Glera	Pulp	Ripening	Legnaro	3	A	2018
18	GSLT3b3	Glera	Skin	Ripening	Legnaro	3	A	2018
19	CPFT1b4	Corvina	Pulp	Softening	Fumane	4	A	2018
20	CSFT1b4	Corvina	Skin	Softening	Fumane	4	A	2018
21	CPFT2b4	Corvina	Pulp	Complete véraison	Fumane	4	A	2018
22	CSFT2b4	Corvina	Skin	Complete véraison	Fumane	4	A	2018
23	CPFT3b4	Corvina	Pulp	Ripening	Fumane	4	A	2018
24	CSFT3b4	Corvina	Skin	Ripening	Fumane	4	A	2018
25	CPVVT1b5	Corvina	Pulp	Softening	V. veneto	5	A	2018
26	CSVVT1b5	Corvina	Skin	Softening	V. veneto	5	A	2018
27	CPVVT2b5	Corvina	Pulp	Complete véraison	V. veneto	5	A	2018
28	CSVVT2b5	Corvina	Skin	Complete véraison	V. veneto	5	A	2018
29	CPVVT3b5	Corvina	Pulp	Ripening	V. veneto	5	A	2018
30	CSVVT3b5	Corvina	Skin	Ripening	V. veneto	5	A	2018
31	CPLT1b6	Corvina	Pulp	Softening	Legnaro	6	A	2018
32	CSLT1b6	Corvina	Skin	Softening	Legnaro	6	A	2018
33	CPLT2b6	Corvina	Pulp	Complete véraison	Legnaro	6	A	2018
34	CSLT2b6	Corvina	Skin	Complete véraison	Legnaro	6	A	2018
35	CPLT3b6	Corvina	Pulp	Ripening	Legnaro	6	A	2018
36	CSLT3b6	Corvina	Skin	Ripening	Legnaro	6	A	2018
37	GPLT1b7	Glera	Pulp	Softening	Legnaro	7	B	2018
38	GSLT1b7	Glera	Skin	Softening	Legnaro	7	B	2018
39	GPLT2b7	Glera	Pulp	Complete véraison	Legnaro	7	B	2018
40	GSLT2b7	Glera	Skin	Complete véraison	Legnaro	7	B	2018
41	GPLT3b7	Glera	Pulp	Ripening	Legnaro	7	B	2018
42	GSLT3b7	Glera	Skin	Ripening	Legnaro	7	B	2018
43	GPFT1b8	Glera	Pulp	Softening	Fumane	8	B	2018
44	GSFT1b8	Glera	Skin	Softening	Fumane	8	B	2018
45	GPFT2b8	Glera	Pulp	Complete véraison	Fumane	8	B	2018
46	GSFT2b8	Glera	Skin	Complete véraison	Fumane	8	B	2018
47	GPFT3b8	Glera	Pulp	Ripening	Fumane	8	B	2018
48	GSFT3b8	Glera	Skin	Ripening	Fumane	8	B	2018
49	GPVVT1b9	Glera	Pulp	Softening	V. veneto	9	B	2018
50	GSVVT1b9	Glera	Skin	Softening	V. veneto	9	B	2018
51	GPVVT2b9	Glera	Pulp	Complete véraison	V. veneto	9	B	2018
52	GSVVT2b9	Glera	Skin	Complete véraison	V. veneto	9	B	2018
53	GPVVT3b9	Glera	Pulp	Ripening	V. veneto	9	B	2018
54	GSVVT3b9	Glera	Skin	Ripening	V. veneto	9	B	2018
55	CPLT1b10	Corvina	Pulp	Softening	Legnaro	10	B	2018
56	CSLT1b10	Corvina	Skin	Softening	Legnaro	10	B	2018
57	CPLT2b10	Corvina	Pulp	Complete véraison	Legnaro	10	B	2018

58	CSLT2b10	Corvina	Skin	Complete véraison	Legnaro	10	B	2018
59	CPLT3b10	Corvina	Pulp	Ripening	Legnaro	10	B	2018
60	CSLT3b10	Corvina	Skin	Ripening	Legnaro	10	B	2018
61	CPFT1b11	Corvina	Pulp	Softening	Fumane	11	B	2018
62	CSFT1b11	Corvina	Skin	Softening	Fumane	11	B	2018
63	CPFT2b11	Corvina	Pulp	Complete véraison	Fumane	11	B	2018
64	CSFT2b11	Corvina	Skin	Complete véraison	Fumane	11	B	2018
65	CPFT3b11	Corvina	Pulp	Ripening	Fumane	11	B	2018
66	CSFT3b11	Corvina	Skin	Ripening	Fumane	11	B	2018
67	CPVVT1b12	Corvina	Pulp	Softening	V. veneto	12	B	2018
68	CSVVT1b12	Corvina	Skin	Softening	V. veneto	12	B	2018
69	CPVVT2b12	Corvina	Pulp	Complete véraison	V. veneto	12	B	2018
70	CSVVT2b12	Corvina	Skin	Complete véraison	V. veneto	12	B	2018
71	CPVVT3b12	Corvina	Pulp	Ripening	V. veneto	12	B	2018
72	CSVVT3b12	Corvina	Skin	Ripening	V. veneto	12	B	2018
73	GPVVT1b13	Glera	Pulp	Softening	V. veneto	13	C	2018
74	GSVVT1b13	Glera	Skin	Softening	V. veneto	13	C	2018
75	GPVVT2b13	Glera	Pulp	Complete véraison	V. veneto	13	C	2018
76	GSVVT2b13	Glera	Skin	Complete véraison	V. veneto	13	C	2018
77	GPVVT3b13	Glera	Pulp	Ripening	V. veneto	13	C	2018
78	GSVVT3b13	Glera	Skin	Ripening	V. veneto	13	C	2018
79	GPLT1b14	Glera	Pulp	Softening	Legnaro	14	C	2018
80	GSLT1b14	Glera	Skin	Softening	Legnaro	14	C	2018
81	GPLT2b14	Glera	Pulp	Complete véraison	Legnaro	14	C	2018
82	GSLT2b14	Glera	Skin	Complete véraison	Legnaro	14	C	2018
83	GPLT3b14	Glera	Pulp	Ripening	Legnaro	14	C	2018
84	GSLT3b14	Glera	Skin	Ripening	Legnaro	14	C	2018
85	GPFT1b15	Glera	Pulp	Softening	Fumane	15	C	2018
86	GSFT1b15	Glera	Skin	Softening	Fumane	15	C	2018
87	GPFT2b15	Glera	Pulp	Complete véraison	Fumane	15	C	2018
88	GSFT2b15	Glera	Skin	Complete véraison	Fumane	15	C	2018
89	GPFT3b15	Glera	Pulp	Ripening	Fumane	15	C	2018
90	GSFT3b15	Glera	Skin	Ripening	Fumane	15	C	2018
91	CPVVT1b16	Corvina	Pulp	Softening	V. veneto	16	C	2018
92	CSVVT1b16	Corvina	Skin	Softening	V. veneto	16	C	2018
93	CPVVT2b16	Corvina	Pulp	Complete véraison	V. veneto	16	C	2018
94	CSVVT2b16	Corvina	Skin	Complete véraison	V. veneto	16	C	2018
95	CPVVT3b16	Corvina	Pulp	Ripening	V. veneto	16	C	2018
96	CSVVT3b16	Corvina	Skin	Ripening	V. veneto	16	C	2018
97	CPLT1b17	Corvina	Pulp	Softening	Legnaro	17	C	2018
98	CSLT1b17	Corvina	Skin	Softening	Legnaro	17	C	2018
99	CPLT2b17	Corvina	Pulp	Complete véraison	Legnaro	17	C	2018
100	CSLT2b17	Corvina	Skin	Complete véraison	Legnaro	17	C	2018
101	CPLT3b17	Corvina	Pulp	Ripening	Legnaro	17	C	2018
102	CSLT3b17	Corvina	Skin	Ripening	Legnaro	17	C	2018
103	CPFT1b18	Corvina	Pulp	Softening	Fumane	18	C	2018
104	CSFT1b18	Corvina	Skin	Softening	Fumane	18	C	2018
105	CPFT2b18	Corvina	Pulp	Complete véraison	Fumane	18	C	2018
106	CSFT2b18	Corvina	Skin	Complete véraison	Fumane	18	C	2018
107	CPFT3b18	Corvina	Pulp	Ripening	Fumane	18	C	2018
108	CSFT3b18	Corvina	Skin	Ripening	Fumane	18	C	2018

Table S6 Mapping statistics for 108 samples analyzed.

Sample ID	Total fragments	Mapped	Mapped (%)	Total aligments	Successfully assigned alignments	Successfully assigned alignments (%)
GPFT1b1	4655694	4222225	90,7	4655694	3788489	81,4
GSFT1b1	5669861	5110816	90,1	5669861	4625279	81,6
GPFT2b1	4206178	3691752	87,8	4206178	3247258	77,2
GSFT2b1	3878788	3484751	89,8	3878788	3032011	78,2
GPFT3b1	8523302	7722694	90,6	8523302	6927262	81,3
GSFT3b1	6635212	5965964	89,9	6635212	5170153	77,9
GPVVT1b2	4034127	3607857	89,4	4034127	3194052	79,2
GSVVT1b2	6648743	5850834	88,0	6648743	5160811	77,6
GPVVT2b2	7537369	6864893	91,1	7537369	6189257	82,1
GSVVT2b2	4962344	4433373	89,3	4962344	3839870	77,4
GPVVT3b2	6692718	5890476	88,0	6692718	5225320	78,1
GSVVT3b2	5678670	5138631	90,5	5678670	4500359	79,3
GPLT1b3	5144106	4686361	91,1	5144106	4172776	81,1
GSLT1b3	6910267	5998470	86,8	6910267	5186832	75,1
GPLT2b3	5846997	5358342	91,6	5846997	4841538	82,8
GSLT2b3	4863889	4356512	89,6	4863889	3780896	77,7
GPLT3b3	7592828	6946848	91,5	7592828	6280821	82,7
GSLT3b3	5752950	5233878	91,0	5752950	4572136	79,5
CPFT1b4	5554313	4915379	88,5	5554313	4339990	78,1
CSFT1b4	5138944	4572688	89,0	5138944	4069119	79,2
CPFT2b4	5022469	4513670	89,9	5022469	3994855	79,5
CSFT2b4	2968529	2578672	86,9	2968529	2180334	73,4
CPFT3b4	9110892	7856740	86,2	9110892	6801528	74,7
CSFT3b4	5833050	5291351	90,7	5833050	4681506	80,3
CPVVT1b5	2946399	2620937	89,0	2946399	2336893	79,3
CSVVT1b5	2904310	2322544	80,0	2904310	2071028	71,3
CPVVT2b5	4993142	4432868	88,8	4993142	3906147	78,2
CSVVT2b5	4115869	2366359	57,5	4115869	1131102	27,5
CPVVT3b5	6478501	5502298	84,9	6478501	4707638	72,7
CSVVT3b5	7038770	6325013	89,9	7038770	5497798	78,1
CPLT1b6	5673559	5039905	88,8	5673559	4473102	78,8
CSLT1b6	851082	595658	70,0	851082	527448	62,0
CPLT2b6	5576766	4828992	86,6	5576766	4154243	74,5
CSLT2b6	923414	783418	84,8	923414	683045	74,0
CPLT3b6	7209101	6240826	86,6	7209101	5395893	74,8
CSLT3b6	6828436	6206280	90,9	6828436	5414323	79,3
GPLT1b7	3381020	3110798	92,0	3381020	2821894	83,5
GSLT1b7	5517437	4871728	88,3	5517437	4321171	78,3
GPLT2b7	5794385	5324742	91,9	5794385	4800466	82,8
GSLT2b7	4636679	4171682	90,0	4636679	3639780	78,5
GPLT3b7	9450335	8569754	90,7	9450335	7688911	81,4
GSLT3b7	5954859	5379829	90,3	5954859	4683102	78,6
GPFT1b8	4955065	4475621	90,3	4955065	3994429	80,6
GSFT1b8	5731569	5094433	88,9	5731569	4526095	79,0
GPFT2b8	4229050	3766084	89,1	4229050	3328267	78,7
GSFT2b8	3580860	3214962	89,8	3580860	2860780	79,9
GPFT3b8	8218333	7452183	90,7	8218333	6743403	82,1
GSFT3b8	7002075	6322591	90,3	7002075	5541761	79,1
GPVVT1b9	5028747	4566382	90,8	5028747	4126256	82,1
GSVVT1b9	5931961	5133522	86,5	5931961	4431074	74,7
GPVVT2b9	4955400	4481219	90,4	4955400	3997652	80,7
GSVVT2b9	4318730	3847153	89,1	4318730	3381480	78,3
GPVVT3b9	6855645	6192860	90,3	6855645	5591875	81,6
GSVVT3b9	6564710	5860301	89,3	6564710	5060512	77,1
CPLT1b10	12785350	11522726	90,1	12785350	10131950	79,2
CSLT1b10	12283487	11006017	89,6	12283487	9754816	79,4
CPLT2b10	9530308	8570068	89,9	9530308	7604728	79,8
CSLT2b10	9872791	9022621	91,4	9872791	7967444	80,7
CPLT3b10	11508033	8486638	73,7	11508033	5499175	47,8
CSLT3b10	13677194	12565121	91,9	13677194	11004919	80,5
CPFT1b11	11662012	10387420	89,1	11662012	9111585	78,1
CSFT1b11	12088122	10849048	89,7	12088122	9591268	79,3
CPFT2b11	10145004	9195885	90,6	10145004	8119337	80,0
CSFT2b11	8988293	7783542	86,6	8988293	6258825	69,6
CPFT3b11	10221624	8764980	85,7	10221624	7450649	72,9

CSFT3b11	9213462	8393113	91,1	9213462	7290758	79,1
CPVVT1b12	9637651	8659972	89,9	9637651	7666486	79,5
CSVVT1b12	7335591	6540723	89,2	7335591	5961005	81,3
CPVVT2b12	10268500	9161684	89,2	10268500	8007399	78,0
CSVVT2b12	9970776	6353573	63,7	9970776	3028625	30,4
CPVVT3b12	11467785	9842593	85,8	11467785	8514193	74,2
CSVVT3b12	9981936	9088659	91,1	9981936	7887099	79,0
GPVVT1b13	11523199	10698301	92,8	11523199	9680105	84,0
GSVVT1b13	12517302	11317674	90,4	12517302	9961534	79,6
GPVVT2b13	10192578	8413887	82,5	10192578	6826789	67,0
GSVVT2b13	13011023	11481926	88,2	13011023	9695806	74,5
GPVVT3b13	10675645	9144383	85,7	10675645	7766980	72,8
GSVVT3b13	11538925	10466685	90,7	11538925	9031289	78,3
GPLT1b14	13523065	12663211	93,6	13523065	11539207	85,3
GSLT1b14	9636245	8456535	87,8	9636245	7181494	74,5
GPLT2b14	11674765	9708204	83,2	11674765	7860735	67,3
GSLT2b14	12357045	11170623	90,4	12357045	9644151	78,0
GPLT3b14	13779261	12449920	90,4	13779261	11115842	80,7
GSLT3b14	11095330	10238318	92,3	11095330	8995453	81,1
GPFT1b15	12461885	11634677	93,4	12461885	10511587	84,3
GSFT1b15	12013217	10924431	90,9	12013217	9630498	80,2
GPFT2b15	10959141	10078445	92,0	10959141	9021560	82,3
GSFT2b15	11558672	10603292	91,7	11558672	9336125	80,8
GPFT3b15	11389355	10532111	92,5	11389355	9584380	84,2
GSFT3b15	8110960	7481212	92,2	8110960	6548165	80,7
CPVVT1b16	31970629	28010803	87,6	31970629	24544860	76,8
CSVVT1b16	8419	7315	86,9	8419	6450	76,6
CPVVT2b16	9958412	8999522	90,4	9958412	8114202	81,5
CSVVT2b16	11143768	9828338	88,2	11143768	8513811	76,4
CPVVT3b16	11337115	9811681	86,5	11337115	8515943	75,1
CSVVT3b16	10640739	9805797	92,2	10640739	8634773	81,1
CPLT1b17	11068414	9737637	88,0	11068414	8406173	75,9
CSLT1b17	5999	5316	88,6	5999	4669	77,8
CPLT2b17	9787200	8731421	89,2	9787200	7692914	78,6
CSLT2b17	193084	166784	86,4	193084	145903	75,6
CPLT3b17	10202352	7911006	77,5	10202352	5719363	56,1
CSLT3b17	9912731	9259988	93,4	9912731	8268393	83,4
CPFT1b18	10596171	9411865	88,8	10596171	8165993	77,1
CSFT1b18	4658	4064	87,2	4658	3571	76,7
CPFT2b18	9579533	8611909	89,9	9579533	7585612	79,2
CSFT2b18	182650	143282	78,4	182650	122683	67,2
CPFT3b18	10613212	9283743	87,5	10613212	8004731	75,4
CSFT3b18	10619300	9767130	92,0	10619300	8587103	80,9

Table S7 Matrix of raw, mean raw and VST normalized counts. *Table S7 has been uploaded as a separate Excel spread sheet.*

Table S8 Differentially expressed transcripts based on ANOVA analysis. *Table S8 has been uploaded as a separate Excel spread sheet.*

Table S9 Screening guidelines

Phase	Feature	Rule	Number of genes
I	Unexpressed genes	A gene was considered *unexpressed* if the value of its expression is below the threshold 5 in at least 2 of 3 replicates, for all the 36 experimental conditions. Examples of removed genes in **Fig. S5**.	10837
II	Genes with low variability among the experimental conditions	For gene i, the variability of the expressions among the 36 experimental conditions was measured by means of the Pearson ratio<br>$$\eta_i^2 = \frac{\sum_{j=1}^{36}\left(m_{ij} - m_i\right)^2}{\sum_{r=1}^{3}\sum_{j=1}^{36}(x_{ijr} - m_i)^2}$$<br>Gene i was considered to have a low variability between the experimental conditions if $\eta_i^2 < 0.5$. Examples of removed genes in **Fig. S6**.	11604
III	Genes with outliers	Some genes showed expressions characterized by a high peak in one of the 36 experimental conditions, which in general has to be considered an outlier, i.e. it can be due to errors in recording data or specific conditions. It is reasonable to remove these genes from the analysis. They were identified as follows:<br>*i.* for gene i, let $m_{i(1)}, \cdots, m_{i(36)}$ be the averages $m_{i1}, \cdots, m_{i36}$, sorted in increasing order;<br>*ii.* let $\Delta m_i = m_{i(36)} - m_{i(35)}$ and let SD$_i$ be the standard error of $m_{i(1)}, \cdots, m_{i(35)}$;<br>*iii.* identify a peak in $m_{i(36)}$ if $\Delta m_i > k \cdot SD_i$, where k is a fixed threshold.<br>In our analysis we found that a reasonable value for k was 6. Examples of genes identified with this procedure are given in **Fig. S7**.	62

Table S10 Set of the 8158 modulated genes analyzed using the statistical pipeline. *Table S10 has been uploaded as a separate Excel spread sheet.*

Table S11 Characterization of the 102 clusters identified by the VIM analysis. *Table S11 has been uploaded as a separate Excel spread sheet.*

Table S12 Detailed description of all the 102 clusters of gene expression defined in the present study. *Table S12 has been uploaded as a separate PDF file.*

Table S13 List of 740 genes belonging to the 11 soil specific clusters. *Table S13 has been uploaded as a separate Excel spread sheet.*

Table S14 Enrichment analysis of the promoter regions of the 740 soil specific genes.

Enriched motif in promoter	TF	TF family	P val.	FDR	Score
GGTAGGTG	VIT_07s0130g00040	MYB13/14/15, -3	5.4E-14	9.1E-12	1.1E+01
CCACCTACCCCC	VIT_00s1352g00010	MYB148	2.8E-12	2.3E-10	1.8E+01
GCCGCC	VIT_15s0021g01610	-	5.3E-11	2.9E-09	7.0E+00
GCCGCC	VIT_16s0013g00900	-	2.2E-09	9.3E-08	5.0E+00
CACGTG	VIT_12s0028g01110	bHLH048	4.5E-09	1.5E-07	7.0E+00
CCACGTG	VIT_18s0001g10270	bHLH085	1.5E-08	4.1E-07	9.0E+00
GCCGCC	VIT_07s0005g03190	-	2.6E-08	6.1E-07	7.0E+00
CACGTG	VIT_01s0244g00010	-	5.3E-08	1.1E-06	6.0E+00
ACGTGG	VIT_02s0012g02250	bZIP04	7.2E-08	1.3E-06	5.0E+00
GCACGTG	VIT_05s0049g00460	bHLH024	1.0E-07	1.7E-06	9.0E+00
CACGCAA	VIT_17s0000g03660	NAC06	1.4E-07	1.9E-06	5.0E+00
CACGTG	VIT_07s0104g00090	MYC1	1.4E-07	1.9E-06	6.0E+00
CACGTG	VIT_07s0141g00220	bHLH032	1.4E-07	1.9E-06	9.0E+00
CCACGTG	VIT_01s0010g00930	bZIP02	1.7E-07	2.1E-06	1.0E+01
CACGTGC	VIT_17s0000g06930	bHLH079	2.4E-07	2.6E-06	1.0E+01
CACGTGC	VIT_07s0151g00450	bHLH034	2.6E-07	2.7E-06	1.0E+01
CACGTG	VIT_07s0005g05100	-	2.9E-07	2.8E-06	9.0E+00
CACGTG	VIT_00s0532g00050	bHLH090	3.0E-07	2.8E-06	7.0E+00
ACGCAA	VIT_17s0000g06400	NAC05	3.6E-07	3.2E-06	3.0E+00
ACGTGGC	VIT_18s0001g12120	bZIP46	9.3E-07	7.8E-06	7.0E+00
ACACGTG	VIT_12s0055g00420	bZIP30	1.6E-06	1.3E-05	1.0E+01
CACGTG	VIT_01s0010g00540	bHLH001	2.2E-06	1.7E-05	8.0E+00
GCCGCC	VIT_14s0081g00730	-	4.2E-06	3.0E-05	9.0E+00
CACCGACC	VIT_00s0662g00040	-	4.6E-06	3.2E-05	6.0E+00
CACGTG	VIT_17s0000g04790	bHLH077	5.1E-06	3.4E-05	5.0E+00
GCCG	VIT_09s0002g08830	-	9.0E-06	5.8E-05	4.0E+00
GCCG	VIT_01s0150g00120	-	1.0E-05	6.2E-05	5.0E+00
GACACGTG	VIT_08s0007g03420	bZIP25	1.1E-05	6.6E-05	8.0E+00
GCACGTG	VIT_04s0023g01930	bHLH017	1.3E-05	7.6E-05	8.0E+00
GACGTG	VIT_08s0040g00870	bZIP24	1.9E-05	1.1E-04	4.0E+00

Table S15 MYB13/14/15 DAPseq peaks for soils-specific genes. *Table S15 has been uploaded as a separate Excel spread sheet.*

Methods S1 Detailed description of the experimental plan

Setting up the experimental plan required the transfer of approximately 40 m^3 of each soil from the site of origin to the experimental farm. The choice of land collection areas was based on the availability of landowners, on the original use of the area, and on the homogeneity of the substrate. In this regard, to avoid mixing the deeper layers of the soil during the transfer phases, the sampling was limited to the most superficial layer, i.e., the first 30 cm of depth and, to make samples comparable, all soils were mixed before filling caissons, given the impossibility of maintaining their original structure and stratification, due to the size of caissons and the presence of skeleton in F and VV soils. Concrete caissons containing the different soils had no base to avoid water stagnation. Kober 5BB was chosen as rootstock since its performances are quite stable and uniform under many different environmental conditions avoiding favoring the plant growth on one substrate rather than another. Each cultivar/soil combination was set in three biological replicates constituted of three independent caissons, each one containing 4 plants, for a total number of 18 randomly distributed caissons (2 cultivars x 3 soils x 3 biological replicates) and 72 individual plants (**Fig. 1**). Plants were grown using a spurred cordon training system with a plant-to-plant distance equal to 100 cm, and North-South orientation of rows. To avoid introducing additional variables and to not overshadow any possible difference in plant phenotype, no fertilization and irrigation were carried out considering fertility and water retention as intrinsic features of different soils. The weeding was carried out mechanically and manually, while treatments with fungicides or pesticides were carried out following the local protocols against grapevine leafhopper (*Scaphoideus titanus*, *Cicadellidae*), powdery mildew (*Erysiphe necator*) and downy mildew (*Plasmopara viticola*).

Growing conditions

The air temperature for 2017 and 2018 growing season were monitored downloading data recorded from an ARPAV meteorological station (https://www.arpa.veneto.it/bollettini/ storico/ Mappa_2020_TEMP.htm?t=RG; station n°111) located in close proximity to the experimental

field (Lat. 45.348, Lon. 11.953), 2 m from the ground level. Average daily temperatures were used to define average monthly and seasonal temperature trends. Monthly precipitations were obtained from the same source.

Methods S2 Soil classification according to ARPAV

Fumane soil ("F")

According to the Veneto Land Map (ARPAV, www.arpa.veneto.it), the soil taken from the Valpolicella area, in the locality of Fumane, can be identified by the code 'AR2.2' (**Fig. S18**). It refers to valley and conoid fills, characterized by mixed, fine, and gravelly, extremely calcareous deposits, derived from the alteration of rocks of sedimentary origin. They are gently sloping surfaces (1- 3% slope), with traces of intertwined channels not very evident. Land use is mainly made up of arable land (corn), vineyards and meadows. The parental material consists of clay and lime mixed with gravel, extremely calcareous. However, they are deep soils, with a moderately fine texture, a common skeleton, an alkaline reaction, and good drainage.

Legnaro soil ("L")

The soil taken from the locality of Legnaro, precisely within the boundaries of the experimental farm L. Toniolo of the University of Padua, can be identified by the code BR4.6 (**Fig. S18**). The description refers to the modal plain formed by the Brenta river in recent times. They are flat surfaces that develop with an NW-SE course, starting from the meandering plain, up to the lagoon edge, and are connected to the bumps. Land use is mainly made up of arable land (maize). The parental material consists of a good fraction of silt. The soils are deep, of medium texture, alkaline reaction, very calcareous, strongly calcareous in depth, mediocre drainage, deep brim.

Vittorio Veneto soil ("VV")

The land taken from the Valdobbiadene-Conegliano area of Vittorio Veneto can be identified by the code AA2.2 (**Fig. S18**). It refers to ancient surfaces of the Piave river and the proglacial plain of the amphitheater of Vittorio Veneto (coneides of Nervesa and Vittorio Veneto), with traces of intertwined canals, sub-flat (0.5-1% slope). The starting material is extremely calcareous, sandy,

and gravelly. The soils are deep, moderately fine texture, coarse in depth, with an abundant skeleton that allows moderately rapid drainage, the reaction is alkaline. Generally, the soil is use d for arable crops (corn), vineyards and meadows.

Methods S3 RNAseq library construction

Libraries were constructed starting from 700 ng of total RNA using the Kapa mRNA HyperPrep Kit (Roche) combined with the Kapa Dual Index Kit (Roche) according to the manufacturer's instructions. The pooled library was quantified by qPCR and the fragment insertion size of 300 bp was evaluated on the Agilent 2200 Tapestation. Paired-end reads of 100 nucleotides (nt) were obtained using an Illumina HiSeq 2500 sequencer, and sequencing data were generated using the base-calling software Illumina Casava v1.8.2. After RNA Sequencing the raw data (FASTQ) underwent quality control analysis using FastQC Version 0.11.5 and the Illumina reads were pre-processed to remove low-quality reads and sequencing adapters with Trimmomatic Version 0.39 (Bolger et al., 2014). The resulting pre-processed reads were aligned to the reference genome of Vitis vinifera 12X (Ensembl Plants Genome release 43) with the Subread aligner Version 1.6.4 (Liao et al., 2014). Raw read counts were extracted from the Subread alignments using the featureCount Version 1.6.4 read summarization program (Liao et al., 2014).

Methods S4 Variable Importance Measure (VIM) analysis

Notation

Covariates:

- Cultivar [Corvina, Glera]
- Stage [Softening (S), Complete-Véraison/Mid-Ripening (PV/MR), Harvest (H)]
- Soil [Fumane, Vittorio Veneto, Legnaro]
- Tissue [Skin, Pulp]

Each cultivar/soil combination was set in three biological replicates constituted of three independent caissons, each one containing 4 plants, for a total number of 18 randomly distributed caissons (2 cultivars x 3 soils x 3 biological replicates) and 72 individual plants.

We will use the term *experimental condition* to denote the 36 possible combinations of the 4 variables, e.g. {Cultivar:Corvina x Stage:S x Tissue:Pulp x Soil:Vittorio Veneto}.

We denote with x_{ijr} the expression of gene *i* in the *j*th experimental condition for the *r*th replicate, with m_{ij} its average expression (over the three replicates) in the *j*th experimental condition, with m_i its overall average expression:

$$m_{ij} = \frac{1}{3}\sum_{r=1}^{3} x_{ijr} \qquad m_i = \frac{1}{108}\sum_{r=1}^{3}\sum_{j=1}^{36} x_{ijr}$$

In the following the patterns of the gene expressions will be displayed using a 6-panels graphical representation such as, for examples, those visualized in **Fig. S5**. The 6 panels are obtained by crossing variables Soil and Tissue, so that each panel shows the expression patterns for a given Soil and a given Tissue. In each panel a two-dimensional graph is plotted, with the *x* and the *y* axis representing the levels of Stage and the expression values, respectively. A line shows the pattern of the average expressions m_{ij}. When a single gene is displayed, three points are also present for each Stage level, representing the three replicates. The variable Cultivar is accounted for by means of the color of points and lines (red=Corvina, cyan=Glera).

General description of the pipeline

The gene expressions have been analyzed with data mining procedures, in order to summarize the most important relationships in data, with specific attention to discover the extent to which the variables Cultivar, Stage, Soil and Tissue - separately or in interaction - affect gene expressions. The data mining process was composed of three steps:

1. **Screening:** we identified, out of the whole set of 30661 genes, a subset of 22503 genes with uninteresting patterns with respect to the aim of the present study (unexpressed genes, genes with almost constant expressions, genes with outlier expressions). After deleting this "noisy" subset, the resulting dataset was composed of 8158 genes deserving statistical analysis.
2. **Profiles definition:** in the second step we performed a cluster analysis using the *k*-mean algorithm, in order to reduce the 8158 genes into a number of clusters containing genes with

similar patterns among the experimental conditions. We obtained 102 clusters, globally accounting for about 80% of the total variance of the genes expressions. For each cluster we defined an average profile, to be used as a representative of the cluster, and an index of its representativeness, based on the variability of the expressions around the average profile.

3. **Profiles characterization:** in the third step we exploited an advanced machine learning algorithm, the Gradient Boosting Machine (GBM) (Friedman, 2001), with the aim of evaluating to what extent each experimental condition affects the gene expression patterns. To this aim Variable Importance Measures (*VIM*s) have been computed, a tool able to describe the impact of given variables on a selected outcome, considering the presence of possible, even complex, interactions among the variables themselves. In other words, for each gene, we defined how the variability of its expressions can be accounted for by stage, cultivar, year, site, or an interaction of them, with a multivariate approach. The median *VIM*s within the clusters defined in step 2, allow to characterize the clusters under this point of view, thus determining the relationship between the clusters and the experimental conditions. In the end, the *VIM*s have been crossed with the results coming from a Principal Component Analysis of the cluster profiles, in order to better interpret results.

Step 1: Screening

We identified the genes to remove according to the criteria summarized in Table 1. **Fig. S5**, **S6**, and **S7** show some representative expression profiles of amongst these removed genes. In the end, 22503 genes were identified for removal and the remaining 8158 genes were selected for the analysis and were passed to the following two Steps.

Step 2: Profiles definition

The data matrix for this step was given by the (8158 x 36) table containing the average expressions m_{ij} of the genes, standardized by rows, that will be denoted $\tilde{m}_{ij}$ in the following. A cluster analysis was performed with the *k*-means algorithm, in order to define groups of genes with similar patterns along the experimental conditions. After examining different alternatives, the number of clusters was determined so as to explain about 80% of the total variability of the genes expressions. This choice was judged an acceptable trade-off between the need of a

number of clusters as low as possible (easier interpretation) and a good separation between the clusters (high internal cohesion); a set of k = 102 clusters was identified (**Fig. S8**).

For each cluster c, an average profile $P_c = (P_{1c}, P_{2c}, \ldots, P_{36c})$ was defined, where P_{jc} denotes the average standardized expression of the genes belonging to cluster c in the experimental condition j, to be used as a representative of the cluster:

$$P_{jc} = \frac{1}{n_c} \sum_{i \in c} \tilde{m}_{ij}$$

where n_c is the number of genes belonging to cluster c. The internal cohesion of each cluster was measured by the following homogeneity index

$$R_c = 1 - \frac{\sum_{j=1}^{36} \sum_{i \in c} \left(\tilde{m}_{ij} - P_{jc} \right)^2}{\sum_{j=1}^{36} \sum_{i \in c} \left(\tilde{m}_{ij} - \bar{P}_c \right)^2}$$

where $\bar{P}_c = \frac{1}{36} \sum_{j=1}^{36} P_{jc}$. **Fig. S9** shows two examples of clusters with high and low Rc, respectively.

Step 3: Profiles characterization

Step 3.1: Modelling the relationship between gene expressions and covariates

In this step the genes have been analyzed one-to-one in order to measure the impact of the covariates on gene expressions. Then, the analyzed data were given by the 8158 matrices of dimension (36 x 5), where each row describes one experimental condition according to the variables Cultivar, Stage, Soil, Tissue, Gene Expression. The idea was to fit a predictive model to the outcome variable Gene Expression, with the covariates as predictors, in order to inspect, for each gene, the extent to which covariates affect the gene expression. From a statistical point of view, two main issues have to be considered in setting the predictive model: the possible nonlinearity of relationships between the outcome and the predictors and the possible presence of interactions among the predictors in affecting the outcome. At the same time, we are not only interested to prediction itself but also to the extrapolation of the role played by covariates, for interpretative purposes. This is the main reason why, in spite of the need of complex models, it is often inopportune to take advantage of powerful nonlinear regression techniques such as, for example, neural networks, which, in change of a significant predictive accuracy, are impenetrable

black boxes. In the last decades the predictive and interpretative power of statistical tools has been greatly improved by the introduction of data mining algorithms able to deal with complex relationships among variables from a multivariate perspective. These new algorithms generate, together with predictions, variable importance measures (*VIM*s) identifying the most important predictors of the outcome. Within this innovative approach, called algorithmic modelling (Breiman, 2001a), a great deal of attention has been devoted to the ensemble learning philosophy (Friedman, 2003, 2006; Friedman and Popescu, 2003, 2005). Learning ensembles are sequences of ensemble members, i.e. statistical models predicting the outcome as a function of the set of predictors. Final predictions are obtained by a linear combination of the prediction of each ensemble member. Learning ensembles can be built using different prediction methods, i.e. different base learners as ensemble members. The most interesting proposals use decision trees (more specifically CART, Classification And Regression Trees - Breiman et al., 1984) as base learners and are called tree-based learning ensembles. Popular examples are the Random Forest technique (RF - Breiman, 2001b) or the tree-based Gradient Boosting Machine (GBM - Friedman, 2001). Both these algorithmic techniques identify the most important predictors within the set of covariates, by means of the computation of *VIM*s. In this case study we have used GBM to fit each gene expression as a function of the environmental variables. Then, for each gene we have been able to compute 4 *VIM*s, measuring the extent to which each environmental variable affects the gene expression, on its own or jointly/in interaction with the others.

Let VIM_{ih} (*h*=1,2,3,4) be variable importance measure of the *h*th covariate for gene *i*. We computed the average *VIM*s for the 102 clusters obtained in Step 2

$$VIM_h^c = \frac{1}{n_c}\sum_{i \in c} VIM_{ih}$$

and used them to characterize the cluster profiles according to the relationship between gene expression and covariates (see the example of cluster no. 58 in **Fig. S10**).

Step 3.2: Principal Component Analysis of the cluster average profiles

Subsequently, we performed a dimensionality reduction via Principal Component Analysis (PCA) of the (36 x 102) matrix containing, by columns, the average profiles $P_c = (P_{1c}, P_{2c}, \ldots, P_{36c})$ of the 102 clusters.

Fig. S11-S13 displays some selected two-dimensional objects scores plots, which highlight that the Principal Components, computed as linear combinations of the cluster profiles, are able to discriminate the variables characterizing the 36 experimental conditions with a remarkable accuracy.

Step 3.3: Combining PCA, VIMs and cluster homogeneity index

To gain an immediate and comprehensive insight on the cluster profiles, we have developed a set of interactive graphical tools for the investigation of the characteristics of the 102 clusters according to several variables. **Fig. S15** to **S17** show some noteworthy examples. **Fig. S15** shows that the loadings of the clusters in the first Principal Component (Dim1) are associated with the importance of the variable Stage (RnkVIMStage = rank of clusters according to VIM_{Stage}^{c}; low values denote high importance of Stage). Specifically, the higher the importance of Stage, the higher the loadings (in absolute value). The expressions of genes belonging to clusters with positive loadings exhibit a positive trend with respect to Stage (i.e., a pattern with increasing values as stage moves forward), whereas the opposite happens for genes with negative loading. All the clusters denote a good homogeneity (represented by the colour of points, with red denoting a high value of the index R_c). This graph is a valid tool for the identification of clusters composed by genes with expressions highly associated to Stage, and with very similar patterns within the clusters.

Like the preceding figure, **Fig. S16** shows that cluster loadings in the second Principal Component (Dim2) are associated with the importance of the variable Tissue (RnkVIMTissue = rank of clusters according to VIM_{Tissue}^{c}; low values denote high importance of Tissue for that cluster). Also in this case, the higher the importance of Tissue, the higher the absolute values of the loadings. The expressions of genes belonging to clusters with positive loadings exhibit a median higher expression in pulp, whereas the opposite happens for genes with negative loadings. Even in this case all the clusters denote a good homogeneity. This graph helps to identify clusters composed of genes associated to the Tissue, with quite similar patterns within the clusters. **Fig. S17** can be interpreted analogously to **Fig. S15** and **S16**, with reference to Cultivar.

References

Breiman L., (1996a), The heuristic of instability in model selection, Annals of Statistics, 24, 6, 2350-2383.

Breiman L., (1996b), Bagging Predictions, Machine Learning, 24, 2, 123-140.

Breiman L., (2001a), Statistical Modeling: The Two Cultures, Statistical Science, 16, 3, 199-231.

Breiman L., (2001b), Random Forests, Machine Learning, 45, 1, 5-32.

Breiman L., Friedman J.H, Olshen R.A. and Stone C.J. (1984), Classification and Regression Trees, Chapman & Hall, New York.

Friedman J.H. (2001), Greedy function approximation: a gradient boosting machine, Annals of Statistics, 29: 1189-1232.

Friedman, J.H. (2006) Recent advances in predictive (machine) learning, Journal of classification 23(2): 175-197.

Friedman, J. H. (2006), Separating signal from background using ensembles of rules, In Lyons L. and Ünel M.K. (eds.), Statistical Problems in Particle Physics, Astrophysics and Cosmology, Proceedings of PHYSTAT05, Imperial College Press, London

Friedman, J. H., and Popescu, B. E. (2003), Importance Sampled Learning Ensembles, Stanford University, Department of Statistics, Technical Report.

Friedman, J. H., and Popescu, B. E. (2008), Predictive learning via rule ensembles, The Annals of Applied Statistics, 2 (3): 916-954
