# Supplementary material for: Dissecting the effect of soil on plant phenology and berry transcriptional plasticity in two Italian grapevine varieties (Vitis vinifera L.)
Source: Hortic Res. 2023 Mar 28;10(5):uhad056. doi: 10.1093/hr/uhad056 (PMC10199706; doi:10.1093/hr/uhad056)
Supplement: Web_Material_uhad056 [file web_material_uhad056.zip › Table S12 - Detailed description of all the 102 clusters of gene expression defined in the present study..pdf]

Supplementary material

Preliminary report

A. Vannozzi, S. Zenoni, P. Zuccolotto, M. Sandri

July 20, 2020

Cluster no. 1

```
## Number of genes in the cluster: 33
## Homogeneity Index:    0.83
## Variable importance for Stage:     Rank =   1  - Median =   1120
## Variable importance for Cultivar:  Rank =  92  - Median =      8
## Variable importance for Tissue:    Rank =  96  - Median =  11.06
## Variable importance for Soil:      Rank =  31  - Median =   6.62
##
## Gene ID               Gene Annotation
## VIT_02s0025g04170  -  Heat shock transcription factor B2B
## VIT_13s0019g02280  -  Regulator of chromosome condensation (RCC1)
## VIT_05s0102g00620  -  No hit
## VIT_00s0194g00030  -  Heat shock protein 81-2 (HSP81-2)
## VIT_10s0003g00260  -  DnaJ homolog, subfamily B, member 4
## VIT_09s0002g00640  -  Small heat stress protein class CIII
## VIT_16s0022g00510  -  Heat shock 22 kDa protein
## VIT_01s0010g00600  -  Phosphatidylinositol 4-kinase type-II
## VIT_08s0007g06710  -  Aha1 domain-containing protein
## VIT_13s0019g03170  -  Heat shock protein 16.9 kDa class I
## VIT_01s0026g02540  -  Zinc finger (C3HC4-type ring finger)
## VIT_17s0000g07190  -  Heat shock protein 101
## VIT_09s0002g00630  -  No hit
## VIT_06s0004g04470  -  Heat shock protein 70
## VIT_04s0043g00310  -  Ribulose-phosphate 3-epimerase
## VIT_00s1569g00010  -  Heat shock protein 81-2 (HSP81-2)
## VIT_08s0007g00130  -  Heat shock protein 70
## VIT_13s0047g00110  -  Ripening regulated protein DDTFR8
## VIT_00s2341g00010  -  Heat shock protein 81-4 (HSP81-4)
## VIT_04s0008g01110  -  Heat shock transcription factor A6B
## VIT_09s0018g01680  -  Unknown protein
## VIT_13s0019g02780  -  Heat shock protein 17.6 kDa class I
## VIT_01s0011g04990  -  Chaperonin
## VIT_05s0102g00600  -  No hit
## VIT_18s0041g01230  -  Heat shock protein 70
## VIT_06s0004g05770  -  Heat shock protein 17.6 kDa class I
## VIT_01s0010g02290  -  Heat shock protein 26a, chloroplast
## VIT_11s0037g00510  -  Heat shock protein 70
## VIT_07s0005g01970  -  Galactinol synthase
## VIT_19s0085g01050  -  Heat shock protein 17.6 kDa class I
## VIT_07s0005g01980  -  Glycosyl transferase family 8 protein
## VIT_00s0179g00150  -  Heat shock transcription factor A6B
## VIT_08s0007g04000  -  Unknown protein
```

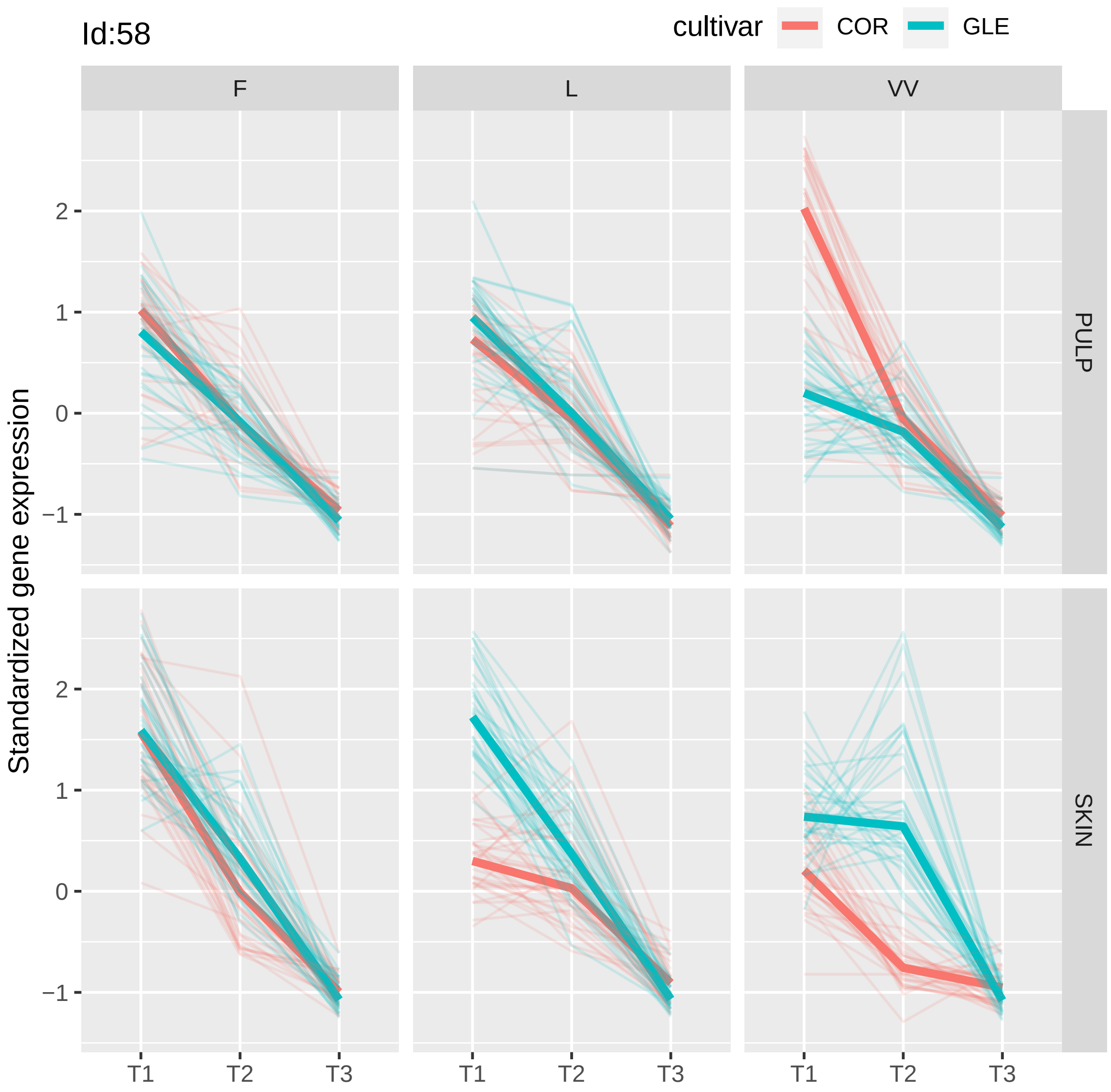
Id:58
cultivar
COR
GLE
F
L
VV
PULP
SKIN
Standardized gene expression
2
1
0
−1
T1
T2
T3

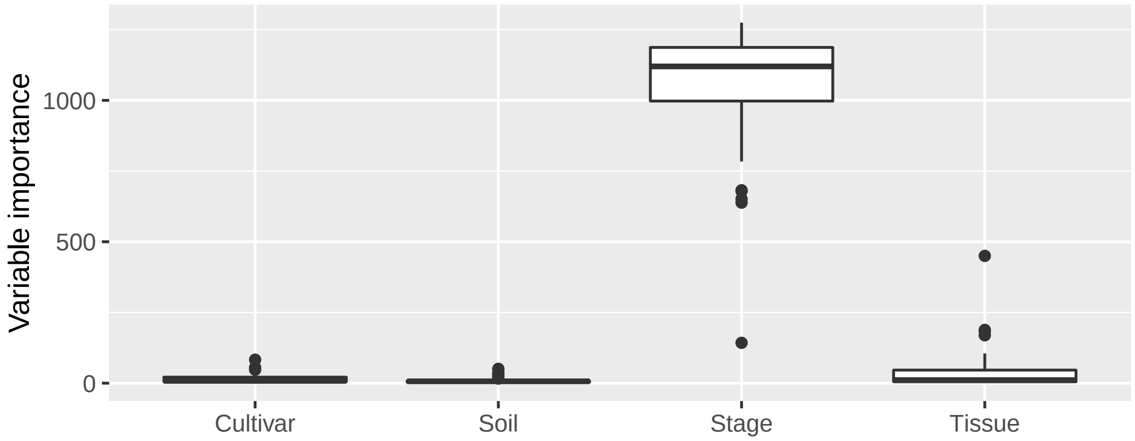
Variable importance
1000
500
0
Cultivar
Soil
Stage
Tissue

Cluster no. 2

```
## Number of genes in the cluster: 185
## Homogeneity Index:    0.92
## Variable importance for Stage:     Rank =   2  - Median =   1110
## Variable importance for Cultivar:  Rank =  97  - Median =   5.94
## Variable importance for Tissue:    Rank = 100  - Median =    8.1
## Variable importance for Soil:      Rank =  73  - Median =   4.58
##
## Gene ID                Gene Annotation
## VIT_10s0003g04560  -  Auxin-Induced in Root cultures 9 AIR9
## VIT_14s0108g00850  -  Ribosomal protein L34 (RPL34A) 60S
## VIT_02s0025g03590  -  Phospholipid hydroperoxide glutathione peroxidase
## VIT_17s0000g02120  -  Unknown protein
## VIT_14s0066g01590  -  NHL repeat-containing protein
## VIT_01s0011g03750  -  Phosphomannose isomerase
## VIT_13s0067g01450  -  Lateral organ boundaries domain 21
## VIT_09s0070g00480  -  LRX2 (leucine-rich repeat/extensin 2)
## VIT_12s0134g00200  -  Methyltransferase
## VIT_09s0002g04240  -  Membrane protein
## VIT_11s0016g04960  -  Aspartate-glutamate racemase
## VIT_02s0025g03260  -  Aquaporin NIP5;1
## VIT_08s0007g02750  -  No hit
## VIT_13s0067g01000  -  PHD finger protein alfin
## VIT_17s0000g09400  -  Unknown protein
## VIT_18s0001g13230  -  Beta-galactosidase BG1 [Vitis vinifera]
## VIT_18s0001g05040  -  Pollen Ole e 1 allergen and extensin
## VIT_03s0038g03220  -  Chitin elicitor-binding CEBIP LysM domain-containing
## VIT_16s0050g00080  -  Remorin
## VIT_10s0116g00250  -  Unknown
## VIT_07s0031g02480  -  Beta-galactosidase
## VIT_03s0063g01340  -  Gibberellin 20 oxidase 2
## VIT_08s0040g02210  -  Lectin protein kinase
## VIT_05s0077g01080  -  PAPA-1-like family protein
## VIT_04s0079g00420  -  Expansin  (VvEXPA4)
## VIT_14s0066g01580  -  K+ uptake permease 6
## VIT_07s0031g00610  -  Remorin
## VIT_05s0020g01740  -  RanGAP1 interacting protein
## VIT_05s0020g01970  -  Vesicle-associated membrane protein 27 VAP27-2
## VIT_04s0023g03810  -  Jasmonate O-methyltransferase
## VIT_07s0005g01480  -  PMR5 (powdery mildew resistant 5)
## VIT_06s0004g07310  -  Indole-3-acetate beta-glucosyltransferase
## VIT_05s0062g01360  -  Unknown protein
## VIT_01s0011g04310  -  Zinc finger (C3HC4-type ring finger)
## VIT_08s0058g01420  -  Unknown protein
## VIT_07s0129g00190  -  Binding
## VIT_14s0066g01300  -  Unknown protein
## VIT_12s0035g00690  -  TCP family transcription factor TCP23
## VIT_05s0020g01440  -  Unknown protein
## VIT_18s0001g08250  -  Tubulin alpha-3 chain
## VIT_12s0055g01150  -  Brassinosteroid insensitive 1-associated receptor kinase 1
## VIT_01s0010g01760  -  Remorin
## VIT_06s0061g00350  -  ABC Transporter (VvWBC24 - VvABCG24)
## VIT_18s0001g08610  -  ERF/AP2 Gene Family (VvAP2-19)
## VIT_16s0050g01990  -  Unknown
## VIT_03s0038g02150  -  Unknown
```

```
## VIT_10s0042g00170  -  TCP family transcription factor 7
## VIT_18s0157g00180  -  S-adenosylmethionine carrier 1
## VIT_05s0020g00400  -  Galactosyltransferase family protein
## VIT_09s0002g01040  -  Subtilase
## VIT_15s0046g00120  -  Unknown protein
## VIT_13s0139g00360  -  Unknown protein
## VIT_17s0000g06880  -  Heparanase protein 2 precursor
## VIT_06s0009g00880  -  Axial regulator YABBY2
## VIT_15s0046g00040  -  Calcium-transporting ATPase 2, endoplasmic reticulum-type ECA2
## VIT_11s0016g05460  -  BRI1-KD interacting protein
## VIT_13s0019g04300  -  Ribosomal protein L12 (RPL12A) 60S
## VIT_07s0005g04730  -  No hit
## VIT_07s0141g00590  -  Magnesium transporter CorA-like family protein (MRS2-2)
## VIT_18s0001g14100  -  ABA-responsive protein (HVA22)HVA22H
## VIT_09s0002g08420  -  Unknown
## VIT_11s0016g02960  -  Glucan endo-1,3-beta-glucosidase 4 precursor
## VIT_04s0008g03210  -  Lachrymatory factor synthase
## VIT_08s0007g03620  -  Ribosomal protein P2 (RPP2A) acidic 60S
## VIT_01s0011g02740  -  Phosphoenolpyruvate carboxylase
## VIT_14s0128g00030  -  Unknown protein
## VIT_18s0001g12100  -  Auxilin
## VIT_08s0007g07920  -  Unknown protein
## VIT_07s0129g00960  -  Protein kinase pto
## VIT_04s0023g00310  -  CBS domain-containing protein
## VIT_03s0063g01050  -  RNA recognition motif (RRM)-containing protein
## VIT_09s0018g01240  -  Balbiani ring 1
## VIT_06s0009g01640  -  Receptor protein kinase
## VIT_08s0007g07630  -  Tryptophan/tyrosine permease family
## VIT_02s0025g00310  -  Ribosomal protein S10 (RPS10C) 40S
## VIT_13s0320g00060  -  Carboxyl-terminal peptidase
## VIT_01s0010g01390  -  Phototropic-responsive NPH3
## VIT_12s0028g01740  -  DNAJ heat shock N-terminal domain-containing protein
## VIT_01s0146g00070  -  11-beta-hydroxysteroid dehydrogenase
## VIT_18s0117g00080  -  R protein L6
## VIT_01s0011g00110  -  putative MADS-box sepallata 4 (VviSEP4)
## VIT_06s0004g06050  -  Thioesterase family
## VIT_17s0000g03130  -  Unknown protein
## VIT_17s0000g06680  -  Unknown protein
## VIT_05s0020g04510  -  GDP-mannose 3,5-epimerase 1
## VIT_17s0000g03520  -  Receptor kinase RK20-1
## VIT_05s0020g00870  -  UbiE/COQ5 methyltransferase
## VIT_06s0004g05430  -  Tropinone reductase
## VIT_19s0014g00980  -  Gamma-glutamyl hydrolase precursor
## VIT_11s0016g03360  -  Lipase-related
## VIT_12s0057g00980  -  Phytochrome defective C (PHYC)
## VIT_01s0011g05480  -  Dynein light subunit lc6, flagellar outer arm
## VIT_08s0056g01520  -  Acetylglucosaminyltransferase
## VIT_08s0007g00930  -  Regulator of chromosome condensation (RCC1)
## VIT_17s0000g05840  -  Calmodulin binding IQD31 (IQ-domain 31)
## VIT_14s0060g00460  -  Receptor-like protein kinase
## VIT_14s0108g01560  -  Alpha-1,4 glucan phosphorylase, L isozyme, chloroplast precursor
## VIT_02s0154g00500  -  VAP27-1 (VAMP/synaptobrevin-associated protein 27-1)
## VIT_01s0010g02750  -  Lipoxygenase
## VIT_09s0002g02290  -  Beta-galactosidase
## VIT_08s0007g01350  -  No hit
```

```
## VIT_02s0025g01080  -  No hit
## VIT_14s0171g00300  -  4-coumarate-CoA ligase
## VIT_19s0027g00080  -  Kinesin motor protein
## VIT_17s0000g02010  -  Atypical receptor kinase MARK
## VIT_19s0014g03250  -  Beta-mannosidase 4
## VIT_14s0060g00690  -  ADP-ribosylation factor A1B
## VIT_08s0040g03150  -  L-ascorbate peroxidase 1, cytosolic (APX1)
## VIT_01s0244g00140  -  Aspartate kinase
## VIT_01s0010g02670  -  3-oxoacyl-[acyl-carrier-protein] reductase, chloroplast
## VIT_02s0012g00460  -  Glycine-rich protein
## VIT_18s0001g11970  -  Unknown protein
## VIT_00s0878g00020  -  Stachyose synthase precursor
## VIT_13s0019g02230  -  PAP/25A associated domain containing protein
## VIT_16s0039g01560  -  PLATZ transcription factor
## VIT_12s0028g02350  -  basic helix-loop-helix (bHLH) family
## VIT_14s0036g01060  -  Unknown protein
## VIT_02s0154g00350  -  L-lactate dehydrogenase A
## VIT_19s0014g02900  -  Ring finger protein 185
## VIT_18s0117g00190  -  Unknown protein
## VIT_03s0038g02930  -  Tetracycline transporter protein
## VIT_00s0583g00030  -  Sphingolipid delta 4 desaturase DES-1
## VIT_05s0094g00510  -  Unknown protein
## VIT_08s0007g05060  -  ABC Transporter (VvMDR17 - VvABCB17)
## VIT_17s0000g08900  -  LRR receptor-like kinase 2
## VIT_18s0157g00020  -  GIGANTEA protein
## VIT_18s0041g01350  -  Receptor-like protein kinase HAIKU2
## VIT_06s0004g01910  -  ADP,ATP carrier protein
## VIT_10s0003g02470  -  SRG1 (senescence-related gene 1) oxidoreductase
## VIT_05s0029g01150  -  Elongation factor 1-alpha 1
## VIT_14s0171g00170  -  Unknown protein
## VIT_19s0014g03240  -  Beta-mannosidase 4
## VIT_09s0018g01900  -  PEPR1 (PEP1 receptor 1)
## VIT_15s0021g01130  -  Rho GTPase; Rho GTP binding protein
## VIT_02s0012g00990  -  LOL1 (LSD ONE like 1)
## VIT_02s0025g03700  -  ATP binding protein
## VIT_08s0056g01120  -  MATE efflux family protein
## VIT_19s0085g01110  -  Aspartic Protease (VvAP46)
## VIT_05s0020g02690  -  Copper-binding family protein
## VIT_01s0137g00650  -  Unknown protein
## VIT_09s0018g00520  -  Plastocyanin domain-containing protein
## VIT_14s0171g00130  -  Ribulose-phosphate 3-epimerase
## VIT_11s0052g01570  -  MATE efflux family protein
## VIT_12s0059g00230  -  Epoxide hydrolase 2
## VIT_17s0000g00530  -  Armadillo/beta-catenin repeat
## VIT_01s0026g02420  -  Unknown protein
## VIT_01s0026g00270  -  K+ uptake permease 6
## VIT_08s0007g08450  -  LRR receptor-like kinase 2
## VIT_19s0015g00300  -  KCO1 (two pore K channel)
## VIT_09s0070g00380  -  Unknown protein
## VIT_07s0031g00250  -  Transporter-related
## VIT_08s0056g01000  -  MATE efflux family protein
## VIT_19s0014g03460  -  Unknown protein
## VIT_19s0014g03190  -  Unknown protein
## VIT_04s0023g01630  -  Steroid 22-alpha-hydroxylase (CYP90B1) (DWF4)
## VIT_08s0058g00810  -  D-alanine--D-alanine ligase
```

```
## VIT_10s0003g00040  -  Dof zinc finger protein DOF1.4
## VIT_06s0004g04610  -  fasciclin arabinogalactan-protein (FLA4)
## VIT_07s0005g02120  -  Cyclase/dehydrase
## VIT_14s0030g01360  -  Kinesin motor protein
## VIT_15s0021g00130  -  RPP13 (recognition of peronospora parasitica 13)
## VIT_18s0072g01040  -  Invertase, neutral/alkaline
## VIT_05s0077g01130  -  Seed maturation protein PM36
## VIT_14s0060g01570  -  Kinesin motor protein
## VIT_15s0046g02820  -  R protein  PRF  disease resistance protein
## VIT_14s0083g01210  -  feronia receptor-like kinase
## VIT_17s0000g07640  -  Mannose-1-phosphate guanyltransferase
## VIT_02s0488g00020  -  Palmitoyl-protein thioesterase 1 precursor
## VIT_01s0011g02470  -  Calcium-binding protein CML
## VIT_11s0052g00970  -  AAA-type ATPase
## VIT_19s0015g01660  -  Phenylalanine-tRNA ligase
## VIT_02s0025g01740  -  Auxin response factor 9
## VIT_08s0056g00570  -  Serine/threonine protein kinase activity
## VIT_06s0009g00810  -  Periplasmic beta-glucosidase precursor
## VIT_01s0026g01790  -  Calmodulin binding protein
## VIT_15s0021g00880  -  ferredoxin-related
## VIT_00s2077g00020  -  Alkaline alpha galactosidase II
## VIT_14s0030g01790  -  Ndr family protein
## VIT_08s0007g03710  -  ABC Transporter (VvPDR23 - VvABCG53)
## VIT_17s0000g00290  -  Alpha-mannosidase precursor lysosomal
## VIT_15s0046g02420  -  Aquaporin TMP-C
## VIT_10s0003g02460  -  SRG1 (senescence-related gene 1) oxidoreductase
## VIT_08s0007g03670  -  Lipase GDSL
## VIT_04s0008g00950  -  Calcineurin B protein 10
## VIT_06s0080g00810  -  lycopene ß-cyclase (LBCY2) (VvLBCY1)
```

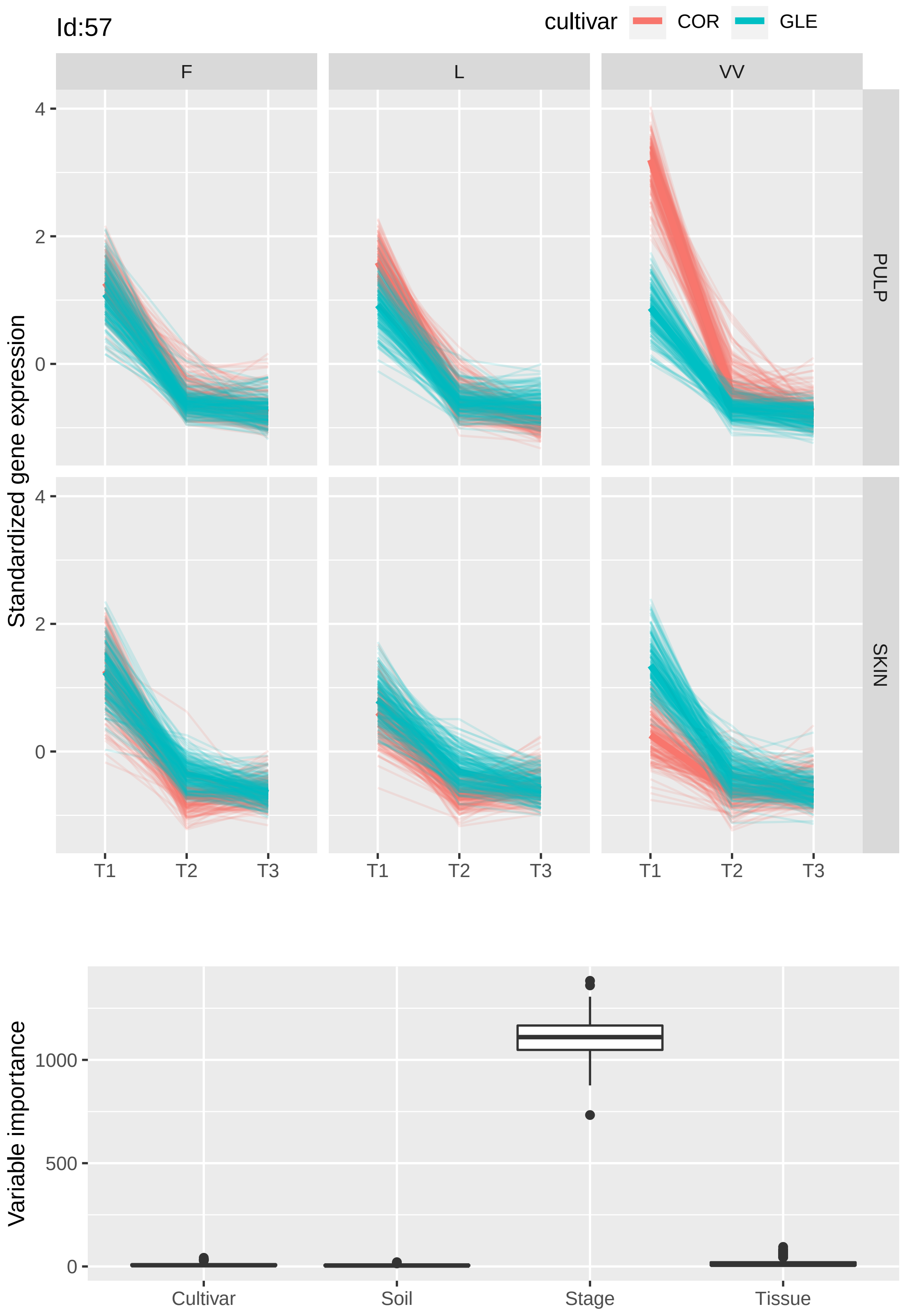
Id:57
cultivar
COR
GLE
F
L
VV
PULP
SKIN
Standardized gene expression
4
2
0
T1
T2
T3
Variable importance
1000
500
0
Cultivar
Soil
Stage
Tissue

Cluster no. 3

```
## Number of genes in the cluster: 166
## Homogeneity Index:    0.88
## Variable importance for Stage:     Rank =   3  - Median =   1100
## Variable importance for Cultivar:  Rank =  96  - Median =   7.12
## Variable importance for Tissue:    Rank =  88  - Median =  15.45
## Variable importance for Soil:      Rank =  57  - Median =   5.35
##
## Gene ID               Gene Annotation
## VIT_18s0001g14360  -  Tubulin beta-1 chain
## VIT_05s0049g01860  -  Receptor-like protein kinase
## VIT_08s0007g07400  -  Glutamate dehydrogenase 1
## VIT_10s0003g00220  -  Unknown
## VIT_16s0022g00670  -  Vacuolar invertase 1, GIN1
## VIT_06s0061g00680  -  Unknown protein
## VIT_17s0000g03790  -  Unknown protein
## VIT_09s0002g08960  -  EREBP-4
## VIT_10s0116g00350  -  Unknown protein
## VIT_12s0028g03350  -  Squamosa promoter-binding protein (VvSBP11)
## VIT_09s0002g04360  -  Thylakoid membrane phosphoprotein 14 kda, chloroplast precursor
## VIT_16s0022g02200  -  Subtilase
## VIT_14s0060g01580  -  Protein phosphatase 2C
## VIT_02s0025g03240  -  Protein kinase
## VIT_06s0004g00720  -  Glucan endo-1,3-beta-glucosidase 4 precursor
## VIT_06s0004g04320  -  Alcohol dehydrogenase 3
## VIT_00s0370g00070  -  Epoxide hydrolase
## VIT_13s0019g02330  -  GDP-mannose pyrophosphorylase (GMP1)
## VIT_13s0019g04660  -  Amino acid permease
## VIT_15s0045g00370  -  Phosphatidylglycerol specific phospholipase C
## VIT_04s0008g04250  -  Aspartate aminotransferase
## VIT_12s0034g01860  -  Phosphogluconate dehydrogenase
## VIT_14s0030g00710  -  Na/H antiporter
## VIT_17s0000g06780  -  Dimethylaniline monooxygenase
## VIT_18s0001g12660  -  TUBBY like protein 6 TLP6
## VIT_05s0020g03170  -  Lipoxygenase
## VIT_08s0007g05530  -  Glutaredoxin
## VIT_15s0046g02750  -  R protein  PRF  disease resistance protein
## VIT_18s0001g12170  -  CYP72A1
## VIT_05s0020g03320  -  Copper amine oxidase
## VIT_01s0150g00250  -  Peptide transporter PTR2-B
## VIT_05s0094g00930  -  Phosphoglucomutase/phosphomannomutase C terminal
## VIT_01s0026g01950  -  Homeobox-leucine zipper protein HB13
## VIT_01s0011g06200  -  Zinc finger protein 4
## VIT_13s0067g01930  -  Unknown protein
## VIT_01s0011g04450  -  MATE efflux family protein
## VIT_03s0088g00050  -  Serine carboxypeptitase 1
## VIT_08s0007g04590  -  UGT73C2 (UDP-glucosyl transferase 73C2)
## VIT_00s0286g00020  -  Curculin (mannose-binding) lectin
## VIT_00s0780g00020  -  Glucan endo-1,3-beta-glucosidase 5 precursor
## VIT_00s0189g00050  -  No hit
## VIT_08s0007g03820  -  PTL (PETAL LOSS)
## VIT_03s0063g00210  -  Receptor protein kinase
## VIT_04s0023g01640  -  Steroid 22-alpha-hydroxylase (CYP90B1) (DWF4)
## VIT_06s0004g04310  -  Alcohol dehydrogenase
## VIT_19s0093g00310  -  Glutathione S-transferase 8 GSTU19
```

```
## VIT_06s0004g03240  -  Elongation factor 1-alpha 1
## VIT_08s0007g01760  -  Steroid 5 alpha reductase DET2
## VIT_05s0020g01420  -  FPF1 (flowering promoting factor 1)
## VIT_00s0301g00050  -  3'(2') 5'-bisphosphate nucleotidase
## VIT_08s0032g01110  -  Axial regulator YABBY2
## VIT_11s0016g01020  -  flavonoid 3-monooxygenase
## VIT_12s0028g01350  -  RKF1 (receptor-like kinase in flowers 1)
## VIT_03s0038g04570  -  ADP-glucose pyrophosphorylase large subunit 1
## VIT_03s0038g00440  -  Unknown protein
## VIT_19s0014g04400  -  S-locus protein kinase
## VIT_14s0108g01420  -  DEFENSE NO death 1
## VIT_13s0074g00690  -  ABC Transporter (VvPDR26 - VvABCG56)
## VIT_03s0038g01520  -  R protein  PRF  disease resistance protein
## VIT_04s0023g01170  -  Unknown protein
## VIT_03s0038g03800  -  Protein kinase APK1B
## VIT_19s0014g02320  -  Unknown protein
## VIT_13s0074g00040  -  Phytol kinase 2, chloroplast precursor
## VIT_07s0031g01850  -  BRI1 protein
## VIT_18s0001g00610  -  Rho GTPase activator
## VIT_14s0171g00120  -  Unknown protein
## VIT_03s0063g00180  -  U-box domain-containing protein
## VIT_07s0031g02170  -  Glutamate receptor GLR3.6
## VIT_00s0229g00100  -  Unknown
## VIT_19s0015g00440  -  Nuclear transcription factor Y subunit B-3
## VIT_16s0098g00720  -  Lectin protein kinase
## VIT_19s0027g01890  -  Amino acid permease
## VIT_09s0002g03860  -  RPS5 (resistant to p. syringae 5)
## VIT_14s0030g00250  -  Sugar transporter ERD6-like 3
## VIT_13s0019g01610  -  XH/XS domain-containing protein
## VIT_14s0060g01270  -  Pseudouridine synthase
## VIT_02s0025g00990  -  ABC Transporter (VvMRP16 - VvABCC16)
## VIT_02s0025g01050  -  Ubiquitin-conjugating enzyme E2 D/E
## VIT_07s0005g06420  -  Thioredoxin family
## VIT_18s0001g09130  -  Armadillo/beta-catenin repeat
## VIT_02s0154g00250  -  Oxysterol binding protein
## VIT_04s0023g01880  -  Zinc Finger Homeodomain Transcription Factor (VvZHD3)
## VIT_03s0017g01420  -  Reticuline oxidase
## VIT_13s0019g03210  -  Lectin-like protein
## VIT_09s0002g01750  -  Unknown protein
## VIT_08s0007g00410  -  Myb domain protein 91
## VIT_16s0050g02400  -  ERF/AP2 Gene Family (VvERF054)
## VIT_10s0003g02000  -  RKF1 (receptor-like kinase in flowers 1)
## VIT_07s0151g00310  -  NIMA protein kinase NEK1
## VIT_15s0046g01450  -  Alpha-L-fucosidase
## VIT_00s0983g00010  -  Abhydrolase domain-containing protein
## VIT_04s0079g00200  -  Auxin response factor 8
## VIT_08s0007g06200  -  Rhodanese domain-containing protein
## VIT_11s0016g00870  -  Strictosidine synthase (YLS2)
## VIT_11s0016g01300  -  Myb protein (VvMYBPAR)
## VIT_09s0054g01620  -  myb family
## VIT_06s0004g05170  -  Lectin protein kinase
## VIT_05s0077g02150  -  Chalcone reductase
## VIT_05s0077g02350  -  No hit
## VIT_03s0180g00070  -  Cyclase
## VIT_14s0060g02250  -  Unknown protein
```

```
## VIT_06s0061g00150  -  Zinc finger (CCCH-type) family protein
## VIT_03s0038g04490  -  Diaminopimelate decarboxylase
## VIT_01s0127g00700  -  Unknown protein
## VIT_05s0094g00480  -  Ethylene-responsive protein
## VIT_09s0002g01380  -  ATMYB66/WER/WER1 (WEREWOLF 1)
## VIT_11s0118g00780  -  Zinc finger (C3HC4-type ring finger)
## VIT_04s0008g01100  -  cytochrome P450 (CYP711A1; MAX1) (VvMAX1)
## VIT_05s0094g00920  -  formin protein AHF1
## VIT_00s0665g00030  -  Unknown protein
## VIT_18s0001g15000  -  ACT domain containing protein (ACR4)
## VIT_01s0011g04060  -  Receptor-like kinase RHG1
## VIT_06s0080g00420  -  Glucan endo-1,3-beta-glucosidase 7 precursor
## VIT_12s0028g03100  -  GPRI1 (GOLDEN2 1)
## VIT_15s0046g02560  -  basic helix-loop-helix (VvMYCA1)
## VIT_18s0117g00260  -  Co-chaperone-curved DNA binding protein A
## VIT_07s0129g00330  -  Lateral organ boundaries protein 39
## VIT_04s0023g02690  -  Beta-galactosidase
## VIT_07s0129g01000  -  F-box family protein
## VIT_02s0012g00360  -  1-aminocyclopropane-1-carboxylate oxidase
## VIT_16s0115g00340  -  UDP-glucose: anthocyanidin 5,3-O-glucosyltransferase
## VIT_16s0050g00100  -  Myosin-related
## VIT_02s0154g00240  -  Oxysterol binding protein
## VIT_14s0006g01600  -  Unknown
## VIT_04s0023g00580  -  Auxin-responsive SAUR32
## VIT_04s0023g02070  -  Trichohyalin AUL1 (auxin-like 1 protein)
## VIT_16s0098g00730  -  Ribosomal protein L9 (RPL90A/C) 60S
## VIT_17s0000g04850  -  OBF binding protein 4
## VIT_14s0030g01160  -  R protein disease resistance protein
## VIT_14s0068g00330  -  PTF1 (plastid transcription factor 1) TCP13
## VIT_09s0018g01950  -  Cellulose synthase CSLC05
## VIT_00s0199g00220  -  Phosphofructokinase
## VIT_06s0004g00620  -  Accelerated cell death 1 ACD1
## VIT_18s0001g06220  -  F-box protein (FBW2)
## VIT_04s0079g00160  -  Auxin response factor 8
## VIT_16s0050g00760  -  Short-chain dehydrogenase/reductase (SDR)
## VIT_14s0030g01870  -  NIMA protein kinase
## VIT_02s0025g01980  -  Cellulose synthase CSLG3
## VIT_03s0063g02640  -  Beta-ketoacyl-CoA synthase
## VIT_01s0026g01450  -  DnaJ homolog, subfamily B, member 4
## VIT_08s0007g08520  -  Unknown protein
## VIT_01s0150g00370  -  C2 domain-containing protein
## VIT_14s0030g01030  -  Alcohol dehydrogenase
## VIT_14s0030g00260  -  Sugar transporter ERD6-like 3
## VIT_03s0091g01100  -  Unknown protein
## VIT_02s0025g01170  -  COP1-interactive protein 1 / CIP1
## VIT_14s0108g00350  -  3-dehydroquinate synthase
## VIT_01s0150g00540  -  Unknown protein
## VIT_16s0050g01420  -  Coiled-coil domain-containing protein 111
## VIT_03s0038g03810  -  Serine/threonine Protein kinase BNK1
## VIT_07s0005g02470  -  Cellulose synthase CESA3
## VIT_19s0027g01690  -  Disease resistance protein RPS2
## VIT_00s1209g00010  -  ADP,ATP carrier protein
## VIT_00s0265g00090  -  Unknown protein
## VIT_17s0000g02490  -  No hit
```

```
## VIT_05s0049g01320  -  Unknown protein
## VIT_00s1584g00010  -  Elongation factor 1-alpha
## VIT_13s0064g00350  -  No hit
## VIT_07s0005g03190  -  ERF/AP2 Gene Family (VvERF096)
## VIT_14s0030g01050  -  R protein disease resistance protein
## VIT_05s0020g00910  -  F-box family protein
## VIT_10s0003g03170  -  CYP710A1 C-22 sterol desaturase
## VIT_14s0030g01170  -  CC-NBS-LRR class
## VIT_07s0031g00450  -  Basic helix-loop-helix protein SPATULA
## VIT_14s0030g01040  -  No hit
## VIT_04s0008g04060  -  RD22
```

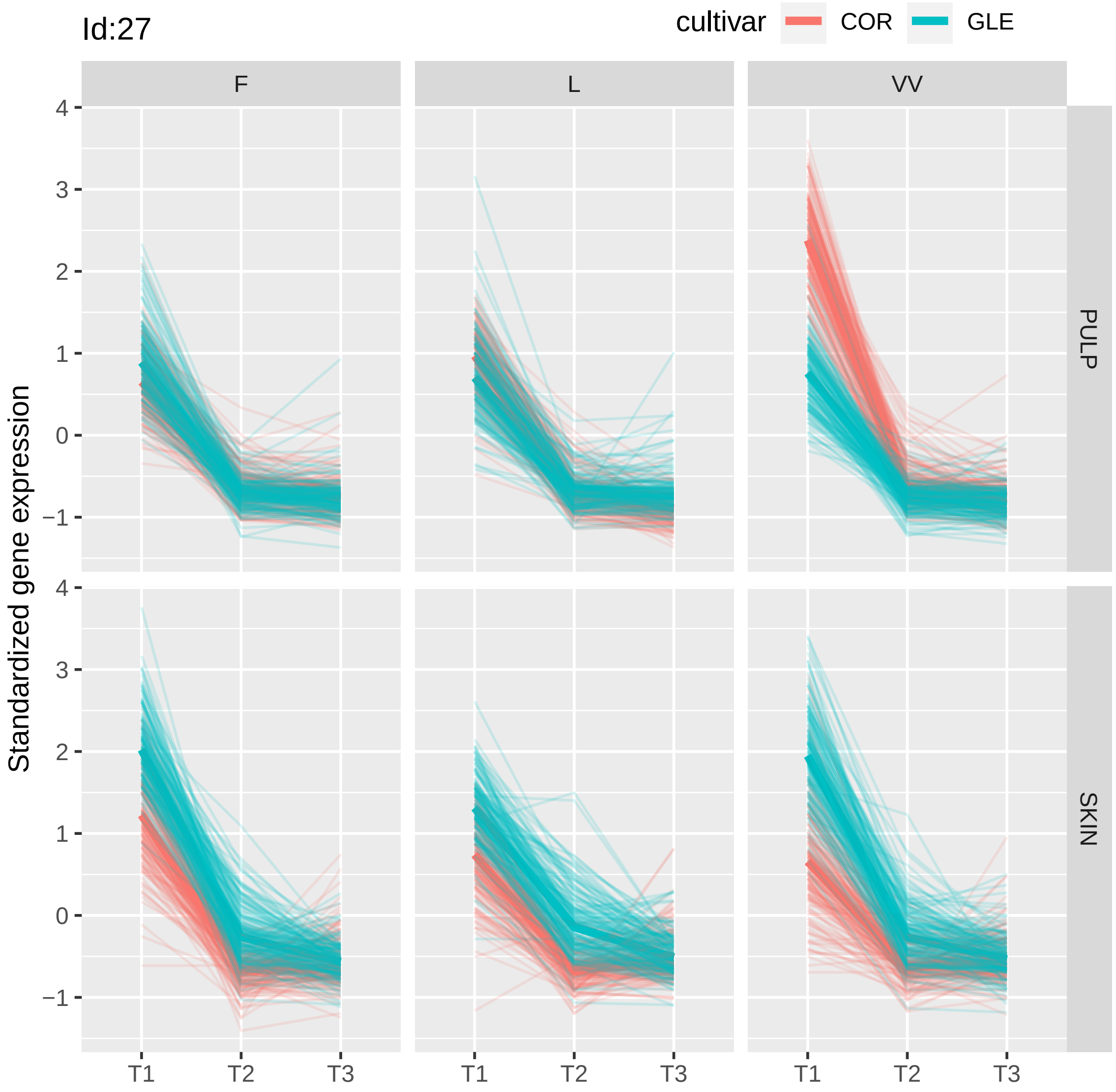
Id:27
cultivar
COR
GLE
F
L
VV
PULP
SKIN
Standardized gene expression
4
3
2
1
0
−1
T1
T2
T3

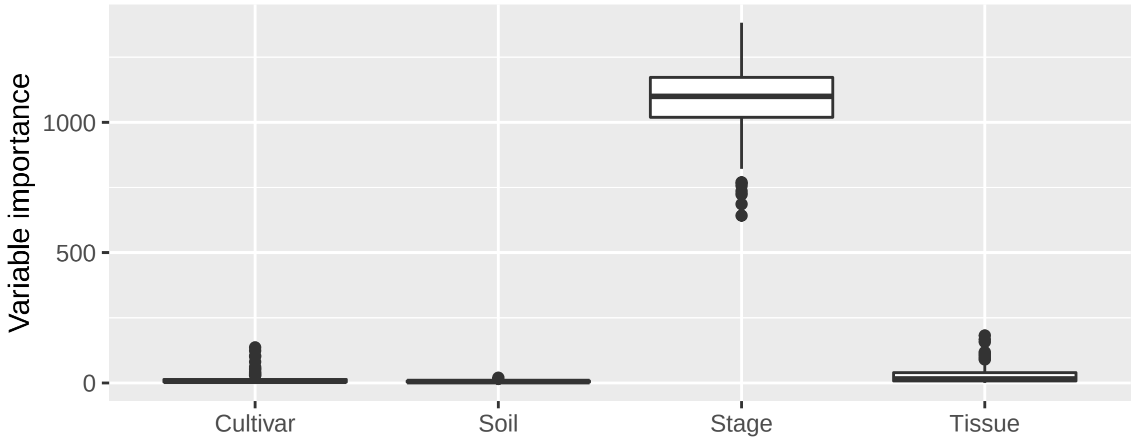
Variable importance
1000
500
0
Cultivar
Soil
Stage
Tissue

Cluster no. 4

```
## Number of genes in the cluster: 66
## Homogeneity Index:    0.89
## Variable importance for Stage:     Rank =   4  - Median =  986.2
## Variable importance for Cultivar:  Rank = 102  - Median =   4.29
## Variable importance for Tissue:    Rank =  81  - Median =  23.87
## Variable importance for Soil:      Rank =  79  - Median =   3.92
##
## Gene ID               Gene Annotation
## VIT_06s0080g01090  -  CCR4-NOT transcription complex subunit 7/8
## VIT_04s0023g02650  -  CYP90D1 ROT3 (ROTUNDIFOLIA 3) steroid hydroxylase
## VIT_18s0076g00250  -  Sucrose-proton symporter 2 SUC2 (SUT2-2)
## VIT_01s0011g06330  -  Microtubule associated protein (MAP65-6)
## VIT_01s0026g02270  -  Kinase
## VIT_00s0187g00130  -  Tonoplast dicarboxylate transporter
## VIT_05s0020g03180  -  Photosystem I reaction center subunit II, chloroplast precursor
## VIT_07s0104g00790  -  Endonuclease
## VIT_18s0089g00010  -  Disease resistance protein
## VIT_00s0233g00010  -  No hit
## VIT_14s0006g02760  -  Unknown protein
## VIT_10s0003g02890  -  LHCII type I CAB-1
## VIT_16s0013g01070  -  ERF/AP2 Gene Family (VvERF085)
## VIT_19s0014g00100  -  Chalcone isomerase 3
## VIT_00s2188g00010  -  Tonoplast dicarboxylate transporter
## VIT_19s0014g03520  -  fiber protein
## VIT_18s0089g01170  -  LHCB5 (light harvesting complex of photosystem ii 5)
## VIT_12s0059g00190  -  Expansin  (VvEXPB2)
## VIT_02s0025g01040  -  Receptor protein kinase
## VIT_01s0011g04550  -  Unknown protein
## VIT_13s0156g00060  -  Serine/threonine-protein phosphatase PP2A catalytic subunit
## VIT_17s0000g09610  -  CYP71D10
## VIT_17s0000g05640  -  Nitrate transporter 1:2
## VIT_04s0008g01120  -  Glutaredoxin
## VIT_17s0000g06350  -  LHCA4 (Photosystem I light harvesting complex gene 4)
## VIT_08s0007g05350  -  Haloacid dehalogenase hydrolase
## VIT_19s0014g01050  -  UNE1 (unfertilized embryo sac 1)
## VIT_12s0059g00270  -  NADPH-protochlorophyllide oxidoreductase
## VIT_03s0017g02260  -  Protease inhibitor/seed storage/lipid transfer protein (LTP)
## VIT_04s0044g01410  -  Photosystem I reaction center subunit N (PSAN)
## VIT_07s0031g02210  -  No hit
## VIT_01s0137g00210  -  Photosystem II core complex proteins psbY, chloroplast precursor
## VIT_02s0025g04460  -  ERF/AP2 Gene Family (VvERF021)
## VIT_16s0098g01000  -  No hit
## VIT_19s0014g03160  -  NADPH:protochlorophyllide oxidoreductase
## VIT_08s0040g00540  -  VQ motif-containing protein
## VIT_07s0031g02160  -  Protein phosphatase 2C DBP
## VIT_15s0024g00040  -  LHCA3 (Photosystem I light harvesting complex gene 3)
## VIT_15s0021g02510  -  GATA transcription factor 2
## VIT_10s0003g04350  -  Photosystem I subunit X (PSAK)
## VIT_00s0615g00030  -  Mannitol dehydrogenase (ELI3-1)
## VIT_10s0003g02900  -  LHCII type I CAB-1
## VIT_07s0031g02200  -  Auxin efflux carrier
## VIT_19s0014g02240  -  Ethylene responsive element binding factor 4
## VIT_16s0098g01900  -  Pectinesterase family
## VIT_06s0080g00920  -  Photosystem I subunit O (PSAO)
```

```
## VIT_13s0019g04140  -  LHCA1
## VIT_12s0055g01110  -  LHCB6 (light harvesting complex PSII)
## VIT_08s0007g02020  -  Proton-dependent oligopeptide transport (POT) family protein
## VIT_17s0053g00070  -  Metal-nicotianamine transporter YSL5
## VIT_16s0013g00980  -  ERF/AP2 Gene Family (VvERF082)
## VIT_02s0154g00090  -  Vacuolar invertase 2, GIN2
## VIT_03s0063g00770  -  Carboxyesterase 12; CXE12
## VIT_12s0035g02150  -  ferric reduction oxidase 7 FRO7
## VIT_14s0083g01100  -  Alpha-1,4-glucan-protein synthase 1
## VIT_04s0008g05510  -  SYNC1 protein, related
## VIT_16s0013g00950  -  ERF/AP2 Gene Family (VvERF080)
## VIT_16s0013g01050  -  ERF/AP2 Gene Family (VvERF107)
## VIT_02s0154g00260  -  Nitrate transporter
## VIT_03s0063g00460  -  ERF/AP2 Gene Family (VvERF117)
## VIT_17s0053g00380  -  Metal-nicotianamine transporter YSL7
## VIT_11s0016g01850  -  Anthocyanidin-3-glucoside rhamnosyltransferase
## VIT_07s0005g02220  -  LHCII type I CAB-1
## VIT_11s0016g00660  -  ERF/AP2 Gene Family (VvERF009),Dehydration Responsive Element-Binding
## VIT_02s0154g00520  -  Aspartyl protease
## VIT_06s0004g05780  -  UDP-glucoronosyl and UDP-glucosyl transferase
```

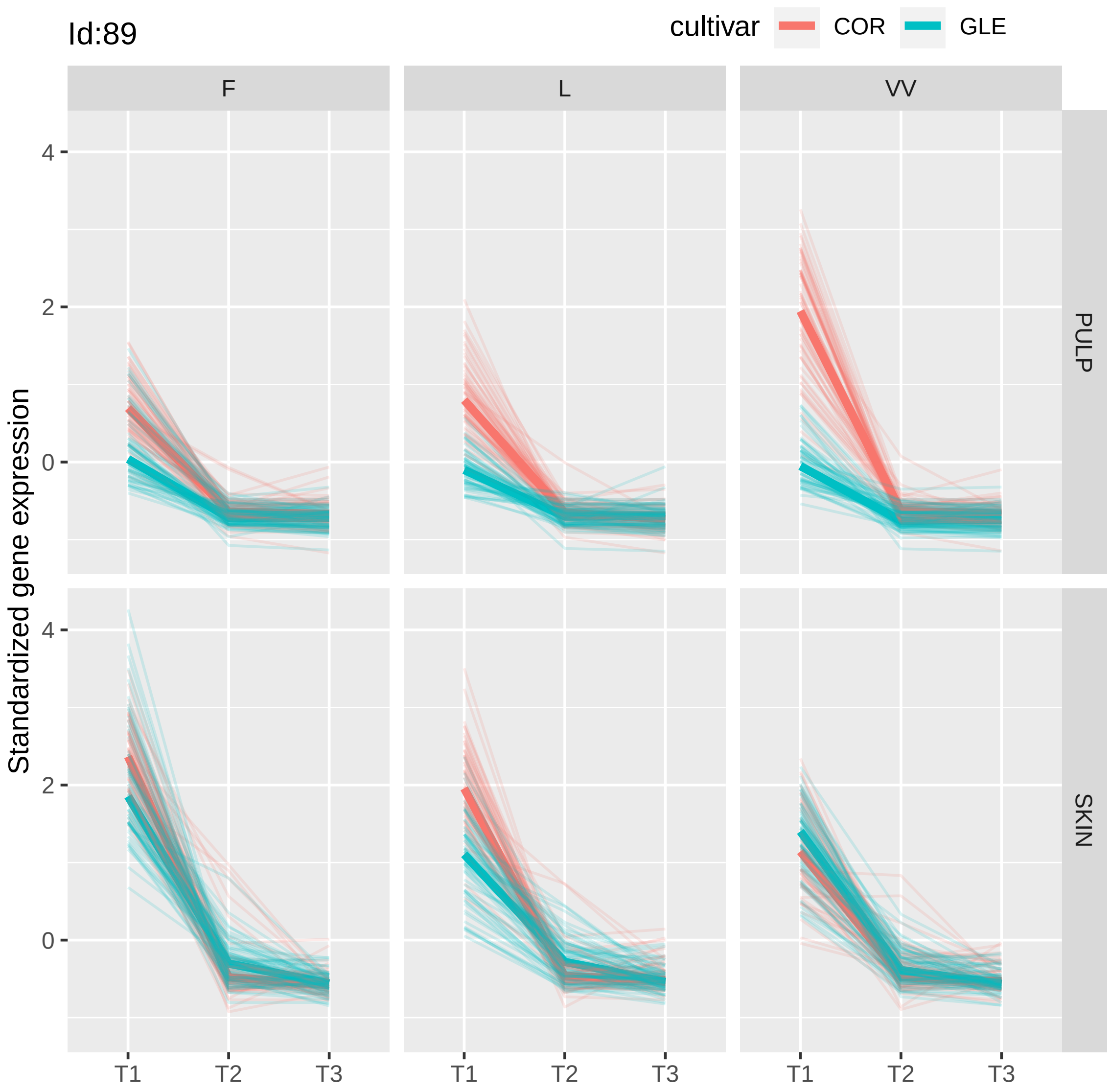
Id:89
cultivar
COR
GLE
F
L
VV
PULP
SKIN
Standardized gene expression
4
2
0
T1
T2
T3

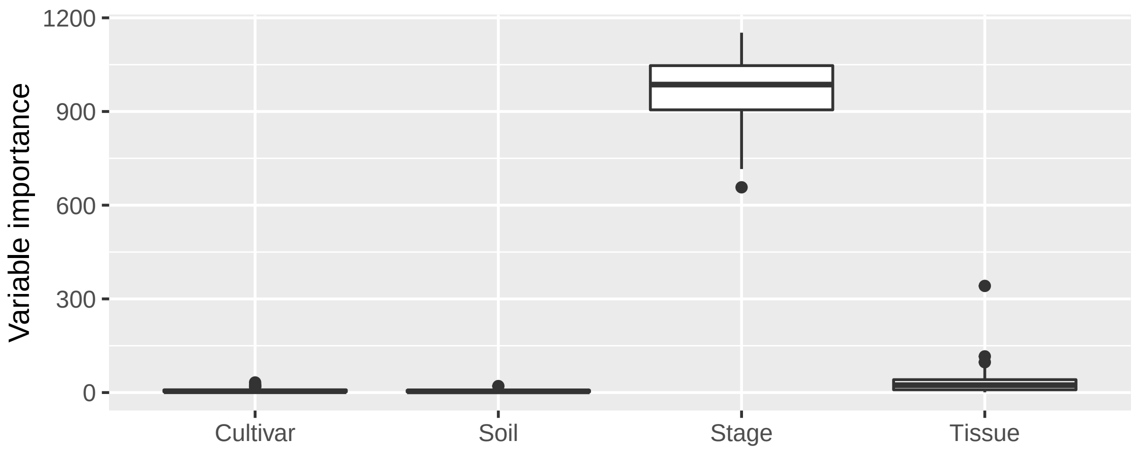
Variable importance
1200
900
600
300
0
Cultivar
Soil
Stage
Tissue

Cluster no. 5

```
## Number of genes in the cluster: 98
## Homogeneity Index:    0.86
## Variable importance for Stage:     Rank =   5  - Median =  985.4
## Variable importance for Cultivar:  Rank =  83  - Median =  10.46
## Variable importance for Tissue:    Rank =  95  - Median =  11.12
## Variable importance for Soil:      Rank =  67  - Median =   4.92
##
## Gene ID                Gene Annotation
## VIT_15s0048g02870  -  Homeobox-leucine zipper protein HB-7 (VvATHB-8)
## VIT_06s0004g05390  -  Tropinone reductase
## VIT_02s0087g00580  -  Nitrate transporter
## VIT_02s0025g02590  -  Homeobox-leucine zipper protein ATHB-12 (VvATHB-2)
## VIT_08s0007g04820  -  Pectate lyase
## VIT_07s0129g00730  -  CYP81E1 Isoflavone 2'-hydroxylase
## VIT_14s0068g00750  -  C2 domain-containing protein
## VIT_15s0021g00940  -  Senescence-associated protein
## VIT_10s0003g01310  -  Unknown protein
## VIT_18s0001g12190  -  CYP721A1
## VIT_00s0533g00050  -  Extensin
## VIT_18s0001g00280  -  OPCL1 (OPC-8:0 COA ligase1)
## VIT_05s0020g02210  -  Histidine-containing phosphotransfer protein
## VIT_16s0050g01930  -  EIX receptor 2
## VIT_18s0001g12180  -  CYP721A1
## VIT_06s0004g05950  -  Unknown protein
## VIT_18s0001g09040  -  LIM domain protein WLIM1
## VIT_17s0000g02470  -  Thaumatin
## VIT_16s0050g00240  -  UDP-glycosyltransferase 88A4
## VIT_08s0007g07230  -  VvMyb5a
## VIT_13s0067g01150  -  Unknown protein
## VIT_01s0011g00590  -  Acclimation of photosynthesis to environment
## VIT_12s0057g00220  -  Unknown protein
## VIT_07s0191g00070  -  SNF1-related protein kinase 2.7 SNRK2.7 SRK2F
## VIT_05s0062g00630  -  UDP-glucose transferase (UGT75B2)
## VIT_06s0004g04510  -  Heat shock protein 70
## VIT_08s0007g04800  -  Carbonic anhydrase precursor
## VIT_12s0057g01020  -  fasciclin-like arabinogalactan protein FLA2
## VIT_09s0002g06300  -  Unknown
## VIT_01s0011g00600  -  Acclimation of photosynthesis to environment
## VIT_12s0059g02360  -  Myb CAPRICE (VvCPC1)
## VIT_11s0016g01810  -  Unknown protein
## VIT_06s0004g00410  -  Carboxyl-terminal peptidase
## VIT_06s0004g04620  -  Unknown
## VIT_03s0088g00110  -  Serine carboxypeptidase SCPL17
## VIT_04s0044g01010  -  Pectinesterase family
## VIT_00s1194g00020  -  No hit
## VIT_15s0048g01750  -  fasciclin arabinogalactan-protein (FLA8)
## VIT_03s0091g01240  -  Serine carboxypeptidase S10 / Anthocyanin Acyl-transferase
## VIT_04s0044g01020  -  Pectinesterase family
## VIT_13s0067g02340  -  Proton-dependent oligopeptide transport (POT) family protein
## VIT_11s0016g04570  -  RKL1 (Receptor-like kinase 1)
## VIT_18s0122g00330  -  Unknown protein
## VIT_06s0004g02650  -  No hit
## VIT_00s1203g00010  -  Auxin response factor ARF6
## VIT_08s0058g00260  -  Ethylene overproducer 1 (ETO1)
```

```
## VIT_19s0015g02500  -  CYP72A1
## VIT_01s0011g05390  -  Invertase/pectin methylesterase inhibitor
## VIT_14s0060g01720  -  Dormancy/auxin associated protein
## VIT_07s0141g00020  -  Sensitive to proton rhizotoxicity 1
## VIT_08s0007g01300  -  Glyoxal oxidase
## VIT_18s0001g13350  -  Proton-dependent oligopeptide transport (POT) family protein
## VIT_16s0013g01000  -  ERF/AP2 Gene Family (VvERF084)
## VIT_13s0067g00620  -  Cinnamyl alcohol dehydrogenase
## VIT_13s0019g02040  -  Lectin protein kinase
## VIT_18s0076g00360  -  EMB1075 (embryo defective 1075) carboxy-lyase
## VIT_14s0066g00680  -  Ramosa 2
## VIT_12s0059g01250  -  Endo-1,4-beta-glucanase
## VIT_07s0104g00410  -  Endo-1,3;1,4-beta-D-glucanase precursor
## VIT_02s0025g00230  -  Unknown
## VIT_00s0199g00240  -  Pyrophosphate-fructose-6-phosphate 1-phosphotransferase
## VIT_02s0025g01200  -  Leaf senescence protein
## VIT_13s0067g02710  -  Unknown protein
## VIT_18s0001g09920  -  Cyclin delta-3 (CYCD3_1)
## VIT_07s0129g00810  -  CYP81E8
## VIT_18s0001g13420  -  SHI-related sequence 5
## VIT_16s0013g01080  -  ERF/AP2 Gene Family (VvERF086)
## VIT_15s0048g00790  -  TCH2 (touch 2)
## VIT_13s0067g03390  -  Pseudo-response regulator 7 (APRR7)
## VIT_06s0004g04270  -  Histone H2A.4 HTA12
## VIT_04s0008g05640  -  Protease inhibitor/seed storage/lipid transfer protein (LTP)
## VIT_09s0002g05180  -  No hit
## VIT_12s0059g00870  -  MAPKKK15
## VIT_13s0067g02220  -  Amino acid permease
## VIT_18s0001g09360  -  Hydrogenobyrinic acid a,c-diamide synthase
## VIT_00s0710g00020  -  SNF1-related protein kinase 2.7 SNRK2.7 SRK2F
## VIT_07s0005g03390  -  Unknown protein
## VIT_06s0004g03530  -  Nitrate excretion transporter1
## VIT_18s0001g11830  -  Calmodulin
## VIT_03s0063g02350  -  Organ-specific protein S2
## VIT_08s0007g07870  -  basic helix-loop-helix (bHLH) family
## VIT_08s0040g01800  -  BTB/POZ; NPH3
## VIT_15s0046g00170  -  VvMybPA1
## VIT_09s0096g00010  -  Disease resistance protein (NBS-LRR class)
## VIT_18s0072g01110  -  GASA like
## VIT_14s0006g03260  -  Rhomboid
## VIT_16s0050g01590  -  UDP-glucose: anthocyanidin 5,3-O-glucosyltransferase
## VIT_14s0006g02830  -  Ribosomal protein S7 (RPS7A) 40S
## VIT_12s0028g01150  -  Protein TRANSPARENT TESTA 12
## VIT_13s0073g00530  -  Beta-D-glucosidase
## VIT_15s0021g01860  -  Unknown protein
## VIT_13s0073g00550  -  Beta-D-glucosidase
## VIT_15s0046g02410  -  Aquaporin TMP-C
## VIT_12s0055g00160  -  UDP-glycosyltransferase 71E1
## VIT_08s0007g08000  -  No hit
## VIT_17s0000g05550  -  Proton-dependent oligopeptide transport (POT) family protein
## VIT_04s0008g06140  -  No hit
## VIT_04s0044g00970  -  Unknown protein
```

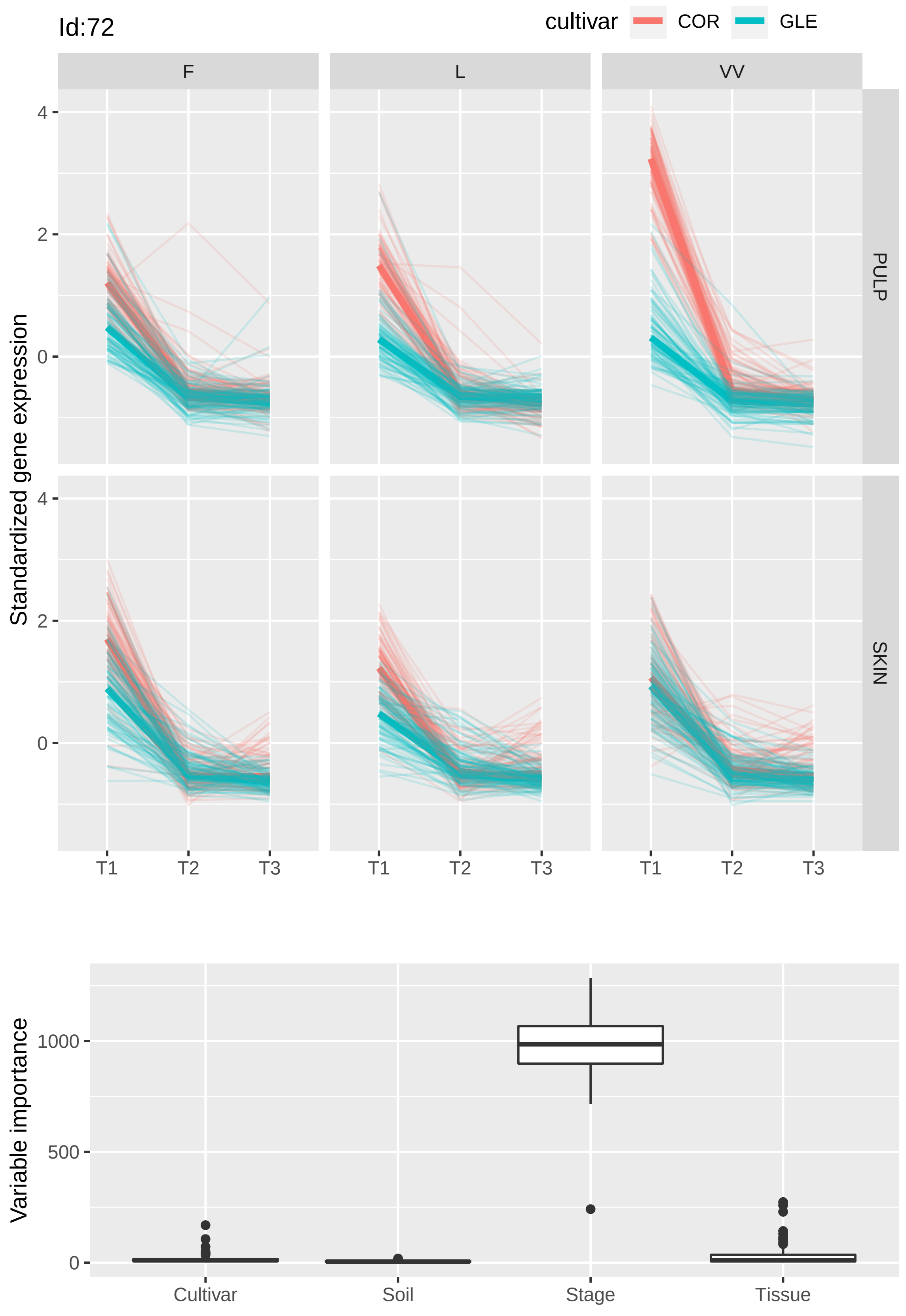

Id:72
cultivar
COR
GLE
F
L
VV
PULP
SKIN
Standardized gene expression
4
2
0
T1
T2
T3
Variable importance
1000
500
0
Cultivar
Soil
Stage
Tissue

Cluster no. 6

```
## Number of genes in the cluster: 40
## Homogeneity Index:    0.84
## Variable importance for Stage:     Rank =   6  - Median =  970.1
## Variable importance for Cultivar:  Rank =  63  - Median =  20.92
## Variable importance for Tissue:    Rank =  74  - Median =  32.31
## Variable importance for Soil:      Rank =  39  - Median =   5.97
##
## Gene ID               Gene Annotation
## VIT_14s0108g01070  -  NAC domain-containing protein (VvNAC11)
## VIT_18s0001g12690  -  Isoflavone reductase (synthesis of phytoalexines)
## VIT_01s0127g00450  -  Iron sulfur cluster assembly protein 1
## VIT_14s0108g00110  -  Serine carboxypeptidase S10
## VIT_01s0127g00460  -  Iron sulfur cluster assembly protein 1
## VIT_01s0137g00550  -  CYP71B34
## VIT_12s0028g00920  -  Glutathione S-transferase 9 GSTF9
## VIT_16s0098g01780  -  Soluble starch synthase 1, chloroplast precursor
## VIT_01s0137g00520  -  CYP71B35
## VIT_12s0134g00470  -  No hit
## VIT_06s0004g00570  -  VvMyb5b
## VIT_02s0012g01010  -  Leucine-rich repeat
## VIT_03s0180g00010  -  Cysteine proteinase
## VIT_05s0020g04380  -  Calcium-transporting ATPase 13 ACA13
## VIT_07s0005g00170  -  Rubber elongation factor (REF)
## VIT_12s0059g00880  -  WRKY Transcription Factor (VvWRKY37)
## VIT_00s0194g00110  -  4-nitrophenylphosphatase
## VIT_12s0028g03900  -  No hit
## VIT_12s0035g01420  -  Zinc finger (C3HC4-type ring finger)
## VIT_10s0116g01660  -  Lactoylglutathione lyase
## VIT_18s0001g12700  -  No hit
## VIT_02s0154g00390  -  Autophagy 8f (APG8f)
## VIT_07s0031g02820  -  Magnesium transporter CorA
## VIT_14s0060g00240  -  Myb domain protein 61
## VIT_12s0028g03890  -  Unknown protein
## VIT_08s0056g01230  -  Cycling DOF factor 2
## VIT_15s0046g01740  -  Unknown protein
## VIT_13s0064g01450  -  Electron carrier/ protein disulfide oxidoreductase
## VIT_12s0035g01410  -  No hit
## VIT_06s0004g08120  -  No hit
## VIT_10s0003g00800  -  Unknown
## VIT_10s0405g00070  -  RAB GTPase RABA5A
## VIT_05s0020g04720  -  Magnesium transporter CorA-like family protein
## VIT_13s0074g00200  -  MRNA cap guanine-N7 methyltransferase 1
## VIT_18s0041g01890  -  Unknown protein
## VIT_07s0031g02630  -  Aldehyde Dehydrogenase (VvALDH3H5)
## VIT_19s0015g00190  -  Zinc transporter ZIP11
## VIT_11s0016g00020  -  Translation initiation factor eIF-5A
## VIT_11s0016g04120  -  No hit
## VIT_12s0028g02170  -  ATP synthase protein I
```

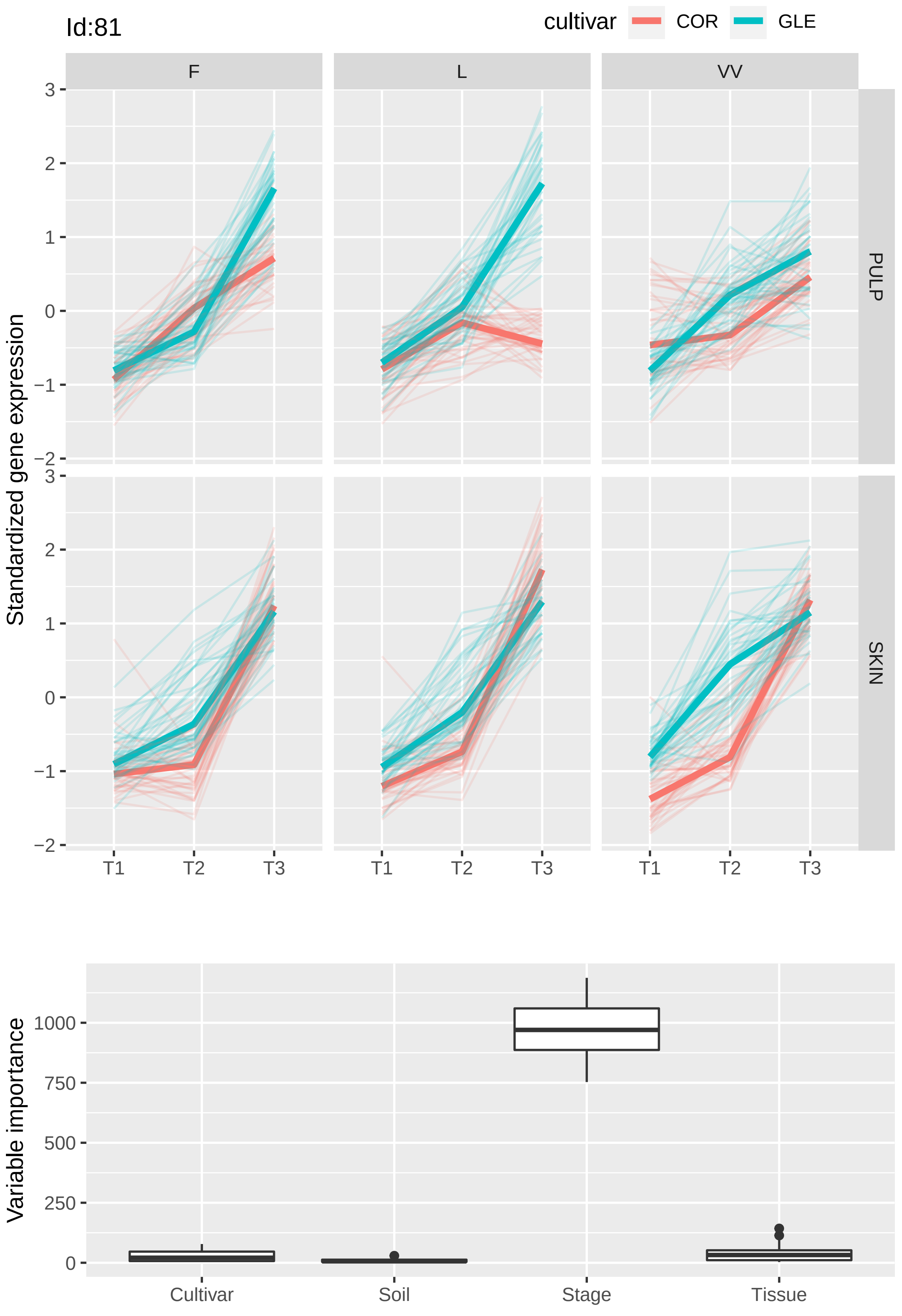
Id:81
cultivar
COR
GLE
F
L
VV
PULP
SKIN
Standardized gene expression
3
2
1
0
−1
−2
T1
T2
T3
Variable importance
1000
750
500
250
0
Cultivar
Soil
Stage
Tissue

Cluster no. 7

```
## Number of genes in the cluster: 115
## Homogeneity Index:    0.92
## Variable importance for Stage:     Rank =   7  - Median =  956.8
## Variable importance for Cultivar:  Rank =  91  - Median =   8.39
## Variable importance for Tissue:    Rank =  89  - Median =  14.97
## Variable importance for Soil:      Rank =  70  - Median =   4.66
##
## Gene ID               Gene Annotation
## VIT_01s0011g04910  -  Serine carboxypeptidase CPVL precursor
## VIT_17s0000g03010  -  Phosphatidyl serine synthase
## VIT_15s0046g03520  -  Phosphomannomutase
## VIT_16s0115g00160  -  LEM3 (ligand-effect modulator 3)
## VIT_16s0098g01720  -  Abl interactor 2 (ABIL2)
## VIT_06s0061g01370  -  Unknown protein
## VIT_08s0040g00630  -  Hydroxyproline-rich glycoprotein
## VIT_11s0016g04500  -  Protein phosphatase 2 (formerly 2A), regulatory subunit B'
## VIT_13s0019g01850  -  Transposon protein, putative, CACTA
## VIT_00s1322g00010  -  Tubulin alpha-3 chain
## VIT_15s0046g00220  -  Kinesin light chain
## VIT_00s0353g00060  -  ARK3 (Arabidopsis Receptor Kinase 3)
## VIT_08s0007g01530  -  Zinc finger (C3HC4-type ring finger)
## VIT_07s0005g04710  -  Protein kinase
## VIT_06s0004g05940  -  Uncoupling protein 1
## VIT_00s0203g00150  -  Octicosapeptide/Phox/Bem1p (PB1) domain-containing protein
## VIT_16s0039g02470  -  Chromomethylase CMT2
## VIT_16s0050g02530  -  Myb Triptychon
## VIT_00s1251g00010  -  UDP-glucosyl transferase 85A2
## VIT_14s0066g01050  -  Glycogen synthase kinase 3 beta
## VIT_08s0040g03320  -  Glutamate N-acetyltransferase
## VIT_06s0004g04130  -  Protein tyrosine phosphatase protein (PAS2)
## VIT_18s0001g12770  -  Trichohyalin AUL1 (auxin-like 1 protein)
## VIT_00s2279g00010  -  LEM3 (ligand-effect modulator 3) family protein
## VIT_00s0373g00010  -  Tubulin alpha-3 chain
## VIT_01s0026g00960  -  Unknown protein
## VIT_13s0019g01640  -  Unknown protein
## VIT_05s0062g01430  -  Glycosyl hydrolase family 17 protein
## VIT_06s0061g01310  -  Nucleobase-ascorbate transporter 12 (NAT12)
## VIT_08s0007g08470  -  24-sterol C-methyltransferase
## VIT_19s0014g01350  -  Ribulose bisphosphate carboxylase, large chain
## VIT_00s0203g00140  -  Octicosapeptide/Phox/Bem1p (PB1) domain-containing protein
## VIT_16s0039g02460  -  Chromomethylase CMT2
## VIT_00s0301g00180  -  NADPH quinone oxidoreductase
## VIT_13s0074g00730  -  PID (PINOID)
## VIT_10s0003g02750  -  IMP dehydrogenase/GMP reductase
## VIT_05s0020g03980  -  Calmodulin-binding region IQD19
## VIT_14s0083g00080  -  Unknown protein
## VIT_06s0009g00210  -  Calmodulin-binding region IQ5
## VIT_06s0004g06110  -  Exo-1,3-beta-glucanase
## VIT_06s0004g05850  -  Zinc finger (C3HC4-type ring finger)
## VIT_04s0079g00260  -  Unknown protein
## VIT_00s0448g00020  -  R protein  PRF  disease resistance protein
## VIT_07s0005g03630  -  Ankyrin
## VIT_02s0025g01380  -  Endo-1,4-beta-glucanase
## VIT_11s0016g00100  -  Adapter protein SPIKE1 (SPK1)
```

```
## VIT_13s0019g02220  -  No hit
## VIT_01s0010g02810  -  Unknown protein
## VIT_02s0012g01450  -  Basic helix-loop-helix BHLH071
## VIT_18s0001g05700  -  Unknown protein
## VIT_15s0046g00240  -  Lateral organ boundaries protein 1
## VIT_19s0014g02970  -  Choline transporter
## VIT_01s0026g00330  -  NHL repeat-containing protein
## VIT_18s0001g08550  -  Squalene monooxygenase
## VIT_04s0023g03060  -  Kinesin motor protein
## VIT_02s0025g03760  -  Myosin-related
## VIT_02s0154g00590  -  Unknown
## VIT_04s0008g05830  -  Armadillo/beta-catenin repeat
## VIT_18s0001g10420  -  Remorin
## VIT_17s0000g07840  -  Unknown protein
## VIT_06s0061g01230  -  Cellulose synthase CSLA02
## VIT_01s0011g03180  -  Lysine and histidine specific transporter
## VIT_02s0025g01330  -  Polygalacturonase GH28
## VIT_16s0098g00560  -  SP1L2 (SPIRAL2)
## VIT_19s0014g04290  -  S-locus protein kinase
## VIT_11s0065g00980  -  Replication factor A 1, rfa1
## VIT_13s0067g01460  -  Avr9/Cf-9 rapidly elicited protein
## VIT_17s0000g05820  -  Ubiquitin-conjugating enzyme E2 A
## VIT_13s0101g00520  -  Unknown protein
## VIT_06s0004g01890  -  Cu2+-exporting ATPase HMA5 (heavy metal ATPase 5)
## VIT_07s0005g01140  -  Unknown protein
## VIT_00s0227g00140  -  MLO1
## VIT_03s0091g01130  -  Meprin and TRAF homology domain-containing protein
## VIT_05s0102g00590  -  No hit
## VIT_11s0016g01280  -  DNA-directed RNA polymerase III subunit C4
## VIT_17s0000g07950  -  Unknown protein
## VIT_11s0016g00110  -  Potassium channel tetramerisation domain-containing protein
## VIT_11s0103g00400  -  XH/XS domain-containing protein
## VIT_03s0088g01250  -  D-threo-aldose 1-dehydrogenase
## VIT_12s0035g01220  -  Unknown protein
## VIT_06s0004g05190  -  D-xylose-H+ symporter
## VIT_18s0001g04680  -  RPG related protein 1 RR1
## VIT_06s0004g06100  -  MYB divaricata
## VIT_08s0007g01920  -  MYB divaricata
## VIT_01s0026g00900  -  Metalloendoproteinase 1 precursor
## VIT_13s0019g01620  -  No hit
## VIT_08s0007g05420  -  Tubulin folding cofactor B
## VIT_06s0061g00730  -  Aquaporin GAMMA-TIP3/TIP1;3
## VIT_15s0048g02610  -  Ribosomal protein P1  acidic 60S
## VIT_08s0007g06820  -  Kinesin motor protein
## VIT_17s0000g09620  -  Rhodanese domain-containing protein
## VIT_10s0116g00170  -  S-locus lectin protein kinase
## VIT_00s0323g00100  -  Vacuolar processing enzyme beta
## VIT_14s0066g01280  -  Kinesin light chain
## VIT_11s0016g02430  -  Dual-specific kinase DSK1
## VIT_08s0007g07270  -  Dicarboxylate/tricarboxylate carrier (DTC)
## VIT_01s0011g05660  -  Zinc finger (Ran-binding) family
## VIT_04s0008g03780  -  Myb-related protein 3R-1 (Plant c-MYB-like protein 1) MYB3R1
## VIT_14s0066g01830  -  SAG101 (senescence-associated gene 101)
## VIT_00s0229g00140  -  Enoyl-CoA hydratase
## VIT_11s0016g04670  -  No hit
```

```
## VIT_10s0116g00910  -  CERK1 (chitin elicitor receptor kinase 1)
## VIT_13s0019g01660  -  Dehydration-induced protein (ERD15)
## VIT_14s0083g00230  -  Unknown
## VIT_14s0030g01970  -  Auxin-induced protein PCNT115
## VIT_01s0127g00870  -  Polygalacturonase JP630
## VIT_12s0028g01240  -  fanconi anemia, complementation group D2
## VIT_12s0035g01900  -  Pectinesterase family
## VIT_18s0001g15720  -  Leucine Rich Repeat receptor-like kinase
## VIT_03s0132g00100  -  ATHVA22A (Arabidopsis thaliana HVA22 homologue A)
## VIT_12s0059g01740  -  Phagocytosis and cell motility protein ELMO1
## VIT_08s0007g00030  -  Cation/hydrogen exchanger 20 (CHX20)
## VIT_12s0028g01230  -  fanconi anemia, complementation group D2
## VIT_08s0007g00440  -  Expansin  (VvEXPA11)
## VIT_06s0004g00070  -  Expansin  (VvEXPA5)
```

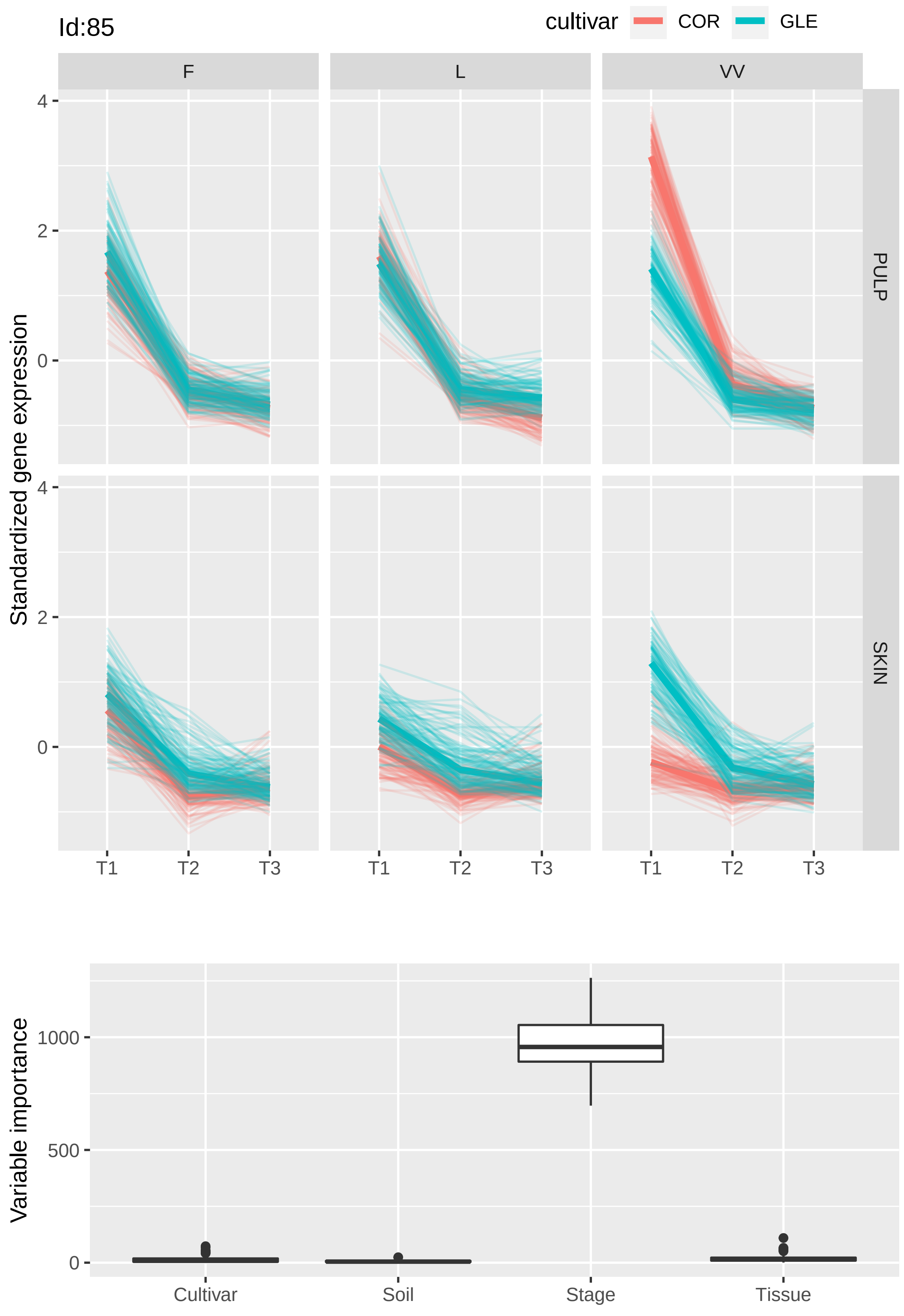

Id:85
cultivar
COR
GLE
F
L
VV
PULP
SKIN
Standardized gene expression
4
2
0
T1
T2
T3
Variable importance
1000
500
0
Cultivar
Soil
Stage
Tissue

Cluster no. 8

```
## Number of genes in the cluster: 93
## Homogeneity Index:    0.79
## Variable importance for Stage:     Rank =   8  - Median =  893.5
## Variable importance for Cultivar:  Rank =  84  - Median =  10.17
## Variable importance for Tissue:    Rank =  66  - Median =  45.22
## Variable importance for Soil:      Rank =  37  - Median =   6.07
##
## Gene ID               Gene Annotation
## VIT_12s0057g00310  -  Gamete expressed1 (GEX1)
## VIT_02s0025g03320  -  CYP86A2
## VIT_13s0064g00920  -  Zinc finger (CCCH-type) family protein
## VIT_16s0050g00570  -  Pectinacetylesterase
## VIT_19s0015g01710  -  Kinesin family member 22
## VIT_05s0077g01300  -  Aldo-keto reductase
## VIT_19s0090g01350  -  Aspartyl protease
## VIT_14s0060g00450  -  Protein-serine/threonine kinase haspin-related
## VIT_15s0046g01500  -  Translation initiation factor eIF-1A
## VIT_12s0035g01240  -  No hit
## VIT_05s0020g04390  -  Ca2+-ATPase 13 ACA13, plasma membrane
## VIT_12s0028g03620  -  Unknown protein
## VIT_18s0072g01010  -  Peptide chain release factor eRF subunit 1
## VIT_02s0025g00680  -  Tiny root hair 1
## VIT_06s0004g01210  -  PRLI-interacting factor A
## VIT_00s0169g00040  -  CTV.22
## VIT_16s0013g01940  -  Kinase-like protein TMKL1
## VIT_07s0005g02730  -  Myb Radialis
## VIT_18s0122g01210  -  Cuticular water permeability
## VIT_00s1045g00030  -  Unknown
## VIT_16s0022g01690  -  Band 7 family
## VIT_18s0001g14480  -  Thaumatin SCUTL1
## VIT_18s0001g08590  -  No hit
## VIT_12s0057g01110  -  Nudix hydrolase 2
## VIT_17s0000g06220  -  Exostosin
## VIT_00s0207g00050  -  No hit
## VIT_18s0041g00580  -  Proton-dependent oligopeptide transport (POT) family protein
## VIT_00s0337g00010  -  Salt tolerance protein 2
## VIT_17s0000g10000  -  Nitrate-responsive NOI protein
## VIT_13s0047g01130  -  Zfwd2 protein (ZFWD2)
## VIT_01s0011g03420  -  Unknown protein
## VIT_03s0063g01380  -  Unknown protein
## VIT_17s0000g02740  -  No hit
## VIT_12s0028g03860  -  Zinc finger (C3HC4-type ring finger) protein (RMA1)
## VIT_04s0023g02510  -  Glycosyl transferase family 1 protein
## VIT_19s0177g00140  -  Thiazole biosynthetic enzyme, chloroplast (ARA6)
## VIT_05s0049g01900  -  Pentatricopeptide (PPR) repeat-containing
## VIT_08s0007g02560  -  Ovate family protein 13 OFP13
## VIT_09s0002g04580  -  Unknown protein
## VIT_01s0011g01650  -  FK506-binding protein genes family (VvFKBP17-1)
## VIT_19s0015g01780  -  Zinc finger (C2H2 type) family
## VIT_02s0025g01640  -  Calcineurin B 4-1
## VIT_04s0008g03960  -  DNA polymerase theta subunit
## VIT_14s0066g01710  -  Leaf senescence protein
## VIT_17s0000g02730  -  myb domain protein 4R1
## VIT_11s0065g00160  -  Splicing factor YT521-B
```

```
## VIT_15s0048g02380  -  DCL1 (DICER 1)
## VIT_19s0090g01690  -  WNK3 (Arabidopsis WNK kinase 3)
## VIT_13s0156g00170  -  Molybdopterin synthase (CNX2)
## VIT_17s0000g09130  -  Ethylene-responsive DEAD box RNA helicase (RH30)
## VIT_18s0001g07350  -  No hit
## VIT_03s0063g01410  -  Expp1 protein precursor
## VIT_13s0067g02650  -  Nucleotidyltransferase family protein
## VIT_13s0064g01440  -  SYN3 (Sister chromatid cohesion 1 protein 3)
## VIT_11s0052g01150  -  Nicotianamine synthase
## VIT_12s0028g01060  -  No hit
## VIT_02s0012g02220  -  Xyloglucan endotransglucosylase/hydrolase 30
## ENSRNA049996267  -
## VIT_08s0007g06490  -  DNA polymerase delta, subunit B
## VIT_17s0000g05870  -  Aldose 1-epimerase protein
## VIT_13s0067g00860  -  Early flowering 4
## VIT_18s0001g07820  -  No hit
## VIT_18s0072g00220  -  Histone-lysine N-methyltransferase ASHH1
## VIT_14s0030g00500  -  Copine BON3 (BONZAI 3)
## VIT_14s0066g01920  -  Cellular repressor of E1A-stimulated genes (CREG)
## VIT_16s0098g00240  -  Ser/Thr receptor-like kinase1
## VIT_08s0007g08730  -  ABC Transporter (VvAOH1 - VvABCA1)
## VIT_17s0000g01350  -  Unknown
## VIT_08s0007g04480  -  Pectinesterase family
## VIT_09s0002g00650  -  Auxin-induced protein
## VIT_09s0002g01320  -  Germin-like protein
## VIT_03s0038g00820  -  3-oxo-5-alpha-steroid 4-dehydrogenase, C-terminal
## VIT_12s0035g01350  -  Cysteinyl-tRNA synthetase
## VIT_00s1351g00020  -  No hit
## VIT_11s0016g02220  -  Transducin family protein / WD-40 repeat
## VIT_19s0090g01790  -  Armadillo/beta-catenin repeat
## VIT_02s0025g03440  -  Gibberellin 20-oxidase
## VIT_00s0198g00170  -  AAA-type ATPase
## VIT_07s0005g05960  -  Cytokinin dehydrogenase 5 precursor
## VIT_13s0019g00700  -  Unknown protein
## VIT_06s0004g07180  -  Pentatricopeptide repeat-containing
## VIT_05s0020g04300  -  Ca2+-ATPase 13 ACA13, plasma membrane
## VIT_08s0007g08740  -  No hit
## VIT_01s0137g00260  -  No hit
## VIT_05s0020g03070  -  Cyclin-T1-4
## VIT_09s0002g00760  -  Unknown protein
## VIT_06s0004g03090  -  Syntaxin of plants SYP7
## VIT_05s0094g00170  -  Protein transport protein Sec61 subunit beta
## VIT_01s0011g04960  -  Unknown protein
## VIT_02s0241g00100  -  Unknown protein
## VIT_00s1959g00010  -  UDP-N-acetylglucosamine pyrophosphorylase
## VIT_04s0008g03070  -  No hit
## VIT_14s0066g02540  -  Acid phosphatase
```

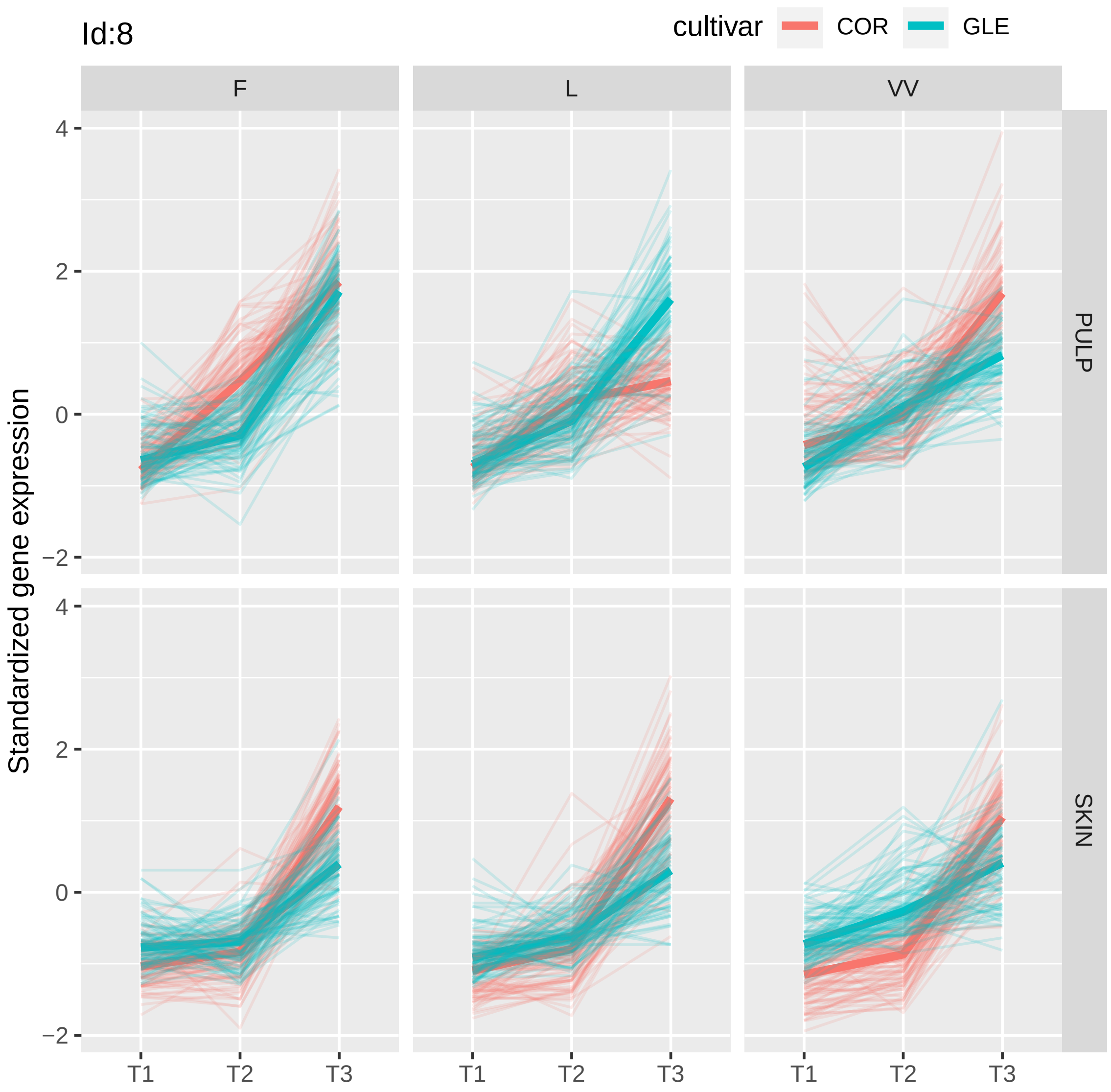
Id:8
cultivar
COR
GLE
F
L
VV
PULP
SKIN
Standardized gene expression
4
2
0
−2
T1
T2
T3

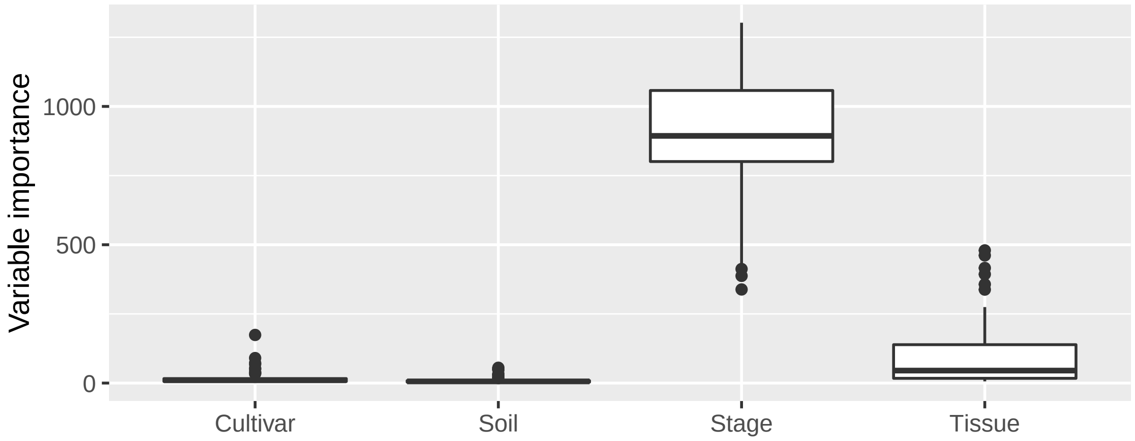
Variable importance
1000
500
0
Cultivar
Soil
Stage
Tissue

Cluster no. 9

```
## Number of genes in the cluster: 108
## Homogeneity Index:    0.84
## Variable importance for Stage:     Rank =   9  - Median =  877.2
## Variable importance for Cultivar:  Rank =  70  - Median =     15
## Variable importance for Tissue:    Rank =  86  - Median =  16.73
## Variable importance for Soil:      Rank =  53  - Median =   5.52
##
## Gene ID                Gene Annotation
## VIT_11s0016g03370  -  Exostosin-like
## VIT_17s0000g00840  -  Unknown protein
## VIT_09s0002g06010  -  Phospholipase/carboxylesterase
## VIT_16s0050g00380  -  Tubulin beta-1
## VIT_19s0014g01910  -  Unknown protein
## VIT_08s0040g02650  -  Unknown protein
## VIT_12s0134g00560  -  Unknown protein
## VIT_06s0061g01250  -  Unknown protein
## VIT_14s0068g01870  -  Nodulin
## VIT_09s0002g02060  -  Unknown protein
## VIT_00s0194g00010  -  Phenylalanine-tRNA ligase
## VIT_14s0068g01620  -  Pollen proteins Ole e I
## VIT_11s0052g00120  -  Purple acid phosphatase 28- ATPAP28/PAP28
## VIT_09s0002g04020  -  Aquaporin DELTA-TIP
## VIT_18s0001g10640  -  No hit
## VIT_11s0016g00190  -  Transcription factor
## VIT_10s0042g01070  -  Zinc finger (C3HC4-type ring finger)
## VIT_05s0020g03640  -  Unknown protein
## VIT_19s0085g00010  -  Auxin-regulated protein
## VIT_13s0019g03450  -  Unknown protein
## VIT_08s0058g00540  -  IMK2 (inflorescence meristem receptor-like kinase 2)
## VIT_03s0038g00940  -  Auxin-responsive
## VIT_13s0019g03590  -  Catalytic
## VIT_09s0002g06110  -  Zinc finger (Ran-binding)
## VIT_09s0002g00540  -  Lipase GDSL 1
## VIT_04s0008g07370  -  Glutamine dumper  2
## VIT_05s0094g01430  -  Unknown protein
## VIT_07s0005g04220  -  Receptor-like protein kinase
## VIT_17s0000g01760  -  Beta-glucosidase 24
## VIT_06s0061g01040  -  Argonaute protein
## VIT_12s0121g00230  -  Beta-D-xylosidase
## VIT_13s0019g04500  -  Transketolase
## VIT_11s0052g00160  -  ATP binding , related
## VIT_10s0042g01150  -  ARGONAUTE 2 (AGO2)
## VIT_06s0009g01740  -  Geranylgeranyl reductase
## VIT_17s0000g08750  -  ATPase-like domain-containing
## VIT_02s0012g01870  -  No hit
## VIT_14s0108g00440  -  Tubulin alpha
## VIT_13s0019g02540  -  Subtilisin protease C1
## VIT_08s0007g04560  -  PLATZ transcription factor
## VIT_18s0001g10620  -  No hit
## VIT_07s0005g01840  -  Patatin
## VIT_00s0204g00130  -  Unknown
## VIT_14s0066g00120  -  Unknown protein
## VIT_18s0001g10610  -  No hit
## VIT_18s0001g11310  -  OBF binding protein 1
```

```
## VIT_02s0025g04180  -  Unknown protein
## VIT_05s0020g03650  -  Unknown protein
## VIT_19s0090g00630  -  Tetraacyldisaccharide 4'-kinase
## VIT_04s0008g05430  -  RDR6 (RNA-dependent RNA polymerase 6)
## VIT_00s0187g00050  -  No hit
## VIT_18s0001g12400  -  Unknown protein
## VIT_19s0015g01550  -  Phenylalanine-tRNA ligase
## VIT_00s0499g00020  -  Unknown protein
## VIT_14s0030g01300  -  Unknown
## VIT_05s0102g00660  -  No hit
## VIT_00s0802g00020  -  Unknown protein
## VIT_16s0050g02350  -  Regulator of nonsense transcripts 1
## VIT_18s0041g00370  -  Double strand break repair protein (XRCC4)
## VIT_06s0009g01090  -  Heat shock  HSP20 family protein
## VIT_07s0005g06160  -  No hit
## VIT_05s0020g00480  -  Mitochondrial substrate carrier family protein
## VIT_01s0146g00090  -  11-beta-hydroxysteroid dehydrogenase
## VIT_04s0079g00230  -  Sucrose synthase
## VIT_01s0011g02620  -  No hit
## VIT_06s0004g06670  -  Phosphoinositide-specific phospholipase C
## VIT_00s0187g00090  -  No hit
## VIT_17s0000g07420  -  EDS1 (Enhanced disease susceptibility 1)
## VIT_09s0002g05980  -  CYP734A7 castasterone 26-hydroxylase
## VIT_17s0053g00700  -  Sucrose synthase 2
## VIT_02s0025g01850  -  Cellulose synthase CSLG3
## VIT_01s0026g01910  -  myb domain protein 88
## VIT_10s0003g01900  -  RKF1 (receptor-like kinase in flowers 1)
## VIT_00s0373g00030  -  HSL2 (HAESA-like 2)
## VIT_00s0658g00020  -  Protein kinase Xa21
## VIT_02s0025g02020  -  Abl interactor 3 (ABIL3)
## VIT_06s0004g00220  -  Protein kinase APK1B
## VIT_19s0014g02830  -  Whitefly-induced gp91-phox
## VIT_09s0002g06880  -  Beta-1,3-glucanase
## VIT_15s0048g00770  -  Kinesin motor KHC
## VIT_14s0128g00190  -  Heavy-metal-associated domain-containing protein
## VIT_10s0003g05210  -  Wall-associated kinase 1 (WAK1)
## VIT_03s0038g02520  -  Aquaporin PIP3
## VIT_01s0026g01490  -  Proton-dependent oligopeptide transport (POT) family protein
## VIT_13s0073g00150  -  Heat shock protein-related
## VIT_18s0089g01010  -  Nitrate transporter NTL1
## VIT_02s0025g04120  -  Calmodulin binding protein
## VIT_13s0019g00650  -  No hit
## VIT_14s0006g02260  -  MATE efflux family protein
## VIT_02s0154g00580  -  Unknown
## VIT_18s0001g10040  -  LRX1 (leucine-rich repeat/extensin 1)
## VIT_02s0025g03190  -  2-oxoglutarate-dependent dioxygenase
## VIT_02s0025g01630  -  SOS3 (salt overly sensitive 3)
## VIT_18s0001g03570  -  Thaumatin ATLP-1
## VIT_14s0066g00100  -  Unknown protein
## VIT_03s0017g02240  -  Glucan endo-1,3-beta-glucosidase precursor
## VIT_06s0004g03420  -  Dof zinc finger protein DOF5.3
## VIT_00s0186g00090  -  Zinc finger (C3HC4-type ring finger)
## VIT_08s0105g00250  -  Aluminum activated malate transporter 1
## VIT_12s0057g00470  -  C2 domain containing protein
## VIT_06s0004g03770  -  Embryo-specific protein
```

```
## VIT_18s0001g04890  -  Low affinity sulphate transporter
## VIT_17s0000g07440  -  Replication protein A 70 kDa DNA-binding subunit
## VIT_06s0004g05870  -  Tubulin beta-3 chain
## VIT_08s0007g03780  -  Unknown protein
## VIT_18s0001g03540  -  Auxin transporter protein 4
## VIT_18s0041g01610  -  No hit
## VIT_10s0042g01180  -  ARGONAUTE 2 (AGO2)
```

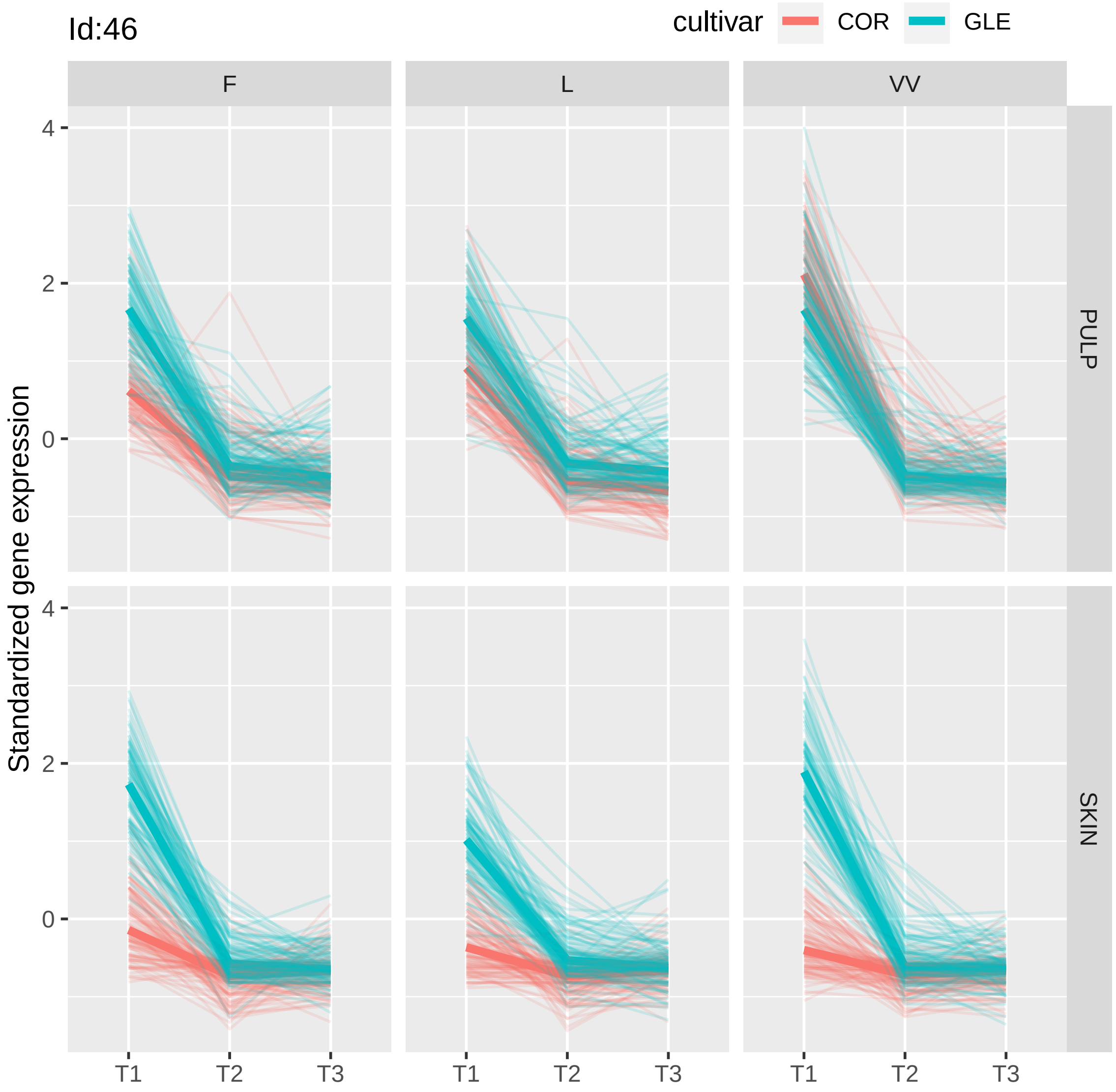

Id:46
cultivar
COR
GLE
F
L
VV
PULP
SKIN
Standardized gene expression
4
2
0
T1
T2
T3

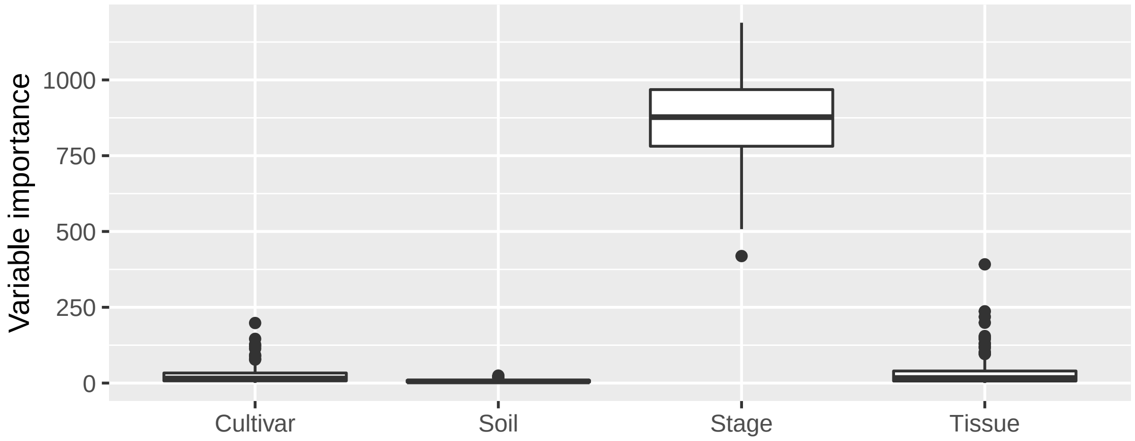

Variable importance
1000
750
500
250
0
Cultivar
Soil
Stage
Tissue

Cluster no. 10

```
## Number of genes in the cluster: 69
## Homogeneity Index:    0.87
## Variable importance for Stage:     Rank =  10  - Median =  854.9
## Variable importance for Cultivar:  Rank =  80  - Median =  11.21
## Variable importance for Tissue:    Rank =  53  - Median =  108.9
## Variable importance for Soil:      Rank =  20  - Median =   7.84
##
## Gene ID                Gene Annotation
## VIT_01s0011g01280  -  Zinc transporter ZIP5
## VIT_19s0015g01000  -  Zinc finger (C3HC4-type ring finger)
## VIT_04s0008g05010  -  Unknown protein
## VIT_18s0001g00470  -  Monocopper oxidase SKS5 (SKU5 Similar 5)
## VIT_00s0904g00010  -  Photosystem II oxygen-evolving enhancer protein PSBQ
## VIT_14s0108g01130  -  EXS family protein / ERD1/XPR1/SYG1
## VIT_12s0059g01240  -  Nitrate transporter (NTP3)
## VIT_12s0028g03120  -  No hit
## VIT_06s0004g04860  -  Expansin  (VvEXPA6)
## VIT_04s0023g02730  -  Nodulin MtN21 family
## VIT_01s0026g01290  -  Glycosyl transferase family 14 protein
## VIT_05s0094g01590  -  Kelch repeat-containing F-box protein
## VIT_18s0001g06370  -  L-ascorbate peroxidase, chloroplast
## VIT_07s0031g00680  -  Red chlorophyll catabolite reductase (accelerated cell death 2)
## VIT_13s0064g00160  -  Unknown protein
## VIT_04s0079g00680  -  phytoene synthase (PSY) (VvPSY1)
## VIT_06s0009g01110  -  Cation exchanger (CAX7)
## VIT_10s0042g00450  -  CC-NBS-LRR class
## VIT_16s0039g00970  -  Glutathione S-transferase 8 GSTU8
## VIT_03s0038g02170  -  Thaumatin
## VIT_09s0002g04130  -  Proton-dependent oligopeptide transport (POT) family protein
## VIT_11s0016g03380  -  Exostosin-like
## VIT_01s0127g00220  -  PMR5 (powdery mildew resistant 5)
## VIT_14s0066g02530  -  Fumarate reductase/succinate dehydrogenase flavoprotein
## VIT_19s0177g00340  -  Unknown protein
## VIT_18s0001g09510  -  CYP81B2v1
## VIT_00s0271g00100  -  Unknown
## VIT_05s0020g00930  -  Soluble diacylglycerol acyltransferase
## VIT_12s0059g02480  -  CYCP2;1 (cyclin p2;1)
## VIT_00s0800g00010  -  Alpha-1,4-glycosyltransferase
## VIT_05s0020g04600  -  Metallothionein
## VIT_18s0072g00970  -  DegP protease,
## VIT_00s0762g00040  -  S-locus lectin protein kinase
## VIT_08s0040g00980  -  Tubulin beta-1 chain
## VIT_04s0044g00530  -  PAP/fibrillin family
## VIT_18s0001g06900  -  Unknown protein
## VIT_14s0128g00300  -  Ctp
## VIT_16s0039g01820  -  Unknown protein
## VIT_16s0050g02240  -  Unknown protein
## VIT_17s0000g08070  -  Aldehyde Dehydrogenase (VvALDH2B4)
## VIT_01s0244g00170  -  Cytokinin-repressed protein CR9
## VIT_12s0028g02370  -  Methyltransferase type 11
## VIT_14s0128g00320  -  Amidase
## VIT_07s0005g02590  -  Polygalacturonase GH28
## VIT_05s0020g03440  -  Photosystem II 11 kDa protein PSB27
## VIT_17s0000g07790  -  N-hydroxythioamide S-beta-glucosyltransferase
```

```
## VIT_00s2525g00010  -  Unknown
## VIT_00s0229g00160  -  formyltetrahydrofolate deformylase
## VIT_03s0063g00110  -  Cyclin delta-3
## VIT_16s0098g00820  -  Peroxidase 3
## VIT_05s0049g00410  -  1-aminocyclopropane-1-carboxylate oxidase
## VIT_04s0008g04240  -  ferredoxin
## VIT_03s0063g02600  -  Leucine-rich repeat transmembrane protein kinase
## VIT_00s0179g00320  -  Unknown protein
## VIT_17s0000g04490  -  Extracellular Ca2+ sensing receptor
## VIT_13s0019g00420  -  F-box family protein
## VIT_07s0104g00420  -  Endo-1,3;1,4-beta-D-glucanase precursor
## VIT_18s0001g08310  -  Inositol 1,3,4-trisphosphate 5/6-kinase
## VIT_17s0000g07060  -  UDP-glucosyltransferase HRA25
## VIT_18s0001g14450  -  Ferredoxin:nadp+ Oxidoreductase PETH
## VIT_06s0061g01060  -  Magnesium transporter CorA 10
## VIT_16s0098g01540  -  Thylakoid lumenal 17.4 kDa protein, chloroplast
## VIT_16s0098g00850  -  Caffeic acid O-methyltransferase (COMT1)
## VIT_13s0064g00670  -  No hit
## VIT_15s0021g00320  -  No hit
## VIT_18s0001g01140  -  Peroxidase 64
## VIT_02s0025g02000  -  Ribosomal protein S6
## VIT_05s0020g02310  -  Pyruvate,orthophosphate dikinase
## VIT_10s0003g03310  -  EMB2756 (embryo defective 2756)
```

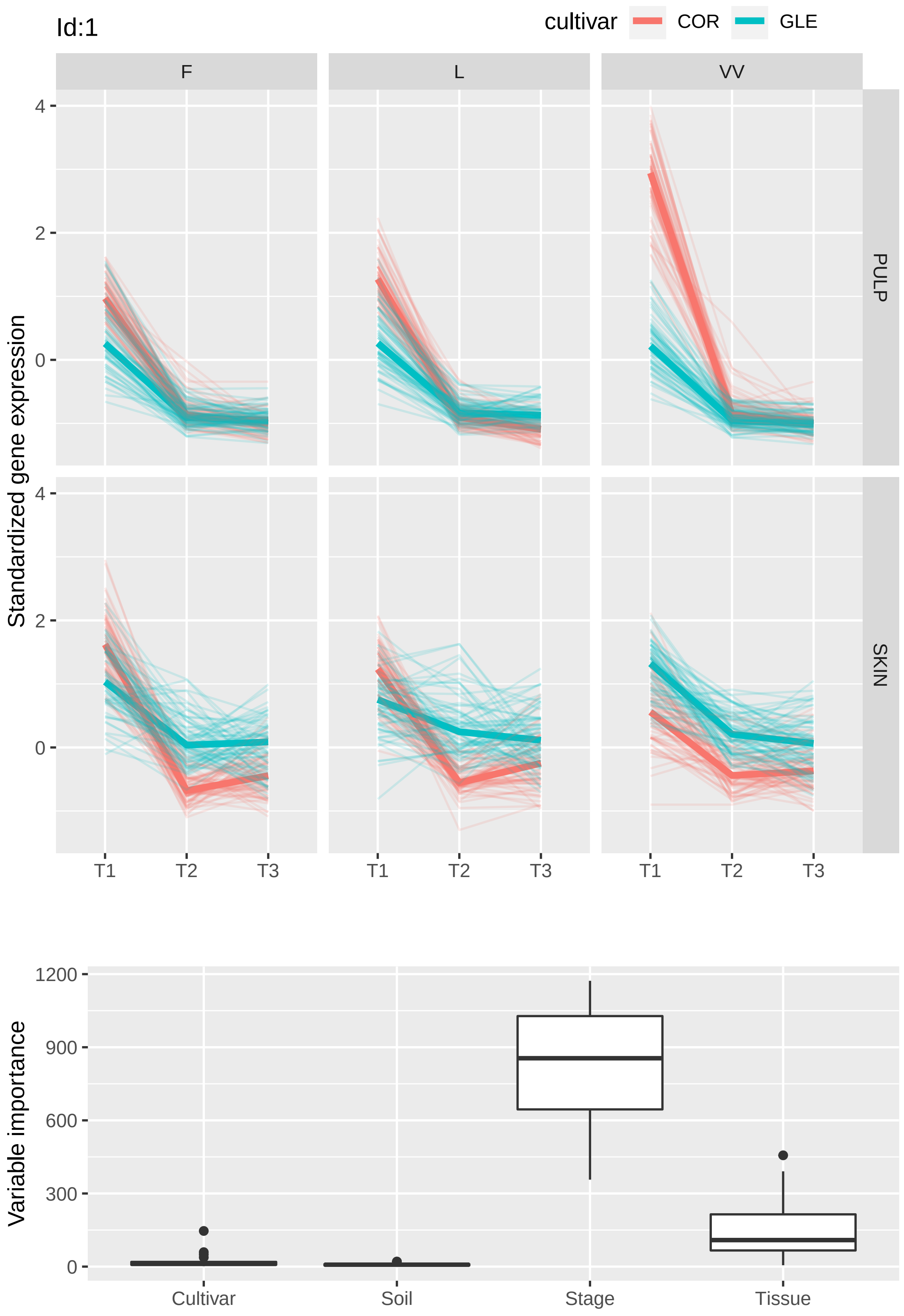

Id:1
cultivar
COR
GLE
F
L
VV
PULP
SKIN
Standardized gene expression
4
2
0
T1
T2
T3
Variable importance
1200
900
600
300
0
Cultivar
Soil
Stage
Tissue

Cluster no. 11

```
## Number of genes in the cluster: 173
## Homogeneity Index:     0.9
## Variable importance for Stage:     Rank =  11  - Median =  846.6
## Variable importance for Cultivar:  Rank =  79  - Median =  11.55
## Variable importance for Tissue:    Rank =  98  - Median =   9.27
## Variable importance for Soil:      Rank =  60  - Median =   5.15
##
## Gene ID                Gene Annotation
## VIT_04s0008g02750  -  Basic Leucine Zipper Transcription Factor (VvbZIP09)
## VIT_01s0011g06050  -  RNA recognition motif (RRM)-containing protein
## VIT_13s0074g00240  -  Uridylate kinase
## VIT_04s0008g01910  -  ferredoxin-related
## VIT_12s0028g00040  -  Unknown protein
## VIT_12s0034g01090  -  Lung seven transmembrane receptor
## VIT_08s0007g03630  -  Calmodulin binding protein
## VIT_17s0000g05460  -  Nodulin
## VIT_15s0046g01190  -  Hydroquinone glucosyltransferase
## VIT_07s0104g01620  -  Zinc finger (C2H2 type) MAGPIE
## VIT_06s0009g02020  -  Tubulin beta-4 chain
## VIT_07s0104g01250  -  flavin-containing monooxygenase
## VIT_14s0066g01820  -  SAG101 (senescence-associated gene 101)
## VIT_00s0338g00030  -  Cellular retinaldehyde-binding/triple function, C-terminal
## VIT_03s0038g03660  -  Armadillo/beta-catenin repeat protein / U-box domain-containing
## VIT_02s0025g03250  -  Protein kinase
## VIT_04s0069g00850  -  Beta-galactosidase
## VIT_08s0007g07880  -  Polygalacturonase GH28
## VIT_12s0028g01520  -  S-receptor kinase
## VIT_16s0098g01700  -  Epoxide hydrolase
## VIT_09s0018g01670  -  Aspartic Protease (VvAP26)
## VIT_18s0001g12860  -  Unknown protein
## VIT_07s0141g00360  -  Lipase class 3
## VIT_14s0128g00020  -  Na+/H+ exchanger
## VIT_07s0141g00600  -  Unknown protein
## VIT_08s0007g02470  -  Aspartic Protease (VvAP24)
## VIT_12s0028g03210  -  Tyrosine aminotransferase
## VIT_15s0046g00430  -  Harpin-induced 1
## VIT_06s0004g02370  -  Cinnamyl alcohol dehydrogenase
## VIT_04s0008g05480  -  BRI1-KD interacting protein 118
## VIT_09s0002g00370  -  Unknown protein
## VIT_08s0007g08280  -  Remorin
## VIT_11s0016g04760  -  Unknown protein
## VIT_04s0008g01970  -  ER (ERECTA)
## VIT_08s0058g00490  -  Unknown protein
## VIT_05s0077g02330  -  Transducin protein
## VIT_17s0000g01800  -  Unknown protein
## VIT_16s0098g00150  -  Receptor serine/threonine kinase PR5K
## VIT_02s0154g00080  -  Multi-copper oxidase (SKU5)
## VIT_00s0361g00050  -  Protein-serine/threonine kinase
## VIT_18s0122g00980  -  Glucan endo-1,3-beta-glucosidase 7 precursor
## VIT_19s0015g01670  -  Phenylalanine-tRNA ligase
## VIT_07s0031g00130  -  Kinesin protein FRA1 (fragile fiber 1)
## VIT_19s0090g01230  -  Microtubule associated protein (MAP65-1a)
## VIT_07s0031g02550  -  ABC Transporter (VvWBC17 - VvABCG17)
## VIT_12s0057g00890  -  Glycosyl hydrolase family 17 protein
```

```
## VIT_18s0001g13200  -  Cytokinin dehydrogenase 5 precursor
## VIT_18s0001g06610  -  Plastid-targeted protein 2
## VIT_04s0008g03760  -  Unknown protein
## VIT_11s0052g01560  -  MATE efflux family protein
## VIT_16s0100g00480  -  Dihydrodipicolinate reductase
## VIT_09s0002g08540  -  Serine-rich protein
## VIT_02s0025g03390  -  Aquaporin TMP-C
## VIT_00s0392g00050  -  Protein-serine/threonine kinase
## VIT_07s0129g00770  -  CYP81D2
## VIT_07s0005g00010  -  Glutathione S-transferase 8 GSTF8
## VIT_04s0008g00700  -  No hit
## VIT_06s0004g02520  -  Telomere repeat binding factor like TRFL6
## VIT_12s0059g00530  -  EDM2
## VIT_08s0058g00740  -  Iron-responsive transporter
## VIT_07s0031g00880  -  Nodulation protein
## VIT_08s0007g06640  -  Growth-regulating factor related
## VIT_06s0061g01490  -  ABC Transporter (VvPDR20 - VvABCG50)
## VIT_18s0001g04790  -  Aspartic Protease (VvAP42)
## VIT_13s0064g01240  -  Ribosomal protein L31 (RPL31C) 60S
## VIT_06s0004g06560  -  ABC Transporter (VvPDR18 - VvABCG48)
## VIT_18s0001g09530  -  Zinc finger (C3HC4-type ring finger)
## VIT_14s0066g02040  -  Chaperone BCS1 mitochondrial
## VIT_18s0122g00620  -  Cinnamoyl-CoA reductase
## VIT_17s0000g06970  -  Diacylglycerol kinase 1
## VIT_05s0077g01070  -  Universal stress protein (USP) family protein
## VIT_14s0081g00720  -  basic helix-loop-helix (bHLH) family
## VIT_06s0004g00440  -  No hit
## VIT_01s0011g00610  -  Unknown protein
## VIT_18s0001g05180  -  Beta-D-xylosidase
## VIT_18s0001g05220  -  WD-40 repeat
## VIT_06s0004g02830  -  Unknown protein
## VIT_01s0146g00520  -  Sterile alpha motif (SAM) domain-containing
## VIT_14s0128g00330  -  Mevalonate kinase (MK)
## ENSRNA049469401  -
## VIT_18s0122g00610  -  3-hydroxy-3-methylglutaryl-coenzyme A reductase 3
## VIT_17s0000g01460  -  Protein kinase AKIN gamma
## VIT_07s0005g01750  -  No hit
## VIT_11s0016g04860  -  Ethylene-responsive element-binding protein EREBP-4
## VIT_08s0040g01860  -  Unknown protein
## VIT_12s0134g00170  -  No hit
## VIT_16s0039g02550  -  Seed specific protein Bn15D1B
## VIT_07s0005g00060  -  No hit
## VIT_07s0031g00790  -  Steroid nuclear receptor, ligand-binding
## VIT_08s0007g00150  -  Zinc finger (C3HC4-type ring finger)
## VIT_08s0058g01010  -  Unknown
## VIT_11s0052g00620  -  AHA2 (Arabidopsis H(+)-ATPase 2)
## VIT_08s0007g00980  -  Integral membrane family protein UPF0497
## VIT_06s0009g02500  -  Plastocyanin domain-containing protein
## VIT_08s0058g01090  -  CBL-interacting protein kinase 14 (CIPK14)
## VIT_14s0083g00240  -  Stress protein
## VIT_14s0006g01970  -  Auxin efflux carrier family
## VIT_15s0046g01380  -  Unknown
## VIT_09s0002g06990  -  Phosphatidic acid phosphatase / PAP2
## VIT_11s0052g01250  -  Xyloglucan endotransglucosylase/hydrolase 23
## VIT_16s0050g01620  -  ABC Transporter (VvWBC23 - VvABCG23)
```

```
## VIT_19s0014g04310  -  S-locus protein kinase
## VIT_11s0052g00890  -  Unknown protein
## VIT_06s0004g04580  -  Protein kinase family protein
## VIT_05s0020g03380  -  WNK1 (with no lysine (K) 1)
## VIT_00s1034g00010  -  E3 ubiquitin-protein ligase CHIP
## VIT_09s0002g00130  -  H(+)-ATPase (VvPH5)
## VIT_07s0104g00540  -  CYCP1;1; CYCP1;1
## VIT_14s0068g01490  -  NAC domain-containing protein (VvNAC73)
## VIT_13s0067g02280  -  basic helix-loop-helix (bHLH) family
## VIT_09s0002g07120  -  Adenine phosphoribosyltransferase
## VIT_00s0357g00130  -  Annexin ANN6
## VIT_11s0118g00800  -  KNAT2 (knotted1-like homeobox gene 3)
## VIT_02s0025g00300  -  No hit
## VIT_07s0104g01150  -  RNA-binding protein
## VIT_02s0033g00300  -  myb family
## VIT_03s0180g00090  -  Receptor kinase
## VIT_06s0004g00740  -  Acyl-CoA synthetases (Acyl-activating enzyme 18)
## VIT_04s0008g00870  -  Phosphoethanolamine/phosphocholine phosphatase
## VIT_14s0066g01850  -  Peroxidase
## VIT_00s1667g00010  -  LEM3 (ligand-effect modulator 3) family protein
## VIT_00s0391g00010  -  Pentatricopeptide (PPR) repeat-containing
## VIT_09s0002g02640  -  Ribosomal protein S30 (RPS30C) 40S
## VIT_13s0019g01010  -  ABA2 (ABA deficient 2)
## VIT_05s0102g01170  -  Sterol-4-alpha-carboxylate 3-dehydrogenase, decarboxylating
## VIT_15s0046g00360  -  Aspartic Protease (VvAP37)
## VIT_03s0132g00090  -  ATHVA22A (Arabidopsis thaliana HVA22 homologue A)
## VIT_18s0086g00250  -  Potassium transporter (KUP6)
## VIT_09s0002g07890  -  R protein disease resistance protein
## VIT_16s0050g01610  -  UDP-glycosyltransferase 88A4
## VIT_03s0063g00910  -  No hit
## VIT_08s0007g00160  -  Glycosyltransferase family 14
## VIT_12s0055g00290  -  Anthocyanidin 3-O-glucosyltransferase
## VIT_18s0001g06460  -  Queuine tRNA-ribosyltransferase
## VIT_11s0052g01700  -  CBL-interacting protein kinase 21 (CIPK21)
## VIT_13s0073g00170  -  Exocyst subunit EXO70 A1
## VIT_12s0057g01550  -  Unknown protein
## VIT_17s0000g03840  -  Agenet domain-containing protein
## VIT_14s0066g01650  -  Nodulin MtN21 family
## VIT_18s0001g07390  -  No hit
## VIT_13s0084g00430  -  Unknown
## VIT_07s0005g00050  -  Heavy-metal-associated domain-containing protein
## VIT_18s0001g10450  -  ABA-responsive element-binding protein 2 (AREB2), Basic Leucine Zipper
## VIT_15s0046g03700  -  3-beta hydroxysteroid dehydrogenase
## VIT_03s0038g02680  -  Zeta-carotene desaturase
## VIT_02s0025g01750  -  Cellulose synthase CSLG3
## VIT_12s0028g02650  -  RAB GTPase RABA1F
## VIT_13s0019g02530  -  Subtilisin protease C1
## VIT_14s0036g01450  -  Unknown protein
## VIT_08s0007g03340  -  Ribosomal protein L10A (RPL10aA) 60S
## VIT_03s0017g01210  -  Phosphate-induced protein 1
## VIT_15s0046g01130  -  Myb Triptychon
## VIT_05s0020g02180  -  Unknown protein
## VIT_06s0004g02880  -  Receptor-like kinase
## VIT_00s0324g00080  -  UDP-glucoronosyl and UDP-glucosyl transferase
## VIT_08s0040g03050  -  RabGAP/TBC domain-containing protein
```

```
## VIT_05s0049g00390  -  E8 protein
## VIT_14s0060g01190  -  Nicotianamine synthase
## VIT_12s0059g00540  -  EDM2
## VIT_07s0031g02910  -  Receptor-like kinase
## ENSRNA049995927  -
## VIT_04s0044g01850  -  Auxin efflux carrier
## VIT_14s0108g01180  -  Unknown protein
## VIT_18s0001g05530  -  Annexin 1 (ANN1)
## VIT_06s0061g00790  -  Pheophorbide a oxygenase
## VIT_05s0020g01830  -  UPF0497 family
## VIT_15s0048g00170  -  H(+)-ATPase 4 AHA4
## VIT_02s0025g03230  -  Fringe protein
## VIT_00s0304g00020  -  UNE2 (unfertilized embryo sac 2)
## VIT_03s0038g03980  -  Unknown protein
## VIT_13s0019g04600  -  ABC Transporter (VvWBC15 - VvABCG15)
## VIT_02s0025g03450  -  basic helix-loop-helix (VvJAF13)
## VIT_03s0017g00540  -  Subtilase
```

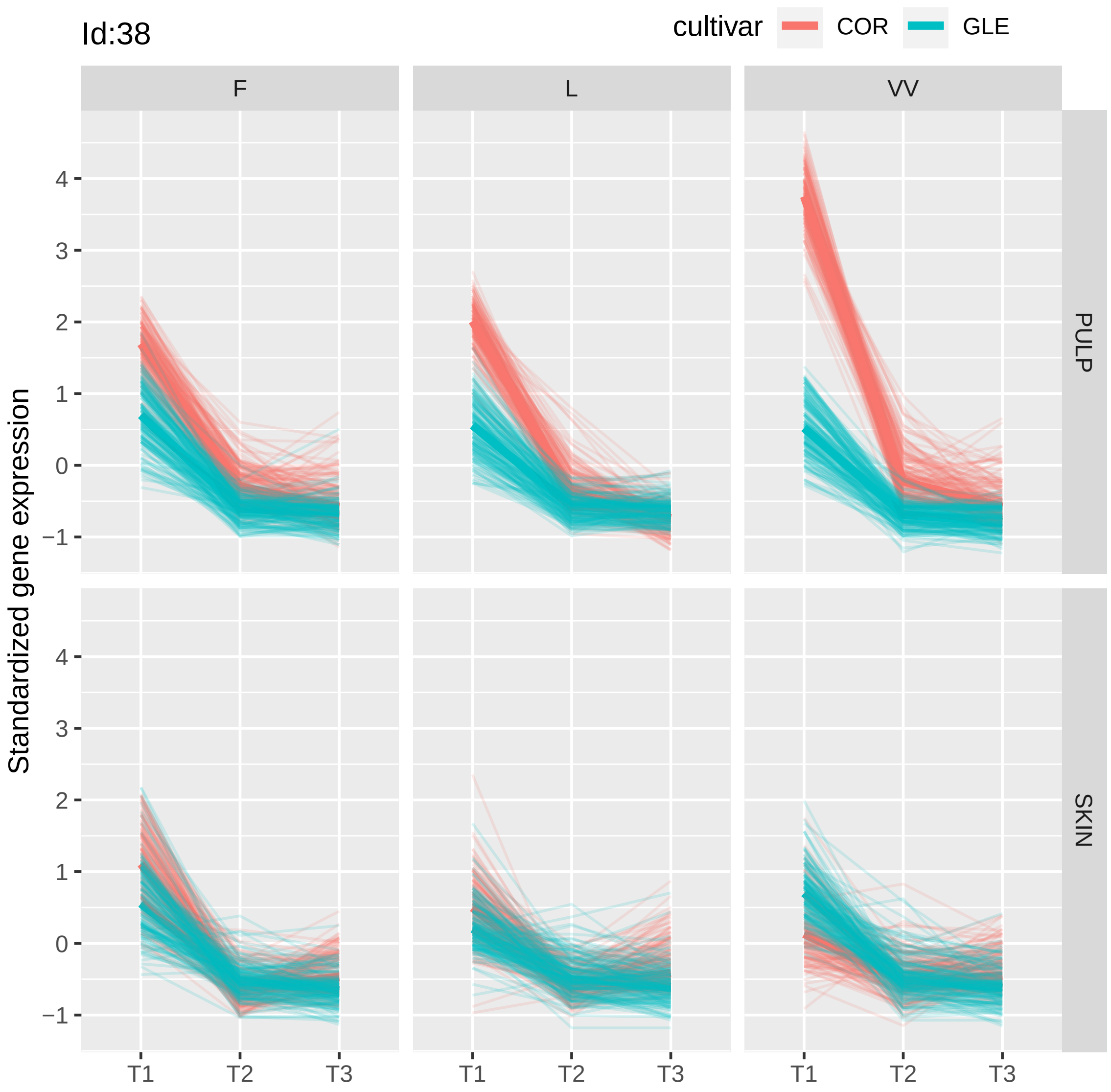
Id:38
cultivar
COR
GLE
F
L
VV
PULP
SKIN
Standardized gene expression
4
3
2
1
0
−1
T1
T2
T3

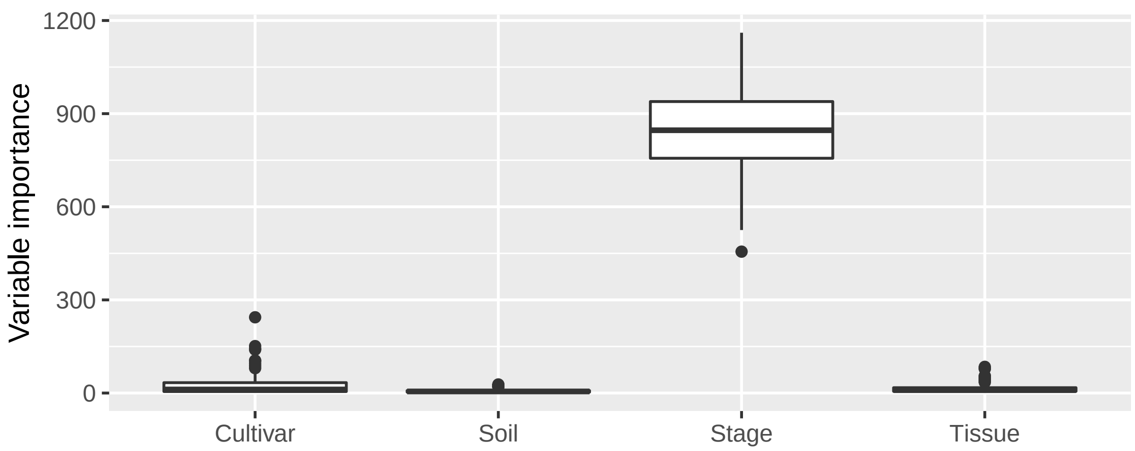
Variable importance
1200
900
600
300
0
Cultivar
Soil
Stage
Tissue

Cluster no. 12

```
## Number of genes in the cluster: 71
## Homogeneity Index:    0.79
## Variable importance for Stage:     Rank =  12  - Median =  836.1
## Variable importance for Cultivar:  Rank =  77  - Median =  12.22
## Variable importance for Tissue:    Rank =  91  - Median =  14.36
## Variable importance for Soil:      Rank =  17  - Median =   8.31
##
## Gene ID                Gene Annotation
## VIT_17s0000g04750  -  UDP-glycosyltransferase 89B2
## VIT_06s0004g04650  -  Metallothionein
## VIT_06s0004g00910  -  Protease inhibitor/seed storage/lipid transfer protein (LTP)
## VIT_01s0011g01050  -  Vacuolar sorting receptor 6
## VIT_01s0010g00730  -  Pentatricopeptide (PPR) repeat-containing protein
## VIT_14s0108g00970  -  Pentatricopeptide (PPR) repeat
## VIT_09s0002g00580  -  Lipase GDSL 3
## VIT_12s0057g00990  -  Beta 1,2 N-acetylglucosaminyltransferase
## VIT_10s0003g05450  -  Reticuline oxidase precursor
## VIT_04s0008g05620  -  Adenine phosphoribosyltransferase
## VIT_00s0225g00010  -  Unknown protein
## VIT_01s0011g01940  -  Unknown protein
## VIT_14s0060g00270  -  Pentatricopeptide (PPR) repeat-containing
## VIT_03s0063g02310  -  Acidic leucine-rich nuclear phosphoprotein 32 member B
## VIT_02s0025g02170  -  Trehalase precursor
## VIT_07s0031g02280  -  MYB divaricata
## VIT_02s0234g00130  -  Ethylene responsive element binding factor 1
## VIT_04s0008g03660  -  Embryonic flower 1
## VIT_00s0587g00040  -  Unknown protein
## VIT_15s0046g01050  -  Abscisic acid receptor PYL9  RCAR1
## VIT_02s0025g03600  -  Phospholipid hydroperoxide glutathione peroxidase
## VIT_18s0001g15530  -  Pentatricopeptide (PPR) repeat-containing
## VIT_16s0050g02440  -  HAT dimerisation domain-containing protein
## VIT_04s0023g02110  -  Unknown protein
## VIT_14s0006g00170  -  Pentatricopeptide (PPR) repeat-containing protein
## VIT_17s0000g03460  -  Unknown protein
## VIT_05s0020g01260  -  Pentatricopeptide (PPR) repeat-containing protein
## VIT_18s0001g07210  -  Unknown
## VIT_08s0007g03070  -  Pentatricopeptide (PPR) repeat-containing protein
## VIT_17s0000g06770  -  Pentatricopeptide (PPR) repeat-containing protein
## VIT_11s0016g01470  -  Lecithine cholesterol acyltransferase
## VIT_03s0063g01460  -  Cytochrome b6f complex subunit (petM)
## VIT_18s0001g07200  -  SUPPRESSOR OF ACAULIS 51
## VIT_04s0008g04360  -  No hit
## VIT_16s0050g00970  -  Unknown
## VIT_18s0001g13790  -  Cytochrome P450, family 83, subfamily B, polypeptide 1
## VIT_04s0044g00110  -  High-mobility group B 2
## VIT_08s0007g07310  -  No hit
## VIT_13s0067g00850  -  No hit
## VIT_01s0026g02310  -  SUR2
## VIT_08s0040g02940  -  Pentatricopeptide (PPR) repeat-containing protein
## VIT_18s0001g00750  -  Zinc finger (DNL type)
## VIT_12s0059g01120  -  CAF protein
## VIT_02s0109g00250  -  4-coumarate-CoA ligase
## VIT_18s0001g06120  -  Cis-zeatin O-beta-D-glucosyltransferase
## VIT_13s0019g01490  -  C2 domain-containing protein
```

```
## VIT_15s0048g02780  -  Unknown protein
## VIT_17s0000g06480  -  Pentatricopeptide (PPR) repeat-containing protein
## VIT_18s0001g12030  -  Uracil-DNA glycosylase
## VIT_09s0002g04370  -  CRR2 (chlororespiratory reduction 2)
## VIT_18s0001g04630  -  Aspartate aminotransferase
## VIT_08s0040g03390  -  Unknown
## VIT_00s2393g00010  -  GATA transcription factor 28
## VIT_11s0118g00510  -  Ubiquitin-associated (UBA)/TS-N domain-containing
## VIT_03s0091g01200  -  Sinapoylglucose:malate sinapoyltransferase (SNG1)
## VIT_15s0021g02410  -  Malate dehydrogenase, cytosolic
## VIT_09s0054g01050  -  No hit
## VIT_08s0007g03200  -  RNA-binding region RNP-1
## VIT_00s0287g00030  -  No hit
## VIT_12s0059g01950  -  Pentatricopeptide (PPR) repeat-containing protein
## VIT_06s0009g03560  -  Pentatricopeptide (PPR) repeat-containing protein
## ENSRNA049462811  -
## VIT_08s0007g03140  -  Rac GTPase activating protein 1
## VIT_01s0010g01680  -  Glutaredoxin
## VIT_09s0002g05150  -  IAA19
## VIT_03s0038g01310  -  Auxin responsive SAUR protein
## VIT_19s0014g05070  -  Kinesin family member C1
## VIT_08s0007g03860  -  S-receptor kinase
## VIT_19s0027g01340  -  Clathrin heavy chain
## VIT_12s0034g02120  -  No hit
## VIT_04s0044g01540  -  UDP-glucuronic acid:anthocyanin glucuronosyltransferase
```

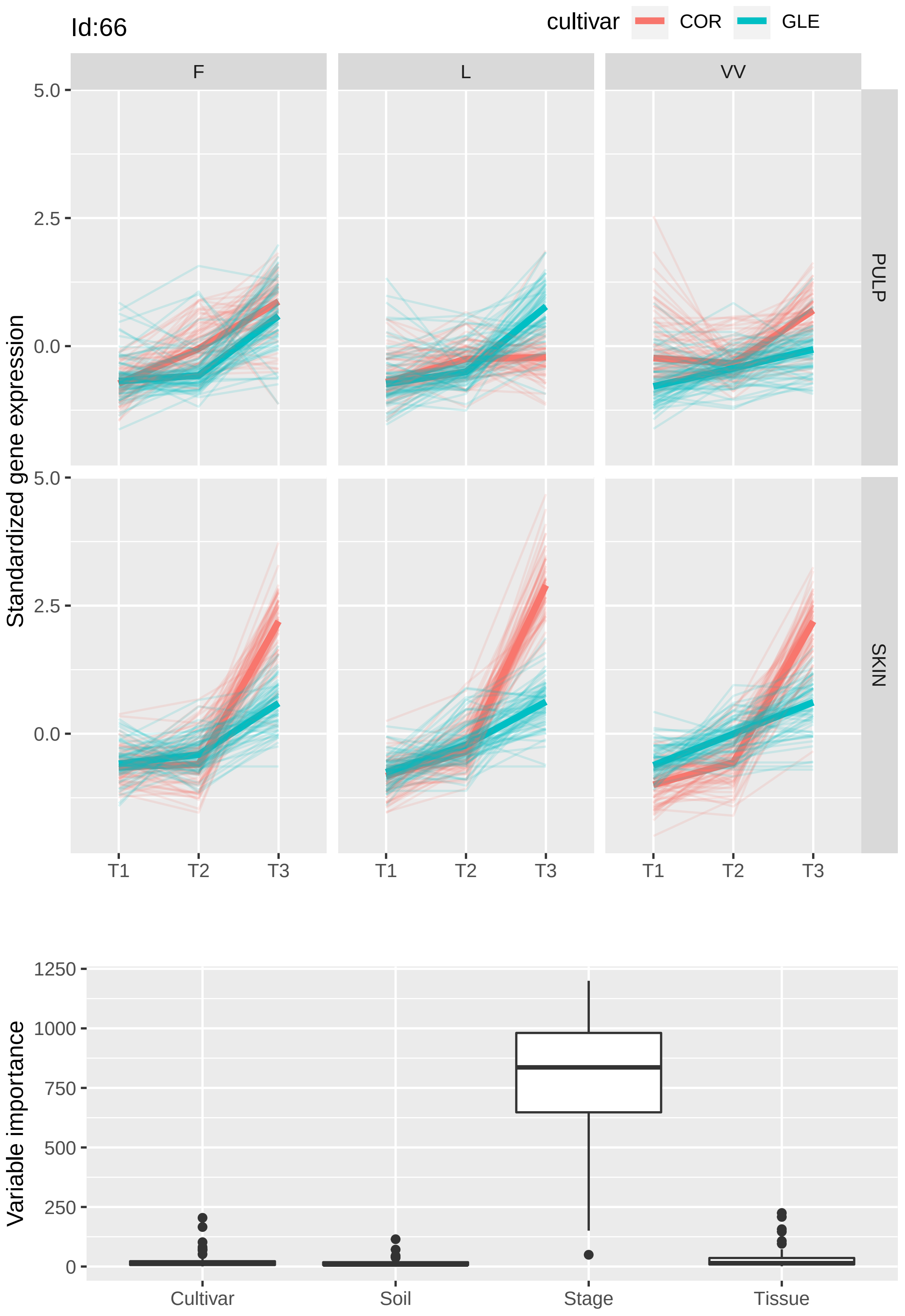

Id:66
cultivar
COR
GLE
F
L
VV
PULP
SKIN
Standardized gene expression
5.0
2.5
0.0
T1
T2
T3
Variable importance
1250
1000
750
500
250
0
Cultivar
Soil
Stage
Tissue

Cluster no. 13

```
## Number of genes in the cluster: 66
## Homogeneity Index:     0.9
## Variable importance for Stage:     Rank =  13  - Median =  823.9
## Variable importance for Cultivar:  Rank =  65  - Median =  20.79
## Variable importance for Tissue:    Rank =  45  - Median =  147.8
## Variable importance for Soil:      Rank =  55  - Median =   5.43
##
## Gene ID               Gene Annotation
## VIT_04s0023g03860  -  Rac-like GTP-binding protein ARAC1 precursor (GTPase protein ROP3)
## VIT_06s0004g05680  -  Glutathione S-transferase 25 GSTU7
## VIT_19s0014g04270  -  S-locus protein kinase
## VIT_18s0122g00680  -  Oxygen-evolving complex PsbP
## VIT_18s0001g08090  -  IAA9
## VIT_13s0064g01430  -  Pigment defective 322
## VIT_06s0061g00440  -  Heavy-metal-associated domain-containing protein
## VIT_04s0008g01950  -  YGGT
## VIT_18s0001g09140  -  Esterase
## VIT_04s0008g04380  -  Vernalization insensitive 3
## VIT_08s0040g03010  -  Pigment defective 149
## VIT_12s0134g00550  -  Equilibrative nucleoside transporter (ENT3)
## VIT_15s0048g02520  -  Zinc transporter (ZAT)
## VIT_06s0004g05340  -  Tropinone reductase
## VIT_05s0094g00150  -  Membrane-associated 30 kDa protein, chloroplast precursor (M30)
## VIT_13s0019g05380  -  Unknown protein
## VIT_08s0040g03230  -  Unknown protein
## VIT_18s0001g05710  -  Hydrolase, alpha/beta fold
## VIT_06s0004g00610  -  Accelerated cell death 1 ACD1
## VIT_19s0135g00230  -  CYP72A1
## VIT_18s0001g02440  -  Aldehyde Dehydrogenase (VvALDH3H1)
## VIT_16s0013g00220  -  Metacaspase AtMCP1b
## VIT_05s0094g00820  -  Unknown protein
## VIT_02s0012g00980  -  ferredoxin-6, chloroplast precursor
## VIT_14s0006g01410  -  fructokinase-2
## VIT_12s0035g01330  -  RPS2 (resistant to p. syringae 2)
## VIT_02s0025g04710  -  Unknown protein
## VIT_18s0122g01140  -  Wuschel-related homeobox 13
## VIT_03s0017g01330  -  1,4-dihydroxy-2-naphthoate octaprenyltransferase
## VIT_00s0759g00010  -  Porphobilinogen deaminase, chloroplast precursor
## VIT_05s0077g00560  -  Serine carboxypeptidase S10
## VIT_18s0001g09390  -  Protein phosphatase 2C
## VIT_19s0014g00710  -  RKF1 (receptor-like kinase in flowers 1)
## VIT_05s0124g00530  -  Ankyrin protein kinase
## VIT_14s0030g00640  -  DnaJ homolog, subfamily B, member 12
## VIT_19s0090g01780  -  Zinc finger (CCCH-type) family protein
## VIT_06s0004g04700  -  Outer envelope protein 16
## VIT_06s0004g07120  -  Gamma interferon responsive lysosomal thiol reductase family protein
## VIT_01s0150g00630  -  Exostosin-like
## VIT_09s0002g08090  -  Oxidoreductase, 2OG-Fe(II) oxygenase
## VIT_18s0001g05020  -  Senescence-associated protein
## VIT_16s0098g01180  -  Aldo/keto reductase
## VIT_06s0009g01670  -  forkhead-associated domain-containing protein
## VIT_07s0129g00250  -  Phototropic-responsive NPH3
## VIT_19s0014g00700  -  Leucine-rich repeat protein kinase
## VIT_08s0040g00160  -  Unknown protein
```

```
## VIT_04s0008g01960  -  Copper-transporting ATPase PAA2
## VIT_06s0009g01660  -  RPS2 (resistant to p. syringae 2)
## VIT_00s0207g00110  -  Antigen receptor
## VIT_14s0068g01990  -  Unknown protein
## VIT_12s0034g01180  -  PAP/fibrillin family
## VIT_00s0975g00010  -  Cellulose synthase CSLE1
## VIT_19s0014g03200  -  COP1-interacting protein 7
## VIT_12s0034g01210  -  PAP/fibrillin family
## VIT_10s0071g00150  -  R protein disease resistance protein
## VIT_07s0031g00590  -  Triacylglycerol lipase
## VIT_08s0007g04340  -  FK506-binding protein genes family (VvFKBP16-4)
## VIT_11s0149g00040  -  Adenylate kinase
## VIT_09s0054g01770  -  Oxidoreductase/ transcriptional repressor
## VIT_01s0010g03890  -  Unknown
## VIT_11s0016g00730  -  LHCA6 (Photosystem I light harvesting complex gene 6)
## VIT_02s0241g00120  -  Receptor protein kinase
## VIT_13s0067g01660  -  Sister chromatid cohesion 1 protein 1 DIF1/SYN1
## VIT_03s0038g00670  -  fructose-bisphosphate aldolase, chloroplast precursor
## VIT_08s0056g01480  -  Cation exchanger, CAX7
## VIT_06s0004g05180  -  Ribulose bisphosphate carboxylase/oxygenase activase, chloroplast
```

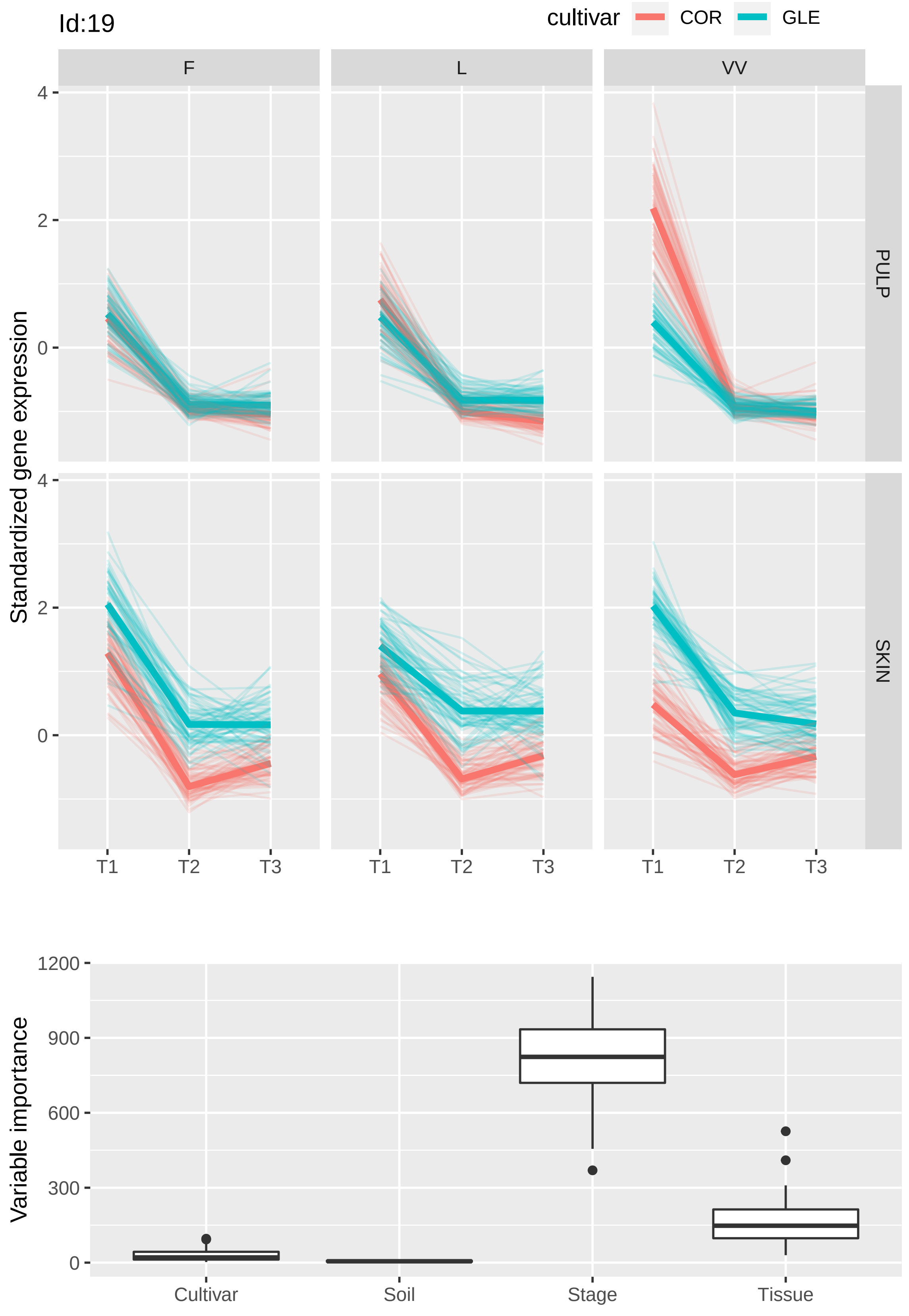
Id:19
cultivar
COR
GLE
F
L
VV
PULP
SKIN
Standardized gene expression
4
2
0
T1
T2
T3
Variable importance
1200
900
600
300
0
Cultivar
Soil
Stage
Tissue

Cluster no. 14

```
## Number of genes in the cluster: 104
## Homogeneity Index:     0.9
## Variable importance for Stage:     Rank =  14  - Median =  815.9
## Variable importance for Cultivar:  Rank =  93  - Median =    7.2
## Variable importance for Tissue:    Rank =  42  - Median =  170.3
## Variable importance for Soil:      Rank =  87  - Median =   3.55
##
## Gene ID               Gene Annotation
## VIT_10s0116g01780  -  Cationic peroxidase
## VIT_02s0012g01220  -  Unknown protein
## VIT_11s0052g00100  -  basic helix-loop-helix (bHLH) family
## VIT_17s0000g00270  -  GT2-like trihelix DNA-binding protein
## VIT_12s0035g00970  -  Evolutionarily conserved C-terminal region 11 ECT11
## VIT_02s0012g01380  -  No hit
## VIT_04s0023g01660  -  Scarecrow-like transcription factor 7 (SCL7)
## VIT_18s0001g06940  -  Purine permease 1 (PUP1)
## VIT_07s0031g00620  -  zeaxanthin epoxidase (ZEP; ABA1) (VvZEP1)
## VIT_04s0008g05240  -  Brittle 1 protein
## VIT_02s0236g00130  -  SNF1-related protein kinase SRK2F (SnRK-5)
## VIT_11s0016g01400  -  PSBX (photosystem II subunit X)
## VIT_14s0066g01640  -  Nodulin MtN21 family
## VIT_03s0038g03560  -  CAO (CHAOS); chromatin binding
## VIT_04s0008g02640  -  PAP/fibrillin family
## VIT_05s0020g01840  -  3,8-divinyl protochlorophyllide a 8-vinyl reductase
## VIT_07s0104g01530  -  Transcription termination factor mitochondrial mTERF
## VIT_01s0146g00360  -  Constans-like 14
## VIT_06s0004g08190  -  ERF/AP2 Gene Family (VvERF055)
## VIT_10s0071g01100  -  Alpha-galactosidase precursor
## VIT_16s0098g01280  -  Rhodanese domain-containing protein
## VIT_01s0010g03620  -  LHCA2 (Photosystem I light harvesting complex gene 2)
## VIT_07s0005g02480  -  Myb domain protein 61
## VIT_13s0019g00240  -  Glycosyltransferase family 14 Beta-1-3-galactosyl-O-glycosyl-glycoprot
## VIT_06s0004g05110  -  Unknown
## VIT_07s0031g00650  -  Defective chloroplasts and leaves protein / DCL protein
## VIT_08s0058g00280  -  Lectin protein kinase
## VIT_00s0125g00280  -  Photosystem I reaction center subunit III (PSAF)
## VIT_11s0078g00310  -  Isoamylase-type starch-debranching enzyme 1
## VIT_07s0151g00930  -  Unknown protein
## VIT_12s0028g02420  -  1-aminocyclopropane-1-carboxylate oxidase homolog 1
## VIT_01s0026g02200  -  TCP family transcription factor TCP15
## VIT_08s0058g00930  -  Alanine--glyoxylate aminotransferase 2 3, mitochondrial
## VIT_17s0000g03800  -  Unknown protein
## VIT_19s0014g00790  -  RKF1 (receptor-like kinase in flowers 1)
## VIT_11s0118g00650  -  Unknown
## VIT_19s0090g00700  -  No hit
## VIT_08s0040g00850  -  Thylakoid lumenal 20 kDa protein
## VIT_09s0002g06770  -  Unknown protein
## VIT_00s0772g00010  -  Protein kinase
## VIT_06s0061g00770  -  Ribosomal protein L9, chloroplast (CL9) 50S
## VIT_17s0000g05650  -  Unknown protein
## VIT_04s0008g01900  -  Unknown protein
## VIT_11s0052g00420  -  Allyl alcohol dehydrogenase
## VIT_18s0001g00760  -  Plastocyanin, chloroplast precursor
## VIT_01s0011g05290  -  Inorganic carbon transport protein
```

```
## VIT_19s0027g00200  -  Photosystem II 10 kDa polypeptide PSBR
## VIT_09s0002g03460  -  Taurine dioxygenase
## VIT_14s0081g00060  -  Photosystem II reaction centre W (PsbW)
## VIT_08s0007g03040  -  E8 protein
## VIT_00s0975g00020  -  Cellulose synthase CSLE1
## VIT_05s0077g00110  -  Unknown protein
## VIT_10s0003g02680  -  Phytochrome E
## VIT_17s0000g05250  -  Unknown protein
## VIT_17s0000g09060  -  Unknown
## VIT_08s0007g02190  -  LHCB4 chlorophyll A-B binding protein CP29
## VIT_08s0007g06450  -  Omega-6 fatty acid desaturase, endoplasmic reticulum (FAD2)
## VIT_12s0028g02000  -  Triacylglycerol lipase
## VIT_07s0141g00570  -  Photosystem II reaction centre W (PsbW)
## VIT_07s0005g04920  -  Photosystem I reaction center subunit IV B (PSAE B)
## VIT_00s0174g00010  -  No hit
## VIT_01s0010g00940  -  Unknown protein
## VIT_01s0011g00930  -  FK506-binding protein genes family (VvFKBP13)
## VIT_07s0095g00290  -  Serine/threonine kinase BRLK
## VIT_05s0020g01930  -  TUBBY like protein 3 TLP3
## VIT_19s0014g01930  -  Unknown protein
## VIT_00s0753g00020  -  Histone H2A.4 HTA12
## VIT_08s0007g03110  -  Transposon protein, Mutator sub-class
## VIT_09s0002g04330  -  Ribosomal protein S17 30S
## VIT_01s0026g01930  -  Unknown protein
## VIT_04s0008g01730  -  PSBX (photosystem II subunit X)
## VIT_04s0023g01150  -  Unknown protein
## VIT_12s0057g00630  -  LHCB2.1 (Photosystem II light harvesting complex gene 2.1)
## VIT_03s0038g02790  -  Ribosomal protein L2, Chloroplast
## VIT_19s0015g01760  -  Photosystem I reaction center subunit V (PSAG)
## VIT_08s0007g00710  -  Unknown
## VIT_15s0046g02910  -  Ribosomal protein L21, chloroplast / CL21 (RPL21) 50S
## VIT_07s0005g01470  -  Unknown protein
## VIT_12s0028g01080  -  Photosystem II oxygen-evolving complex precursor, 23kda PSBP
## VIT_09s0018g01870  -  D-3-phosphoglycerate dehydrogenase, chloroplast precursor (3-PGDH)
## VIT_06s0004g07070  -  Protein kinase
## VIT_18s0089g00400  -  Carboxyesterase18 CXE18
## VIT_09s0002g04320  -  Photosystem I reaction center subunit VI PSAH
## VIT_17s0000g00110  -  Rubredoxin
## VIT_18s0001g06720  -  Rieske [2Fe-2S] domain
## VIT_04s0008g07340  -  Constans-like 4
## VIT_15s0046g01140  -  WRKY Transcription Factor (VvWRKY47)
## VIT_06s0009g01380  -  Ethylene-insensitive 3 (EIN3)
## VIT_00s0353g00050  -  Serine/threonine kinase BRLK
## VIT_07s0129g00950  -  Protein kinase
## VIT_11s0016g00560  -  Peroxiredoxin bcp
## VIT_02s0012g01940  -  Myb KAN2 (KANADI 2)
## VIT_06s0004g04080  -  GTP cyclohydrolase II; 3 4-dihydroxy-2-butanone-4-phoshate synthase
## VIT_16s0098g00690  -  Major Facilitator Superfamily
## VIT_01s0010g01900  -  Pentatricopeptide (PPR) repeat-containing protein
## VIT_01s0010g01170  -  YGGT
## VIT_03s0038g02780  -  Ribosomal protein L23
## VIT_10s0092g00240  -  Alpha-glucosidase 1 (AGLU1)
## VIT_14s0036g00310  -  RPS2 (resistant to p. syringae 2)
## VIT_13s0084g00850  -  NADPH:quinone oxidoreductase
## VIT_18s0041g00490  -  Proton-dependent oligopeptide transport (POT) family protein
```

```
## VIT_16s0022g01390  -  flavonoid 3-monooxygenase
## VIT_07s0151g00520  -  Homocysteine S-methyltransferase 3
## VIT_01s0011g03360  -  Unknown
```

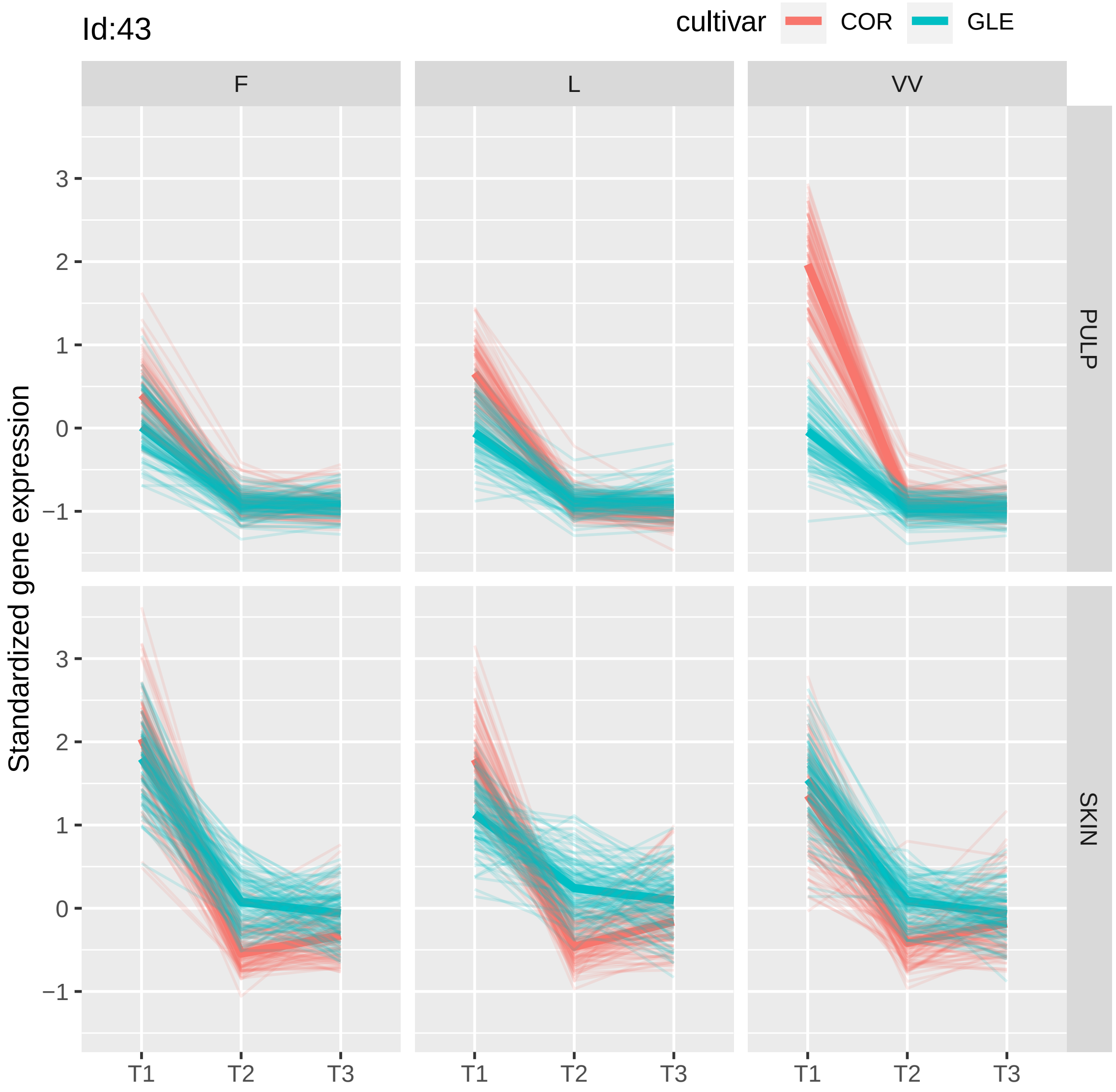

Id:43
cultivar
COR
GLE
F
L
VV
PULP
SKIN
Standardized gene expression
3
2
1
0
−1
T1
T2
T3

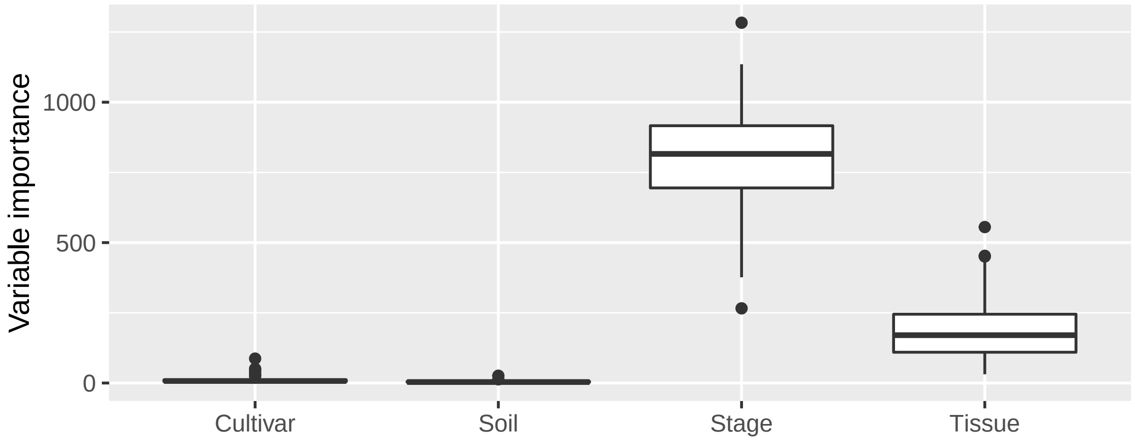

Variable importance
1000
500
0
Cultivar
Soil
Stage
Tissue

Cluster no. 15

```
## Number of genes in the cluster: 161
## Homogeneity Index:    0.83
## Variable importance for Stage:     Rank =  15  - Median =  800.9
## Variable importance for Cultivar:  Rank =  66  - Median =  18.87
## Variable importance for Tissue:    Rank =  82  - Median =  23.86
## Variable importance for Soil:      Rank =  26  - Median =   6.96
##
## Gene ID                Gene Annotation
## VIT_19s0014g01800  -  Homogentisate 1,2-dioxygenase
## VIT_14s0171g00480  -  F-box domain containing protein
## VIT_18s0041g00730  -  Peptide chain release factor eRF subunit 1
## VIT_14s0068g00810  -  Helicase
## VIT_15s0021g02720  -  Zinc finger (C2H2 type) family
## VIT_18s0041g00720  -  No hit
## VIT_05s0102g00250  -  Unknown protein
## VIT_19s0014g02370  -  3-hydroxybutyryl-CoA dehydrogenase
## VIT_04s0008g03770  -  Aspartate aminotransferase P1
## VIT_13s0067g03660  -  Unknown protein
## VIT_00s0194g00070  -  Constans-like 10
## VIT_15s0046g00160  -  WD-40 repeat
## VIT_01s0011g02760  -  No hit
## VIT_06s0080g01270  -  HAT dimerisation domain-containing protein
## VIT_19s0015g01900  -  Dimethylmenaquinone methyltransferase
## VIT_18s0001g12240  -  Peptide chain release factor eRF subunit 1
## VIT_14s0171g00470  -  No hit
## VIT_00s0270g00010  -  No hit
## VIT_06s0004g08100  -  VP1/ABI3 family regulatory protein
## VIT_11s0016g01210  -  Pentatricopeptide (PPR) repeat-containing
## VIT_03s0038g03720  -  fertility restorer
## VIT_04s0008g06970  -  DnaJ-like zinc-finger protein
## VIT_08s0007g07450  -  Splicing factor YT521-B
## VIT_14s0066g01950  -  Metalloendoproteinase 1 precursor
## VIT_18s0001g12260  -  Incomplete root hair elongation
## VIT_08s0040g01440  -  TRNA (guanine-N(7)-)-methyltransferase
## VIT_10s0092g00770  -  Cytochrome c biogenesis orf256
## VIT_14s0083g00200  -  DDT domain-containing protein
## VIT_17s0000g02710  -  myb domain protein 4R1
## VIT_01s0011g02770  -  Zinc finger CCHC domain-containing protein 8
## VIT_14s0030g01600  -  DNA-binding storekeeper protein
## VIT_14s0128g00700  -  EMB2261 (embryo defective 2261)
## VIT_01s0010g02380  -  F-box domain containing protein
## VIT_09s0054g01570  -  Strubbelig receptor family 5
## VIT_14s0108g00980  -  Cycling DOF factor 1
## VIT_01s0011g05970  -  Heat stress transcription factor A-8
## VIT_19s0090g00370  -  Unknown protein
## VIT_18s0122g00110  -  Unknown protein
## VIT_16s0013g00550  -  No hit
## VIT_18s0001g13940  -  Pentatricopeptide (PPR) repeat-containing
## VIT_08s0040g00310  -  Zinc finger (C3HC4-type ring finger)
## VIT_08s0007g03080  -  Pentatricopeptide repeat
## VIT_02s0033g01170  -  Replication protein RPA 70kDa subunit
## VIT_00s0270g00090  -  Pentatricopeptide repeat-containing protein
## VIT_05s0102g00380  -  Unknown
## VIT_00s0525g00030  -  Pentatricopeptide (PPR) repeat-containing
```

```
## VIT_04s0023g03120  -  Histone H3
## VIT_03s0038g00590  -  Unknown protein
## VIT_07s0005g02530  -  Unknown protein
## VIT_06s0004g07930  -  Nucleotidyltransferase family
## VIT_02s0154g00610  -  Pex19 protein
## VIT_03s0038g03830  -  DNA-binding bromodomain-containing protein
## VIT_08s0040g02880  -  Unknown protein
## VIT_14s0060g00420  -  Pyruvate dehydrogenase kinase
## VIT_00s0181g00040  -  TIR1 (transport inhibitor response 1)
## VIT_10s0092g00210  -  Transcription factor jumonji (jmj) protein
## VIT_00s0415g00040  -  Glycine-rich protein
## VIT_07s0141g00810  -  Zinc finger, SWIM-type
## VIT_10s0003g01470  -  Auxin-independent growth promoter (axi 1)
## VIT_19s0014g02260  -  Unknown protein
## VIT_07s0005g06050  -  Homeobox-1
## VIT_18s0001g02960  -  Nucleotidyltransferase family protein, putative, expressed
## VIT_19s0090g01380  -  No hit
## VIT_10s0003g02950  -  No hit
## VIT_14s0083g00720  -  Pentatricopeptide (PPR) repeat-containing
## VIT_09s0002g05250  -  Aminotransferase class I and II
## VIT_08s0032g00840  -  Sucrose-phosphatase.
## VIT_18s0001g09660  -  CYP81D2
## VIT_10s0003g00150  -  NSL1 (necrotic spotted lesions 1)
## VIT_11s0118g00320  -  Transposase
## VIT_04s0023g02700  -  Thioredoxin H
## VIT_11s0206g00060  -  ARR18
## VIT_17s0053g00360  -  Pentatricopeptide (PPR) repeat
## VIT_11s0016g04940  -  Zinc finger (CCCH-type) family protein
## VIT_18s0075g00710  -  Regulator of nonsense transcripts 3 UPF3
## VIT_11s0016g03810  -  Unknown protein
## VIT_16s0039g00260  -  Pectate lyase
## VIT_18s0122g00840  -  EMB2261 (embryo defective 2261)
## VIT_12s0059g01430  -  Argonaute (AGO1)
## VIT_16s0013g02150  -  No hit
## VIT_01s0146g00330  -  Hydroxyproline-rich glycoprotein family protein
## VIT_13s0074g00470  -  OTU cysteine protease
## VIT_13s0067g03760  -  U4/U6 small nuclear ribonucleoprotein PRP3
## VIT_04s0008g03040  -  RNA recognition motif (RRM)-containing
## VIT_00s0181g00030  -  No hit
## VIT_14s0006g00780  -  No hit
## ENSRNA049465035  -
## VIT_11s0052g00070  -  DNA ligase IV
## VIT_10s0003g00970  -  Pentatricopeptide (PPR) repeat-containing protein
## VIT_12s0035g01090  -  Regulator of nonsense transcripts 3 UPF3
## VIT_18s0072g00800  -  Histone H3
## VIT_07s0104g00280  -  No hit
## VIT_08s0007g03980  -  Pentatricopeptide (PPR) repeat-containing
## VIT_05s0020g02850  -  Peroxiredoxin-5
## VIT_05s0051g00730  -  Zinc finger (C3HC4-type ring finger)
## VIT_05s0020g02280  -  Unknown protein
## VIT_01s0010g01670  -  Zinc knuckle
## VIT_12s0142g00050  -  PUMILIO 7 (APUM7)
## VIT_06s0080g00360  -  Basic Leucine Zipper Transcription Factor (VvbZIP20)
## VIT_07s0191g00210  -  Helicase domain-containing protein
## VIT_02s0025g05130  -  High mobility group HMG-I and HMG-Y, DNA-binding
```

```
## VIT_02s0012g00610  -  Allyl alcohol dehydrogenase
## VIT_15s0048g01770  -  Unknown
## VIT_04s0008g02460  -  H+-transporting ATPase, Plasma membrane
## VIT_12s0121g00030  -  PHP domain-containing protein
## VIT_08s0040g01840  -  No hit
## VIT_10s0116g00630  -  Unknown protein
## VIT_00s0317g00150  -  Pectate lyase
## VIT_05s0077g01320  -  Pentatricopeptide (PPR) repeat-containing protein
## VIT_07s0191g00200  -  RNA helicase
## VIT_00s0204g00020  -  Transcription factor
## VIT_10s0116g01790  -  Unknown
## VIT_01s0026g00230  -  U4/U6 small nuclear ribonucleoprotein PRP31
## VIT_08s0040g02440  -  PWWP domain-containing protein
## VIT_18s0001g09300  -  Protein Mpv17
## VIT_12s0035g00240  -  DAG protein, chloroplast precursor
## VIT_14s0068g01240  -  Pentatricopeptide (PPR) repeat-containing
## VIT_18s0001g03680  -  Protein kinase
## VIT_16s0039g00160  -  Metallophosphoesterase
## VIT_12s0055g00400  -  Unknown
## VIT_10s0003g01390  -  Cupin, RmlC-type
## VIT_12s0034g01030  -  R protein MLA10
## VIT_01s0011g01030  -  H(+)-ATPase 6 AHA6
## VIT_19s0014g00990  -  DNA polymerase delta subunit 3
## VIT_11s0052g00850  -  Replicon protein A (ATRPA2/ROR1/RPA2)
## VIT_05s0124g00540  -  EMB1674
## VIT_12s0059g01100  -  PRLI-interacting factor K
## VIT_06s0004g03370  -  Unknown protein
## VIT_01s0011g03390  -  Unknown protein
## VIT_19s0090g00030  -  Serine/threonine Protein kinase BNK1
## VIT_11s0016g03500  -  Unknown protein
## VIT_16s0039g02430  -  No hit
## VIT_05s0051g00140  -  RRS1 (resistant to ralstonia solanacearum 1)
## VIT_16s0098g00220  -  Membrane located receptor
## VIT_08s0007g07890  -  Pentatricopeptide (PPR) repeat-containing
## VIT_06s0061g00280  -  2-phosphoglycerate kinase
## VIT_00s0479g00010  -  Protein kinase 6
## VIT_15s0046g02500  -  Unknown protein
## VIT_06s0061g01020  -  Unknown protein
## VIT_03s0063g02630  -  Unknown protein
## VIT_16s0098g01570  -  Acyl-CoA binding protein 2 (ACBP2)
## VIT_13s0074g00100  -  3-phosphoinositide-dependent protein kinase
## VIT_08s0007g08840  -  Glycosyl transferaseHGA1
## VIT_05s0077g00690  -  Galactokinase (GAL1)
## VIT_04s0008g01280  -  No hit
## VIT_00s2262g00010  -  Low expression of osmotically responsive genes 1
## VIT_08s0056g01500  -  Unknown protein
## VIT_02s0012g02090  -  Unknown protein
## VIT_04s0008g06760  -  Chlororespiratory reduction 4 CRR4
## VIT_08s0032g01220  -  Calcium Dependent Protein Kinase (VvCPK9)
## VIT_16s0039g02440  -  Unknown protein
## VIT_00s0341g00040  -  Exostosin family protein
## ENSRNA049996635  -
## VIT_13s0074g00480  -  Phospholipid-transporting ATPase
## VIT_19s0015g01830  -  Unknown protein
## VIT_06s0004g06330  -  No hit
```

```
## VIT_00s0988g00010  -  CF9
## VIT_08s0040g01810  -  Auxin response factor 10
## VIT_16s0050g00790  -  Hydroxyproline-rich glycoprotein
## VIT_13s0019g03610  -  RNA recognition motif (RRM)-containing protein
## VIT_11s0052g01280  -  Xyloglucan endotransglucosylase/hydrolase 23
```

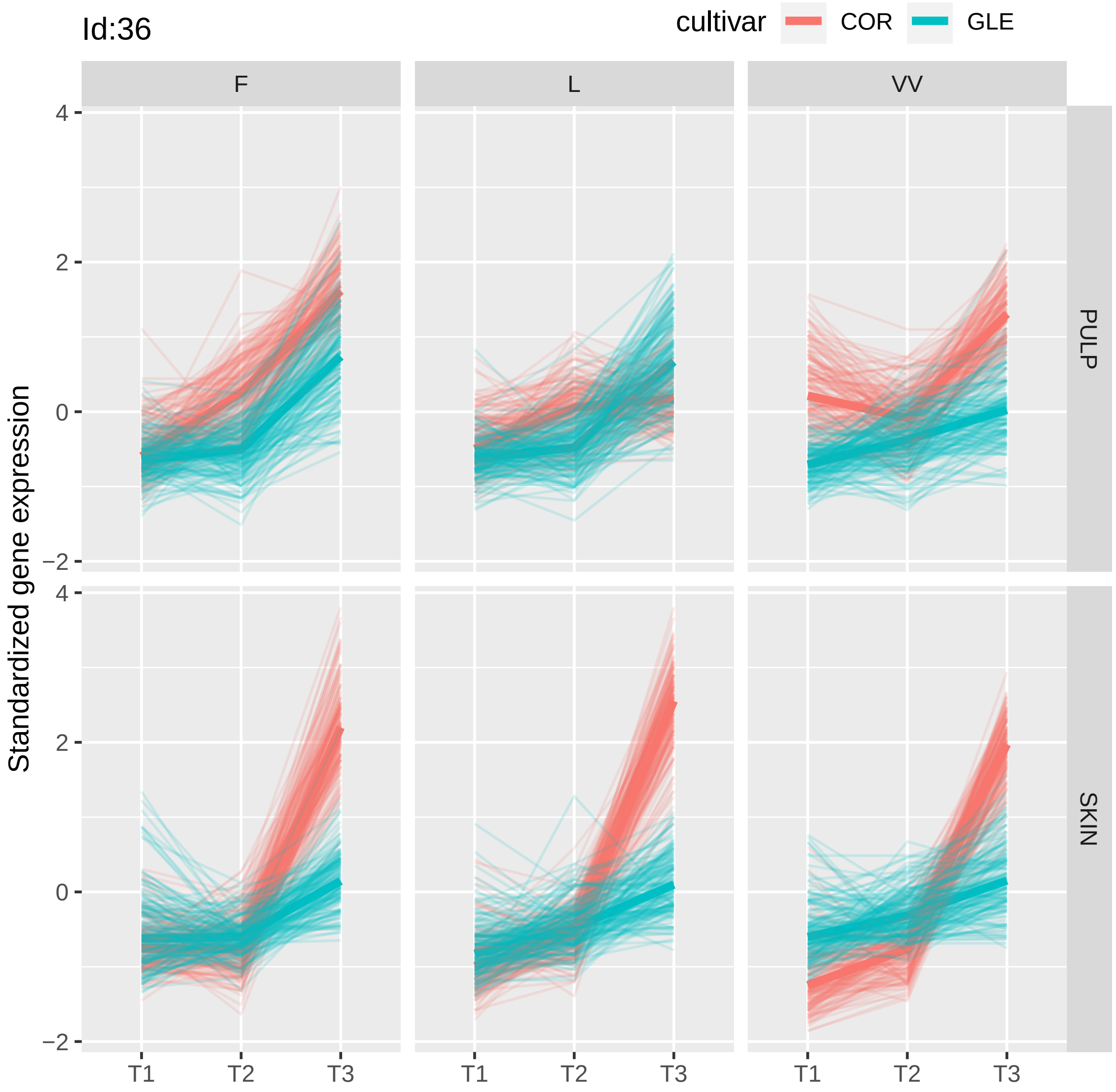

Id:36
cultivar
COR
GLE
F
L
VV
PULP
SKIN
Standardized gene expression
4
2
0
−2
T1
T2
T3

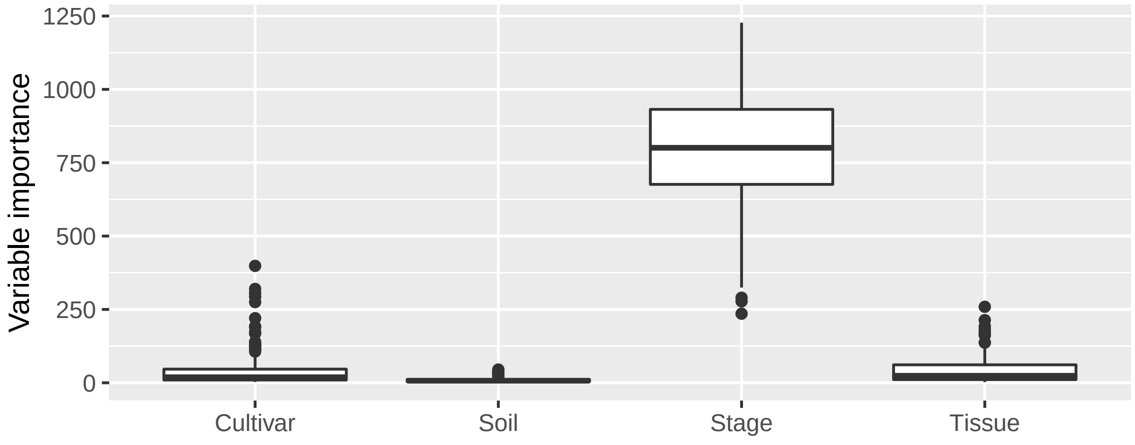

Variable importance
1250
1000
750
500
250
0
Cultivar
Soil
Stage
Tissue

Cluster no. 16

```
## Number of genes in the cluster: 109
## Homogeneity Index:    0.84
## Variable importance for Stage:     Rank =  16  - Median =  775.2
## Variable importance for Cultivar:  Rank = 100  - Median =   4.89
## Variable importance for Tissue:    Rank =  69  - Median =  38.33
## Variable importance for Soil:      Rank =  63  - Median =   5.11
##
## Gene ID               Gene Annotation
## VIT_15s0048g02090  -  Thioesterase family
## VIT_00s0299g00100  -  Arabidopsis thaliana homeobox protein 2
## VIT_15s0045g00380  -  Phosphoesterase
## VIT_13s0019g02300  -  Wall-associated receptor kinase-like 20
## VIT_13s0158g00080  -  Serine carboxypeptidase
## VIT_08s0040g01520  -  Indole-3-acetate beta-glucosyltransferase
## VIT_09s0018g02110  -  Unknown protein
## VIT_17s0000g05070  -  Phytochelatin synthetase
## VIT_03s0038g02230  -  Subtilisin-like serine protease 2
## VIT_14s0006g03100  -  No hit
## VIT_19s0014g05430  -  NDR1 (non race-specific disease resistance)
## VIT_05s0020g04490  -  No hit
## VIT_08s0040g02380  -  Hydrolase, alpha/beta fold
## VIT_09s0002g06920  -  Unknown protein
## VIT_05s0020g04500  -  Unknown protein
## VIT_14s0068g00010  -  feronia receptor-like kinase
## VIT_16s0022g00680  -  Multi-copper oxidase (SKU5)
## VIT_17s0000g02450  -  Tubulin beta-6
## VIT_18s0001g10550  -  LHCA5 (Photosystem I light harvesting complex gene 5)
## VIT_00s0179g00360  -  Unknown protein
## VIT_18s0001g13970  -  Myosin-related
## VIT_17s0053g00590  -  Kinase interacting protein
## VIT_18s0001g08340  -  Unknown
## VIT_07s0031g01320  -  Basic Leucine Zipper Transcription Factor (VvbZIP23)
## VIT_00s0207g00150  -  Ternary complex factor MIP1
## VIT_05s0049g00400  -  Desacetoxyvindoline 4-hydroxylase
## VIT_09s0002g06780  -  Unknown protein
## VIT_01s0026g01780  -  Leucine-rich repeat transmembrane
## VIT_19s0014g01470  -  Thioredoxin TTL1 (Tetratricopetide-repeat thioredoxin-like 1)
## VIT_10s0003g00860  -  Calmodulin-binding region IQD9
## VIT_00s0414g00040  -  Cellulose synthase CSLE1
## VIT_16s0022g01210  -  myb domain protein 85
## VIT_08s0058g01070  -  Xanthine/uracil permease
## VIT_03s0038g01130  -  Auxin-responsive
## VIT_06s0004g06960  -  BCL2 binding anthogene
## VIT_18s0001g10500  -  Abscisic acid 8` hydroxylase (CYP707A2) (VvA8H-CYP707A2.3)
## VIT_15s0048g00370  -  Transketolase, chloroplast precursor
## VIT_01s0026g00490  -  Nodulin
## VIT_19s0014g00090  -  Glucan endo-1,3-beta-glucosidase 4 precursor
## VIT_03s0017g01410  -  No hit
## VIT_19s0014g03660  -  LHCII type III, chloroplast precursor
## VIT_01s0137g00820  -  Phototropic-responsive NPH3
## VIT_03s0063g00010  -  LRX2 (leucine-rich repeat/extensin 2)
## VIT_08s0058g00640  -  Patellin-5
## VIT_13s0106g00230  -  Ankyrin
## VIT_14s0068g01200  -  Inducer of CBF expression 1 ICE1
```

```
## VIT_17s0000g04260  -  Universal stress protein (USP) family protein
## VIT_04s0044g00880  -  WD40
## VIT_06s0004g08360  -  NADH dehydrogenase I subunit N
## VIT_12s0134g00650  -  Anthocyanin 5-aromatic acyltransferase
## VIT_19s0015g00530  -  fasciclin arabinogalactan-protein (FLA1)
## VIT_17s0000g01120  -  Unknown protein
## VIT_00s1328g00020  -  Phytosulfokines PSK4
## VIT_15s0021g01040  -  CYP72A1
## VIT_17s0000g02440  -  Integral membrane protein
## VIT_17s0000g05300  -  Pre-mRNA cleavage complex II protein Clp1
## VIT_13s0067g00110  -  CYP72A1
## VIT_17s0000g04130  -  MYB divaricata
## VIT_00s2300g00010  -  Acyltransferase
## VIT_19s0014g01950  -  Anthocyanidin 3-O-glucosyltransferase
## VIT_11s0016g05300  -  Heme oxygenase 1
## VIT_10s0116g01610  -  Monocopper oxidase SKS4 (SKU5 Similar 4)
## VIT_11s0016g04810  -  BAS1 (PHYB activation tagged suppressor 1)
## VIT_18s0041g00920  -  UDP-glucose: anthocyanidin 5,3-O-glucosyltransferase
## VIT_18s0041g00930  -  UDP-glucose: anthocyanidin 5,3-O-glucosyltransferase
## VIT_03s0017g01470  -  Berberine bridge enzyme
## VIT_17s0000g08990  -  Alliinase
## VIT_14s0108g00590  -  FtsH protease
## VIT_18s0072g00820  -  VFB3 (VIER F- box  proteinE 3)
## VIT_05s0094g01290  -  Leucine-rich repeat family protein
## VIT_07s0005g00730  -  Pectinesterase family
## VIT_18s0001g06430  -  Homeobox-leucine zipper protein ATHB-6
## VIT_03s0038g01510  -  Unknown protein
## VIT_00s0414g00020  -  Cellulose synthase CSLE1
## VIT_01s0011g00830  -  Nodulin
## VIT_07s0031g00850  -  Patatin
## VIT_05s0094g01010  -  Indole-3-acetate beta-glucosyltransferase
## VIT_14s0030g01700  -  Unknown
## VIT_05s0094g01020  -  Indole-3-acetate beta-glucosyltransferase
## VIT_05s0094g01260  -  Allyl alcohol dehydrogenase
## VIT_15s0021g01150  -  Calcium-dependent protein kinase-related
## VIT_04s0044g01740  -  3-hydroxy-3-methylglutaryl-coenzyme A reductase 3
## VIT_12s0028g02850  -  Isoflavone methyltransferase/Orcinol O-methyltransferase 2 oomt2
## VIT_12s0055g00580  -  Receptor protein kinase
## VIT_08s0007g04950  -  Xyloglucan endotransglucosylase/hydrolase 32
## VIT_14s0066g02640  -  No hit
## VIT_17s0000g03990  -  Aspartyl protease
## VIT_01s0137g00470  -  SEC14 cytosolic factor
## VIT_13s0019g03570  -  Unknown protein
## VIT_03s0038g02450  -  Constans interacting protein 4
## VIT_07s0005g05100  -  Phytochrome  interacting  factor 3-like 5
## VIT_05s0020g01110  -  Pectinacetylesterase
## VIT_00s0194g00290  -  4-hydroxy-3-methylbut-2-enyl diphosphate reductase
## VIT_06s0004g03130  -  Auxin response factor 4
## VIT_00s0817g00010  -  Hydrolase
## VIT_07s0005g00030  -  Glutathione S-transferase (VvGST2)
## VIT_11s0016g01160  -  GCN5 N-acetyltransferase (GNAT)
## VIT_09s0018g01360  -  Cuticle protein
## VIT_00s0194g00280  -  Cyclin D3_1
## VIT_19s0027g01740  -  RPS5 (resistant to p. syringae 5)
```

```
## VIT_07s0005g02370  -  Germin-like protein 2 [Vitis vinifera]
## VIT_01s0011g02780  -  Unknown protein
## VIT_09s0002g00960  -  Inter-alpha-trypsin inhibitor heavy chain
## VIT_18s0076g00210  -  Alpha/beta hydrolase fold
## VIT_13s0067g01640  -  Lectin-like receptor kinase 7;2
## VIT_09s0018g01340  -  Cuticle protein
## VIT_01s0137g00050  -  myb family
## VIT_04s0044g00620  -  Scarecrow gene regulator-like
## VIT_07s0255g00110  -  WD40
```

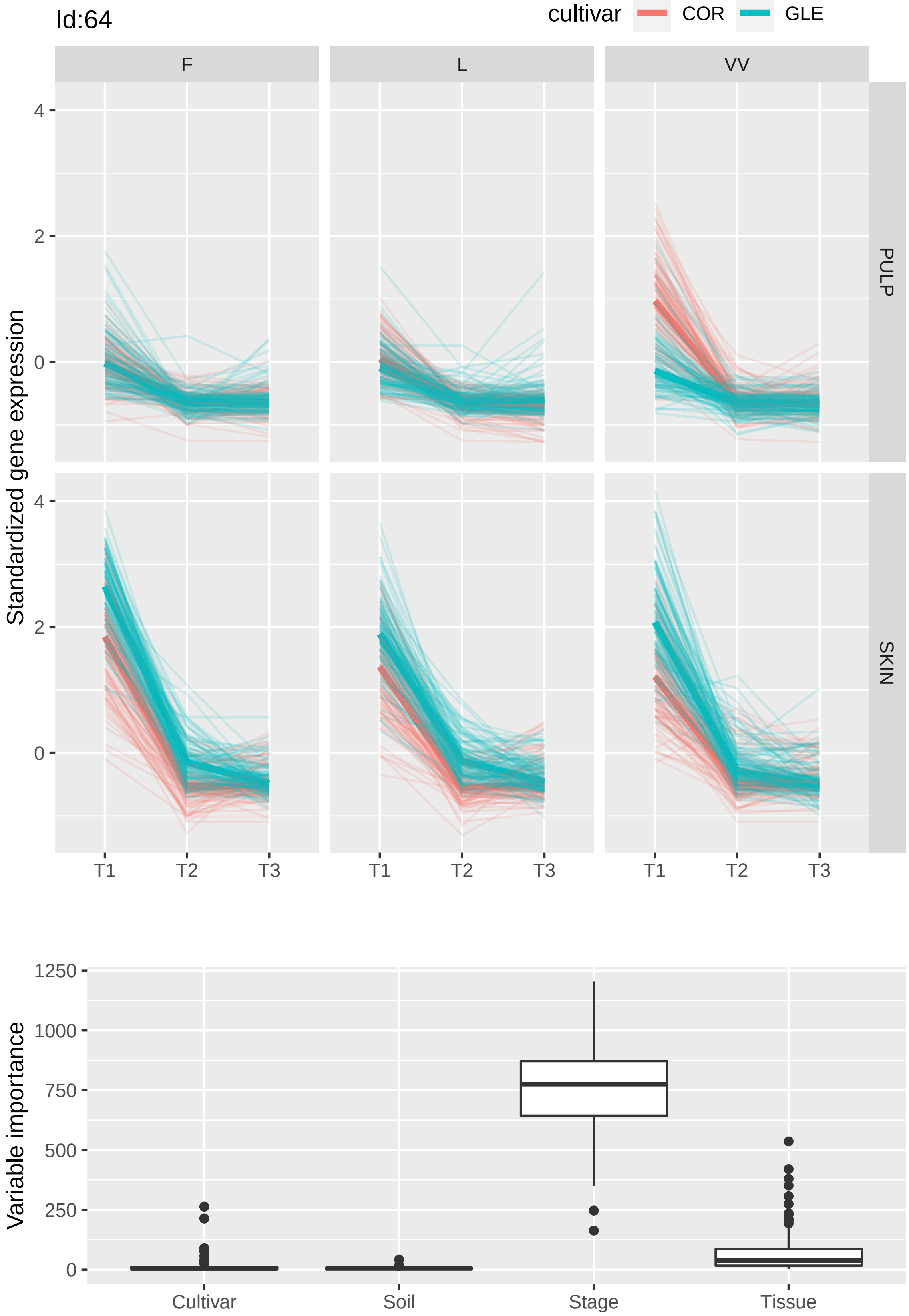
Id:64
cultivar
COR
GLE
F
L
VV
PULP
SKIN
Standardized gene expression
4
2
0
T1
T2
T3
Variable importance
1250
1000
750
500
250
0
Cultivar
Soil
Stage
Tissue

Cluster no. 17

```
## Number of genes in the cluster: 25
## Homogeneity Index:    0.66
## Variable importance for Stage:     Rank =  17  - Median =  743.6
## Variable importance for Cultivar:  Rank =  61  - Median =  25.86
## Variable importance for Tissue:    Rank =  65  - Median =  67.47
## Variable importance for Soil:      Rank =   4  - Median =  20.45
##
## Gene ID               Gene Annotation
## VIT_13s0019g00300  -  Sensitivity to red light reduced protein (SRR1)
## VIT_14s0066g01330  -  No hit
## VIT_05s0062g01190  -  Pirin
## VIT_04s0023g01800  -  Maf, septum formation protein
## VIT_13s0067g01960  -  Dehydration Responsive Element-Binding Transcription Factor (VvDREB19)
## VIT_12s0057g00240  -  Unknown protein
## VIT_19s0014g03730  -  No hit
## VIT_19s0014g02180  -  No hit
## VIT_17s0000g09650  -  Glyoxal oxidase
## VIT_06s0004g03980  -  Ankyrin
## VIT_18s0001g10470  -  Protein kinase MK6
## VIT_17s0000g02140  -  3-hydroxyisobutyrate dehydrogenase
## VIT_17s0000g00410  -  No hit
## VIT_02s0025g03730  -  Translation initiation factor eIF-2B alpha subunit
## VIT_19s0177g00110  -  Translation initiation factor eIF-1
## VIT_13s0019g03760  -  Lateral organ boundaries protein 11
## VIT_05s0049g00740  -  No hit
## ENSRNA049468488  -
## VIT_07s0005g06610  -  Unknown protein
## VIT_18s0086g00320  -  No hit
## ENSRNA049469858  -
## VIT_09s0002g04180  -  Unknown protein
## VIT_18s0001g08870  -  Unknown
## VIT_19s0085g00400  -  No hit
## VIT_14s0030g01730  -  No hit
```

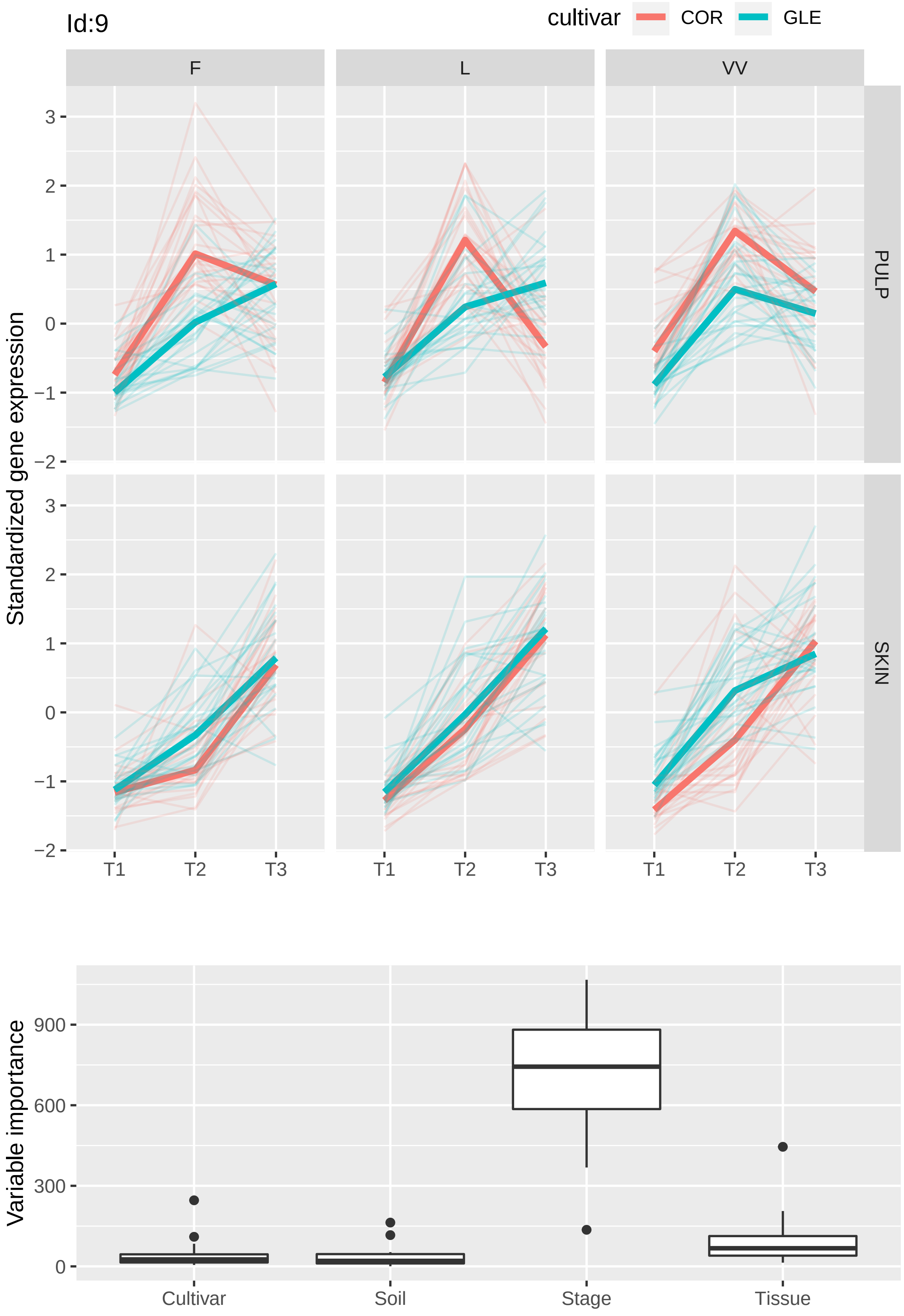

Id:9
cultivar
COR
GLE
F
L
VV
PULP
SKIN
Standardized gene expression
3
2
1
0
−1
−2
T1
T2
T3
Variable importance
900
600
300
0
Cultivar
Soil
Stage
Tissue

Cluster no. 18

```
## Number of genes in the cluster: 47
## Homogeneity Index:    0.79
## Variable importance for Stage:     Rank =  18  - Median =  737.7
## Variable importance for Cultivar:  Rank =  72  - Median =  14.73
## Variable importance for Tissue:    Rank =  90  - Median =  14.64
## Variable importance for Soil:      Rank =   9  - Median =  10.78
##
## Gene ID                Gene Annotation
## VIT_12s0059g01790  -  Caffeic acid O-methyltransferase (VvOMT1)
## VIT_00s0541g00020  -  Basic Leucine Zipper Transcription Factor (VvbZIP55)
## VIT_15s0048g02680  -  Calmodulin-binding region IQD17
## VIT_12s0059g01750  -  Caffeic acid O-methyltransferase (VvOMT2)
## VIT_10s0003g02430  -  flavonol synthase
## VIT_08s0007g01320  -  Proton-dependent oligopeptide transport (POT) family protein
## VIT_05s0020g00960  -  No hit
## VIT_07s0129g00200  -  Cyclin-D5-1
## VIT_14s0060g01960  -  Pectinesterase; Pectinesterase inhibitor
## VIT_04s0044g02010  -  Gibberellin 20 oxidase 2
## VIT_11s0037g00010  -  ERF/AP2 Gene Family (VvRAV6)
## VIT_11s0016g01410  -  Mechanosensitive ion channel
## VIT_01s0011g05360  -  High mobility group protein HMGI/Y-1
## VIT_06s0004g03930  -  Leucine-rich repeat protein kinase
## VIT_06s0004g07880  -  Allergen
## VIT_01s0011g06470  -  Galacturonic acid reductase
## VIT_06s0061g00700  -  Auxin-regulated protein
## VIT_13s0067g00830  -  R protein  PRF  disease resistance protein
## VIT_11s0037g00020  -  No hit
## VIT_16s0013g00650  -  Histone-lysine N-methyltransferase, H3 lysine-9 specific SUVH5
## VIT_18s0001g14130  -  Zinc finger (C2H2 type) family
## VIT_07s0151g00440  -  AP2 domain containing protein
## VIT_15s0048g00940  -  ATP-dependent DNA helicase 2 subunit 2
## VIT_18s0001g05950  -  Cis-zeatin O-beta-D-glucosyltransferase
## VIT_19s0027g00320  -  Unknown
## VIT_12s0034g02530  -  R protein disease resistance protein
## VIT_09s0002g02220  -  Protein kinase CDG1
## VIT_06s0061g01220  -  No hit
## VIT_08s0007g01310  -  Proton-dependent oligopeptide transport (POT) family protein
## VIT_05s0077g01280  -  Glycosyl hydrolase family 3 beta xylosidase BXL1
## VIT_11s0103g00520  -  Unknown
## VIT_08s0007g08500  -  Nonsense-mediated mRNA decay NMD3
## VIT_07s0197g00210  -  R protein disease resistance protein
## VIT_04s0044g01260  -  SEU3B protein
## VIT_17s0000g04340  -  Unknown protein
## VIT_14s0068g00040  -  No hit
## VIT_16s0098g01560  -  Glucan endo-1,3-beta-D-glucosidase
## VIT_05s0029g01300  -  MAGE (melanoma antigen-encoding gene)
## VIT_06s0080g00780  -  NAC domain-containing protein (VvNAC74)
## VIT_13s0067g01310  -  R protein disease resistance protein
## VIT_06s0004g02930  -  Zinc finger (C3HC4-type ring finger)
## VIT_18s0001g07650  -  VIN3
## VIT_08s0007g03480  -  No hit
## VIT_10s0003g01980  -  RKF1 (receptor-like kinase in flowers 1)
## VIT_11s0016g01460  -  BRXL4 (Brevis radix like 4)
## VIT_13s0047g01010  -  R protein MLA10
```

```
## VIT_19s0014g03450  -  Unknown
```

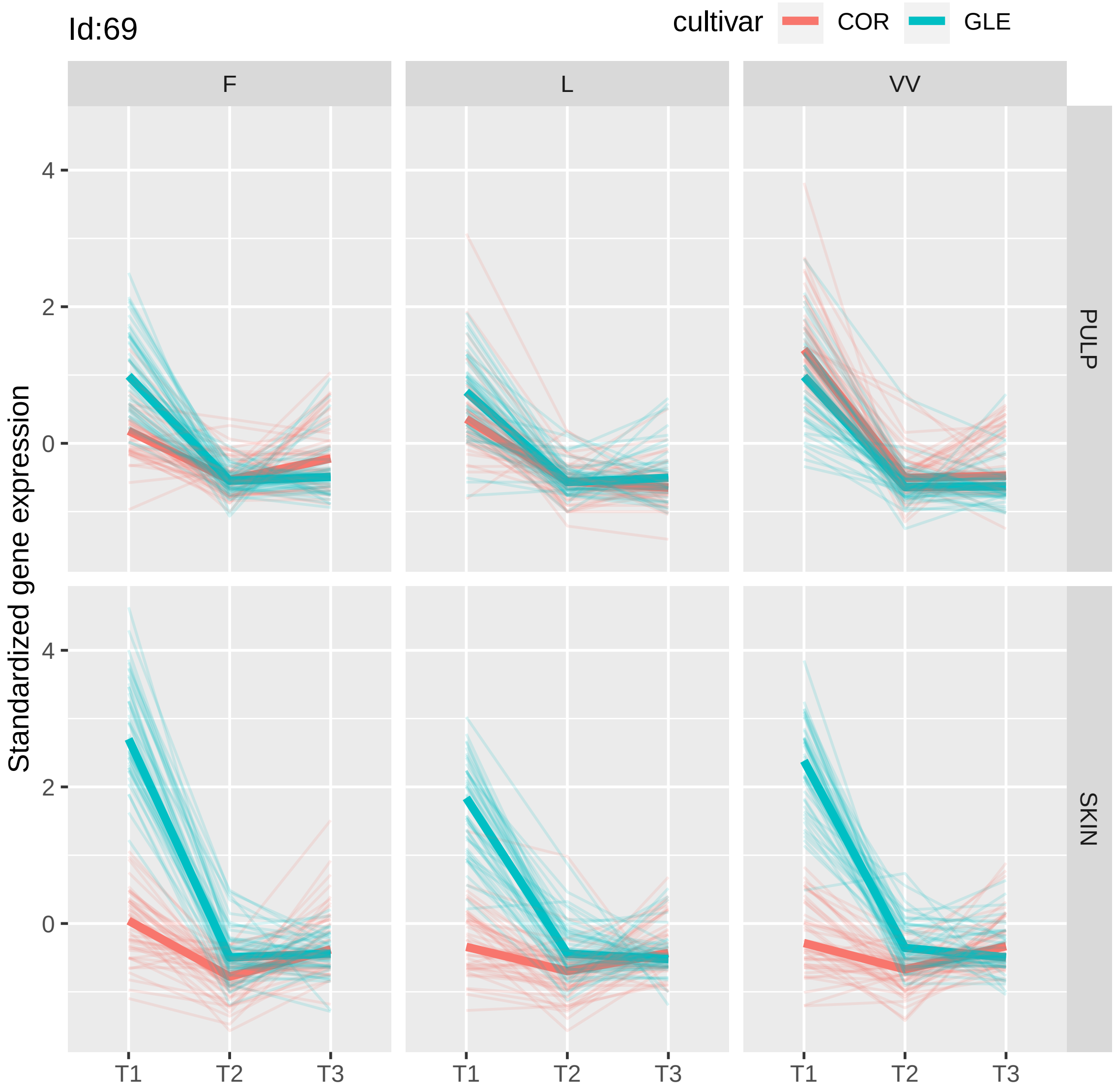
Id:69
cultivar
COR
GLE
F
L
VV
PULP
SKIN
Standardized gene expression
4
2
0
T1
T2
T3

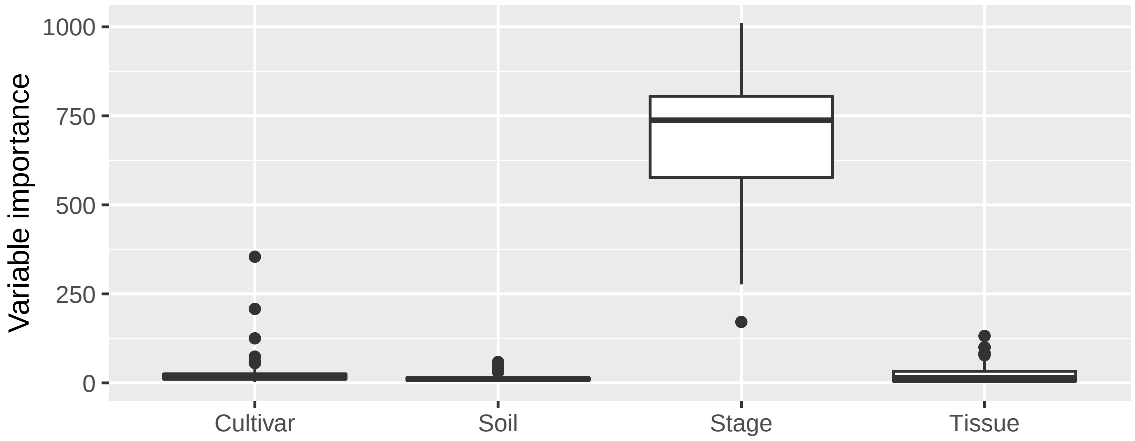
Variable importance
1000
750
500
250
0
Cultivar
Soil
Stage
Tissue

Cluster no. 19

```
## Number of genes in the cluster: 108
## Homogeneity Index:    0.88
## Variable importance for Stage:     Rank =  19  - Median =  690.7
## Variable importance for Cultivar:  Rank =  48  - Median =  48.33
## Variable importance for Tissue:    Rank =  60  - Median =  82.17
## Variable importance for Soil:      Rank =  34  - Median =   6.18
##
## Gene ID                Gene Annotation
## VIT_13s0064g01200  -  No hit
## VIT_18s0001g10480  -  Unknown protein
## VIT_11s0037g00130  -  fip1 motif-containing protein
## VIT_18s0001g02030  -  Signal peptidase 1
## VIT_18s0001g14350  -  No hit
## VIT_19s0015g01050  -  Unknown protein
## VIT_04s0008g06710  -  Zinc finger (C2H2 type) family
## VIT_01s0011g00510  -  Unknown protein
## VIT_16s0013g02160  -  No hit
## VIT_01s0011g04680  -  Glycosyltransferase family 14
## VIT_11s0037g00150  -  WRKY DNA-binding protein 32 (WRKY-14), WRKY Transcription Factor (VvWR
## VIT_02s0025g00220  -  Chlororespiratory reduction 4 (CRR4)
## VIT_07s0005g05640  -  Unknown protein
## VIT_06s0004g04240  -  Histone H2B
## VIT_08s0040g00140  -  Unknown protein
## VIT_02s0033g01070  -  Anthraniloyal-CoA: methanol anthraniloyal transferase
## VIT_08s0007g05940  -  Unknown
## VIT_08s0007g08350  -  Cyclin-related
## VIT_09s0018g01490  -  Anthraniloyal-CoA: methanol anthraniloyal transferase
## VIT_15s0048g02120  -  Myb domain protein 3R2
## VIT_19s0015g01060  -  Unknown protein
## VIT_18s0001g05600  -  Synaptonemal complex protein 1 (ZYP1A)
## VIT_04s0008g00660  -  Early flowering 3
## VIT_08s0007g04680  -  Expansin  (VvEXPA12)
## VIT_18s0001g09710  -  MRG
## VIT_11s0016g05600  -  Zinc finger (ubiquitin-hydrolase) domain-containing
## VIT_14s0060g02100  -  Pentatricopeptide (PPR) repeat-containing
## VIT_16s0098g00580  -  Zinc finger protein UFM
## VIT_06s0004g01850  -  Cytochrome c-type biogenesis protein
## VIT_12s0055g00420  -  BZIP transcription factor (VvABF-6), Basic Leucine Zipper Transcriptio
## VIT_17s0000g04830  -  DNA Polymerase epsilon, subunit B
## VIT_17s0119g00330  -  Unknown protein
## VIT_09s0002g01510  -  No hit
## VIT_03s0091g00200  -  DNA repair protein REV1
## VIT_06s0004g08330  -  No hit
## VIT_07s0005g00280  -  Zinc finger (c3hc4-type ring finger) nitrogen limitation adaptation
## VIT_06s0080g00370  -  F-box domain containing protein
## VIT_08s0032g00680  -  SPX (SYG1/Pho81/XPR1) domain-containing protein
## VIT_05s0102g00870  -  R protein  PRF  disease resistance protein
## VIT_17s0119g00310  -  Unknown protein
## VIT_11s0118g00370  -  Oxidoreductase, 2OG-Fe(II) oxygenase
## VIT_03s0038g00300  -  Minichromosome maintenance protein 4
## VIT_09s0018g02080  -  Coatomer beta subunit
## VIT_15s0046g01370  -  Carrier protein, Mitochondrial
## VIT_11s0016g05620  -  TPR4/WSIP2 (topless-related 4)
## VIT_00s0184g00040  -  Mini-chromosome maintenance protein MCM6
```

```
## VIT_14s0068g01010  -  Unknown protein
## VIT_07s0104g00230  -  IWS1 C-terminus
## VIT_15s0048g02270  -  No apical meristem cup-shaped cotyledon2
## VIT_08s0040g02850  -  RSZ33 (Arginine/serine-rich Zinc knuckle-containing protein 33)
## VIT_10s0116g00980  -  Unknown protein
## VIT_14s0068g00710  -  DEAD/DEAH box helicase
## VIT_05s0029g01130  -  Pentatricopeptide (PPR) repeat-containing protein
## VIT_16s0098g01230  -  Cytochrome b5
## VIT_13s0064g00130  -  R protein disease resistance protein
## VIT_17s0000g09030  -  Disease resistance protein (NBS-LRR class)
## VIT_17s0000g03440  -  EMB2454 (embryo defective 2454)
## VIT_15s0046g03360  -  Unknown protein
## VIT_06s0004g05570  -  Subtilase
## VIT_05s0020g01360  -  Myb family
## VIT_18s0001g10560  -  Myosin heavy chain
## VIT_02s0012g00060  -  Unknown
## ENSRNA049469370  -
## VIT_17s0000g08910  -  Unknown protein
## VIT_11s0052g00590  -  Unknown protein
## VIT_15s0021g01460  -  RWD domain-containing protein
## VIT_10s0003g01680  -  Trehalose synthase
## VIT_08s0040g02860  -  RSZ33 (Arginine/serine-rich Zinc knuckle-containing protein 33)
## VIT_00s0593g00010  -  Pentatricopeptide (PPR) repeat-containing protein
## VIT_18s0001g15340  -  Pentatricopeptide (PPR) repeat-containing protein
## VIT_05s0094g00530  -  Calmodulin binding protein
## VIT_09s0018g01740  -  Unknown
## VIT_03s0038g02950  -  Pentatricopeptide (PPR) repeat-containing protein
## VIT_10s0003g02610  -  Unknown protein
## VIT_05s0077g00020  -  Protein transporter
## VIT_04s0210g00040  -  Strictosidine synthase
## VIT_18s0122g00600  -  Unknown protein
## VIT_07s0005g00490  -  UDP-glucuronic acid decarboxylase 1
## VIT_00s0144g00290  -  No hit
## VIT_14s0083g00440  -  PHD finger transcription factor
## VIT_14s0060g02290  -  Splicing factor, arginine/serine-rich 1/9
## VIT_18s0117g00370  -  R protein L6
## VIT_05s0020g00010  -  Protein transporter
## VIT_07s0005g06480  -  EMB1974 (embryo defective 1974)
## VIT_08s0007g00200  -  Ankyrin repeat
## VIT_15s0021g01500  -  RWD domain-containing protein
## VIT_13s0019g04620  -  OTU cysteine protease
## VIT_02s0025g02990  -  Import inner membrane translocase subunit Tim13, Mitochondrial
## VIT_15s0046g02370  -  Unknown protein
## VIT_01s0011g06290  -  Purple acid phosphatase 3 ATPAP3/PAP3
## VIT_02s0012g01750  -  NHL8 (NDR1/HIN1-like 8)
## VIT_08s0007g03010  -  Hydrolase, alpha/beta fold
## VIT_19s0014g02870  -  Cellulase
## VIT_19s0090g00450  -  Unknown protein
## VIT_05s0049g01390  -  Unknown protein
## VIT_07s0031g02350  -  Heat shock protein binding
## VIT_05s0049g00470  -  Electron transfer flavoprotein beta-subunit
## VIT_09s0002g02330  -  Nucleosome/chromatin assembly factor group A
## VIT_01s0011g04920  -  Mitogen-activated Protein Kinase (VvMPK1)
## VIT_07s0005g03940  -  ATBARD1/BARD1 (breast cancer associated ring 1);
## VIT_18s0075g00350  -  Sucrose-phosphate synthase isoform C
```

```
## VIT_12s0059g01290  -  Unknown protein
## VIT_11s0016g03720  -  Aspartate aminotransferase, cytoplasmic (Transaminase A)
## VIT_11s0037g00480  -  Unknown protein
## VIT_08s0105g00470  -  PHD finger-like domain-containing protein 5A
## VIT_17s0000g08620  -  U3 small nucleolar RNA-associated protein MPP10
## VIT_02s0025g01560  -  UDP-glucose 4-epimerase GEPI48
## VIT_09s0002g03950  -  Unknown protein
```

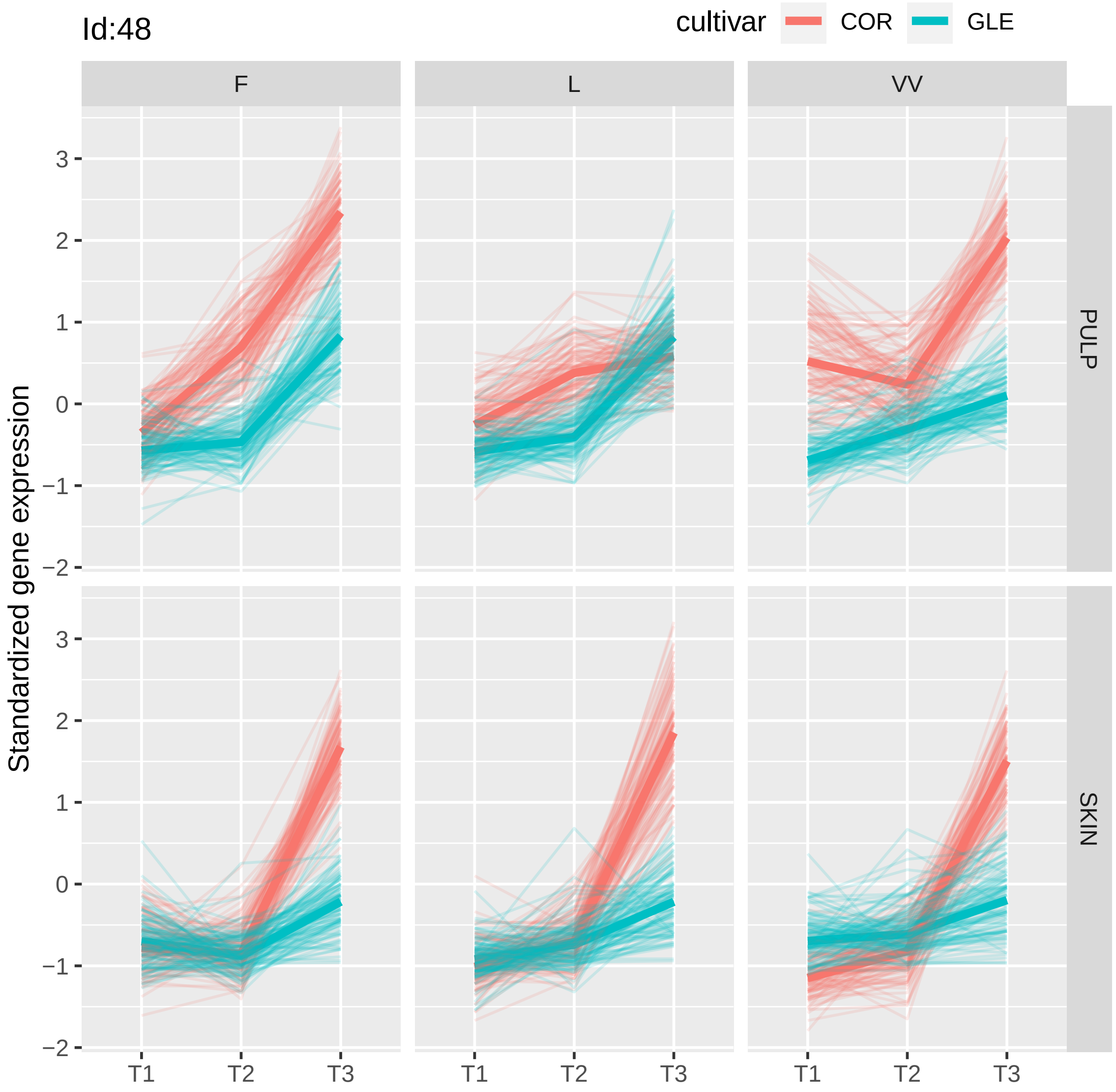
Id:48
cultivar
COR
GLE
F
L
VV
PULP
SKIN
Standardized gene expression
3
2
1
0
−1
−2
T1
T2
T3

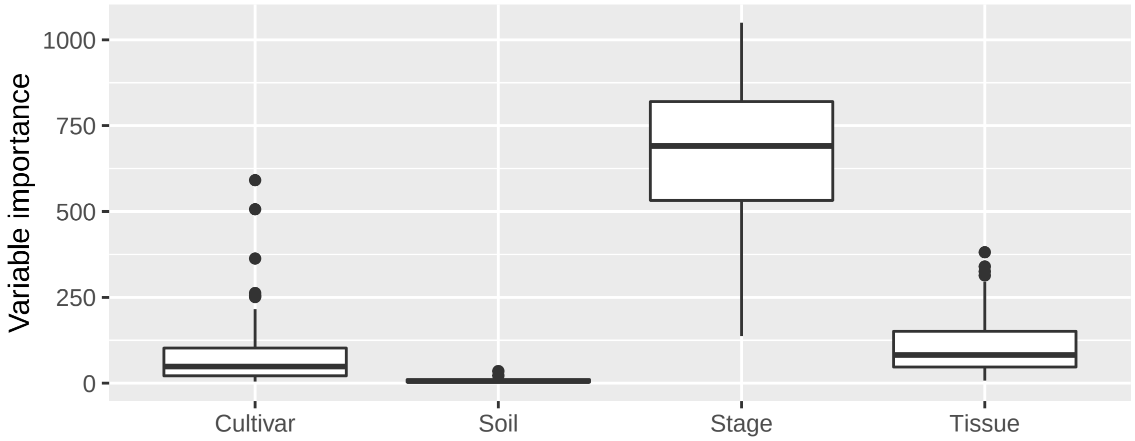
Variable importance
1000
750
500
250
0
Cultivar
Soil
Stage
Tissue

Cluster no. 20

```
## Number of genes in the cluster: 155
## Homogeneity Index:    0.88
## Variable importance for Stage:     Rank =  20  - Median =  659.8
## Variable importance for Cultivar:  Rank =  95  - Median =   7.15
## Variable importance for Tissue:    Rank =  48  - Median =  139.3
## Variable importance for Soil:      Rank =  83  - Median =   3.64
##
## Gene ID               Gene Annotation
## VIT_16s0050g01240  -  Heavy-metal-associated domain-containing protein
## VIT_13s0084g00090  -  Nodulin MtN21 family
## VIT_10s0003g04020  -  CBL- interacting  protein kinase 23 (VvCIPK04)
## VIT_08s0040g01370  -  Heavy-metal-associated domain-containing protein
## VIT_18s0001g08300  -  Tubulin alpha-6 chain
## VIT_01s0010g03710  -  Squamosa promoter-binding protein (VvSBP2)
## VIT_01s0010g02870  -  AAA-type ATPase
## VIT_10s0003g01830  -  Aquaporin NIP1;2
## VIT_01s0011g02600  -  No hit
## VIT_04s0008g05410  -  Serine hydrolase [Vitis vinifera]
## VIT_08s0056g01530  -  Anthranilate phosphoribosyltransferase
## VIT_18s0122g00990  -  Glutamate receptor Ionotropic
## VIT_00s0802g00010  -  No hit
## VIT_11s0016g05110  -  Sodium hypersensitive 1
## VIT_03s0132g00050  -  ATHVA22A (Arabidopsis thaliana HVA22 homologue A)
## VIT_15s0046g00410  -  Seven in absentia SINA1p
## VIT_07s0104g00830  -  Sugar transporter ERD6-like 7
## VIT_19s0015g00340  -  Unknown protein
## VIT_05s0094g00950  -  BT3 (BTB and TAZ domain protein 3)
## VIT_14s0030g00210  -  Sugar transporter ERD6-like 5
## VIT_01s0011g05670  -  Nudix hydrolase 18
## VIT_05s0020g00680  -  ABC Transporter (VvPDR17 - VvABCG47)
## VIT_05s0102g00610  -  No hit
## VIT_16s0013g02030  -  Unknown protein
## VIT_14s0060g00990  -  Unknown
## VIT_16s0050g02340  -  Regulator of nonsense transcripts 1
## VIT_01s0010g02090  -  Indeterminate(ID)-domain 5
## VIT_16s0022g01870  -  Unknown protein
## VIT_00s0389g00040  -  CYP72A1
## VIT_02s0241g00030  -  Sterol 4-alpha-methyl-oxidase 1 (SMO1)
## VIT_18s0001g09910  -  L-asparaginase
## VIT_11s0052g01510  -  MATE efflux family protein
## VIT_18s0001g12970  -  Ethylene-responsive protein
## VIT_19s0014g00300  -  Alpha,alpha-trehalose-phosphate synthase
## VIT_05s0077g01030  -  Unknown
## VIT_18s0001g14640  -  No hit
## VIT_18s0089g01020  -  Ethylene-responsive transcription factor
## VIT_10s0003g04770  -  Leucine-rich repeat transmembrane protein kinase
## VIT_11s0016g03140  -  ERD7 (EARLY-responsive TO dehydration 7)
## VIT_05s0020g04190  -  PINHEAD (Protein ZWILLE)
## VIT_17s0000g00710  -  DNA-binding protein
## VIT_16s0050g02320  -  Regulator of nonsense transcripts 1
## VIT_18s0041g00390  -  Cysteine protease inhibitor
## VIT_07s0031g02740  -  Auxin-responsive SAUR12
## VIT_19s0015g01560  -  Phenylalanine-tRNA ligase
## VIT_02s0025g03640  -  Cu2+-exporting ATPase HMA5 (heavy metal ATPase 5)
```

```
## VIT_11s0016g00960  -  Subtilase
## VIT_02s0025g00510  -  Respiratory burst oxidase protein F (RBOHF)
## VIT_17s0000g00720  -  DNA-binding protein
## VIT_12s0028g00050  -  Beta-1,3 glucanase
## VIT_02s0087g00840  -  ABC Transporter (VvWBC29 - VvABCG29)
## VIT_18s0001g08240  -  Leucine-rich repeat transmembrane protein kinase
## VIT_11s0016g02880  -  NAC domain-containing protein (VvNAC19)
## VIT_10s0116g00500  -  Myb caprice CPC
## VIT_00s0732g00010  -  Homeobox-leucine zipper protein 22
## VIT_03s0038g04550  -  Unknown
## VIT_18s0089g00970  -  No hit
## VIT_04s0008g05400  -  Serine hydrolase [Vitis vinifera]
## VIT_01s0010g03540  -  Microtubule associated protein (MAP65-6)
## VIT_11s0037g00960  -  NIK1 (NSP- interacting  kinase 1)
## VIT_00s0340g00040  -  Beta-hexosaminidase beta chain precursor
## VIT_11s0052g01540  -  MATE efflux family protein
## VIT_01s0026g02550  -  MYR1 (MYB-related protein 1)
## VIT_08s0032g00190  -  F-box family protein
## VIT_16s0050g01580  -  UDP-glucose: anthocyanidin 5,3-O-glucosyltransferase
## VIT_00s0361g00040  -  Anthocyanidin reductase (VvANR) [Vitis vinifera] GeneID: 100232981
## VIT_12s0035g00990  -  Big apical meristem 1 BAM1
## VIT_08s0032g00300  -  Annexin A3
## VIT_02s0025g03680  -  Tubulin beta-8 chain
## VIT_09s0018g01220  -  Zinc finger (C3HC4-type ring finger)
## VIT_03s0038g01350  -  Glutamine amidotransferase class-I
## VIT_03s0063g02010  -  Protease
## VIT_17s0000g00770  -  NAC domain-containing protein (VvNAC07)
## VIT_07s0141g00290  -  IAA16
## VIT_09s0002g01890  -  Hydroxyproline-rich glycoprotein
## VIT_12s0028g02520  -  TCP family transcription factor 4
## VIT_04s0008g02760  -  Unknown protein
## VIT_13s0019g01650  -  Expansin  (VvEXPA13)
## VIT_07s0129g00970  -  Protein kinase family
## VIT_13s0019g01830  -  Calmodulin binding protein
## VIT_19s0015g01600  -  Phenylalanine-tRNA ligase
## VIT_00s0225g00120  -  Unknown protein
## VIT_15s0021g01340  -  Phagocytosis and cell motility protein ELMO1
## VIT_00s1353g00010  -  BAM1 (big apical meristem 1)
## VIT_07s0129g00030  -  Short-root transcription factor (SHR)
## VIT_17s0000g08870  -  Carboxyl-terminal proteinase
## VIT_13s0019g05230  -  NAC domain-containing protein (VvNAC21)
## VIT_11s0016g03240  -  Avr9/Cf-9 rapidly elicited protein 146
## VIT_14s0066g02020  -  Proton-dependent oligopeptide transport (POT) family protein
## VIT_11s0016g01710  -  Myosin-like protein XIK
## VIT_01s0011g02520  -  EXS family protein / ERD1/XPR1/SYG1
## VIT_18s0001g00170  -  Unknown protein
## VIT_08s0007g04150  -  RKL1 (Receptor-like kinase 1)
## VIT_14s0030g01760  -  Unknown protein
## VIT_14s0006g02160  -  SAM dependent carboxyl methyltransferase Methyltransf_7
## VIT_16s0050g02330  -  Regulator of nonsense transcripts 1
## VIT_06s0004g04140  -  myb domain protein 59
## VIT_19s0090g01600  -  Kinesin motor protein
## VIT_18s0001g00550  -  Rho guanyl-nucleotide exchange factor ROPGEF1
## VIT_14s0030g00410  -  Arabidopsis histidine phosphotransfer AHP1
## VIT_19s0027g00740  -  Inhibitor of apoptosis
```

```
## VIT_04s0069g00270  -  Glutamate receptor 2.8
## VIT_17s0000g05990  -  ABA-responsive protein (HVA22a)
## VIT_01s0127g00600  -  DC1 domain-containing protein
## VIT_11s0016g01370  -  Early flowering 4
## VIT_12s0028g00400  -  1,3-beta-glucan synthase
## VIT_13s0067g00990  -  Heat shock protein-related
## VIT_08s0007g03240  -  Carbonic anhydrase precursor
## VIT_07s0104g00490  -  Unknown
## VIT_04s0023g03680  -  Histidine kinase 1 (AHK1)
## VIT_07s0104g01390  -  Arsenate reductase (glutaredoxin)
## VIT_01s0026g01090  -  Unknown
## VIT_17s0000g10100  -  Response regulator ARR11 Type-B
## VIT_18s0001g00800  -  Tetracycline transporter protein
## VIT_13s0067g03780  -  Leucine-rich repeat protein kinase
## VIT_05s0020g02670  -  Ring zinc finger protein
## VIT_08s0007g00020  -  Cation/hydrogen exchanger 20 (CHX20)
## VIT_04s0008g04510  -  Potassium channel K+ rectifying channel 1
## VIT_08s0040g02190  -  Unknown
## VIT_07s0031g01910  -  Unknown protein
## VIT_14s0060g00440  -  Glucan endo-1,3-beta-glucosidase-related
## VIT_17s0000g00730  -  ATP binding / DNA binding
## VIT_02s0154g00310  -  Protease inhibitor/seed storage/lipid transfer protein (LTP)
## VIT_13s0019g02730  -  Unknown protein
## VIT_13s0019g03370  -  Myb family
## VIT_13s0156g00110  -  Chaperone BCS1 mitochondrial
## VIT_04s0069g00260  -  Glutamate receptor 2.8
## VIT_14s0006g01420  -  Ser/Thr receptor kinase
## VIT_19s0177g00210  -  Protein disulfide isomerase (PDIL) protein
## VIT_01s0011g06410  -  BRL2 (BRI1-like 2)
## VIT_05s0020g03340  -  Unknown protein
## VIT_00s0214g00040  -  Lectin phloem protein 2-like B1 PP2-B1
## VIT_05s0077g01420  -  Unknown protein
## VIT_02s0025g03010  -  Copper chaperone (CCH)
## VIT_10s0003g05150  -  Peptidoglycan-binding LysM
## VIT_19s0014g04670  -  basic helix-loop-helix (bHLH) family
## VIT_08s0040g01240  -  basic helix-loop-helix (bHLH) family
## VIT_15s0046g03430  -  Metal ion binding
## VIT_01s0150g00020  -  Receptor protein kinase
## VIT_18s0001g06880  -  Aminoacyl-tRNA synthetase, class Ia
## VIT_08s0007g01850  -  Glycine-rich protein
## VIT_14s0068g00730  -  Unknown protein
## VIT_02s0154g00010  -  Auxin-responsive SAUR11
## VIT_07s0005g03370  -  Unknown
## VIT_15s0048g02310  -  NAC domain-containing protein (VvNAC52)
## VIT_13s0064g01870  -  R protein MLA10
## VIT_08s0007g02380  -  Heat shock protein-related
## VIT_06s0004g04520  -  OBP3 (OBF-binding protein 3)
## VIT_13s0019g04050  -  TET6 (tetraspanin6)
## VIT_11s0149g00090  -  Clavata1 receptor kinase (CLV1)
## VIT_01s0011g01730  -  ATPP2-A9 (Phloem protein 2-A9)
## VIT_11s0149g00100  -  DICER-like 4
## VIT_11s0149g00120  -  DICER-like 4
## VIT_12s0028g01950  -  Protein kinase Xa21
## VIT_19s0138g00110  -  1,3-beta-glucan synthase
```

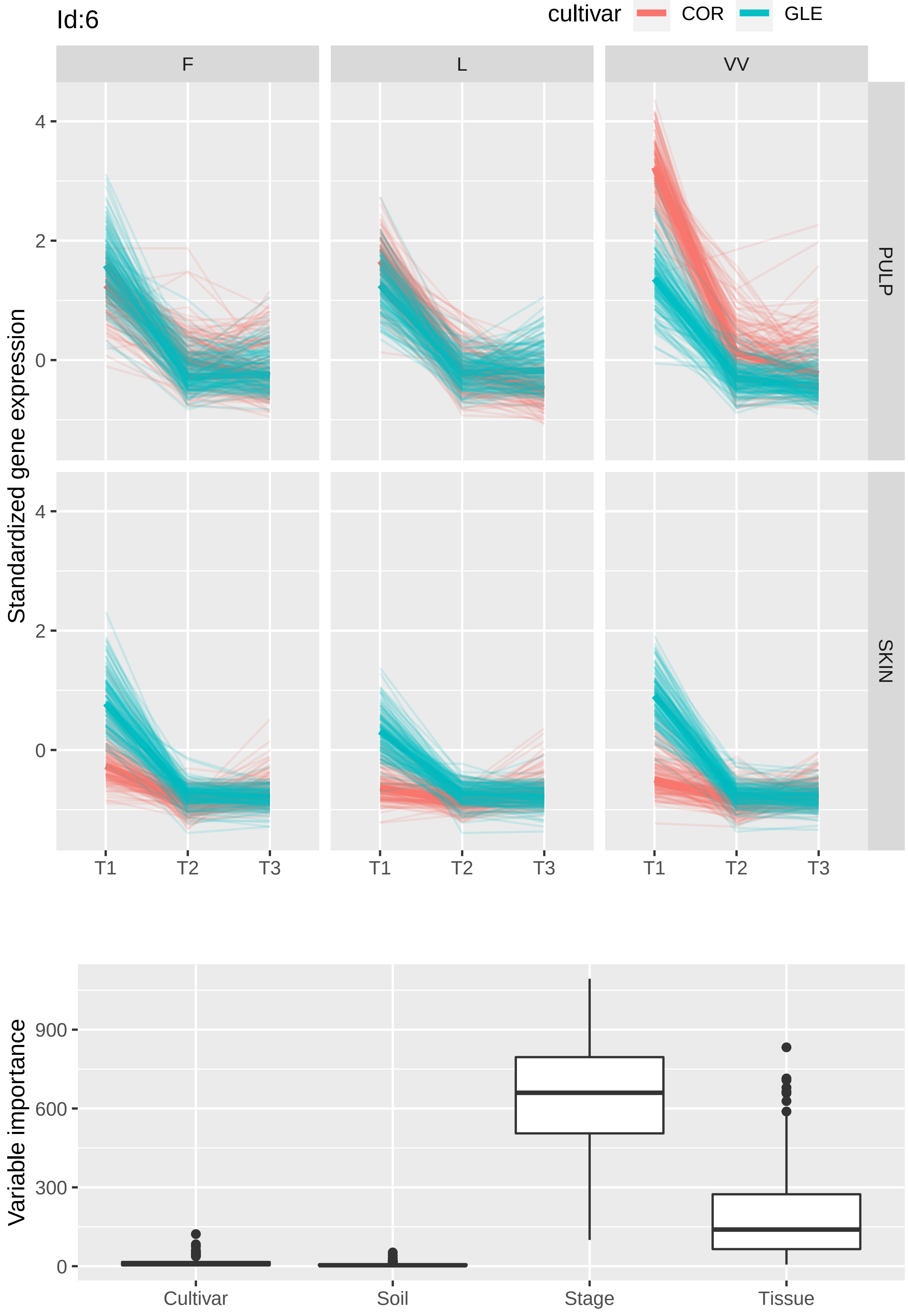
Id:6
cultivar
COR
GLE
F
L
VV
PULP
SKIN
Standardized gene expression
4
2
0
T1
T2
T3
Variable importance
900
600
300
0
Cultivar
Soil
Stage
Tissue

Cluster no. 21

```
## Number of genes in the cluster: 61
## Homogeneity Index:    0.63
## Variable importance for Stage:     Rank =  21  - Median =  639.7
## Variable importance for Cultivar:  Rank =  53  - Median =   40.7
## Variable importance for Tissue:    Rank =  75  - Median =     32
## Variable importance for Soil:      Rank =   8  - Median =  12.55
##
## Gene ID               Gene Annotation
## VIT_19s0014g02190  -  Tyrosine aminotransferase
## VIT_14s0006g03050  -  Unknown protein
## VIT_15s0048g00890  -  Cornichon family
## VIT_13s0019g01250  -  Metallophosphoesterase
## VIT_07s0031g01930  -  myb TKI1 (TSL-KINASE INTERACTING PROTEIN 1)
## VIT_01s0011g01980  -  fasciclin arabinogalactan-protein (FLA21)
## VIT_15s0046g02840  -  Ankyrin protein kinase
## VIT_14s0060g01360  -  Pentatricopeptide repeat
## VIT_12s0057g01320  -  Endoribonuclease E
## VIT_01s0011g03430  -  No hit
## VIT_11s0016g03860  -  Raspberry3
## VIT_00s0399g00060  -  Alternative oxidase / immutans protein (IM)
## VIT_08s0007g02530  -  ABA-responsive protein (HVA22) HVA22J
## VIT_15s0021g00330  -  Proton-dependent oligopeptide transport (POT) family protein
## VIT_15s0046g02210  -  Zinc finger (C3HC4-type ring finger)
## VIT_01s0026g02470  -  Zinc finger (C3HC4-type ring finger)
## VIT_01s0026g00860  -  Sterile alpha motif (SAM) domain-containing
## VIT_01s0011g01970  -  Pentatricopeptide (PPR) repeat
## VIT_13s0019g01810  -  Scarecrow transcription factor 14 (SCL14)
## VIT_09s0002g07130  -  CUE domain containing protein
## VIT_10s0116g00850  -  Serine/threonine Protein kinase BNK1
## VIT_01s0011g05620  -  No hit
## VIT_15s0048g02890  -  Ubiquitin family
## VIT_13s0067g03200  -  Proton-dependent oligopeptide transport (POT) family protein
## VIT_08s0007g00750  -  SRG1 (senescence-related gene 1)
## VIT_06s0004g00820  -  Ribosomal protein S2; Squalene/phytoene synthase
## VIT_01s0244g00100  -  Serine carboxypeptidase S10
## VIT_17s0000g00560  -  UPF0497 family
## ENSRNA049468811  -
## VIT_00s0347g00040  -  Avr9/Cf-9 rapidly elicited protein 20
## VIT_01s0011g05610  -  Caleosin
## VIT_11s0052g01380  -  Calmodulin-binding protein
## VIT_04s0008g02340  -  Exocyst subunit EXO70 protein
## VIT_05s0020g03080  -  Acyl-CoA synthetase long-chain member 6
## VIT_01s0011g03340  -  No hit
## VIT_13s0139g00010  -  R protein MLA10
## VIT_12s0059g02530  -  fringe-related protein
## VIT_18s0089g00170  -  1,4-beta-mannan endohydrolase
## VIT_08s0058g01390  -  WRKY Transcription Factor (VvWRKY25)
## VIT_19s0090g01170  -  UPF0041
## VIT_11s0016g00520  -  Auxin-responsive SAUR38
## VIT_05s0077g01290  -  Unknown protein
## VIT_15s0046g03190  -  myb domain protein 17
## VIT_18s0122g00370  -  Laccase
## VIT_00s1927g00020  -  Ubiquitin-conjugating enzyme E2 O
## VIT_18s0122g00390  -  Laccase
```

```
## VIT_13s0019g00380  -  R protein MLA10
## VIT_08s0007g07810  -  basic helix-loop-helix (bHLH) family
## VIT_12s0059g02520  -  fringe
## VIT_04s0008g04170  -  Unknown
## VIT_13s0019g00490  -  PHD zinc finger protein
## VIT_14s0030g00020  -  Unknown
## VIT_13s0139g00310  -  DNA-directed RNA polymerase II subunit E
## VIT_04s0008g04830  -  ABC Transporter (VvPDR31 - VvABCG61)
## VIT_06s0061g00220  -  Transcription initiation factor TFIIB
## VIT_14s0081g00110  -  Pentatricopeptide (PPR) repeat-containing
## VIT_09s0054g00840  -  Unknown protein
## VIT_12s0059g02510  -  Zinc finger (B-box type)
## VIT_10s0092g00560  -  Transducin protein
## VIT_14s0006g01150  -  ATP-binding cassette, sub-family F, member 1
## VIT_12s0028g01210  -  DNA-directed RNA Polymerase II subunit F
```

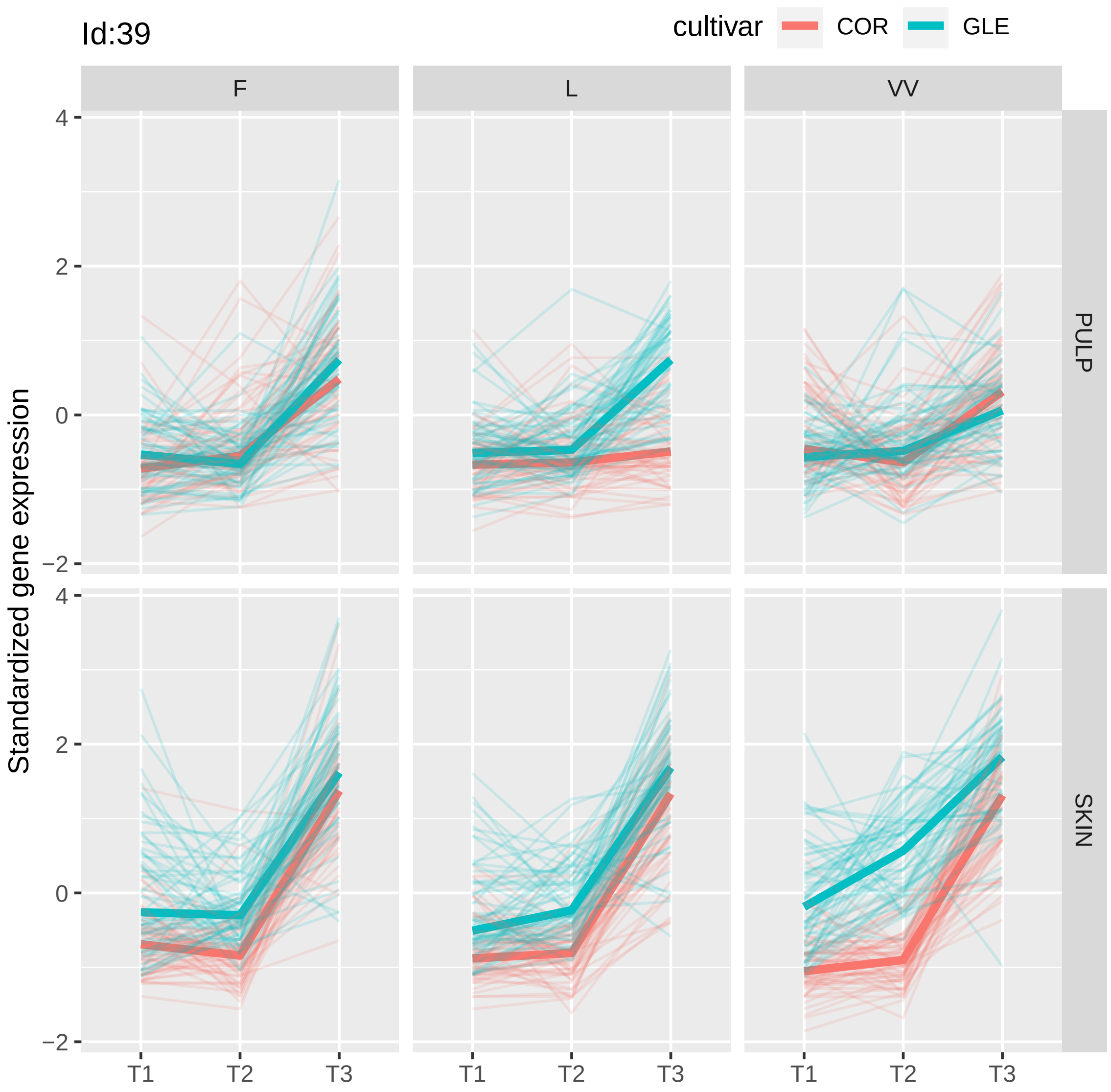
Id:39
cultivar
COR
GLE
F
L
VV
PULP
SKIN
Standardized gene expression
4
2
0
−2
T1
T2
T3

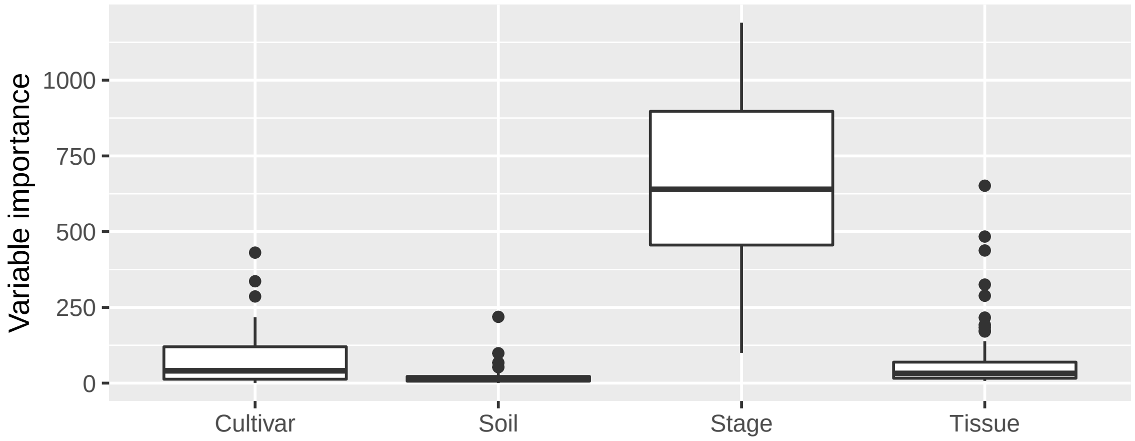
Variable importance
1000
750
500
250
0
Cultivar
Soil
Stage
Tissue

Cluster no. 22

```
## Number of genes in the cluster: 49
## Homogeneity Index:    0.91
## Variable importance for Stage:     Rank =  22  - Median =  619.3
## Variable importance for Cultivar:  Rank =  55  - Median =  37.28
## Variable importance for Tissue:    Rank = 101  - Median =   6.28
## Variable importance for Soil:      Rank =  51  - Median =   5.57
##
## Gene ID               Gene Annotation
## VIT_11s0016g05770  -  Alkaline alpha galactosidase 2
## VIT_03s0038g00470  -  Subtilase family protein
## VIT_14s0066g02350  -  Galactinol synthase
## VIT_17s0000g00070  -  Unknown protein
## VIT_03s0088g01200  -  Unknown protein
## VIT_07s0005g02230  -  S-adenosylmethionine synthetase 1 (SAM1)
## VIT_07s0129g00560  -  No hit
## VIT_01s0026g00910  -  Unknown
## VIT_04s0008g06150  -  No hit
## VIT_19s0093g00550  -  11,12 9-cis epoxycarotenoid dioxygenase (NCED3) (VvNCED3)
## VIT_19s0014g03800  -  RANGAP2 (RAN GTPASE activating protein 2)
## VIT_00s0207g00230  -  Phototropic-responsive NPH3
## VIT_01s0011g04290  -  Receptor serine/threonine kinase PR5K
## VIT_18s0122g00750  -  HSP20-like chaperone
## VIT_18s0072g00150  -  Unknown protein
## VIT_17s0053g00970  -  No hit
## VIT_05s0029g00990  -  Tripeptidyl-peptidase 2
## VIT_09s0002g02020  -  F-box family protein
## VIT_18s0001g08510  -  Lipase family
## VIT_01s0011g04300  -  Receptor serine/threonine kinase PR5K
## VIT_06s0004g00880  -  Protease inhibitor/seed storage/lipid transfer protein (LTP)
## VIT_07s0005g01300  -  Auxin-induced beta-glucosidase
## VIT_08s0056g01080  -  MATE efflux family protein
## VIT_16s0022g01430  -  Phosphate-induced protein 1
## VIT_04s0008g01720  -  Mechanosensitive ion channel
## VIT_09s0002g00330  -  Pectinesterase PME1
## VIT_01s0127g00850  -  Polygalacturonase BURP
## VIT_05s0020g02080  -  Zinc finger (C3HC4-type ring finger)
## VIT_07s0005g01310  -  Threonyl-tRNA synthetase, class IIa
## VIT_07s0141g00550  -  No hit
## VIT_12s0059g02150  -  Aconitate hydratase, cytoplasmic
## VIT_08s0007g00690  -  Laccase
## VIT_11s0016g05830  -  No hit
## VIT_18s0001g10210  -  PAB8 (poly(A) binding protein 8)
## VIT_04s0210g00030  -  Strictosidine synthase
## VIT_03s0091g01290  -  Serine carboxypeptidase S10
## VIT_01s0011g04080  -  Zinc finger (C3HC4-type ring finger)
## VIT_12s0134g00630  -  Quercetin 3-O-glucoside-6''-O-malonyltransferase
## VIT_00s0411g00010  -  Unknown
## VIT_12s0134g00590  -  Anthocyanidin 3-O-glucoside-6''-O-malonyltransferase
## VIT_03s0167g00010  -  Auxin response factor ARF6
## VIT_02s0087g00750  -  Proteinase inhibitor I4, serpin
## VIT_17s0000g08480  -  ATMYB66/WER/WER1 (WEREWOLF 1)
## VIT_19s0015g02940  -  CYP72A59
## VIT_01s0011g00030  -  ACT domain containing protein (ACR4)
## VIT_04s0008g00290  -  Clavata1 receptor kinase (CLV1)
```

```
## VIT_19s0090g00360  -  Unknown
## VIT_07s0031g03170  -  1-acyl-sn-glycerol-3-phosphate acyltransferase
## VIT_14s0030g00140  -  CC-NBS-LRR class
```

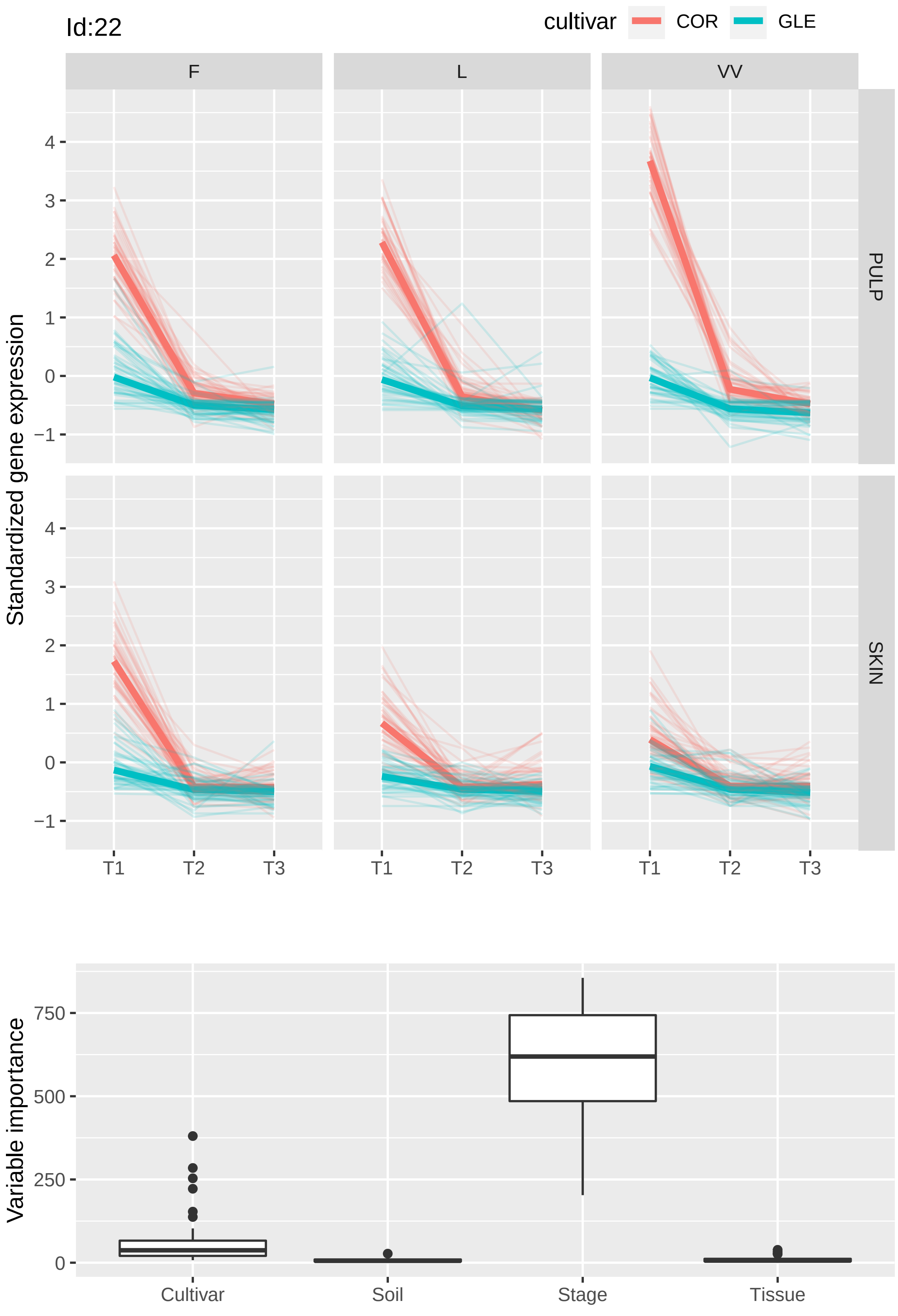
Id:22
cultivar
COR
GLE
F
L
VV
PULP
SKIN
Standardized gene expression
4
3
2
1
0
−1
T1
T2
T3
Variable importance
750
500
250
0
Cultivar
Soil
Stage
Tissue

Cluster no. 23

```
## Number of genes in the cluster: 102
## Homogeneity Index:    0.82
## Variable importance for Stage:     Rank =  23  - Median =  612.3
## Variable importance for Cultivar:  Rank =  52  - Median =  41.35
## Variable importance for Tissue:    Rank =  87  - Median =  15.64
## Variable importance for Soil:      Rank =  46  - Median =   5.73
##
## Gene ID                Gene Annotation
## VIT_09s0002g00550  -  Lipase GDSL 1
## VIT_15s0048g02280  -  NAC domain-containing protein (VvNAC54)
## VIT_00s0227g00200  -  formin protein AHF1
## VIT_11s0052g00140  -  Mannosyl-oligosaccharide 1,2-alpha-mannosidase
## VIT_00s0174g00330  -  formin protein AHF1
## VIT_00s0371g00100  -  Mannitol dehydrogenase
## VIT_11s0052g01650  -  Pathogenesis-related protein 1 precursor (PRP 1)
## VIT_03s0088g00320  -  Peptidase M50
## VIT_00s0261g00020  -  Unknown protein
## VIT_03s0063g02340  -  Dehydration-responsive protein (RD22)
## VIT_00s0202g00040  -  Ankyrin repeat protein
## VIT_14s0066g00970  -  Anthranilate phosphoribosyltransferase, chloroplast precursor
## VIT_08s0056g01140  -  Exostosin
## VIT_11s0052g00150  -  Mannosyl-oligosaccharide 1,2-alpha-mannosidase
## VIT_15s0107g00270  -  Mechanosensitive ion channel
## VIT_07s0151g00340  -  Sulfate transporter 3.1 (AST12) (AtST1)
## VIT_11s0016g03180  -  ABI1 (ABA insensitive 1)
## VIT_02s0025g02840  -  Ankyrin 3, epithelial isoform a
## VIT_00s0414g00030  -  Cellulose synthase CSLE1
## VIT_03s0038g02090  -  Phospholipase C.
## VIT_19s0014g02350  -  Squamosa promoter-binding protein (VvSBP18)
## VIT_17s0000g02650  -  myb domain protein 7
## VIT_14s0108g00150  -  Calmodulin-binding region IQD28
## VIT_19s0090g00530  -  Geranyl diphosphate synthase small subunit
## VIT_03s0063g00800  -  Carboxyesterase 12; CXE12
## VIT_14s0060g01730  -  No hit
## VIT_18s0001g12200  -  CYP721A1
## VIT_19s0015g01120  -  Clavata-like kinase
## VIT_01s0010g00720  -  Unknown
## VIT_09s0018g00630  -  Cycloartenol synthase
## VIT_13s0019g00730  -  Annexin ANN3
## VIT_13s0064g01320  -  CYP712A1
## VIT_11s0037g00570  -  Anthranilate N-benzoyltransferase
## VIT_00s0256g00020  -  Unknown
## VIT_07s0095g00180  -  S-receptor kinase 1
## VIT_11s0016g05840  -  Protease inhibitor/seed storage/lipid transfer protein (LTP)
## VIT_16s0013g00970  -  Ethylene responsive element binding factor 5
## VIT_11s0016g03640  -  Rac-like GTP-binding protein ARAC7 (GTPase protein ROP9)
## VIT_05s0077g01720  -  SAG101 (senescence-associated gene 101)
## VIT_17s0000g02110  -  Unknown
## VIT_16s0013g00990  -  ERF/AP2 Gene Family (VvERF105)
## VIT_19s0093g00350  -  Glutathione S-transferase 25 GSTU25
## VIT_18s0001g08420  -  Unknown
## VIT_11s0016g03030  -  Calmodulin binding protein
## VIT_00s2527g00010  -  Beta-fructosidase (BFRUCT3)
## VIT_05s0049g01120  -  Glutathione S-transferase 25 GSTU7
```

```
## VIT_18s0041g00500  -  Proton-dependent oligopeptide transport (POT) family protein
## VIT_04s0008g04010  -  RNA recognition motif (RRM)-containing protein
## VIT_07s0031g00500  -  Subtilase
## VIT_07s0129g00940  -  Protein kinase
## VIT_02s0087g00030  -  Alpha-glucosidase
## VIT_18s0041g00510  -  Proton-dependent oligopeptide transport (POT) family protein
## VIT_09s0054g01750  -  Unknown protein
## VIT_05s0020g04400  -  No hit
## VIT_14s0219g00230  -  Pectate lyase
## VIT_09s0002g08680  -  Unknown
## VIT_18s0001g08690  -  Unknown
## VIT_17s0000g08920  -  Ribitol dehydrogenase
## VIT_05s0077g02010  -  Epoxide hydrolase
## VIT_14s0060g02620  -  Unknown protein
## VIT_00s0256g00010  -  Unknown
## VIT_07s0031g00280  -  Anthranilate N-hydroxycinnamoyl/benzoyltransferase
## VIT_00s0809g00010  -  Unknown
## VIT_05s0029g01000  -  HcrVf3 protein
## VIT_04s0043g00370  -  Ammonium transporter 1;2
## VIT_03s0038g00490  -  GATA transcription factor 12
## VIT_13s0019g00750  -  Annexin ANN4
## VIT_03s0088g00150  -  Serine carboxypeptidase SCPL17
## VIT_05s0020g02880  -  ADP-glucose pyrophosphorylase
## VIT_03s0091g00690  -  Salt tolerance zinc finger
## VIT_10s0092g00250  -  Alpha-glucosidase 1 (AGLU1)
## VIT_10s0092g00260  -  Alpha-glucosidase 1 (AGLU1)
## VIT_01s0011g03890  -  Receptor serine/threonine kinase PR5K
## VIT_18s0001g10070  -  Receptor kinase
## VIT_19s0014g04630  -  S-locus protein kinase
## VIT_12s0059g02660  -  No hit
## VIT_09s0002g09250  -  CYP82A2
## VIT_05s0020g04240  -  Calcium-transporting ATPase 12 ACA12
## VIT_05s0077g02020  -  Epoxide hydrolase
## VIT_15s0046g01560  -  Unknown protein
## VIT_00s0207g00250  -  No hit
## VIT_08s0105g00310  -  Rhodanese domain-containing protein
## VIT_05s0020g04220  -  Calcium-transporting ATPase 13 ACA13
## VIT_01s0026g01030  -  Zinc finger (C3HC4-type ring finger)
## VIT_15s0046g02570  -  Unknown
## VIT_19s0015g00940  -  Glucose-6-phosphate/phosphate-tranlocator
## VIT_09s0002g09200  -  CYP82M1v3
## VIT_12s0028g00130  -  KCO6 (Ca2+ activated outward rectifying K+ channel 6)
## VIT_17s0000g04950  -  Steroid sulfotransferase
## VIT_00s0274g00050  -  Unknown protein
## VIT_00s0414g00060  -  Cellulose synthase CSLE1
## VIT_00s0233g00090  -  Vacuolar protein sorting 41 (VPS41)
## VIT_05s0049g00250  -  2-oxoglutarate-dependent dioxygenase
## VIT_05s0049g01090  -  Glutathione S-transferase 25 GSTU7
## VIT_09s0002g09270  -  CYP82M1v4
## VIT_15s0046g01970  -  Anthocyanidine rhamnosyl-transferase
## VIT_03s0038g01370  -  ABA-responsive protein (HVA22)HVA22H
## VIT_07s0129g00090  -  Ribosomal protein L26 (RPL26A) 60S
## VIT_05s0020g04330  -  Ca2+-ATPase 13 ACA13, plasma membrane
## VIT_11s0016g01610  -  Unknown
```

```
## VIT_06s0061g01200  -  No hit
## VIT_15s0048g00980  -  NADPH HC toxin reductase
```

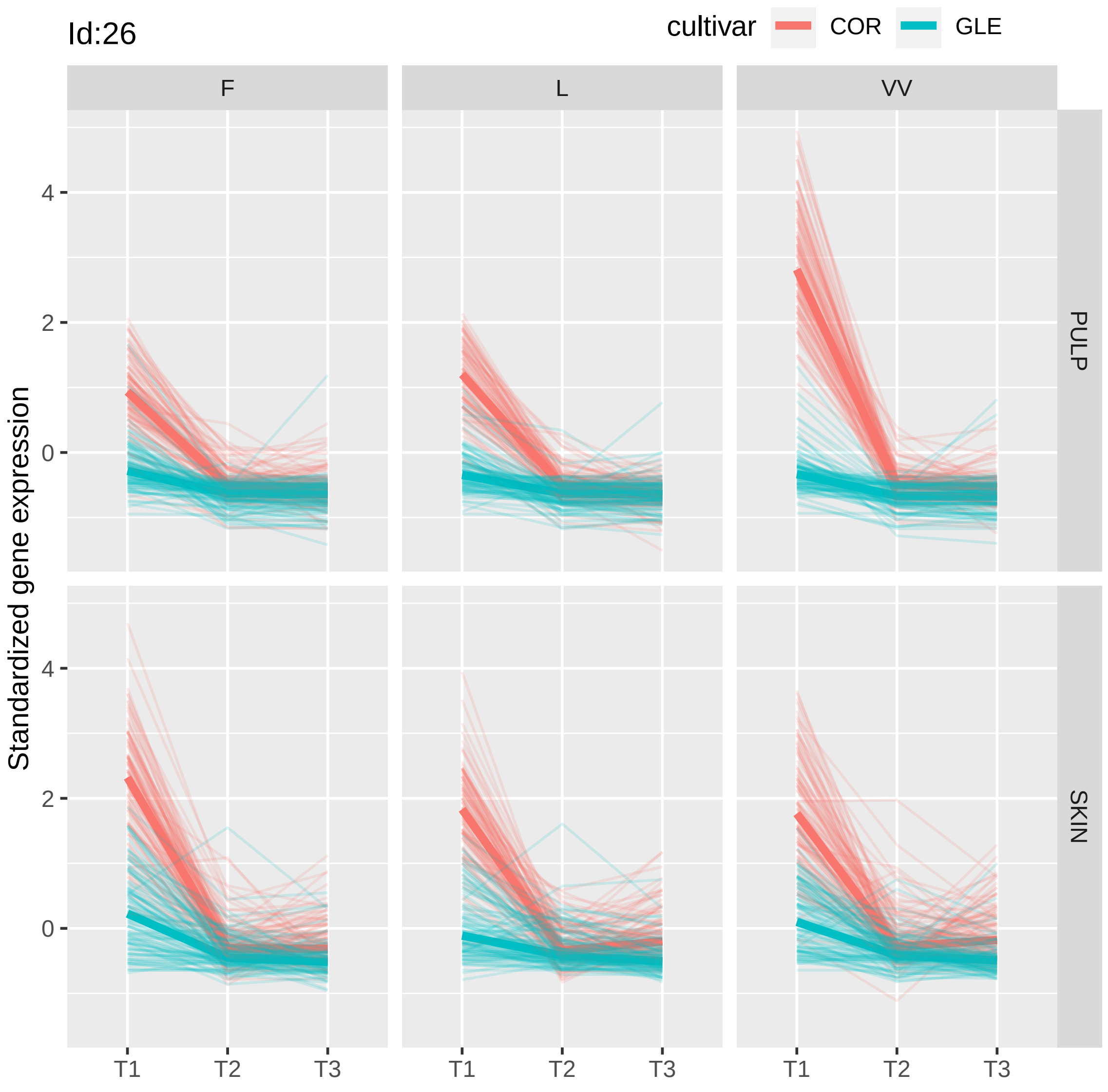

Id:26
cultivar
COR
GLE
F
L
VV
PULP
SKIN
Standardized gene expression
4
2
0
T1
T2
T3

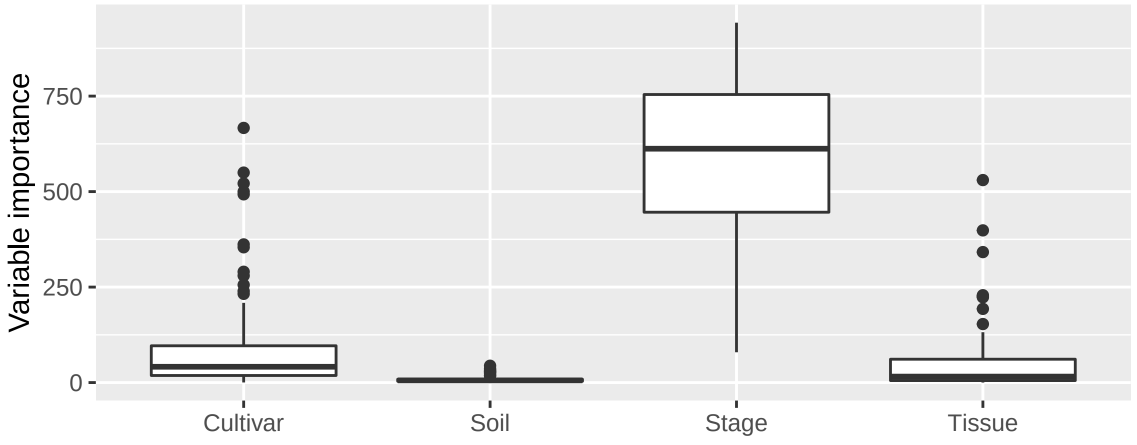

Variable importance
750
500
250
0
Cultivar
Soil
Stage
Tissue

Cluster no. 24

```
## Number of genes in the cluster: 89
## Homogeneity Index:    0.86
## Variable importance for Stage:     Rank =  24  - Median =  603.1
## Variable importance for Cultivar:  Rank =  99  - Median =   5.57
## Variable importance for Tissue:    Rank =  79  - Median =  28.32
## Variable importance for Soil:      Rank =  45  - Median =   5.75
##
## Gene ID               Gene Annotation
## VIT_09s0002g01060  -  Unknown protein
## VIT_09s0002g01200  -  Unknown protein
## VIT_01s0026g01420  -  Wall-associated kinase 4
## VIT_09s0070g00560  -  EIX receptor
## VIT_04s0008g05120  -  Phospholipase/carboxylesterase
## VIT_00s0253g00060  -  OBF binding protein 2
## VIT_12s0034g02470  -  Disease resistance protein (CC-NBS-LRR class)
## VIT_12s0028g02200  -  Riboflavin kinase
## VIT_18s0117g00050  -  R protein L6
## VIT_11s0052g00490  -  No hit
## VIT_16s0013g01990  -  CLL1B clavata1-like receptor S/T protein kinase
## VIT_04s0044g01870  -  Auxin efflux carrier
## VIT_13s0074g00110  -  Bark storage protein A precursor
## VIT_18s0001g13460  -  putative MADS-box Apetala  3a (VviAP3a)
## VIT_13s0139g00220  -  R protein MLA10
## VIT_05s0062g00700  -  UDP-glucose:flavonoid 7-O-glucosyltransferase
## VIT_03s0091g01120  -  Meprin and TRAF homology domain-containing protein
## VIT_19s0015g01680  -  Unknown protein
## VIT_18s0001g11320  -  Ent-kaurene oxidase
## VIT_00s0587g00010  -  Replication factor A 1, rfa1
## VIT_01s0011g04980  -  Sulfate transporter 91
## VIT_02s0025g02190  -  Short-chain dehydrogenase/reductase
## VIT_13s0064g01290  -  basic helix-loop-helix (bHLH) family
## VIT_18s0117g00040  -  No hit
## VIT_07s0095g00760  -  Methionyl-tRNA synthetase
## VIT_11s0052g01550  -  MATE efflux family protein
## VIT_05s0049g01810  -  No hit
## VIT_13s0067g03580  -  No hit
## VIT_09s0002g04080  -  IAA9
## VIT_19s0085g00950  -  NAC domain-containing protein (VvNAC69)
## VIT_01s0010g03060  -  Unknown protein
## VIT_07s0005g02080  -  No hit
## VIT_08s0040g01140  -  Serine carboxypeptidase K10B2.2 precursor
## VIT_00s0194g00080  -  Phototropic-responsive NPH3
## VIT_09s0002g04410  -  No hit
## VIT_10s0003g02440  -  Xyloglucan endotransglucosylase/hydrolase 23
## VIT_07s0129g00910  -  Protein kinase
## VIT_13s0067g02400  -  Myosin heavy chain
## VIT_08s0007g08910  -  Cis-zeatin O-beta-D-glucosyltransferase
## VIT_18s0001g12620  -  U-box domain-containing protein
## VIT_19s0014g04720  -  Unknown protein
## VIT_12s0057g00200  -  Vesicle-associated membrane protein
## VIT_03s0038g04160  -  DC1 domain-containing protein
## VIT_08s0007g02440  -  Aspartyl protease
## VIT_04s0008g01740  -  Spermine synthase ACAULIS5
## VIT_11s0016g02860  -  Nudix hydrolase 8
```

```
## VIT_10s0003g05130  -  Wall-associated receptor kinase 5
## VIT_14s0066g00870  -  ATPase-like domain-containing
## VIT_01s0026g00300  -  Zinc finger (C3HC4-type ring finger)
## VIT_04s0008g03640  -  FAD-binding domain-containing protein
## VIT_08s0007g06310  -  myb family
## VIT_06s0004g07650  -  Taxadien-5-alpha-ol-O-acetyltransferase
## VIT_13s0019g02640  -  Subtilisin protease C1
## VIT_10s0003g04670  -  Homeobox-leucine zipper transcription factor PHABULOSA
## VIT_05s0077g02100  -  Senescence-associated protein
## VIT_13s0064g00410  -  R protein MLA10
## VIT_14s0108g01670  -  Ac-CoA carboxylase
## VIT_09s0018g00310  -  ABC Transporter (VvMRP7 - VvABCC7)
## VIT_07s0129g01090  -  ADP-ribosylation factor
## VIT_13s0019g04880  -  No hit
## VIT_03s0038g04620  -  Isoflavone reductase (synthesis of phytoalexines)
## VIT_17s0000g09310  -  Unknown protein
## VIT_18s0001g12610  -  Radialis-like protein 6
## VIT_08s0007g06960  -  Disease resistance-responsive
## VIT_03s0091g00210  -  Ethylene-responsive protein
## VIT_02s0025g00970  -  ABC Transporter (VvMRP14 - VvABCC14)
## VIT_19s0015g01150  -  Kinesin 4 motor protein
## VIT_05s0020g03760  -  RNA helicase SDE3 (SDE3)
## VIT_15s0045g00440  -  Disease resistance protein (TIR-NBS-LRR class)
## VIT_05s0077g01410  -  No hit
## VIT_01s0011g05450  -  Unknown protein
## VIT_09s0002g00810  -  S-2-hydroxy-acid oxidase, peroxisomal
## VIT_09s0002g03520  -  Arabidopsis histidine phosphotransfer AHP4
## VIT_04s0008g04950  -  TIFY gene family (VvTIFY2)
## VIT_01s0011g05600  -  Receptor-like protein kinase
## VIT_04s0008g03990  -  Unknown
## VIT_00s0780g00040  -  RGLG2 (ring Domain LIGASE2)
## VIT_13s0064g00240  -  No hit
## VIT_04s0044g00310  -  Nodulin MtN21 family
## VIT_04s0008g04000  -  Dehydration-responsive protein (RD22)
## VIT_05s0077g01750  -  SAG101 (senescence-associated gene 101)
## VIT_03s0038g02010  -  Amidase
## VIT_07s0005g02050  -  Carboxylic ester hydrolase
## VIT_01s0011g01300  -  Polygalacturonase QRT3
## VIT_10s0071g00590  -  GRAM domain-containing protein / ABA-responsive
## VIT_08s0040g03170  -  No hit
## VIT_18s0001g04910  -  Sulfate transporter 1.3
## VIT_06s0004g05070  -  Zinc transporter (ZIP2)
## VIT_00s0181g00070  -  ARV2
```

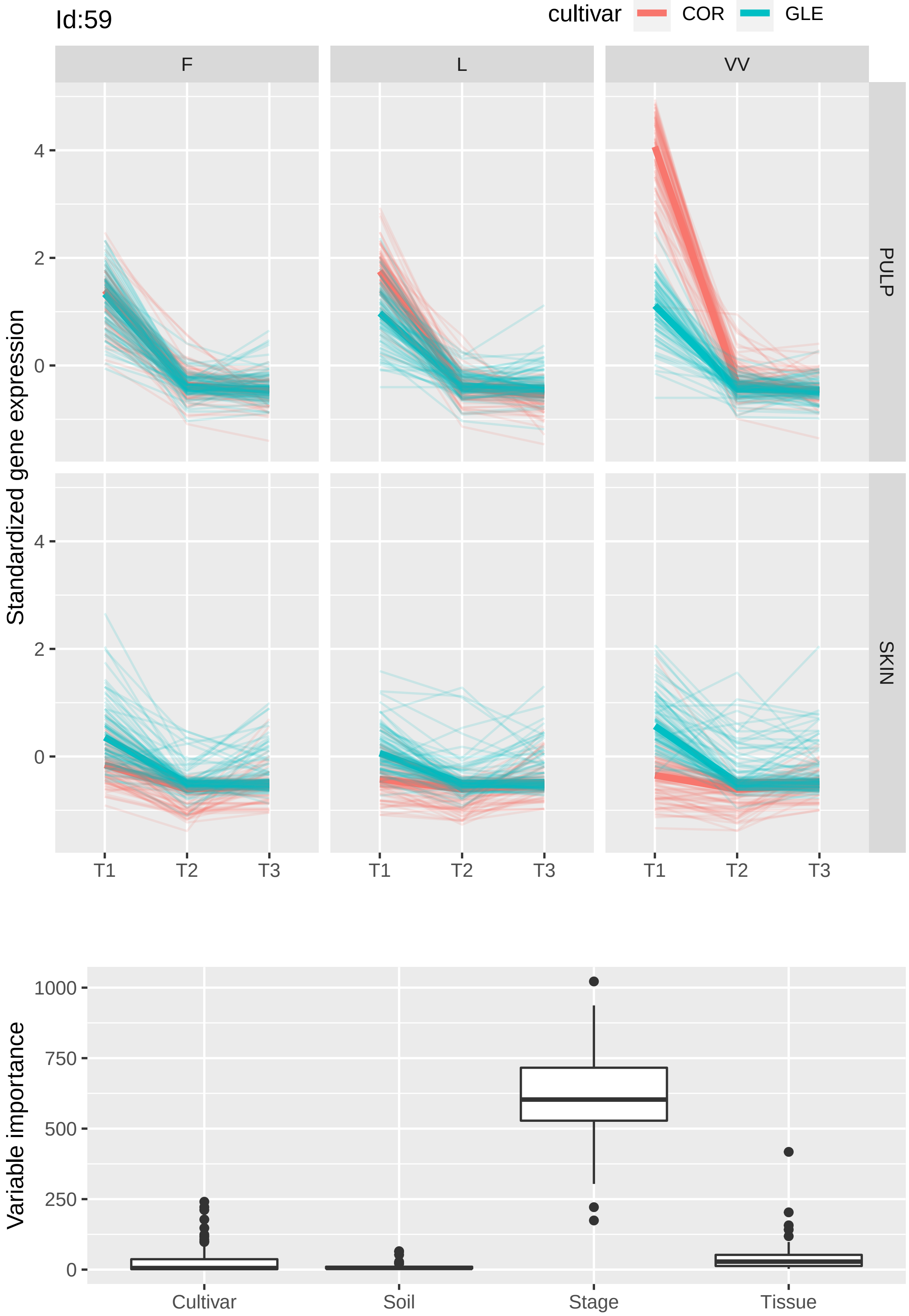
Id:59
cultivar
COR
GLE
F
L
VV
PULP
SKIN
Standardized gene expression
4
2
0
T1
T2
T3
Variable importance
1000
750
500
250
0
Cultivar
Soil
Stage
Tissue

Cluster no. 25

```
## Number of genes in the cluster: 63
## Homogeneity Index:    0.83
## Variable importance for Stage:     Rank =  25  - Median =  595.7
## Variable importance for Cultivar:  Rank =  22  - Median =  186.9
## Variable importance for Tissue:    Rank =  59  - Median =  83.34
## Variable importance for Soil:      Rank =  42  - Median =   5.87
##
## Gene ID                Gene Annotation
## VIT_16s0098g00770  -  Auxin-amidohydrolase precursor
## VIT_12s0134g00510  -  Cytochrome B6-F complex iron-sulfur subunit, PETC
## VIT_05s0094g00470  -  Zinc finger (C3HC4-type ring finger)
## VIT_18s0001g01490  -  Oxidoreductase N-terminal domain-containing
## VIT_16s0050g02480  -  ABC transporter C member 15 (VvABCC1)
## VIT_13s0064g00370  -  Alpha-N-acetylglucosaminidase
## VIT_09s0018g00400  -  Unknown protein
## VIT_08s0007g04530  -  carotene hydroxylase (CYP97C1; LUT1) (VvLUT1)
## VIT_16s0100g00310  -  Unknown protein
## VIT_15s0048g01010  -  2'-hydroxy isoflavone/dihydroflavonol reductase
## VIT_11s0052g01390  -  Adenosylmethionine-8-amino-7- oxononanoate aminotransferase
## VIT_17s0000g00930  -  Unknown protein
## VIT_19s0014g04980  -  Cinnamyl alcohol dehydrogenase
## VIT_18s0041g00670  -  Proton-dependent oligopeptide transport (POT) family protein
## VIT_19s0014g03770  -  Retrotransposon
## VIT_16s0050g01000  -  Unknown protein
## VIT_06s0004g04110  -  Unknown protein
## VIT_12s0059g00220  -  Epoxide hydrolase
## VIT_09s0054g01110  -  Cycloartenol synthase
## VIT_19s0015g02380  -  Ankyrin repeat
## VIT_13s0067g00810  -  R protein  PRF  disease resistance protein
## VIT_19s0014g03780  -  Unknown protein
## VIT_19s0015g01250  -  No hit
## VIT_13s0073g00160  -  Unknown protein
## VIT_16s0039g00990  -  Glutathione S-transferase 8 GSTU8
## VIT_08s0056g01290  -  Receptor-like kinase 17
## VIT_06s0080g00320  -  Esterase/lipase/thioesterase
## VIT_12s0057g01260  -  Unknown protein
## VIT_01s0011g04750  -  Unknown
## VIT_16s0050g00170  -  HcrVf3 protein
## VIT_08s0007g01040  -  Aldo-keto reductase
## VIT_15s0021g02370  -  Unknown protein
## VIT_01s0011g06460  -  Deoxymugineic acid synthase
## VIT_14s0030g02240  -  Carbon-sulfur lyase
## VIT_19s0015g02130  -  Ankyrin repeat
## VIT_03s0038g03410  -  NAC domain-containing protein (VvNAC41)
## VIT_19s0015g00620  -  Lectin protein kinase
## VIT_08s0007g01610  -  KOW domain-containing transcription factor family protein
## VIT_00s0181g00020  -  Armadillo/beta-catenin repeat
## VIT_13s0067g01950  -  Chromatin remodeling 42
## VIT_01s0011g01140  -  Cationic amino acid transporter 5
## VIT_18s0041g02410  -  Aldehyde oxidase 1
## VIT_01s0011g01120  -  R protein MLA10
## VIT_01s0011g01860  -  No hit
## VIT_08s0007g08050  -  Carboxyl-terminal processing protease
## VIT_00s0848g00030  -  Somatic embryogenesis receptor kinase 2 SERK2
```

```
## VIT_05s0094g01150  -  Allyl alcohol dehydrogenase
## VIT_04s0008g02670  -  Cryptochrome DASH
## VIT_05s0094g01190  -  flavonoid 3-monooxygenase
## VIT_00s0304g00070  -  Lipid-associated family protein
## VIT_06s0004g07390  -  Calcineurin phosphoesterase
## VIT_07s0031g01640  -  ABC Transporter (VvGCN3 - VvABCF3)
## VIT_19s0014g04990  -  No hit
## VIT_18s0001g08750  -  Unknown
## VIT_13s0139g00130  -  R protein MLA10
## VIT_03s0017g01490  -  Berberine bridge enzyme
## VIT_08s0007g01590  -  fructose 1,6-bisphosphatase
## VIT_05s0077g02050  -  Gamete expressed2 (GEX2)
## VIT_18s0001g00290  -  OPCL1 (OPC-8:0 COA ligase1)
## VIT_14s0036g00010  -  R protein  PRF  disease resistance protein
## VIT_05s0049g01130  -  Aldo/keto reductase
## VIT_16s0115g00310  -  Ribosomal protein L35
## VIT_15s0048g02550  -  Pentatricopeptide (PPR) repeat
```

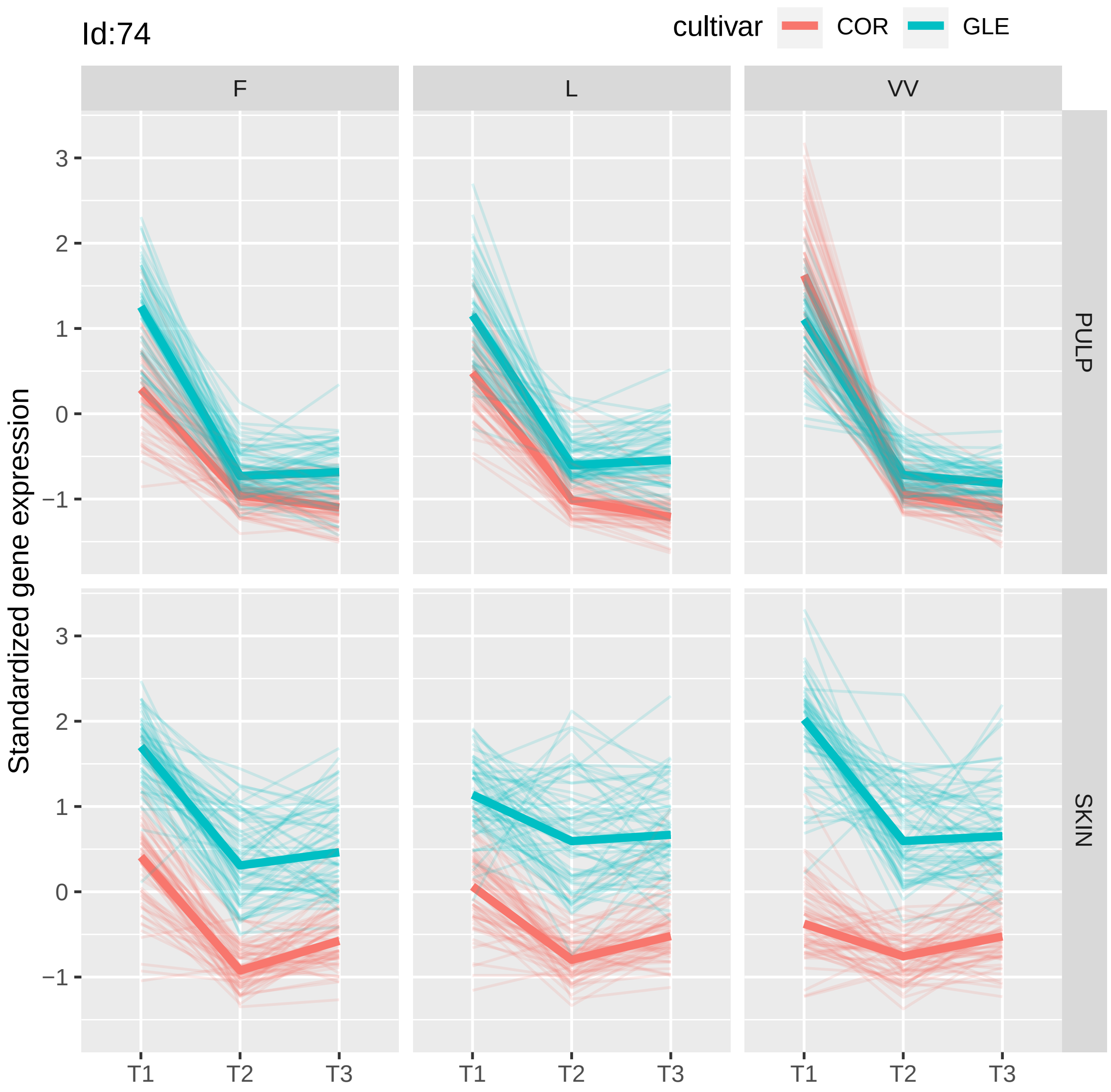

Id:74
cultivar
COR
GLE
F
L
VV
PULP
SKIN
Standardized gene expression
3
2
1
0
−1
T1
T2
T3

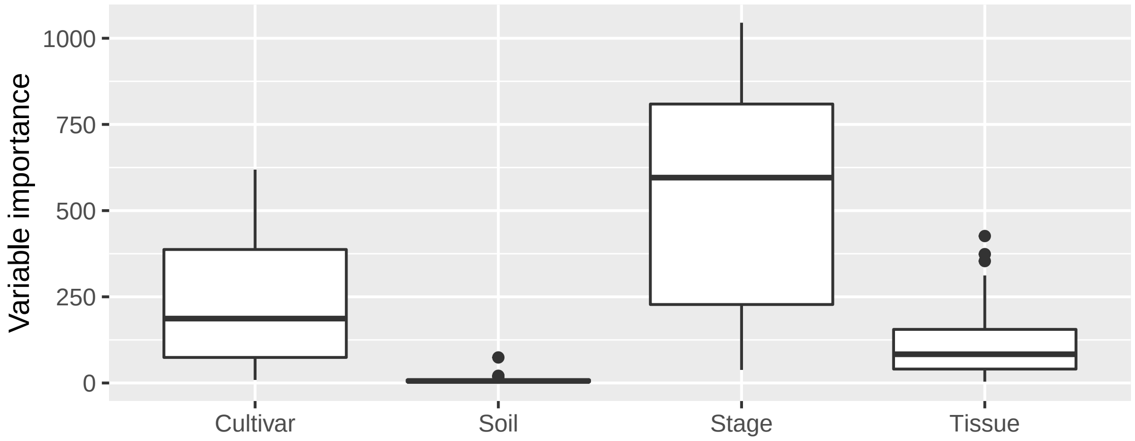

Variable importance
1000
750
500
250
0
Cultivar
Soil
Stage
Tissue

Cluster no. 26

```
## Number of genes in the cluster: 52
## Homogeneity Index:    0.87
## Variable importance for Stage:     Rank =  26  - Median =  581.5
## Variable importance for Cultivar:  Rank =  90  - Median =   8.39
## Variable importance for Tissue:    Rank =  67  - Median =  41.36
## Variable importance for Soil:      Rank =  43  - Median =   5.84
##
## Gene ID               Gene Annotation
## VIT_08s0007g06250  -  Strubbelig receptor family 2
## VIT_09s0070g00080  -  Aquaporin NIP6;1
## VIT_08s0007g07000  -  Disease resistance-responsive
## VIT_17s0000g08760  -  Receptor kinase TRKa
## VIT_02s0012g01140  -  Protein kinase
## VIT_01s0011g03050  -  Unknown protein
## VIT_13s0067g00220  -  Aquaporin TMP-C
## VIT_04s0008g04710  -  Beta-ketoacyl-CoA synthase
## VIT_17s0000g05830  -  Unknown protein
## VIT_04s0008g07140  -  Chloroplast nucleoid DNA binding protein
## VIT_08s0007g04510  -  RPG related protein 1 RR1
## VIT_08s0007g06950  -  Disease resistance-responsive
## VIT_04s0008g00420  -  Clavata1 receptor kinase (CLV1)
## VIT_09s0054g00820  -  Unknown protein
## VIT_11s0052g00240  -  Accelerated cell death 11 (ACD11)
## VIT_11s0037g01000  -  Clathrin assembly protein 10
## VIT_01s0011g06610  -  Glutamate decarboxylase
## VIT_03s0038g02800  -  Cyclin B2;4
## VIT_00s1365g00010  -  fimbrin 1
## VIT_18s0122g00850  -  Tonoplast monosaccharide transporter2
## VIT_03s0091g01010  -  LIM domain protein WLIM1
## VIT_19s0014g00510  -  Receptor kinase 2
## VIT_18s0001g14040  -  Endo-1,4-beta-glucanase
## VIT_14s0066g00610  -  Unknown protein
## ENSRNA049995874  -
## VIT_15s0046g00290  -  Auxin response factor 18
## VIT_01s0026g00180  -  Serine/threonine kinase protein
## VIT_18s0001g08500  -  Lipase family
## VIT_06s0080g00660  -  No hit
## VIT_15s0048g01210  -  Subtilisin serine endopeptidase (XSP1)
## VIT_07s0031g00940  -  Sulfate transporter 2.1 (AST68)
## VIT_03s0038g04000  -  Cysteine endopeptidase, papain-type (XCP1)
## VIT_15s0048g01200  -  Subtilisin serine endopeptidase (XSP1)
## VIT_12s0057g00210  -  No hit
## VIT_11s0016g03710  -  Abl interactor protein 1 (ABIL1)
## VIT_00s0804g00020  -  Unknown protein
## VIT_00s1373g00020  -  Unknown protein
## VIT_00s2512g00010  -  No hit
## VIT_19s0014g02740  -  Metallothionein
## VIT_15s0048g01000  -  2'-hydroxy isoflavone/dihydroflavonol reductase
## VIT_02s0025g03540  -  Tubulin beta-6 chain
## VIT_14s0068g00510  -  Unknown
## VIT_05s0020g00420  -  Polygalacturonase GH28
## VIT_00s0945g00010  -  Unknown protein
## VIT_04s0008g06530  -  formin-2
## VIT_12s0134g00340  -  S-locus lectin protein kinase
```

```
## VIT_09s0002g02460  -  Phosphatase
## VIT_02s0025g04210  -  UDP-glucose 4-epimerase
## VIT_06s0004g01870  -  No transmitting tract
## VIT_04s0079g00290  -  Regulator of chromosome condensation (RCC1)
## VIT_00s0941g00020  -  Constans interacting protein 6
## VIT_15s0024g01490  -  Callose synthase catalytic subunit
```

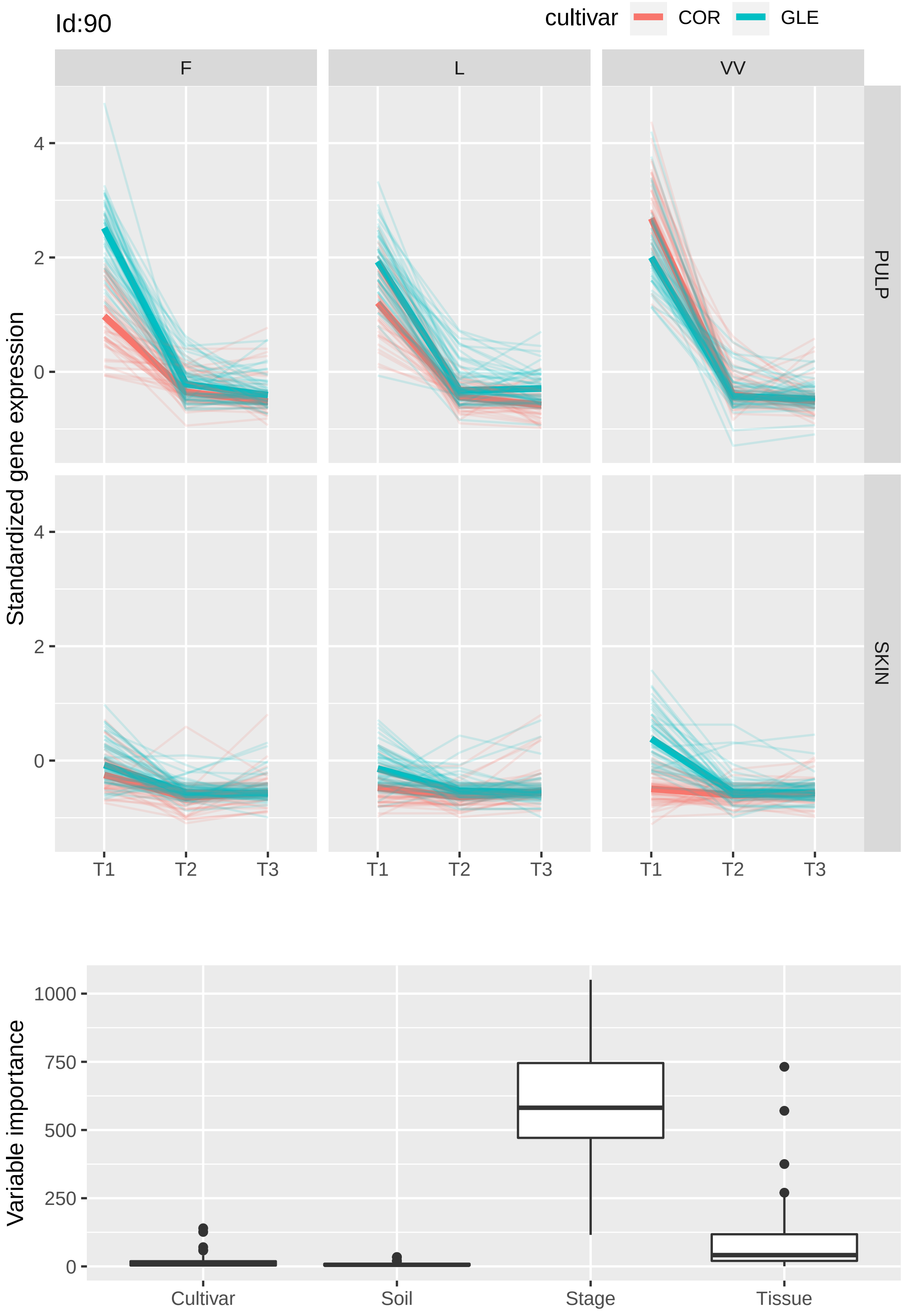

Id:90
cultivar
COR
GLE
F
L
VV
PULP
SKIN
Standardized gene expression
4
2
0
T1
T2
T3
Variable importance
1000
750
500
250
0
Cultivar
Soil
Stage
Tissue

Cluster no. 27

```
## Number of genes in the cluster: 84
## Homogeneity Index:     0.8
## Variable importance for Stage:     Rank =  27  - Median =  579.3
## Variable importance for Cultivar:  Rank =  75  - Median =  12.68
## Variable importance for Tissue:    Rank =  46  - Median =  141.7
## Variable importance for Soil:      Rank =  16  - Median =   8.38
##
## Gene ID                Gene Annotation
## VIT_10s0003g00180  -  Drought induced 19 protein
## VIT_03s0038g02970  -  Ataxia telangiectasia and Rad3 related
## VIT_01s0026g01660  -  Alpha-amylase isozyme C2 precursor
## VIT_01s0011g04210  -  Amino acid permease
## VIT_15s0048g00190  -  SPX (SYG1/Pho81/XPR1) domain-containing protein
## VIT_16s0050g01110  -  Polygalacturonase GH28
## VIT_10s0003g00350  -  NAC domain-containing protein (VvNAC37)
## VIT_14s0081g00160  -  Myosin-like protein XIC
## VIT_07s0205g00040  -  Unknown protein
## VIT_18s0089g01260  -  Pentatricopeptide repeat
## VIT_18s0001g02480  -  Unknown protein
## VIT_00s1542g00010  -  Receptor protein kinase
## VIT_01s0011g01530  -  No hit
## VIT_05s0020g04680  -  Auxin-induced protein 22D
## VIT_18s0072g00850  -  No hit
## VIT_00s0313g00020  -  Mechanosensitive ion channel MSCS-like 2
## VIT_12s0057g00250  -  NDB3 (alternative NAD(P)H dehydrogenase 32)
## VIT_01s0011g01810  -  No hit
## VIT_05s0020g04670  -  No hit
## VIT_08s0007g04350  -  Protein kinase
## VIT_01s0011g04040  -  No hit
## VIT_14s0219g00010  -  Protein phosphatase 2 (formerly 2A), regulatory subunit B'
## VIT_19s0014g02380  -  No hit
## VIT_04s0023g02610  -  Epoxide hydrolase 2
## VIT_09s0002g04690  -  Pentatricopeptide (PPR) repeat-containing protein
## VIT_14s0171g00530  -  Protein phosphatase 2 (formerly 2A), regulatory subunit B'
## VIT_09s0054g01060  -  Tassel serine threonine kinase 1
## VIT_04s0008g05610  -  Centromeric protein
## VIT_00s1542g00020  -  Pto serine/threonine kinase
## VIT_11s0118g00260  -  Multimeric translocon complex in the outer envelope membrane 132 TOC13
## VIT_05s0049g01200  -  S-locus lectin protein kinase
## VIT_19s0014g02450  -  ALF5 (Aberrant lateral root formation 5)
## VIT_18s0001g02760  -  Lipase GDSL
## VIT_10s0003g01540  -  Pentatricopeptide (PPR) repeat-containing protein
## VIT_00s0207g00040  -  No hit
## VIT_08s0056g00250  -  Harpin-induced 1
## VIT_03s0038g03400  -  Endochitinase 1, basic
## VIT_13s0067g02120  -  Arogenate dehydrogenase
## VIT_12s0059g01380  -  1-aminocyclopropane-1-carboxylate oxidase 1
## VIT_12s0028g02330  -  No hit
## VIT_12s0057g01000  -  Unknown protein
## VIT_17s0000g03340  -  Wall-associated kinase 4
## VIT_04s0008g05700  -  ACT domain-containing protein
## VIT_14s0006g00860  -  Valine--tRNA ligase
## VIT_18s0089g00160  -  1,4-beta-mannan endohydrolase
## VIT_04s0044g01560  -  Protein kinase
```

```
## VIT_01s0011g03200  -  Transmembrane protein FT27/PFT27
## VIT_02s0025g04300  -  Thaumatin
## VIT_01s0011g01100  -  Pentatricopeptide (PPR) repeat-containing protein
## VIT_18s0001g11130  -  Calmodulin-binding protein AR781
## VIT_17s0000g07350  -  Exocyst subunit EXO70 E2
## VIT_01s0011g02320  -  Unknown protein
## ENSRNA049470021  -
## VIT_19s0014g00620  -  Receptor kinase 2
## VIT_19s0090g01070  -  Glucan endo-1,3-beta-glucosidase 7 precursor
## VIT_04s0008g04420  -  ADP-ribosylation factor GTPase activating protein
## VIT_05s0020g03140  -  Sugar transporter 13
## VIT_10s0003g05430  -  Reticuline oxidase precursor
## VIT_14s0060g00650  -  No hit
## VIT_17s0000g03600  -  ATSYTE/NTMC2T3.1/NTMC2TYPE3.1/SYTE
## VIT_17s0000g09380  -  WNK kinase 3
## VIT_00s0640g00010  -  Transcriptional corepressor LEUNIG
## VIT_01s0011g05150  -  Bet v I allergen
## VIT_02s0236g00120  -  Unknown
## VIT_09s0002g07640  -  Glycerol kinase
## VIT_00s2381g00010  -  CC-NBS-LRR class
## VIT_14s0030g01610  -  Cytidine/deoxycytidylate deaminase
## VIT_01s0010g02200  -  Zinc finger (C3HC4-type ring finger)
## VIT_18s0122g00690  -  Laccase
## VIT_02s0025g04310  -  Thaumatin
## VIT_07s0005g02420  -  Haloacid dehalogenase hydrolase
## VIT_09s0002g05490  -  ABC Transporter (VvPDR12 - VvABCG42)
## VIT_12s0034g01820  -  Pentatricopeptide repeat-containing
## VIT_14s0006g00420  -  Unknown protein
## VIT_17s0000g06930  -  Unfertilized embryo sac 10 UNE10
## VIT_08s0007g01050  -  Aldo-keto reductase
## VIT_11s0118g00090  -  Leucine-rich repeat family protein
## VIT_01s0011g05880  -  RDR1 (RNA-dependent RNA polymerase 1)
## VIT_04s0008g05930  -  Zinc finger protein 3
## VIT_18s0001g11580  -  CYP82A3
## VIT_18s0001g08760  -  No hit
## VIT_16s0098g01220  -  RabGAP/TBC domain-containing protein
## VIT_09s0002g06940  -  ABC transporter g family pleiotropic drug resistance 12 PDR12
## VIT_18s0072g00840  -  Sensitive to proton rhizotoxicity 1
```

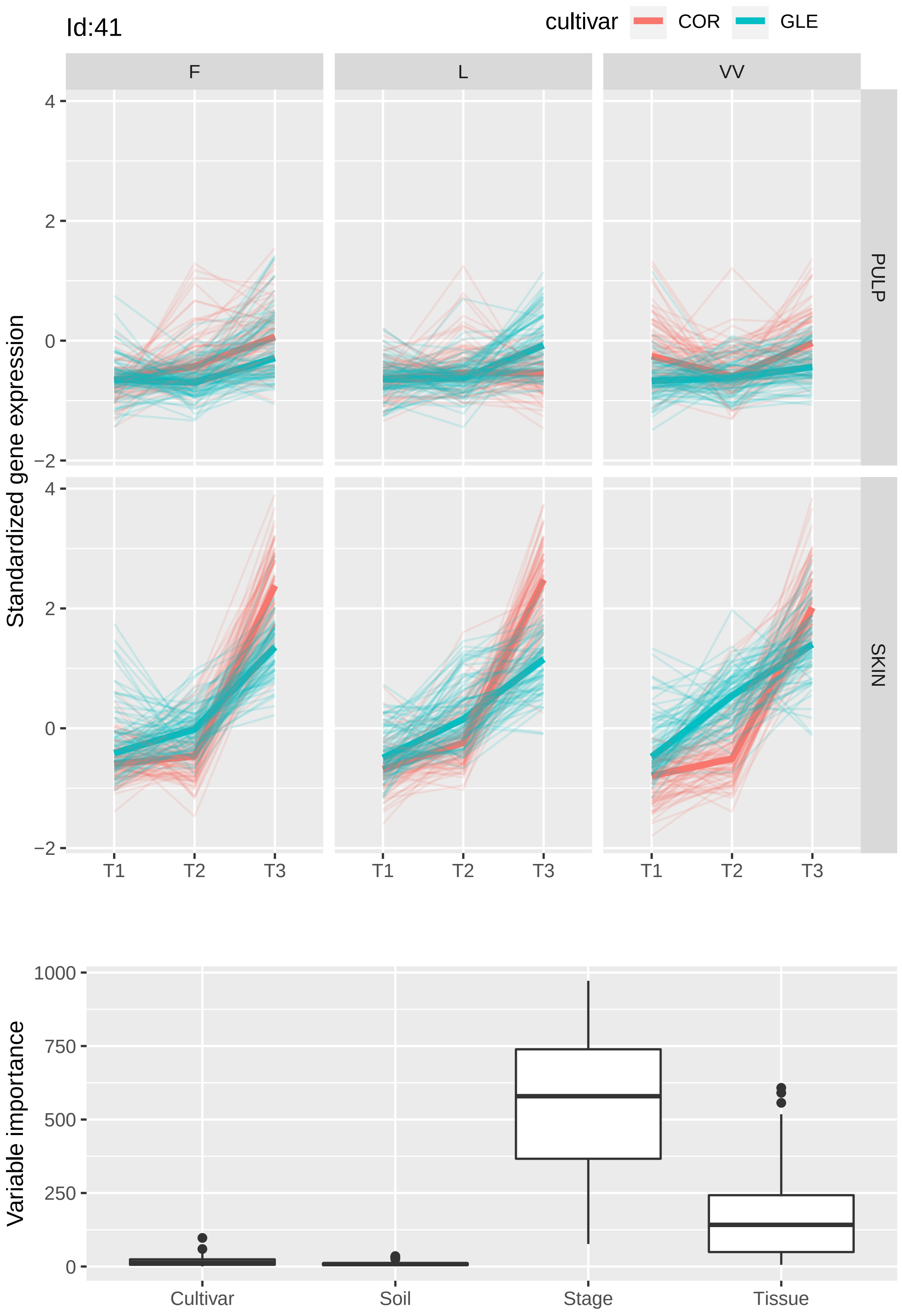
Id:41
cultivar
COR
GLE
F
L
VV
PULP
SKIN
Standardized gene expression
4
2
0
−2
T1
T2
T3
Variable importance
1000
750
500
250
0
Cultivar
Soil
Stage
Tissue

Cluster no. 28

```
## Number of genes in the cluster: 142
## Homogeneity Index:    0.77
## Variable importance for Stage:     Rank =  28  - Median =  543.7
## Variable importance for Cultivar:  Rank =  25  - Median =  155.9
## Variable importance for Tissue:    Rank =  93  - Median =  13.38
## Variable importance for Soil:      Rank =  15  - Median =   8.48
##
## Gene ID                Gene Annotation
## VIT_19s0090g00590  -  myb domain protein 101
## VIT_00s0203g00180  -  Unknown protein
## VIT_14s0068g01360  -  GEM-like protein 5
## VIT_12s0059g01550  -  Triacylglycerol/steryl ester lipase
## VIT_00s0375g00040  -  NAC domain-containing protein (VvNAC03)
## VIT_16s0050g02260  -  Unknown protein
## VIT_17s0000g05110  -  CYP78A4
## VIT_06s0004g07190  -  Unknown protein
## VIT_14s0108g00800  -  No hit
## VIT_05s0094g00340  -  Chitinase class IV
## VIT_18s0001g11910  -  1-acyl-sn-glycerol-3-phosphate acyltransferase 4
## VIT_05s0062g00980  -  Aldo/keto reductase AKR
## VIT_08s0007g01650  -  Auxin-independent growth promoter
## VIT_08s0007g05340  -  Kinesin motor protein
## VIT_04s0044g01080  -  Pentatricopeptide (PPR) repeat
## VIT_01s0011g05810  -  Adagio protein 1
## VIT_01s0011g05980  -  No hit
## VIT_00s0225g00230  -  Alliin lyase precursor
## VIT_11s0052g00580  -  No hit
## VIT_05s0049g00750  -  No hit
## VIT_17s0000g01340  -  Unknown
## VIT_11s0118g00560  -  Nodulin
## VIT_15s0046g00470  -  Dynamin-like protein 2b
## VIT_19s0015g01890  -  BRL3 (BRI1-like 3)
## VIT_10s0003g03790  -  Jasmonate ZIM domain-containing protein (VvJAZ5)
## VIT_00s0125g00060  -  Unknown
## VIT_00s0233g00030  -  Trehalose-6-phosphate phosphatase
## VIT_18s0001g11610  -  No hit
## VIT_02s0033g01400  -  Alternative oxidase 1a, (AOX1A)
## VIT_09s0002g00750  -  ABC Transporter (VvMDR9 - VvABCB9)
## VIT_17s0000g06380  -  YGL010w
## VIT_06s0009g01770  -  Lipase GDSL
## VIT_02s0033g01390  -  No hit
## VIT_03s0038g00660  -  Unknown protein
## VIT_05s0094g00330  -  Chitinase, class IV [Vitis vinifera]
## VIT_19s0093g00460  -  No hit
## VIT_03s0063g00620  -  Transcriptional factor B3
## VIT_07s0151g01080  -  Unknown protein
## VIT_09s0002g06130  -  Zinc finger (CCCH-type) family protein
## VIT_10s0003g00430  -  Unknown protein
## VIT_18s0001g11930  -  Thaumatin SCUTL2
## VIT_14s0171g00140  -  Lys Motif-Type Receptor-Like Kinase LYK10
## ENSRNA049467585  -
## VIT_04s0008g01680  -  EMB2745
## VIT_05s0049g00060  -  Oxidative-stress responsive 1
## VIT_05s0124g00520  -  No hit
```

```
## VIT_14s0066g00880  -  Helicase RCK (rock-n-rollers)
## VIT_13s0019g00800  -  Histone H4
## VIT_19s0014g02440  -  MATE efflux family protein
## VIT_04s0069g00440  -  Nuclear pore complex protein Nup93
## VIT_19s0090g00040  -  Unknown protein
## VIT_09s0002g04290  -  Hydroxyphenylpyruvate reductase (HPPR)
## VIT_19s0014g02430  -  Aberrant lateral root formation 5
## VIT_18s0001g08990  -  No hit
## VIT_01s0150g00070  -  SUVH4 (SU(VAR)3-9 homolog 4)
## VIT_08s0040g02530  -  No hit
## ENSRNA049468587  -
## VIT_04s0069g01010  -  Harpin-induced protein
## VIT_17s0000g02870  -  S-adenosyl-L-methionine:salicylic acid carboxyl methyltransferase
## VIT_01s0011g03440  -  DNA mismatch repair protein
## VIT_13s0067g02530  -  Unknown protein
## VIT_08s0040g02520  -  Inositol-1,4,5-trisphosphate 5-phosphatase CVP2, type I
## VIT_14s0006g01370  -  VTC2
## VIT_10s0116g01510  -  DNA helicase SNF2 domain-containing protein
## VIT_14s0171g00410  -  Ankyrin repeat
## VIT_06s0004g08420  -  ATP-dependent DNA helicase recQ
## VIT_11s0016g03490  -  Glyoxal oxidase
## VIT_17s0000g03400  -  Unknown protein
## VIT_00s0340g00050  -  Endo-1,4-beta-glucanase korrigan (KOR)
## VIT_09s0002g02940  -  Myo-inositol oxygenase 1
## VIT_00s0340g00060  -  Endo-1,4-beta-glucanase korrigan (KOR)
## VIT_19s0015g01310  -  Amino acid permease 7
## VIT_03s0063g01990  -  Ternary complex factor MIP1
## VIT_12s0055g01240  -  P-glycoprotein 11
## VIT_12s0035g01050  -  Octicosapeptide/Phox/Bem1p (PB1) domain-containing protein
## VIT_05s0029g00520  -  Galacturonosyltransferase 1
## VIT_05s0051g00490  -  Outer membrane protein
## VIT_04s0023g02200  -  S-adenosyl-L-methionine:salicylic acid carboxyl methyltransferase
## ENSRNA049469269  -
## VIT_04s0023g03590  -  Unknown
## VIT_05s0020g03190  -  Haloacid dehalogenase hydrolase
## VIT_06s0004g00130  -  Pyruvate kinase
## VIT_17s0000g04740  -  Transducin family protein / WD-40 repeat
## VIT_14s0060g00350  -  Aspartic Protease (VvAP35)
## VIT_17s0000g03480  -  No hit
## VIT_07s0005g04490  -  Transducin protein
## VIT_18s0001g11200  -  Unknown
## VIT_07s0005g01430  -  PROLIFERA protein
## VIT_11s0052g01320  -  Xyloglucan endotransglycosylase 6
## VIT_05s0029g00530  -  Poly(A) binding protein
## VIT_06s0004g02310  -  No hit
## VIT_19s0090g00600  -  No hit
## VIT_12s0142g00280  -  Cell division cyle protein 45
## VIT_11s0052g01200  -  Xyloglucan endotransglucosylase/hydrolase 23
## VIT_01s0146g00440  -  Unknown protein
## VIT_19s0014g02500  -  Dynamin family protein
## VIT_13s0019g04110  -  Retrotransposon protein
## VIT_04s0008g03000  -  Cycloartenol synthase
## VIT_19s0014g00410  -  PMR5 (powdery mildew resistant 5)
## VIT_00s0125g00220  -  Serine/theronine protein kinase 2
```

```
## VIT_01s0244g00130  -  Inducer of CBF expression 1 ICE1
## VIT_01s0011g01040  -  RPM1 (resistance to p. syringae pv maculicola 1)
## VIT_13s0156g00030  -  No hit
## VIT_07s0031g01260  -  No hit
## VIT_12s0035g00920  -  Unknown protein
## VIT_04s0008g02990  -  Cycloartenol synthase
## VIT_19s0014g03090  -  CBS domain-containing protein
## VIT_04s0023g01980  -  Syntaxin 1B/2/3/4
## VIT_16s0098g01790  -  Calmodulin-binding region IQD21
## VIT_12s0035g02190  -  MLO-like protein 13
## VIT_03s0038g03740  -  Pentatricopeptide (PPR) repeat-containing
## VIT_13s0067g00890  -  Phosphoinositide-specific phospholipase C
## VIT_02s0154g00110  -  Trehalose-6-phosphate phosphatase (AtTPPA)
## VIT_04s0044g01490  -  2-oxoisovalerate dehydrogenase alpha subunit, mitochondrial precursor
## VIT_13s0106g00340  -  No hit
## VIT_00s0125g00210  -  Serine/theronine protein kinase 2
## VIT_05s0020g04950  -  CwfJ
## VIT_11s0052g01180  -  Xyloglucan endotransglucosylase/hydrolase 23
## VIT_12s0035g00040  -  Splicing factor 3B subunit 3
## VIT_16s0013g01570  -  myb domain protein 92
## VIT_11s0052g01270  -  Xyloglucan endotransglycosylase 6
## VIT_04s0008g05980  -  APM1 (Aminopeptidase M1)
## VIT_18s0041g01680  -  No hit
## VIT_07s0104g00250  -  basic helix-loop-helix (bHLH) family
## VIT_11s0052g01260  -  Xyloglucan endotransglucosylase/hydrolase 23
## ENSRNA049469094  -
## VIT_03s0038g02610  -  Monooxygenase (MO2)
## ENSRNA049469046  -
## VIT_00s0125g00230  -  No hit
## VIT_15s0021g00540  -  Invertase/pectin methylesterase inhibitor
## VIT_11s0016g03920  -  Polyol transporter 5
## VIT_09s0002g03970  -  Unknown protein
## VIT_06s0004g04380  -  Unknown protein
## VIT_18s0001g08810  -  Unknown protein
## VIT_04s0069g00520  -  No hit
## VIT_15s0048g02480  -  Caffeate 3-O-methyltransferase 1
## VIT_00s2071g00010  -  Cis-prenyltransferase
## VIT_11s0078g00460  -  PUMILIO 5 (APUM5)
## VIT_19s0085g00450  -  No hit
## VIT_19s0085g00560  -  Pentatricopeptide (PPR) repeat-containing
## VIT_15s0048g02490  -  Catechol O-methyltransferase
## VIT_04s0023g02480  -  Dehydrin 1b
```

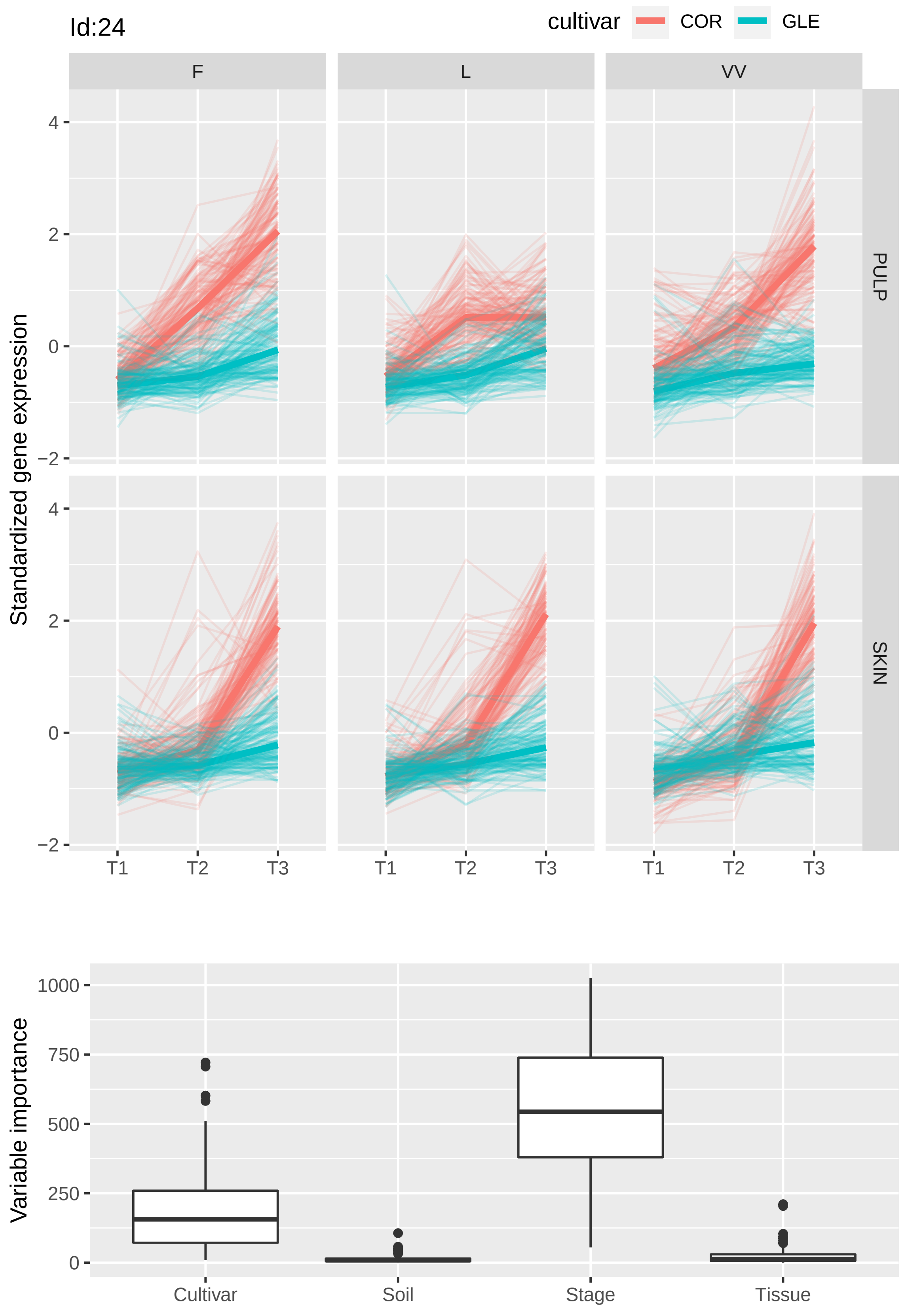
Id:24
cultivar
COR
GLE
F
L
VV
PULP
SKIN
Standardized gene expression
T1
T2
T3
Variable importance
Cultivar
Soil
Stage
Tissue

Cluster no. 29

```
## Number of genes in the cluster: 78
## Homogeneity Index:    0.88
## Variable importance for Stage:     Rank =  29  - Median =  541.7
## Variable importance for Cultivar:  Rank =  81  - Median =  11.16
## Variable importance for Tissue:    Rank =  37  - Median =    239
## Variable importance for Soil:      Rank =  98  - Median =   2.68
##
## Gene ID               Gene Annotation
## VIT_11s0052g01120  -  Zinc finger protein 2
## VIT_07s0129g00290  -  formamidase, putative / formamide amidohydrolase, putative
## VIT_07s0005g01660  -  Pentatricopeptide (PPR) repeat-containing protein
## VIT_01s0011g03550  -  Unknown
## VIT_11s0016g01430  -  Zinc finger (C3HC4-type ring finger)
## VIT_09s0054g00430  -  Unknown protein
## VIT_14s0108g00400  -  Glutaredoxin family
## VIT_19s0090g00830  -  Aspartic Protease (VvAP45)
## VIT_10s0116g00050  -  Receptor protein kinase
## VIT_04s0008g03050  -  Acetylglutamate kinase
## VIT_07s0129g00300  -  formamidase, putative / formamide amidohydrolase, putative
## VIT_06s0004g05280  -  Tropinone reductase
## VIT_00s0193g00110  -  Ankyrin repeat protein
## VIT_06s0009g02450  -  Integrase
## VIT_17s0000g01700  -  Leucine-rich repeat transmembrane protein kinase
## VIT_10s0116g00100  -  Strubbelig receptor family 8
## VIT_14s0030g00840  -  R protein disease resistance protein
## VIT_04s0069g00920  -  WRKY Transcription Factor (VvWRKY10)
## VIT_06s0004g00800  -  Amino acid permease
## VIT_04s0044g00150  -  Wuschel-related homeobox 13
## VIT_13s0019g00740  -  Amino acid binding protein
## VIT_17s0000g05020  -  Squamosa promoter-binding protein 6 (SPL6)
## VIT_07s0129g00310  -  Formamidase
## VIT_01s0010g01660  -  Receptor protein kinase
## VIT_02s0154g00070  -  Abnormal floral organs
## VIT_00s0978g00020  -  Unknown protein
## VIT_06s0061g01480  -  ABC Transporter (VvPDR21 - VvABCG51)
## VIT_06s0061g01560  -  PINOID
## VIT_11s0016g03010  -  Glutamate carboxypeptidase 2
## VIT_13s0074g00540  -  Lysine histidine transporter 1
## VIT_18s0001g05890  -  Dehydration Responsive Element-Binding Transcription Factor (VvDREB32)
## VIT_00s0214g00020  -  TORMOZembryo defective UTP13
## VIT_18s0001g11240  -  MAPKKK5 (Mitogen-activated protein kinase kinase kinase 5)
## VIT_05s0020g03130  -  Plastocyanin domain-containing protein
## VIT_12s0028g03060  -  HSL1 (HAESA-like 1)
## VIT_07s0031g00120  -  Leucine-rich repeat transmembrane protein kinase
## VIT_18s0001g08230  -  Receptor-like protein kinase HAIKU2
## VIT_15s0048g03000  -  GTPase RABA3
## VIT_10s0003g00050  -  Squamosa promoter-binding protein (VvSBP9)
## VIT_05s0049g00270  -  E8 protein
## VIT_18s0001g00820  -  Thioredoxin-like protein CDSP32
## VIT_08s0007g06270  -  Squamosa promoter-binding protein (VvSBP8)
## VIT_15s0048g01600  -  Geraniol 10-hydroxylase
## VIT_04s0008g01880  -  Cytokinin dehydrogenase 7
## VIT_14s0030g00870  -  CC-NBS-LRR class
## VIT_02s0025g03420  -  Unknown protein
```

```
## VIT_18s0001g15700  -  GLTP3 (glycolipid transfer protein 3)
## VIT_16s0022g00350  -  Receptor protein kinase
## VIT_02s0241g00050  -  Unknown protein
## VIT_03s0063g01690  -  flavonoid 3-monooxygenase
## VIT_15s0048g01880  -  Armadillo/beta-catenin repeat
## VIT_17s0000g09170  -  Receptor lectin kinase
## VIT_14s0083g01030  -  putative MADS-box Fruitfull  2 (VviFUL2)
## VIT_18s0001g08100  -  Retroelement pol polyprotein
## VIT_13s0067g01670  -  Ankyrin repeat protein
## VIT_03s0038g03180  -  flavin-containing monooxygenase 1
## VIT_00s0615g00020  -  Cinnamyl alcohol dehydrogenase
## VIT_09s0002g00490  -  Lipase GDSL 5
## VIT_14s0108g01660  -  Biotin carboxyl carrier protein of acetyl-CoA carboxylase
## VIT_10s0003g01600  -  WRKY Transcription Factor (VvWRKY30)
## VIT_10s0003g03270  -  Inward rectifying potassium channel
## VIT_14s0036g01350  -  R protein disease resistance protein
## VIT_15s0021g01060  -  CYP72A1
## VIT_15s0021g02110  -  Dehydration Responsive Element-Binding Transcription Factor (VvDREB21)
## VIT_06s0009g00580  -  Receptor protein kinase
## VIT_03s0063g02250  -  Polyol transporter 5
## VIT_10s0003g02810  -  WRKY Transcription Factor (VvWRKY31)
## VIT_19s0090g01410  -  Wax synthase isoform 1
## VIT_01s0127g00010  -  Carboxyl-terminal proteinase
## VIT_10s0003g04990  -  feronia receptor-like kinase
## VIT_05s0077g01090  -  RKF2 (receptor-like serine/threonine kinase 2)
## VIT_04s0023g01760  -  Unknown
## VIT_04s0043g00340  -  Myb KAN1 (KANADI 1)
## VIT_07s0031g01270  -  Ring-H2 finger A2A
## VIT_00s1361g00010  -  Unknown
## VIT_00s1213g00010  -  Cellulose synthase CSLE1
## VIT_08s0032g00050  -  Acyl-activating enzyme 15
## VIT_06s0061g01300  -  Prephenate dehydratase
```

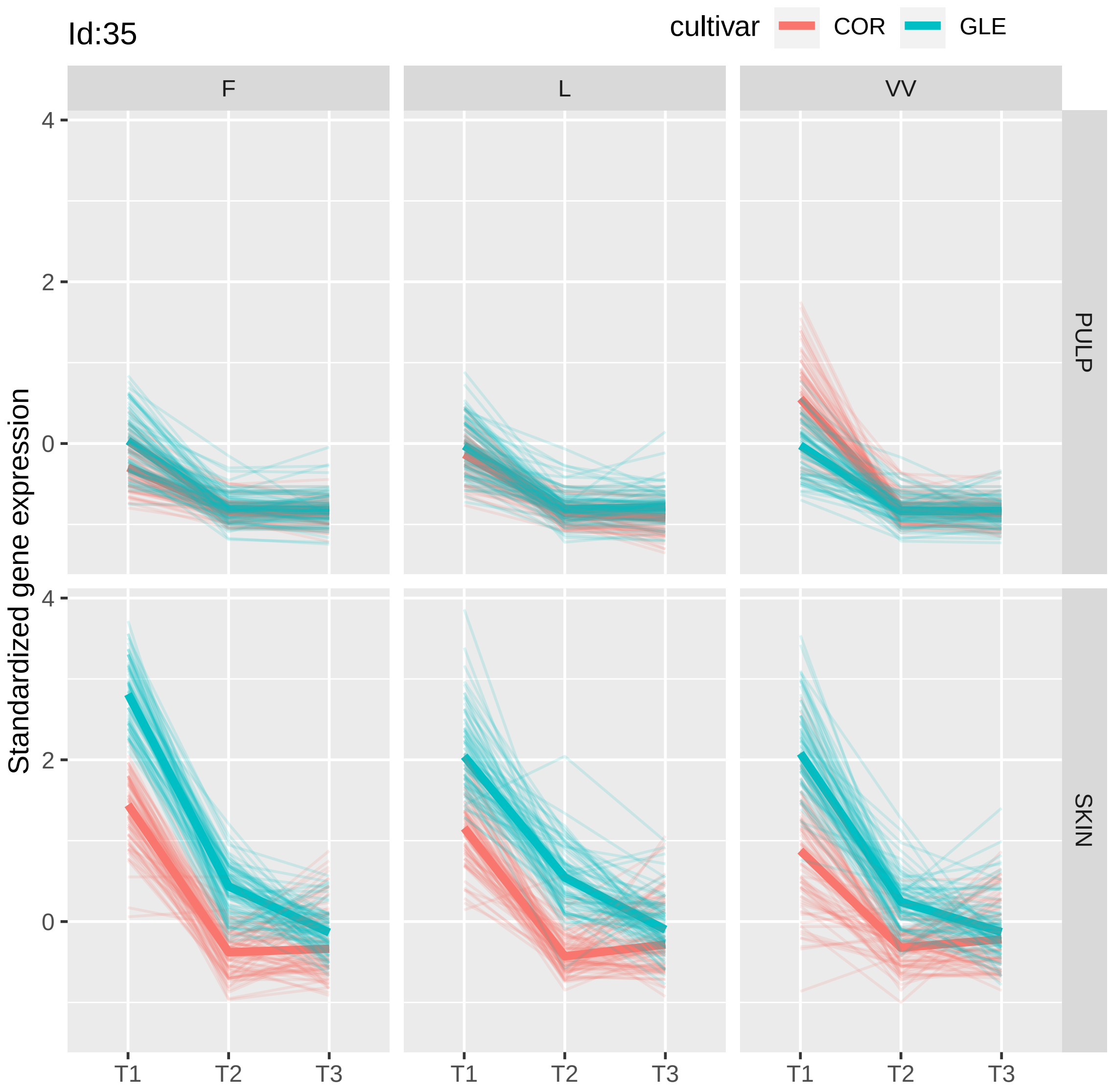

Id:35
cultivar
COR
GLE
F
L
VV
PULP
SKIN
Standardized gene expression
4
2
0
T1
T2
T3

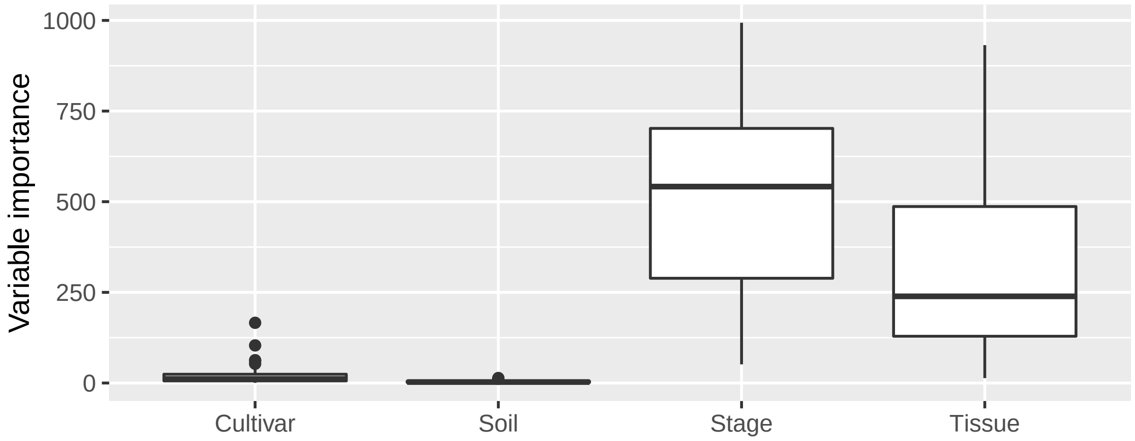

Variable importance
1000
750
500
250
0
Cultivar
Soil
Stage
Tissue

Cluster no. 30

```
## Number of genes in the cluster: 100
## Homogeneity Index:     0.8
## Variable importance for Stage:     Rank =  30  - Median =  531.1
## Variable importance for Cultivar:  Rank =  62  - Median =  22.09
## Variable importance for Tissue:    Rank =  35  - Median =  269.5
## Variable importance for Soil:      Rank =  25  - Median =   6.97
##
## Gene ID                Gene Annotation
## VIT_14s0006g02240  -  2-hydroxyacyl-CoA lyase 1
## VIT_16s0022g00960  -  Invertase/pectin methylesterase inhibitor
## VIT_05s0020g03870  -  MLK/Raf-related protein kinase 1
## VIT_08s0040g00770  -  Cysteine protease inhibitor
## VIT_19s0015g00610  -  Unknown protein
## VIT_06s0004g05120  -  ARR1 typeB
## VIT_14s0066g01140  -  Unknown protein
## VIT_05s0020g04080  -  1,2-dihydroxy-3-keto-5-methylthiopentene dioxygenase 2
## VIT_15s0046g00060  -  Arginine/serine-rich splicing factor RSP31 (RSP31)
## VIT_19s0085g00600  -  Glyceraldehyde-3-phosphate dehydrogenase, cytosolic
## VIT_11s0016g00340  -  DNA repair protein RAD51
## VIT_09s0002g03440  -  Protein kinase
## VIT_18s0001g03580  -  Ubiquitin-fold modifier 1 precursor
## VIT_04s0023g00130  -  Unknown protein
## VIT_12s0057g00690  -  Histidine kinase (AHK2)
## VIT_18s0001g15020  -  DnaJ homolog, subfamily A, member 4
## VIT_11s0016g01930  -  Diacylglycerol acyltransferase
## VIT_14s0108g01520  -  Glycosyl transferase family 8 protein
## VIT_09s0002g08660  -  F-box protein (FBL14)
## VIT_08s0056g00710  -  Protein kinase C inhibitor
## VIT_01s0011g04830  -  Extensin
## VIT_14s0060g00410  -  EDA32 (embryo sac development arrest 32)
## VIT_00s0665g00040  -  RAB GTPase RABH1E
## VIT_13s0067g00960  -  Ser/Thr protein kinase
## VIT_18s0041g00590  -  Proton-dependent oligopeptide transport (POT) family protein
## VIT_18s0001g14110  -  Transcription factor E2F/dimerisation partner (TDP) E2F2
## VIT_13s0019g01290  -  Crossover junction endonuclease MUS81
## VIT_08s0007g05560  -  Protein kinase
## VIT_07s0005g02600  -  Ribosomal protein L7Ae
## VIT_19s0090g00240  -  R protein disease resistance protein
## VIT_09s0002g07110  -  KEG (keep on going)
## VIT_08s0007g07960  -  DnaJ homolog, subfamily C, member 9
## VIT_04s0008g04500  -  No hit
## VIT_05s0020g04550  -  No hit
## VIT_13s0074g00210  -  Pyruvate kinase
## VIT_08s0040g00150  -  PQ-loop repeat / transmembrane
## VIT_14s0219g00110  -  Unknown protein
## VIT_19s0085g00610  -  No hit
## VIT_00s1188g00010  -  Unknown
## VIT_00s0508g00030  -  Membrane protein
## VIT_00s0335g00080  -  R protein MLA10
## VIT_14s0060g02640  -  Myb family
## VIT_04s0008g01380  -  Unknown protein
## VIT_13s0084g00230  -  flavonol sulfotransferase
## VIT_19s0090g01430  -  EDM2
## VIT_06s0009g03330  -  Cig3
```

```
## VIT_12s0057g01360  -  Unknown protein
## VIT_11s0037g00610  -  Unknown protein
## VIT_02s0025g01320  -  No hit
## VIT_08s0007g05900  -  GCN5 N-acetyltransferase (GNAT)
## VIT_18s0001g09490  -  Metal transporter Nramp2
## VIT_00s0338g00010  -  F-box family protein
## VIT_12s0028g03360  -  Serine/threonine-protein kinase bub1,checkpoint-associated
## VIT_05s0051g00930  -  DNA primase large subunit
## VIT_00s0179g00190  -  Transcription factor jumonji (jmjC) domain-containing protein
## VIT_08s0007g05250  -  Cig3
## VIT_04s0044g00860  -  Protein BRUSHY 1 (Protein TONSOKU) (Protein MGOUN 3)
## VIT_00s2317g00010  -  RAB GDP dissociation inhibitor 2 ATGDI2
## VIT_19s0014g02650  -  Glycosyl transferase family 14 protein
## VIT_08s0056g00430  -  Unknown protein
## VIT_13s0084g00220  -  Unknown protein
## VIT_12s0028g03600  -  Unknown protein
## VIT_00s0313g00050  -  RAB GDP dissociation inhibitor 1 ATGD1
## VIT_06s0009g02230  -  Cysteine protease inhibitor
## VIT_02s0025g02040  -  Unknown protein
## VIT_16s0050g02670  -  Spermine synthase
## VIT_06s0004g03910  -  Unknown protein
## VIT_04s0023g02880  -  GATA transcription factor 14
## VIT_10s0003g03670  -  Unknown protein
## VIT_16s0039g00900  -  CYP89A28
## VIT_10s0003g01720  -  Nucleoside triphosphatase
## VIT_13s0019g01240  -  Actin beta/gamma 1
## VIT_02s0025g04430  -  Sugar transporter 1
## VIT_02s0234g00100  -  Ubiquitinyl hydrolase 1
## VIT_11s0016g00090  -  Ubiquitin-specific protease 23 (UBP23)
## VIT_05s0029g00640  -  Retrotransposon
## VIT_09s0002g02130  -  CA-responsive protein
## VIT_05s0049g01890  -  Unknown
## VIT_06s0061g01610  -  DNA polymerase epsilon, subunit A
## VIT_18s0001g03890  -  No hit
## VIT_14s0066g01350  -  No hit
## VIT_11s0118g00490  -  Octicosapeptide/Phox/Bem1p (PB1) domain-containing protein
## VIT_13s0019g03220  -  Proline transporter 1 (ProT1)
## VIT_07s0005g03310  -  Cofilin
## VIT_10s0116g01350  -  Heat shock protein binding
## VIT_11s0016g03330  -  Unknown protein
## VIT_06s0004g00380  -  Unknown protein
## VIT_05s0077g02120  -  Unknown protein
## VIT_17s0000g02360  -  Receptor protein kinase
## VIT_19s0090g01660  -  RNA-binding protein Musashi
## VIT_13s0067g03430  -  ARR9 typeA
## VIT_14s0108g00810  -  Mini zinc finger 2 MIF2
## VIT_11s0052g01330  -  Xyloglucan endotransglucosylase/hydrolase 23
## VIT_13s0158g00240  -  No hit
## VIT_05s0020g04270  -  Ca2+-ATPase 13 ACA13, plasma membrane
## VIT_16s0098g00790  -  Copper-binding family protein
## VIT_05s0062g00610  -  Xyloglucan endotransglucosylase/hydrolase 23
## VIT_01s0011g05250  -  Senescence-associated protein
## VIT_05s0020g04730  -  Zinc finger (C3HC4-type ring finger)
## VIT_05s0020g02910  -  STE11 protein kinase homolog NPK1
```

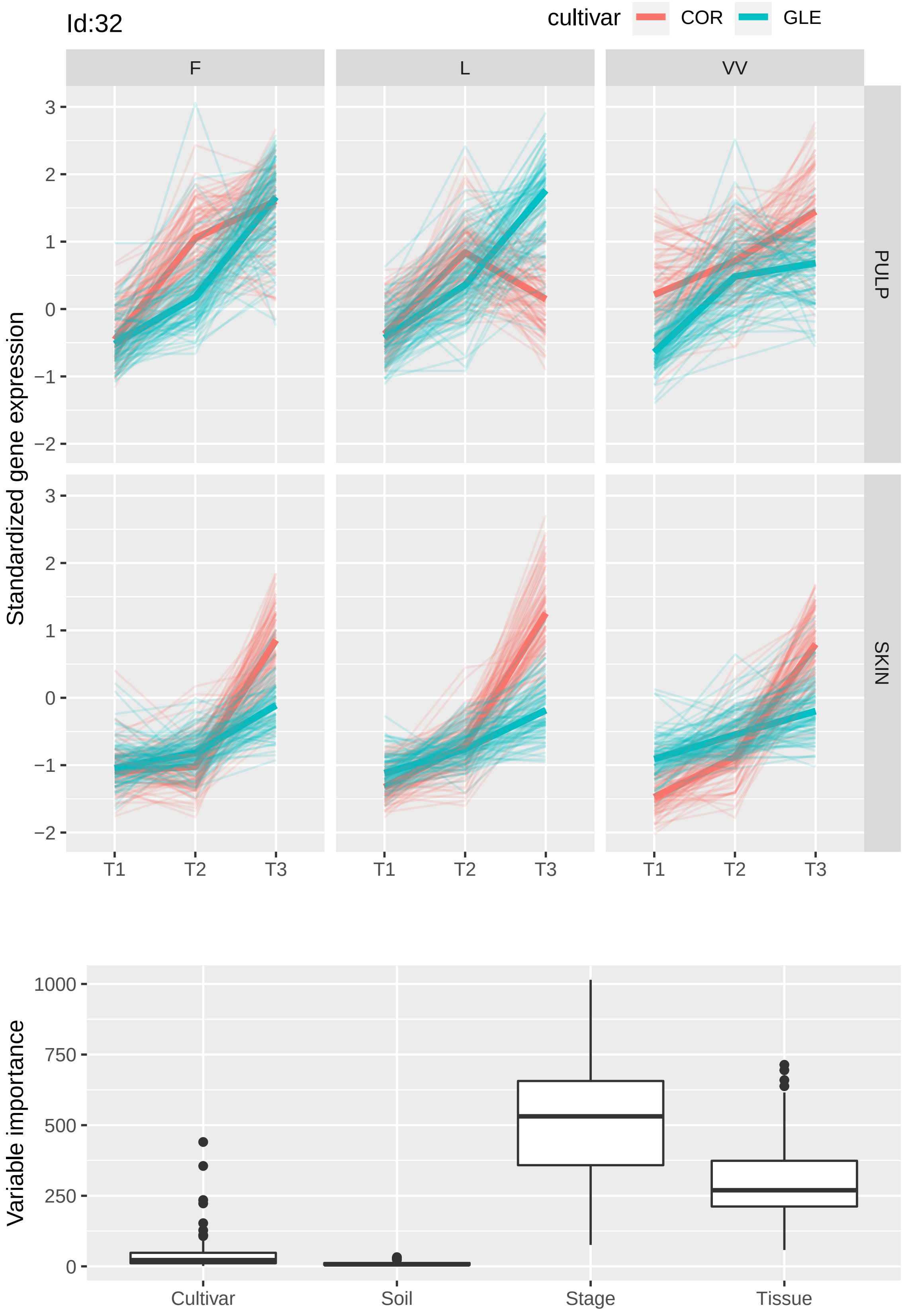

Id:32
cultivar
COR
GLE
F
L
VV
PULP
SKIN
Standardized gene expression
T1
T2
T3
Variable importance
Cultivar
Soil
Stage
Tissue

Cluster no. 31

```
## Number of genes in the cluster: 84
## Homogeneity Index:    0.82
## Variable importance for Stage:     Rank =  31  - Median =  523.5
## Variable importance for Cultivar:  Rank =  98  - Median =    5.7
## Variable importance for Tissue:    Rank =  63  - Median =  72.16
## Variable importance for Soil:      Rank =  58  - Median =   5.25
##
## Gene ID                Gene Annotation
## VIT_17s0000g01260  -  Squamosa promoter-binding protein (VvSBP15)
## VIT_09s0002g02230  -  Harpin-induced 1
## VIT_04s0023g01140  -  GNS1/SUR4 membrane protein
## VIT_15s0046g01210  -  Arbutin synthase
## VIT_10s0003g02780  -  Unknown protein
## VIT_00s2829g00010  -  Unknown
## VIT_09s0002g00670  -  Auxin responsive SAUR protein
## VIT_04s0023g02840  -  Leucine-rich repeat disease resistance protein
## VIT_10s0042g01020  -  Isochorismatase
## VIT_00s0323g00060  -  Invertase/pectin methylesterase inhibitor
## VIT_00s0238g00020  -  Unknown protein
## VIT_01s0137g00460  -  RGLG2 (ring Domain LIGASE2)
## VIT_17s0000g06020  -  TCP family transcription factor TCP14
## VIT_11s0016g05290  -  Bundle-sheath defective protein 2 family / bsd2 family
## VIT_06s0080g00590  -  Harpin-induced protein
## VIT_18s0001g01470  -  No hit
## VIT_10s0003g04040  -  Mitochondrial transcription termination factor
## VIT_00s2269g00010  -  Armadillo/beta-catenin repeat
## VIT_13s0084g00810  -  Armadillo
## VIT_15s0021g01880  -  Arabidopsis thaliana homeobox protein 2
## VIT_13s0067g01410  -  Anthranilate N-hydroxycinnamoyl/benzoyltransferase
## VIT_15s0048g00670  -  Ribosomal protein S13 (RPS13A) 40S
## VIT_04s0008g05530  -  Unknown protein
## VIT_12s0059g02180  -  MATE efflux family protein
## VIT_09s0002g01400  -  myb domain protein 7
## VIT_07s0104g01440  -  Phototropic-responsive NPH3
## VIT_13s0019g02140  -  Tropinone reductase
## VIT_15s0021g02270  -  Unknown protein
## VIT_08s0007g06670  -  Homeobox-leucine zipper protein 14
## VIT_03s0038g01110  -  Auxin-responsive SAUR31
## VIT_18s0001g08430  -  Branched-chain-amino-acid aminotransferase 2, chloroplast (Atbcat-2)
## VIT_08s0007g08680  -  Unknown protein
## VIT_05s0029g01450  -  No hit
## VIT_04s0043g00850  -  Unknown protein
## VIT_17s0000g05600  -  (-)-isopiperitenol dehydrogenase
## VIT_08s0007g02500  -  Transcription termination factor mitochondrial mTERF
## VIT_05s0029g00080  -  SERK family receptor-like protein kinase
## VIT_03s0038g01090  -  Auxin responsive SAUR protein
## VIT_11s0016g03940  -  Heat shock transcription factor C1
## VIT_18s0041g00830  -  UDP-glucose: anthocyanidin 5,3-O-glucosyltransferase
## VIT_14s0036g00610  -  Ethylene-induced calmodulin-binding protein B
## VIT_15s0046g01960  -  Hydroquinone glucosyltransferase
## VIT_10s0003g04320  -  PINOID
## VIT_18s0001g11800  -  BEL1-like homeodomain protein 4 (SAWTOOTH 2)
## VIT_11s0016g05230  -  Allyl alcohol dehydrogenase
## VIT_14s0068g01020  -  Unknown protein
```

```
## VIT_19s0014g05220  -  Unknown protein
## VIT_02s0087g00150  -  No hit
## VIT_02s0033g00260  -  Pinoresinol-lariciresinol reductase
## VIT_08s0007g07710  -  CYP93A1 2-hydroxyisoflavanone synthase
## VIT_04s0008g00740  -  Protein phosphatase 2C
## VIT_18s0001g06690  -  Gibberellin 20 oxidase 2
## VIT_00s0253g00140  -  Polyneuridine-aldehyde esterase precursor
## VIT_15s0046g01340  -  Unknown protein
## VIT_18s0001g12880  -  Jasmonate O-methyltransferase
## VIT_12s0034g01240  -  GNS1/SUR4 membrane
## VIT_00s1679g00010  -  Avr9/Cf-9 rapidly elicited protein 75
## VIT_18s0001g09780  -  S-locus receptor protein kinase
## VIT_18s0001g10330  -  Subtilisin protease
## VIT_10s0003g01960  -  RKF1 (receptor-like kinase in flowers 1)
## VIT_05s0062g00430  -  UDP-glucose:flavonoid 7-O-glucosyltransferase
## VIT_06s0009g03390  -  No hit
## VIT_01s0182g00140  -  PHO1-like protein
## VIT_04s0008g03630  -  Binding
## VIT_15s0048g00550  -  Abnormal floral organs
## VIT_18s0041g01040  -  UDP-glycosyltransferase 88A4
## VIT_19s0014g00160  -  LHCII type I CAB-1
## VIT_01s0011g04700  -  Heavy-metal-associated domain-containing protein
## VIT_03s0063g00030  -  Unknown protein
## VIT_19s0014g02730  -  Cytochrome c oxidase subunit XVII assembly protein
## VIT_05s0020g03750  -  Protease inhibitor/seed storage/lipid transfer protein (LTP)
## VIT_16s0039g01710  -  myb domain protein 9
## VIT_00s0394g00010  -  Carboxyl-terminal proteinase
## VIT_14s0108g00750  -  Zinc Finger Homeodomain Transcription Factor (VvZHD8)
## VIT_18s0001g09740  -  S-locus receptor protein kinase
## VIT_10s0003g03560  -  Beta-amyrin synthase
## VIT_04s0008g01800  -  myb domain protein 7
## VIT_15s0048g01390  -  Gibberellin receptor GID1L3
## VIT_05s0077g02260  -  Nodulin MtN3 family
## VIT_00s1566g00010  -  UDP-rhamnose/rhamnosyltransferase
## VIT_03s0063g02460  -  Transcription termination factor mitochondrial mTERF
## VIT_07s0031g00750  -  MATE efflux family protein
## VIT_08s0040g02820  -  Unknown protein
## VIT_08s0040g02950  -  Zinc finger (C3HC4-type ring finger)
```

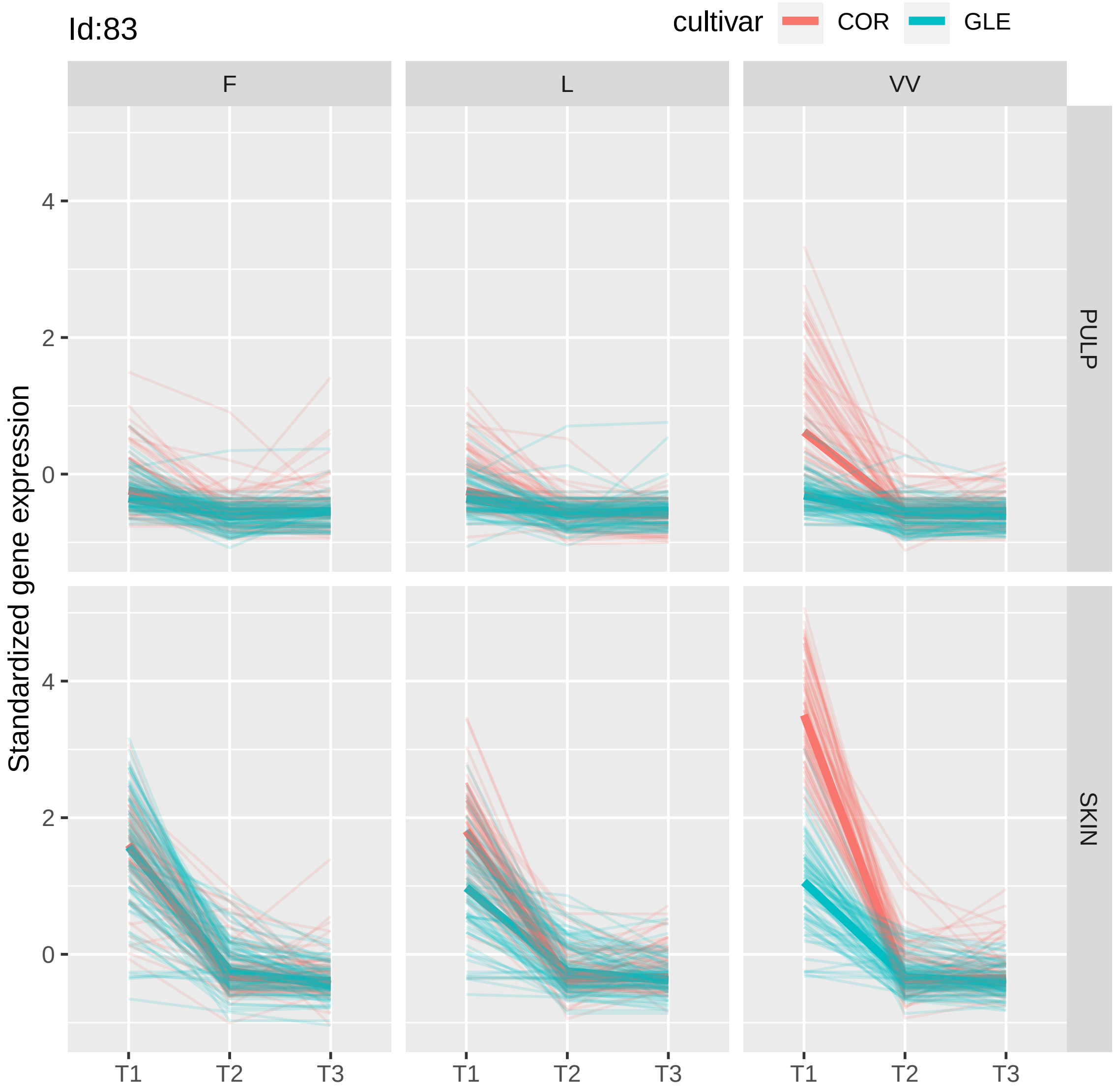

Id:83
cultivar
COR
GLE
F
L
VV
PULP
SKIN
Standardized gene expression
4
2
0
T1
T2
T3

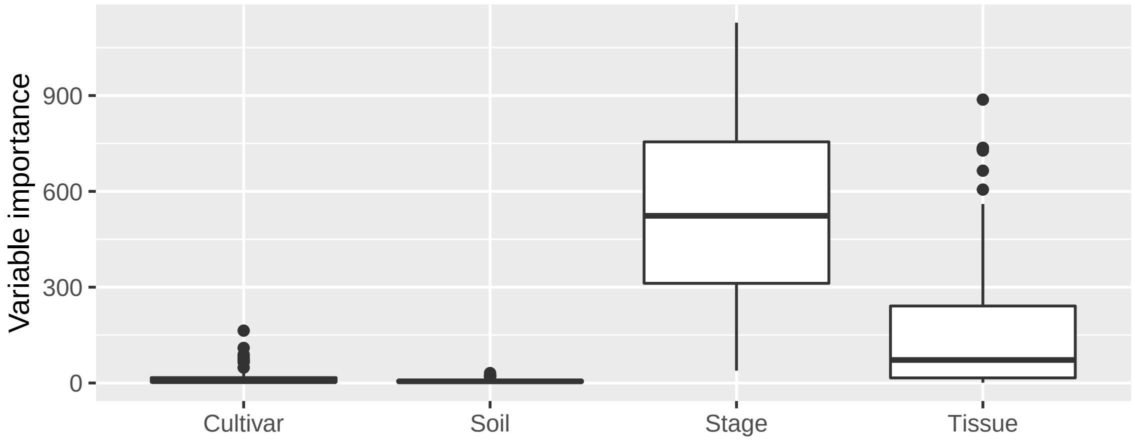

Variable importance
900
600
300
0
Cultivar
Soil
Stage
Tissue

Cluster no. 32

```
## Number of genes in the cluster: 210
## Homogeneity Index:    0.89
## Variable importance for Stage:     Rank =  32  - Median =    523
## Variable importance for Cultivar:  Rank =  86  - Median =   9.86
## Variable importance for Tissue:    Rank =  49  - Median =  138.2
## Variable importance for Soil:      Rank =  89  - Median =   3.51
##
## Gene ID                Gene Annotation
## VIT_07s0005g04130  -  Acid phosphatase class B
## VIT_02s0025g01150  -  No hit
## VIT_12s0142g00190  -  No hit
## VIT_07s0031g00540  -  Rapid ALkalinization Factor RALFL34
## VIT_18s0001g03080  -  Chitin elicitor-binding CEBIP LysM domain-containing
## VIT_03s0088g00380  -  Tubulin alpha
## VIT_07s0005g01320  -  Unknown
## VIT_19s0015g01620  -  Phenylalanine-tRNA ligase
## VIT_04s0044g00380  -  Nodulin MtN21 family
## VIT_08s0007g06170  -  Anthranilate phosphoribosyltransferase
## VIT_13s0064g01260  -  DNA-damage-repair/toleration protein (DRT100)
## VIT_00s0264g00050  -  Unknown protein
## VIT_19s0090g01040  -  No hit
## VIT_12s0028g02160  -  Ribulose bisphosphate carboxylase, large chain
## VIT_19s0014g03640  -  Myosin VII
## VIT_00s0253g00040  -  Monocopper oxidase SKS17 (SKU5 Similar 17)
## VIT_14s0068g00940  -  Receptor protein kinase
## VIT_12s0028g02540  -  Glucan endo-1,3-beta-glucosidase 1 precursor
## VIT_06s0004g04640  -  Metallothionein 2b
## VIT_14s0083g00400  -  Leaf senescence protein
## VIT_06s0004g06660  -  Phosphoinositide-specific phospholipase C
## VIT_14s0108g01320  -  Plastocyanin domain-containing protein
## VIT_17s0000g01190  -  Phosphatidylglycerol specific phospholipase C
## VIT_01s0011g00120  -  Unknown protein
## VIT_00s0220g00180  -  Glycosyl hydrolase family 20 protein
## VIT_14s0006g00450  -  myb domain protein 20
## VIT_06s0004g05080  -  Zinc finger (C3HC4-type ring finger)
## VIT_02s0025g00960  -  V-type H+-transporting ATPase subunit E
## VIT_17s0000g05570  -  Receptor protein kinase
## VIT_14s0066g01840  -  SAG101 (senescence-associated gene 101)
## VIT_09s0002g02300  -  Indeterminate(ID)-domain 7
## VIT_08s0032g00780  -  Calcium Dependent Protein Kinase (VvCPK8)
## VIT_01s0011g02720  -  No hit
## VIT_16s0039g00390  -  Unknown protein
## VIT_07s0005g00090  -  Auxin-responsive GH3
## VIT_15s0048g02530  -  Harpin-induced 1
## VIT_11s0016g02950  -  Profilin 1 (PRO1) (PFN1) (PRF1) / allergen Ara t 8
## VIT_12s0028g01970  -  Serine/threonine-protein kinase NAK
## VIT_07s0130g00060  -  Unknown protein
## VIT_14s0083g00260  -  No hit
## VIT_14s0068g00360  -  LNG1 (LONGIFOLIA1)
## VIT_00s0445g00010  -  Strubbelig receptor family 1
## VIT_00s2380g00010  -  S-locus lectin protein kinase
## VIT_11s0016g00200  -  CBL-interacting protein kinase 6 (CIPK6)
## VIT_00s0304g00010  -  Unknown protein
## VIT_06s0009g00310  -  Unknown protein
```

```
## VIT_00s0274g00070  -  basic helix-loop-helix (bHLH) family
## VIT_01s0137g00240  -  Pectate lyase
## VIT_01s0026g00980  -  Glycosyl transferase family 8 protein
## VIT_11s0016g01110  -  Unknown protein
## VIT_19s0090g01470  -  Protease inhibitor/seed storage/lipid transfer protein (LTP)
## VIT_08s0007g07600  -  Pyruvate kinase, cytosolic isozyme
## VIT_17s0000g02670  -  Transducin protein
## VIT_18s0001g04800  -  Hyperosmotically inducible periplasmic protein
## VIT_14s0006g00920  -  Unknown protein
## VIT_11s0016g00470  -  Sucrose synthase
## VIT_02s0025g02770  -  Glucan endo-1,3-beta-glucosidase 7 precursor
## VIT_04s0044g00360  -  MTN21
## VIT_02s0012g00890  -  Unknown protein
## VIT_13s0019g04750  -  Kinesin morphogenesis of root hair 2 (MRH2)
## VIT_04s0044g01040  -  basic helix-loop-helix (bHLH) family
## VIT_08s0040g00720  -  Snf1 protein kinase (KIN10) (SKIN10)
## VIT_17s0000g05000  -  putative MADS-box sepallata 2 (VviSEP2)
## VIT_05s0051g00590  -  Pectate lyase
## VIT_18s0041g00090  -  Cation efflux MTP11
## VIT_09s0002g06820  -  Equilibrative nucleoside transporter ENT8
## VIT_17s0000g05700  -  Phosphoribosylanthranilate transferase
## VIT_03s0038g01050  -  Phenylalanine-tRNA ligase
## VIT_19s0015g01650  -  Phenylalanine-tRNA ligase
## VIT_01s0011g04710  -  Copper-binding family protein
## VIT_17s0000g05880  -  Calcineurin phosphoesterase
## VIT_09s0002g00450  -  Subtilase
## VIT_13s0019g02490  -  Subtilisin protease C1
## VIT_02s0025g03480  -  Kinesin motor domain containing protein
## VIT_14s0036g00540  -  Ribulose bisphosphate carboxylase, large chain
## VIT_08s0007g07580  -  MYR1 (MYB-related protein 1)
## VIT_18s0117g00270  -  Zinc Finger Homeodomain Transcription Factor (VvZHD12)
## VIT_19s0085g00890  -  Myb APL (altered phloem development)
## VIT_01s0011g02710  -  No hit
## VIT_04s0008g03570  -  Unknown protein
## VIT_03s0038g04110  -  AT-hook DNA-binding protein
## VIT_12s0028g01730  -  Unknown protein
## VIT_16s0050g01860  -  SLAH3 (SLAC1 Homologue 3)
## VIT_02s0025g05110  -  MATE efflux family protein
## VIT_12s0028g03110  -  Caffeoyl-CoA 3-O-methyltransferase
## VIT_02s0025g02380  -  No hit
## VIT_12s0059g01260  -  Plastidic glucose transporter 1
## VIT_06s0004g00150  -  MYR1 (MYB-related protein 1)
## VIT_12s0134g00390  -  S-locus lectin protein kinase family
## VIT_01s0010g00330  -  Clavata1 receptor kinase (CLV1)
## VIT_17s0000g06370  -  Thioredoxin 2
## VIT_11s0016g04600  -  myb family
## VIT_08s0007g07700  -  Unknown protein
## VIT_03s0091g00490  -  Unknown protein
## VIT_00s0776g00010  -  Strubbelig receptor family 3
## VIT_04s0008g01040  -  Lysine decarboxylase
## VIT_11s0016g02420  -  UDP-N-acetylglucosamine O-acyltransferase protein
## VIT_18s0001g05840  -  RNA recognition motif (RRM)-containing protein
## VIT_01s0127g00690  -  MAP4K alpha1
## VIT_03s0038g02390  -  Ubiquitin-conjugating enzyme E2 D/E UBC28
```

```
## VIT_18s0001g14610  -  Clavata1 receptor kinase (CLV1)
## VIT_18s0117g00030  -  No hit
## VIT_17s0000g01060  -  No hit
## VIT_13s0064g00260  -  No hit
## VIT_07s0031g02720  -  Myosin heavy chain
## VIT_19s0014g05050  -  Heat shock  HSP20 family protein
## VIT_10s0116g01760  -  Myb caprice CPC
## VIT_07s0151g00800  -  Unknown
## VIT_04s0008g06050  -  Phytosulfokine receptor precursor
## VIT_17s0000g08860  -  UBX domain-containing protein
## VIT_14s0083g01220  -  feronia receptor-like kinase
## VIT_02s0025g03180  -  Nodulin MtN21 family
## VIT_17s0000g00040  -  Pentatricopeptide (PPR) repeat-containing
## VIT_18s0117g00060  -  R protein L6
## VIT_06s0009g00450  -  Aluminium-tolerance ALMT1
## VIT_03s0038g02730  -  Mutator-like transposase
## VIT_03s0180g00040  -  Cyclin D3_2
## VIT_00s0525g00050  -  Metal transporter CNNM4 (Cyclin-M4)
## VIT_04s0044g01300  -  DNA cross-link repair protein
## VIT_17s0000g00460  -  V-type H+-transporting ATPase subunit C
## VIT_12s0028g01560  -  Zinc finger (C3HC4-type ring finger)
## VIT_06s0004g06650  -  1-phosphatidylinositol-4,5-bisphosphate phosphodiesterase
## VIT_00s0652g00010  -  Dof zinc finger protein DOF1.4
## VIT_00s0567g00060  -  Patatin
## VIT_14s0060g02090  -  Copper chaperone (CCH)
## VIT_01s0146g00420  -  No hit
## VIT_11s0016g05880  -  Ubiquitin-specific protease 12 UBP12
## VIT_07s0031g02340  -  Amino acid permease 2
## VIT_13s0019g04420  -  BTB/POZ; NPH3
## VIT_00s0131g00010  -  Annexin ANN3
## VIT_11s0052g00260  -  Unknown protein
## VIT_07s0031g00760  -  EF hand
## VIT_15s0046g01900  -  ferric reduction oxidase 2
## VIT_13s0156g00100  -  Unknown protein
## VIT_10s0042g01160  -  Elongation factor EF-2
## VIT_11s0016g04220  -  Copper-binding family protein
## VIT_11s0016g00210  -  No hit
## VIT_07s0129g00490  -  Hydrogenobyrinic acid a,c-diamide synthase
## VIT_14s0083g00410  -  Unknown
## VIT_01s0011g02940  -  basic helix-loop-helix (bHLH) family
## VIT_08s0007g02950  -  Unknown protein
## VIT_12s0142g00310  -  Oxygen evolving enhancer 3 (PsbQ)
## VIT_04s0008g01860  -  CYP72A58
## VIT_11s0052g00010  -  No hit
## VIT_14s0060g02320  -  Respiratory burst oxidase protein B (ATRBOHB)
## VIT_15s0048g00680  -  NIK3 (NSP- interacting  kinase 3)
## VIT_17s0000g08590  -  Unknown protein
## VIT_15s0048g02320  -  NAC domain-containing protein (VvNAC51)
## VIT_17s0000g06920  -  Remorin
## VIT_05s0077g01920  -  No hit
## VIT_02s0087g00370  -  Anthranilate N-hydroxycinnamoyl/benzoyltransferase
## VIT_17s0000g02500  -  RAB GTPase RABA6B
## VIT_01s0011g05100  -  Bet v I allergen family
## VIT_06s0004g07910  -  Ovate family protein 3 OFP3
```

```
## VIT_11s0052g00020  -  Balbiani ring 1
## VIT_14s0068g00370  -  No hit
## VIT_09s0070g00210  -  Metal transporter Nramp1
## VIT_03s0091g00240  -  Haloacid dehalogenase hydrolase
## VIT_06s0080g00680  -  No hit
## VIT_12s0057g00800  -  Receptor Like Protein 27
## VIT_18s0072g00440  -  UDP-glucose:sterol glucosyltransferase
## VIT_12s0028g00510  -  Lipase class 3
## VIT_01s0026g00730  -  Unknown protein
## VIT_04s0023g00590  -  Regulator of ribonuclease activity a
## VIT_13s0019g01410  -  OBP3 (OBF-binding protein 3)
## VIT_09s0002g04780  -  Sulfate transporter 3.4
## VIT_04s0008g01160  -  Indeterminate(ID)-domain 7
## VIT_01s0011g01790  -  Galactose-binding like
## VIT_07s0005g00940  -  Kinesin motor protein
## VIT_10s0042g00370  -  R protein disease resistance protein
## VIT_10s0003g04050  -  Unknown protein
## VIT_18s0001g02020  -  Amino acid permease 3
## VIT_06s0004g06820  -  Unknown protein
## VIT_06s0004g01780  -  Serine/threonine-protein kinase OXI1
## VIT_17s0000g01050  -  Corticosteroid 11-beta-dehydrogenase isozyme 1
## VIT_18s0001g09250  -  Lateral organ boundaries protein 38
## VIT_17s0000g08290  -  Dof zinc finger protein DOF5.6
## VIT_09s0002g02620  -  Lectin jacalin
## VIT_14s0128g00080  -  Lipase GDSL
## VIT_07s0129g00480  -  Molecular chaperone DnaJ
## VIT_14s0108g00030  -  Pectate lyase
## VIT_14s0060g02150  -  Rapid ALkalinization Factor RALFL33
## VIT_03s0017g01010  -  Peptidase C14, caspase catalytic subunit p20
## VIT_13s0073g00680  -  Endonuclease/exonuclease/phosphatase family protein
## VIT_18s0001g06380  -  R protein L6
## VIT_08s0040g00710  -  3'-5' exonuclease domain-containing protein
## VIT_04s0008g03240  -  BAM2 (big apical meristem 2)
## VIT_17s0000g00830  -  Nodulin MtN3 family
## VIT_14s0006g02880  -  neoxanthin synthase (NSY) (VvNSY1)
## VIT_10s0597g00020  -  UBA; HSC20, C-terminal oligomerisation
## VIT_10s0003g05370  -  Cytochrome b5-like Fatty acid hydroxylase
## VIT_14s0066g01390  -  Metal-dependent phosphohydrolase HD domain-containing protein
## VIT_10s0003g03250  -  Unknown protein
## VIT_01s0010g00510  -  Unknown protein
## VIT_09s0002g02120  -  Beta-galactosidase
## VIT_14s0066g02590  -  Nitrate transporter NRT1-5
## VIT_14s0128g00430  -  Protein kinase NPK1 (ANP1)
## VIT_14s0171g00110  -  Lipase family
## VIT_18s0089g00050  -  NBS-LRR disease resistance protein
## VIT_07s0104g01420  -  Glutaredoxin
## VIT_02s0025g00700  -  Aluminum-activated malate transporter 9
## VIT_09s0002g04620  -  Unknown protein
## VIT_18s0001g12510  -  5' nucleotidase
## VIT_00s2507g00010  -  F-box family protein
## VIT_10s0116g00810  -  Root hair defective 3 GTP-binding (RHD3)
## VIT_10s0116g00930  -  Root hair defective 3 GTP-binding (RHD3)
## VIT_08s0040g01890  -  Aquaporin PIP2;4
## VIT_19s0014g01780  -  Basic Leucine Zipper Transcription Factor (VvbZIP52)
## VIT_15s0048g02290  -  NAC domain-containing protein (VvNAC54)
```

```
## VIT_08s0040g02470  -  Inorganic pyrophosphatase
```

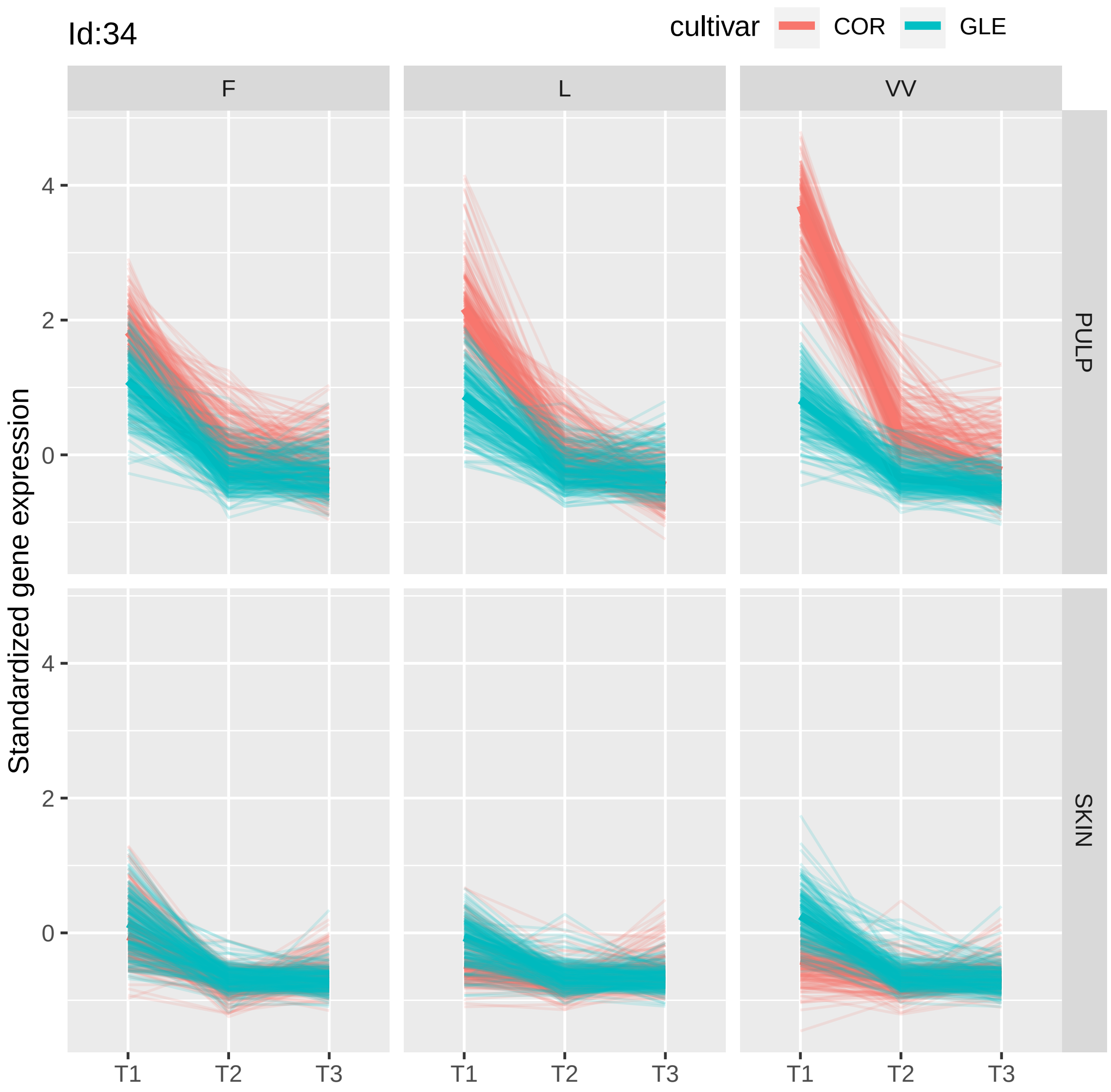
Id:34
cultivar
COR
GLE
F
L
VV
PULP
SKIN
Standardized gene expression
4
2
0
T1
T2
T3

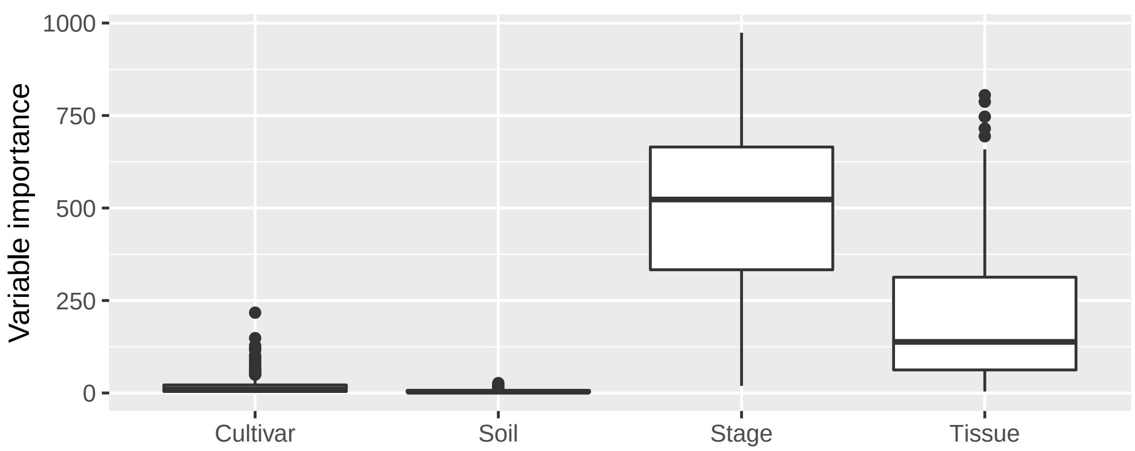
Variable importance
1000
750
500
250
0
Cultivar
Soil
Stage
Tissue

Cluster no. 33

```
## Number of genes in the cluster: 78
## Homogeneity Index:    0.73
## Variable importance for Stage:     Rank =  33  - Median =  481.6
## Variable importance for Cultivar:  Rank =  45  - Median =  50.77
## Variable importance for Tissue:    Rank =  76  - Median =   30.9
## Variable importance for Soil:      Rank =  11  - Median =  10.35
##
## Gene ID                Gene Annotation
## VIT_16s0115g00110  -  Unknown
## VIT_06s0061g00760  -  Zinc finger (C2H2 type) family
## VIT_12s0028g03010  -  Glutaredoxin
## VIT_13s0156g00260  -  Homeobox-leucine zipper protein HAT14
## VIT_14s0068g02120  -  CYP94B3
## VIT_15s0048g00830  -  LOB domain-containing 18 (Asymmetric leaves 2-like protein 20)
## VIT_16s0115g00120  -  transformation/transcription domain-associated protein
## VIT_09s0054g01890  -  No hit
## VIT_16s0115g00130  -  Clathrin, heavy polypeptide
## VIT_01s0010g01340  -  GTPase activating protein
## VIT_12s0059g01220  -  Pyrophosphate-dependent phosphofructokinase beta subunit
## VIT_18s0001g10300  -  basic helix-loop-helix (bHLH) family
## VIT_01s0011g04870  -  Homeobox-leucine zipper protein 17 (HB-17)
## VIT_12s0028g02320  -  Pentatricopeptide (PPR) repeat-containing
## VIT_07s0031g02330  -  Anaphase-promoting complex component APC8
## VIT_04s0008g05770  -  CBL-interacting protein kinase 25 (CIPK25)
## VIT_16s0100g00040  -  No hit
## VIT_00s0366g00010  -  No hit
## VIT_19s0090g01420  -  Wax synthase isoform 1
## VIT_00s0204g00120  -  Subtilisin-like serine protease 3
## VIT_18s0001g12450  -  No hit
## VIT_00s0302g00060  -  No hit
## VIT_14s0060g01330  -  Lipoamide dehydrogenase
## VIT_00s0198g00040  -  CTV.22
## VIT_04s0008g04230  -  ABC Transporter (VvPDR28 - VvABCG58)
## VIT_11s0016g02340  -  Cytidine deaminase
## VIT_12s0034g01810  -  2OG-Fe(II) oxygenase
## VIT_08s0007g06470  -  VQ motif-containing protein
## VIT_12s0035g01340  -  Unknown protein
## VIT_18s0122g00570  -  MBD9 (methyl-CPG-binding Domain 9)
## VIT_15s0048g00500  -  Pectinesterase family
## VIT_00s1881g00010  -  No hit
## VIT_08s0058g01110  -  Ovate family protein 4 OFP4
## VIT_05s0020g01680  -  No hit
## VIT_00s0313g00010  -  Centromere protein
## VIT_09s0096g00680  -  C2-HC type zinc finger protein C.e-MyT1
## VIT_18s0001g09110  -  Photosystem II PsbC protein
## VIT_15s0048g00480  -  Pectinesterase inhibitor
## VIT_08s0040g00130  -  Copper-binding family protein
## VIT_08s0007g07640  -  NAC domain-containing protein (VvNAC61)
## VIT_13s0067g03010  -  Unknown
## VIT_10s0003g00140  -  ERF/AP2 Gene Family (VvERF064)
## VIT_13s0106g00720  -  Unknown protein
## VIT_15s0048g00970  -  Cytokinesis Sec1 protein (KEULE)
## VIT_07s0104g01340  -  Nodulin MtN3 family
## VIT_14s0066g01090  -  myb domain protein 24
```

```
## VIT_00s0616g00010  -  Glycerol-3-phosphate permease
## VIT_08s0056g00290  -  Calcium-binding protein CML
## VIT_09s0096g00430  -  C2-HC type zinc finger protein C.e-MyT1
## VIT_14s0068g00740  -  Avr9/Cf-9 rapidly elicited protein 146
## VIT_18s0089g00190  -  1,4-beta-mannan endohydrolase
## VIT_05s0051g00150  -  RRS1 (resistant to ralstonia solanacearum 1)
## VIT_09s0096g00720  -  C2-HC type zinc finger protein C.e-MyT1
## VIT_07s0005g01580  -  Invertase/pectin methylesterase inhibitor
## VIT_17s0000g06070  -  No hit
## VIT_18s0001g09000  -  No hit
## VIT_14s0060g00040  -  Retrotransposon
## VIT_19s0140g00050  -  No hit
## VIT_09s0002g00140  -  Lipase class 3
## VIT_14s0036g01270  -  F-type H+-transporting ATPase subunit a
## VIT_19s0090g01200  -  Unknown protein
## VIT_10s0003g00320  -  Unknown protein
## VIT_01s0011g03670  -  Bifunctional nuclease
## VIT_12s0028g02140  -  F-box/LRR-repeat MAX2
## VIT_19s0014g04930  -  Germacrene-D synthase (VvTPS28)
## VIT_12s0028g03560  -  Unknown protein
## VIT_16s0039g01550  -  Unknown protein
## VIT_18s0001g15390  -  Gaiacol peroxidase
## VIT_00s0378g00040  -  CC-NBS-LRR class
## VIT_16s0022g01980  -  Zf A20 and AN1 domain-containing stress-associated protein 1
## VIT_09s0002g08300  -  R protein MLA10
## VIT_07s0129g00060  -  Adenylylsulfate kinase
## VIT_08s0007g04760  -  Tankyrase 1
## VIT_09s0070g00960  -  No hit
## VIT_02s0012g03110  -  CTV.22
## VIT_15s0021g01710  -  No hit
## VIT_16s0050g00840  -  Unknown protein
## VIT_03s0091g00510  -  Unknown
```

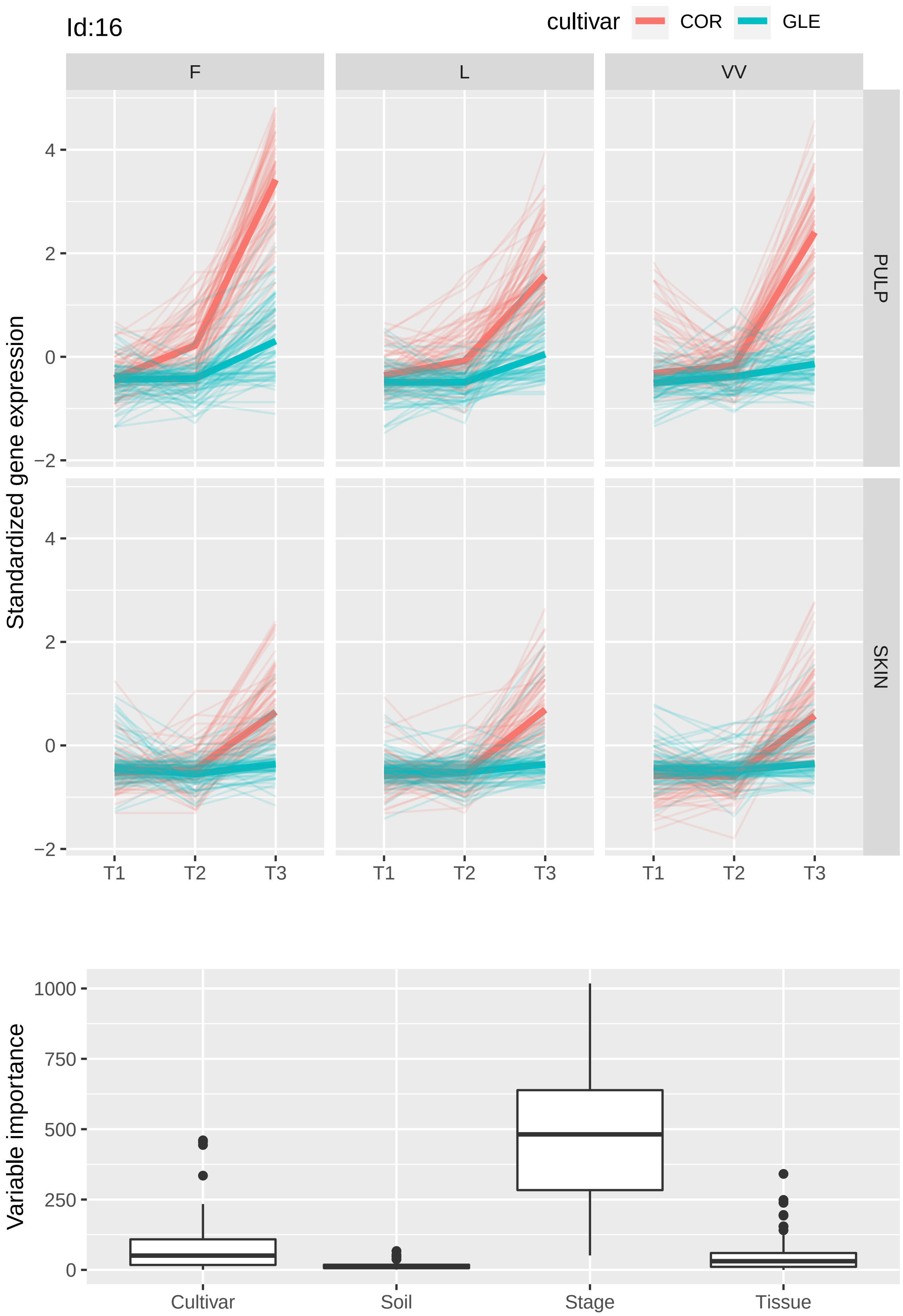

Id:16
cultivar
COR
GLE
F
L
VV
PULP
SKIN
Standardized gene expression
4
2
0
−2
T1
T2
T3
Variable importance
1000
750
500
250
0
Cultivar
Soil
Stage
Tissue

Cluster no. 34

```
## Number of genes in the cluster: 116
## Homogeneity Index:    0.79
## Variable importance for Stage:     Rank =  34  - Median =  449.8
## Variable importance for Cultivar:  Rank =  85  - Median =   9.96
## Variable importance for Tissue:    Rank =  34  - Median =    275
## Variable importance for Soil:      Rank =  72  - Median =   4.58
##
## Gene ID                Gene Annotation
## VIT_02s0033g01330  -  Acyl-CoA binding protein
## VIT_06s0004g07790  -  Lateral organ boundaries Domain 15
## VIT_18s0001g15460  -  Stearyl acyl carrier protein desaturase
## VIT_19s0177g00290  -  No hit
## VIT_01s0026g00940  -  ARR3 typeA
## VIT_19s0090g01370  -  No hit
## VIT_00s0394g00040  -  Alliinase EGF
## VIT_00s0590g00020  -  No hit
## VIT_14s0066g01190  -  Unknown protein
## VIT_05s0049g00760  -  No hit
## VIT_08s0056g01600  -  No hit
## VIT_01s0010g01710  -  Phytosulfokine receptor
## VIT_04s0008g01230  -  Harpin-induced protein
## VIT_19s0014g02880  -  Glycosyl hydrolase family 5 protein
## VIT_14s0030g00520  -  Disease resistance protein (NBS-LRR class)
## VIT_16s0100g00400  -  ERF/AP2 Gene Family (VvERF019),Dehydration Responsive Element-Binding
## VIT_15s0021g02700  -  Expansin  (VvEXPB4)
## VIT_00s0662g00020  -  Unknown protein
## VIT_18s0001g06080  -  UDP-glycosyltransferase 85A1
## VIT_04s0043g00840  -  Armadillo/beta-catenin repeat protein / U-box domain-containing protei
## VIT_05s0049g00510  -  Ethylene response factor ERF1
## VIT_02s0012g00500  -  Invertase/pectin methylesterase inhibitor
## VIT_11s0016g00640  -  Auxin response factor 11 ARF19
## VIT_10s0116g00370  -  Unknown protein
## VIT_19s0090g01340  -  No hit
## VIT_06s0004g03390  -  TET3 (tetraspanin3)
## VIT_08s0007g04470  -  Vacuolar protein sorting 37C
## VIT_17s0000g09770  -  Cysteine proteinase
## VIT_03s0038g04750  -  Universal stress protein (USP) family protein
## VIT_14s0030g01260  -  CRK1 protein
## VIT_13s0067g00660  -  Steroid 23-alpha-hydroxylase
## VIT_00s0125g00190  -  Potassium transporter (KUP1)
## VIT_01s0010g02480  -  Ankyrin repeat
## VIT_11s0103g00010  -  Potassium-sodium symporter HKT2
## VIT_01s0026g00850  -  Zinc finger protein 5
## VIT_06s0004g06830  -  Asparagine synthetase
## VIT_02s0025g04330  -  Thaumatin VVTL1 [Vitis vinifera]
## VIT_11s0016g02700  -  IAA-amino acid hydrolase 2 (ILL2)
## VIT_04s0008g02660  -  Calmodulin-binding protein
## VIT_00s0125g00200  -  No hit
## VIT_07s0005g02710  -  Unknown protein
## VIT_08s0056g01240  -  No hit
## VIT_05s0049g00520  -  No hit
## VIT_09s0096g00660  -  C2-HC type zinc finger protein C.e-MyT1
## VIT_07s0005g01350  -  Universal stress protein (USP) family protein
## VIT_01s0244g00110  -  Unknown protein
```

```
## VIT_18s0001g06070  -  Unknown protein
## VIT_14s0030g01210  -  R protein disease resistance protein
## VIT_14s0060g02300  -  Hypoxia-responsive
## VIT_02s0025g01350  -  Primase polypeptide 1
## VIT_17s0000g10090  -  Xanthine/uracil/vitamin C permease
## VIT_08s0040g00470  -  Calmodulin-7 (CAM7)
## VIT_18s0001g00990  -  Calcium Dependent Protein Kinase (VvCPK15)
## VIT_08s0007g07670  -  NAC domain-containing protein (VvNAC60)
## VIT_00s1608g00010  -  Ubiquitin-specific protease 23 (UBP23)
## VIT_08s0040g00510  -  ZIFL2 (zinc induced facilitator-like 2)
## VIT_16s0013g00440  -  No hit
## VIT_03s0088g01180  -  Proline iminopeptidase
## VIT_16s0013g00450  -  No hit
## VIT_06s0061g00040  -  Hexokinase 6
## VIT_18s0001g03640  -  No hit
## VIT_08s0007g05000  -  S-adenosylmethionine synthetase
## VIT_08s0040g01950  -  Zinc finger (C3HC4-type ring finger)
## VIT_18s0001g13250  -  Thioredoxin H-type 1
## VIT_11s0037g00390  -  Unknown protein
## VIT_04s0008g04990  -  Potassium channel (VvK1.2)
## VIT_08s0040g00490  -  Caspase
## VIT_09s0002g01870  -  Unknown protein
## VIT_01s0010g02460  -  Glyceraldehyde-3-phosphate dehydrogenase, cytosolic 3
## VIT_06s0004g03340  -  Ribose-5-phosphate isomerase
## VIT_14s0030g01200  -  R protein disease resistance protein
## VIT_00s0313g00070  -  putative MADS-box Short Vegetal Phase 1 (VviSVP1)
## VIT_04s0044g01760  -  Unknown
## VIT_06s0004g01140  -  GRIK1 (geminivirus rep  interacting  kinase 1)
## VIT_10s0116g00570  -  Nuclear transport factor 2B
## VIT_08s0007g08620  -  No hit
## VIT_03s0017g01450  -  No hit
## VIT_13s0073g00490  -  Serine/threonine-protein phosphatase BSL3
## VIT_17s0000g03630  -  No hit
## ENSRNA049469468  -
## ENSRNA049468949  -
## VIT_14s0083g00940  -  Auxin-independent growth promoter
## VIT_04s0023g03600  -  SEN1 (dark inducible 1)
## VIT_04s0023g03670  -  ERD7 (EARLY-responsive TO dehydration 7)
## VIT_04s0008g01840  -  TT2 (transparent testa 2)
## VIT_07s0005g00750  -  Sucrose synthase
## VIT_13s0073g00440  -  BSU1-like protein 3 BSL3
## ENSRNA049468680  -
## VIT_14s0108g01450  -  Calmodulin-binding
## VIT_05s0020g04570  -  CBL-interacting protein kinase 7 (CIPK7)
## VIT_10s0003g01020  -  Unknown protein
## VIT_10s0116g00550  -  Oligosaccharide transporter OST3/OST6
## VIT_11s0016g02380  -  1-aminocyclopropane-1-carboxylate oxidase 2
## VIT_02s0025g04110  -  MAPKKK HA-tagged protein kinase
## VIT_19s0014g03750  -  No hit
## VIT_19s0014g03810  -  No hit
## VIT_14s0219g00210  -  Ubiquitin family
## VIT_08s0105g00370  -  myb family
## VIT_13s0084g00050  -  Cysteine proteinase inhibitor
## VIT_14s0066g01060  -  Polygalacturonase GH28
## VIT_14s0066g01600  -  NHL repeat-containing protein
```

```
## VIT_02s0087g00780  -  No hit
## VIT_09s0002g06420  -  Lactoylglutathione lyase
## VIT_18s0041g00430  -  Patellin-1
## VIT_01s0127g00680  -  SRO2 (similar to rcd one 2)
## VIT_04s0008g04210  -  GLB3 (2-on-2 hemoglobin like gene 3)
## VIT_18s0041g00630  -  Proton-dependent oligopeptide transport (POT) family protein
## ENSRNA049996412  -
## VIT_18s0041g00600  -  Proton-dependent oligopeptide transport (POT) family protein
## VIT_02s0025g03630  -  Cu2+-exporting ATPase HMA5 (heavy metal ATPase 5)
## VIT_18s0001g14520  -  Unknown protein
## VIT_18s0122g00350  -  DC1 domain-containing protein
## VIT_05s0029g00190  -  Glucan endo-1,3-beta-glucosidase 4 precursor
## VIT_18s0001g00360  -  Dehydrin (VvDHN2)
## ENSRNA049468692  -
## VIT_02s0012g01040  -  NAC domain-containing protein (VvNAC13)
```

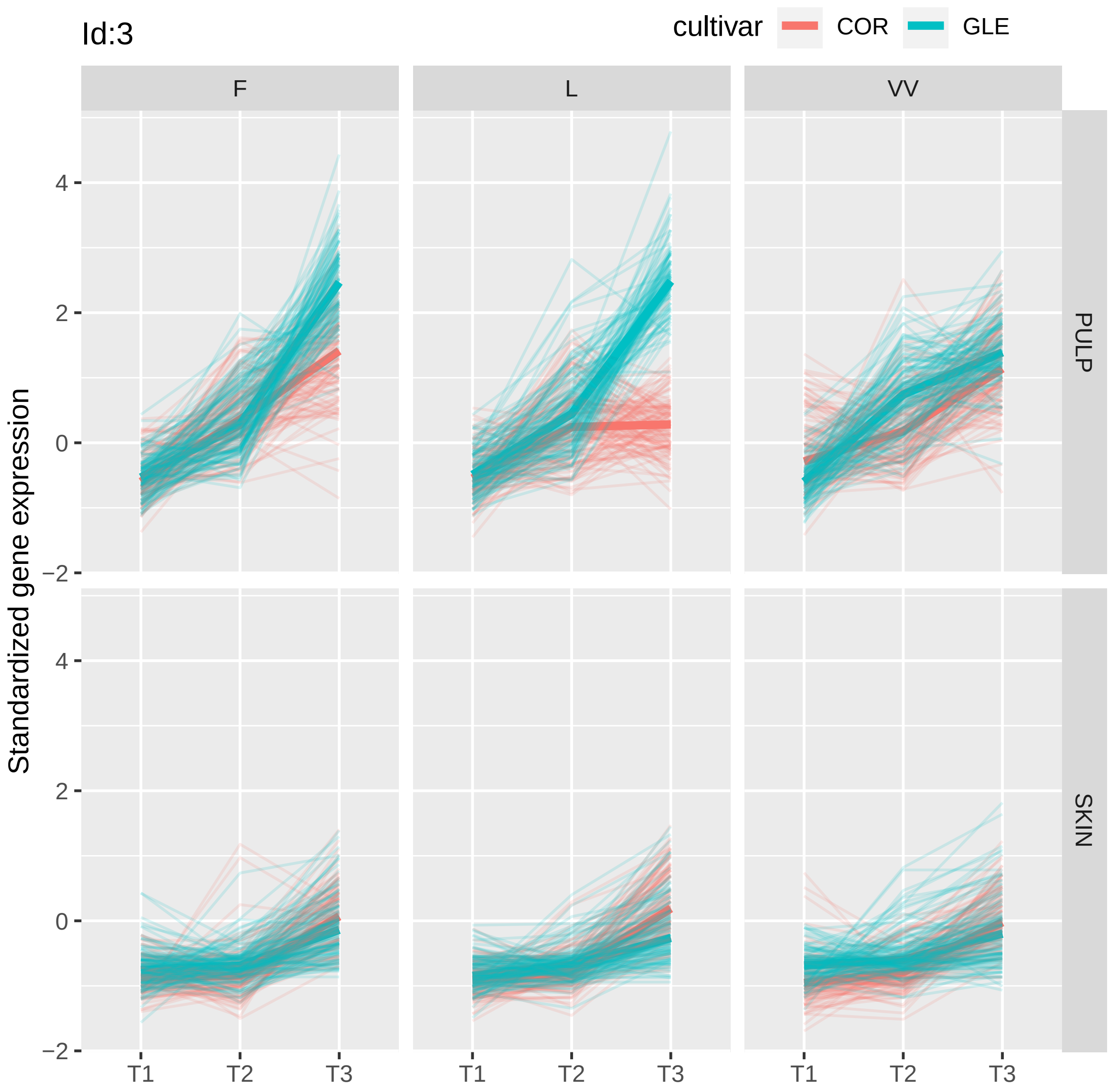

Id:3
cultivar
COR
GLE
F
L
VV
PULP
SKIN
Standardized gene expression
4
2
0
−2
T1
T2
T3

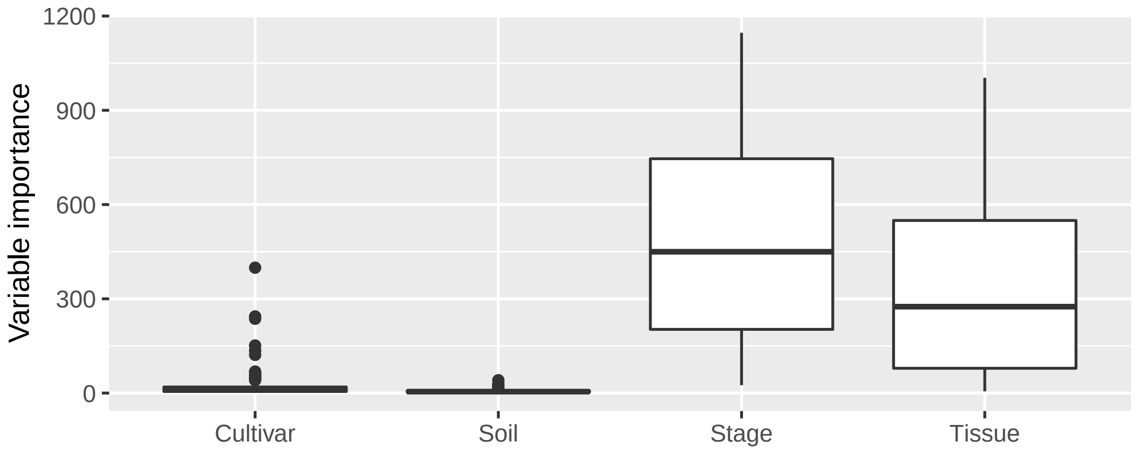

Variable importance
1200
900
600
300
0
Cultivar
Soil
Stage
Tissue

Cluster no. 35

```
## Number of genes in the cluster: 45
## Homogeneity Index:    0.85
## Variable importance for Stage:     Rank =  35  - Median =  434.7
## Variable importance for Cultivar:  Rank =  27  - Median =  148.2
## Variable importance for Tissue:    Rank =  77  - Median =  29.82
## Variable importance for Soil:      Rank =  54  - Median =   5.46
##
## Gene ID               Gene Annotation
## VIT_16s0050g01600  -  UDP-glycosyltransferase 88A4
## VIT_13s0106g00550  -  flavonol 3-sulfotransferase
## VIT_15s0024g01760  -  R protein L6
## VIT_18s0001g13960  -  SAUR_E
## VIT_12s0035g01030  -  Unknown protein
## VIT_18s0001g03610  -  Auxin-independent growth promoter
## VIT_02s0012g01350  -  No hit
## VIT_14s0068g01540  -  PBS1 (avrPphB susceptible 1)
## VIT_16s0022g00870  -  Invertase/pectin methylesterase inhibitor
## VIT_04s0008g01850  -  Trihelix DNA-binding protein (GT2)
## VIT_07s0031g02790  -  Unknown
## VIT_06s0004g07030  -  MRH1 (morphogenesis of root hair 1)
## VIT_11s0016g05170  -  Nitrate transporter
## VIT_07s0104g01680  -  Dephospho-CoA kinase
## VIT_18s0001g00720  -  Protein kinase
## VIT_05s0049g00230  -  E8 protein
## VIT_19s0014g00460  -  PMR5 (powdery mildew resistant 5)
## VIT_19s0014g04820  -  V-type H+-transporting ATPase 21kDa proteolipid subunit
## VIT_19s0085g00740  -  ZIP family transporter
## VIT_09s0002g08590  -  Pentatricopeptide (PPR) repeat-containing protein
## VIT_14s0030g00200  -  Sugar transporter ERD6-like 5
## VIT_16s0039g00430  -  Cyclin-dependent protein kinase CDT1A
## VIT_18s0041g01640  -  Leucine-rich repeat family protein
## VIT_07s0104g01400  -  Glutaredoxin
## VIT_01s0026g00630  -  N-hydroxythioamide S-beta-glucosyltransferase
## VIT_08s0007g08780  -  Unknown protein
## VIT_11s0037g00530  -  Cellulose synthase IRX3
## VIT_11s0118g00420  -  Heparanase protein 3 precursor
## VIT_01s0010g01380  -  Ankyrin repeat
## VIT_10s0003g05160  -  Wall-associated kinase 3 (WAK3)
## VIT_13s0067g00750  -  R protein  PRF  disease resistance protein
## VIT_13s0067g00740  -  R protein  PRF  disease resistance protein
## VIT_03s0038g02030  -  Amidase
## VIT_09s0002g06680  -  Embryo-specific 3
## VIT_02s0025g04670  -  Senescence-inducible chloroplast stay-green protein 2
## VIT_02s0025g03810  -  Potassium channel AKT5
## VIT_12s0028g02280  -  Calcium-dependent protein kinase 13 CPK13
## VIT_14s0128g00790  -  Lipoxygenase (LOX1)
## VIT_12s0059g01070  -  Chromosome associate protein subunit H
## VIT_13s0047g00990  -  Cinnamyl alcohol dehydrogenase
## VIT_19s0085g00790  -  Zinc transporter ZIP5
## VIT_12s0034g01270  -  RPS4 (resistant to p. syringae 4)
## VIT_13s0067g03640  -  R protein MLA10
## VIT_13s0147g00150  -  TIR-NBS-LRR-TIR disease resistance protein
## VIT_13s0101g00300  -  R protein MLA10
```

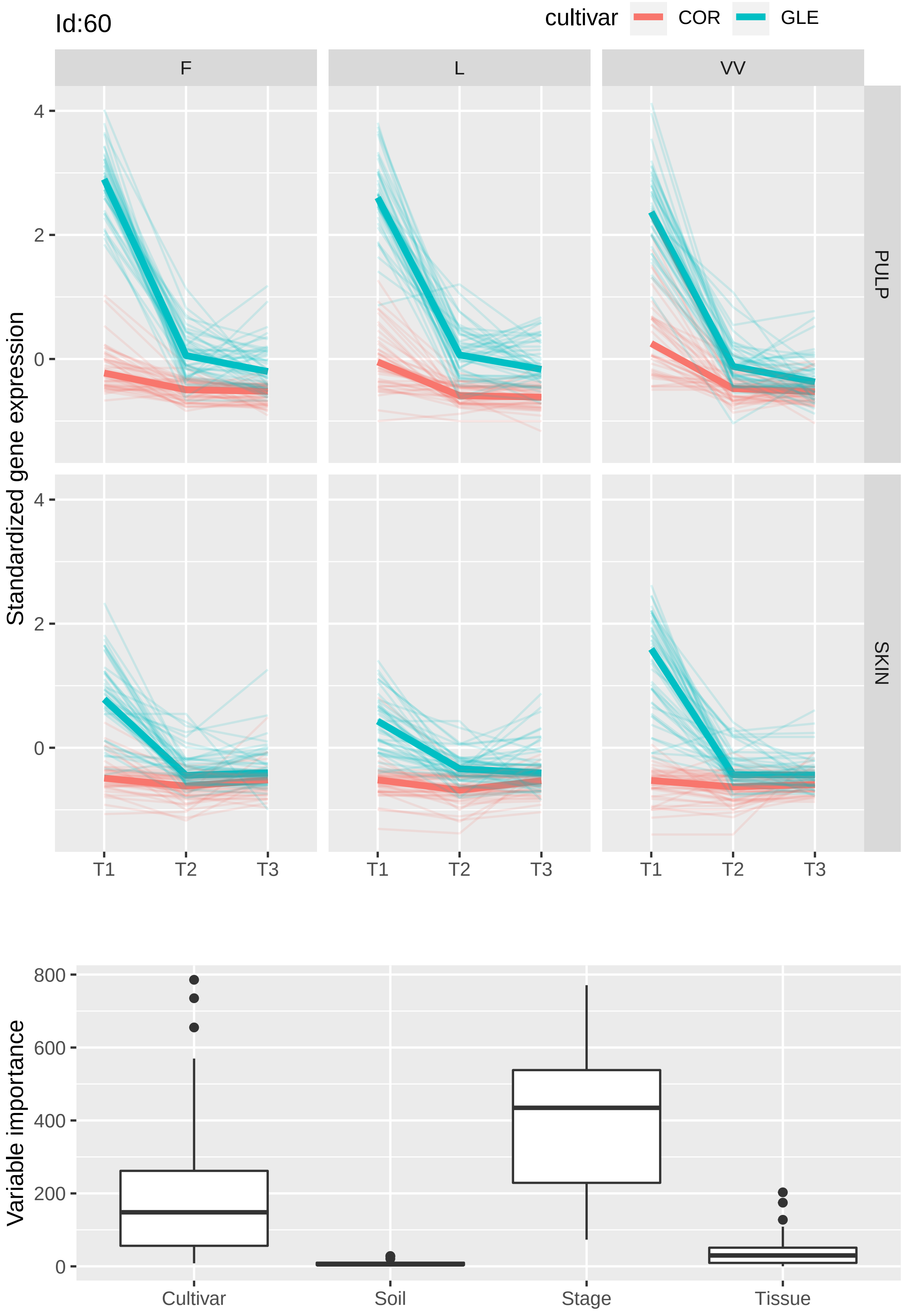
Id:60
cultivar
COR
GLE
F
L
VV
PULP
SKIN
Standardized gene expression
4
2
0
T1
T2
T3
Variable importance
800
600
400
200
0
Cultivar
Soil
Stage
Tissue

Cluster no. 36

```
## Number of genes in the cluster: 79
## Homogeneity Index:    0.82
## Variable importance for Stage:     Rank =  36  - Median =  400.6
## Variable importance for Cultivar:  Rank =  50  - Median =  46.79
## Variable importance for Tissue:    Rank =  31  - Median =  295.1
## Variable importance for Soil:      Rank =  32  - Median =   6.34
##
## Gene ID                Gene Annotation
## VIT_12s0035g00250  -  EMB2758
## VIT_15s0048g01740  -  Growth-regulating factor 9
## VIT_15s0046g01510  -  BTB/POZ domain-containing protein
## VIT_14s0030g02200  -  Basic Leucine Zipper Transcription Factor (VvbZIP38)
## VIT_05s0124g00510  -  Oxalyl-CoA decarboxylase [Vitis vinifera]
## VIT_18s0001g14730  -  Glutaredoxin
## VIT_12s0059g00900  -  Pentatricopeptide repeat
## VIT_18s0001g14250  -  Unknown protein
## VIT_00s0291g00100  -  ABC Transporter (VvTAP1 - VvABCB21)
## VIT_05s0077g00850  -  Ubiquitin-conjugating enzyme E2 O
## VIT_16s0039g00730  -  DNA polymerase delta, subunit D
## VIT_18s0001g02490  -  PAB3 (poly(A) binding protein 3)
## VIT_09s0002g06810  -  Binding
## VIT_04s0008g01180  -  Esterase
## VIT_11s0016g00780  -  Unknown protein
## VIT_05s0077g02320  -  Hypoxia-responsive
## VIT_04s0008g05420  -  SPX2 (SYG1/Pho81/XPR1) domain-containing protein SPX2
## VIT_19s0015g01810  -  Unknown protein
## VIT_07s0031g01200  -  RNase L inhibitor protein
## VIT_14s0060g01010  -  basic helix-loop-helix (bHLH) family
## VIT_13s0064g01210  -  Zf A20 and AN1 domain-containing stress-associated protein 2
## VIT_00s1430g00010  -  Protein kinase
## VIT_00s1916g00010  -  DNA binding
## VIT_08s0217g00030  -  Ran-binding protein 1 RanBP1
## VIT_03s0038g00580  -  GATA transcription factor 25
## VIT_06s0009g02120  -  Nuclear transcription factor Y subunit B related
## VIT_02s0012g01630  -  Transmembrane protein 41B
## VIT_11s0016g05480  -  Clathrin assembly protein 2
## VIT_14s0006g02280  -  Cyclin-dependent kinase D1;2
## VIT_09s0002g00910  -  Exocyst subunit EXO70 D1
## VIT_07s0031g01880  -  Ubiquitin-specific protease 27
## VIT_13s0158g00230  -  IMP dehydrogenase/GMP reductase
## VIT_03s0063g00350  -  Unknown
## VIT_03s0063g00340  -  No hit
## VIT_07s0104g00860  -  WD40
## VIT_05s0020g03060  -  Cyclin-T1-4
## VIT_11s0016g03550  -  NADP adrenodoxin-like ferredoxin reductase
## VIT_04s0008g03030  -  RNA recognition motif (RRM)-containing
## VIT_16s0022g01250  -  Unknown protein
## VIT_05s0051g00640  -  Purple acid phosphatase 23- ATPAP23/PAP23
## VIT_10s0071g00740  -  No hit
## VIT_15s0107g00170  -  Dynamin-like 3
## VIT_18s0001g14340  -  No hit
## VIT_02s0025g01410  -  Acyl-CoA synthetase long-chain member 2
## VIT_05s0136g00390  -  Unknown protein
## VIT_08s0007g01150  -  Unc51-like kinase
```

```
## VIT_12s0035g00300  -  Carbohydrate transmembrane transporter SFP1
## VIT_00s0291g00080  -  Unknown protein
## VIT_10s0003g01500  -  Zinc finger (C3HC4-type ring finger)
## VIT_00s0936g00010  -  Protein kinase
## VIT_12s0059g01480  -  Lipase family
## VIT_04s0023g00220  -  Zfwd2 protein (ZFWD2)
## VIT_18s0001g06390  -  Auxin-independent growth promoter
## VIT_18s0001g07300  -  MADS box interactor
## VIT_05s0020g00620  -  Kinesin phragmoplast orienting kinesin 1
## VIT_17s0000g06000  -  basic helix-loop-helix (bHLH) family
## VIT_18s0001g10430  -  Trimethylguanosine synthase
## VIT_06s0004g06580  -  PfkB-type carbohydrate kinase
## VIT_14s0171g00080  -  Hcr2-0B
## VIT_13s0019g02440  -  Mitochondrial substrate carrier family protein
## VIT_07s0005g00120  -  Unknown
## VIT_05s0020g00260  -  Unknown protein
## VIT_19s0014g02860  -  Cellulase
## VIT_08s0056g01040  -  MATE efflux family protein
## VIT_15s0021g02490  -  Protein transport protein Sec61 subunit beta
## VIT_07s0031g00470  -  DNA polymerase alpha
## VIT_18s0001g15010  -  F-box and leucine-rich repeat protein 1
## VIT_18s0041g02120  -  R protein L6
## VIT_16s0098g00800  -  Copper-binding family protein
## VIT_03s0038g03130  -  Flavin containing monooxygenase 3
## VIT_10s0523g00020  -  Tyrosine specific protein phosphatase
## VIT_06s0004g04100  -  ABC Transporter (VvMDR13 - VvABCB13)
## VIT_06s0004g07560  -  Ribosomal protein S29 28S
## VIT_12s0028g00940  -  Electron transfer flavoprotein alpha-subunit precursor (Alpha-ETF)
## VIT_02s0025g05120  -  ACT domain-containing protein (ACR8)
## VIT_19s0015g00860  -  Oligopeptide transporter 1
## VIT_02s0025g04620  -  Phospholipase D beta 1
## VIT_01s0026g02210  -  Unknown protein
## VIT_01s0150g00390  -  Minichromosome maintenance protein 2
```

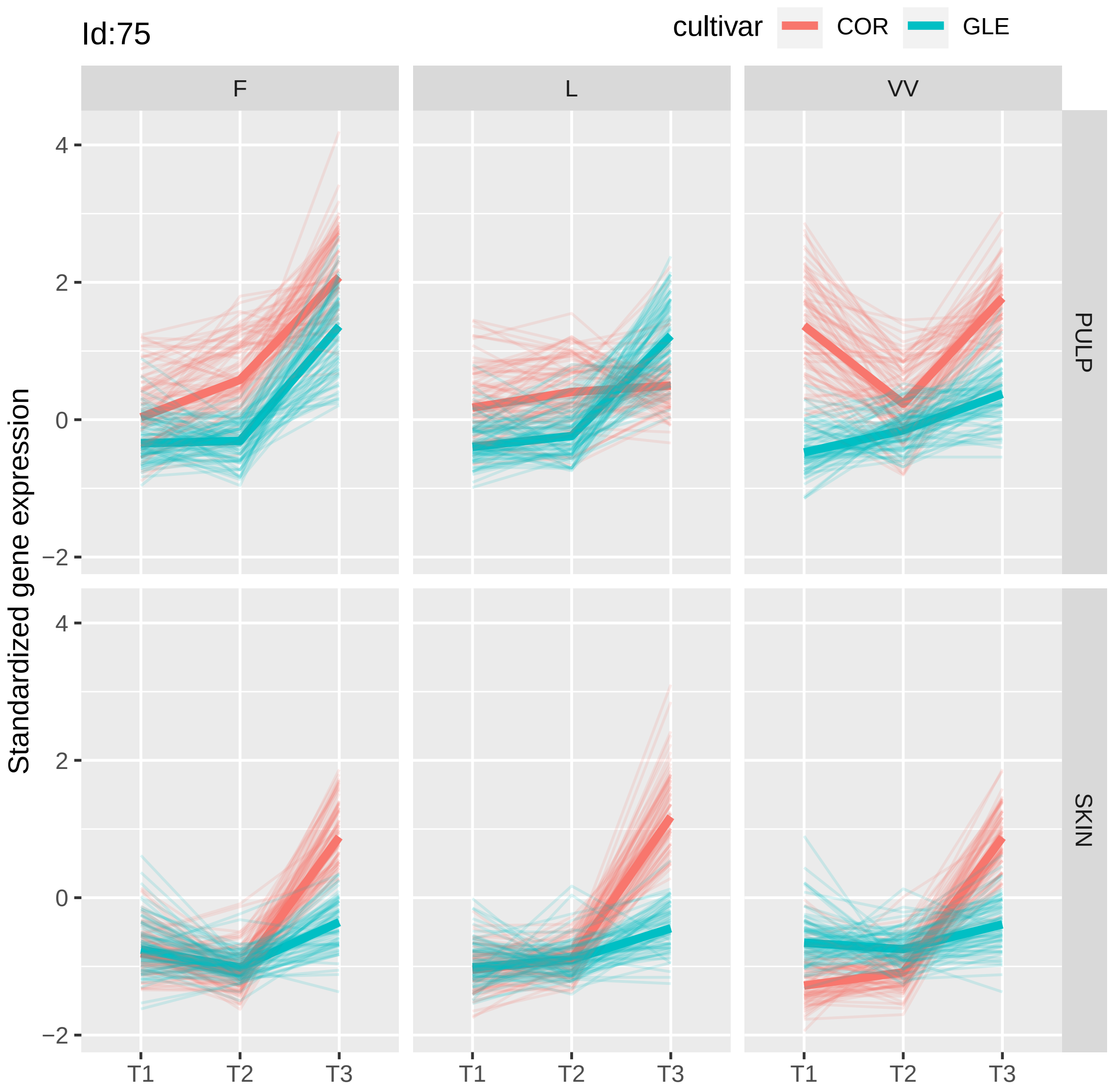
Id:75
cultivar
COR
GLE
F
L
VV
PULP
SKIN
Standardized gene expression
4
2
0
−2
T1
T2
T3

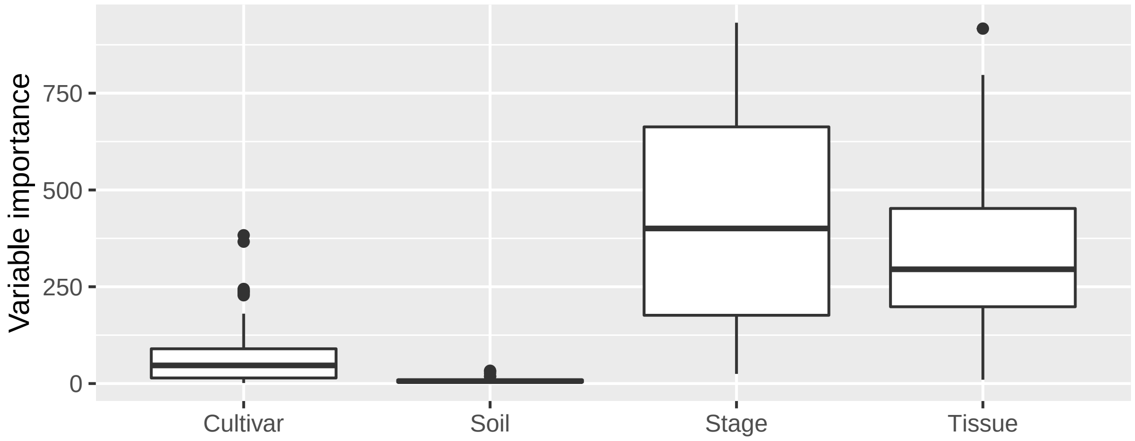
Variable importance
750
500
250
0
Cultivar
Soil
Stage
Tissue

Cluster no. 37

```
## Number of genes in the cluster: 78
## Homogeneity Index:    0.89
## Variable importance for Stage:     Rank =  37  - Median =  399.4
## Variable importance for Cultivar:  Rank =  40  - Median =  63.73
## Variable importance for Tissue:    Rank =  94  - Median =  12.99
## Variable importance for Soil:      Rank =  14  - Median =    8.5
##
## Gene ID                Gene Annotation
## VIT_12s0057g01350  -  Constans-like 9
## VIT_18s0041g01880  -  putative MADS-box Agamous 3 (VviAG3)
## VIT_14s0108g00890  -  Beta-1,3-galactosyltransferase
## VIT_18s0001g13680  -  Histone H3
## VIT_18s0072g00260  -  Ethylene-responsive transcription factor related to APETALA2 6
## VIT_06s0004g03890  -  Histone H4
## VIT_00s0253g00080  -  Methyl jasmonate esterase
## VIT_07s0005g01450  -  Basic Leucine Zipper Transcription Factor (VvbZIP22)
## VIT_01s0137g00680  -  No hit
## VIT_14s0128g00390  -  Mitochondrial FAD carrier
## VIT_00s0527g00010  -  Cig3
## VIT_06s0004g04830  -  Subtilisin protease C1
## VIT_05s0020g04800  -  Histone H1
## VIT_01s0137g00670  -  Quinolinate synthase
## VIT_06s0004g04950  -  Scarecrow-like transcription factor 14 SCL14
## VIT_06s0004g00160  -  Pentatricopeptide (PPR) repeat-containing protein
## VIT_13s0019g00760  -  Histone H4
## ENSRNA049996497  -
## VIT_00s0301g00090  -  NDA2 (alternative NAD(P)H dehydrogenase 2)
## VIT_14s0060g02360  -  Histone H2A.4 HTA12
## VIT_08s0007g00040  -  Histone H4
## VIT_00s0253g00100  -  Methyl jasmonate esterase
## VIT_02s0025g02600  -  Glyoxal oxidase
## VIT_14s0066g01540  -  Serine/threonine kinase SYMRK
## VIT_08s0007g05500  -  Condensin subunit 1
## VIT_04s0023g01890  -  Unknown protein
## VIT_01s0026g00580  -  Receptor protein kinase
## VIT_03s0038g01750  -  Disease resistance RPP8 2
## VIT_04s0008g02980  -  Lupeol synthase
## VIT_02s0025g00810  -  Cation/hydrogen exchanger (CHX18)
## VIT_00s0240g00050  -  Glutathione S-transferase 8 GSTU19
## VIT_05s0094g00550  -  Stearoyl-acyl-[acyl-carrier-protein] desaturase
## VIT_10s0003g03780  -  Unknown protein
## VIT_19s0014g02950  -  No hit
## VIT_10s0003g01180  -  Ketol-acid reductoisomerase
## VIT_18s0001g11520  -  flavonoid 3-monooxygenase
## VIT_08s0007g05830  -  No hit
## VIT_10s0003g03490  -  GA 2-oxidase
## VIT_00s0640g00020  -  Cig3
## VIT_04s0008g06930  -  Transposase, IS4
## VIT_11s0016g05010  -  Lactoylglutathione lyase
## VIT_05s0077g01310  -  Aldo-keto reductase
## VIT_16s0148g00360  -  PR5K (PR5-like receptor kinase)
## VIT_16s0100g00240  -  Mandelonitrile lyase-like protein
## VIT_17s0000g05060  -  Phytochelatin synthetase
## VIT_05s0029g00240  -  Pentatricopeptide (PPR) repeat-containing
```

```
## VIT_10s0003g05820  -  SKP1-like 1 ASK1
## VIT_05s0077g01140  -  Basic Leucine Zipper Transcription Factor (VvbZIP14)
## VIT_05s0094g00740  -  Stearoyl-acyl-[acyl-carrier-protein] desaturase
## VIT_14s0006g02590  -  Glutamine amidotransferase
## VIT_18s0001g07610  -  EMB1674 (embryo defective 1674) kinase interacting family protein
## VIT_07s0005g03490  -  VHS domain-containing protein
## VIT_09s0096g00520  -  Coniferyl alcohol acyltransferase
## VIT_14s0108g00710  -  ABC Transporter (VvSMC1)
## VIT_19s0014g04790  -  Organic cation/carnitine transporter4
## VIT_18s0076g00340  -  CYP707A3
## VIT_06s0004g00020  -  NAC domain-containing protein (VvNAC44)
## VIT_16s0100g00250  -  Mandelonitrile lyase-like protein
## VIT_00s0409g00060  -  Cysteine-rich receptor-like protein kinase 25
## VIT_02s0012g00530  -  Ribose-phosphate pyrophosphokinase 1
## VIT_01s0026g01740  -  CBS
## VIT_18s0001g14420  -  Unknown
## VIT_07s0005g01040  -  Glycosyl transferase family 8 protein
## VIT_15s0045g00090  -  ABC transporter C member 9
## VIT_19s0090g01250  -  Jasmonate O-methyltransferase
## VIT_12s0028g00670  -  Zinc knuckle
## VIT_05s0077g01590  -  s9_Pathogenesis protein 10 [Vitis vinifera]
## VIT_07s0005g00870  -  Erg-1
## VIT_09s0054g00570  -  No hit
## VIT_19s0177g00310  -  Tryptophan synthase beta chain 1
## VIT_06s0009g03420  -  C2 domain-containing protein
## VIT_08s0007g05760  -  No hit
## VIT_07s0005g00140  -  Peripheral-type benzodiazepine receptor
## VIT_12s0059g00580  -  Rho GDP-dissociation inhibitor 2
## VIT_19s0014g04810  -  Vetispiradiene synthase (VvTPS26), Cubebol/d-Cadinene syn
## VIT_08s0007g07730  -  CYP93A1 2-hydroxyisoflavanone synthase
## VIT_08s0040g01620  -  ABC Transporter (VvWBC22 - VvABCG22)
## VIT_12s0059g01090  -  Early-responsive to dehydration
```

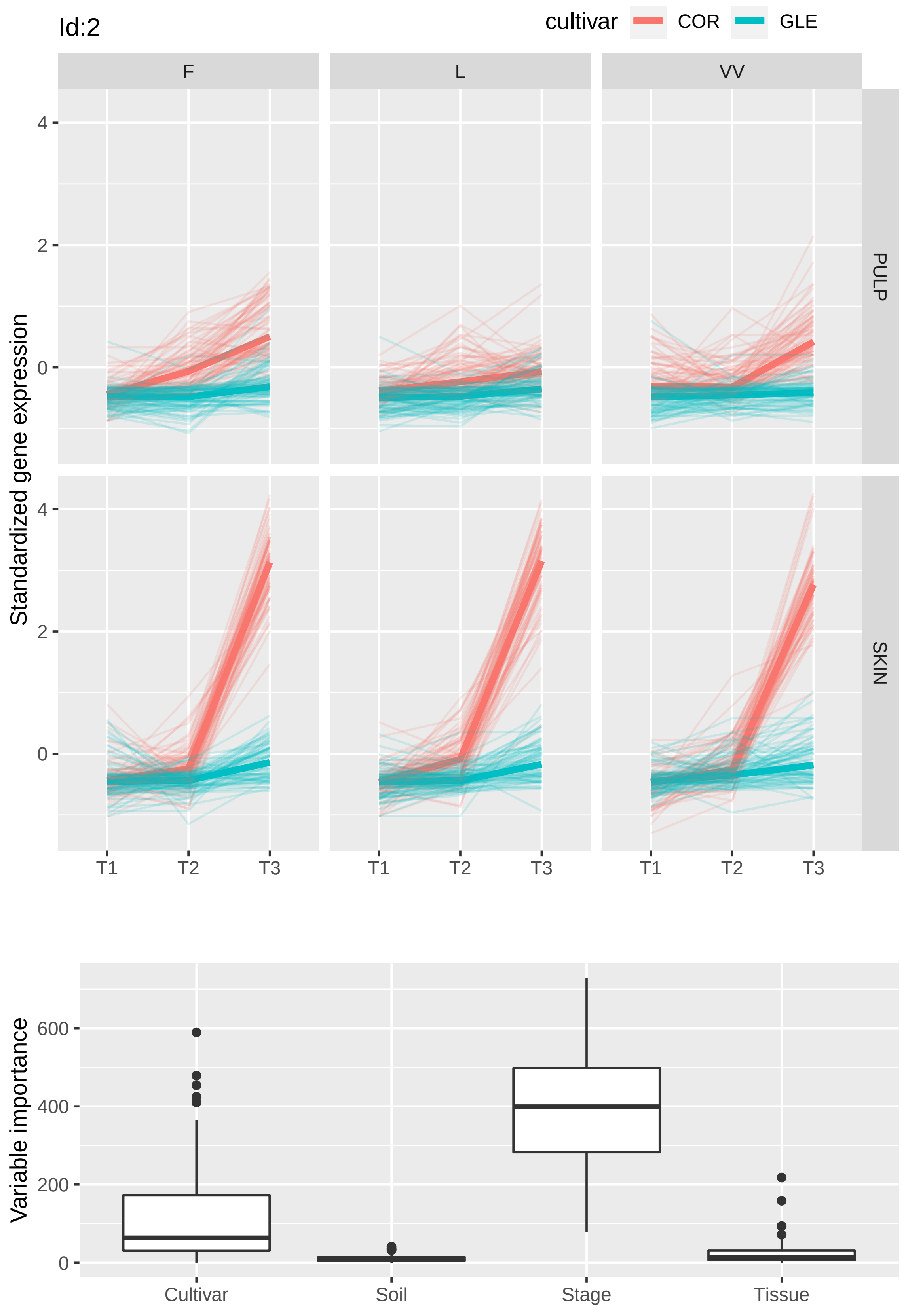
Id:2
cultivar
COR
GLE
F
L
VV
PULP
SKIN
Standardized gene expression
4
2
0
T1
T2
T3
Variable importance
600
400
200
0
Cultivar
Soil
Stage
Tissue

Cluster no. 38

```
## Number of genes in the cluster: 43
## Homogeneity Index:    0.81
## Variable importance for Stage:     Rank =  38  - Median =  397.8
## Variable importance for Cultivar:  Rank =  14  - Median =  325.1
## Variable importance for Tissue:    Rank =  99  - Median =   8.58
## Variable importance for Soil:      Rank =  68  - Median =   4.84
##
## Gene ID               Gene Annotation
## VIT_18s0001g12210  -  CYP721A1
## VIT_18s0001g01160  -  Alcohol dehydrogenase
## VIT_15s0024g01170  -  No hit
## VIT_05s0094g01250  -  Allyl alcohol dehydrogenase
## VIT_08s0040g00690  -  Unknown
## VIT_13s0064g00140  -  R protein disease resistance protein
## VIT_13s0064g01370  -  Polygalacturonase inhibiting protein 1 PGIP1
## VIT_19s0027g01070  -  R protein disease resistance protein
## VIT_19s0027g01060  -  Tetratricopeptide repeat (TPR)-containing
## VIT_13s0158g00290  -  R protein MLA10
## VIT_12s0059g00990  -  Cellulose synthase CSLB04
## VIT_05s0020g02770  -  Unknown protein
## VIT_10s0092g00590  -  Leucine-rich repeat family
## VIT_00s0193g00080  -  Ankyrin
## VIT_00s0958g00020  -  FK506-binding protein genes family (VvFKBPb)
## VIT_12s0035g00880  -  Lactoylglutathione lyase
## VIT_00s0274g00060  -  Glycine-rich protein
## VIT_13s0067g03140  -  WRKY Transcription Factor (VvWRKY41)
## VIT_18s0001g05780  -  Leucine-rich repeat family protein
## VIT_08s0007g02240  -  Calcium/proton exchanger CAX3
## VIT_15s0046g01170  -  dihydroflavonol 4-reductase (dihydrokaempferol 4-reductase)
## VIT_05s0020g04750  -  Ankyrin repeat protein
## VIT_19s0135g00250  -  Ankyrin repeat protein
## VIT_17s0000g04040  -  No hit
## VIT_19s0014g02410  -  L-ascorbate peroxidase 1, cytosolic (APX1)
## VIT_08s0007g06080  -  Beta 1-3 glucanase [Vitis vinifera]
## VIT_08s0040g02350  -  Xylan synthase
## VIT_15s0046g00390  -  Seven in absentia SINA4
## VIT_08s0040g02340  -  Xylan synthase
## VIT_18s0001g14320  -  Unknown protein
## VIT_03s0180g00140  -  Acetyl xylan esterase AxeA
## VIT_16s0098g01910  -  Germin-like protein subfamily 3 member 4
## VIT_03s0088g00810  -  Pathogenesis-related protein 1 precursor (PRP 1)
## VIT_19s0015g00660  -  E3 ubiquitin-protein ligase HUWE1
## VIT_12s0035g00540  -  Disease resistance protein (NBS-LRR class)
## VIT_00s2705g00010  -  Unknown
## VIT_18s0041g00480  -  Proton-dependent oligopeptide transport (POT) family protein
## VIT_07s0095g00190  -  No hit
## VIT_10s0003g02490  -  SRG1 (senescence-related gene 1) oxidoreductase
## VIT_02s0033g00420  -  Ribosomal protein S28 (RPS28C) 40S
## VIT_13s0067g01830  -  Steroid 5alpha-reductase
## VIT_08s0040g02970  -  Protein phosphatase 2C
## VIT_05s0020g04360  -  Ca2+-ATPase 13 ACA13, plasma membrane
```

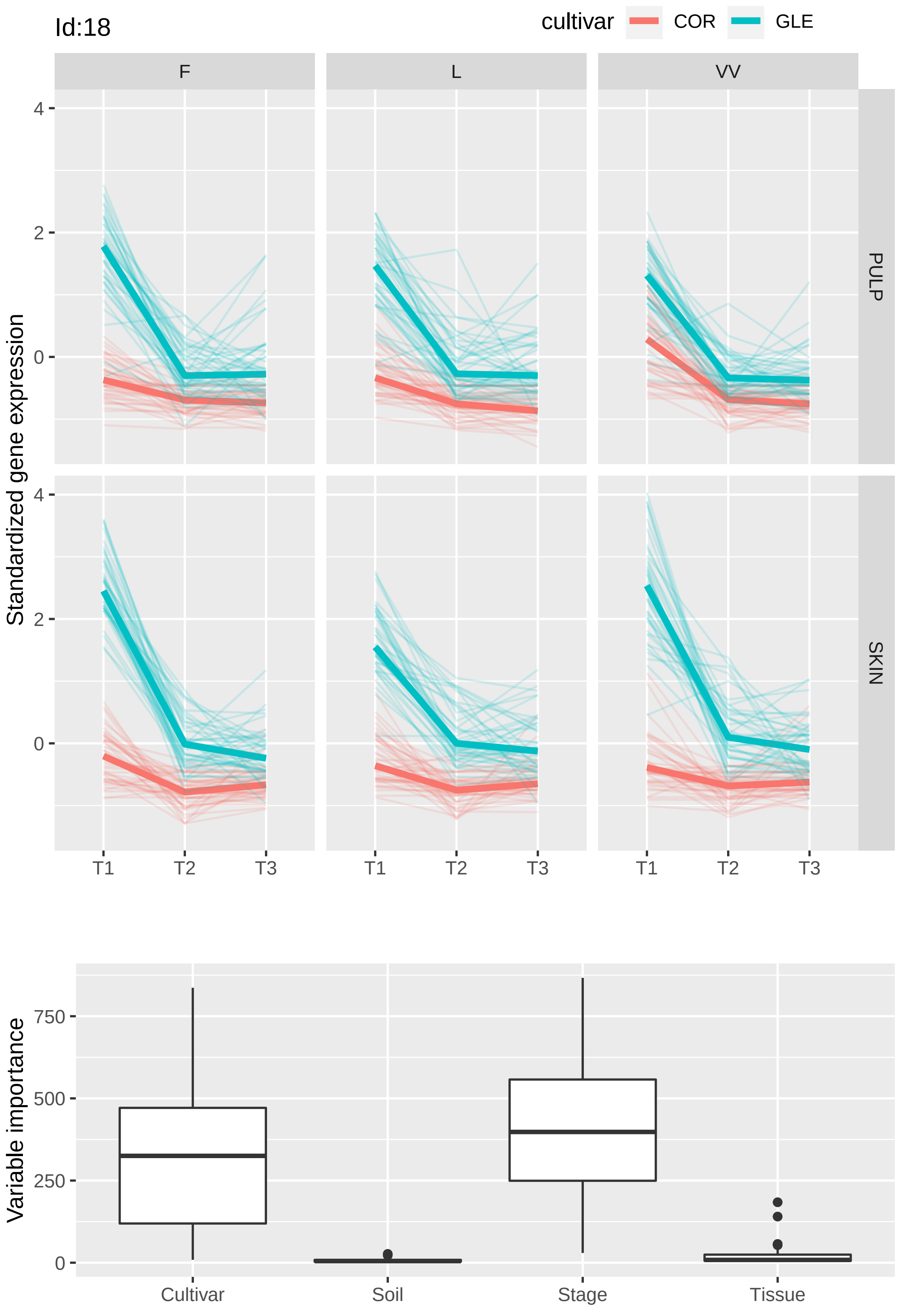
Id:18
cultivar
COR
GLE
F
L
VV
PULP
SKIN
Standardized gene expression
4
2
0
T1
T2
T3
Variable importance
750
500
250
0
Cultivar
Soil
Stage
Tissue

Cluster no. 39

```
## Number of genes in the cluster: 122
## Homogeneity Index:    0.83
## Variable importance for Stage:     Rank =  39  - Median =  389.9
## Variable importance for Cultivar:  Rank =  36  - Median =   84.6
## Variable importance for Tissue:    Rank =  64  - Median =  68.19
## Variable importance for Soil:      Rank =  65  - Median =   5.03
##
## Gene ID               Gene Annotation
## VIT_13s0073g00690  -  Unknown protein
## VIT_04s0023g03650  -  Galactose mutarotase-like
## VIT_00s0207g00130  -  Pathogenesis-related protein 1 precursor (PRP 1)
## VIT_00s0269g00150  -  IMP dehydrogenase/GMP reductase
## VIT_16s0050g01090  -  ß-carotene hydroxylase (BCH2) (VvBCH2)
## VIT_06s0061g00670  -  Salutaridine reductase
## VIT_14s0068g00030  -  feronia receptor-like kinase
## VIT_01s0011g05200  -  Phosphoethanolamine N-methyltransferase 1
## VIT_06s0080g00570  -  Unknown protein
## VIT_18s0041g00550  -  Proton-dependent oligopeptide transport (POT) family protein
## VIT_06s0061g00640  -  Salutaridine reductase
## VIT_14s0083g00110  -  Allene oxide cyclase (jasmonates from fatty acids)
## VIT_08s0056g00780  -  MATE efflux family protein
## VIT_01s0150g00320  -  Nudix hydrolase 13
## VIT_18s0001g06820  -  MATE efflux family protein ripening responsive
## VIT_14s0006g01620  -  myb domain protein 4 (VvMybC2-L3)
## VIT_18s0117g00390  -  far-red impaired responsive family protein
## VIT_11s0016g02260  -  Unknown
## VIT_17s0000g02990  -  MATE efflux family protein
## VIT_01s0026g00520  -  Nodulin MtN21 family
## VIT_05s0077g01240  -  Calmodulin-binding transcription activator 6
## VIT_02s0025g01260  -  NADPH HC toxin reductase
## VIT_05s0020g04020  -  Unknown protein
## VIT_00s0346g00080  -  Sinapyl alcohol dehydrogenase
## VIT_17s0000g07270  -  No hit
## VIT_02s0087g00720  -  Receptor protein kinase
## VIT_18s0001g02010  -  No hit
## VIT_01s0026g00500  -  Nodulin MtN21 family
## VIT_01s0026g02190  -  ABA-responsive protein (HVA22c)
## VIT_09s0002g05700  -  Phototropic-responsive NPH3
## VIT_00s0324g00020  -  Hydrolase, alpha/beta fold
## VIT_03s0063g00790  -  Carboxyesterase 13; CXE13
## VIT_01s0026g00550  -  Nodulin MtN21 family
## VIT_05s0020g04880  -  Seed specific protein Bn15D14A
## VIT_12s0034g02440  -  R protein MLA10
## VIT_04s0008g00440  -  Clavata1 receptor kinase (CLV1)
## VIT_05s0020g01640  -  Transcription termination factor mitochondrial mTERF
## VIT_14s0036g00120  -  RPS2 (resistant to p. syringae 2)
## VIT_16s0039g01770  -  Major pollen allergen Car b 1 isoforms 1A and 1B
## VIT_07s0141g00860  -  IFA binding protein
## VIT_04s0008g03550  -  Aquaporin TIP4;1
## VIT_17s0000g03890  -  Peptidyl-prolyl isomerase C
## VIT_03s0063g02490  -  Glucan endo-1,3-beta-glucosidase 7 precursor
## VIT_18s0001g06760  -  Clathrin assembly protein 16
## VIT_18s0001g11470  -  CyP82A3
## VIT_00s0269g00140  -  Ankyrin
```

```
## VIT_16s0039g00570  -  10-deacetylbaccatin III 10-O-acetyltransferase
## VIT_00s0322g00020  -  HHP4 (heptahelical protein 4)
## VIT_06s0004g01110  -  Cation efflux family protein MTPc3
## VIT_05s0020g03240  -  S-receptor kinase
## VIT_10s0071g00190  -  R protein disease resistance protein
## VIT_00s0214g00010  -  DnaJ homolog, subfamily C, member 13
## VIT_18s0001g11490  -  CYP82C1p
## VIT_16s0050g01980  -  EIX receptor 2
## VIT_01s0011g02030  -  Serine carboxypeptidase S10
## VIT_18s0001g02000  -  Zinc finger (C2H2 type) family
## VIT_02s0012g02340  -  CYP76C4
## VIT_00s0494g00010  -  Growth-regulating factor 1
## VIT_05s0062g01160  -  Pectinesterase family
## VIT_18s0041g00700  -  NAC domain-containing protein (VvNAC46)
## VIT_15s0048g01590  -  CYP76B1
## VIT_08s0032g00890  -  Alpha-L-arabinosidase
## VIT_09s0002g05930  -  Unknown protein
## VIT_16s0050g01900  -  Leucine-rich repeat protein kinase
## VIT_19s0015g02480  -  Ankyrin repeat
## VIT_16s0100g00620  -  Calmodulin-binding region IQD24
## VIT_09s0002g02990  -  FRK1 (FLG22-induced receptor-like kinase 1)
## VIT_18s0001g12900  -  S-adenosyl-L-methionine:salicylic acid carboxyl methyltransferase
## VIT_16s0050g01950  -  Disease resistance family protein / LRR family
## VIT_01s0011g02960  -  leucoanthocyanidin reductase 1 (VvLAR1) [Vitis vinifera] GENE ID: 1002
## VIT_11s0052g00410  -  Allyl alcohol dehydrogenase
## VIT_15s0021g00140  -  Speckle-type POZ protein-related
## VIT_08s0007g05240  -  Phosphatidylinositol-4-phosphate 5-kinase
## VIT_13s0158g00330  -  No hit
## VIT_01s0011g04730  -  Unknown protein
## VIT_19s0014g00520  -  RKF1 (receptor-like kinase in flowers 1)
## VIT_05s0077g01730  -  Unknown
## VIT_17s0000g02970  -  MATE efflux family protein
## VIT_05s0094g01080  -  Ankyrin protein kinase
## VIT_12s0057g00950  -  No hit
## VIT_08s0007g02100  -  Alpha-1,4-glycosyltransferase
## VIT_12s0059g02220  -  MATE efflux family protein
## VIT_05s0077g01820  -  Protein phosphatase 2C
## VIT_08s0007g00210  -  Unknown protein
## VIT_19s0014g05160  -  RPS2 (resistant to p. syringae 2)
## VIT_13s0156g00460  -  CC-NBS-LRR class
## VIT_05s0094g01160  -  Allyl alcohol dehydrogenase
## VIT_02s0025g01910  -  Cellulose synthase CSLG3
## VIT_16s0148g00220  -  Ser/Thr receptor-like kinase1
## VIT_08s0007g03050  -  E8 protein
## VIT_01s0011g05170  -  Major Latex Protein Family
## VIT_04s0008g03200  -  Lachrymatory factor synthase
## VIT_02s0087g01010  -  CRK10 (cysteine-rich RLK10)
## VIT_02s0033g01370  -  Sodium:solute symporter family protein
## VIT_09s0002g02050  -  1-deoxy-D-xylulose 5-phosphate synthase
## VIT_15s0045g00750  -  Rpp4 candidate 3
## VIT_00s0218g00010  -  Cinnamyl alcohol dehydrogenase
## VIT_04s0069g00730  -  Glutamate receptor protein
## VIT_13s0084g00130  -  CXE carboxylesterase
## VIT_18s0001g06790  -  Protein TRANSPARENT TESTA 12 (DDTFR18)
## VIT_05s0020g01240  -  Geranylgeranyl pyrophosphate synthase
```

```
## VIT_01s0011g04270  -  Receptor kinase homolog LRK10
## VIT_12s0035g01280  -  R protein disease resistance protein
## VIT_17s0000g04880  -  Dirigent protein
## VIT_07s0005g00410  -  Glycosyl hydrolase family 1 protein
## VIT_00s0469g00050  -  Cellulose synthase CSLE1
## VIT_17s0053g00040  -  Metal-nicotianamine transporter YSL5
## VIT_01s0011g04900  -  Serine carboxypeptidase-like 50 SCPL50 (
## VIT_10s0042g00250  -  R protein disease resistance protein
## VIT_04s0008g07250  -  Aspartyl protease
## VIT_06s0009g02750  -  No hit
## VIT_13s0158g00010  -  R protein MLA10
## VIT_05s0020g00890  -  ABC Transporter (VvMDR10 - VvABCB10)
## VIT_02s0025g04650  -  putative MADS-box Supressor of Constans overexpression 1c (VviSOC1c)
## VIT_09s0002g02900  -  Leucine-rich repeat protein kinase
## VIT_00s0358g00010  -  No hit
## VIT_05s0020g03310  -  Diamine oxidase
## VIT_09s0002g03030  -  FRK1 (FLG22-induced receptor-like kinase 1)
## VIT_03s0017g01750  -  ABC protein 6 non-intrinsic
## VIT_04s0008g06210  -  Nodulin
## VIT_06s0004g02390  -  Triacylglycerol lipase
## VIT_00s2704g00010  -  No hit
```

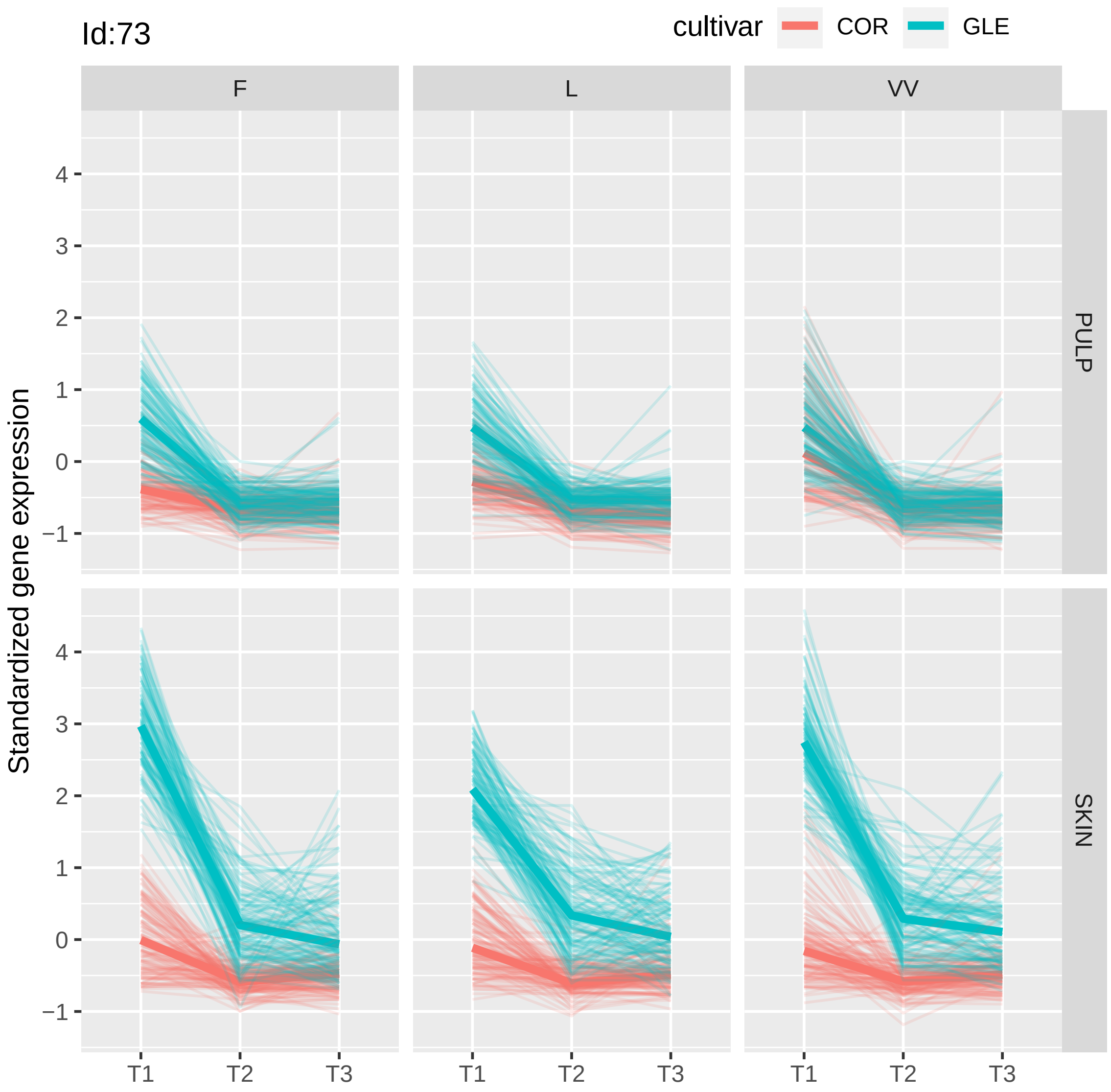
Id:73
cultivar COR GLE
F
L
VV
PULP
SKIN
Standardized gene expression
4
3
2
1
0
−1
T1
T2
T3

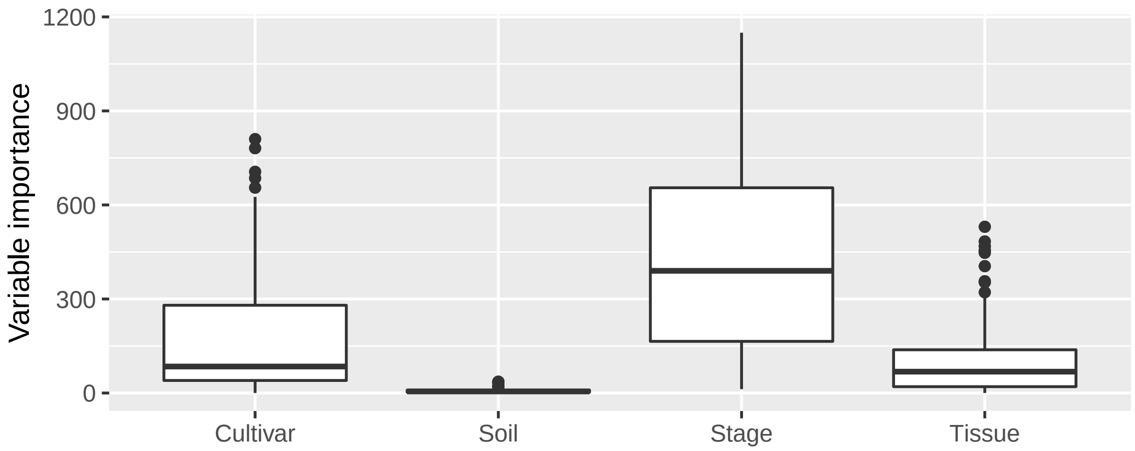
Variable importance
1200
900
600
300
0
Cultivar
Soil
Stage
Tissue

Cluster no. 40

```
## Number of genes in the cluster: 42
## Homogeneity Index:    0.88
## Variable importance for Stage:     Rank =  40  - Median =  377.2
## Variable importance for Cultivar:  Rank =  69  - Median =  16.17
## Variable importance for Tissue:    Rank =  39  - Median =    217
## Variable importance for Soil:      Rank =  30  - Median =   6.66
##
## Gene ID               Gene Annotation
## VIT_05s0102g00360  -  On-specific serine/threonine protein kinase
## VIT_02s0025g02800  -  No hit
## VIT_08s0032g00460  -  No hit
## VIT_00s0301g00100  -  BFN1 (bifunctional nuclease I)
## VIT_14s0171g00100  -  Blue (type 1) copper domain
## VIT_03s0063g01920  -  Pentatricopeptide (PPR) repeat
## VIT_18s0072g00950  -  Permease nonimprinted in Prader-Willi/Angelman
## VIT_06s0004g02510  -  Unknown protein
## VIT_02s0012g00880  -  AT-hook DNA-binding protein
## VIT_01s0010g02680  -  DNA-binding storekeeper protein
## VIT_14s0060g02000  -  ATPP2-B14
## VIT_07s0005g00780  -  No hit
## VIT_04s0044g01970  -  Subtilisin protease precursor
## VIT_08s0007g01940  -  UDP-sulfoquinovose:DAG sulfoquinovosyltransferase
## VIT_03s0063g01760  -  No hit
## VIT_16s0050g00480  -  CYP715A1
## VIT_13s0158g00430  -  R protein MLA10
## VIT_15s0048g00840  -  Lateral organ boundaries domain protein 19 (LBD19)
## VIT_01s0127g00050  -  SOUL heme-binding
## VIT_00s0762g00030  -  S-locus lectin protein kinase
## VIT_14s0060g02020  -  ATPP2-B15 (Phloem protein 2-B15)
## VIT_12s0028g02960  -  No hit
## VIT_17s0000g03850  -  Aluminum-activated malate transporter 9
## VIT_03s0063g00170  -  basic helix-loop-helix (bHLH) family
## VIT_18s0001g09480  -  PUMILIO 8 (APUM8)
## VIT_17s0000g09190  -  Octicosapeptide/Phox/Bem1p (PB1) domain-containing protein
## VIT_01s0127g00670  -  Proline extensin-like receptor kinase 1 (PERK1)
## VIT_01s0026g00740  -  Nucleobase-ascorbate transporter 6 (NAT6)
## VIT_11s0016g03050  -  MATE efflux family protein
## VIT_08s0040g01200  -  Short-chain type alcohol dehydrogenase
## VIT_18s0001g01060  -  Zinc finger (C3HC4-type ring finger)
## VIT_10s0071g00320  -  GRAM domain-containing protein / ABA-responsive
## VIT_13s0067g02300  -  Hypoxia-responsive
## VIT_07s0031g02860  -  Elongation factor EF-2
## VIT_07s0205g00010  -  Unknown
## VIT_03s0038g02140  -  Auxin transporter protein 2
## VIT_00s0347g00080  -  Curculin (mannose-binding) lectin
## VIT_16s0013g00480  -  Unknown protein
## VIT_01s0011g03540  -  Lateral organ boundaries protein 41
## VIT_07s0129g00930  -  Unknown protein
## VIT_05s0020g02920  -  Unknown protein
## VIT_10s0071g01060  -  Pyruvate kinase
```

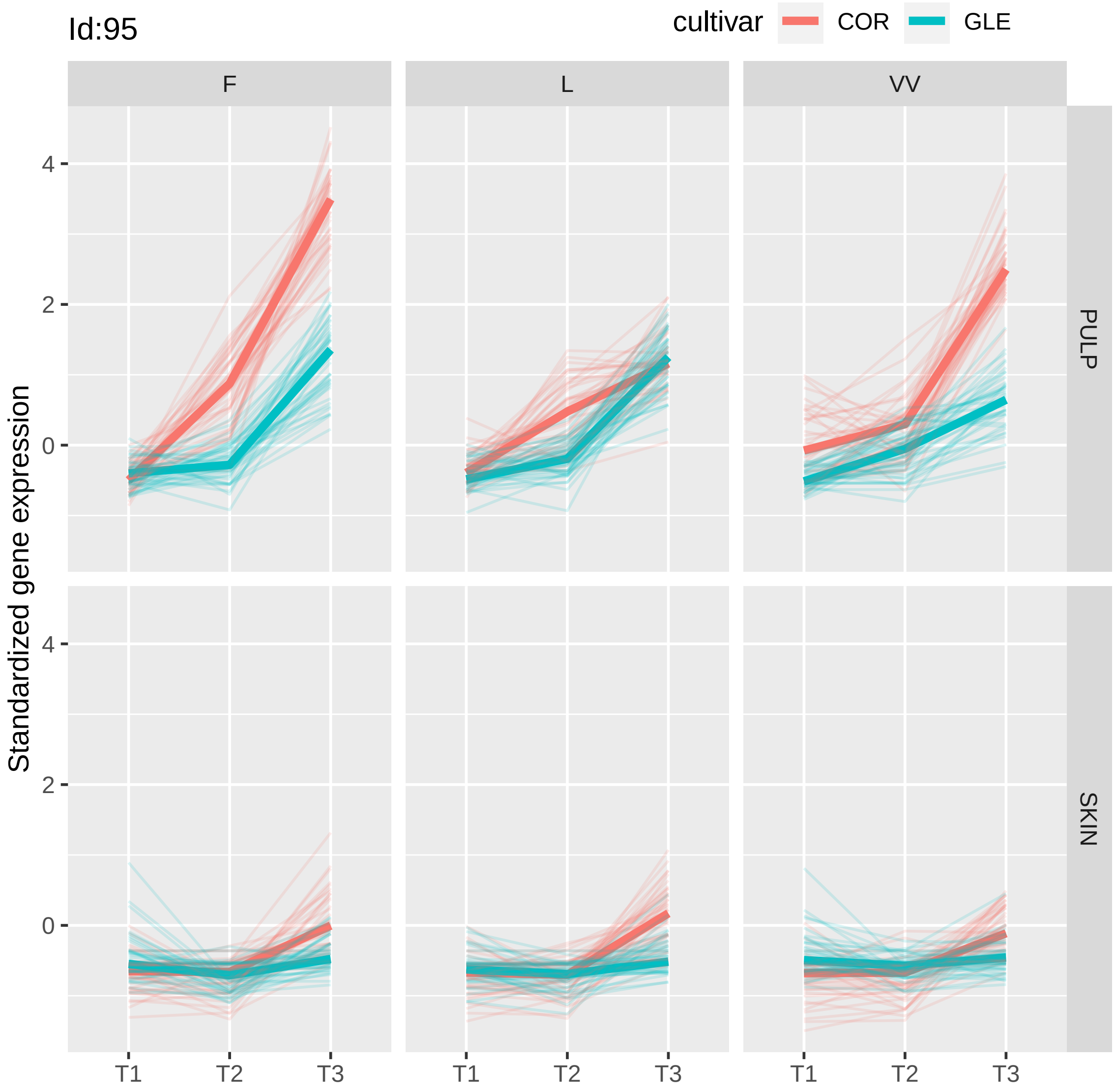
Id:95
cultivar
COR
GLE
F
L
VV
PULP
SKIN
Standardized gene expression
4
2
0
T1
T2
T3

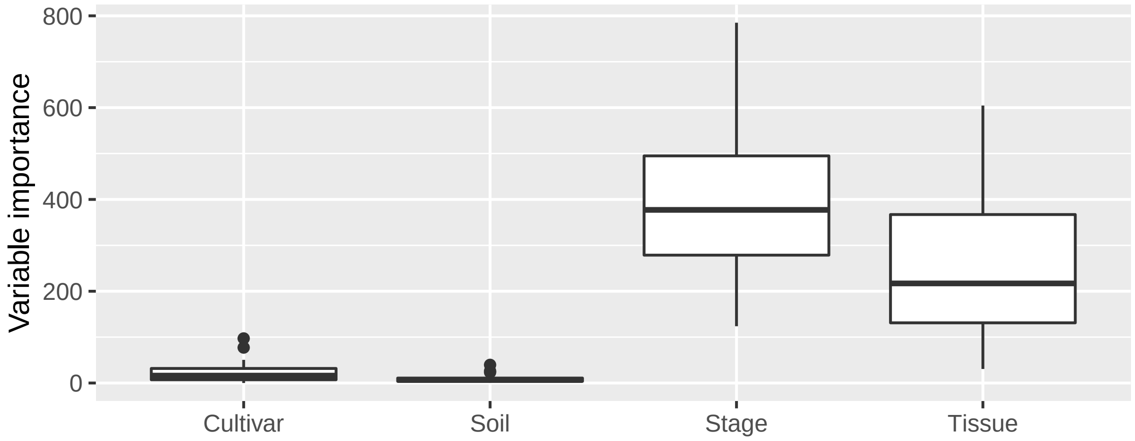
Variable importance
800
600
400
200
0
Cultivar
Soil
Stage
Tissue

Cluster no. 41

```
## Number of genes in the cluster: 112
## Homogeneity Index:    0.87
## Variable importance for Stage:     Rank =  41  - Median =  376.1
## Variable importance for Cultivar:  Rank = 101  - Median =   4.46
## Variable importance for Tissue:    Rank =  33  - Median =    277
## Variable importance for Soil:      Rank =  81  - Median =   3.78
##
## Gene ID               Gene Annotation
## VIT_12s0028g03270  -  Ethylene-responsive transcription factor 9
## VIT_00s0181g00180  -  LHCB3 (light-harvesting chlorophyll binding protein 3)
## VIT_13s0064g01480  -  Lipoxygenase LOX1
## VIT_18s0001g09230  -  Salt tolerance zinc finger
## VIT_08s0007g07390  -  Unknown protein
## VIT_16s0013g00900  -  ERF/AP2 Gene Family (VvERF079)
## VIT_17s0000g08720  -  RKF2 (receptor-like serine/threonine kinase 2)
## VIT_12s0059g00150  -  Ankyrin repeat
## VIT_14s0006g02290  -  Dehydration Responsive Element-Binding Transcription Factor (VvDREB20)
## VIT_06s0004g07680  -  Nodulin
## VIT_18s0001g11860  -  Polygalacturonate 4-alpha-galacturonosyltransferase
## VIT_09s0002g01410  -  TT2 (transparent testa 2)
## VIT_18s0001g07320  -  2-oxoglutarate/malate carrier protein, Mitochondrial
## VIT_12s0035g00900  -  TIFY gene family (VvJAZI 0)
## VIT_18s0001g08410  -  Homeobox-leucine zipper protein 22
## VIT_00s0386g00050  -  Xyloglucan endotransglucosylase/hydrolase precursor
## VIT_00s0194g00300  -  Proline-rich family protein
## VIT_05s0020g01100  -  Myb domain protein 61
## VIT_06s0009g03670  -  F-box family protein
## VIT_02s0033g01020  -  Anthraniloyal-CoA: methanol anthraniloyal transferase
## VIT_14s0068g00140  -  No hit
## VIT_03s0088g00290  -  Phytosulfokines PSK2
## VIT_07s0129g00920  -  Receptor-like protein kinase
## VIT_09s0002g08510  -  Expansin  (VvEXPB1)
## VIT_06s0061g01010  -  Prolylcarboxypeptidase
## VIT_11s0052g01140  -  Membrane protein
## VIT_11s0052g00530  -  Zinc-binding protein
## VIT_11s0016g03350  -  Dehydration Responsive Element-Binding Transcription Factor (VvDREB16)
## VIT_04s0044g00750  -  S-receptor kinase
## VIT_13s0067g02910  -  Non-specific lipid-transfer protein
## VIT_12s0028g04000  -  Ankyrin repeat
## VIT_08s0032g00790  -  ABC Transporter (VvWBC18 - VvABCG18)
## VIT_16s0022g02080  -  Exostosin family protein
## VIT_00s0225g00070  -  MATE efflux family protein
## VIT_13s0067g01590  -  Lectin protein kinase
## VIT_12s0059g01580  -  Unknown
## VIT_11s0016g03580  -  Catalytic
## VIT_19s0090g00660  -  Lipase GDSL
## VIT_12s0059g00140  -  Ankyrin repeat protein
## VIT_06s0004g00980  -  Dirigent protein pDIR9
## VIT_15s0048g02650  -  Unknown protein
## VIT_14s0036g00460  -  Myb domain protein 102
## VIT_07s0031g01680  -  CYP86A1
## VIT_01s0026g00880  -  Transducin protein
## VIT_13s0019g03040  -  Indole-3-acetate beta-glucosyltransferase
## VIT_05s0029g00480  -  Eceriferum 2 (CER2)
```

```
## VIT_11s0016g04840  -  Avr9/Cf-9 rapidly elicited protein 11
## VIT_14s0006g02550  -  Non-specific lipid-transfer protein 2 (LTP 2)
## VIT_00s0480g00090  -  Catechol oxidase (EC 1.10.3.1) precursor - grape
## VIT_12s0028g02400  -  Leaf senescence protein
## VIT_11s0052g00750  -  Serine carboxypeptidase 1 precursor
## VIT_08s0105g00190  -  U-box domain-containing protein
## VIT_18s0001g05670  -  myb domain protein 93
## VIT_04s0023g03710  -  myb domain protein 4 (VvMyb4b)
## VIT_12s0057g01030  -  Glucose-6-phosphate 1-dehydrogenase 2, chloroplast precursor
## VIT_14s0006g02570  -  Protease inhibitor/seed storage/lipid transfer protein (LTP)
## VIT_02s0025g01240  -  UDP-glucuronosyl/UDP-glucosyltransferase
## VIT_05s0102g00310  -  GUN4
## VIT_03s0063g01260  -  Nodulin 1A, Senescence-associated
## VIT_09s0054g00530  -  Zinc finger (B-box type)
## VIT_00s0194g00320  -  No hit
## VIT_00s0286g00050  -  S-locus protein kinase
## VIT_08s0007g05360  -  Strictosidine synthase
## VIT_04s0069g00610  -  Glutamate receptor protein
## VIT_15s0048g00530  -  Auxin-responsive SAUR11
## VIT_07s0031g00920  -  Inositol-3-phosphate synthase
## VIT_18s0122g01240  -  Serine/threonine Protein kinase BNK1
## VIT_00s0620g00010  -  Triacylglycerol lipase
## VIT_18s0001g06640  -  Zinc finger (C3HC4-type ring finger)
## VIT_17s0000g02050  -  Nudix hydrolase 17
## VIT_15s0046g02150  -  WRKY Transcription Factor (VvWRKY48)
## VIT_14s0006g02530  -  Non-specific lipid-transfer protein 2 (LTP 2)
## VIT_18s0001g07640  -  Eceriferum 2 (CER2)
## VIT_16s0050g00830  -  Beta-ketoacyl-CoA synthase
## VIT_18s0001g15220  -  Unknown protein
## VIT_04s0023g01020  -  BEL1 homeobox 2 protein (BLH2) (SAWTOOTH 1)
## VIT_06s0004g01550  -  Phosphoribosylanthranilate transferase
## VIT_06s0004g00970  -  Dirigent protein
## VIT_17s0000g08380  -  Unknown protein
## VIT_14s0066g02270  -  Endonuclease/exonuclease/phosphatase family protein
## VIT_18s0001g01320  -  Wall-associated receptor kinase 5
## VIT_07s0130g00040  -  myb domain protein 4
## VIT_14s0068g00160  -  No hit
## VIT_00s1314g00010  -  basic helix-loop-helix (bHLH) family
## VIT_08s0007g01420  -  Glutathione S-transferase 8 GSTU8
## VIT_14s0060g01090  -  LNG1 (LONGIFOLIA1)
## VIT_12s0035g00180  -  Leucine-rich repeat protein kinase
## VIT_05s0029g01580  -  Ankyrin repeat protein
## VIT_04s0008g01290  -  CGA1 (cytokinin-responsive GATA factor 1)
## VIT_18s0001g01760  -  putative MADS-box Pistillata (VviPI)
## VIT_11s0052g00800  -  Protein kinase family
## VIT_06s0004g07220  -  Indole-3-acetate beta-glucosyltransferase
## VIT_14s0066g00300  -  Unknown
## VIT_00s0265g00100  -  Lectin-receptor like protein kinase 3
## VIT_19s0015g00270  -  No hit
## VIT_07s0151g00010  -  No hit
## VIT_02s0012g02030  -  Homeobox-7
## VIT_01s0010g00710  -  Armadillo/beta-catenin repeat protein / U-box domain-containing protei
## VIT_11s0016g01310  -  TT2 (transparent testa 2)
## VIT_04s0008g05520  -  Unknown protein
```

```
## VIT_08s0040g02250  -  No hit
## VIT_02s0087g00630  -  Alcohol oxidase
## VIT_08s0040g03400  -  Short-chain dehydrogenase/reductase
## VIT_05s0029g01410  -  No hit
## VIT_09s0002g03940  -  ERF/AP2 Gene Family (VvERF034),Dehydration Responsive Element-Binding
## VIT_01s0150g00240  -  No hit
## VIT_11s0016g00880  -  Strictosidine synthase; Soluble quinoprotein glucose dehydrogenase
## VIT_00s0313g00040  -  PAS2 (PASTICCINO 2)
## VIT_01s0127g00340  -  Unknown
## VIT_18s0001g13520  -  Zinc finger (B-box type)
## VIT_01s0150g00330  -  U-box domain-containing protein
## VIT_04s0023g02810  -  Aldehyde Dehydrogenase (VvALDH3F1)
```

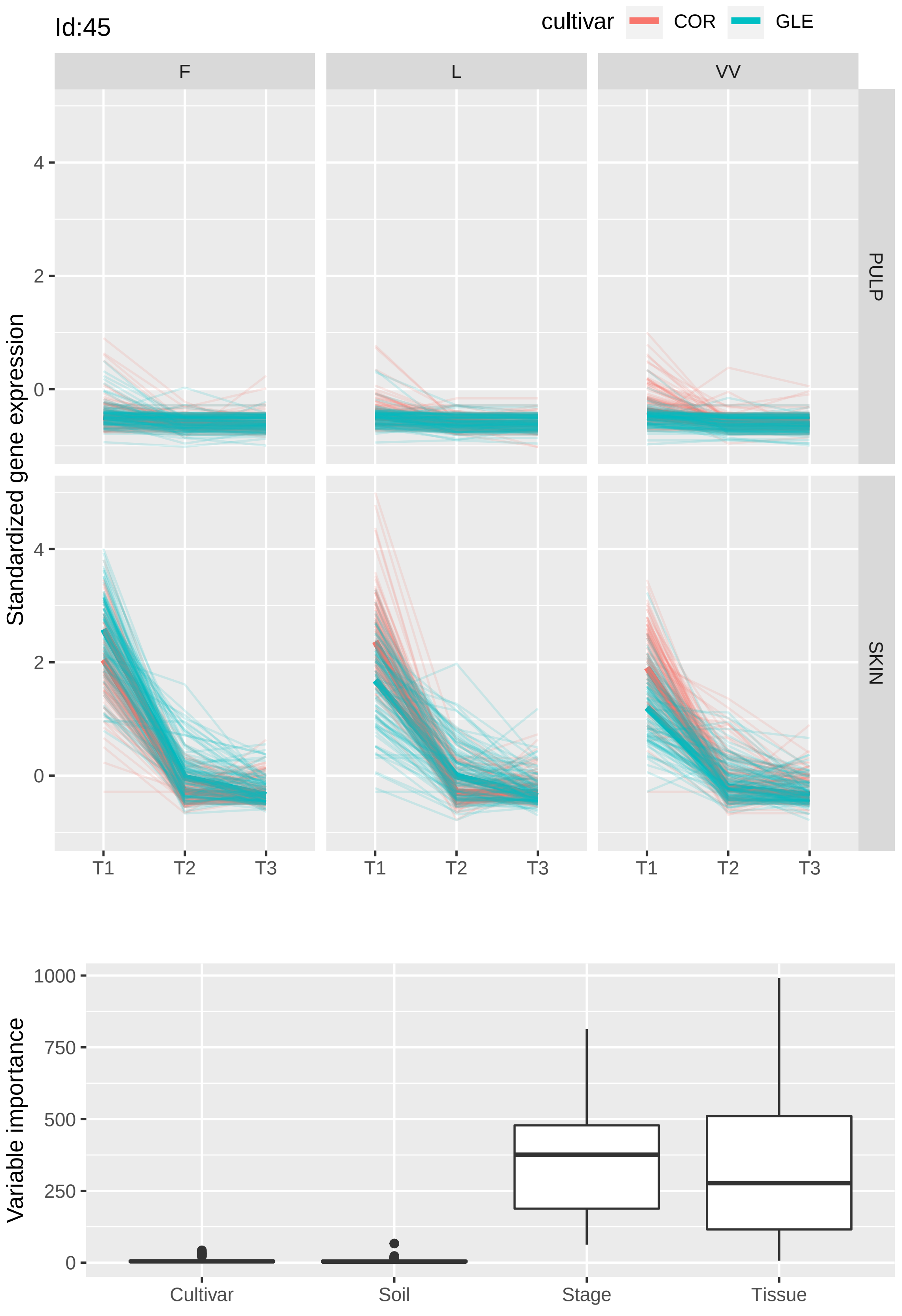

Id:45
cultivar
COR
GLE
F
L
VV
PULP
SKIN
Standardized gene expression
4
2
0
T1
T2
T3
Variable importance
1000
750
500
250
0
Cultivar
Soil
Stage
Tissue

Cluster no. 42

```
## Number of genes in the cluster: 74
## Homogeneity Index:    0.88
## Variable importance for Stage:     Rank =  42  - Median =  363.3
## Variable importance for Cultivar:  Rank =  94  - Median =   7.16
## Variable importance for Tissue:    Rank =  43  - Median =  170.2
## Variable importance for Soil:      Rank =  10  - Median =  10.58
##
## Gene ID               Gene Annotation
## VIT_16s0039g01870  -  Protein kinase
## VIT_00s0193g00030  -  Pentatricopeptide (PPR) repeat-containing protein
## VIT_10s0042g00870  -  Stilbene synthase (VvSTS2)
## VIT_16s0100g00880  -  Stilbene synthase (VvSTS19)
## VIT_11s0065g00350  -  Trans-cinnamate 4-monooxygenase
## VIT_16s0100g00900  -  Stilbene synthase (VvSTS20)
## VIT_16s0100g00810  -  Stilbene synthase (VvSTS13)
## VIT_16s0148g00290  -  Ser/Thr receptor-like kinase1
## VIT_12s0028g02010  -  PMR5 (powdery mildew resistant 5)
## VIT_11s0016g02070  -  basic helix-loop-helix (bHLH) family
## VIT_16s0039g01110  -  Phenylalanin ammonia-lyase [Vitis vinifera]
## VIT_16s0100g00990  -  Stilbene synthase (VvSTS27)
## VIT_18s0001g01960  -  RKF3 (receptor-like kinase IN in flowers 3)
## VIT_12s0059g01720  -  No hit
## VIT_16s0100g00780  -  Stilbene synthase (VvSTS10)
## VIT_07s0129g01010  -  Unknown
## VIT_16s0100g01070  -  Stilbene synthase (VvSTS35)
## VIT_10s0042g00920  -  Stilbene synthase (VvSTS5)
## VIT_16s0100g01000  -  Stilbene synthase (VvSTS28)
## VIT_16s0100g00920  -  Stilbene synthase (VvSTS22)
## VIT_14s0068g01920  -  Peroxidase
## VIT_16s0100g01010  -  Stilbene synthase (VvSTS29)
## VIT_16s0100g00840  -  Stilbene synthase (VvSTS16)
## VIT_18s0122g01470  -  CYP87A2
## VIT_10s0003g04080  -  Unknown
## VIT_16s0100g01160  -  Stilbene synthase (VvSTS45)
## VIT_16s0039g01300  -  Phenylalanine ammonia-lyase [Vitis vinifera]
## VIT_09s0002g05530  -  ABC Transporter (VvPDR13 - VvABCG43)
## VIT_16s0100g00910  -  Stilbene synthase (VvSTS21)
## VIT_16s0100g01140  -  Stilbene synthase (VvSTS42)
## VIT_16s0039g01100  -  Phenylalanin ammonia-lyase [Vitis vinifera]
## VIT_16s0100g01170  -  Stilbene synthase (VvSTS46)
## VIT_16s0100g01130  -  Stilbene synthase (VvSTS41)
## VIT_16s0100g00830  -  Stilbene synthase (VvSTS15)
## VIT_16s0100g00850  -  Stilbene synthase (VvSTS17)
## VIT_16s0100g01020  -  Stilbene synthase (VvSTS30)
## VIT_16s0100g00770  -  Stilbene synthase (VvSTS9)
## VIT_16s0100g00860  -  Stilbene synthase (VvSTS18)
## VIT_16s0100g01030  -  Stilbene synthase (VvSTS31)
## VIT_16s0100g01120  -  Stilbene synthase (VvSTS39)
## VIT_16s0100g01200  -  Stilbene synthase (VvSTS48)
## VIT_16s0100g00950  -  Stilbene synthase (VvSTS25)
## VIT_11s0016g03190  -  Unknown
## VIT_16s0100g00800  -  Stilbene synthase (VvSTS12)
## VIT_16s0039g01360  -  Phenylalanin ammonia-lyase [Vitis vinifera]
## VIT_16s0100g00940  -  Stilbene synthase (VvSTS24)
```

```
## VIT_05s0077g01560  -  s12 (VvPR10.3)_Pathogenesis protein 10 [Vitis vinifera]
## VIT_16s0100g01110  -  Stilbene synthase (VvSTS37)
## VIT_16s0100g01150  -  Stilbene synthase (VvSTS43)
## VIT_09s0002g02970  -  Unknown
## VIT_10s0042g00930  -  Stilbene synthase (VvSTS6)
## VIT_18s0001g04280  -  (-)-germacrene D synthase (VvTPS07), Germacrene D Syn
## VIT_05s0077g01690  -  s1_Pathogenesis protein 10 [Vitis vinifera]
## VIT_16s0039g01240  -  Phenylalanin ammonia-lyase [Vitis vinifera]
## VIT_16s0100g01100  -  Stilbene synthase (VvSTS36)
## VIT_16s0100g00750  -  Stilbene synthase (VvSTS7)
## VIT_05s0077g01550  -  s14_Pathogenesis protein 10 [Vitis vinifera]
## VIT_06s0004g01020  -  Dirigent protein
## VIT_16s0039g01130  -  Phenylalanin ammonia-lyase [Vitis vinifera]
## VIT_18s0001g00740  -  Ornithine decarboxylase
## VIT_16s0100g01040  -  Stilbene synthase (VvSTS32)
## VIT_12s0035g01000  -  Serine protease inhibitor, serine-type
## VIT_05s0077g01530  -  s16 (VvPR10.1)_Pathogenesis protein 10 [Vitis vinifera]
## VIT_11s0016g03420  -  Ring-H2 finger protein ATL4J
## VIT_07s0005g02560  -  Chitinase Class I
## VIT_05s0077g01540  -  s15_Pathogenesis protein 10 [Vitis vinifera]
## VIT_06s0004g01030  -  Dirigent protein
## VIT_10s0003g00480  -  Isoflavone methyltransferase/ Orcinol O-methyltransferase 1 oomt1
## VIT_16s0100g01190  -  Stilbene synthase (VvSTS47)
## VIT_01s0146g00480  -  TIFY gene family (VvJAZ2)
## VIT_00s2849g00010  -  Phenylalanine ammonia-lyase
## VIT_13s0019g03520  -  No hit
## VIT_14s0006g00140  -  No hit
## VIT_11s0016g05540  -  Dicyanin
```

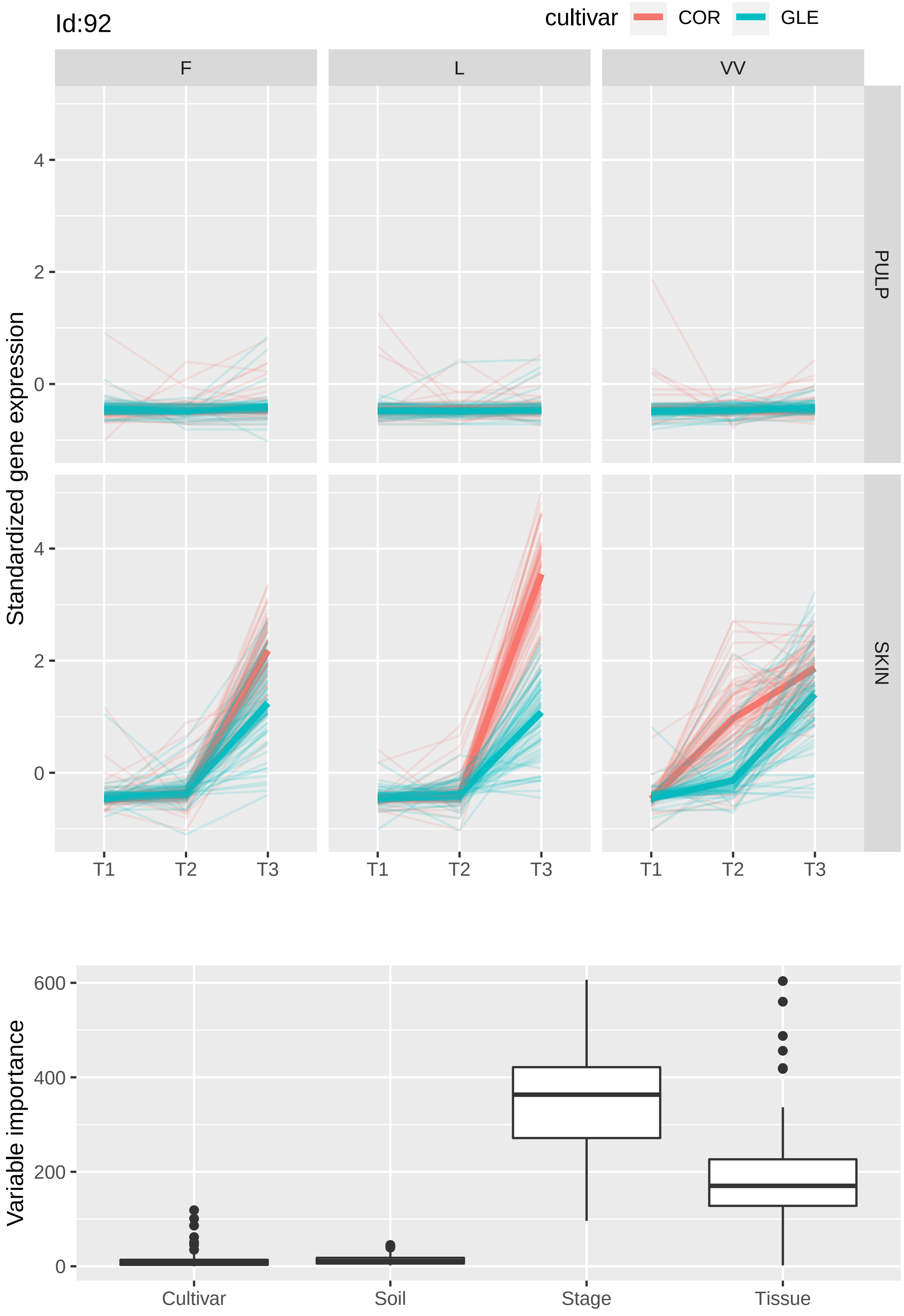

Id:92
cultivar
COR
GLE
F
L
VV
PULP
SKIN
Standardized gene expression
4
2
0
T1
T2
T3
Variable importance
600
400
200
0
Cultivar
Soil
Stage
Tissue

Cluster no. 43

```
## Number of genes in the cluster: 104
## Homogeneity Index:    0.89
## Variable importance for Stage:     Rank =  43  - Median =  361.5
## Variable importance for Cultivar:  Rank =  87  - Median =   9.79
## Variable importance for Tissue:    Rank =  50  - Median =  127.1
## Variable importance for Soil:      Rank =  64  - Median =   5.06
##
## Gene ID               Gene Annotation
## VIT_08s0040g01510  -  No hit
## VIT_12s0059g01900  -  Unknown protein
## VIT_05s0020g03770  -  Inositol polyphosphate 5'-phosphatase
## VIT_18s0001g12890  -  Jasmonate O-methyltransferase
## VIT_18s0001g14980  -  3-methyl-2-oxobutanoate dehydrogenase
## VIT_18s0001g01680  -  Protein kinase
## VIT_18s0041g00840  -  UDP-glucose: anthocyanidin 5,3-O-glucosyltransferase
## VIT_16s0022g01970  -  Anthocyanidin 3-O-glucosyltransferase
## VIT_08s0007g00700  -  Aspartic Protease (VvAP21)
## VIT_14s0083g00850  -  Lipase GDSL 7
## VIT_01s0011g03400  -  Proton-dependent oligopeptide transport (POT) family protein
## VIT_12s0028g03990  -  Ankyrin repeat
## VIT_02s0025g01300  -  Unknown protein
## VIT_14s0060g00680  -  Lipase GDSL
## VIT_12s0059g02320  -  Unknown protein
## VIT_18s0001g05850  -  Dehydration Responsive Element-Binding Transcription Factor (VvDREB31)
## VIT_06s0004g03020  -  Beta-galactosidase
## VIT_12s0028g03930  -  Retrotransposon protein, Unclassified
## VIT_19s0014g04640  -  S-locus protein kinase
## VIT_02s0025g00750  -  Pinoresinol forming dirigent protein
## VIT_02s0025g00180  -  Bisphosphoglycerate mutase
## VIT_14s0006g01280  -  myb domain protein 113 (VvMYBA7)
## VIT_18s0001g09790  -  S-domain receptor kinase
## VIT_18s0157g00170  -  Alliin lyase precursor
## VIT_08s0007g07550  -  GATA transcription factor 11
## VIT_14s0068g00150  -  No hit
## VIT_00s0253g00150  -  Methyl jasmonate esterase
## VIT_10s0003g00340  -  ACR4 (Arabidopsis CRINKLY4)
## VIT_11s0016g01450  -  Unknown protein
## VIT_18s0001g06950  -  Purine permease 1 (PUP1)
## VIT_00s0194g00330  -  Proline-rich family protein
## VIT_02s0025g00760  -  Pinoresinol forming dirigent protein
## VIT_01s0011g02840  -  Cytochrome B561
## VIT_13s0019g01200  -  Unknown
## VIT_05s0102g00190  -  No hit
## VIT_13s0067g01180  -  Leaf senescence related
## VIT_02s0033g01050  -  Anthraniloyal-CoA: methanol anthraniloyal transferase
## VIT_03s0180g00150  -  Receptor protein kinase
## VIT_00s0361g00090  -  Phototropic-responsive NPH3 protein
## VIT_06s0061g00290  -  No hit
## VIT_11s0016g00300  -  Pectinesterase family
## VIT_00s0375g00030  -  Alpha-galactosidase
## VIT_18s0001g14760  -  Lipase 3 (EXL3) family II extracellular
## VIT_07s0005g06300  -  Phototropic-responsive NPH3
## VIT_05s0062g00520  -  UDP-glucose:flavonoid 7-O-glucosyltransferase
## VIT_01s0011g02000  -  Serine carboxypeptidase S10
```

```
## VIT_00s0125g00290  -  Lateral organ boundaries domain protein 4 (LBD4)
## VIT_00s0480g00070  -  Polyphenol oxidase II, chloroplast precursor
## VIT_05s0029g01490  -  Ankyrin repeat
## VIT_07s0005g01790  -  Acyl-CoA synthetase long-chain member 2
## VIT_00s0391g00030  -  Unknown
## VIT_01s0011g03210  -  Aspartic Protease (VvAP1)
## VIT_01s0137g00170  -  B-keto acyl reductase
## VIT_07s0129g00660  -  Indole-3-acetic acid-amido synthetase GH3.2
## VIT_00s0181g00200  -  LHCB3 (light-harvesting chlorophyll binding protein 3)
## VIT_00s0480g00060  -  Polyphenol oxidase [Vitis vinifera]
## VIT_03s0091g00450  -  Progesterone 5-beta-reductase
## VIT_17s0000g09080  -  myb domain protein 4
## VIT_18s0075g00270  -  Unknown
## VIT_12s0028g03960  -  Ankyrin repeat protein family
## VIT_07s0151g01000  -  Photosystem I reaction center subunit II (PSAD)
## VIT_18s0001g06910  -  Purine permease 1 PUP1
## VIT_11s0016g05340  -  ERF/AP2 Gene Family (VvERF043)
## VIT_11s0037g00320  -  Serine carboxypeptidase S10
## VIT_18s0001g03860  -  Unknown protein
## VIT_01s0011g01320  -  Cholinephosphate cytidylyltransferase
## VIT_07s0031g02990  -  Ammonium transporter 2
## VIT_12s0028g01850  -  Receptor kinase TRKe
## VIT_00s0286g00110  -  S-locus protein kinase
## VIT_18s0041g00230  -  TIR-NBS-LRR disease resistance
## VIT_15s0021g02680  -  Cyclin-U2-1 (CycU2;1) Cyclin-P3.1
## VIT_06s0061g00970  -  Prolylcarboxypeptidase
## VIT_13s0019g03700  -  Lateral organ boundaries protein 1
## VIT_11s0016g00590  -  Invertase/pectin methylesterase inhibitor
## VIT_14s0030g00220  -  Sugar transporter ERD6-like 5
## VIT_01s0137g00410  -  CYP86A2
## VIT_15s0046g02190  -  WRKY Transcription Factor (VvWRKY49)
## VIT_02s0025g01860  -  Cellulose synthase CSLG3
## VIT_10s0116g00560  -  Polyphenol oxidase II, chloroplast precursor
## VIT_04s0008g03950  -  RD22
## VIT_11s0016g00290  -  Pectinesterase PME3
## VIT_01s0011g03860  -  RKL1 (Receptor-like kinase 1)
## VIT_13s0064g00390  -  Polyamine oxidase
## VIT_12s0057g00930  -  Hydroxycinnamoyl-CoA:shikimate hydroxycinnamoyltransferase
## VIT_09s0002g02870  -  Leucine-rich repeat protein kinase
## VIT_15s0046g00660  -  Wax synthase
## VIT_14s0066g01220  -  ATMYB66/WER/WER1 (WEREWOLF 1)
## VIT_10s0597g00050  -  Heat shock transcription factor B2A
## VIT_07s0129g01070  -  Leucine-rich repeat protein kinase
## VIT_15s0046g00520  -  Wax synthase
## VIT_00s0397g00010  -  HcrVf1 protein
## VIT_06s0004g00990  -  Dirigent protein
## VIT_13s0067g00400  -  Anthranilate synthase alpha 1
## VIT_13s0064g00340  -  Cinnamyl alcohol dehydrogenase
## VIT_00s0421g00040  -  Disease resistance family protein
## VIT_14s0128g00210  -  Xyloglucanase inhibitor
## VIT_09s0070g00240  -  Cinnamoyl-CoA reductase
## ENSRNA049996142  -
## VIT_12s0134g00670  -  Hydroxycinnamoyl-CoA:anthocyanin 5-aromatic acyltransferase
## VIT_01s0026g00080  -  Metal-dependent phosphohydrolase HD domain-containing protein
```

```
## VIT_18s0001g15230  -  Unknown
## VIT_00s0265g00170  -  Lipoxygenase
## VIT_19s0015g01440  -  Unknown protein
## VIT_17s0000g06130  -  Glutathione S-transferase 9 GSTU9
```

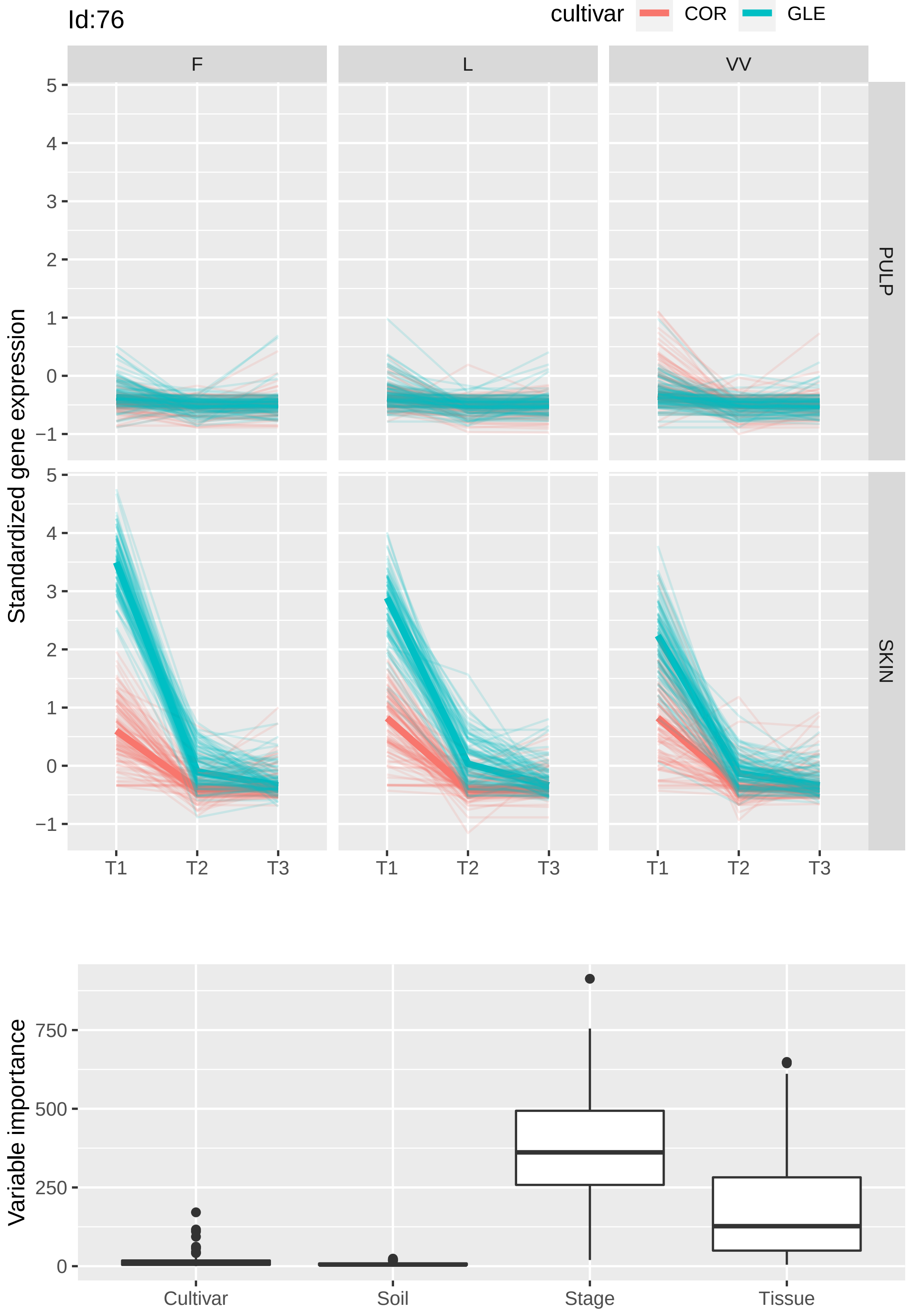
Id:76
cultivar
COR
GLE
F
L
VV
PULP
SKIN
Standardized gene expression
T1
T2
T3
Variable importance
Cultivar
Soil
Stage
Tissue

Cluster no. 44

```
## Number of genes in the cluster: 123
## Homogeneity Index:    0.82
## Variable importance for Stage:     Rank =  44  - Median =  340.6
## Variable importance for Cultivar:  Rank =  54  - Median =  37.72
## Variable importance for Tissue:    Rank =  24  - Median =    455
## Variable importance for Soil:      Rank =  23  - Median =   7.05
##
## Gene ID                Gene Annotation
## VIT_18s0001g10690  -  BKI1 (BRI1 kinase inhibitor 1)
## VIT_05s0077g01450  -  GATA transcription factor 16
## VIT_19s0027g00340  -  Unknown protein
## VIT_04s0008g02000  -  Unknown protein
## VIT_12s0035g00910  -  Bile acid sodium symporter
## VIT_01s0026g00260  -  Potassium transporter (KUP7)
## VIT_19s0014g00810  -  RKF1 (receptor-like kinase in flowers 1)
## VIT_02s0025g00200  -  BEL1 (BELL 1)
## VIT_17s0000g02570  -  Mitogen-activated Protein Kinase (VvMPK10)
## VIT_04s0069g00780  -  Cellulose synthase CSLC05
## VIT_06s0061g00010  -  Magnesium-chelatase subunit chlD, chloroplast precursor
## VIT_19s0177g00280  -  DNAJ heat shock N-terminal domain-containing
## VIT_08s0040g01400  -  Thylakoid lumenal 15.0 kDa protein 2
## VIT_04s0008g05340  -  Bundle-sheath defective protein 2
## VIT_17s0119g00110  -  GRAM domain-containing protein / ABA-responsive
## VIT_18s0001g09450  -  Unknown protein
## VIT_10s0116g01540  -  Aminomethyltransferase
## VIT_00s2326g00010  -  Ankyrin
## VIT_12s0057g00020  -  Protein thylakoid formation1
## VIT_08s0007g08760  -  Thioredoxin reductase
## VIT_06s0004g08380  -  Unknown protein
## VIT_04s0023g00740  -  Thioredoxin F-type 2 (Trx-F2)
## VIT_08s0056g01160  -  BDG1 (BODYGUARD1) hydrolase
## VIT_00s0965g00010  -  Magnesium-chelatase subunit chlD, chloroplast precursor
## VIT_06s0004g06540  -  Undecaprenyl pyrophosphate synthetase
## VIT_03s0063g02020  -  Tic Complex Tic62 Subunit
## VIT_18s0001g11750  -  Ribosomal protein S9
## VIT_13s0067g01490  -  NBS-LRR type disease resistance protein Rps1-k-1
## VIT_19s0015g00260  -  Import inner membrane translocase subunit TIM50
## VIT_06s0004g03690  -  Glutathione S-transferase GSTO1
## VIT_01s0127g00130  -  Cysteine-rich receptor-like protein kinase 3
## VIT_17s0000g07020  -  Cis-zeatin O-beta-D-glucosyltransferase
## VIT_01s0150g00130  -  Unknown protein
## VIT_14s0128g00470  -  Unknown protein
## VIT_06s0061g00170  -  R protein MLA10
## VIT_06s0004g01160  -  10-formyltetrahydrofolate synthetase
## VIT_14s0060g01430  -  Unknown protein
## VIT_11s0103g00550  -  Subtilisin-like serine protease 3
## VIT_17s0000g03820  -  Unknown protein
## VIT_00s0291g00060  -  Inorganic phosphate transporter 2-1, chloroplast precursor
## VIT_19s0085g00380  -  Phosphoglycerate kinase
## VIT_08s0056g00100  -  Protein kinase
## VIT_18s0089g01390  -  Unknown protein
## VIT_15s0021g00970  -  NAK-type protein kinase
## VIT_19s0090g01000  -  VTC2 (vitamin C defective)
## VIT_07s0005g01210  -  VvMybF1
```

```
## VIT_16s0098g01550  -  No hit
## VIT_14s0128g00050  -  Thylakoid lumenal 18.3 kDa protein
## VIT_15s0021g01300  -  Pentatricopeptide (PPR) repeat-containing protein
## VIT_03s0180g00110  -  Stress enhanced protein 1 (SEP1)
## VIT_14s0066g01410  -  Peptidylprolyl isomerase
## VIT_02s0012g00570  -  Pseudo-response regulator 2 (APRR2) (TOC2)
## VIT_10s0003g04470  -  ABC Transporter (VvMRP20 - VvABCC20)
## VIT_06s0061g00330  -  Unknown protein
## VIT_14s0060g02430  -  Photosystem II oxygen evolving complex protein PsbP
## VIT_05s0049g00310  -  E8 protein
## VIT_08s0007g00840  -  Ribulose bisphosphate carboxylase/oxygenase activase, chloroplast
## VIT_01s0026g01120  -  R protein L6
## VIT_18s0001g15360  -  Thylakoid lumenal 29.8 kDa protein
## VIT_14s0066g00850  -  Nitrate transporter2.5
## VIT_10s0071g00440  -  Serine/threonine-protein kinase receptor ARK3
## VIT_17s0000g05470  -  Nodulin
## VIT_13s0084g00880  -  NADPH:quinone oxidoreductase
## VIT_05s0020g02130  -  1-deoxy-D-xylulose-5-phosphate synthase, chloroplast precursor (VvDXS)
## VIT_18s0072g00980  -  Leucine-rich repeat protein kinase
## VIT_11s0016g01880  -  lycopene epsilon-cyclase (LECY) (VvLECY1)
## VIT_07s0005g01830  -  Unknown protein
## VIT_01s0137g00290  -  Oxysterol binding protein
## VIT_19s0135g00060  -  Glyoxalase II
## VIT_01s0026g00190  -  Armadillo/beta-catenin repeat
## VIT_08s0056g01690  -  LrgB-like family protein
## VIT_02s0025g02450  -  Unknown protein
## VIT_03s0180g00280  -  Indole-3-acetate beta-glucosyltransferase
## VIT_04s0069g01000  -  Drug/metabolite transporter DMT family transporter
## VIT_02s0012g02540  -  Chlororespiratory reduction 4 (CRR4)
## VIT_00s0207g00210  -  Photosystem II PsbO protein
## VIT_19s0014g04090  -  Serine/threonine-protein kinase receptor ARK3
## VIT_08s0007g04610  -  UGT73C2 (UDP-glucosyl transferase 73C2)
## VIT_15s0024g00660  -  Esterase
## VIT_08s0007g06480  -  Unknown protein
## VIT_14s0068g01950  -  Ribosomal protein L27, chloroplast (RPL27) 50S
## VIT_07s0151g00590  -  Inositol-1,4,5-trisphosphate 5-phosphatase CVP2, type I
## VIT_12s0059g02250  -  Ribosomal protein L6
## VIT_19s0014g03850  -  Cytochrome B6-F complex iron-sulfur subunit, PETC
## VIT_05s0020g03490  -  ferredoxin
## VIT_13s0101g00320  -  Pseudo-response regulator 2 (APRR2) (TOC2)
## VIT_16s0013g00260  -  Superoxide dismutase, Fe-Mn family
## VIT_19s0015g02410  -  Receptor protein kinase
## VIT_00s0684g00030  -  SEN1 (dark inducible 1)
## VIT_15s0046g01780  -  Ribosomal protein L5
## VIT_12s0059g01810  -  Photosystem II psbZ
## VIT_04s0044g01310  -  NADH-plastoquinone oxidoreductase subunit o
## VIT_04s0044g01430  -  Unknown protein
## VIT_19s0015g00730  -  Cellulose synthase CSLE1
## VIT_14s0068g00680  -  Glyceraldehyde-3-phosphate dehydrogenase A, chloroplast precursor
## VIT_09s0002g07500  -  Hydrolase, alpha/beta fold
## VIT_02s0154g00410  -  Unknown protein
## VIT_08s0040g00390  -  Magnesium-protoporphyrin IX monomethyl ester [oxidative] cyclase
## VIT_03s0038g02510  -  Glyoxylate reductase
## VIT_10s0003g04940  -  Acyl-CoA synthetases (Acyl-activating enzyme 14)
## VIT_06s0004g06140  -  Ribosomal protein L4
```

```
## VIT_11s0118g00310  -  1,2-diacylglycerol 3-beta-galactosyltransferase
## VIT_18s0001g02740  -  Photosystem II 22 kDa protein PSBS
## VIT_07s0031g02780  -  Cyclase/dehydrase
## VIT_17s0000g00280  -  Magnesium-protoporphyrin O-methyltransferase
## VIT_04s0023g02130  -  1,4-beta-mannan endohydrolase
## VIT_12s0057g00750  -  CF9
## VIT_04s0008g00450  -  Haloacid dehalogenase hydrolase
## VIT_15s0046g01880  -  Brassinosteroid-responsive ring-H2 (BRH1)
## VIT_08s0007g01490  -  Unknown protein
## VIT_08s0040g00300  -  No hit
## VIT_02s0025g02500  -  Metal-nicotianamine transporter YSL1
## VIT_17s0000g10260  -  AarF domain containing kinase
## VIT_16s0050g01700  -  Receptor serine/threonine kinase PR5K
## VIT_12s0028g01790  -  FAD-linked oxidoreductase 1
## VIT_03s0063g00750  -  Carboxylesterase CXE
## VIT_01s0026g01300  -  Unknown protein
## VIT_13s0156g00540  -  Disease resistance protein (CC-NBS-LRR class)
## VIT_12s0035g00080  -  Cleavage and polyadenylation specificity factor subunit 3
## VIT_13s0064g00270  -  Cinnamyl alcohol dehydrogenase
## VIT_04s0023g01090  -  Unknown
## VIT_11s0206g00110  -  Serine carboxypeptidase S28
## VIT_17s0000g06210  -  GASA like
```

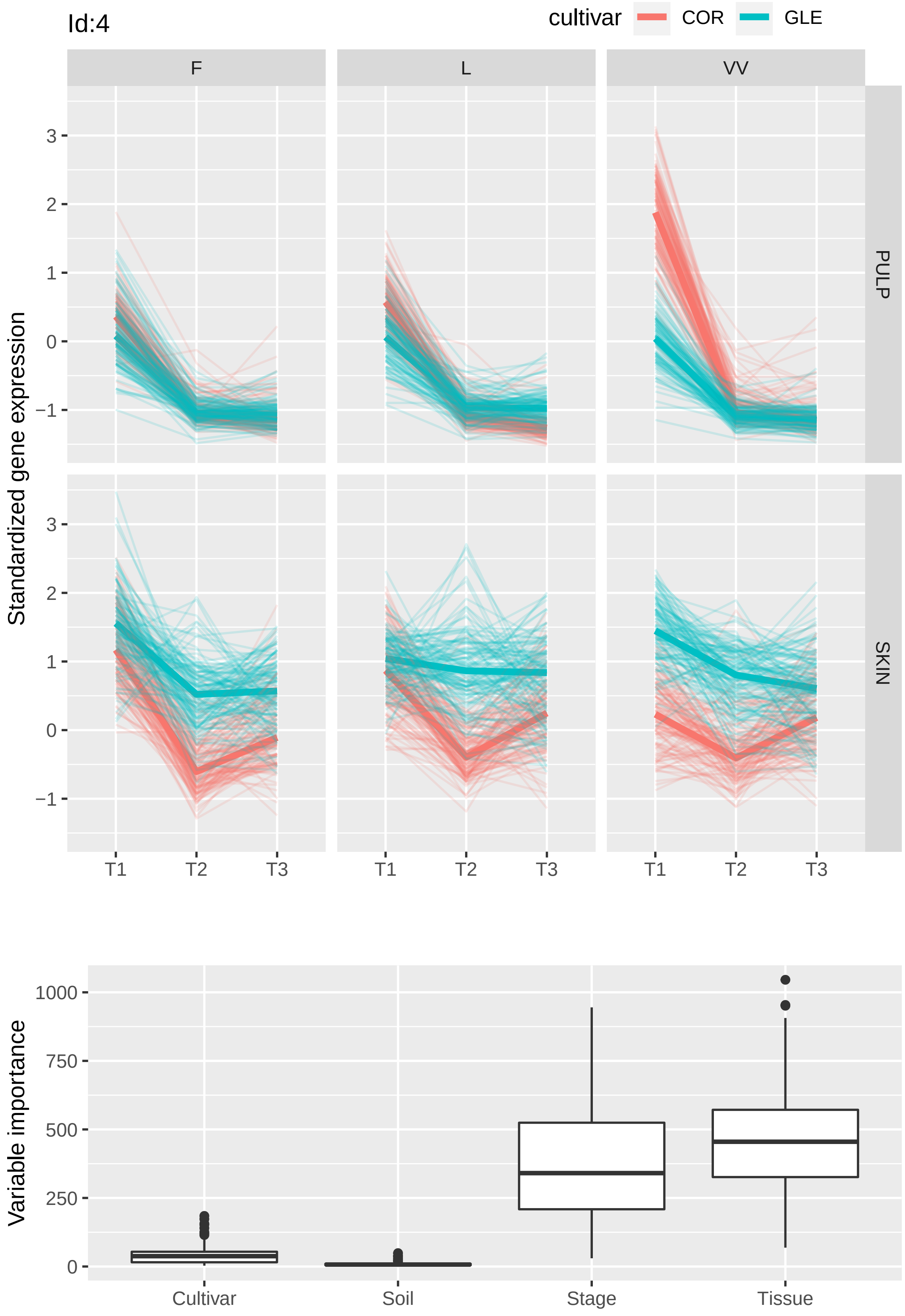
Id:4
cultivar
COR
GLE
F
L
VV
PULP
SKIN
Standardized gene expression
3
2
1
0
−1
T1
T2
T3
Variable importance
1000
750
500
250
0
Cultivar
Soil
Stage
Tissue

Cluster no. 45

```
## Number of genes in the cluster: 136
## Homogeneity Index:    0.89
## Variable importance for Stage:     Rank =  45  - Median =  333.4
## Variable importance for Cultivar:  Rank =  51  - Median =  44.09
## Variable importance for Tissue:    Rank =  73  - Median =  33.63
## Variable importance for Soil:      Rank =  27  - Median =   6.78
##
## Gene ID               Gene Annotation
## VIT_05s0062g00250  -  Xyloglucan endotransglucosylase/hydrolase 15
## VIT_06s0061g01380  -  Pop3 peptide
## VIT_12s0059g00520  -  RabGAP/TBC domain-containing protein
## VIT_18s0001g15730  -  Dof zinc finger protein DOF3.5
## VIT_05s0020g03880  -  TSO1 (chinese for 'ugly')
## VIT_13s0064g00590  -  Cyclin-related
## VIT_14s0083g00620  -  NIK1 (NSP- interacting  kinase 1)
## VIT_07s0129g00870  -  Protein kinase family
## VIT_08s0040g00820  -  CYP94A1
## VIT_02s0025g00360  -  1-aminocyclopropane-1-carboxylate synthase
## VIT_08s0058g00450  -  Substrate carrier, Mitochondrial
## VIT_18s0001g04920  -  No hit
## VIT_07s0104g01810  -  Glutathione S-transferase 13 GSTF13
## VIT_12s0028g02440  -  Unknown protein
## VIT_05s0049g02080  -  Calcium ion binding protein
## VIT_11s0016g05000  -  Dynein light chain LC8-type
## VIT_02s0025g02080  -  Nodulin MtN3 family protein
## VIT_17s0000g04090  -  Cell division cycle associated 7
## VIT_07s0104g01800  -  Glutathione S-transferase 13 GSTF13
## VIT_08s0007g08320  -  Pentatricopeptide (PPR) repeat-containing
## VIT_10s0003g01690  -  Unknown protein
## VIT_00s1455g00010  -  Expansin  (VvEXLB4)
## VIT_03s0038g02110  -  Co-chaperone-curved DNA binding protein A
## VIT_06s0004g02850  -  Aquaporin PIP2B
## VIT_12s0134g00280  -  S-locus lectin protein kinase
## VIT_18s0001g12650  -  Unknown protein
## VIT_05s0020g04130  -  Perakine reductase aldo/keto reductase
## VIT_19s0140g00140  -  GA 20-oxidase 2
## VIT_09s0002g06290  -  Ankyrin
## VIT_13s0019g01840  -  Cell division cycle 20-like protein 1
## VIT_03s0091g00500  -  Unknown protein
## VIT_13s0019g00330  -  Aquaporin TIP1;3
## VIT_05s0062g01280  -  Cyclo-DOPA 5-O-glucosyltransferase
## VIT_06s0004g01410  -  S-receptor kinase
## VIT_17s0000g00820  -  Nodulin MtN3 family
## VIT_05s0020g02720  -  Aspartic Protease (VvAP11)
## VIT_04s0023g00320  -  Auxin efflux carrier protein 8
## VIT_02s0012g00550  -  Inositol polyphosphate 5-phosphatase II
## VIT_12s0134g00290  -  Receptor ser/thr protein kinase
## VIT_18s0089g00040  -  NBS-LRR disease resistance protein
## VIT_14s0066g00600  -  Unknown protein
## VIT_05s0029g00400  -  Kinesin PAKRP1L
## VIT_00s0199g00120  -  Heat shock protein binding
## VIT_17s0000g03960  -  Fw2.2 ORFX
## VIT_04s0008g05380  -  Serine hydrolase [Vitis vinifera]
## VIT_14s0068g01570  -  Glutaredoxin-like
```

```
## VIT_04s0044g01860  -  Auxin efflux carrier
## VIT_00s2086g00010  -  1-aminocyclopropane-1-carboxylate oxidase 1
## VIT_13s0064g00600  -  Cyclin-related
## VIT_00s0333g00060  -  Unknown protein
## VIT_15s0024g01230  -  No hit
## VIT_03s0038g04120  -  Cellulose synthase CSLD4
## VIT_02s0236g00060  -  Unknown
## VIT_16s0098g00400  -  Receptor serine/threonine kinase PR5K-l
## VIT_03s0091g00420  -  No hit
## VIT_04s0023g02290  -  S-adenosyl-L-methionine:salicylic acid carboxyl methyltransferase
## VIT_16s0039g02750  -  NADH glutamate dehydrogenase
## VIT_07s0005g02060  -  Boron transporter-like protein 1
## VIT_05s0020g03970  -  Sulfate transporter 3.1 (AST12) (AtST1)
## VIT_00s0214g00130  -  F-box family protein
## VIT_14s0108g01260  -  Unknown protein
## VIT_13s0064g01800  -  R protein MLA10
## VIT_15s0046g00230  -  Lateral organ boundaries protein 1
## VIT_02s0025g02510  -  Metal-nicotianamine transporter YSL1
## VIT_02s0025g03140  -  Nodulin MtN21 family
## VIT_11s0037g00800  -  Serine carboxypeptidase II
## VIT_05s0062g00240  -  Xyloglucan endotransglucosylase/hydrolase 23
## VIT_15s0048g00910  -  Unknown protein
## VIT_05s0049g01830  -  CCT motif constans-like
## VIT_05s0020g01960  -  Na+/H+ antiporter isoform 2
## VIT_17s0000g09760  -  ABC Transporter (VvMDR2 - VvABCB2)
## VIT_12s0055g00940  -  Sulfate transporter 1.2
## VIT_16s0039g02720  -  Glutamate dehydrogenase 2 (GDH2)
## VIT_19s0027g00990  -  No hit
## VIT_12s0134g00300  -  S-locus lectin protein kinase family
## VIT_17s0000g04210  -  Oleosin OLE-5
## VIT_13s0019g02550  -  Subtilisin protease C1
## VIT_00s0144g00190  -  R protein MLA10
## VIT_10s0003g00980  -  Unknown protein
## VIT_03s0038g04720  -  Syntaxin 1B/2/3/4
## VIT_18s0041g02200  -  No hit
## VIT_04s0069g00750  -  AHK5 (Cytokinin independent 2)
## VIT_15s0048g00520  -  Receptor-like protein kinase
## VIT_11s0016g05810  -  Potassium channel SKOR
## VIT_11s0016g03220  -  RNA-directed RNA polymerase
## VIT_03s0091g00390  -  Snakin-1
## VIT_14s0030g00350  -  RKF3 (receptor-like kinase IN in flowers 3)
## VIT_08s0007g00760  -  Thaumatin
## VIT_19s0093g00220  -  Glutathione S-transferase 8 GSTU19
## VIT_07s0005g05520  -  Pectate lyase
## VIT_02s0025g01600  -  Harpin-induced 1
## VIT_16s0022g00080  -  HcrVf2 protein
## VIT_04s0023g02160  -  Unknown protein
## VIT_07s0005g01360  -  Zinc finger (C3HC4-type ring finger)
## VIT_19s0014g04410  -  S-locus protein kinase
## VIT_00s0153g00050  -  Glutathione S-transferase 8 GSTU8
## VIT_07s0151g00680  -  Tetratricopeptide repeat (TPR)-containing
## VIT_07s0031g00020  -  Phosphoric monoester hydrolase
## VIT_19s0014g04050  -  S-locus lectin protein kinase
## VIT_14s0030g00340  -  Sugar transporter ERD6-like 8
## VIT_15s0046g03500  -  Metal ion binding
```

```
## VIT_00s0960g00040  -  Phosphosulfolactate synthase protein
## VIT_16s0100g00290  -  L-idonate dehydrogenase
## VIT_00s0516g00020  -  Calcium-binding EF hand family
## VIT_00s0956g00020  -  Leafy cotyledon1-like protein
## VIT_01s0010g00340  -  EDA18 (embryo sac development arrest 18)
## VIT_15s0046g02810  -  R protein  PRF  disease resistance protein
## VIT_18s0001g00370  -  Ceramidase
## VIT_11s0016g02200  -  Beta-galactosidase
## VIT_09s0002g02360  -  Unknown
## VIT_13s0019g05190  -  RAB GTPase RAB_ALPHA
## VIT_15s0107g00280  -  R protein L6
## VIT_16s0098g00160  -  Receptor serine/threonine kinase
## VIT_16s0050g02540  -  Nodulin MtN3 family
## VIT_04s0210g00050  -  Strictosidine synthase
## VIT_14s0219g00040  -  Zinc finger (C3HC4-type ring finger)
## VIT_06s0080g00330  -  Esterase/lipase/thioesterase family protein
## VIT_04s0008g03510  -  Lignostilbene-alpha,beta-dioxygenase and related enzymes
## VIT_09s0002g06210  -  RPS5 (resistant to p. syringae 5)
## VIT_16s0039g01220  -  C2 domain-containing protein
## VIT_18s0041g01630  -  No hit
## VIT_04s0044g00130  -  Unknown protein
## VIT_07s0005g02290  -  Unknown protein
## VIT_16s0050g01220  -  Heavy-metal-associated domain-containing protein
## VIT_03s0063g01040  -  Peroxidase
## VIT_15s0024g00410  -  Lectin
## VIT_04s0008g05740  -  ATSYTE/NTMC2T2.1/NTMC2TYPE2.1/SYTE
## VIT_02s0012g02460  -  NADP-malic enzyme
## VIT_04s0008g03480  -  Beta-carotene 15,15'-monooxygenase
## VIT_02s0033g00290  -  Pinoresinol-lariciresinol reductase
## VIT_00s0507g00010  -  Membrane bound O-acyl transferase (MBOAT)
## VIT_04s0008g04770  -  Zinc finger protein VAR3, chloroplastic
## VIT_01s0011g00760  -  Beta-glucosidase
## VIT_15s0107g00150  -  Glutathione S-transferase 8 GSTU8
## VIT_19s0090g00510  -  Unknown protein
## VIT_03s0097g00240  -  No hit
```

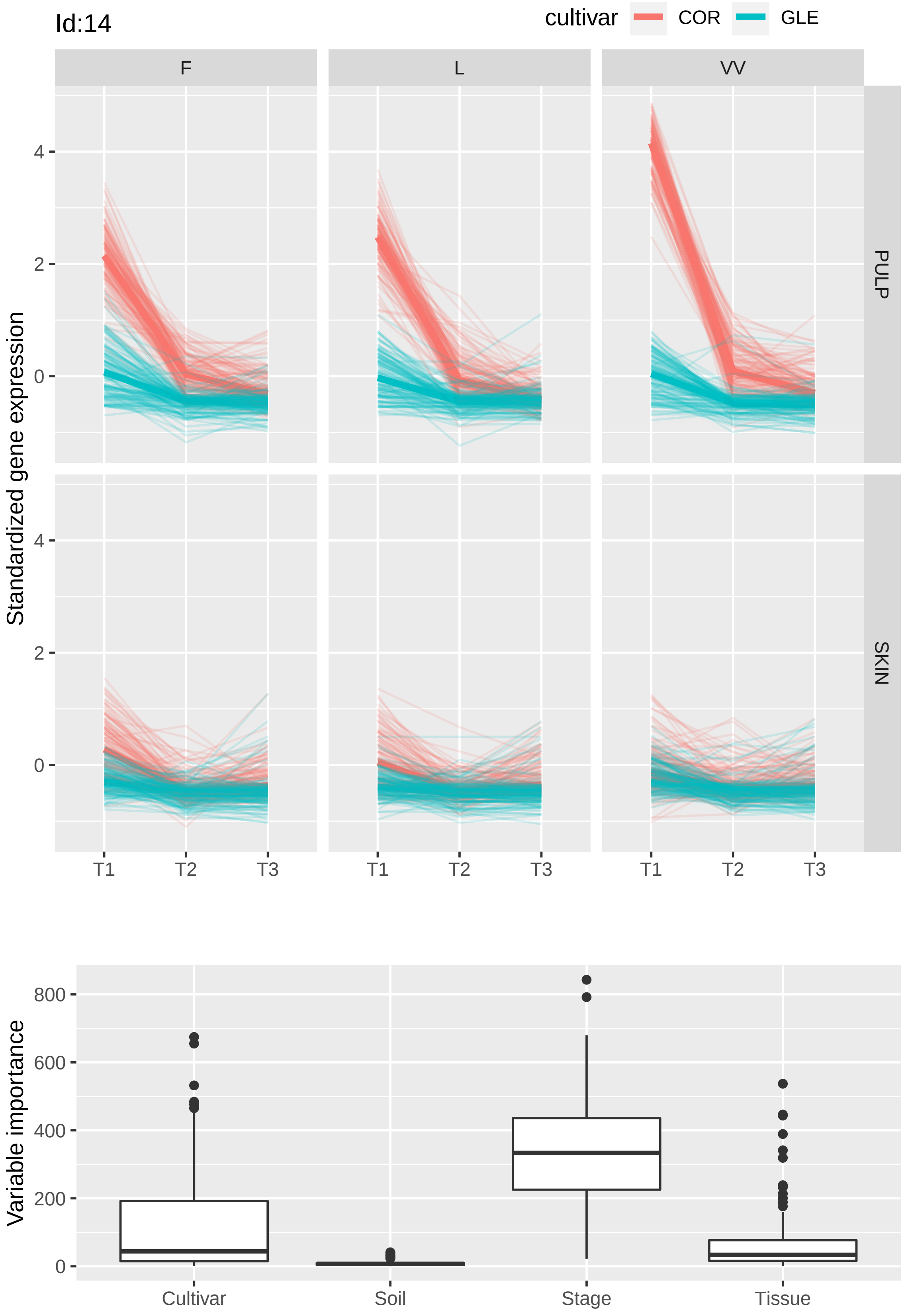

Id:14
cultivar
COR
GLE
F
L
VV
PULP
SKIN
Standardized gene expression
4
2
0
T1
T2
T3
Variable importance
800
600
400
200
0
Cultivar
Soil
Stage
Tissue

Cluster no. 46

```
## Number of genes in the cluster: 28
## Homogeneity Index:    0.73
## Variable importance for Stage:     Rank =  46  - Median =  322.4
## Variable importance for Cultivar:  Rank =  44  - Median =  51.33
## Variable importance for Tissue:    Rank =  55  - Median =    106
## Variable importance for Soil:      Rank =   5  - Median =  19.31
##
## Gene ID               Gene Annotation
## VIT_01s0011g05490  -  F-box domain containing protein
## VIT_04s0008g00960  -  Calcineurin B protein 10
## VIT_00s0226g00060  -  Unknown protein
## VIT_13s0064g00830  -  Disease resistance protein RGA2 (RGA2-blb)
## VIT_12s0028g02180  -  Phosphoenolpyruvate carboxylase.
## VIT_05s0020g05050  -  Cellulose synthase CSLE1
## VIT_14s0066g00550  -  ABC Transporter (VvTAP4 - VvABCB24)
## VIT_08s0007g05570  -  Embryo-abundant protein
## VIT_17s0000g06560  -  Retrovirus Pol polyprotein from transposon TNT 1-94
## VIT_16s0013g01340  -  Aminopeptidase N
## VIT_14s0030g02020  -  Aldo/keto reductase
## VIT_13s0019g04440  -  Zinc finger (C3HC4-type ring finger)
## VIT_16s0050g00990  -  ferric reduction oxidase 4
## VIT_16s0039g02740  -  NADH glutamate dehydrogenase [Vitis vinifera]
## VIT_05s0049g00440  -  Unknown protein
## VIT_13s0106g00020  -  RPM1 (resistance to p. syringae pv maculicola 1)
## VIT_06s0061g00600  -  Nodulin family protein
## VIT_04s0023g02020  -  replication factor C subunit 3/5
## VIT_08s0058g00950  -  Curculin-like (mannose-binding) lectin family
## VIT_04s0023g01610  -  DDM1 (decreased DNA methylation 1)
## VIT_16s0022g01650  -  Receptor serine/threonine kinase PR5K
## VIT_11s0016g04690  -  SOUL heme-binding
## VIT_15s0048g00740  -  No hit
## VIT_02s0025g03820  -  Potassium transporter 2
## VIT_10s0003g02480  -  Xyloglucan endotransglycosylase/hydrolase precursor XTH-21
## VIT_02s0025g04540  -  Beta-lactamase, class A
## VIT_09s0070g00720  -  No hit
## VIT_01s0137g00510  -  CYP71B10
```

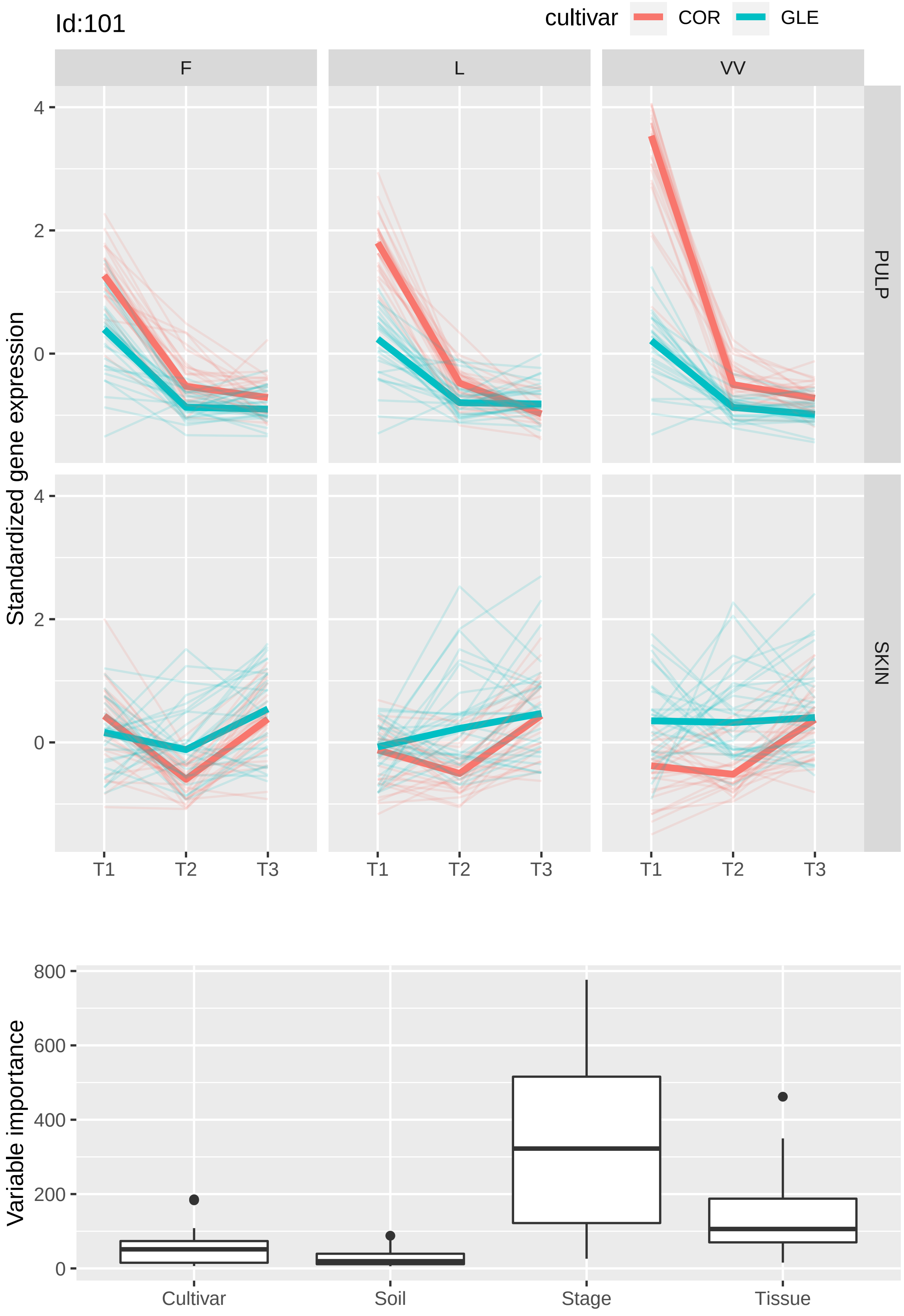
Id:101
cultivar
COR
GLE
F
L
VV
PULP
SKIN
Standardized gene expression
4
2
0
T1
T2
T3
Variable importance
800
600
400
200
0
Cultivar
Soil
Stage
Tissue

Cluster no. 47

```
## Number of genes in the cluster: 45
## Homogeneity Index:    0.85
## Variable importance for Stage:     Rank =  47  - Median =  288.5
## Variable importance for Cultivar:  Rank =  41  - Median =  59.29
## Variable importance for Tissue:    Rank =  52  - Median =  109.9
## Variable importance for Soil:      Rank =  24  - Median =   7.04
##
## Gene ID               Gene Annotation
## VIT_12s0028g00980  -  myb family
## VIT_18s0001g12710  -  No hit
## VIT_08s0032g00950  -  Unknown protein
## VIT_08s0056g01610  -  No hit
## VIT_02s0025g02960  -  Naringenin,2-oxoglutarate 3-dioxygenase
## VIT_04s0008g06570  -  Chorismate mutase, cytosolic (CM2)
## VIT_08s0007g01810  -  ANTR2 (anion transporter 2
## VIT_00s0229g00210  -  MSH4 (MUTS-like protein 4)
## VIT_10s0003g01120  -  No hit
## VIT_09s0002g09140  -  ERF/AP2 Gene Family (VvERF047)
## VIT_09s0054g00130  -  Kinesin family member 22
## VIT_09s0002g08110  -  Unknown
## VIT_05s0094g00200  -  Chitinase class IV
## VIT_14s0006g00100  -  Potassium channel SKOR
## VIT_16s0022g02150  -  Lectin
## VIT_18s0072g00540  -  Unknown
## VIT_18s0001g04780  -  (-)-germacrene D synthase (VvTPS10), (E)-a-Bergamotene Syn
## VIT_13s0067g00330  -  AUX1 auxin influx carrier protein
## VIT_13s0019g03630  -  Unknown
## VIT_01s0011g02260  -  Metal-nicotianamine transporter YSL7
## VIT_15s0048g00880  -  No hit
## VIT_08s0058g00470  -  Abscisic acid receptor PYL4  RCAR10
## VIT_08s0007g00340  -  Kinesin family member 22
## VIT_14s0068g01840  -  Unknown protein
## VIT_12s0034g01930  -  Globulin-like protein
## VIT_18s0001g12680  -  Unknown protein
## ENSRNA049996717  -
## VIT_13s0019g04290  -  No hit
## VIT_01s0011g06180  -  Blight-associated protein p12 precursor
## VIT_01s0137g00330  -  Cysteine proteinase
## VIT_17s0000g08640  -  Unknown protein
## VIT_16s0013g00410  -  No hit
## VIT_03s0063g00450  -  Alpha-amylase
## VIT_16s0098g00890  -  Harpin-induced protein
## VIT_16s0100g00510  -  F-box family protein
## VIT_03s0063g00410  -  Alpha-amylase
## VIT_02s0025g00430  -  Cellulase
## VIT_09s0054g01680  -  No hit
## VIT_16s0013g00390  -  No hit
## VIT_01s0011g03330  -  Unknown protein
## VIT_14s0068g01850  -  No hit
## VIT_15s0046g01430  -  Aldose 1-epimerase
## VIT_04s0069g00550  -  Glutamate receptor protein
## VIT_14s0068g01210  -  Sex determination protein tasselseed-2
## VIT_08s0058g01020  -  Saposin B domain-containing protein
```

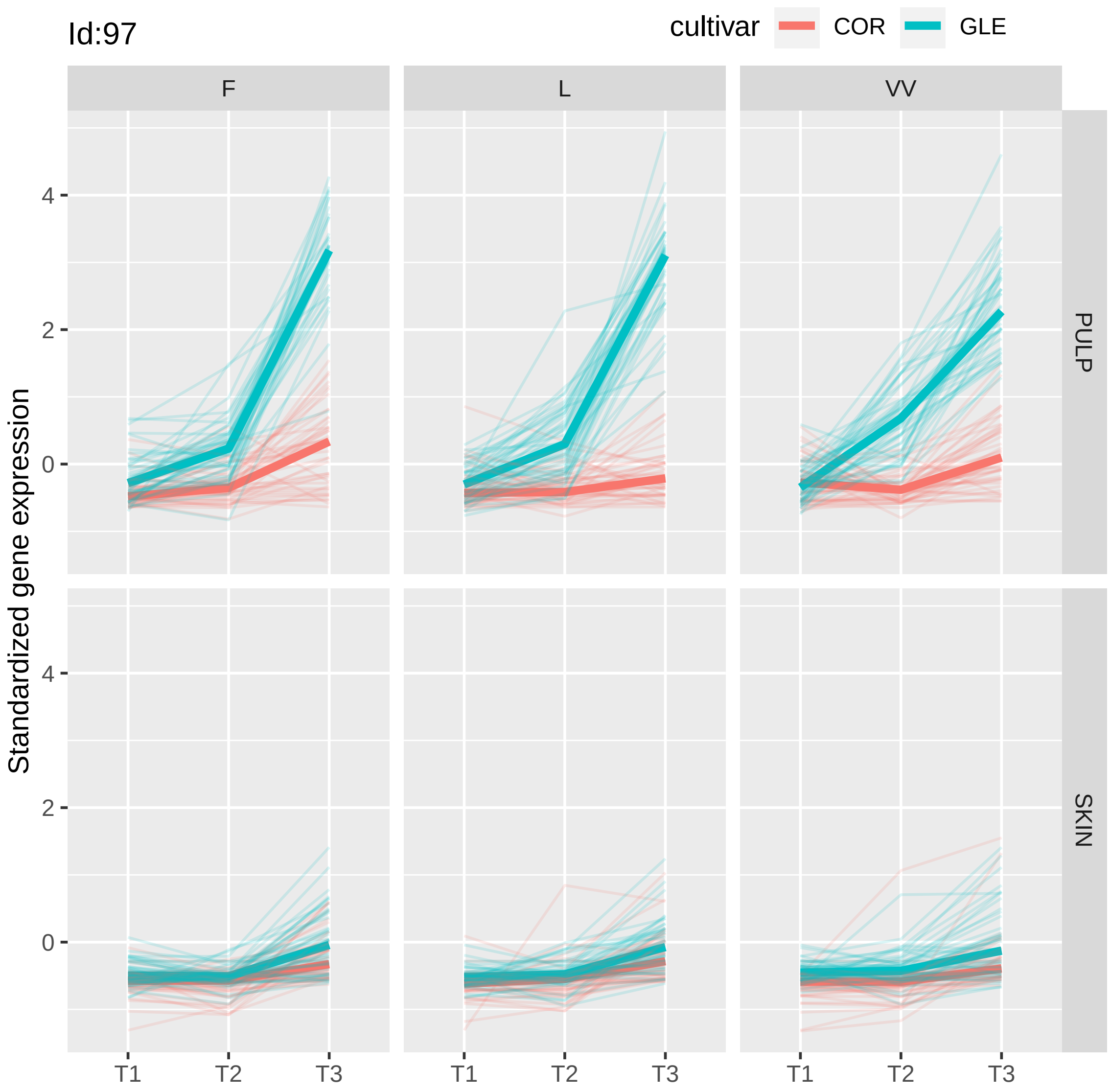
Id:97
cultivar
COR
GLE
F
L
VV
PULP
SKIN
Standardized gene expression
4
2
0
T1
T2
T3

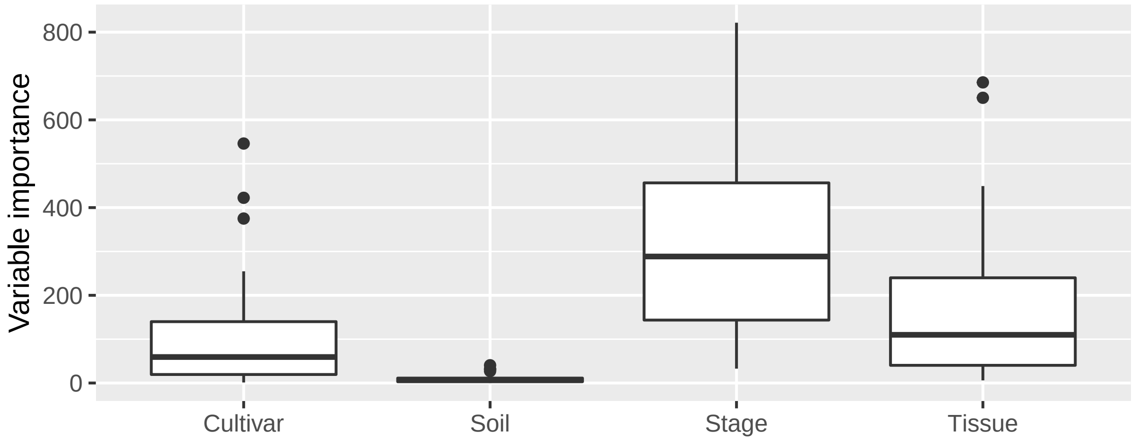
Variable importance
800
600
400
200
0
Cultivar
Soil
Stage
Tissue

Cluster no. 48

```
## Number of genes in the cluster: 37
## Homogeneity Index:    0.81
## Variable importance for Stage:     Rank =  48  - Median =  278.1
## Variable importance for Cultivar:  Rank =  13  - Median =  332.6
## Variable importance for Tissue:    Rank =  84  - Median =  21.11
## Variable importance for Soil:      Rank =  28  - Median =   6.75
##
## Gene ID              Gene Annotation
## VIT_16s0098g00900  -  Pseudo-response regulator 5 (APRR5)
## VIT_10s0003g01410  -  CBL-interacting protein kinase 20 (CIPK20)
## VIT_16s0100g00570  -  Dehydration-responsive protein
## VIT_18s0001g13770  -  Cytochrome P450, family 83, subfamily B, polypeptide 1
## VIT_02s0025g00260  -  Polygalacturonase GH28
## VIT_04s0023g01080  -  Lys Motif-Type Receptor-Like Kinase LYK4
## VIT_11s0052g00630  -  Metallothionein
## VIT_05s0020g01040  -  Unknown protein
## VIT_16s0013g00890  -  ERF/AP2 Gene Family (VvERF077)
## VIT_17s0000g06310  -  Cycling DOF factor 2
## VIT_03s0063g02530  -  Unknown protein
## VIT_07s0141g00520  -  Serine carboxypeptidase 1 precursor
## VIT_01s0146g00100  -  ACT domain containing protein (ACR4)
## VIT_12s0035g02080  -  Unknown protein
## VIT_08s0056g00140  -  Unknown protein
## VIT_01s0137g00740  -  Unknown protein
## VIT_02s0025g01430  -  Zinc finger (C3HC4-type ring finger) family protein ATL1E
## VIT_00s2240g00010  -  GAE3 (UDP-D- glcucuronate  4-epimerase 3)
## VIT_01s0011g00530  -  Ureide permease 2 (AtUPS2)
## VIT_12s0035g02100  -  Glutathione S-transferase Z1 GSTZ1
## VIT_19s0014g00450  -  PMR5 (powdery mildew resistant 5)
## VIT_18s0089g00630  -  CF4
## VIT_18s0001g11950  -  Unknown protein
## VIT_17s0000g06790  -  flavin-containing monooxygenase
## VIT_00s0322g00030  -  Alpha-6-galactosyltransferase
## VIT_14s0036g00090  -  No hit
## VIT_01s0026g01580  -  Glyoxal oxidase
## VIT_14s0036g00080  -  Unknown
## VIT_16s0050g01820  -  No hit
## VIT_16s0050g01830  -  AAA-type ATPase
## VIT_00s2526g00010  -  Endo-1,4-beta-glucanase korrigan (KOR)
## VIT_13s0106g00780  -  Hydrolase, alpha/beta fold
## VIT_01s0010g00990  -  S-adenosylmethionine decarboxylase proenzyme
## VIT_06s0004g06370  -  UDP-glucose: anthocyanidin 5,3-O-glucosyltransferase
## VIT_12s0034g01870  -  Cupin
## VIT_08s0040g01930  -  Unknown protein
## VIT_01s0010g00980  -  No hit
```

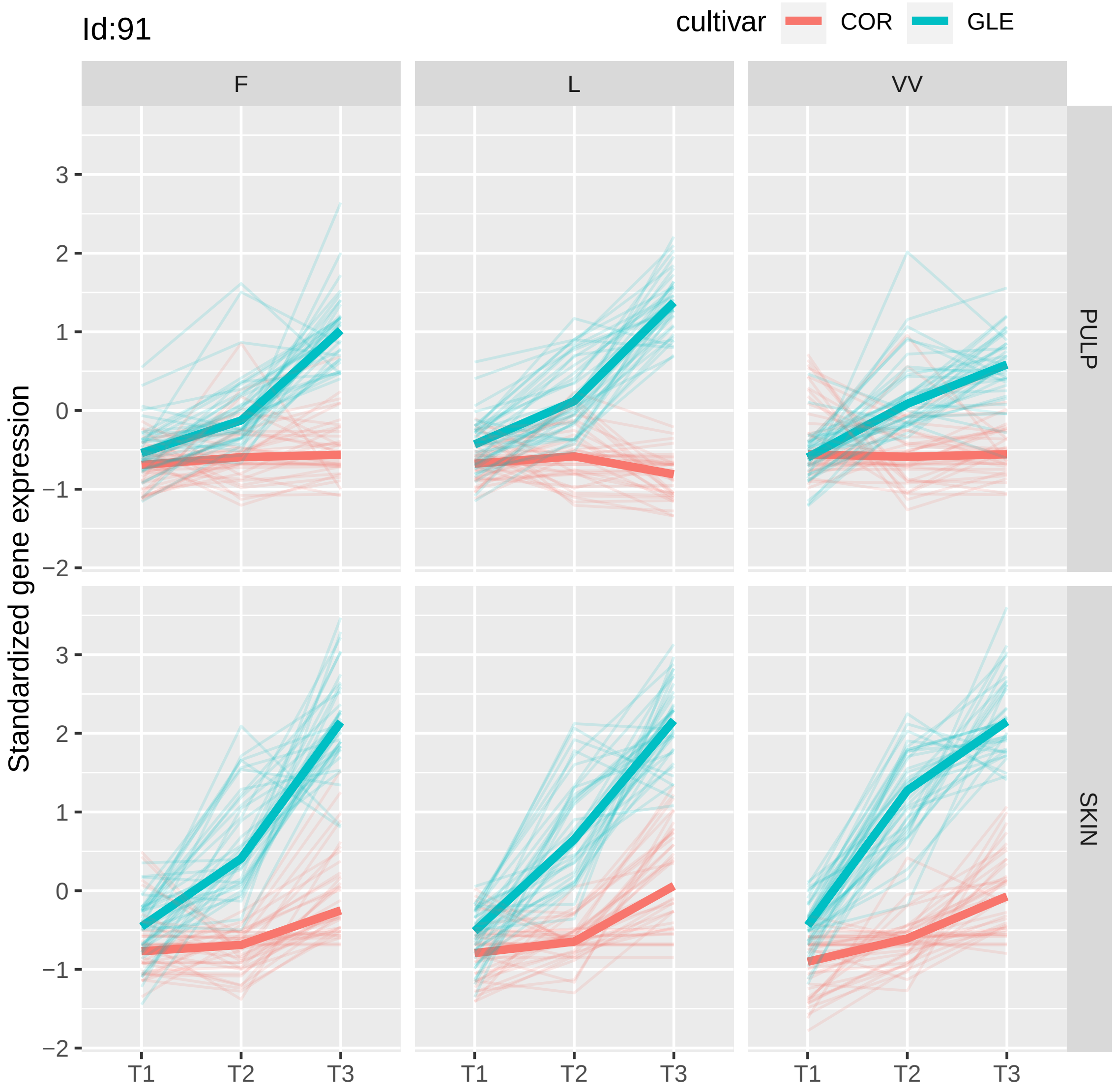

Id:91
cultivar
COR
GLE
F
L
VV
PULP
SKIN
Standardized gene expression
3
2
1
0
−1
−2
T1
T2
T3

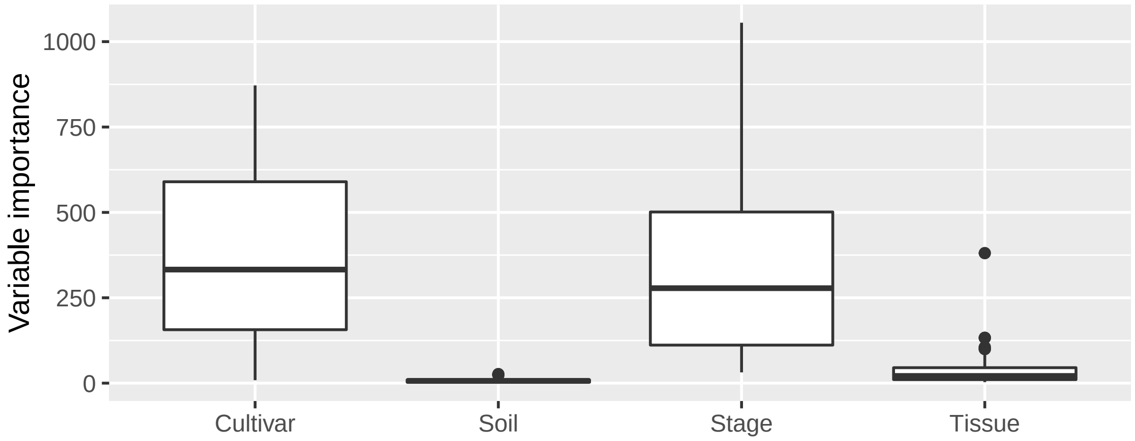

Variable importance
1000
750
500
250
0
Cultivar
Soil
Stage
Tissue

Cluster no. 49

```
## Number of genes in the cluster: 63
## Homogeneity Index:    0.75
## Variable importance for Stage:     Rank =  49  - Median =  259.3
## Variable importance for Cultivar:  Rank =  28  - Median =  144.8
## Variable importance for Tissue:    Rank =  70  - Median =  37.27
## Variable importance for Soil:      Rank =  12  - Median =   9.85
##
## Gene ID                Gene Annotation
## VIT_11s0016g05330  -  SPX2 (SYG1/Pho81/XPR1) domain-containing protein SPX2
## VIT_18s0001g14330  -  No hit
## VIT_13s0139g00340  -  R protein MLA10
## VIT_15s0024g00800  -  No hit
## VIT_09s0002g07530  -  Tetratricopeptide helical
## VIT_06s0004g06940  -  Zinc transporter ZIP6
## VIT_12s0028g01410  -  Heat shock transcription factor A4A
## VIT_05s0049g00100  -  DNA-binding protein
## VIT_05s0051g00230  -  R protein L6
## VIT_07s0191g00050  -  Peroxidase 17
## VIT_05s0062g01010  -  Aldo/keto reductase AKR
## VIT_04s0008g07010  -  Dynamin-like protein 2b
## VIT_00s0265g00070  -  Ubiquitin-conjugating enzyme E2 O
## VIT_09s0054g01820  -  Indeterminate(ID)-domain 2
## VIT_06s0004g04300  -  Histone H2B
## VIT_10s0116g01200  -  WRKY Transcription Factor (VvWRKY29)
## VIT_16s0100g00740  -  Unknown
## VIT_13s0067g02630  -  No hit
## VIT_05s0077g01260  -  Acyl-activating enzyme 7
## VIT_13s0019g03910  -  Unknown
## VIT_01s0011g00160  -  Alpha-1,4-glucan-protein synthase
## VIT_04s0008g00900  -  MYB divaricata
## VIT_06s0004g04250  -  Histone H2B
## VIT_01s0026g02460  -  Zinc finger homeobox 30
## VIT_17s0000g03780  -  No hit
## VIT_18s0001g08370  -  Glycosyl hydrolase family 10 protein
## VIT_13s0019g03130  -  Glucosyltransferase-2
## VIT_14s0068g01500  -  Glycerophosphoryl diester phosphodiesterase
## VIT_19s0014g00150  -  Purine permease 11 PUP11
## VIT_13s0158g00190  -  R protein MLA10
## VIT_13s0019g05100  -  Amino acid binding protein
## VIT_13s0019g03120  -  Glucosyltransferase-2
## VIT_18s0001g03390  -  S-receptor kinase
## VIT_06s0004g07500  -  WRKY Transcription Factor (VvWRKY16)
## VIT_10s0042g01240  -  Transducin protein
## VIT_01s0026g02660  -  Negatively light-regulated protein
## VIT_11s0118g00580  -  Unknown
## VIT_14s0030g01490  -  Cysteine synthase
## VIT_01s0011g03660  -  IMP dehydrogenase/GMP reductase
## VIT_03s0038g03690  -  Unknown protein
## VIT_06s0004g06850  -  MAPKKK14
## VIT_19s0085g01160  -  Unknown protein
## VIT_04s0069g00510  -  WD-40 repeat family protein / beige-related
## VIT_19s0014g04940  -  Chitin-inducible gibberellin-responsive protein 1
## VIT_06s0004g04550  -  Ankyrin repeat
## VIT_18s0075g00070  -  R protein L6
```

```
## VIT_19s0014g02170  -  4-amino-4-deoxychorismate lyase
## VIT_04s0008g04790  -  ABC Transporter (VvPDR29 - VvABCG59)
## VIT_05s0077g00510  -  Beta-fructofuranosidase
## VIT_10s0003g03800  -  Jasmonate ZIM domain-containing protein (VvJAZ6)
## VIT_17s0000g04360  -  Wall-associated kinase 2 (WAK2)
## VIT_12s0028g00580  -  Unknown protein
## VIT_07s0005g02100  -  Shaggy protein kinase alpha (ASK-alpha)
## VIT_13s0067g01770  -  Steroid 5alpha-reductase
## VIT_14s0006g00350  -  Glutamine synthetase cytosolic isozyme 2
## VIT_08s0056g00300  -  Unknown
## VIT_05s0020g01900  -  No hit
## VIT_08s0007g00230  -  No hit
## VIT_11s0016g01420  -  Mechanosensitive ion channel
## VIT_09s0002g03540  -  MYB divaricata
## VIT_11s0037g00470  -  No hit
## VIT_11s0037g00040  -  basic helix-loop-helix (bHLH) family
## VIT_10s0003g03910  -  TCP family transcription factor 24
```

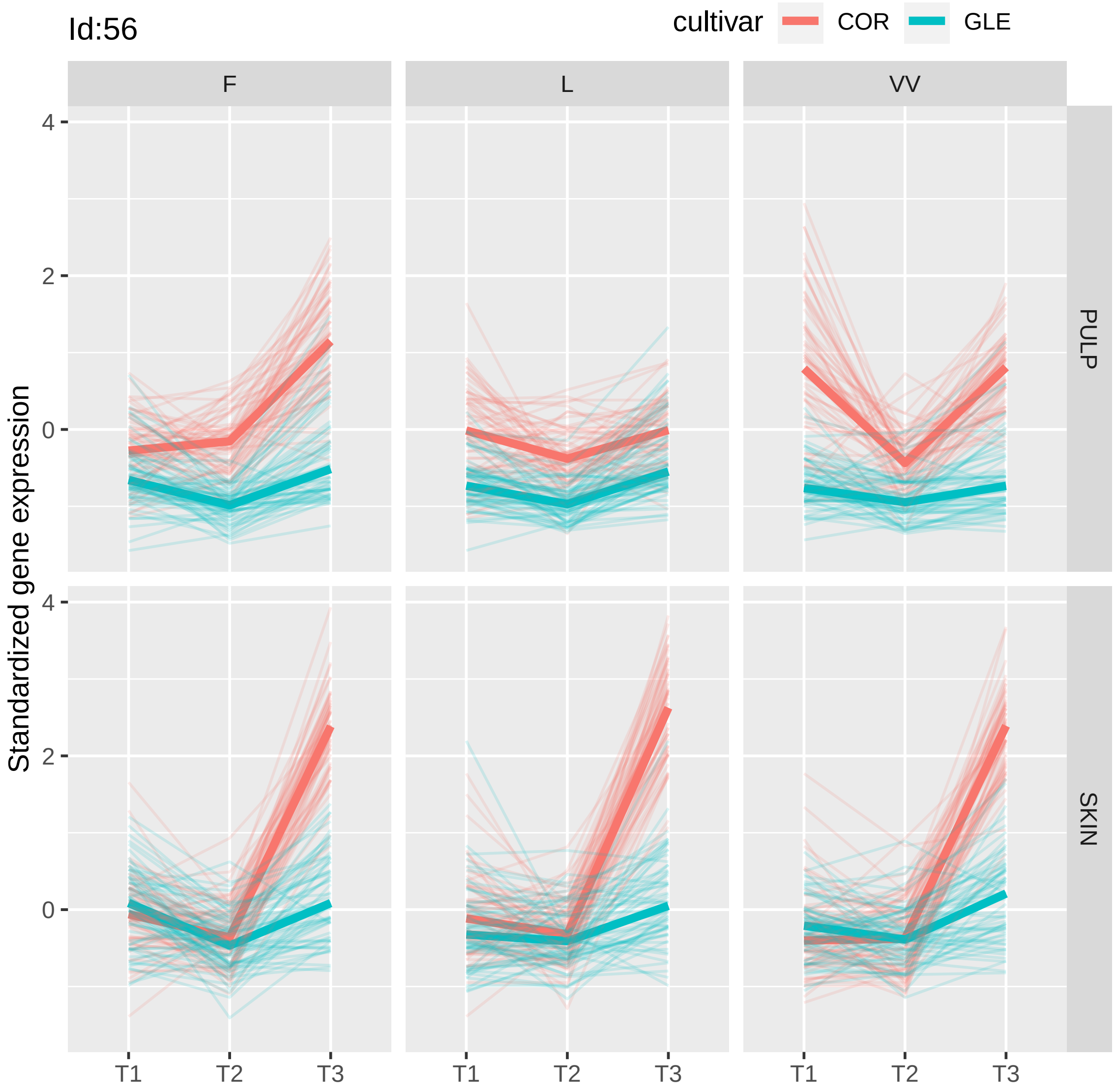

Id:56
cultivar
COR
GLE
F
L
VV
PULP
SKIN
Standardized gene expression
4
2
0
T1
T2
T3

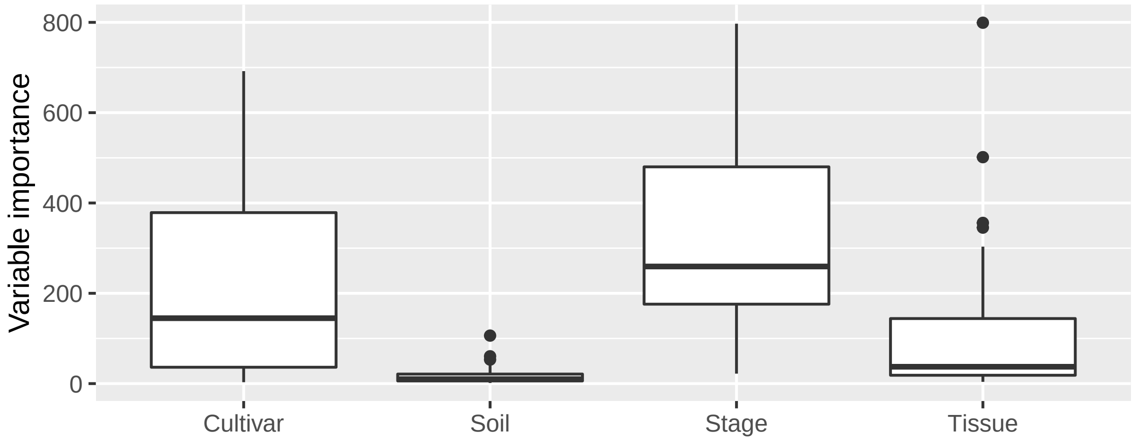

Variable importance
800
600
400
200
0
Cultivar
Soil
Stage
Tissue

Cluster no. 50

```
## Number of genes in the cluster: 9
## Homogeneity Index:    0.89
## Variable importance for Stage:     Rank =  50  - Median =    259
## Variable importance for Cultivar:  Rank =  31  - Median =  112.6
## Variable importance for Tissue:    Rank =  47  - Median =  140.7
## Variable importance for Soil:      Rank =   2  - Median =  27.97
##
## Gene ID               Gene Annotation
## ENSRNA049466084  -
## ENSRNA049465739  -
## ENSRNA049469943  -
## ENSRNA049469358  -
## ENSRNA049469936  -
## ENSRNA049469319  -
## ENSRNA049466124  -
## VIT_00s0227g00030  -  Gag-pol polyprotein
## ENSRNA049467980  -
```

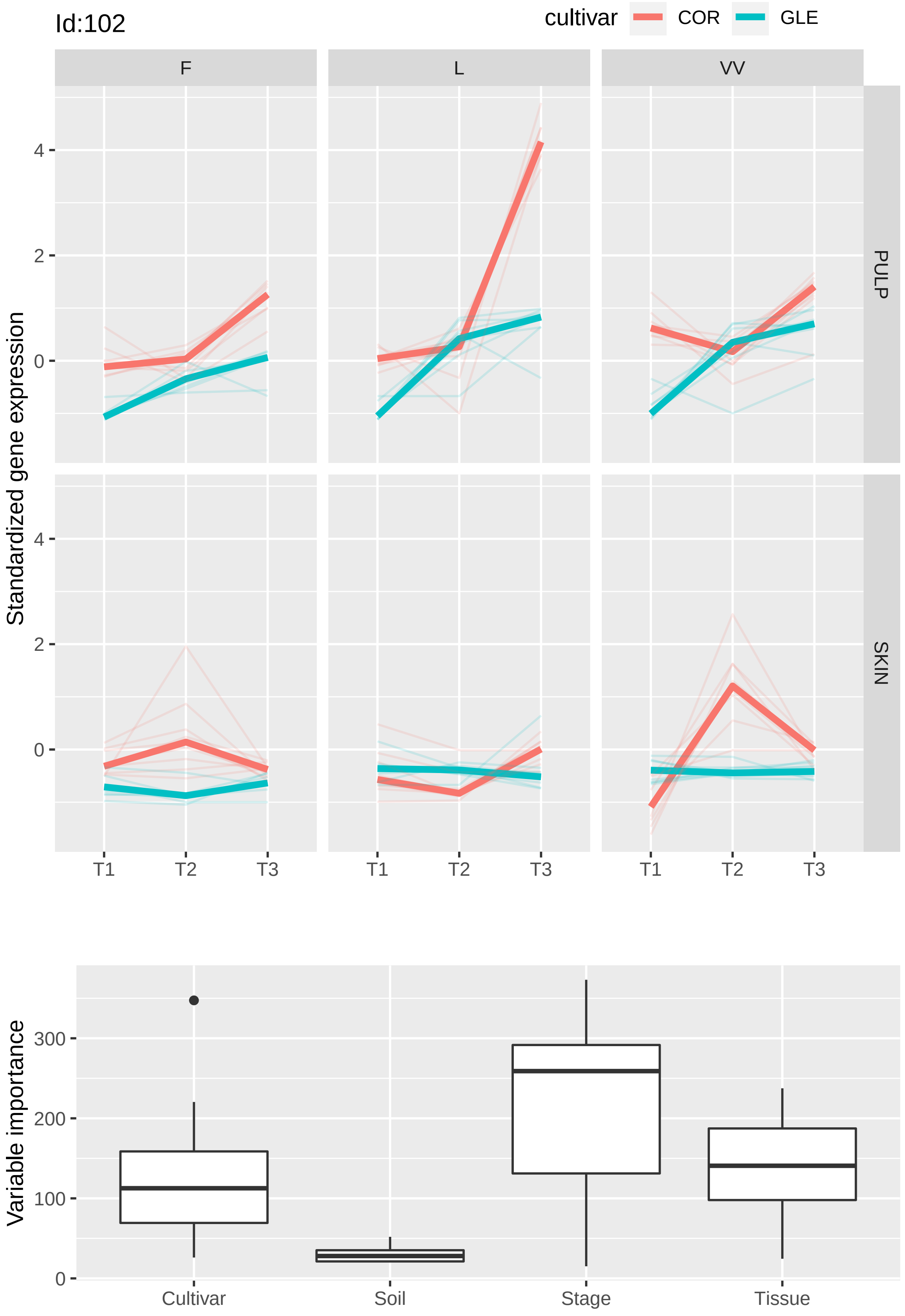

Id:102
cultivar
COR
GLE
F
L
VV
PULP
SKIN
Standardized gene expression
0
2
4
T1
T2
T3
Variable importance
0
100
200
300
Cultivar
Soil
Stage
Tissue

Cluster no. 51

```
## Number of genes in the cluster: 106
## Homogeneity Index:    0.83
## Variable importance for Stage:     Rank =  51  - Median =  224.8
## Variable importance for Cultivar:  Rank =  56  - Median =   33.7
## Variable importance for Tissue:    Rank =  51  - Median =  123.8
## Variable importance for Soil:      Rank =  22  - Median =   7.43
##
## Gene ID                Gene Annotation
## VIT_17s0000g03910  -  flavonoid 3-monooxygenase
## VIT_07s0129g00890  -  Protein kinase
## VIT_12s0059g00080  -  Ankyrin repeat
## VIT_07s0005g02450  -  Unknown protein
## VIT_12s0059g00050  -  Ankyrin repeat
## VIT_13s0067g01970  -  Laccase
## VIT_14s0006g00300  -  Nudix hydrolase 18
## VIT_17s0000g08080  -  Armadillo/beta-catenin repeat protein / U-box domain-containing protei
## VIT_19s0090g01160  -  Lipase GDSL
## VIT_16s0100g01210  -  6-phospho 3-hexuloisomerase
## VIT_16s0013g01030  -  ERF/AP2 Gene Family (VvERF106)
## VIT_15s0048g01570  -  CYP76C2
## VIT_01s0011g05190  -  Bet v I allergen
## VIT_03s0038g01150  -  Auxin-responsive
## VIT_00s0194g00160  -  Unknown protein
## VIT_00s0203g00070  -  Myb domain protein 102
## VIT_16s0013g01060  -  ERF/AP2 Gene Family (VvERF108)
## VIT_19s0085g00940  -  Myb domain protein 67
## VIT_11s0037g00870  -  ERF/AP2 Gene Family (VvAP2-01)
## VIT_02s0033g01000  -  Anthraniloyal-CoA: methanol anthraniloyal transferase
## VIT_17s0000g04400  -  Wall-associated kinase 1 (WAK1)
## VIT_14s0006g01120  -  Lipase GDSL
## VIT_16s0050g00200  -  HcrVf3 protein
## VIT_11s0016g01320  -  VvMybPA2
## VIT_00s0480g00080  -  Polyphenol oxidase II, chloroplast precursor
## VIT_00s0250g00050  -  ARF GTPase activator, ARF-GAP Domain 6
## VIT_04s0023g01850  -  Hydrolase, alpha/beta fold
## VIT_16s0050g00180  -  HcrVf1 protein
## VIT_00s0400g00030  -  HcrVf1 protein
## VIT_03s0038g04220  -  Dihydroflavonol-4-reductase
## VIT_07s0255g00030  -  Lipase GDSL
## VIT_13s0320g00050  -  Lipid transfer protein 3
## VIT_19s0014g02460  -  Plastocyanin domain-containing protein
## VIT_17s0000g04150  -  leucoanthocyanidin reductase 2 (VvLAR2) [Vitis vinifera] GENE ID: 1002
## VIT_07s0151g00700  -  EDA4 (embryo sac development arrest 4)
## VIT_17s0000g00950  -  Hydroxycinnamoyl-CoA quinate hydroxycinnamoyltransferase
## VIT_16s0013g00590  -  Plastocyanin domain-containing protein
## VIT_08s0007g06440  -  No hit
## VIT_10s0003g03650  -  Beta-amyrin synthase
## VIT_12s0055g00250  -  UDP-glucose glucosyltransferase
## VIT_10s0003g03530  -  Lupeol synthase
## VIT_17s0000g01630  -  Calmodulin CML37
## VIT_02s0025g01730  -  3-oxoacyl-[acyl-carrier-protein] synthase
## VIT_10s0003g03660  -  Beta-amyrin synthase
## VIT_00s0294g00100  -  Brassinosteroid insensitive 1-associated receptor kinase 1
## VIT_10s0092g00220  -  Integral membrane family protein UPF0497
```

```
## VIT_08s0040g02840  -  ABC Transporter (VvWBC21 - VvABCG21)
## VIT_14s0036g01310  -  Disease resistance protein RPS2
## VIT_18s0072g01250  -  Desulfoglucosinolate sulfotransferase 16
## VIT_10s0003g03520  -  Beta-amyrin synthase
## VIT_12s0059g02640  -  Uclacyanin I
## VIT_00s0404g00030  -  No hit
## VIT_05s0077g02000  -  Epoxide hydrolase
## VIT_00s0407g00050  -  No hit
## VIT_04s0023g01240  -  Anthocyanidin 3-O-glucosyltransferase
## VIT_01s0011g02060  -  CYP86B1
## VIT_01s0026g00570  -  Bet v I allergen
## VIT_14s0030g00430  -  Unknown protein
## VIT_19s0014g01540  -  ABC Transporter (VvWBC9 - VvABCG9)
## VIT_13s0019g01990  -  Phospholipid/glycerol acyltransferase
## VIT_11s0016g05550  -  Plastocyanin domain-containing protein
## VIT_06s0004g06210  -  CYP86A1
## VIT_02s0033g01030  -  Anthraniloyal-CoA: methanol anthraniloyal transferase
## VIT_01s0011g02410  -  Unknown
## VIT_16s0098g00030  -  Receptor kinase LRK10
## VIT_13s0067g02360  -  Peroxidase, class III
## VIT_17s0000g01090  -  Acyl-(acyl carrier protein) thioesterase
## VIT_15s0046g00510  -  Wax synthase
## VIT_04s0008g05440  -  Ethylene-responsive transcription factor SHINE 3
## VIT_04s0069g00330  -  Glutamate receptor 2.8
## VIT_13s0067g03750  -  ABC Transporter (VvWBC27 - VvABCG27)
## VIT_00s1557g00020  -  Lectin-receptor like protein kinase 3
## VIT_12s0057g00430  -  Auxin-responsive
## VIT_15s0021g01260  -  Unknown
## VIT_17s0000g06290  -  Lipase GDSL
## VIT_03s0038g00340  -  Salt tolerance homolog2
## VIT_08s0007g02700  -  Secoisolariciresinol dehydrogenase
## VIT_06s0080g00120  -  Male sterility 2 (MS2) Acyl-CoA reductase
## VIT_12s0059g00110  -  Ankyrin repeat
## VIT_12s0059g00100  -  Ankyrin repeat
## VIT_07s0197g00180  -  No hit
## VIT_15s0046g00500  -  Wax synthase
## VIT_15s0021g01390  -  Uclacyanin 3
## VIT_18s0001g02540  -  ARR9 typeA
## VIT_14s0036g01100  -  F-box family protein
## VIT_02s0033g00920  -  No hit
## VIT_17s0000g03660  -  NAC domain-containing protein (VvNAC06)
## VIT_18s0001g10830  -  Patatin
## VIT_00s0258g00090  -  Brassinosteroid insensitive 1-associated receptor kinase 1
## VIT_13s0047g00080  -  ZIFL1 (Zinc induced facilitator 1)
## VIT_05s0049g01560  -  Unknown protein
## VIT_14s0006g02630  -  Receptor-like kinase ARK1AS
## VIT_01s0011g03500  -  Homocysteine S-methyltransferase 1
## VIT_08s0007g02920  -  ATAN11 (ANTHOCYANIN11)
## VIT_18s0001g00210  -  Cytokinin riboside 5'-monophosphate phosphoribohydrolase LOG5
## VIT_19s0027g00040  -  CYP72A59
## VIT_06s0009g02010  -  Anthocyanin 3-O-galactosyltransferase
## VIT_14s0066g01480  -  Cis-zeatin O-beta-D-glucosyltransferase
## VIT_17s0000g02930  -  Glutathione S-transferase 25 GSTU7
## VIT_08s0007g02000  -  Glycerol-3-phosphate acyltransferase 8
```

```
## VIT_16s0148g00330  -  Receptor kinase LRK10
## VIT_00s0218g00190  -  UDP-rhamnose:rhamnosyltransferase
## VIT_00s0194g00180  -  Unknown protein
## VIT_00s1830g00010  -  Ser/Thr receptor-like kinase1
## VIT_05s0049g00220  -  2-oxoglutarate-dependent dioxygenase
## VIT_06s0009g03680  -  F-box family protein
```

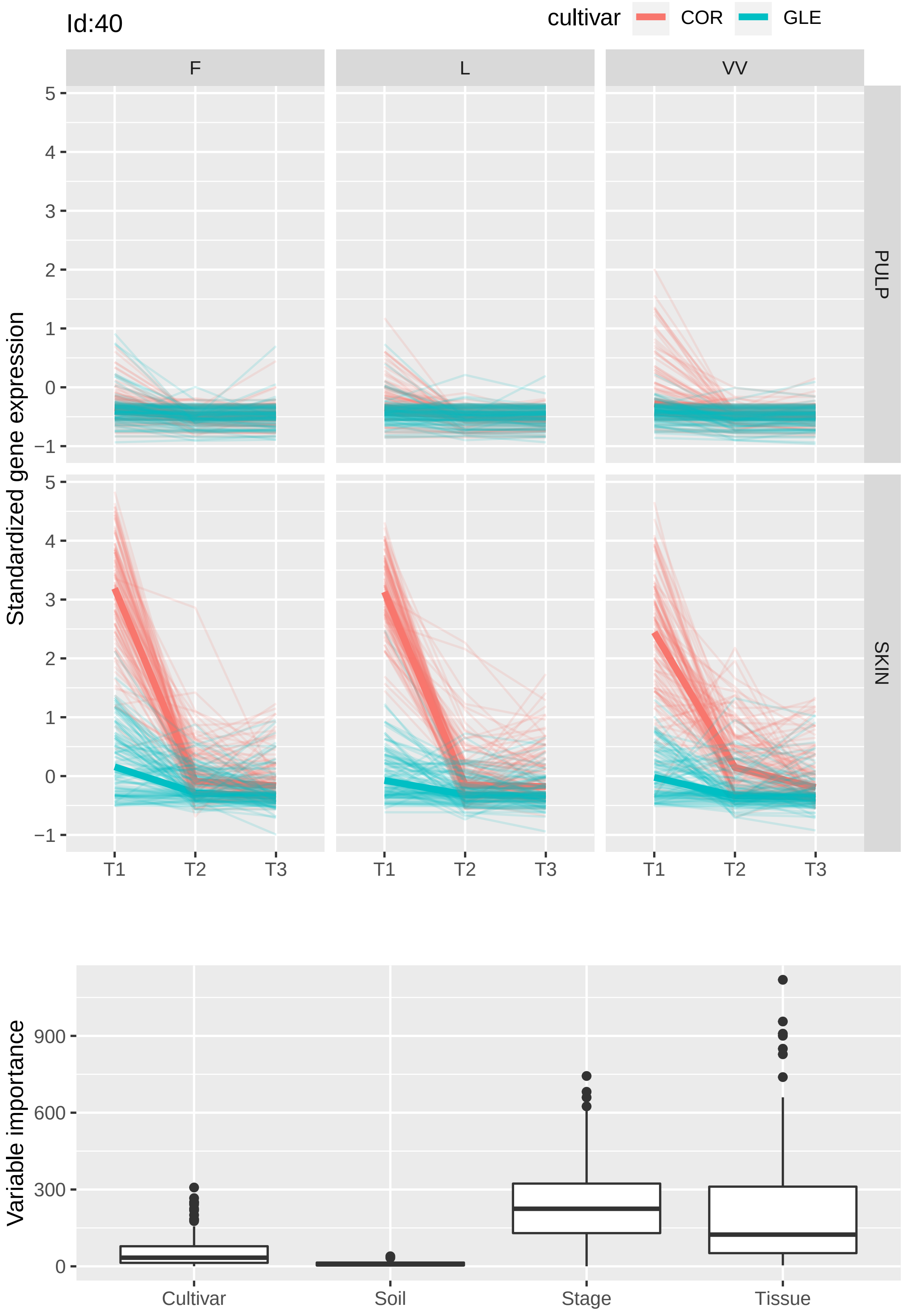

Id:40
cultivar
COR
GLE
F
L
VV
PULP
SKIN
Standardized gene expression
5
4
3
2
1
0
−1
T1
T2
T3
Variable importance
900
600
300
0
Cultivar
Soil
Stage
Tissue

Cluster no. 52

```
## Number of genes in the cluster: 93
## Homogeneity Index:    0.73
## Variable importance for Stage:     Rank =  52  - Median =  219.8
## Variable importance for Cultivar:  Rank =  12  - Median =  386.1
## Variable importance for Tissue:    Rank =  83  - Median =   21.9
## Variable importance for Soil:      Rank =  35  - Median =   6.17
##
## Gene ID                Gene Annotation
## VIT_10s0116g00040  -  No hit
## VIT_14s0068g01760  -  Alcohol dehydrogenase
## VIT_05s0029g01040  -  Aspartate aminotransferase
## VIT_18s0001g10030  -  WRKY Transcription Factor (VvWRKY54)
## VIT_01s0137g00560  -  CYP71B34
## VIT_06s0061g00180  -  WD40
## VIT_02s0087g00910  -  (9,10) (9`,10`) cleavage dioxygenase (CCD4) (VvCCD4a)
## VIT_18s0001g02780  -  Lipase GDSL
## VIT_07s0104g01550  -  Transcription termination factor mitochondrial mTERF
## VIT_05s0062g00140  -  PRD1
## VIT_18s0117g00130  -  Pi starvation-induced protein
## VIT_01s0137g00540  -  CYP71E1
## VIT_03s0088g00250  -  Isoflavone reductase
## VIT_07s0130g00380  -  DNA (cytosine-5)-methyltransferase AthI
## VIT_06s0080g00270  -  Zinc finger (C2H2 type) protein (WIP3)
## VIT_13s0047g01070  -  Calcium-binding EF-hand
## VIT_02s0087g00920  -  No hit
## VIT_12s0059g01800  -  Unknown protein
## VIT_06s0080g00240  -  DTDP-glucose 4-6-dehydratase
## VIT_19s0014g03130  -  Stem-specific protein TSJT1
## VIT_00s2395g00010  -  No hit
## VIT_17s0000g07540  -  paired amphipathic helix protein Sin3a
## VIT_12s0034g01890  -  Cupin region
## VIT_05s0020g03480  -  Ribosomal protein L18P/L5E
## VIT_08s0058g01250  -  Unknown protein
## VIT_04s0008g03090  -  Unknown protein
## VIT_02s0012g01770  -  Rad1
## VIT_17s0000g03540  -  DNA binding
## VIT_02s0025g01450  -  Unknown protein
## VIT_08s0007g03870  -  Phytosulfokines PSK1
## VIT_08s0058g01260  -  No hit
## VIT_15s0021g02670  -  Expansin  (VvEXPB3)
## VIT_15s0046g03180  -  Remorin
## VIT_14s0108g00990  -  No hit
## VIT_03s0088g00220  -  Replication factor A 1, rfa1
## VIT_04s0044g00920  -  Ankyrin
## VIT_00s0532g00030  -  Thioredoxin H
## VIT_15s0046g02110  -  Late embryogenesis abundant protein Lea14-A
## VIT_16s0039g00870  -  CYP89A5
## VIT_14s0068g01400  -  UPF0497 family
## VIT_06s0061g01270  -  DEMETER protein
## VIT_06s0004g02560  -  Kiwellin Ripening-related protein grip22
## VIT_03s0063g01820  -  AOS (allene oxide synthase)
## VIT_05s0029g00810  -  Unknown
## VIT_06s0004g02570  -  Kiwellin Ripening-related protein grip22
## VIT_08s0007g02770  -  IAA-amino acid hydrolase 1 (ILR1)
```

```
## VIT_01s0010g02840  -  Chaperone BCS1 mitochondrial
## VIT_00s0347g00030  -  Zinc finger (B-box type)
## VIT_18s0001g13990  -  No hit
## VIT_16s0039g00910  -  CYP89A28
## VIT_17s0000g07030  -  Cis-zeatin O-beta-D-glucosyltransferase
## VIT_13s0047g00490  -  No hit
## VIT_12s0034g01950  -  Legumin
## VIT_17s0000g07070  -  UDP-glucoronosyl and UDP-glucosyl transferase
## VIT_12s0034g01980  -  Globulin-like protein
## VIT_00s1364g00010  -  Isochorismatase hydrolase
## VIT_15s0048g02990  -  AAA-type ATPase
## VIT_08s0040g02260  -  Protamine P1
## VIT_03s0088g00310  -  No hit
## VIT_12s0055g00800  -  Arachidonic acid-induced DEA1
## VIT_00s0271g00090  -  Unknown protein
## VIT_16s0039g01080  -  U3 small nucleolar RNA-associated protein 22
## VIT_19s0014g04180  -  Curculin (mannose-binding) lectin
## VIT_05s0020g02340  -  Methionine sulfoxide reductase
## VIT_12s0034g01970  -  Cupin
## VIT_18s0001g01380  -  Oxidoreductase N-terminal domain-containing
## VIT_02s0025g02310  -  LHW (LONESOME HIGHWAY)
## VIT_09s0002g00250  -  Disease resistance protein (NBS-LRR class)
## VIT_05s0049g01980  -  3-isopropylmalate dehydratase large subunit 2
## VIT_05s0029g00830  -  Unknown protein
## VIT_14s0006g00660  -  No hit
## VIT_12s0034g01910  -  Cupin family protein
## VIT_03s0088g00060  -  Isoflavone reductase
## VIT_04s0008g02800  -  SAUR_D
## VIT_05s0077g01330  -  Unknown
## VIT_02s0087g01020  -  CRK10 (cysteine-rich RLK10)
## VIT_00s0160g00120  -  No hit
## VIT_03s0088g00140  -  Isoflavone reductase protein 2
## VIT_09s0002g03300  -  disease resistance protein (NBS-LRR class)
## VIT_18s0041g00740  -  UDP-glucose: anthocyanidin 5,3-O-glucosyltransferase
## VIT_00s0801g00020  -  No hit
## VIT_12s0034g01900  -  Globulin-like protein
## VIT_13s0064g00520  -  furcatin hydrolase
## VIT_07s0005g02800  -  ADP-glucose pyrophosphorylase large subunit 2
## VIT_12s0034g01940  -  No hit
## VIT_13s0064g00480  -  Beta-primeverosidase
## VIT_05s0029g00740  -  Unknown protein
## VIT_14s0219g00300  -  No hit
## VIT_12s0034g00930  -  Band 7 family
## VIT_13s0019g04270  -  No hit
## VIT_07s0095g00660  -  Curculin (mannose-binding) lectin
## VIT_09s0002g00270  -  R protein disease resistance protein
## VIT_12s0057g01130  -  R protein disease resistance protein
```

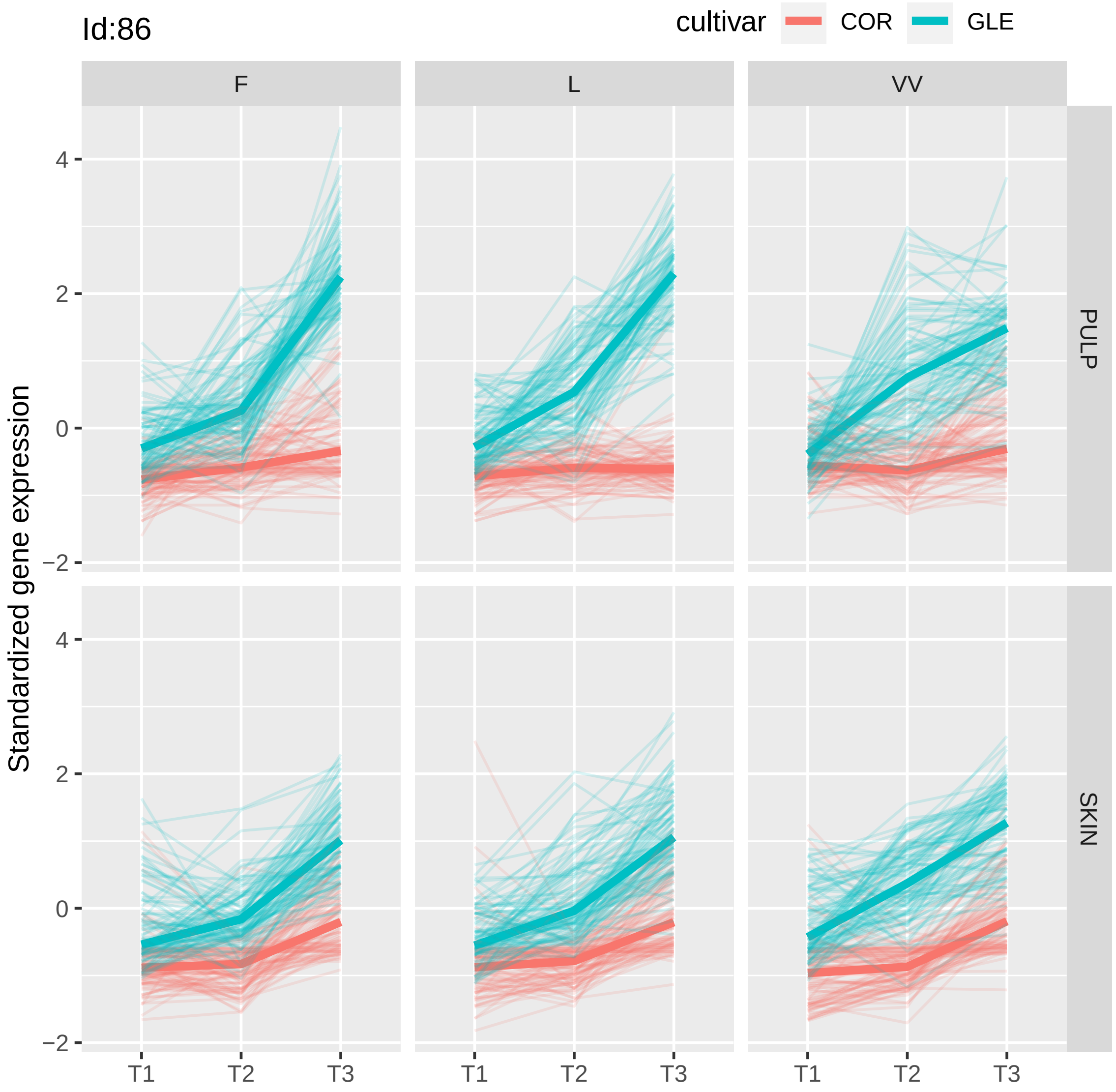

Id:86
cultivar
COR
GLE
F
L
VV
PULP
SKIN
Standardized gene expression
4
2
0
−2
T1
T2
T3

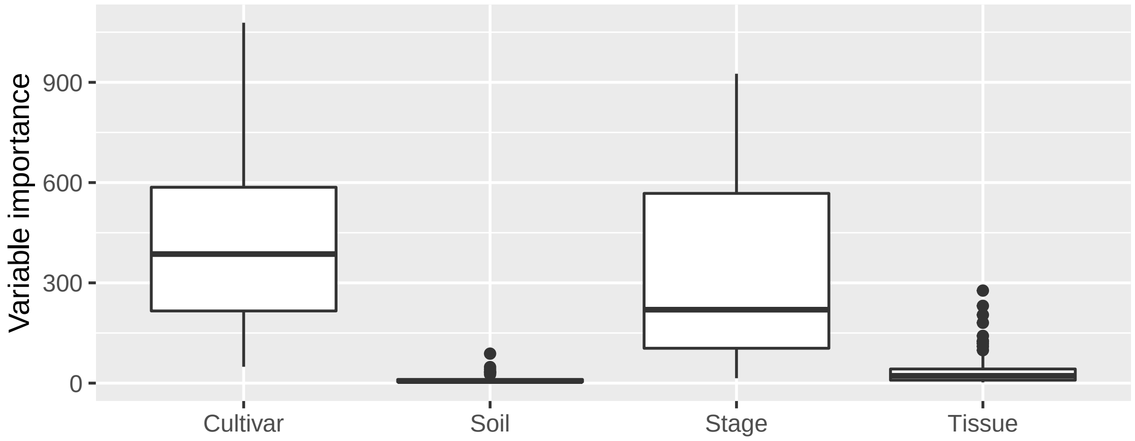

Variable importance
900
600
300
0
Cultivar
Soil
Stage
Tissue

Cluster no. 53

```
## Number of genes in the cluster: 53
## Homogeneity Index:    0.89
## Variable importance for Stage:     Rank =  53  - Median =  197.5
## Variable importance for Cultivar:  Rank =  74  - Median =  13.22
## Variable importance for Tissue:    Rank =  17  - Median =  687.9
## Variable importance for Soil:      Rank =  94  - Median =    3.3
##
## Gene ID               Gene Annotation
## VIT_17s0000g04270  -  Peptidyl-prolyl cis-trans isomerase B
## VIT_12s0134g00400  -  Zinc finger (B-box type)
## VIT_09s0054g01760  -  Unknown protein
## VIT_15s0021g00890  -  Ring-H2 zinc finger protein ATL4
## VIT_09s0002g01030  -  Subtilisin serine proteinase
## VIT_13s0019g03060  -  Glucosyltransferase-2
## VIT_07s0095g00160  -  Serine/threonine kinase BRLK
## VIT_11s0016g00130  -  Omega-6 fatty acid desaturase, chloroplast (FAD6) (FADC)
## VIT_11s0016g05520  -  Plastocyanin domain-containing protein
## VIT_12s0035g00270  -  ferredoxin, chloroplast (PETF)
## VIT_18s0001g11710  -  Photosystem II PsbO protein
## VIT_13s0064g01080  -  Thylakoid lumenal 19 kDa protein, chloroplast precursor
## VIT_05s0020g02480  -  Glutamine synthetase
## VIT_18s0164g00030  -  ATP synthase delta chain, chloroplast precursor
## VIT_11s0052g00540  -  ABC protein 3 ATNAP3 non-intrinsic
## VIT_01s0011g00460  -  Unknown protein
## VIT_14s0066g00160  -  Ubiquinone/menaquinone biosynthesis methyltransferase UbiE
## VIT_11s0065g01100  -  Pentatricopeptide (PPR) repeat-containing protein
## VIT_04s0023g03610  -  MLO4 (mildew resistance locus O 4)
## VIT_11s0016g00580  -  Unknown
## VIT_19s0015g00230  -  Zinc transporter ZIP11
## VIT_13s0064g00900  -  Unknown protein
## VIT_18s0001g08390  -  Unknown
## VIT_04s0008g05590  -  F-type H+-transporting ATPase b chain CfoII
## VIT_02s0087g00380  -  No hit
## VIT_08s0007g07470  -  Galactose mutarotase
## VIT_18s0089g01420  -  Hydrolase, alpha/beta fold
## VIT_06s0061g00620  -  Short-chain dehydrogenase carbonyl reductase 3
## VIT_06s0004g01390  -  S-receptor kinase
## VIT_03s0038g00270  -  Root phototropism protein 3 (Non-phototropic hypocotyl protein 3)
## VIT_06s0004g05230  -  Plastid transcriptionally active18
## VIT_04s0044g00730  -  S-receptor kinase
## VIT_16s0100g00330  -  Unknown protein
## VIT_08s0040g01470  -  Cis-zeatin O-beta-D-glucosyltransferase
## VIT_07s0104g00840  -  RKF3 (receptor-like kinase IN in flowers 3)
## VIT_11s0016g00120  -  Omega-6 fatty acid desaturase, chloroplast (FAD6) (FADC)
## VIT_15s0048g02980  -  Unknown protein
## VIT_08s0007g01570  -  fructose 1,6-bisphosphatase
## VIT_03s0097g00600  -  Octicosapeptide/Phox/Bem1p (PB1) domain-containing protein
## VIT_12s0055g00480  -  Amine oxidase
## VIT_12s0028g00320  -  LHB1B1
## VIT_07s0031g02560  -  UVB-resistance protein UVR8
## VIT_14s0081g00710  -  Unknown protein
## VIT_11s0037g00070  -  Aldehyde Dehydrogenase (VvALDHI1B1)
## VIT_06s0004g01400  -  S-receptor kinase
## VIT_08s0007g03250  -  Unknown protein
```

```
## VIT_13s0064g00790  -  No hit
## VIT_03s0097g00510  -  Oligopeptide transporter OPT4
## VIT_05s0049g01760  -  Proton gradient regulation 5 (PGR5)
## VIT_12s0028g01640  -  S-receptor kinase
## VIT_05s0049g01520  -  Cyanate hydratase
## VIT_06s0004g01380  -  S-receptor kinase
## VIT_07s0005g03960  -  Peptide transporter protein 3
```

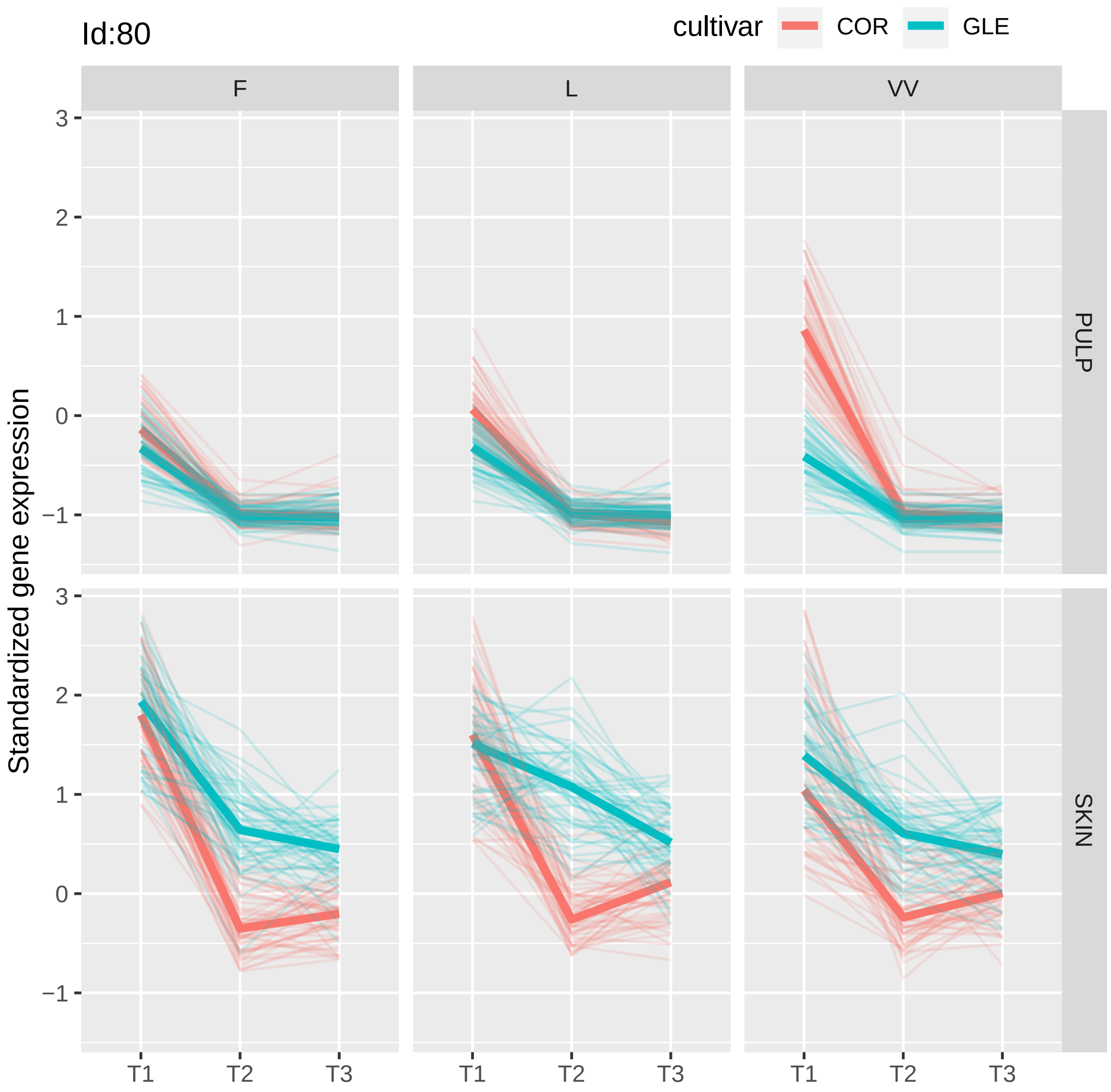
Id:80
cultivar
COR
GLE
F
L
VV
PULP
SKIN
Standardized gene expression
3
2
1
0
−1
T1
T2
T3

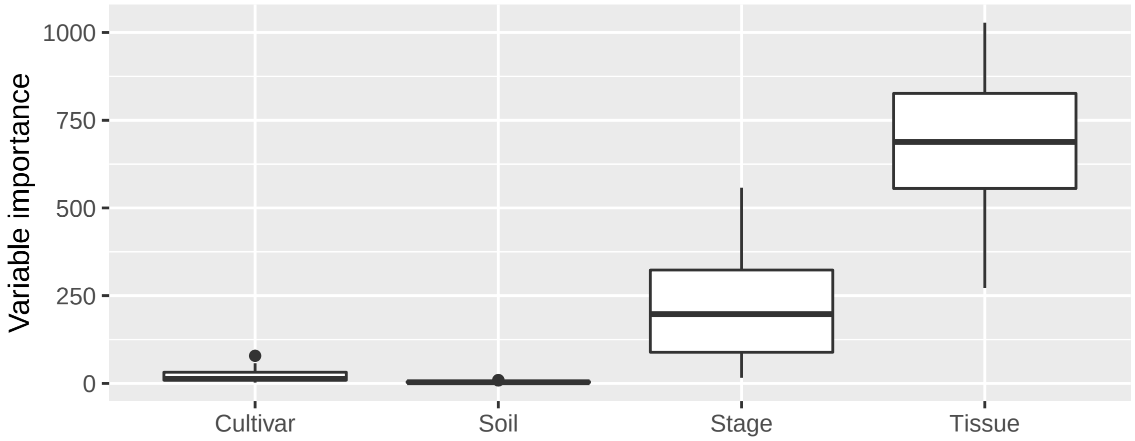
Variable importance
1000
750
500
250
0
Cultivar
Soil
Stage
Tissue

Cluster no. 54

```
## Number of genes in the cluster: 50
## Homogeneity Index:    0.75
## Variable importance for Stage:     Rank =  54  - Median =  188.2
## Variable importance for Cultivar:  Rank =  37  - Median =  82.69
## Variable importance for Tissue:    Rank =  28  - Median =    405
## Variable importance for Soil:      Rank =  69  - Median =   4.76
##
## Gene ID               Gene Annotation
## VIT_01s0137g00200  -  Unknown protein
## VIT_17s0000g08240  -  HT1 (high leaf temperature 1)
## VIT_12s0059g00030  -  Unknown protein
## VIT_14s0083g01110  -  Brassinosteroid-6-oxidase
## VIT_02s0025g03030  -  Zinc finger (C3HC4-type ring finger)
## VIT_12s0059g02730  -  Unknown protein
## VIT_01s0011g06240  -  Cytochrome B561
## VIT_14s0036g01180  -  RPS2 (resistant to p. syringae 2)
## VIT_14s0171g00210  -  Ankyrin
## VIT_13s0019g01040  -  Unknown protein
## VIT_06s0004g02230  -  Unknown protein
## VIT_18s0001g03180  -  Nodulin MtN21 family
## VIT_18s0001g03880  -  Polcalcin
## VIT_12s0035g01630  -  RPS2 (resistant to p. syringae 2)
## VIT_02s0025g04200  -  Unknown
## VIT_19s0085g00650  -  Cation efflux family
## VIT_18s0001g13360  -  Nodulin MtN21 family
## VIT_10s0003g01890  -  RKF1 (receptor-like kinase in flowers 1)
## VIT_10s0003g00390  -  Glutaredoxin
## VIT_02s0025g00950  -  ABC Transporter (VvMRP13 - VvABCC13)
## VIT_04s0023g01490  -  Ovate family protein 7 OFP7
## VIT_01s0010g01830  -  Regulator of nonsense transcripts 1
## VIT_18s0001g02570  -  IAA-amino acid hydrolase 6
## VIT_16s0098g00600  -  No hit
## VIT_18s0001g02520  -  Hypoxanthine phosphoribosyltransferase
## VIT_09s0002g01520  -  Unknown protein
## VIT_12s0121g00240  -  MuDR transposase; Zinc finger, SWIM-type; Malate synthase-like
## VIT_09s0002g03340  -  Unknown
## VIT_11s0016g05320  -  Peroxidase
## VIT_12s0034g00760  -  R protein L6
## VIT_14s0030g02110  -  Ca2+-transporting ATPase type 2 isoform 8
## VIT_03s0091g01050  -  Nucleobase-ascorbate transporter 4 (NAT4)
## VIT_05s0049g01950  -  Tetratricopeptide helical
## VIT_13s0064g00550  -  R protein MLA10
## VIT_12s0059g01280  -  Nucleobase-ascorbate transporter 1 (NAT1)
## VIT_02s0025g03330  -  H(+)-ATPase 4 AHA4
## VIT_08s0007g03690  -  Inosine-uridine preferring nucleoside hydrolase family protein
## VIT_14s0068g01860  -  Unknown protein
## VIT_12s0178g00190  -  Unknown
## VIT_02s0012g00470  -  Unknown
## VIT_00s0211g00030  -  Symbiosis receptor kinase
## VIT_05s0029g01480  -  Receptor kinase TRKe
## VIT_16s0050g01880  -  Receptor kinase homolog LRK10
## VIT_01s0010g00020  -  DNA-3-methyladenine glycosidase I
## VIT_13s0158g00310  -  NBS-LRR type disease resistance protein
## VIT_18s0086g00180  -  MATE efflux family protein
```

```
## VIT_00s0211g00040  -  Nodulation receptor kinase
## VIT_12s0121g00290  -  Diacylglycerol kinase
## VIT_10s0071g00850  -  Binding
## VIT_06s0004g02140  -  MATE efflux family protein
```

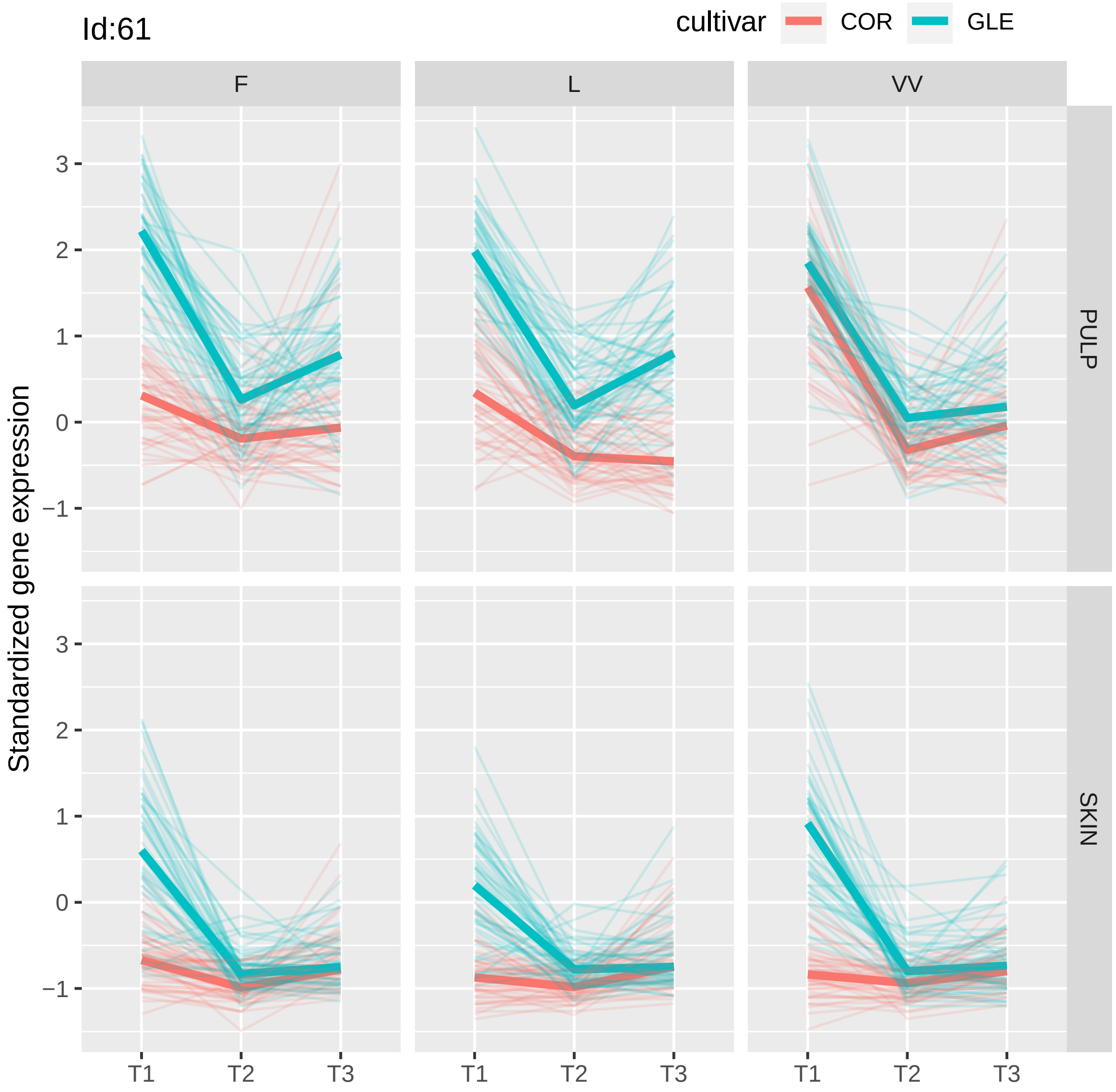

Id:61
cultivar
COR
GLE
F
L
VV
PULP
SKIN
Standardized gene expression
3
2
1
0
−1
T1
T2
T3

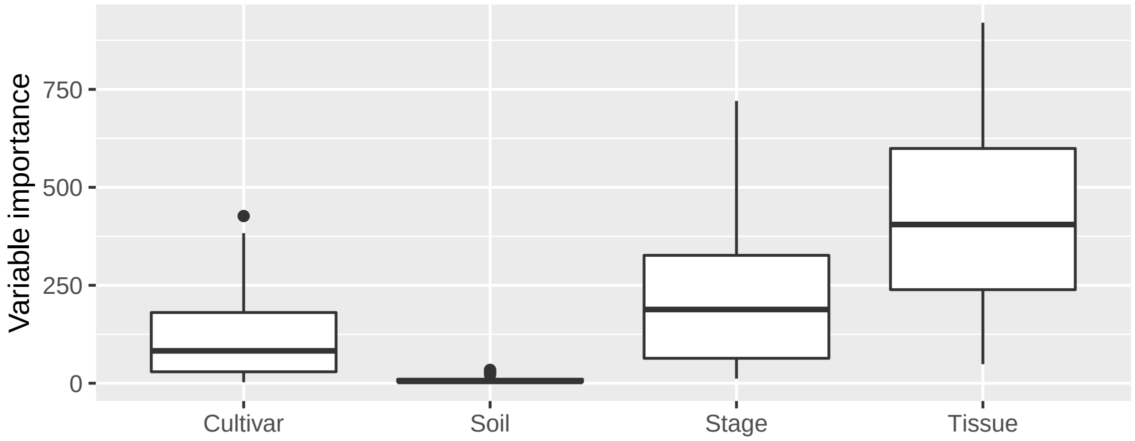

Variable importance
750
500
250
0
Cultivar
Soil
Stage
Tissue

Cluster no. 55

```
## Number of genes in the cluster: 124
## Homogeneity Index:    0.82
## Variable importance for Stage:     Rank =  55  - Median =  161.5
## Variable importance for Cultivar:  Rank =  43  - Median =  56.66
## Variable importance for Tissue:    Rank =  32  - Median =  291.6
## Variable importance for Soil:      Rank =  21  - Median =   7.65
##
## Gene ID                Gene Annotation
## VIT_18s0001g02770  -  Alpha-L-fucosidase 2 precursor
## VIT_07s0031g01920  -  Protein kinase CRK1 CRK1 protein(Cdc2-related kinase 1)
## VIT_13s0106g00280  -  Cytochrome P450, family 79, subfamily A, polypeptide 2
## VIT_06s0009g03120  -  Cytochrome P450, family 79, subfamily A, polypeptide 2
## VIT_02s0025g04290  -  Thaumatin
## VIT_06s0009g03130  -  Cytochrome P450, family 79, subfamily A, polypeptide 2
## VIT_15s0048g02540  -  Pseudo-response regulator 9 (APRR9)
## VIT_13s0047g00200  -  Subtilisin
## VIT_02s0025g00780  -  Cation/hydrogen exchanger (CHX18)
## VIT_02s0025g03310  -  Arsenite transport protein (ArsB)
## VIT_02s0025g04340  -  Osmotin
## VIT_07s0104g00550  -  GTP binding protein
## VIT_18s0089g00180  -  1,4-beta-mannan endohydrolase
## VIT_08s0007g02990  -  Unknown
## VIT_17s0000g07430  -  Unknown
## VIT_13s0064g01470  -  Urophorphyrin III methylase
## VIT_09s0002g05810  -  Boron transporter-like protein 4
## VIT_03s0091g00670  -  Lateral organ boundaries protein 38
## VIT_05s0049g01590  -  Small nuclear ribonucleoprotein D3
## VIT_13s0064g00910  -  Unknown
## VIT_01s0011g06560  -  Potassium transporter (KUP1)
## VIT_18s0122g01480  -  CYP87A2
## VIT_14s0006g00850  -  Valine--tRNA ligase
## VIT_08s0007g07530  -  3-oxoacyl-[acyl-carrier-protein] reductase, chloroplast
## VIT_06s0004g06220  -  Na+/Pi transporter
## VIT_00s2776g00010  -  S-locus lectin protein kinase
## VIT_14s0060g00770  -  Glycosyl transferase family 8 protein
## VIT_14s0108g00930  -  Unknown protein
## VIT_11s0052g00640  -  Nitrogen fixation NifU
## VIT_19s0140g00120  -  Gibberellin 2-beta-dioxygenase 1
## VIT_18s0001g13780  -  Cytochrome P450, family 83, subfamily B, polypeptide 1
## VIT_04s0008g01360  -  Tassel serine threonine kinase 1
## VIT_13s0064g01830  -  R protein disease resistance protein
## VIT_15s0046g03620  -  2-oxo acid dehydrogenase, lipoyl-binding site
## VIT_10s0042g00830  -  Transcription factor jumonji (jmj) protein
## VIT_02s0025g04260  -  Osmotin [Vitis vinifera]
## VIT_02s0025g04230  -  Thaumatin [Vitis vinifera]
## VIT_19s0015g01300  -  Amino acid permease 7
## VIT_09s0002g05570  -  ABC Transporter (VvPDR15 - VvABCG45)
## VIT_01s0146g00350  -  Somatic embryogenesis protein kinase
## VIT_13s0067g03490  -  ARR9 typeA
## VIT_18s0089g00140  -  1,4-beta-mannan endohydrolase
## VIT_14s0128g00110  -  basic helix-loop-helix (bHLH) family
## VIT_02s0025g04250  -  Osmotin
## VIT_02s0236g00070  -  Unknown
## VIT_09s0002g05560  -  ABC Transporter (VvPDR14 - VvABCG44)
```

```
## VIT_06s0004g01010  -  Dirigent protein pDIR9
## VIT_09s0002g05590  -  ABC Transporter (VvPDR16 - VvABCG46)
## VIT_10s0042g00700  -  Leucine-rich repeat receptor-like protein kinase 1
## VIT_08s0007g06060  -  Beta 1-3 glucanase
## VIT_04s0023g01290  -  Anthocyanidin 3-O-glucosyltransferase
## VIT_19s0027g01350  -  R protein MLA10
## VIT_00s0203g00220  -  S-locus lectin protein kinase
## VIT_02s0033g00850  -  Nitrilase
## VIT_02s0033g00700  -  Nitrilase
## VIT_08s0007g06030  -  Beta 1-3 glucanase [Vitis vinifera]
## VIT_13s0156g00470  -  No hit
## VIT_05s0020g03300  -  S-receptor kinase
## VIT_03s0088g00710  -  Pathogenesis-related protein 1 precursor (PRP 1)
## VIT_07s0104g00120  -  Unknown protein
## VIT_08s0007g02810  -  ERF/AP2 Gene Family (VvRAV5)
## VIT_01s0011g06190  -  Histidine kinase (AHK4) (WOL)
## VIT_16s0050g00430  -  Photoassimilate-responsive protein PAR-1a
## VIT_02s0033g00790  -  Nitrilase 4B
## VIT_11s0118g00040  -  Receptor Like Protein 9
## VIT_19s0015g00100  -  CYP71D51v2
## VIT_02s0033g00660  -  Nitrilase 4B
## VIT_19s0015g00130  -  Serine acetyltransferase 3
## VIT_06s0004g06400  -  Flavonoid-O-glucosyltransferase
## VIT_06s0004g06290  -  Binding
## VIT_02s0033g00800  -  Nitrilase 4 (NIT4)
## VIT_11s0016g03400  -  MSS1 (sugar transport protein 13)
## VIT_05s0020g04860  -  Zinc knuckle
## VIT_17s0000g00680  -  No hit
## VIT_05s0020g02830  -  Unknown protein
## VIT_00s0226g00080  -  R protein  PRF  disease resistance protein
## VIT_13s0019g01120  -  R protein  PRF  disease resistance protein
## VIT_19s0027g01170  -  R protein MLA10
## VIT_03s0091g00160  -  NtPRp27 secretory protein
## VIT_09s0002g05370  -  ABC Transporter (VvPDR9 - VvABCG39)
## VIT_11s0052g01780  -  1-deoxy-D-xylulose-5-phosphate synthase
## VIT_16s0050g00400  -  Photoassimilate-responsive protein PAR-1a
## VIT_07s0031g01710  -  WRKY Transcription Factor (VvWRKY22)
## VIT_13s0156g00480  -  R protein disease resistance protein
## VIT_08s0058g00860  -  Unknown protein
## VIT_00s0555g00020  -  flavonoid 3-monooxygenase
## VIT_16s0022g00650  -  No hit
## VIT_05s0094g00350  -  Chitinase class IV
## VIT_02s0025g04270  -  Thaumatin
## VIT_13s0084g00240  -  Steroid sulfotransferase
## VIT_02s0033g01380  -  Alternative oxidase 1D
## VIT_00s0270g00130  -  RKF2 (receptor-like serine/threonine kinase 2)
## VIT_10s0092g00090  -  Ribose-5-phosphate isomerase
## VIT_04s0008g04940  -  Gibberellin 3beta-hydroxylase3
## VIT_08s0007g04860  -  Nodulin family protein
## VIT_15s0046g00740  -  Tetratricopeptide repeat (TPR)-containing
## VIT_03s0063g01150  -  Nodulin 1A, Senescence-associated
## VIT_13s0064g01850  -  R protein MLA10
## VIT_06s0004g06380  -  UDP-glucose: anthocyanidin 5,3-O-glucosyltransferase
## VIT_19s0027g01300  -  Polygalacturonase PGA3
## VIT_10s0003g01940  -  RKF1 (receptor-like kinase in flowers 1)
```

```
## VIT_19s0014g03310  -  No hit
## VIT_11s0016g04260  -  Hydrolase, alpha/beta fold
## VIT_00s0179g00370  -  ESCRT-I complex subunit TSG101
## VIT_17s0000g04030  -  Endonuclease
## VIT_01s0026g00950  -  formin 8
## VIT_19s0027g01330  -  R protein  PRF  disease resistance protein
## VIT_15s0048g01710  -  Alcohol dehydrogenase
## VIT_14s0068g01160  -  Cytokinin-repressed protein CR9
## VIT_12s0035g00610  -  CYP82M1v3
## VIT_13s0019g05010  -  Nodulin-like protein
## VIT_07s0151g01070  -  Ent-copalyl diphosphate synthase (VvTPS68)
## VIT_13s0073g00600  -  Receptor-like kinase 17
## VIT_11s0052g01730  -  1-deoxy-D-xylulose-5-phosphate synthase
## VIT_19s0027g01490  -  R protein MLA10
## VIT_18s0075g00480  -  R protein L6
## VIT_19s0027g01290  -  Exopolygalacturonase
## VIT_05s0077g01680  -  s2_Pathogenesis protein 10 [Vitis vinifera]
## VIT_12s0057g00340  -  Exopolygalacturonase
## VIT_16s0022g00660  -  Trehalose-6-phosphate phosphatase
## VIT_13s0019g05070  -  Nodulin family protein
## VIT_02s0012g01030  -  AL-activated malate transporter 1
## VIT_08s0040g02170  -  MLO6 (mildew resistance locus O 6)
## VIT_12s0035g00140  -  Leucine Rich Repeat receptor-like kinase
```

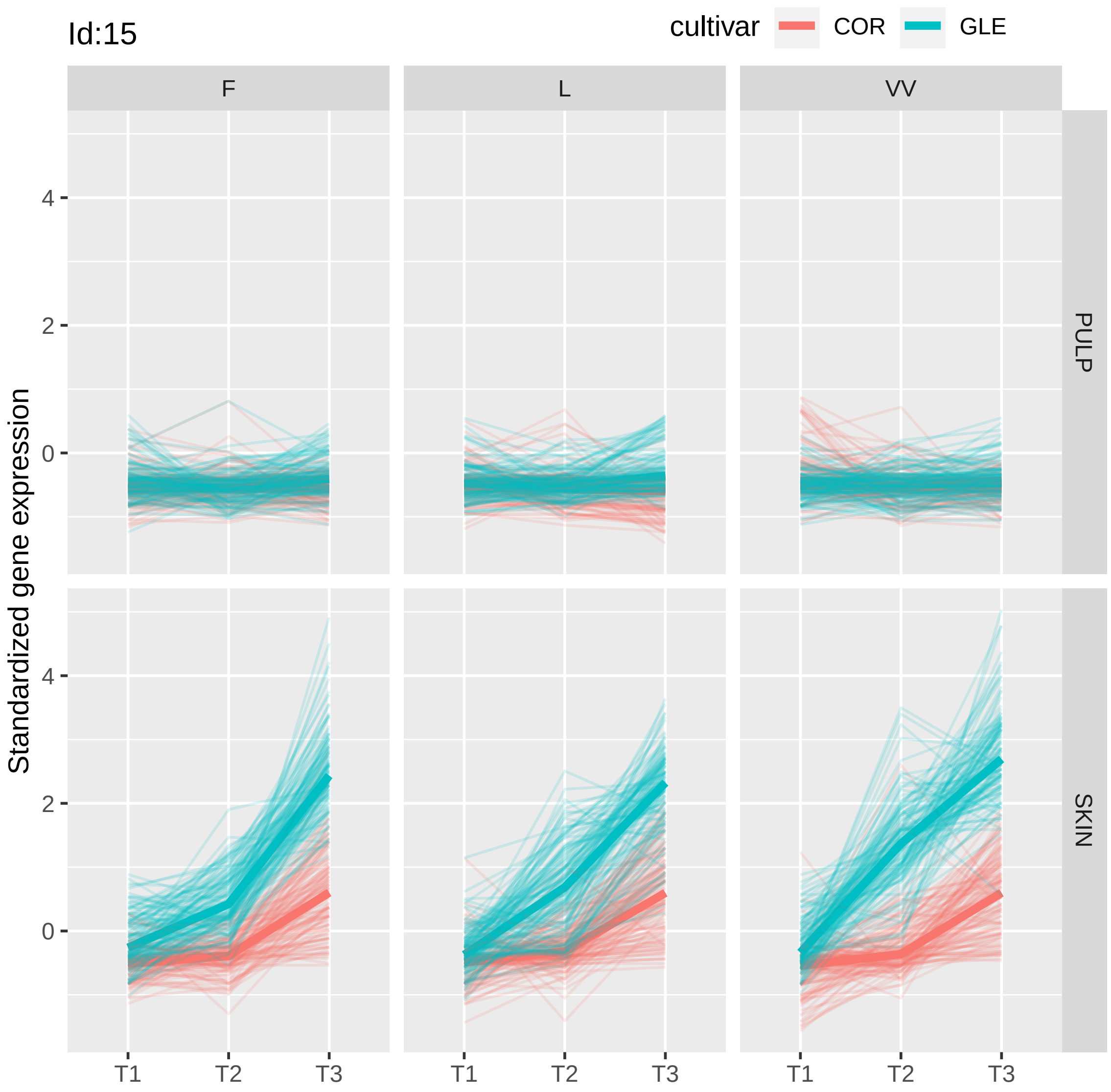
Id:15
cultivar
COR
GLE
F
L
VV
PULP
SKIN
Standardized gene expression
4
2
0
T1
T2
T3

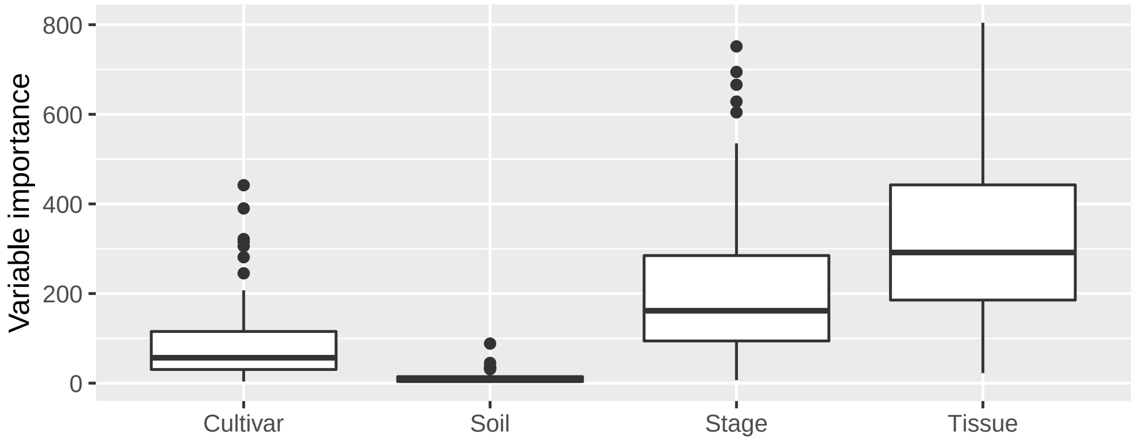
Variable importance
800
600
400
200
0
Cultivar
Soil
Stage
Tissue

Cluster no. 56

```
## Number of genes in the cluster: 72
## Homogeneity Index:    0.75
## Variable importance for Stage:     Rank =  56  - Median =  152.8
## Variable importance for Cultivar:  Rank =  58  - Median =  27.06
## Variable importance for Tissue:    Rank =  25  - Median =  453.9
## Variable importance for Soil:      Rank =  59  - Median =   5.15
##
## Gene ID                Gene Annotation
## VIT_10s0003g00090  -  DRM1 dormancy/auxin associated
## VIT_14s0083g00640  -  Constans 2 (COL2)
## VIT_00s0515g00020  -  R protein MLA10
## VIT_14s0068g01970  -  Unknown protein
## VIT_10s0003g00420  -  Auxin response factor 3
## VIT_12s0028g01030  -  Unknown
## VIT_08s0032g01180  -  CP33 (pigment defective 322)
## VIT_04s0008g02180  -  BRCT domain-containing protein
## VIT_09s0002g02490  -  Dof zinc finger protein 1
## VIT_14s0060g00230  -  Pectinacetylesterase
## VIT_17s0000g01110  -  ATFP3 (Arabidopsis thaliana farnesylated protein 3
## VIT_10s0003g00130  -  ERF Domain protein 12
## VIT_18s0041g00690  -  basic helix-loop-helix (bHLH) family
## VIT_16s0098g01170  -  Homeobox-leucine zipper protein HB-12 (VvATHB-10)
## VIT_13s0019g04990  -  Nodulin-like protein
## VIT_13s0067g03730  -  Unknown
## VIT_02s0025g00910  -  Multidrug resistance-associated protein 14
## VIT_15s0046g00280  -  Unknown
## VIT_04s0044g01670  -  Ankyrin
## VIT_17s0000g01970  -  MKK9
## VIT_14s0068g01690  -  TCP family transcription factor TCP20
## VIT_01s0011g05260  -  DELLA protein GAI1
## VIT_00s0256g00030  -  Ankyrin repeat protein
## VIT_10s0116g01710  -  Galactosyltransferase family protein
## VIT_10s0003g04100  -  Auxin response factor 3
## VIT_13s0019g03100  -  Cis-zeatin O-beta-D-glucosyltransferase
## VIT_00s1682g00020  -  flavonoid 3'-hydroxylase cytochrome P450
## VIT_14s0219g00220  -  CCT motif constans-like
## VIT_14s0068g00130  -  feronia receptor-like kinase
## VIT_00s0256g00050  -  Unknown
## VIT_13s0074g00400  -  PTL (PETAL LOSS)
## VIT_18s0001g03240  -  ERF/AP2 Gene Family (VvERF008),Dehydration Responsive Element-Binding
## ENSRNA049996313  -
## VIT_01s0011g03520  -  Constans-like 16
## VIT_08s0007g03840  -  Unknown protein
## VIT_07s0141g00640  -  ProT1 (PROLINE transporter 1)
## VIT_03s0063g01910  -  Integral membrane protein
## VIT_10s0092g00640  -  Unknown protein
## VIT_05s0051g00130  -  Glutathione S-transferase 25 GSTU7
## VIT_06s0004g07040  -  Glutaredoxin
## VIT_00s1352g00010  -  Myb domain protein 84
## VIT_08s0007g00890  -  Tropinone reductase
## VIT_04s0008g06410  -  No hit
## VIT_15s0046g03660  -  R protein  PRF  disease resistance protein
## VIT_09s0002g03780  -  Integral membrane family protein UPF0497
## VIT_01s0011g02420  -  Unknown
```

```
## VIT_16s0013g01920  -  Ser/Thr protein kinase
## VIT_15s0021g00960  -  1-aminocyclopropane-1-carboxylate oxidase
## VIT_14s0108g01000  -  Calcium-binding EF hand
## VIT_18s0001g06350  -  VQ motif-containing protein
## VIT_00s0541g00010  -  Unknown
## VIT_06s0004g04590  -  Epsin N-terminal homology (ENTH) domain-containing
## VIT_12s0028g01370  -  PMR5 (powdery mildew resistant 5)
## VIT_08s0007g01180  -  S-receptor kinase
## VIT_01s0010g02740  -  4-coumarate-CoA ligase
## VIT_14s0060g01050  -  Pentatricopeptide (PPR) repeat-containing protein
## VIT_17s0000g03560  -  (myb domain protein 62
## VIT_06s0080g00910  -  F-box family protein
## VIT_05s0077g01860  -  ERF/AP2 Gene Family (VvERF058)
## VIT_18s0001g09770  -  S-locus receptor protein kinase
## VIT_05s0029g01430  -  Ankyrin
## VIT_16s0098g01870  -  SOS3 (salt overly sensitive 3)
## VIT_09s0002g00570  -  Lipase GDSL 1
## VIT_02s0012g00450  -  1-aminocyclopropane-1-carboxylate oxidase
## VIT_04s0008g02070  -  Avr9/Cf-9 induced kinase 1
## VIT_08s0007g02280  -  Phospholipase C
## VIT_05s0049g02310  -  Biopterin transport-related protein BT1
## VIT_04s0008g02920  -  Desiccation protein PCC13-62 precursor
## VIT_18s0001g03670  -  Zinc finger (C2H2 type) family
## VIT_08s0007g02850  -  F-box family protein
## VIT_00s0505g00060  -  Photosystem II protein N
## VIT_12s0057g01430  -  Heavy-metal-associated domain-containing protein
```

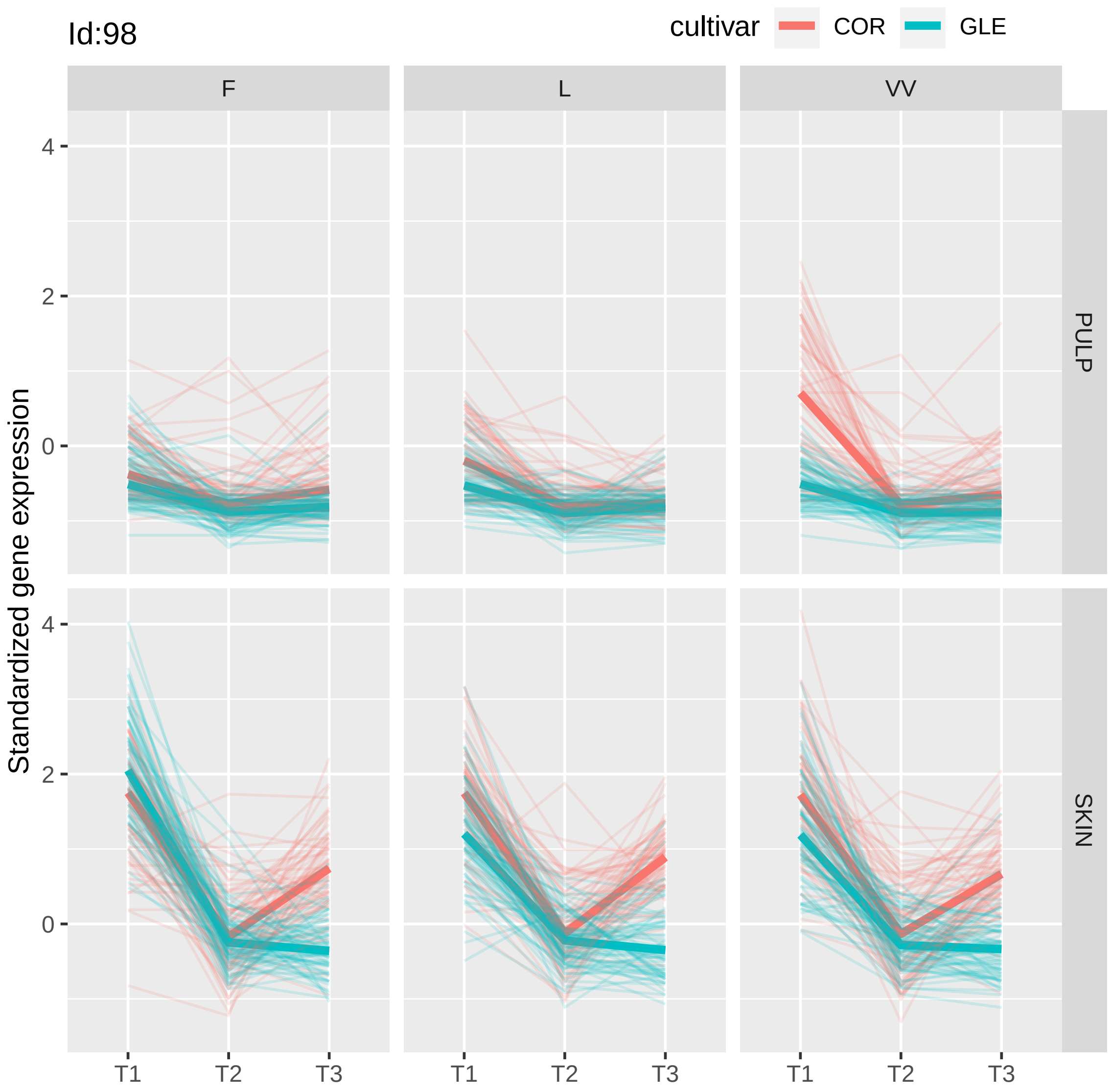
Id:98
cultivar
COR
GLE
F
L
VV
PULP
SKIN
Standardized gene expression
4
2
0
T1
T2
T3

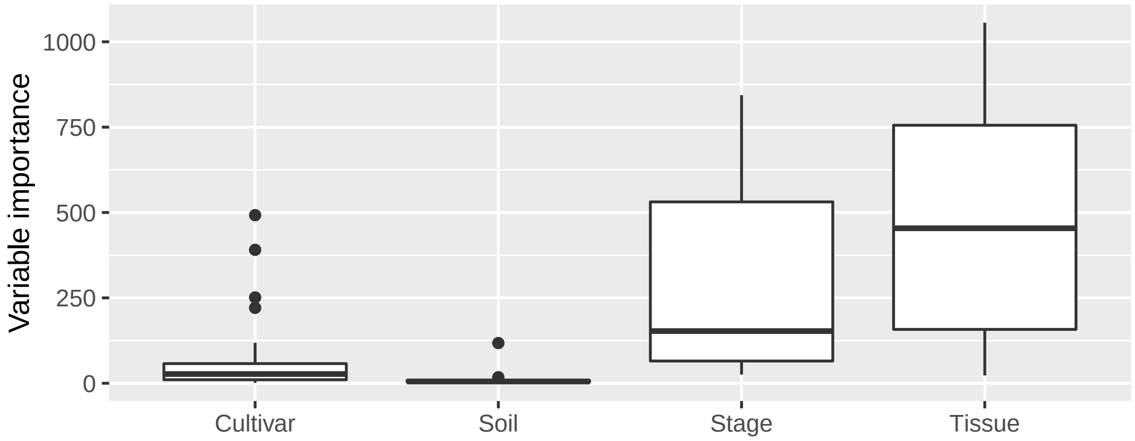
Variable importance
1000
750
500
250
0
Cultivar
Soil
Stage
Tissue

Cluster no. 57

```
## Number of genes in the cluster: 76
## Homogeneity Index:     0.8
## Variable importance for Stage:     Rank =  57  - Median =  151.7
## Variable importance for Cultivar:  Rank =  19  - Median =  205.5
## Variable importance for Tissue:    Rank =  57  - Median =  94.44
## Variable importance for Soil:      Rank =  47  - Median =   5.72
##
## Gene ID                 Gene Annotation
## VIT_05s0094g01570  -  MLO1
## VIT_02s0012g00140  -  Novel plant snare 11
## VIT_11s0016g00690  -  Senescence-associated protein
## VIT_01s0011g04820  -  Molecular chaperone DnaJ
## VIT_05s0020g00730  -  Receptor-like protein kinase
## VIT_08s0040g01690  -  T-complex protein 1 epsilon subunit
## VIT_11s0016g01740  -  Zinc finger (C2H2 type) family
## VIT_13s0019g03650  -  RARE-cold-inducible 2A
## VIT_07s0185g00080  -  Unknown protein
## VIT_09s0002g01000  -  Ferredoxin 4Fe-4S, iron-sulfur binding
## VIT_01s0150g00480  -  Protein binding protein
## VIT_18s0001g15250  -  Unknown
## VIT_08s0007g08380  -  Cellulose synthase CESA3
## VIT_03s0091g01060  -  Cyclin delta-2
## VIT_07s0104g00660  -  2OG-Fe(II) oxygenase
## VIT_00s0769g00010  -  FK506-binding protein genes family (VvFKBP62)
## VIT_18s0075g01040  -  Zinc finger (C2H2 type) family
## VIT_13s0067g00500  -  Transcription elongation factor SPT6
## VIT_07s0005g02950  -  AN3 (ANGUSITFOLIA3)
## VIT_08s0032g00670  -  Glutamate carboxypeptidase (AMP1)
## VIT_18s0001g14440  -  Molecular chaperone DnaJ
## VIT_17s0000g08960  -  Raffinose synthase
## VIT_08s0007g07990  -  No hit
## VIT_14s0068g00050  -  No hit
## VIT_09s0002g08010  -  Vacuolar pyrophosphatase [Vitis vinifera]
## VIT_18s0001g14690  -  Protein kinase
## VIT_06s0004g05740  -  IMP dehydrogenase/GMP reductase
## VIT_11s0037g00860  -  Haemolysin-III related
## VIT_01s0026g00810  -  Leaf senescence protein
## VIT_10s0003g01070  -  Unknown protein
## VIT_13s0084g00360  -  R protein MLA10
## VIT_18s0041g01620  -  R protein L6
## VIT_14s0006g01580  -  Unknown protein
## VIT_03s0038g04650  -  No hit
## VIT_12s0028g01670  -  Trehalose-phosphatase
## VIT_11s0118g00350  -  Vacuolar pyrophosphatase
## VIT_14s0036g01020  -  Polyubiquitin (UBQ4)
## VIT_16s0050g01230  -  No hit
## VIT_18s0001g14270  -  Gibberellin-regulated protein 1 (GASA1)
## VIT_05s0020g02290  -  Endonuclease
## VIT_11s0016g01800  -  Protein kinase MK6
## VIT_11s0016g05780  -  No hit
## VIT_10s0116g01380  -  Amino acid permease 2
## VIT_12s0059g01050  -  No hit
## VIT_18s0001g14260  -  No hit
## VIT_15s0048g02260  -  Calcium-binding EF hand
```

```
## VIT_00s0203g00130  -  Equilibrative nucleoside transporter ENT3
## VIT_05s0020g00440  -  Boron transporter-like protein 1
## VIT_03s0017g00920  -  R protein  PRF  disease resistance protein
## VIT_01s0026g00710  -  Cyclin delta-2
## VIT_19s0014g03600  -  No hit
## VIT_00s0252g00110  -  Carbonic anhydrase
## VIT_15s0046g01850  -  Syntaxin of plants SYP7
## VIT_17s0000g01290  -  RGP1 (reversibly glycosylated polypeptide 1)
## VIT_01s0026g01460  -  Thioredoxin H-type 2 (Trx-H-2)
## VIT_16s0013g00630  -  Cyclin-U4-1 (CycU4;1) CYCP4;1
## VIT_09s0002g00320  -  Pectinesterase PME3
## VIT_03s0017g01620  -  Disease resistance protein RGA4
## VIT_16s0100g00590  -  Unknown protein
## VIT_06s0061g00560  -  Exostosin FRA8 (Fragile fiber8)
## VIT_04s0008g04670  -  Blight-associated protein p12 precursor
## VIT_06s0004g04390  -  UDP-glucuronic acid decarboxylase 1
## VIT_05s0049g00660  -  No hit
## VIT_17s0000g02390  -  Leucine Rich Repeat receptor-like kinase
## VIT_18s0001g05690  -  Protein phosphatase 2C
## VIT_07s0031g02710  -  No hit
## VIT_03s0038g03860  -  Phosphate-induced protein 1
## VIT_05s0029g00170  -  Small ubiquitin modifier 2 (SUMO)
## VIT_04s0210g00060  -  Mucin-like protein
## VIT_00s0238g00090  -  TIR-NBS-LRR disease resistance
## VIT_18s0089g00550  -  TIR-NBS-TIR type disease resistance protein
## VIT_06s0009g01450  -  Salutaridine reductase
## VIT_00s0238g00130  -  RPS4 (resistant to p. syringae 4)
## VIT_18s0089g00520  -  R protein L6
## VIT_12s0035g00340  -  AAA-type ATPase
## VIT_02s0025g00800  -  Cation/hydrogen exchanger (CHX18)
```

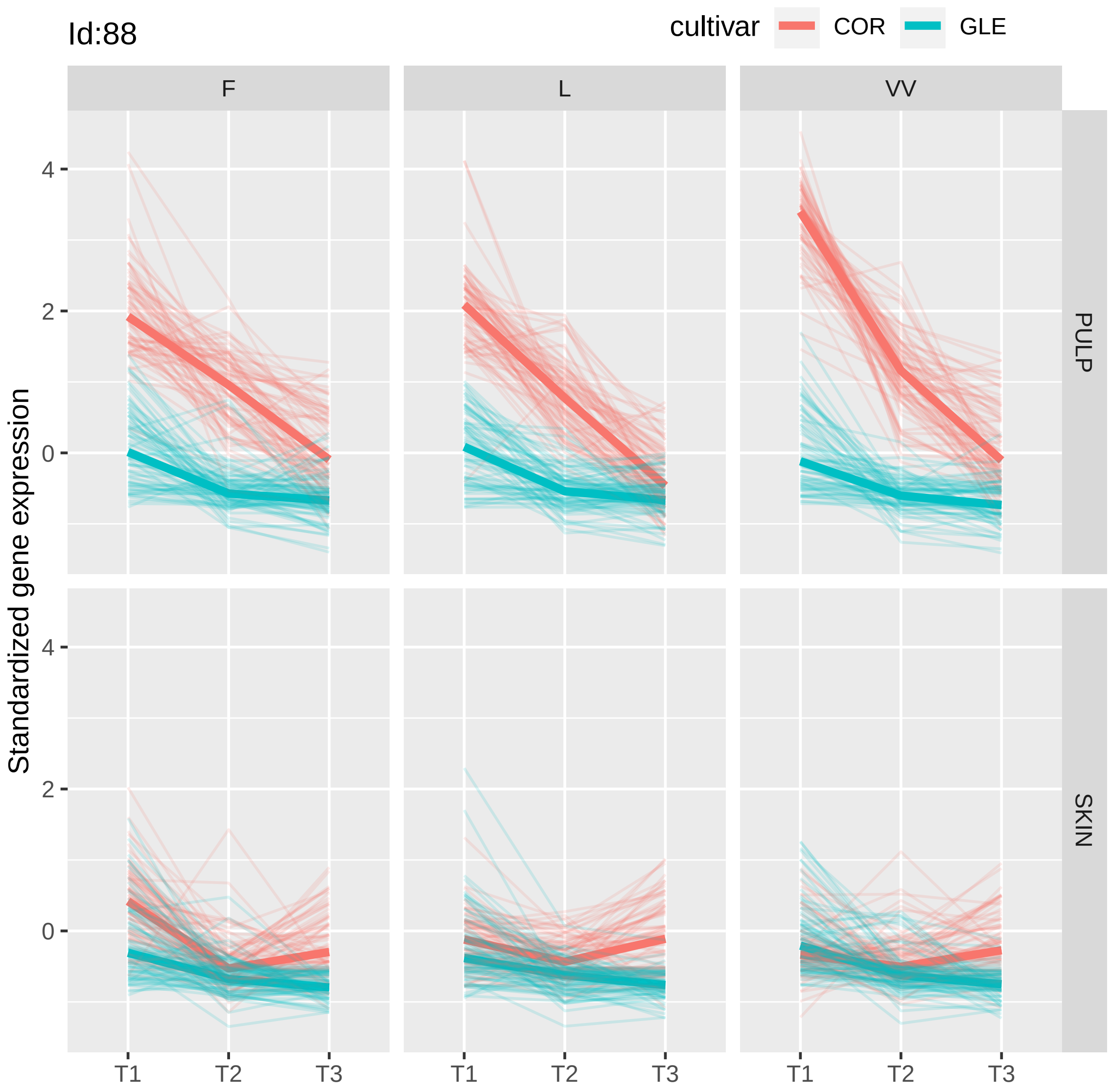

Id:88
cultivar
COR
GLE
F
L
VV
PULP
SKIN
Standardized gene expression
4
2
0
T1
T2
T3

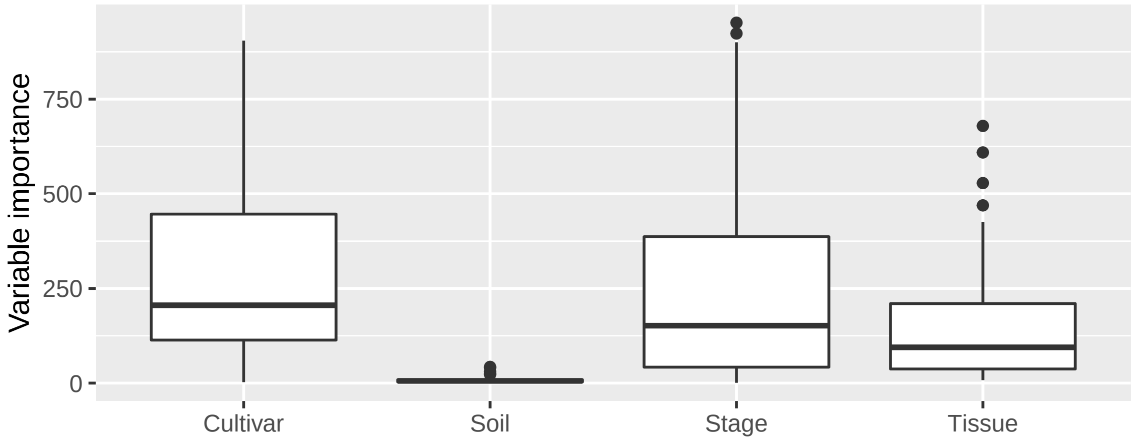

Variable importance
750
500
250
0
Cultivar
Soil
Stage
Tissue

Cluster no. 58

```
## Number of genes in the cluster: 90
## Homogeneity Index:    0.85
## Variable importance for Stage:     Rank =  58  - Median =    150
## Variable importance for Cultivar:  Rank =  88  - Median =   9.25
## Variable importance for Tissue:    Rank =  19  - Median =    616
## Variable importance for Soil:      Rank =  66  - Median =   4.99
##
## Gene ID                Gene Annotation
## VIT_01s0026g00820  -  IAA-amino acid hydrolase 1 (ILR1)
## VIT_11s0016g05430  -  Unknown protein
## VIT_16s0100g00930  -  Stilbene synthase (VvSTS23)
## VIT_10s0042g00840  -  Stilbene Synthase (VvSTS1)
## VIT_19s0177g00230  -  Unknown
## VIT_10s0042g00890  -  Stilbene Synthase (VvSTS3)
## VIT_19s0014g00840  -  Unknown protein
## VIT_04s0069g00790  -  Cyclic nucleotide-regulated ion channel (CNGC14)
## VIT_05s0094g00160  -  No hit
## VIT_12s0059g01400  -  Tyrosine specific protein phosphatase
## VIT_10s0042g00600  -  Leucine Rich Repeat receptor-like kinase
## VIT_13s0156g00590  -  S-receptor kinase
## VIT_16s0039g01280  -  Phenylalanin ammonia-lyase [Vitis vinifera]
## VIT_11s0118g00050  -  EIX receptor
## VIT_05s0020g03280  -  Copper amine oxidase
## VIT_00s1490g00010  -  5'-adenylylsulfate reductase (APR1)
## VIT_06s0061g00120  -  Beta-1,3-glucanase [Vitis riparia]
## VIT_18s0089g01230  -  fructokinase-2
## VIT_01s0150g00430  -  Unknown protein
## VIT_10s0116g01650  -  5'-adenylylsulfate reductase (APR1)
## VIT_07s0005g04880  -  Glutathione S-transferase 25 GSTU7
## VIT_19s0014g04780  -  No hit
## VIT_06s0004g02490  -  ABC Transporter (VvWBC20 - VvABCG20)
## VIT_16s0100g00220  -  No hit
## VIT_07s0005g04890  -  Glutathione S-transferase 25 GSTU7
## VIT_02s0025g01230  -  Amino acid permease
## VIT_08s0007g08750  -  Heat shock transcription factor B3
## VIT_11s0118g00080  -  Disease resistance protein
## VIT_08s0007g06040  -  Beta-1,3-glucanase
## VIT_18s0001g00840  -  Syringolide-induced protein 14-1-1
## VIT_18s0001g12070  -  Photosystem II PsbM
## VIT_19s0027g00010  -  Leucine-rich repeat family protein
## VIT_19s0015g01720  -  fructose-bisphosphate aldolase, cytoplasmic isozyme 1
## VIT_18s0001g02100  -  Unknown protein
## VIT_17s0000g03370  -  Calmodulin-binding protein
## VIT_10s0003g04710  -  BES1/BZR1 homolog protein
## VIT_11s0149g00280  -  Chitinase A
## VIT_06s0009g01140  -  Amino acid permease
## VIT_11s0016g02980  -  Unknown protein
## VIT_04s0008g05790  -  No hit
## VIT_05s0049g01170  -  Unknown protein
## VIT_01s0011g03720  -  BEE1 (BR ENHANCED EXPRESSION 1)
## VIT_08s0058g00690  -  WRKY Transcription Factor (VvWRKY24)
## VIT_06s0080g01160  -  Aspartic Protease (VvAP14)
## VIT_17s0000g06850  -  No hit
## VIT_07s0005g00260  -  ABC Transporter (VvMRP21 - VvABCC21)
```

```
## VIT_19s0014g01180  -  Pathogenesis-related
## VIT_07s0095g00670  -  Curculin (mannose-binding) lectin
## VIT_06s0004g01990  -  No hit
## VIT_04s0044g01880  -  Auxin Efflux Carrier
## VIT_12s0028g02530  -  Zinc finger (C3HC4-type ring finger)
## VIT_10s0042g00960  -  DNAJ heat shock N-terminal domain-containing protein
## VIT_12s0035g00010  -  DNA (cytosine-5)-methyltransferase (ATHIM)
## VIT_06s0004g04030  -  Copper chaperone
## VIT_12s0035g00020  -  Leucine Rich Repeat receptor-like kinase
## VIT_01s0146g00060  -  Glucose-6-phosphate 1-dehydrogenase 2, chloroplast precursor
## VIT_05s0049g01020  -  VvMyb15
## VIT_09s0054g00900  -  Chaperone BCS1 mitochondrial
## VIT_16s0050g01650  -  Unknown
## VIT_08s0040g01770  -  Cyclic nucleotide-gated ion channel 15
## VIT_14s0060g00810  -  Galactinol synthase
## VIT_00s2648g00010  -  FAD-dependent pyridine nucleotide-disulphide oxidoreductase
## VIT_15s0021g02070  -  Unknown protein
## VIT_06s0004g02060  -  Aldehyde Dehydrogenase (VvALDH3J1)
## VIT_07s0104g01260  -  flavin-containing monooxygenase
## VIT_11s0016g02450  -  Serine O-acetyltransferase (SAT-52)
## VIT_00s0203g00170  -  myb domain protein 92
## VIT_14s0068g00420  -  Alpha-amylase isozyme C2 precursor
## VIT_05s0062g00730  -  No hit
## VIT_08s0058g00040  -  Verticillium wilt disease resistance protein Ve2
## VIT_09s0002g06980  -  Chloroplast post-illumination chlorophyll fluorescence increase protei
## VIT_01s0026g02630  -  GTP cyclohydrolase I
## VIT_11s0052g00350  -  Receptor kinase TRKe
## VIT_13s0019g04070  -  MLO1
## VIT_16s0050g02760  -  Receptor serine/threonine kinase
## VIT_12s0034g02570  -  Leucine Rich Repeat receptor-like kinase
## VIT_09s0002g04160  -  Thioesterase family
## VIT_03s0017g02110  -  Anthocyanidin 3-O-glucosyltransferase
## VIT_14s0030g01890  -  Unknown
## VIT_00s0374g00030  -  ARK3 (Arabidopsis Receptor Kinase 3)
## VIT_02s0236g00100  -  Unknown
## VIT_18s0001g14920  -  Unknown protein
## VIT_14s0030g00190  -  Unknown protein
## VIT_00s0294g00060  -  Receptor serine/threonine kinase
## VIT_07s0141g00090  -  Fatty acid elongase 1
## VIT_05s0020g00690  -  PPR2
## VIT_19s0093g00060  -  Polygalacturonase PGA3
## VIT_18s0001g01110  -  Monooxygenase (MO2)
## VIT_05s0020g03690  -  GCN5 N-acetyltransferase (GNAT)
## VIT_05s0094g00260  -  Chitinase, class IV [Vitis vinifera]
```

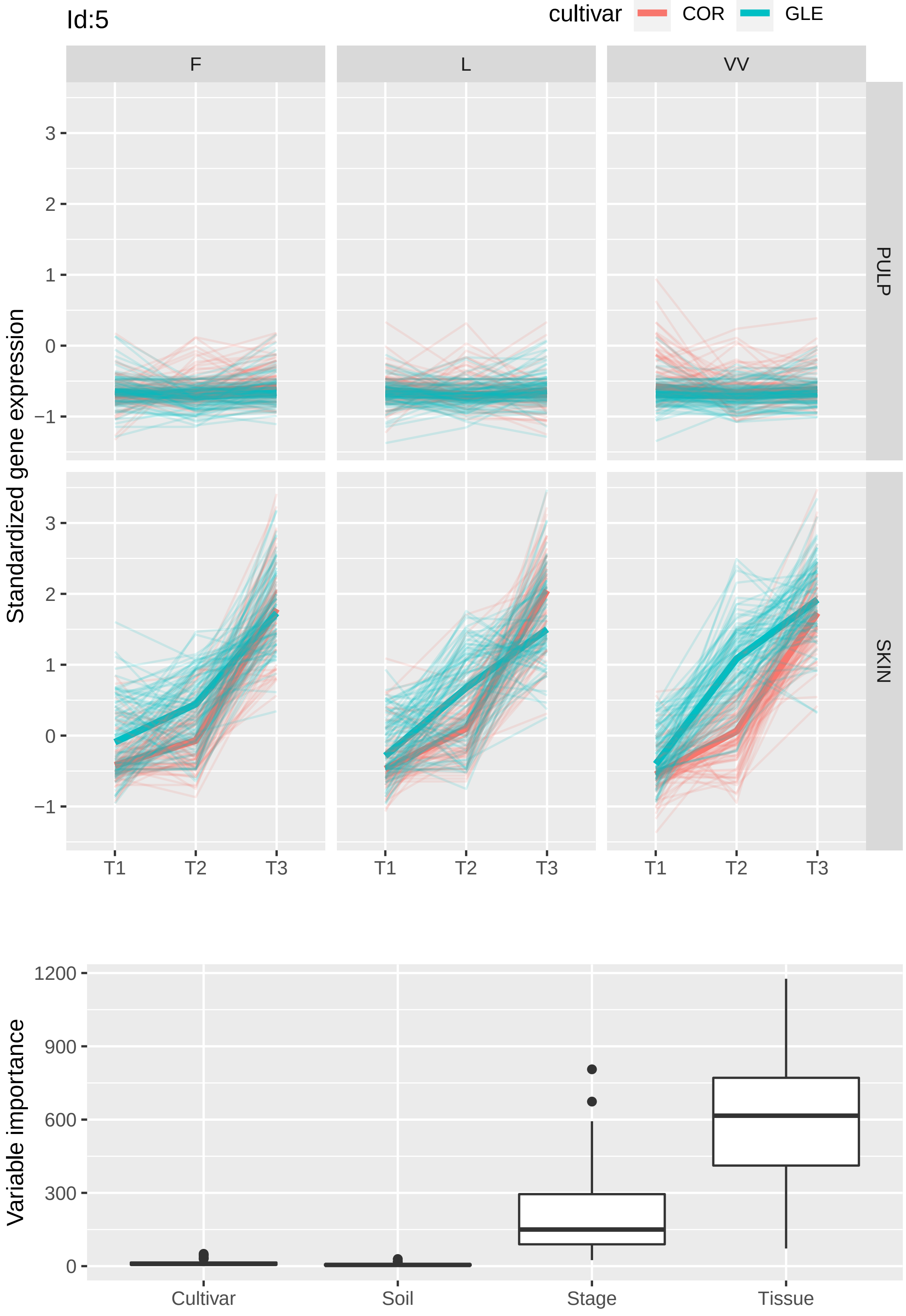
Id:5
cultivar
COR
GLE
F
L
VV
PULP
SKIN
Standardized gene expression
3
2
1
0
−1
T1
T2
T3
Variable importance
1200
900
600
300
0
Cultivar
Soil
Stage
Tissue

Cluster no. 59

```
## Number of genes in the cluster: 119
## Homogeneity Index:    0.82
## Variable importance for Stage:     Rank =  59  - Median =  137.8
## Variable importance for Cultivar:  Rank =  18  - Median =  210.6
## Variable importance for Tissue:    Rank =  44  - Median =  156.8
## Variable importance for Soil:      Rank =  41  - Median =   5.94
##
## Gene ID                Gene Annotation
## VIT_06s0004g07550  -  Wound-induced protein WI12
## VIT_05s0029g00510  -  Aquaporin PIP PIP1A
## VIT_08s0058g00080  -  Verticillium wilt disease resistance protein
## VIT_11s0016g04800  -  No hit
## VIT_09s0002g03410  -  Phytochrome-associated protein 1 (PAP1)
## VIT_18s0001g06150  -  Phosphate-induced protein 1
## VIT_00s0432g00010  -  No hit
## VIT_14s0068g02150  -  Kelch repeat-containing F-box family protein
## ENSRNA049470252  -
## VIT_07s0005g05590  -  Unknown
## VIT_11s0016g04790  -  No hit
## VIT_05s0020g02500  -  Unknown protein
## VIT_18s0157g00210  -  Inositol-1,4,5-trisphosphate 5-Phosphatase
## VIT_01s0011g04400  -  Origin recognition complex subunit 4
## VIT_01s0011g01170  -  No hit
## VIT_08s0007g07440  -  PUMILIO 12 (APUM12)
## VIT_09s0002g06970  -  Palmitoyl-monogalactosyldiacylglycerol delta-7 desaturase, chloroplast
## VIT_18s0001g14140  -  No hit
## VIT_11s0052g01190  -  Xyloglucan endotransglucosylase-hydrolase XTH3
## VIT_08s0007g07940  -  No hit
## VIT_08s0058g00440  -  ferritin
## VIT_14s0083g00880  -  Phosphatidylinositol 4-kinase type-II
## VIT_02s0025g02030  -  No hit
## VIT_07s0031g00660  -  Unknown protein
## VIT_04s0008g01520  -  Heat shock protein 17.6 kDa class II
## VIT_08s0058g00410  -  ferritin 1 (FER1)
## VIT_08s0007g05390  -  ARR3 typeA
## VIT_00s0467g00030  -  R protein MLA10
## VIT_13s0158g00420  -  No hit
## VIT_01s0011g01160  -  Haloacid dehalogenase hydrolase
## VIT_14s0068g01350  -  BTB/POZ domain-containing protein POB1
## VIT_09s0002g05710  -  ATP:ADP antiporter
## VIT_08s0007g00830  -  Lectin protein kinase
## VIT_11s0016g03790  -  No hit
## VIT_08s0058g00400  -  No hit
## VIT_08s0058g00430  -  ferritin
## VIT_18s0001g00950  -  Prolyl 4-hydroxylase alpha-2 subunit precursor
## VIT_18s0001g07310  -  Pleckstrin homology (PH) domain-containing
## VIT_09s0096g00760  -  RPS5 (resistant to p. syringae 5)
## VIT_01s0010g01120  -  Longevity-assurance (LAG1)
## VIT_09s0096g00840  -  NBS-LRR type disease resistance protein
## VIT_11s0052g00570  -  Nodulin MtN21
## VIT_14s0083g00320  -  Cinnamoyl-CoA reductase
## VIT_09s0096g00200  -  RPS5 (resistant to p. syringae 5)
## VIT_11s0037g00450  -  No hit
## VIT_11s0052g01220  -  Xyloglucan endotransglycosylase 6
```

```
## VIT_01s0011g04370  -  Phosphatidylserine synthase 2
## VIT_08s0040g01390  -  Auxin-responsive family protein
## VIT_14s0083g00670  -  No hit
## VIT_08s0007g04770  -  Zinc finger (C2H2 type) family
## VIT_02s0025g04490  -  UDP-galactose transporter 2 ATUTR2/UTR2
## VIT_04s0044g01160  -  Aminotransferase, class V
## VIT_13s0064g01110  -  No hit
## VIT_17s0000g08370  -  RCD one 2 (SRO2)
## VIT_02s0025g00650  -  AAA-type ATPase
## VIT_11s0016g02920  -  Unknown protein
## VIT_14s0083g01140  -  B12D
## VIT_09s0096g00420  -  RPS5 (resistant to p. syringae 5)
## VIT_11s0016g05180  -  Nitrate transporter
## VIT_02s0087g00330  -  Glycosyl transferase family 1 protein
## VIT_09s0002g00190  -  NBS-LRR type disease resistance protein
## VIT_08s0056g01300  -  Cupin, RmlC-type
## VIT_04s0023g01010  -  No hit
## VIT_00s0467g00040  -  No hit
## VIT_04s0008g04920  -  2-oxoglutarate-dependent dioxygenase
## VIT_04s0023g01000  -  PRLI-interacting factor G-like protein
## VIT_03s0110g00170  -  CF4
## VIT_18s0001g08160  -  No hit
## VIT_05s0049g00120  -  Unknown protein
## VIT_15s0045g00250  -  Auxilin
## VIT_03s0063g01960  -  Non-symbiotic hemoglobin class 1
## VIT_01s0011g00820  -  Remorin
## ENSRNA049996376  -
## VIT_08s0007g02360  -  Harpin inducing protein 1-like 9
## VIT_10s0071g00430  -  GRAM domain-containing protein / ABA-responsive
## VIT_00s0285g00030  -  ZFWD1 (zinc finger WD40 repeat protein 1)
## VIT_12s0028g03640  -  Ripening induced protein
## VIT_01s0127g00470  -  Galactinol synthase
## VIT_12s0178g00150  -  Unknown protein
## VIT_07s0005g00820  -  ERF/AP2 Gene Family (VvERF057)
## VIT_13s0158g00320  -  FAD linked oxidase, N-terminal
## VIT_07s0005g01130  -  No hit
## VIT_12s0059g00310  -  Unknown
## VIT_03s0038g02490  -  SKP1
## VIT_14s0068g01800  -  putative MADS-box Flowering Locus C 2 (VviFLC2)
## VIT_03s0038g04580  -  SNF1-Related protein kinase regulatory subunit gamma 1
## VIT_08s0056g00180  -  Translation initiation factor IF-2B subunit delta
## VIT_11s0016g04990  -  Unknown protein
## ENSRNA049467875  -
## VIT_18s0001g13930  -  Auxin response factor 5 (Transcription factor MONOPTEROS)
## VIT_07s0005g00890  -  Polygalacturonase GH28
## VIT_01s0011g02930  -  DNA-binding protein, chloroplast nucleoid
## VIT_14s0060g01870  -  Unknown protein
## VIT_08s0040g01820  -  No hit
## VIT_12s0178g00130  -  Unknown protein
## VIT_19s0015g00890  -  Oligopeptide transporter OPT5
## VIT_00s0163g00030  -  AarF domain containing kinase
## VIT_12s0121g00280  -  Unknown protein
## VIT_04s0023g00910  -  No hit
## VIT_09s0002g07010  -  SNF4
## VIT_08s0105g00400  -  GT-1-like transcription factor
```

```
## VIT_11s0016g02490  -  Ndr family protein
## VIT_16s0098g00360  -  CCT motif constans-like
## VIT_04s0023g01380  -  Scarecrow-like
## VIT_19s0014g05200  -  Unknown protein
## VIT_16s0100g00700  -  TFL1 (Terminal flower 1), VvFLT1C
## VIT_00s1944g00010  -  Pentatricopeptide repeat-containing protein
## VIT_14s0060g01880  -  Unknown protein
## VIT_13s0064g01100  -  No hit
## VIT_06s0004g05590  -  Subtilisin protease C1
## VIT_00s0352g00030  -  No hit
## VIT_11s0016g02800  -  Myo-inositol oxygenase
## VIT_13s0064g01120  -  No hit
## VIT_07s0031g00630  -  Unknown protein
## VIT_18s0122g00360  -  DC1 domain-containing protein
## VIT_18s0001g03690  -  Unknown protein
## VIT_10s0003g03950  -  AERO1 (arabidopsis endoplasmic reticulum oxidoreductins 1)
## VIT_00s0239g00110  -  Mei2 AML1
## VIT_02s0025g01760  -  Cellulose synthase CSLG3
```

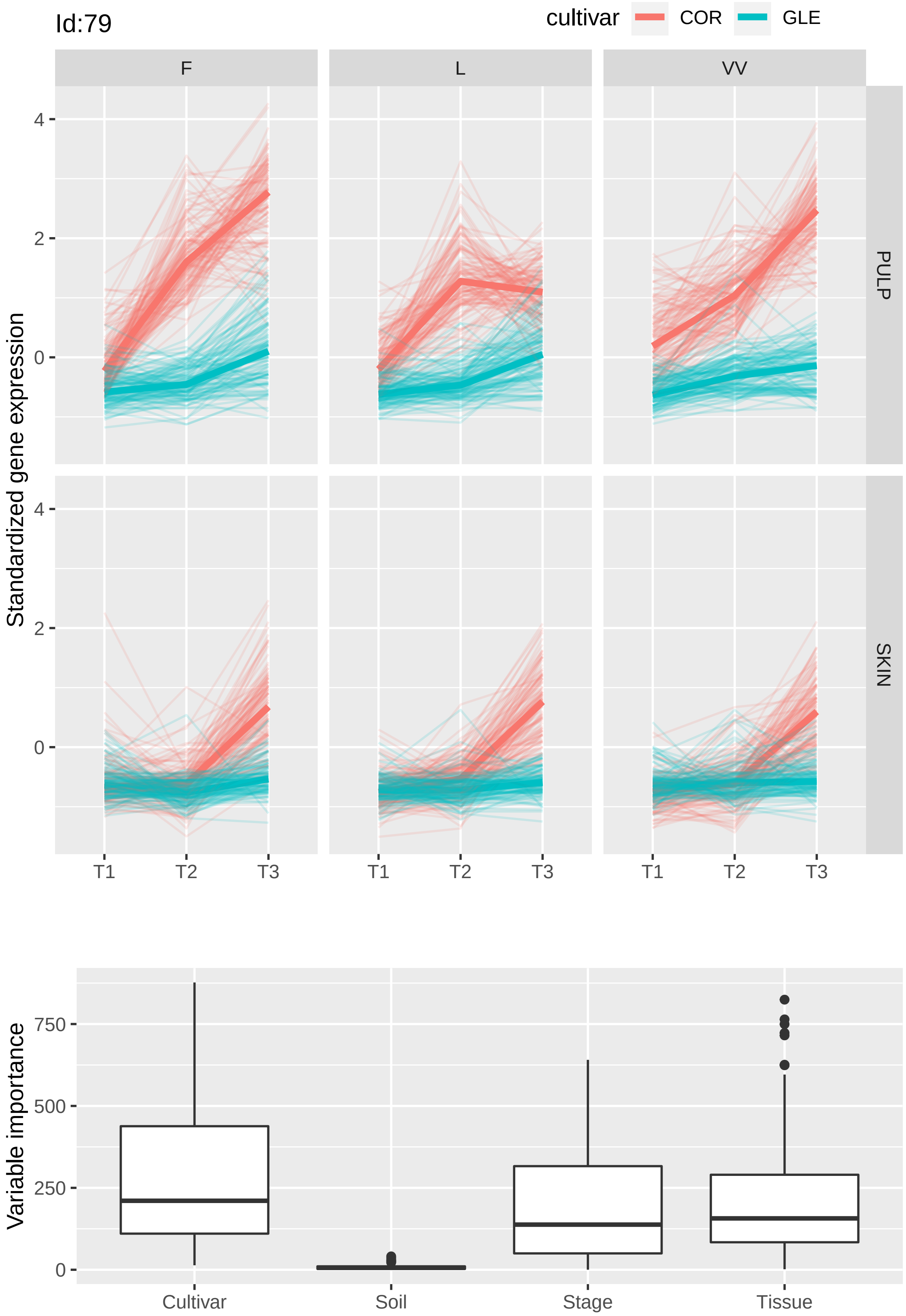

Id:79
cultivar
COR
GLE
F
L
VV
PULP
SKIN
Standardized gene expression
4
2
0
T1
T2
T3
Variable importance
750
500
250
0
Cultivar
Soil
Stage
Tissue

Cluster no. 60

```
## Number of genes in the cluster: 17
## Homogeneity Index:    0.42
## Variable importance for Stage:     Rank =  60  - Median =  135.1
## Variable importance for Cultivar:  Rank =  24  - Median =  172.4
## Variable importance for Tissue:    Rank =  61  - Median =  78.78
## Variable importance for Soil:      Rank =   3  - Median =  24.57
##
## Gene ID               Gene Annotation
## VIT_18s0001g08920  -  No hit
## VIT_11s0016g02910  -  Acid phosphatase class B
## VIT_13s0019g02450  -  BEL1-like homeodomain protein 9 LSN (LARSON)
## VIT_11s0052g00870  -  IAA33
## VIT_18s0001g11760  -  MATE efflux family protein
## VIT_08s0007g03770  -  Unknown protein
## VIT_01s0011g03780  -  Zinc finger protein 6
## VIT_14s0006g02310  -  No hit
## ENSRNA049468071  -
## VIT_06s0004g03400  -  Myosin heavy chain
## VIT_05s0062g00840  -  RAB GTPase ARA4
## VIT_00s0218g00160  -  UDP-rhamnose:rhamnosyltransferase
## VIT_12s0055g01140  -  Lipid transfer protein
## VIT_04s0023g03370  -  flavanone-3-hydroxylase  1 (F3H1) [Vitis vinifera]
## ENSRNA049466798  -
## ENSRNA049467282  -
## VIT_03s0038g03190  -  Flavin containing monooxygenase 3
```

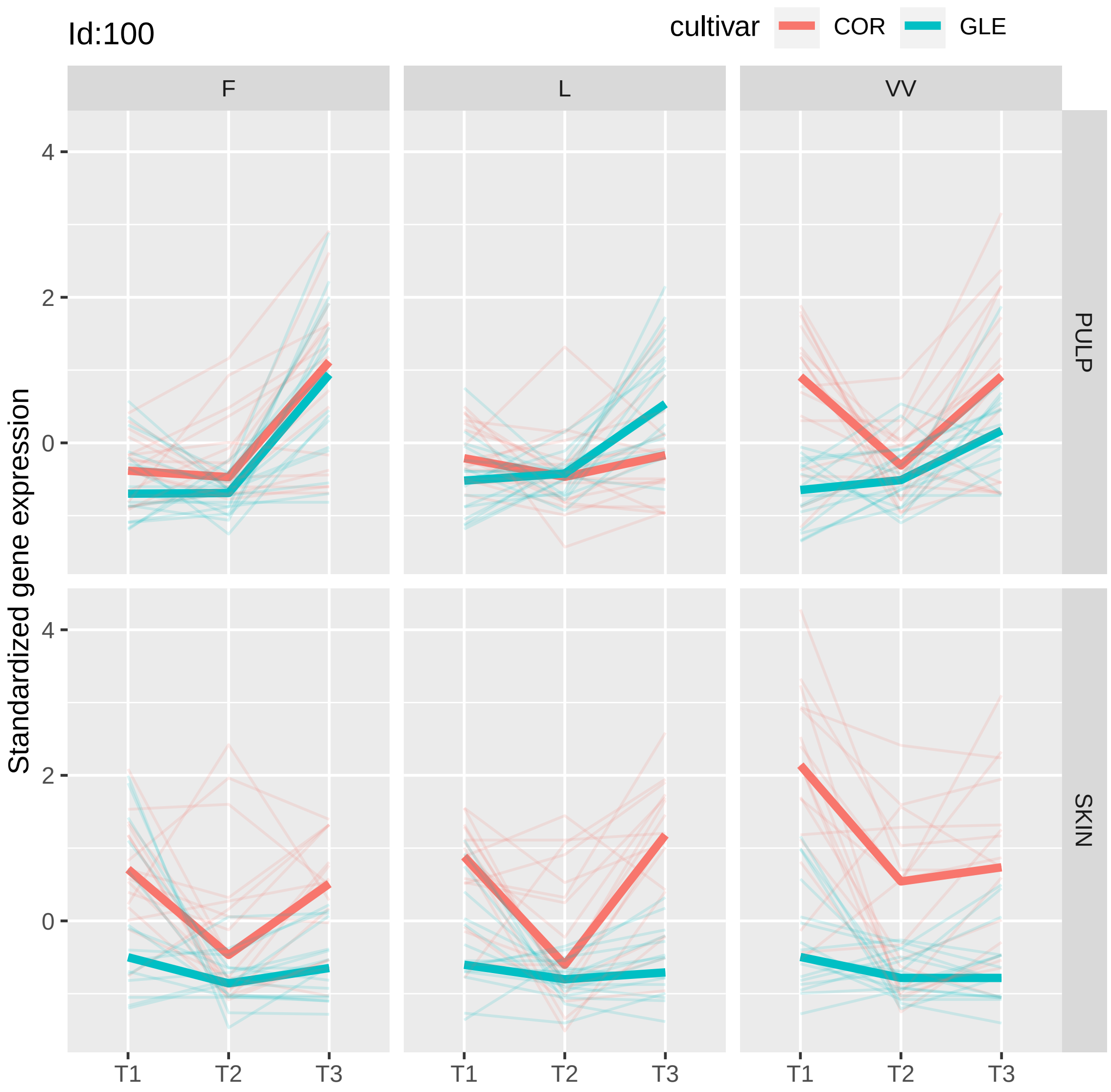

Id:100
cultivar
COR
GLE
F
L
VV
PULP
SKIN
Standardized gene expression
4
2
0
T1
T2
T3

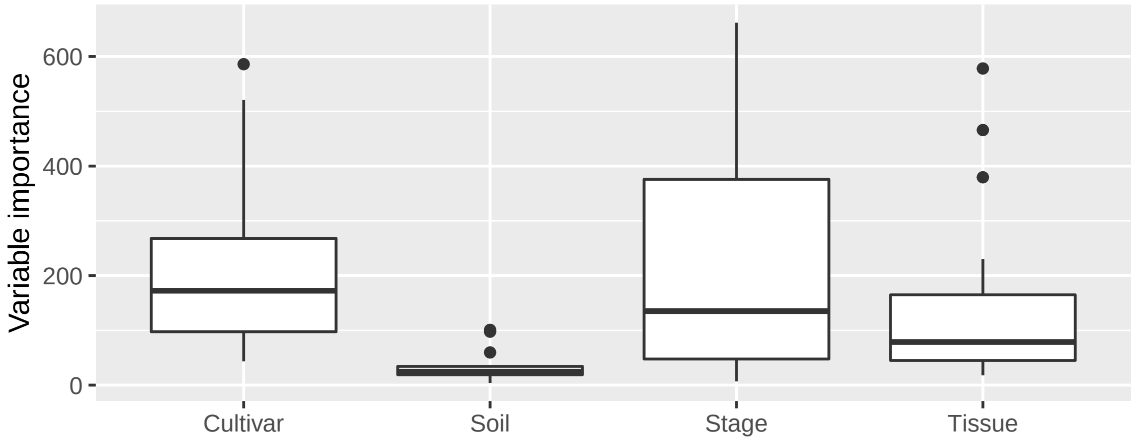

Variable importance
600
400
200
0
Cultivar
Soil
Stage
Tissue

Cluster no. 61

```
## Number of genes in the cluster: 20
## Homogeneity Index:    0.74
## Variable importance for Stage:     Rank =  61  - Median =  123.8
## Variable importance for Cultivar:  Rank =  47  - Median =  49.63
## Variable importance for Tissue:    Rank =  54  - Median =  106.6
## Variable importance for Soil:      Rank =   1  - Median =  38.47
##
## Gene ID               Gene Annotation
## VIT_00s0505g00040  -  Unknown
## VIT_00s0332g00150  -  No hit
## VIT_00s0246g00240  -  Ribosomal protein S1 (Rps1 protein)
## VIT_00s0332g00140  -  NADH dehydrogenase (ubiquinone) Fe-S protein 2
## VIT_09s0002g00310  -  ATP synthase CF0 A subunit
## VIT_00s0275g00030  -  Ycf1
## VIT_00s0396g00050  -  Ribosomal protein S4, Mitochondrial
## VIT_18s0001g01710  -  DEAD box RNA helicase
## VIT_06s0009g02080  -  Chloroplast envelope membrane protein
## VIT_00s0246g00040  -  No hit
## ENSRNA049469952  -
## VIT_07s0031g01060  -  Unknown
## VIT_14s0006g02300  -  DNA-directed RNA polymerase subunit beta'
## VIT_06s0004g05580  -  RNA polymerase beta subunit [Vitis vinifera]
## VIT_09s0070g00650  -  RNA polymerase beta''
## VIT_00s0733g00010  -  F-type H+-transporting ATPase alpha chain
## VIT_09s0002g08340  -  Photosystem I P700 chlorophyll a apoprotein A1
## VIT_13s0067g01850  -  RNA polymerase beta''
## ENSRNA049467717  -
## VIT_19s0027g00800  -  Ycf2
```

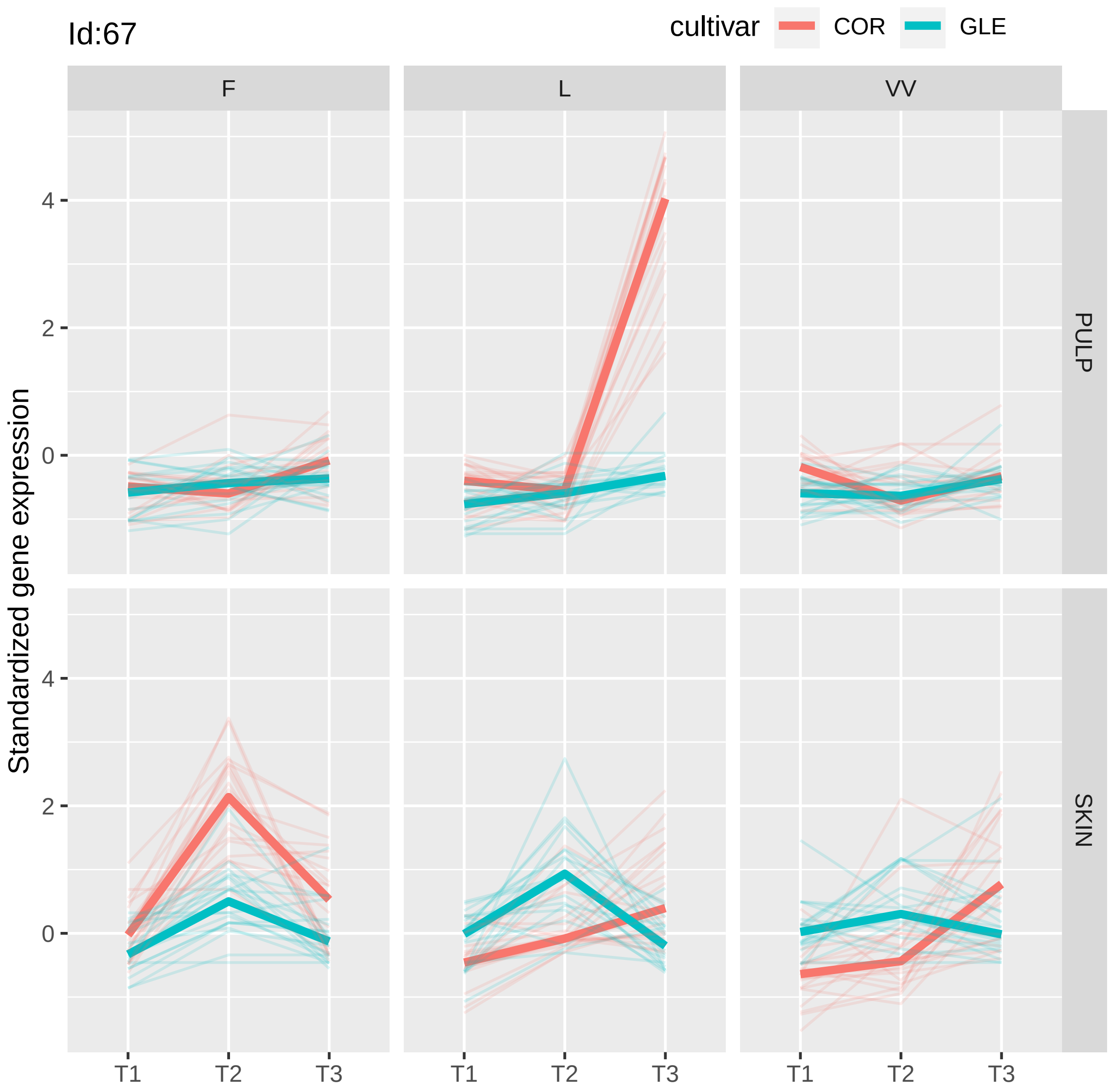
Id:67
cultivar
COR
GLE
F
L
VV
PULP
SKIN
Standardized gene expression
4
2
0
4
2
0
T1
T2
T3
T1
T2
T3
T1
T2
T3

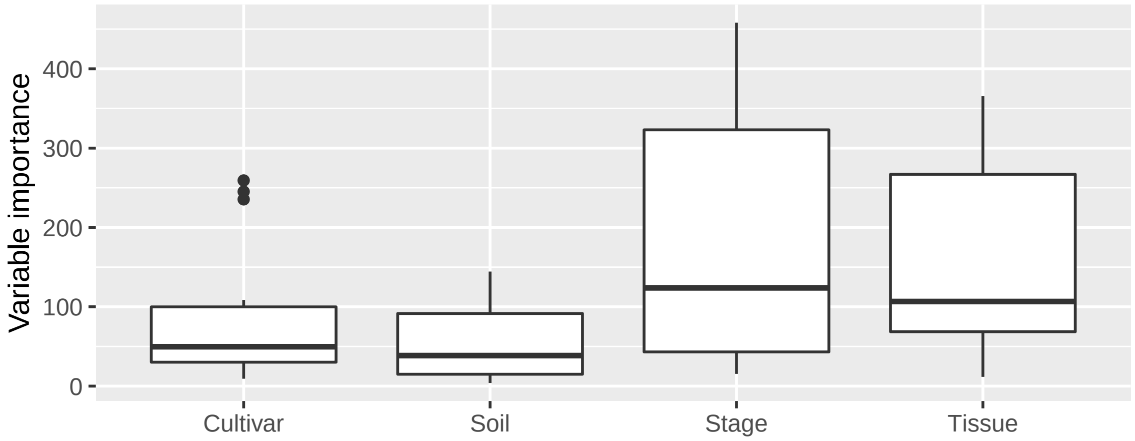
Variable importance
400
300
200
100
0
Cultivar
Soil
Stage
Tissue

Cluster no. 62

```
## Number of genes in the cluster: 76
## Homogeneity Index:    0.77
## Variable importance for Stage:     Rank =  62  - Median =  120.2
## Variable importance for Cultivar:  Rank =   9  - Median =  602.6
## Variable importance for Tissue:    Rank =  92  - Median =  13.49
## Variable importance for Soil:      Rank =  36  - Median =   6.13
##
## Gene ID                Gene Annotation
## VIT_03s0017g01710  -  No hit
## VIT_05s0049g00950  -  Phosphoenolpyruvate carboxylase kinase
## VIT_18s0001g05160  -  Glycosyl hydrolase family 3 protein
## VIT_12s0059g02340  -  Syntaxin 1B/2/3/4
## VIT_14s0006g02720  -  Hexose transporter, Plastid
## VIT_14s0068g01380  -  Constans-like 14
## VIT_18s0157g00190  -  Choline kinase
## VIT_13s0019g03550  -  ERF/AP2 Gene Family (VvAP2-11)
## VIT_08s0007g04600  -  UGT73C2 (UDP-glucosyl transferase 73C2)
## VIT_13s0067g00760  -  R protein  PRF  disease resistance protein
## VIT_00s0487g00030  -  R protein MLA10
## VIT_10s0003g01240  -  14-3-3 protein GF14 iota (GRF12)
## VIT_19s0090g00330  -  1-acyl-sn-glycerol-3-phosphate acyltransferase
## VIT_00s0357g00010  -  Senescence-associated protein
## VIT_03s0017g00830  -  2OG-Fe(II) oxygenase
## VIT_03s0017g00030  -  Ankyrin repeat protein
## VIT_15s0046g00450  -  Phosphatidic acid Phosphatase
## VIT_01s0150g00460  -  Xyloglucan endotransglucosylase/hydrolase precursor
## VIT_08s0007g05860  -  GASA like
## VIT_12s0055g00070  -  UDP-glycosyltransferase 71A13
## VIT_12s0055g00280  -  UDP-glycosyltransferase 71A13
## VIT_09s0002g03450  -  Taurine dioxygenase
## VIT_05s0020g00840  -  No hit
## VIT_04s0008g01640  -  Ankyrin repeat
## VIT_05s0094g01130  -  Allyl alcohol dehydrogenase
## VIT_12s0059g00570  -  fasciclin arabinogalactan-protein (FLA7)
## VIT_02s0025g02540  -  Unknown protein
## VIT_09s0002g06400  -  No hit
## VIT_03s0017g01460  -  No hit
## VIT_14s0171g00360  -  Unknown protein
## VIT_03s0097g00680  -  Zinc finger (C3HC4-type ring finger)
## VIT_18s0157g00090  -  Alliin lyase precursor
## VIT_03s0180g00200  -  Limonoid UDP-glucosyltransferase (VvGT2)
## VIT_18s0041g00170  -  Brassinosteroid insensitive 1-associated receptor kinase 1
## VIT_18s0001g01940  -  Ripening regulated protein DDTFR8
## VIT_12s0035g01680  -  CC-NBS-LRR class
## VIT_19s0014g04040  -  S-receptor protein kinase
## VIT_00s0540g00020  -  Chitinase, class V
## VIT_15s0046g03150  -  DTA2 (downstream target of AGL15 2)
## VIT_14s0060g00790  -  Galactinol synthase
## VIT_02s0025g01360  -  ERF/AP2 Gene Family (VvERF002),Dehydration Responsive Element-Binding
## VIT_03s0180g00320  -  indole-3-acetate beta-glucosyltransferase (VvGT3)
## VIT_04s0023g00470  -  WRKY DNA-binding protein 2 (WRKY-4), WRKY Transcription Factor (VvWRKY
## VIT_12s0055g00200  -  UDP-glucose glucosyltransferase
## VIT_16s0022g01900  -  Pentatricopeptide (PPR) repeat
## VIT_07s0005g06410  -  High-affinity K+ transporter 1 (HKT1)
```

```
## VIT_05s0094g01350  -  Thiol methyltransferase 1
## VIT_13s0073g00180  -  Rieske [2Fe-2S] domain
## VIT_03s0038g00250  -  Purple acid phosphatase 10 ATPAP10/PAP10
## VIT_16s0050g01910  -  Unknown
## VIT_07s0005g01950  -  myb domain protein 78
## VIT_00s0912g00010  -  Unknown protein
## VIT_04s0023g02300  -  No hit
## VIT_00s1281g00010  -  Membrane bound O-acyl transferase (MBOAT)
## VIT_07s0104g00180  -  Nectarin IV NEC4
## VIT_00s0227g00180  -  Acyl-coenzyme A thioesterase 9.
## VIT_17s0000g04300  -  Isopenicillin N synthetase; KH, type 1
## VIT_15s0046g03290  -  Unknown protein
## VIT_00s2795g00010  -  Glyoxylate reductase
## VIT_02s0154g00170  -  flavin-containing monooxygenase 3
## VIT_07s0151g00510  -  GCN5 N-acetyltransferase (GNAT)
## VIT_02s0154g00200  -  Unknown protein
## VIT_03s0017g00710  -  flavonol synthase
## VIT_14s0068g00470  -  UDP-glucosyl transferase
## VIT_00s0214g00120  -  F-box family protein
## VIT_05s0077g00550  -  SFR2 (sensitive TO FREEZING 2)
## VIT_19s0015g02610  -  Glutathione S-transferase (VvGST5)
## VIT_14s0068g00480  -  UDP-glucosyl transferase
## VIT_09s0096g00140  -  R protein MLA10
## VIT_00s2698g00010  -  No hit
## VIT_06s0004g07580  -  Aspartyl-tRNA synthetase
## VIT_02s0154g00140  -  3-oxoacyl-[acyl-carrier-protein] synthase 3 A, chloroplast precursor
## VIT_18s0075g00260  -  Molecular chaperone DnaK
## VIT_00s0823g00020  -  GCN5 N-acetyltransferase (GNAT)
## VIT_07s0141g01030  -  R protein MLA10
## VIT_01s0011g02440  -  Metal-dependent phosphohydrolase HD domain-containing protein-like
```

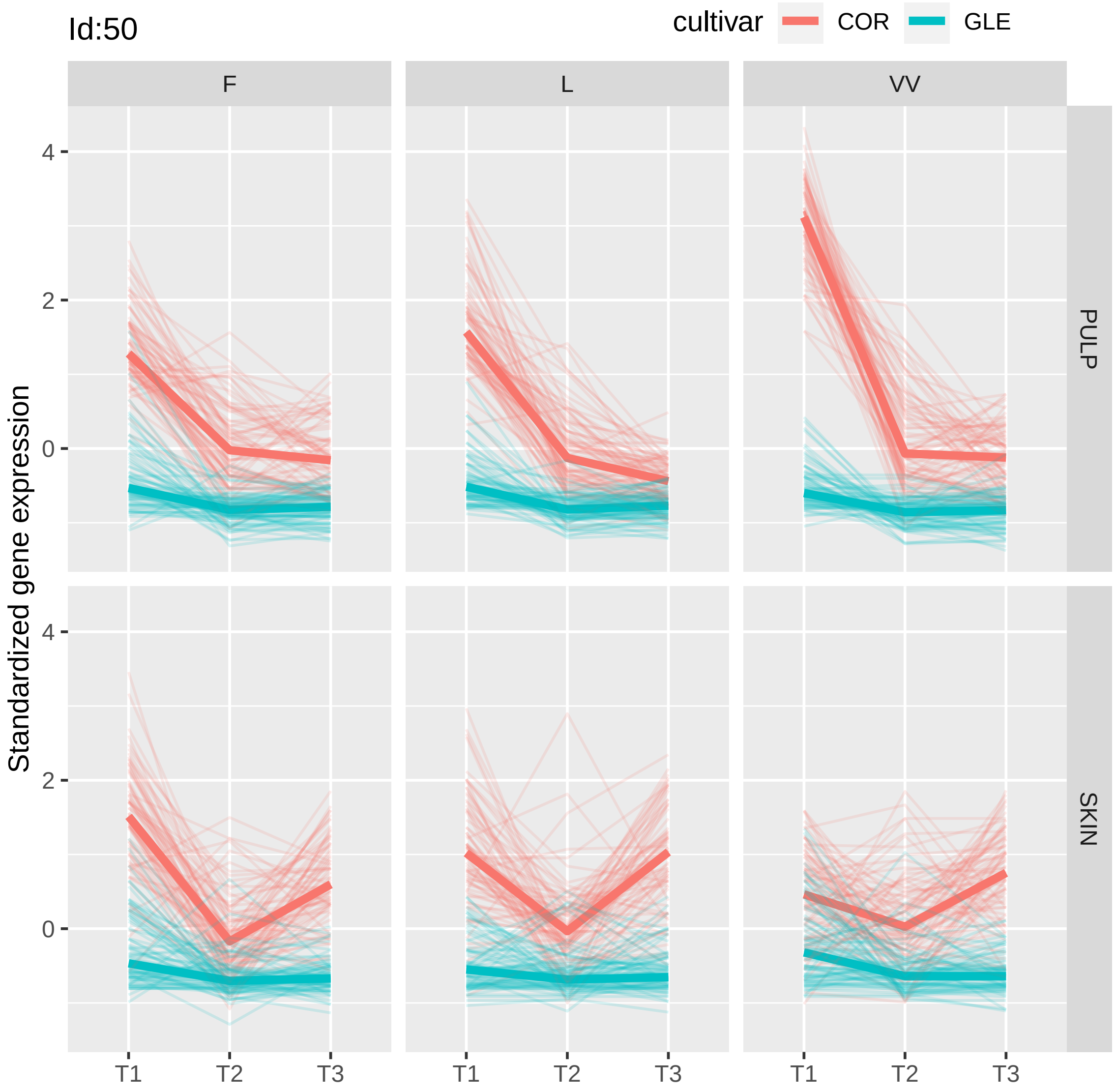
Id:50
cultivar
COR
GLE
F
L
VV
PULP
SKIN
Standardized gene expression
4
2
0
T1
T2
T3

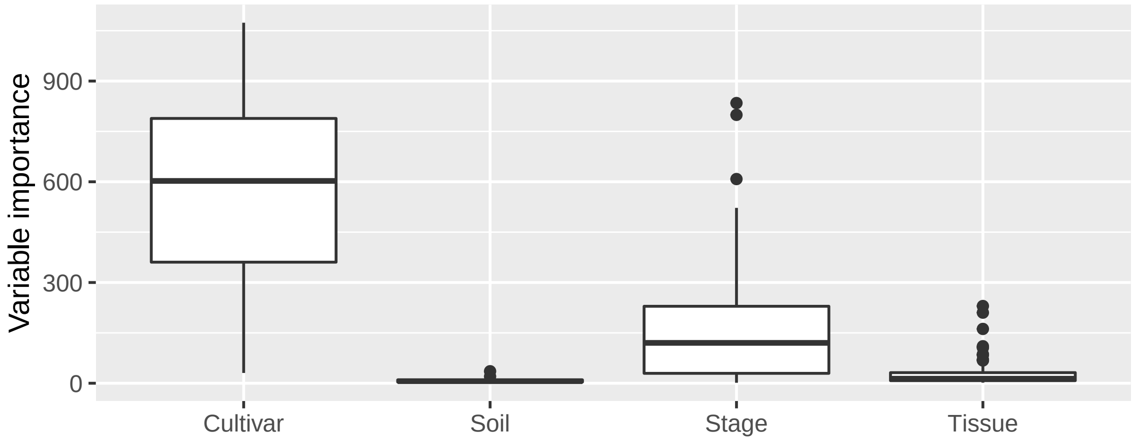
Variable importance
900
600
300
0
Cultivar
Soil
Stage
Tissue

Cluster no. 63

```
## Number of genes in the cluster: 54
## Homogeneity Index:    0.64
## Variable importance for Stage:     Rank =  63  - Median =  103.2
## Variable importance for Cultivar:  Rank =  16  - Median =  295.1
## Variable importance for Tissue:    Rank =  72  - Median =  34.71
## Variable importance for Soil:      Rank =   6  - Median =  17.79
##
## Gene ID                Gene Annotation
## VIT_17s0000g05290  -  Pre-mRNA cleavage complex II protein Clp1
## VIT_02s0087g00640  -  Unknown
## VIT_06s0004g04170  -  Methyl-CpG-binding domain-containing protein
## VIT_01s0137g00430  -  Cellulase
## VIT_12s0059g01640  -  Unknown protein
## VIT_14s0060g01910  -  Nodulin MtN3 family
## VIT_14s0030g00420  -  Inosine-uridine preferring nucleoside hydrolase family protein
## VIT_00s0181g00010  -  Sugar transporter 1 (VvHT8)
## VIT_12s0059g02270  -  Glutamate receptor protein GLR3.4b
## VIT_06s0004g05650  -  Unknown protein
## VIT_11s0016g02580  -  MAPK activating protein-like
## VIT_16s0098g00200  -  Receptor serine/threonine kinase PR5K
## VIT_09s0002g03610  -  MYB divaricata
## VIT_01s0026g01050  -  Myb family
## VIT_09s0002g01590  -  Nuclear transcription factor Y subunit A-8
## VIT_08s0007g03520  -  Purple acid phosphatase 22- ATPAP22/PAP22
## VIT_13s0067g03510  -  ARR9 typeA
## VIT_17s0000g09100  -  Copper amine oxidase
## VIT_02s0025g00210  -  fringe protein
## VIT_08s0007g01840  -  Unknown
## VIT_12s0028g01040  -  Protein kinase
## VIT_18s0001g08600  -  basic helix-loop-helix (bHLH) family
## VIT_08s0007g04380  -  PAB7 (poly(A) binding protein 7)
## VIT_01s0010g01280  -  No hit
## VIT_16s0022g00540  -  Glycerol 3-phosphate permease
## VIT_19s0015g00550  -  Nudix hydrolase 10
## VIT_00s0404g00050  -  fiber protein
## VIT_07s0005g06540  -  CC-NBS-LRR class
## VIT_02s0087g00500  -  MAP kinase 9
## VIT_07s0031g00490  -  MAP3K protein kinase
## VIT_07s0151g00160  -  GCN5 N-acetyltransferase (GNAT)
## VIT_13s0067g01100  -  RPM1 (resistance to p. syringae pv maculicola 1)
## VIT_01s0011g06080  -  Lectin protein kinase family
## VIT_19s0014g04080  -  Serine/threonine-protein kinase receptor ARK3
## VIT_12s0034g01730  -  No hit
## VIT_00s0477g00070  -  Mg2+-importing ATPase (VvPH1)
## ENSRNA049467196  -
## VIT_12s0055g00900  -  RAE1 RNA export 1 homolog
## VIT_01s0026g00220  -  Aldehyde Dehydrogenase (VvALDH2B8)
## VIT_05s0020g05060  -  Cellulose synthase CSLG2
## VIT_00s0227g00100  -  No hit
## VIT_05s0020g01910  -  1,4-alpha-D-glucan maltohydrolase
## VIT_18s0041g01470  -  TIR-NBS-LRR-TIR disease resistance protein
## VIT_18s0001g04660  -  No hit
## VIT_07s0031g02440  -  Amino acid permease 3
## VIT_19s0015g01290  -  Amino acid permease 7
```

```
## VIT_00s0227g00080  -  Disease resistance protein
## VIT_02s0012g00770  -  No hit
## VIT_12s0034g01470  -  RPM1 (resistance to p. syringae pv maculicola 1)
## VIT_08s0007g01360  -  Unknown protein
## VIT_00s1336g00010  -  Unknown
## VIT_15s0048g00330  -  GAE3 (UDP-D- glcucuronate  4-epimerase 3)
## VIT_18s0001g09550  -  Receptor kinase TRKe
## VIT_04s0044g00640  -  S-receptor kinase
```

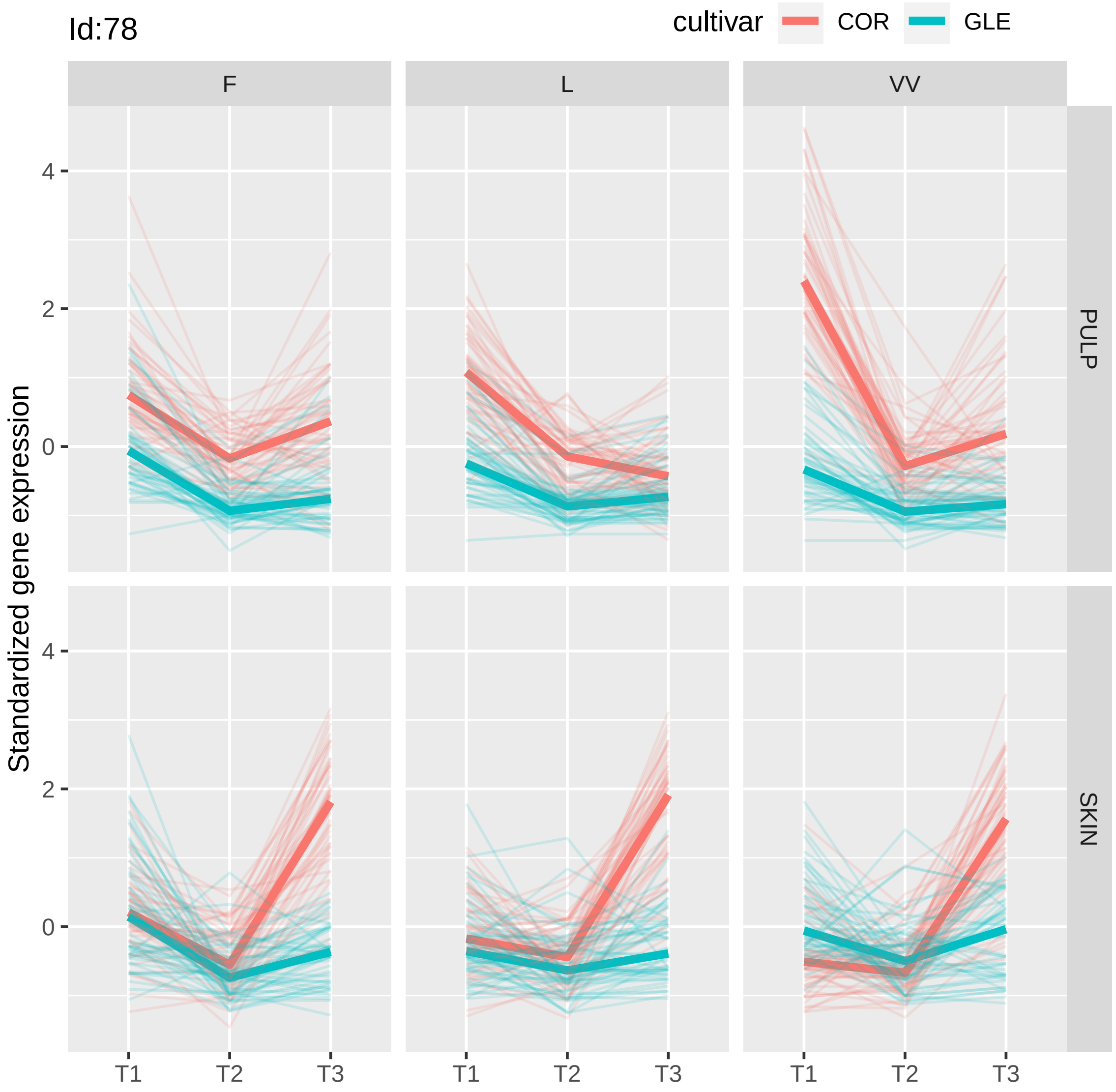
Id:78
cultivar
COR
GLE
F
L
VV
PULP
SKIN
Standardized gene expression
4
2
0
T1
T2
T3

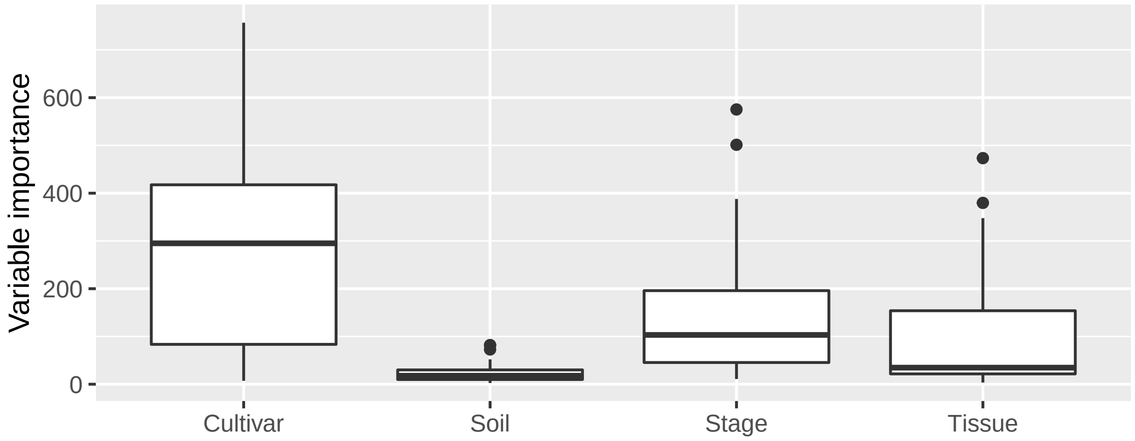
Variable importance
600
400
200
0
Cultivar
Soil
Stage
Tissue

Cluster no. 64

```
## Number of genes in the cluster: 81
## Homogeneity Index:    0.88
## Variable importance for Stage:     Rank =  64  - Median =  85.08
## Variable importance for Cultivar:  Rank =  78  - Median =  11.65
## Variable importance for Tissue:    Rank =  18  - Median =  673.6
## Variable importance for Soil:      Rank =  92  - Median =   3.33
##
## Gene ID                Gene Annotation
## VIT_01s0011g00740  -  Processing peptidase beta subunit, mitochondrial
## VIT_06s0004g06130  -  Thiamine biosynthesis protein ThiC
## VIT_04s0079g00410  -  CIR1/RVE2 (circadian 1)
## VIT_00s0214g00090  -  F-box protein PP2-B10 (Protein phloem protein 2-like B10)
## VIT_00s1525g00010  -  GAE3 (UDP-D- glcucuronate  4-epimerase 3)
## VIT_16s0050g00440  -  No hit
## VIT_14s0030g02310  -  Auxin-induced protein 22D
## VIT_08s0040g02800  -  Lipin
## VIT_13s0019g02080  -  DNA-binding protein
## VIT_08s0007g04970  -  Pentatricopeptide (PPR) repeat-containing
## VIT_16s0050g01660  -  UDP-glucoronosyl/UDP-glucosyl transferase UGT88A1
## VIT_03s0063g00940  -  CRK (CDPK-related kinase)
## VIT_18s0001g11600  -  Transducin protein
## VIT_00s0469g00010  -  Cellulose synthase CSLE1
## VIT_01s0011g05440  -  Unknown protein
## VIT_17s0000g08770  -  Cysteine-rich receptor-like protein kinase 2
## VIT_11s0016g03080  -  Clavata1 receptor kinase (CLV1)
## VIT_01s0026g01730  -  WRKY Transcription Factor (VvWRKY02)
## VIT_07s0095g00640  -  Serine/threonine kinase BRLK
## VIT_08s0040g02760  -  Ankyrin repeat
## VIT_02s0012g00730  -  Purine permease 10 PUP10
## VIT_01s0010g03930  -  WRKY Transcription Factor (VvWRKY03)
## VIT_18s0001g15660  -  Pathogen-related
## VIT_09s0018g00240  -  WRKY Transcription Factor (VvWRKY28)
## VIT_08s0007g04890  -  ACT domain-containing protein
## VIT_14s0068g00380  -  NLI interacting factor (NIF) family protein
## VIT_01s0011g03730  -  (myb domain protein 62
## VIT_14s0081g00730  -  Ethylene response factor ERF1
## VIT_04s0044g00650  -  S-receptor kinase
## VIT_18s0001g11620  -  WAK receptor protein kinase
## VIT_14s0108g00190  -  Aldose 1-epimerase
## VIT_19s0015g00010  -  ABC Transporter (VvMRP2 - VvABCC2)
## VIT_05s0077g01580  -  s10 (VvPR10.2)_Pathogenesis protein 10 [Vitis vinifera]
## VIT_02s0025g02920  -  caffeic acid 3-O-methyltransferase
## VIT_19s0015g00050  -  ABC Transporter (VvMRP5 - VvABCC5)
## VIT_14s0030g01980  -  Auxin-induced protein PCNT115
## VIT_04s0023g00700  -  Serine carboxypeptidase S10
## VIT_16s0013g01500  -  Disease resistance protein
## VIT_08s0007g07170  -  Prenyltransferase
## VIT_11s0016g01970  -  Calmodulin-binding region IQD6
## VIT_06s0004g07240  -  UDP-glucoronosyl and UDP-glucosyl transferase
## VIT_12s0028g00590  -  3-hydroxyisobutyryl-CoA hydrolase
## VIT_03s0017g00620  -  GA 20-oxidase
## VIT_03s0017g01290  -  ABC Transporter (VvWBC13 - VvABCG13)
## VIT_00s0294g00040  -  Receptor serine/threonine kinase
## VIT_01s0150g00120  -  ERF/AP2 Gene Family (VvERF112)
```

```
## VIT_17s0000g05440  -  Unknown protein
## VIT_12s0034g02560  -  DNA (cytosine-5)-methyltransferase (ATHIM)
## VIT_19s0015g00020  -  ABC Transporter (VvMRP3 - VvABCC3)
## VIT_04s0044g00220  -  Monooxygenase
## VIT_04s0008g06420  -  No hit
## VIT_06s0004g07230  -  Indole-3-acetate beta-glucosyltransferase
## VIT_10s0042g01050  -  Serine carboxypeptidase II
## VIT_08s0007g05490  -  Pyruvate kinase
## VIT_19s0015g00060  -  ABC transporter C member 9
## VIT_03s0038g03530  -  LYS/HIS transporter 7
## VIT_16s0148g00180  -  Kinase
## VIT_10s0003g04950  -  Esterase/lipase/thioesterase
## VIT_01s0011g04670  -  ABC Transporter (VvWBC7 - VvABCG7)
## VIT_16s0148g00250  -  Zinc finger (C3HC4-type ring finger)
## VIT_14s0030g01860  -  Transcription factor
## VIT_12s0034g00040  -  UDP-glucose glucosyltransferase
## VIT_17s0000g08150  -  basic helix-loop-helix (bHLH) family
## VIT_08s0040g01710  -  Phenylalanine ammonia-lyase (PAL1)
## VIT_00s0374g00040  -  S-locus lectin protein kinase
## VIT_12s0059g01850  -  Peroxisomal membrane protein
## VIT_17s0000g00650  -  No hit
## VIT_10s0116g00820  -  Adenosine/AMP deaminase
## VIT_10s0116g00680  -  PDF2 (protodermal factor2)
## VIT_12s0034g00080  -  Flavonoid-glucosyltransferase
## VIT_01s0011g03850  -  Unknown protein
## VIT_17s0000g06860  -  SNAP25ous protein SNAP30
## VIT_09s0002g03560  -  ABC Transporter (VvPDR4 - VvABCG34)
## VIT_01s0137g00070  -  B-keto acyl reductase
## VIT_13s0158g00400  -  R protein MLA10
## VIT_01s0010g02770  -  Adenylate kinase-b
## VIT_16s0013g01120  -  ERF/AP2 Gene Family (VvERF092)
## VIT_14s0006g02660  -  Receptor serine/threonine kinase PR5K-l
## VIT_13s0067g02500  -  Xanthine/uracil permease
## VIT_04s0044g00680  -  S-receptor kinase
## VIT_16s0050g02750  -  Zinc finger (C3HC4-type ring finger)
```

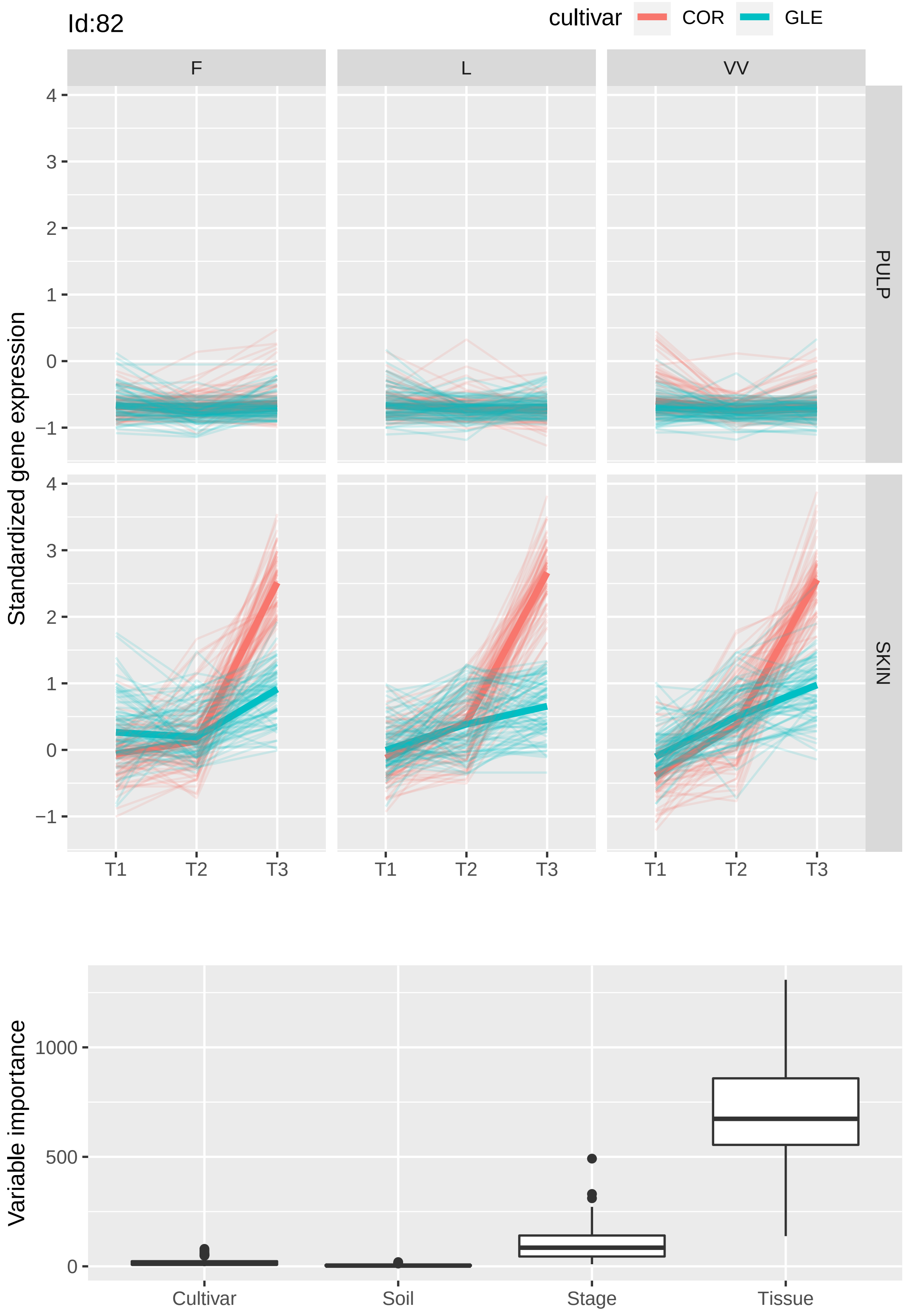
Id:82
cultivar
COR
GLE
F
L
VV
PULP
SKIN
Standardized gene expression
T1
T2
T3
Variable importance
1000
500
0
Cultivar
Soil
Stage
Tissue

Cluster no. 65

```
## Number of genes in the cluster: 51
## Homogeneity Index:    0.75
## Variable importance for Stage:     Rank =  65  - Median =  84.12
## Variable importance for Cultivar:  Rank =  10  - Median =    553
## Variable importance for Tissue:    Rank =  80  - Median =  25.46
## Variable importance for Soil:      Rank =  33  - Median =   6.23
##
## Gene ID               Gene Annotation
## VIT_01s0026g00410  -  Bromo-adjacenty (BAH) domain-containing protein
## VIT_02s0025g00290  -  Pentatricopeptide (PPR) repeat-containing protein
## VIT_16s0022g00440  -  Dynein light chain LC8-type
## VIT_05s0020g02170  -  Sugar transporter ERD6-like 16
## VIT_17s0000g00200  -  Ethylene-responsive transcription factor ERF114
## VIT_03s0017g02180  -  Pentatricopeptide (PPR) repeat-containing protein
## VIT_00s0179g00260  -  Calcium-transporting ATPase 12 ACA12
## VIT_13s0047g00220  -  Ribosomal protein S19
## VIT_01s0026g00420  -  Bromo adjacent homology (BAH) domain-containing protein
## VIT_10s0092g00790  -  Ribosomal protein S19
## VIT_10s0003g01200  -  Transcription regulator/ zinc ion binding
## VIT_06s0004g02950  -  Helicase conserved C-terminal domain containing protein
## VIT_15s0048g02010  -  Shaggy protein kinase theta
## VIT_01s0026g02120  -  Microtubule end binding protein 1 (EB1)
## VIT_10s0092g00780  -  F-type H+-transporting ATPase alpha chain
## ENSRNA049467215  -
## VIT_10s0003g05760  -  Pentatricopeptide repeat-containing protein
## VIT_01s0026g02510  -  Pentatricopeptide (PPR) repeat-containing
## VIT_01s0011g05920  -  S-adenosyl-L-methionine:salicylic acid carboxyl methyltransferase
## VIT_03s0017g00460  -  Magnesium-dependent phosphatase 1
## VIT_00s0179g00250  -  No hit
## VIT_03s0038g01930  -  Peptidyl-prolyl cis-trans isomerase ROC5 (rotamase CYP 5)
## VIT_04s0023g01790  -  phytoene dehydrogenase-related (PDH) (VvPDH2)
## VIT_03s0017g01990  -  UDP-glucose glucosyltransferase
## ENSRNA049469362  -
## VIT_04s0069g00530  -  Glutamate receptor protein
## VIT_05s0049g01350  -  Kinesin phragmoplast-associated kinesin-related protein 2 (PAKRP2)
## VIT_12s0035g01920  -  Methionine S-methyltransferase
## VIT_01s0011g05900  -  Jasmonate O-methyltransferase
## VIT_10s0003g01650  -  Unknown
## VIT_16s0013g00600  -  No hit
## VIT_05s0062g00990  -  Aldo/keto reductase AKR
## VIT_16s0050g02690  -  EMB2261
## VIT_08s0007g01470  -  Copper transporter 3
## VIT_04s0008g00540  -  Oleosin OLE-3
## VIT_13s0106g00460  -  NHL repeat-containing protein
## VIT_19s0014g04600  -  S-locus protein kinase
## VIT_00s0267g00020  -  Dynamin-2A
## VIT_00s0361g00110  -  Phosphoinositide binding
## VIT_00s0324g00050  -  UDP-glucose glucosyltransferase
## VIT_19s0085g00140  -  Abhydrolase domain-containing protein
## VIT_00s2620g00010  -  Endo-1,4-beta-glucanase korrigan (KOR)
## VIT_06s0004g00300  -  Hcr2-p4.1
## VIT_14s0081g00020  -  Pathogenesis-related protein-4 (Chitinase )
## VIT_01s0137g00700  -  Lipase GDSL
## VIT_09s0002g07090  -  Unknown protein
```

```
## VIT_15s0046g01610  -  Acidic endochitinase (CHIB1)
## VIT_05s0077g00500  -  myb domain protein 108
## VIT_03s0038g00020  -  Vacuolar protein sorting 45
## VIT_18s0001g00140  -  Unknown protein
## VIT_09s0054g00420  -  Translocon-associated protein beta TRAP complex
```

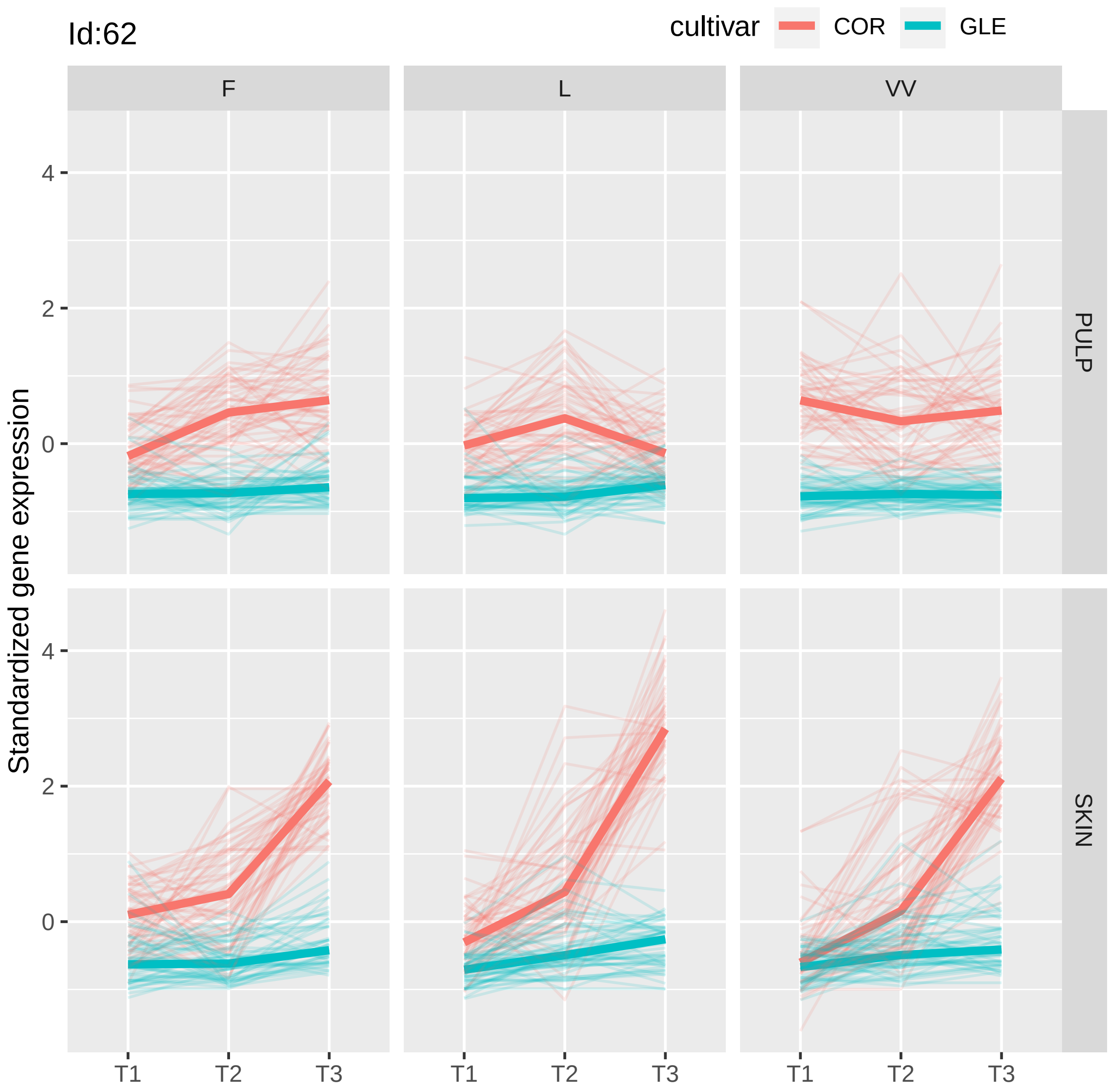
Id:62
cultivar
COR
GLE
F
L
VV
PULP
SKIN
Standardized gene expression
4
2
0
T1
T2
T3

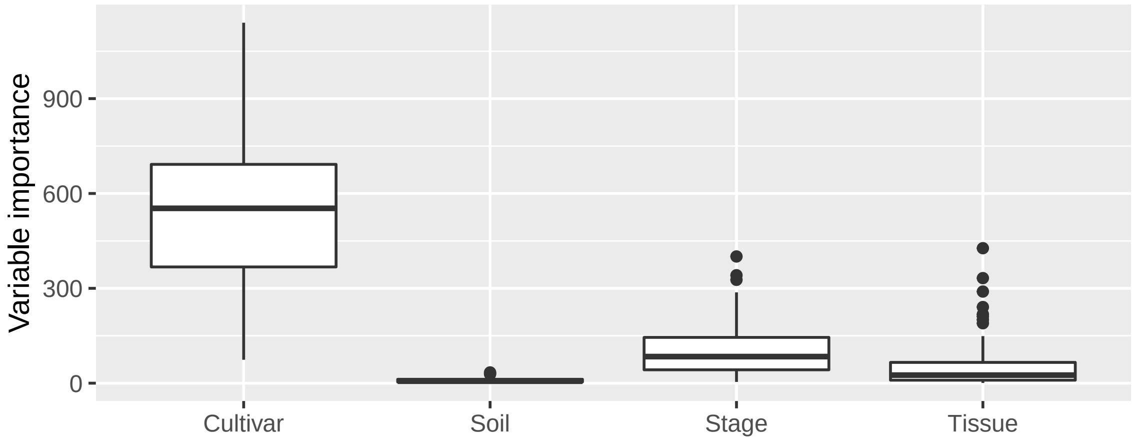
Variable importance
900
600
300
0
Cultivar
Soil
Stage
Tissue

Cluster no. 66

```
## Number of genes in the cluster: 110
## Homogeneity Index:    0.85
## Variable importance for Stage:     Rank =  66  - Median =  78.69
## Variable importance for Cultivar:  Rank =  30  - Median =  121.5
## Variable importance for Tissue:    Rank =  36  - Median =  245.9
## Variable importance for Soil:      Rank =  48  - Median =   5.71
##
## Gene ID               Gene Annotation
## VIT_05s0077g01570  -  s11_Pathogenesis protein 10 [Vitis vinifera]
## VIT_16s0100g00230  -  Glucose-methanol-choline (GMC) oxidoreductase family protein
## VIT_07s0031g01520  -  No hit
## VIT_06s0004g01690  -  Histone H3
## VIT_03s0038g04330  -  Unknown
## VIT_02s0012g02820  -  Geraniol 10-hydroxylase
## VIT_10s0003g01190  -  Ketol-acid reductoisomerase
## VIT_08s0007g03900  -  Heat stress transcription factor A3
## VIT_16s0039g01170  -  Phenylalanine ammonium lyase
## VIT_19s0027g00860  -  NAC domain-containing protein (VvNAC31)
## VIT_05s0094g00540  -  Acyl-[acyl-carrier-protein] desaturase
## VIT_09s0002g00510  -  Lipase GDSL 1
## VIT_13s0019g00540  -  Ethylene-responsive protein
## VIT_18s0001g04120  -  (-)-germacrene D synthase (VvTPS04)
## VIT_08s0032g00240  -  CYP71A22
## VIT_19s0014g04210  -  S-locus protein kinase
## VIT_06s0004g01250  -  Omega-6 fatty acid desaturase, endoplasmic reticulum (FAD2)
## VIT_15s0048g01080  -  Unknown
## VIT_14s0006g01610  -  PMI2 (plastid movement impaired 2)
## VIT_18s0117g00550  -  Laccase
## VIT_19s0015g00110  -  CYP71D10
## VIT_18s0001g13670  -  Glyoxal oxidase-related
## VIT_08s0007g00330  -  Metallothionein
## VIT_12s0028g03480  -  Wax synthase
## VIT_19s0015g01350  -  Stachyose synthase precursor
## VIT_00s0270g00120  -  Alpha-amylase/subtilisin inhibitor
## VIT_02s0025g04280  -  Osmotin
## VIT_04s0023g01330  -  Homeobox protein 5 (VvATHB-6)
## VIT_16s0098g00740  -  Ovate family protein 10 OFP10
## VIT_01s0127g00390  -  Pentatricopeptide (PPR) repeat
## VIT_12s0035g01820  -  Proton-dependent oligopeptide transport (POT) family protein
## VIT_04s0044g00230  -  Monooxygenase (MO2)
## VIT_18s0166g00070  -  No hit
## VIT_15s0048g01350  -  Gibberellin receptor GID1L3
## VIT_02s0012g01590  -  Unknown protein
## VIT_00s0258g00010  -  Receptor serine/threonine kinase
## VIT_11s0118g00160  -  Disease resistance protein
## VIT_19s0085g00050  -  myb domain protein 58
## VIT_04s0023g02900  -  Ferulate-5-hydroxylase
## VIT_19s0014g04200  -  S-locus protein kinase
## VIT_09s0002g08840  -  Unknown
## VIT_10s0003g01160  -  basic helix-loop-helix (bHLH) family
## VIT_00s0301g00120  -  Late embryogenesis abundant protein
## VIT_03s0088g01140  -  Squalene monooxygenase 2
## VIT_16s0022g01020  -  Chalcone synthase [Vitis vinifera]
## VIT_08s0007g00310  -  Protein phosphatase 2C POLTERGEIST-like 4
```

```
## VIT_11s0016g04330  -  Unknown
## VIT_00s0480g00030  -  Polyphenol oxidase
## VIT_09s0002g05610  -  Unknown
## VIT_00s0480g00040  -  Polyphenol oxidase II, chloroplast precursor
## VIT_08s0040g02730  -  Exocyst subunit EXO70 H4
## VIT_10s0003g01170  -  basic helix-loop-helix (bHLH) family
## VIT_15s0048g01250  -  putative MADS-box Supressor of Constans overexpression 1a (VviSOC1a)
## VIT_14s0006g01790  -  Beta-mannosidase
## VIT_03s0017g01550  -  CRK10 (cysteine-rich RLK10)
## VIT_16s0148g00160  -  Receptor serine/threonine kinase
## VIT_19s0014g04470  -  S-locus protein kinase
## VIT_00s0258g00050  -  Receptor serine/threonine kinase
## VIT_06s0004g04970  -  Scarecrow transcription factor 14 (SCL14)
## VIT_06s0004g01480  -  Lipoxygenase LOX1 (VvLOXH)
## VIT_19s0014g04690  -  Indole-3-acetic acid-amido synthetase
## VIT_14s0128g00520  -  Alpha-L-fucosidase 2 precursor
## VIT_02s0033g00870  -  Nitrilase 4B
## VIT_00s0294g00010  -  Receptor serine/threonine kinase
## VIT_04s0008g01250  -  Unknown
## VIT_07s0005g04050  -  Leucine Rich Repeat receptor-like kinase
## VIT_08s0040g00440  -  Unknown protein
## VIT_09s0002g06590  -  UDP-glucose:flavonoid 7-O-glucosyltransferase
## VIT_02s0087g00710  -  Abscisic acid 8` hydroxylase (CYP707A1) (VvA8H-CYP707A1)
## VIT_05s0049g00430  -  1-aminocyclopropane-1-carboxylate oxidase homolog 1
## VIT_15s0048g02060  -  Hydrolase, alpha/beta fold
## VIT_11s0016g01860  -  RPS2 (resistant to p. syringae 2)
## VIT_00s0333g00040  -  Early tobacco anther 1, putative
## VIT_08s0007g04100  -  CYPLXXVIA2
## VIT_00s0160g00360  -  TIR-NBS-LRR disease resistance
## VIT_06s0009g03010  -  Flavonoid 3',5'-hydroxylase (F3'5'H)
## VIT_18s0001g01730  -  Shikimate kinase
## VIT_05s0094g00220  -  Chitinase class IV
## VIT_03s0063g01790  -  Transducin protein
## VIT_09s0002g08640  -  Protein kinase Xa21
## VIT_04s0023g01120  -  Galacturonosyltransferase
## VIT_01s0011g04010  -  Catalytic
## VIT_07s0005g03340  -  VvMyb14
## VIT_03s0088g00260  -  Serine carboxypeptidase S10
## VIT_06s0009g03000  -  Flavonoid 3',5'-hydroxylase
## VIT_16s0050g02740  -  Receptor-like protein kinase
## VIT_03s0038g00430  -  Enhanced disease susceptibility 5 EDS5
## VIT_16s0148g00410  -  Receptor serine/threonine kinase
## VIT_16s0039g01210  -  Ser/Thr receptor-like kinase1
## VIT_11s0052g01090  -  4-coumarate-CoA ligase 2
## VIT_17s0000g09820  -  DNA polymerase III gamma and tau subunit
## VIT_06s0009g02810  -  Flavonoid 3',5'-hydroxylase
## VIT_16s0098g00120  -  Polynucleotidyl transferase, Ribonuclease H fold
## VIT_10s0003g00790  -  Glutamate receptor protein
## VIT_08s0007g03560  -  Anthocyanin membrane protein 1 (Anm1)
## VIT_08s0007g04120  -  CYPLXXVIA2
## VIT_06s0009g02840  -  Flavonoid 3',5'-hydroxylase(F3'5'H)
## VIT_01s0127g00260  -  ATP-citrate synthase
## VIT_16s0039g01200  -  Receptor serine/threonine kinase
## VIT_00s0424g00010  -  Ser/Thr receptor-like kinase1
## VIT_16s0148g00170  -  Protein binding / zinc ion binding
```

```
## VIT_12s0121g00300  -  Brassinosteroid insensitive 1-associated receptor kinase 1
## VIT_00s2248g00010  -  Rust resistance kinase Lr10
## VIT_00s1321g00010  -  Unknown
## VIT_06s0009g02970  -  flavonoid 3',5'-hydroxylase j (F3'5'Hj) [Vitis vinifera]
## VIT_16s0148g00320  -  Ser/Thr receptor-like kinase1
## VIT_16s0098g00020  -  Receptor serine/threonine kinase
## VIT_15s0048g02900  -  CYP78A3p
## VIT_00s0258g00100  -  Receptor serine/threonine kinase
## VIT_00s1467g00010  -  Brassinosteroid insensitive 1-associated receptor kinase 1
```

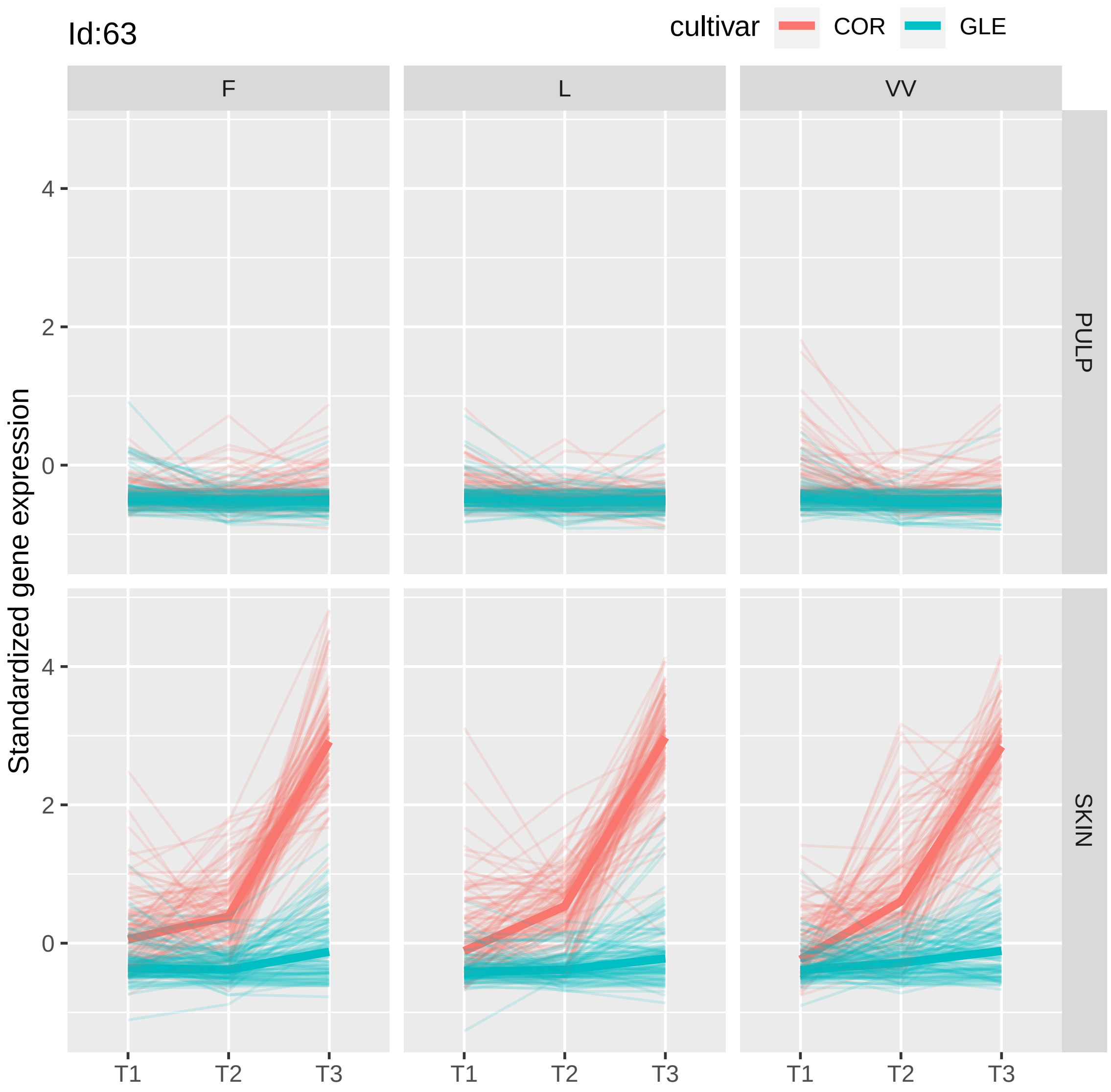
Id:63
cultivar
COR
GLE
F
L
VV
PULP
SKIN
Standardized gene expression
4
2
0
T1
T2
T3

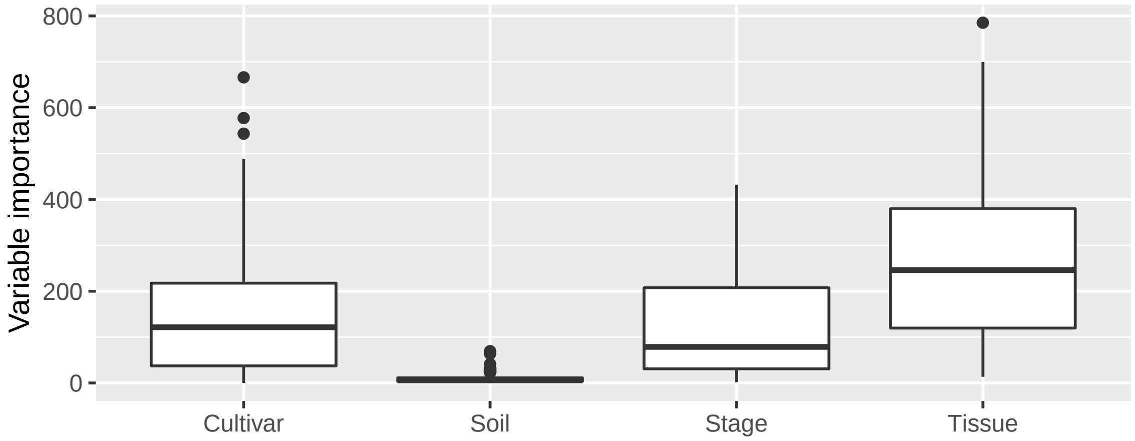
Variable importance
800
600
400
200
0
Cultivar
Soil
Stage
Tissue

Cluster no. 67

```
## Number of genes in the cluster: 47
## Homogeneity Index:    0.79
## Variable importance for Stage:     Rank =  67  - Median =  71.98
## Variable importance for Cultivar:  Rank =  33  - Median =    103
## Variable importance for Tissue:    Rank =  27  - Median =  418.1
## Variable importance for Soil:      Rank =  18  - Median =   7.93
##
## Gene ID                Gene Annotation
## VIT_06s0080g00790  -  MYB divaricata
## VIT_14s0066g01240  -  L-aspartate oxidase
## VIT_09s0002g07860  -  Glycerol kinase
## VIT_14s0083g00530  -  Cell division protein FtsH
## VIT_00s0187g00120  -  Cystatin
## VIT_14s0081g00030  -  Pathogenesis-related protein-4 (Chitinase )
## VIT_01s0011g04940  -  No hit
## VIT_08s0007g02370  -  Unknown
## VIT_08s0007g01930  -  Anthranilate N-benzoyltransferase
## VIT_05s0077g00900  -  No hit
## VIT_13s0147g00110  -  ferredoxin
## VIT_00s0414g00010  -  Cellulose synthase CSLE1
## VIT_16s0098g01380  -  Yippee
## VIT_05s0049g00010  -  Cellulose synthase CSLG2
## VIT_10s0003g04820  -  No hit
## VIT_00s2365g00010  -  Kinase
## VIT_14s0030g00670  -  Dehydroquinate dehydratase
## VIT_17s0000g04370  -  Wall-associated kinase
## VIT_07s0031g00350  -  caffeoyl-CoA O-methyltransferase 1 (CCoAOMT1)
## VIT_08s0058g00150  -  Ammonium transporter 2
## VIT_14s0171g00400  -  Lysine histidine transporter 1
## VIT_05s0020g04820  -  H1flk
## VIT_01s0010g03680  -  Unknown protein
## VIT_03s0017g02170  -  Zinc transporter ZIP5
## VIT_13s0047g00230  -  Pectinesterase family
## VIT_06s0004g06840  -  Protein phosphatase 2C (VvPP2C-5)
## VIT_16s0098g00190  -  Receptor kinase homolog LRK10
## VIT_19s0014g04960  -  Nodulation receptor kinase
## VIT_14s0060g00980  -  Unknown
## VIT_16s0148g00140  -  Ser/Thr receptor-like kinase1
## VIT_01s0011g05960  -  Trehalose-phosphatase
## VIT_14s0171g00490  -  Glucose-6-phosphate 1-dehydrogenase, cytoplasmic isoform
## VIT_00s0301g00080  -  No hit
## VIT_05s0020g01290  -  TOC159 (translocon outer membrane complex 159)
## VIT_03s0017g01700  -  Glycine cleavage system H protein, mitochondrial
## VIT_03s0038g01380  -  Calcium-binding EF hand
## VIT_04s0023g01270  -  Transporter-related
## VIT_04s0044g00510  -  GT2-like trihelix DNA-binding protein
## VIT_04s0008g02860  -  Cation transport protein chaC
## VIT_19s0027g01630  -  Disease resistance protein (NBS-LRR class)
## VIT_07s0005g02130  -  TolB protein-related
## VIT_08s0007g03570  -  Anthocyanin membrane protein 1 (Anm1)
## VIT_18s0001g11560  -  CyP82A3
## VIT_00s0391g00070  -  3-deoxy-D-arabino-heptulosonate 7-phosphate synthase
## VIT_19s0014g04280  -  S-locus protein kinase
## VIT_18s0001g11150  -  Acyl-peptide hydrolase
```

```
## VIT_02s0087g00340  -  NFD4 (nuclear fusion defective 4)
```

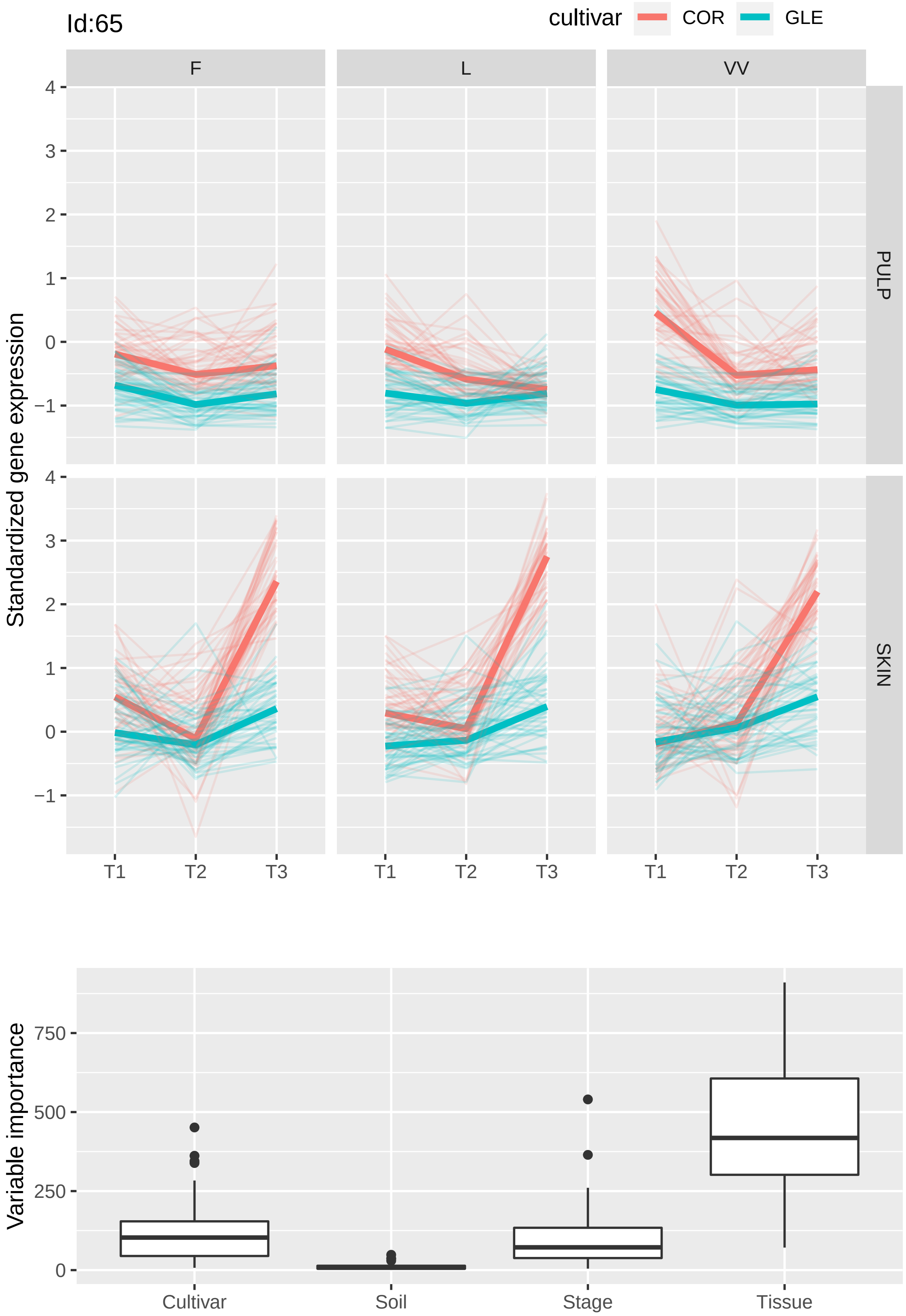

Id:65
cultivar
COR
GLE
F
L
VV
PULP
SKIN
Standardized gene expression
T1
T2
T3
Variable importance
Cultivar
Soil
Stage
Tissue

Cluster no. 68

```
## Number of genes in the cluster: 69
## Homogeneity Index:    0.73
## Variable importance for Stage:     Rank =  68  - Median =  68.07
## Variable importance for Cultivar:  Rank =  73  - Median =  14.55
## Variable importance for Tissue:    Rank =  16  - Median =  697.9
## Variable importance for Soil:      Rank =  19  - Median =   7.85
##
## Gene ID               Gene Annotation
## VIT_05s0051g00070  -  Coenzyme F420 hydrogenase/dehydrogenase beta subunit
## VIT_18s0001g10800  -  Carboxylesterase CXE
## VIT_19s0014g00750  -  Leucine-rich repeat protein kinase
## VIT_10s0071g00450  -  S-locus lectin protein kinase
## VIT_13s0084g00120  -  No hit
## VIT_15s0107g00550  -  Tetratricopeptide repeat domain male sterility MS5
## VIT_01s0026g01710  -  Zinc knuckle
## VIT_12s0059g00760  -  Adenosine 5' phosphosulfate reductase
## VIT_09s0002g02320  -  Cell wall apoplastic invertase
## VIT_01s0150g00440  -  Respiratory burst oxidase protein D (RBOHD)
## VIT_01s0011g05930  -  S-adenosyl-L-methionine:carboxyl methyltransferase
## VIT_10s0116g00920  -  CERK1 (chitin elicitor receptor kinase 1)
## VIT_06s0080g00280  -  Bile acid sodium symporter
## VIT_09s0054g00800  -  Unknown protein
## VIT_12s0028g00520  -  Unknown protein
## VIT_03s0017g02000  -  Anthocyanidin 3-O-glucosyltransferase
## VIT_19s0014g00250  -  Bile acid:sodium symporter
## VIT_14s0108g00340  -  Leucine Rich Repeat receptor-like kinase
## VIT_18s0041g00350  -  Dienelactone hydrolase
## VIT_00s0757g00030  -  Histidine triad family protein / HIT family protein
## VIT_07s0289g00100  -  Acetyl-CoA carboxylase
## VIT_16s0098g00860  -  Flavanone 3-hydroxylase
## VIT_01s0011g03740  -  Glutaminyl-tRNA synthetase
## VIT_07s0031g00480  -  Protein kinase family
## VIT_01s0010g01220  -  Unknown protein
## VIT_15s0046g02170  -  3-methyl-2-oxobutanoate hydroxy-methyl-transferase
## VIT_07s0031g02540  -  Emb2170 (embryo defective 2170)
## VIT_14s0030g00470  -  Stress regulated protein isoform 3
## VIT_19s0015g02520  -  CYP72A1
## VIT_08s0032g00270  -  CYP71D64
## VIT_08s0007g06700  -  Thioredoxin 5
## VIT_12s0121g00060  -  R protein MLA10
## VIT_07s0031g01150  -  FK506-binding protein genes family (VvFKBP16-2)
## VIT_15s0024g00690  -  Unknown protein
## VIT_06s0004g07280  -  UDP-glucuronosyl/UDP-glucosyltransferase
## VIT_18s0001g04980  -  Acetyl-CoA carboxylase 2 (ACC2)
## VIT_14s0083g00460  -  Tryptophan synthase beta chain 2
## VIT_16s0050g01690  -  Receptor kinase homolog LRK10
## VIT_00s0174g00070  -  Ankyrin repeat protein
## VIT_12s0035g02090  -  Leucine-rich repeat family protein
## VIT_12s0028g00060  -  Pentatricopeptide (PPR) repeat-containing protein
## VIT_01s0011g05470  -  PGPS/D12
## VIT_06s0061g01540  -  KCBP- interacting  protein kinase
## VIT_13s0019g04220  -  Tryptophan/tyrosine permease family
## VIT_01s0010g03630  -  Zinc finger (MYND type)
## VIT_01s0010g00920  -  Chaperonin GroEL
```

```
## VIT_00s0211g00100  -  Glycine hydroxymethyltransferase
## VIT_03s0063g01970  -  Non-symbiotic hemoglobin 2
## VIT_05s0020g03010  -  Carboxylesterase CXE
## VIT_13s0047g00210  -  flavonol synthase
## VIT_18s0001g14410  -  Unknown protein
## VIT_12s0059g00710  -  Metalloendopeptidase
## VIT_04s0023g03290  -  Unknown protein
## VIT_18s0001g12840  -  ADP-glucose pyrophosphorylase large subunit CagpL2
## VIT_00s1217g00010  -  3-deoxy-D-arabino-heptulosonate 7-phosphate synthase
## VIT_06s0009g03640  -  Annexin 1 (ANN1)
## VIT_00s0471g00010  -  U-box domain-containing protein
## VIT_11s0037g00830  -  Unknown protein
## VIT_12s0028g01390  -  Small heat shock protein ACD31
## VIT_15s0048g02500  -  Thylakoid membrane phosphoprotein 14 kda, chloroplast precursor
## VIT_11s0016g00740  -  Receptor-like protein kinase 3
## VIT_13s0019g05400  -  RPG related protein 1 RR1
## VIT_01s0150g00170  -  SEC14 cytosolic factor
## VIT_00s0211g00070  -  Glycine hydroxymethyltransferase
## VIT_16s0050g00750  -  Short-chain dehydrogenase/reductase
## VIT_16s0039g00920  -  CYP89A28
## VIT_00s0227g00150  -  Unknown protein
## VIT_16s0050g01260  -  Unknown protein
## VIT_19s0015g02780  -  CYP72A59
```

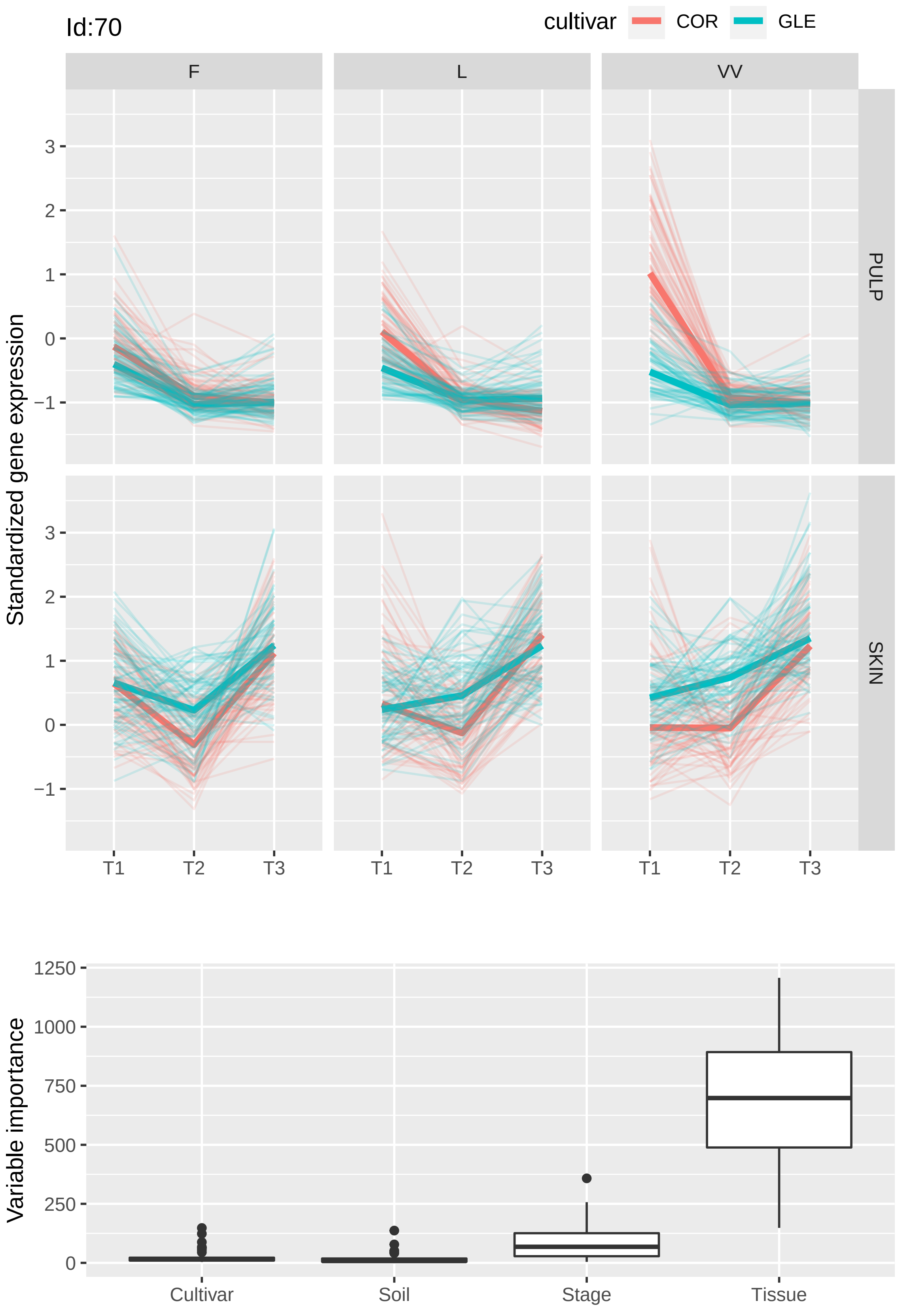
Id:70
cultivar
COR
GLE
F
L
VV
PULP
SKIN
Standardized gene expression
3
2
1
0
−1
T1
T2
T3
Variable importance
1250
1000
750
500
250
0
Cultivar
Soil
Stage
Tissue

Cluster no. 69

```
## Number of genes in the cluster: 33
## Homogeneity Index:    0.62
## Variable importance for Stage:     Rank =  69  - Median =   67.7
## Variable importance for Cultivar:  Rank =  17  - Median =    260
## Variable importance for Tissue:    Rank =  62  - Median =  77.97
## Variable importance for Soil:      Rank =   7  - Median =   15.3
##
## Gene ID               Gene Annotation
## VIT_11s0016g05530  -  Plastocyanin domain-containing protein
## VIT_18s0075g00560  -  fatty acid multifunctional protein (MFP2)
## VIT_17s0000g02790  -  Unknown
## VIT_13s0067g00350  -  U-box domain-containing protein 34
## VIT_09s0002g09040  -  Patellin-5
## VIT_05s0094g01530  -  ARL (ARGOS)
## VIT_05s0020g04160  -  Stem-specific protein TSJT1
## VIT_09s0002g07980  -  La domain-containing protein
## VIT_00s0274g00080  -  Benzoquinone reductase
## VIT_01s0137g00080  -  Aldehyde dehydrogenase (NAD+)
## VIT_09s0002g03670  -  No hit
## VIT_02s0012g00170  -  1,4-alpha-D-glucan maltohydrolase
## VIT_06s0061g01470  -  ABC Transporter (VvPDR22 - VvABCG52)
## VIT_15s0046g02300  -  Beta-cyanoalanine synthase
## VIT_14s0108g00100  -  Serine carboxypeptidase S10
## VIT_18s0001g08710  -  Basic Leucine Zipper Transcription Factor (VvbZIP44)
## VIT_09s0002g05010  -  Kelch repeat-containing F-box family protein
## VIT_04s0044g02000  -  BCL-2-associated athanogene 4
## VIT_02s0109g00420  -  Disease resistance protein (CC-NBS-LRR class)
## VIT_17s0053g00160  -  Apyrase
## VIT_00s0349g00030  -  Skp1
## VIT_07s0129g00110  -  Ca2+-ATPase 2 ACA2, plasma membrane
## VIT_07s0095g00040  -  Disease resistance protein (TIR-NBS-LRR class)
## VIT_11s0016g03200  -  Gibberellin oxidase
## VIT_08s0007g00320  -  GTP binding protein
## VIT_15s0046g01360  -  No hit
## VIT_05s0051g00680  -  Unknown protein
## VIT_05s0077g00540  -  No hit
## VIT_16s0022g00240  -  EIX receptor 2
## VIT_16s0039g01380  -  Shikimate kinase
## VIT_00s0483g00040  -  No hit
## VIT_00s0153g00070  -  Glutathione S-transferase 8 GSTU8
## VIT_00s0407g00060  -  Catalytic/ transferase
```

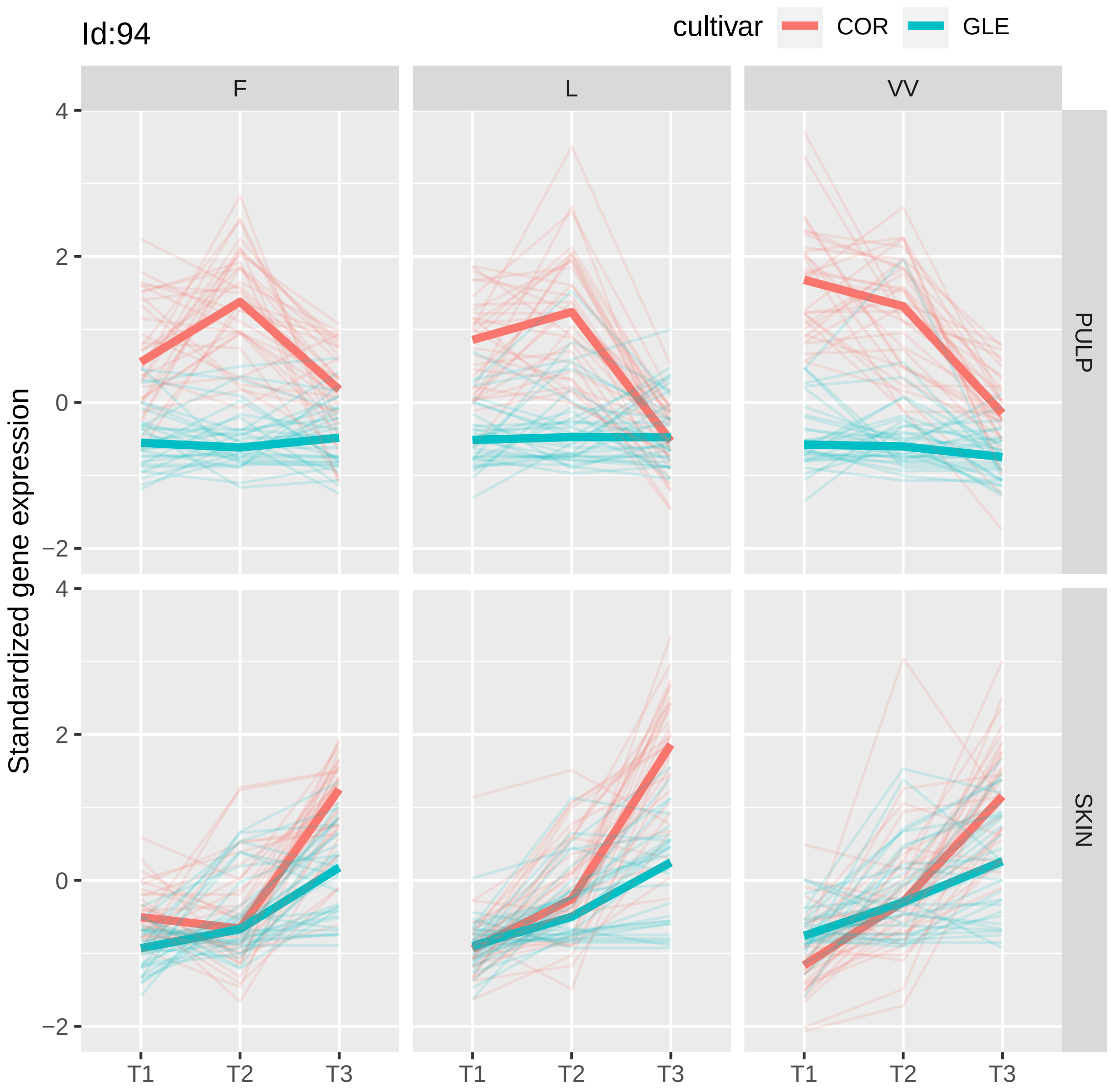
Id:94
cultivar
COR
GLE
F
L
VV
PULP
SKIN
Standardized gene expression
4
2
0
−2
T1
T2
T3

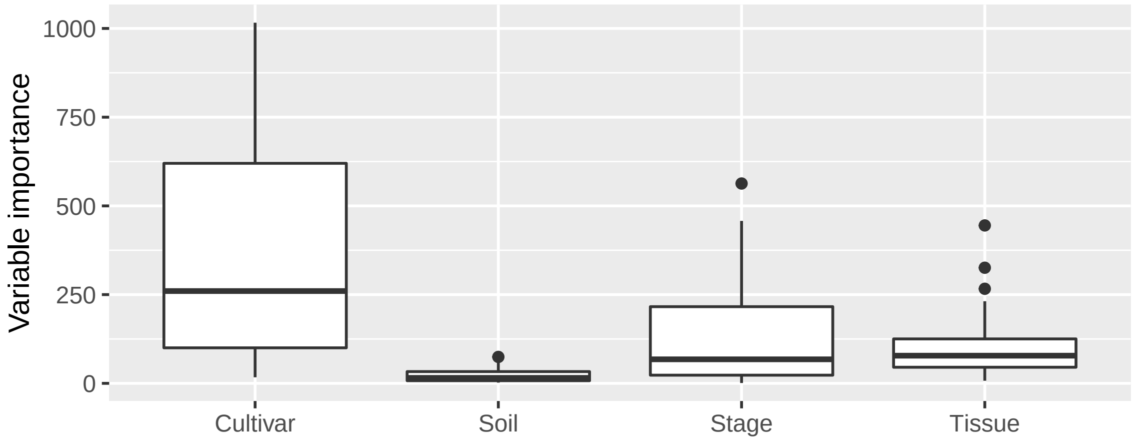
Variable importance
1000
750
500
250
0
Cultivar
Soil
Stage
Tissue

Cluster no. 70

```
## Number of genes in the cluster: 91
## Homogeneity Index:    0.83
## Variable importance for Stage:     Rank =  70  - Median =  66.47
## Variable importance for Cultivar:  Rank =  38  - Median =  79.08
## Variable importance for Tissue:    Rank =  22  - Median =  504.9
## Variable importance for Soil:      Rank =  71  - Median =   4.61
##
## Gene ID                Gene Annotation
## VIT_16s0039g00820  -  CYP89A5
## VIT_17s0000g08680  -  IMP dehydrogenase/GMP reductase
## VIT_11s0016g02900  -  Leucine-rich repeat family protein / extensin
## VIT_06s0061g00890  -  Carboxypeptidase
## VIT_00s2839g00010  -  No hit
## VIT_01s0011g04740  -  LPR1 (Low Phosphate Root1) multicopper oxidase
## VIT_17s0000g04990  -  putative MADS-box Fruitfull  1 (VviFUL1)
## VIT_07s0141g00270  -  Auxin-induced protein 22D
## VIT_00s0218g00220  -  UDP-glucoronosyl/UDP-glucosyl transferase
## VIT_07s0129g00240  -  C2H2 zinc finger protein AZF1
## VIT_12s0028g04030  -  Ankyrin repeat
## VIT_19s0135g00160  -  Zinc finger (C3HC4-type ring finger)
## VIT_12s0059g01190  -  Homeobox protein shoot MERISTEMLESS (STM)
## VIT_12s0028g01870  -  Receptor kinase TRKa
## VIT_15s0048g02630  -  RNA binding motif protein 38
## VIT_18s0001g09720  -  S-domain receptor kinase
## VIT_05s0020g03350  -  SEC14 cytosolic factor
## VIT_16s0039g00760  -  CYP89A2
## VIT_07s0005g02440  -  Pectinacetylesterase
## VIT_18s0001g09730  -  S-domain receptor kinase
## VIT_17s0000g02850  -  No hit
## VIT_00s0225g00080  -  MATE efflux family protein
## VIT_00s0399g00020  -  Protease inhibitor/seed storage/lipid transfer protein (LTP)
## VIT_10s0003g01550  -  IMK2 (inflorescence meristem receptor-like kinase 2)
## VIT_18s0001g14810  -  Lipase 3 (EXL3) family II extracellular
## VIT_07s0129g00360  -  Peroxidase 73
## VIT_14s0030g00860  -  No hit
## VIT_04s0008g00860  -  Ndr family protein
## VIT_02s0025g04360  -  S-N-methylcoclaurine 3'-hydroxylase
## VIT_18s0001g15550  -  Serine carboxypeptidase II
## VIT_02s0025g01090  -  Unknown protein
## VIT_07s0129g00320  -  formamidase, putative / formamide amidohydrolase, putative
## VIT_15s0046g00590  -  Wax synthase
## VIT_05s0077g00530  -  Unknown protein
## VIT_08s0040g01540  -  Extra sporogenous cells
## VIT_12s0028g01860  -  Protein kinase Xa21
## VIT_00s0194g00340  -  No hit
## VIT_17s0000g06730  -  No hit
## VIT_02s0025g02970  -  Flavanone 3-hydroxylase (F3H)
## VIT_10s0003g00600  -  Lipase GDSL
## VIT_12s0055g00320  -  UDP-glucose: anthocyanidin 5,3-O-glucosyltransferase
## VIT_14s0083g00120  -  Myb domain protein 91
## VIT_13s0019g02810  -  Glucuronosyl transferase
## VIT_04s0008g02520  -  ATP binding protein
## VIT_14s0030g00990  -  R protein disease resistance protein
## VIT_08s0040g01550  -  Extra sporogenous cells
```

```
## VIT_01s0150g00300  -  Indole-3-acetic acid amido synthetase
## VIT_09s0054g01220  -  Cycloartenol synthase
## VIT_02s0025g00350  -  Heavy-metal-associated domain-containing protein
## VIT_05s0049g01650  -  Cyclopropane fatty acid synthase
## VIT_01s0011g04720  -  Multi copper oxidase type 1
## VIT_14s0060g02630  -  Unknown protein
## VIT_14s0068g00090  -  feronia receptor-like kinase
## VIT_12s0142g00780  -  Leucine-rich repeat transmembrane protein kinase
## VIT_00s0409g00080  -  S-receptor kinase KIK1 precursor
## VIT_12s0028g01920  -  Receptor kinase TRKe
## VIT_13s0067g02390  -  Unknown
## VIT_12s0035g00070  -  Leucine Rich Repeat receptor-like kinase
## VIT_12s0142g00760  -  Leucine-rich repeat protein kinase
## VIT_14s0219g00280  -  Glycerol-3-phosphate dehydrogenase (NAD+)
## VIT_15s0046g03310  -  Unknown protein
## VIT_15s0046g03240  -  Unknown protein
## VIT_19s0015g01800  -  Nucleoside triphosphatase
## VIT_10s0092g00370  -  CYP71D7
## VIT_18s0086g00200  -  Lectin protein kinase family
## VIT_18s0041g00200  -  R protein L6
## VIT_05s0049g01400  -  Alcohol oxidase
## VIT_00s0409g00070  -  No hit
## VIT_07s0031g00080  -  WRKY Transcription Factor (VvWRKY21)
## VIT_19s0090g00840  -  WRKY Transcription Factor (VvWRKY55)
## VIT_09s0054g01360  -  Cycloartenol synthase
## VIT_18s0001g15520  -  Leaf senescence protein
## VIT_14s0006g02650  -  Receptor-like protein kinase
## VIT_06s0004g05360  -  Tropinone reductase
## VIT_00s1543g00010  -  Unknown protein
## VIT_15s0021g02690  -  Unfertilized embryo sac 10 UNE10
## VIT_09s0054g01480  -  Beta-amyrin synthase
## VIT_01s0011g01890  -  Glutathione S-transferase, C-terminal
## VIT_03s0038g00500  -  ferulate 5-hydroxylase
## VIT_09s0054g01390  -  Beta-amyrin synthase
## VIT_01s0010g02220  -  Zinc finger (C3HC4-type ring finger)
## VIT_00s0516g00010  -  Unknown
## VIT_12s0055g00310  -  UDP-glucose glucosyltransferase
## VIT_08s0040g02180  -  Mlo3
## VIT_10s0003g00560  -  Wuschel homeobox 9
## VIT_11s0016g03590  -  Transducin protein
## VIT_09s0054g01370  -  Beta-amyrin synthase
## VIT_19s0014g04170  -  Curculin (mannose-binding) lectin
## VIT_12s0034g01120  -  UDP-glycosyltransferase 71A13
## VIT_04s0023g00990  -  Unknown protein
## VIT_11s0065g00040  -  CYP706A12
```

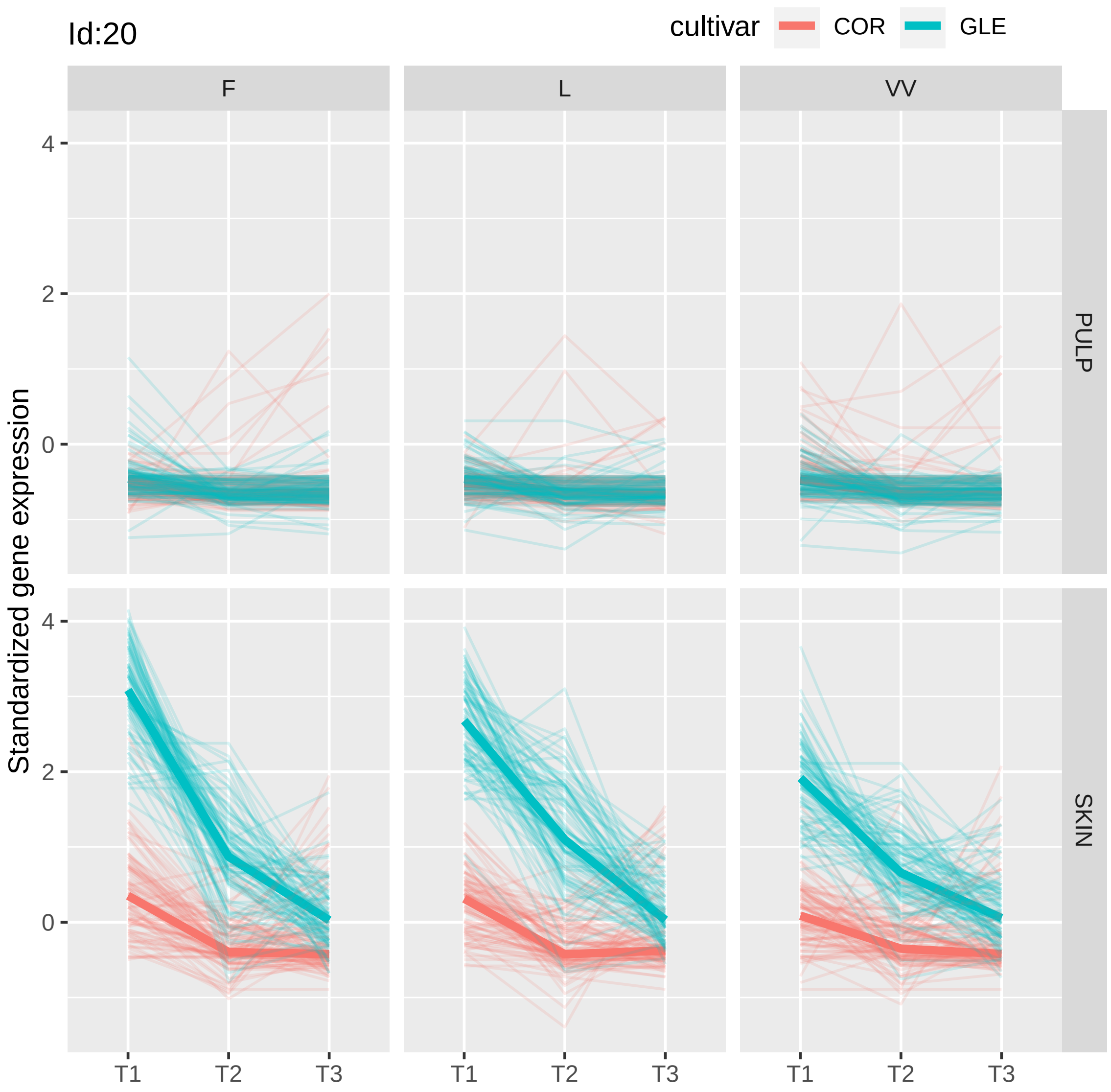

Id:20
cultivar
COR
GLE
F
L
VV
PULP
SKIN
Standardized gene expression
4
2
0
T1
T2
T3

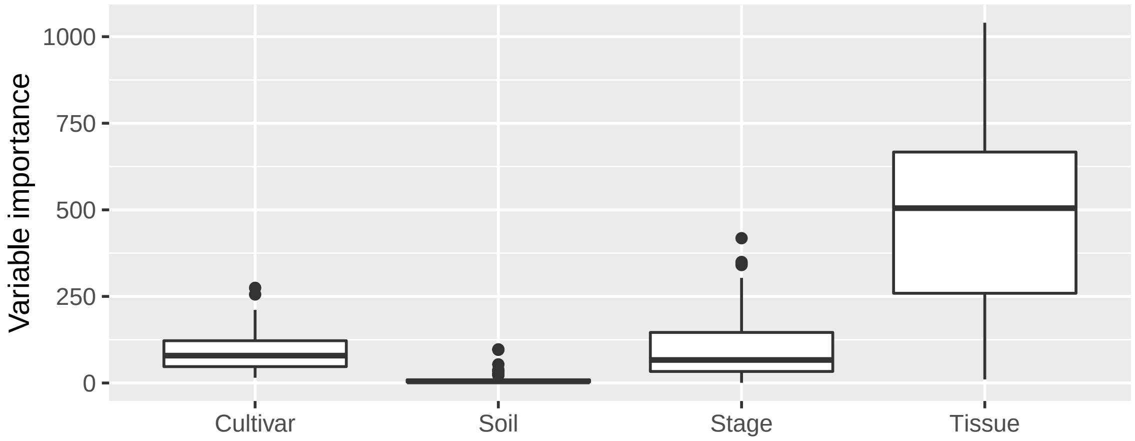

Variable importance
1000
750
500
250
0
Cultivar
Soil
Stage
Tissue

Cluster no. 71

```
## Number of genes in the cluster: 46
## Homogeneity Index:    0.86
## Variable importance for Stage:     Rank =  71  - Median =  66.12
## Variable importance for Cultivar:  Rank =  49  - Median =  47.24
## Variable importance for Tissue:    Rank =  15  - Median =    698
## Variable importance for Soil:      Rank =  82  - Median =   3.73
##
## Gene ID               Gene Annotation
## VIT_05s0049g00840  -  No hit
## VIT_05s0049g00780  -  No hit
## VIT_06s0004g01830  -  NIMA-related protein kinase
## VIT_09s0002g00340  -  Alanine aminotransferase 2
## VIT_18s0075g00520  -  Retrotransposon protein, Unclassified
## VIT_02s0025g00030  -  Zinc finger (C3HC4-type ring finger)
## VIT_08s0058g01120  -  Unknown
## VIT_01s0150g00380  -  Unknown protein
## VIT_18s0001g07840  -  Unknown protein
## VIT_11s0016g04920  -  Early nodulin 93
## VIT_04s0044g01120  -  Alcohol dehydrogenase 2 [Vitis vinifera]
## VIT_08s0040g02590  -  Kinesin motor protein
## VIT_08s0040g03260  -  Nuclear transcription factor Y subunit B-5
## VIT_08s0058g01000  -  Aspartate aminotransferase
## VIT_05s0049g02240  -  AWPM-19
## VIT_07s0005g00660  -  Late embryogenis abundant protein 5
## VIT_16s0022g01770  -  Phosphopyruvate hydratase.
## VIT_11s0016g00540  -  Respiratory burst oxidase protein E (RBOHE)
## VIT_14s0108g00090  -  Cupin, RmlC-type
## VIT_18s0001g02730  -  Unknown
## VIT_00s1466g00010  -  No hit
## VIT_01s0146g00260  -  Nodulin MtN3
## VIT_11s0016g00710  -  TIFY gene family (VvJAZ9)
## VIT_14s0060g01300  -  Universal stress protein (USP) family protein
## VIT_14s0060g01320  -  Universal stress protein (USP) family protein
## VIT_05s0049g00040  -  Cellulose synthase CSLG2
## VIT_19s0015g01370  -  Molecular chaperone DnaJ
## VIT_08s0056g00740  -  Unknown protein
## VIT_13s0158g00100  -  putative MADS-box Agamous-like 15a (VviAGL15a)
## VIT_02s0025g01070  -  CHCH domain containing protein
## VIT_15s0046g00150  -  DOF affecting germination 1
## VIT_18s0001g02250  -  Ras-related protein Rab-7A
## VIT_12s0059g00590  -  Allergenic protein Pt2L4
## VIT_07s0104g00150  -  Outer envelope membrane protein
## VIT_08s0032g00760  -  Translation initiation factor eIF-2 beta subunit
## VIT_13s0158g00090  -  Unknown
## VIT_04s0008g00840  -  Ser/Thr specific protein phosphatase 2A B regulatory subunit beta isof
## VIT_16s0050g00670  -  Kinesin motor
## VIT_03s0091g00470  -  Protein phosphatase 2C
## VIT_05s0049g00050  -  Cellulose synthase CSLG2
## VIT_03s0063g00640  -  Transcriptional factor B3
## VIT_11s0016g04590  -  ABC transporter g family pleiotropic drug resistance 12 PDR12
## VIT_05s0049g01850  -  Unknown protein
## VIT_14s0060g01490  -  DnaJ homolog, subfamily A, member 5
## VIT_00s1428g00020  -  No hit
## VIT_08s0007g03830  -  fructose-bisphosphate aldolase cytoplasmic isozyme
```

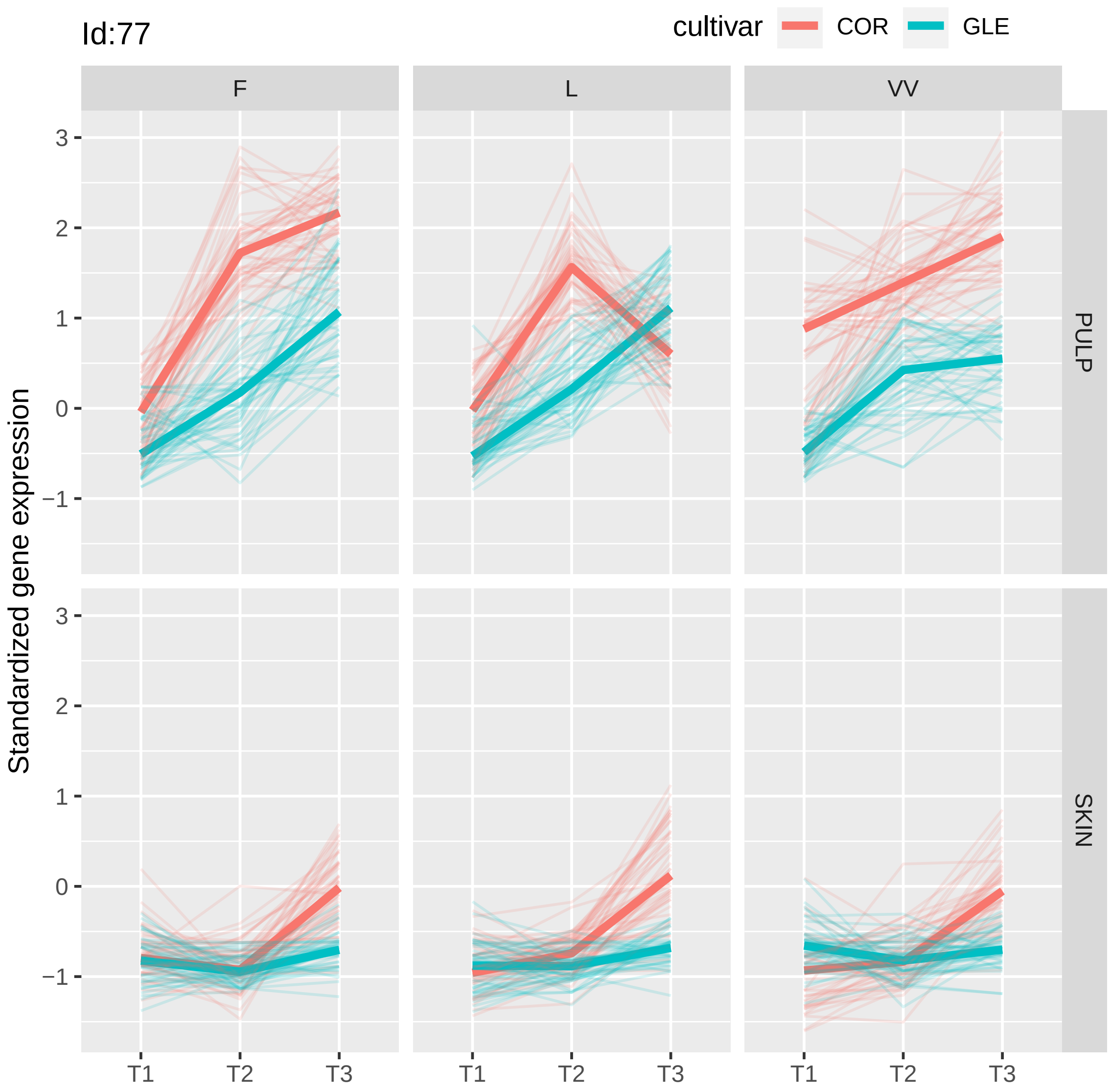
Id:77
cultivar
COR
GLE
F
L
VV
PULP
SKIN
Standardized gene expression
3
2
1
0
−1
T1
T2
T3

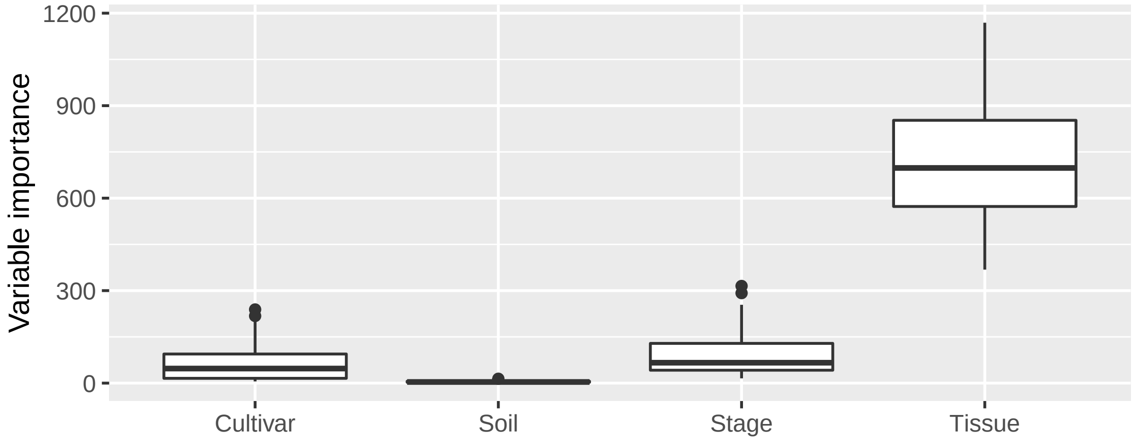
Variable importance
1200
900
600
300
0
Cultivar
Soil
Stage
Tissue

Cluster no. 72

```
## Number of genes in the cluster: 114
## Homogeneity Index:    0.79
## Variable importance for Stage:     Rank =  72  - Median =  61.93
## Variable importance for Cultivar:  Rank =   8  - Median =  681.2
## Variable importance for Tissue:    Rank =  71  - Median =  35.72
## Variable importance for Soil:      Rank =  29  - Median =   6.71
##
## Gene ID                Gene Annotation
## VIT_04s0008g00610  -  Zinc finger (CCCH-type) family protein
## VIT_18s0117g00380  -  R protein L6
## VIT_18s0001g04380  -  Unknown protein
## VIT_18s0041g01740  -  No hit
## VIT_17s0000g08010  -  Trehalose 6-phosphate synthase
## VIT_16s0098g01520  -  WD-40 repeat
## VIT_11s0016g00800  -  Unknown protein
## VIT_15s0045g01390  -  Protein kinase
## VIT_19s0015g02240  -  Dynamin-like 3
## VIT_03s0091g00310  -  Indole-3-acetic acid-amido synthetase GH3.8
## VIT_10s0042g00320  -  Myosin-like protein XIH
## VIT_09s0002g03170  -  CHUP1 (chloroplast unusual positioning 1)
## VIT_15s0021g01700  -  No hit
## VIT_10s0003g02130  -  Unknown
## VIT_00s0487g00010  -  Disease resistance protein (TIR-NBS-LRR class)
## VIT_13s0019g00780  -  Histone H4
## VIT_03s0088g00190  -  Brassinosteroid insensitive 1-associated receptor kinase 1
## VIT_14s0060g01970  -  F-box domain containing protein
## VIT_04s0044g00870  -  U-box domain containing protein
## VIT_03s0088g00180  -  No hit
## VIT_09s0018g01390  -  STE20/SPS1 proline-alanine-rich protein kinase
## VIT_12s0035g00740  -  No hit
## VIT_03s0091g00860  -  DNA polymerase kappa subunit
## VIT_17s0000g05980  -  Zinc finger protein / regulator of chromosome condensation (RCC1)
## VIT_02s0025g00890  -  Unknown
## VIT_13s0067g01730  -  Steroid 5alpha-reductase
## VIT_00s0285g00020  -  CBL-interacting protein kinase 23 (CIPK23)
## VIT_06s0061g01030  -  Aspartic Protease (VvAP13)
## VIT_00s0790g00010  -  Pentatricopeptide (PPR) repeat-containing protein
## VIT_15s0048g02830  -  Rac GTPase activating protein
## VIT_09s0018g01370  -  STE20/SPS1 proline-alanine-rich protein kinase
## VIT_14s0060g02120  -  Rad9
## VIT_04s0079g00370  -  Myosin-like protein XIA
## VIT_00s0184g00190  -  No hit
## VIT_00s1390g00010  -  DEAD  box  RNA helicase RH16
## VIT_16s0115g00220  -  Myosin-like protein XIA
## VIT_16s0039g02280  -  EIX receptor 2
## VIT_19s0177g00160  -  Phytochelatin synthetase
## VIT_18s0041g01110  -  Pentatricopeptide (PPR) repeat-containing
## VIT_13s0019g05170  -  Zinc finger (C2H2 type) family
## VIT_12s0034g01480  -  Disease resistance protein (NBS-LRR class) Rps1-k-2
## VIT_04s0044g01280  -  Beta-1,3-glucan synthase
## VIT_12s0134g00380  -  S-locus lectin protein kinase family
## VIT_18s0041g01650  -  R protein L6
## VIT_08s0032g00360  -  N-6 Adenine-specific DNA methylase
## VIT_13s0019g01140  -  R protein MLA10
```

```
## VIT_13s0139g00180  -  Disease resistance protein (NBS class)
## VIT_07s0005g04140  -  Unknown protein
## VIT_08s0007g00820  -  Proteinase inhibitor
## VIT_05s0062g01000  -  Aldo/keto reductase
## VIT_16s0039g01440  -  ATORC3/ORC3 (Origin recognition complex protein 3)
## VIT_05s0077g01490  -  Unknown
## VIT_08s0007g08020  -  No hit
## VIT_11s0016g04310  -  No hit
## VIT_16s0039g01430  -  ATORC3/ORC3 (Origin recognition complex protein 3)
## VIT_15s0021g01680  -  Pentatricopeptide repeat-containing protein
## VIT_08s0007g01130  -  No hit
## VIT_06s0004g01560  -  Unknown
## VIT_05s0029g00870  -  RPS4 (resistant to p. syringae 4)
## VIT_01s0011g01760  -  Lectin
## VIT_08s0056g00210  -  Cyclin-dependent kinase B1;2
## VIT_13s0067g01260  -  SAC3/GANP
## VIT_09s0002g04890  -  R protein  PRF  disease resistance protein
## VIT_12s0055g00660  -  Leucine-rich repeat protein kinase
## VIT_04s0008g06260  -  Exosome complex component RRP42
## VIT_00s0207g00060  -  Unknown protein
## VIT_16s0100g00070  -  Prohibitin 1
## VIT_04s0023g02540  -  Peroxisomal biogenesis factor 11 (PEX11C)
## VIT_03s0088g01050  -  Transposon protein, Mutator sub-class
## VIT_16s0039g01780  -  DNA replication complex GINS protein PSF2
## VIT_03s0097g00580  -  No hit
## ENSRNA049467803  -
## VIT_12s0035g00730  -  No hit
## VIT_15s0046g00440  -  Phosphatidylinositol 3- and 4-kinase / ubiquitin
## VIT_16s0098g00260  -  No hit
## VIT_11s0037g00980  -  Unknown
## VIT_04s0044g01230  -  Unknown
## VIT_12s0055g00640  -  Serine/theronine protein kinase 2
## VIT_11s0052g01300  -  Xyloglucan endotransglycosylase 6
## VIT_05s0077g00950  -  ATP citrate lyase a-subunit
## VIT_06s0004g04180  -  Zinc finger (C2H2 type) protein (ZAT11)
## VIT_15s0048g00080  -  Unknown
## VIT_06s0061g01210  -  Lysine histidine transporter 1
## VIT_13s0158g00200  -  R protein MLA10
## VIT_13s0019g03980  -  Transcription initiation factor TFIIF beta subunit
## VIT_04s0023g03900  -  Zinc finger (FYVE type)
## VIT_07s0104g00850  -  Pto kinase interactor
## VIT_13s0158g00170  -  R protein MLA10
## VIT_01s0026g00280  -  Trehalose 6-phosphate synthase
## VIT_01s0011g06280  -  Oxidoreductase
## VIT_07s0197g00220  -  R protein disease resistance protein
## VIT_15s0046g02800  -  R protein  PRF  disease resistance protein
## VIT_19s0015g02730  -  Glutathione S-transferase 25 GSTU25
## VIT_13s0067g00910  -  R protein MLA10
## VIT_11s0103g00750  -  CMP/dCMP deaminase, zinc-binding
## VIT_14s0068g00300  -  ABRC5
## VIT_05s0077g00120  -  DNA replication protein
## VIT_07s0104g00800  -  Indole-3-acetic acid-amido synthetase GH3.6
## VIT_10s0116g01260  -  Prefoldin
## VIT_07s0141g01010  -  No hit
```

```
## VIT_02s0154g00190  -  flavin-containing monooxygenase 3
## VIT_02s0025g04130  -  Rhomboid ATRBL2
## VIT_02s0241g00010  -  Pentatricopeptide (PPR) repeat-containing protein
## VIT_09s0002g04940  -  R protein  PRF  disease resistance protein
## VIT_16s0022g00390  -  No hit
## VIT_18s0122g00470  -  Spindle checkpoint protein Bub1b
## VIT_07s0255g00160  -  Unknown protein
## VIT_14s0108g00450  -  No hit
## VIT_03s0091g00580  -  Protein Mpv17
## VIT_00s0285g00050  -  No hit
## VIT_04s0008g02310  -  ABI3-interacting protein 2, AIP2
## VIT_15s0045g00770  -  Ribosomal protein S15
## VIT_18s0001g08560  -  Unknown protein
## VIT_08s0007g00650  -  No hit
```

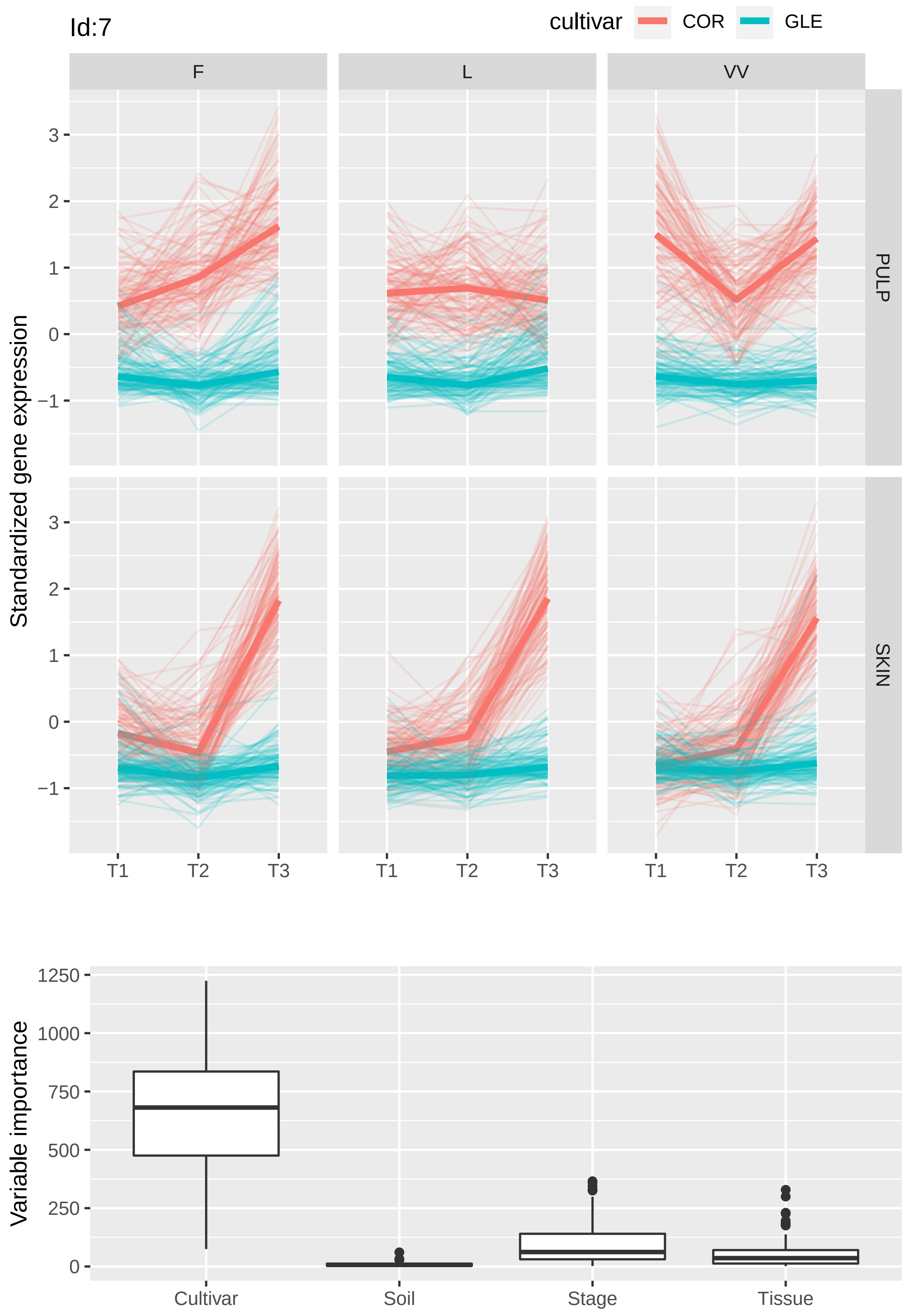
Id:7
cultivar
COR
GLE
F
L
VV
PULP
SKIN
Standardized gene expression
3
2
1
0
−1
T1
T2
T3
Variable importance
1250
1000
750
500
250
0
Cultivar
Soil
Stage
Tissue

Cluster no. 73

```
## Number of genes in the cluster: 54
## Homogeneity Index:    0.88
## Variable importance for Stage:     Rank =  73  - Median =  59.34
## Variable importance for Cultivar:  Rank =  76  - Median =  12.38
## Variable importance for Tissue:    Rank =  11  - Median =  752.5
## Variable importance for Soil:      Rank =  76  - Median =    4.1
##
## Gene ID               Gene Annotation
## VIT_14s0060g00250  -  Unknown
## VIT_08s0032g00310  -  Ubiquitin-conjugating enzyme E2 A
## VIT_16s0022g02100  -  Embryo-specific 3
## VIT_02s0025g02880  -  Subtilase
## VIT_01s0010g01850  -  Regulator of nonsense transcripts 1
## VIT_00s2015g00020  -  F-box family protein
## VIT_02s0087g00190  -  SH3 domain-containing protein 2
## VIT_18s0117g00070  -  R protein L6
## VIT_18s0001g12990  -  Anthranilate N-benzoyltransferase protein 1
## VIT_18s0089g01000  -  F-box family protein
## VIT_08s0007g01660  -  Chaperone protein dnaJ-related
## VIT_16s0050g00140  -  Heavy-metal-associated domain-containing protein
## VIT_18s0001g12390  -  Glucan endo-1,3-beta-glucosidase 7 precursor
## VIT_18s0001g08720  -  CW7 protein
## VIT_14s0068g01830  -  Unknown protein
## VIT_02s0025g01990  -  Zinc finger (C3HC4-type ring finger)
## VIT_13s0067g02420  -  No hit
## VIT_12s0057g01330  -  Zinc finger (C3HC4-type ring finger)
## VIT_08s0032g00150  -  F-box family protein
## VIT_06s0004g01270  -  Callose synthase catalytic subunit
## VIT_00s0662g00030  -  Dehydration Responsive Element-Binding Transcription Factor (VvDREB37)
## VIT_14s0006g01980  -  Zinc finger (C3HC4-type ring finger)
## VIT_01s0010g02100  -  Indeterminate(ID)-domain 5
## VIT_01s0137g00790  -  No hit
## VIT_18s0001g08270  -  Amino acid permease 6
## VIT_12s0028g01160  -  Transparent testa 12 protein
## VIT_00s0259g00110  -  ATFP3 (Arabidopsis thaliana farnesylated protein 3
## VIT_16s0050g01250  -  Heavy-metal-associated domain-containing protein
## VIT_03s0038g04450  -  Basic Leucine Zipper Transcription Factor (VvbZIP07)
## VIT_00s0299g00110  -  Glycoprotein homolog, related
## VIT_17s0000g06820  -  Zinc finger (C2H2 type) family
## VIT_06s0004g02720  -  Pleckstriny (PH) domain-containing protein
## VIT_08s0032g00140  -  F-box family protein
## VIT_15s0046g02230  -  Heavy-metal-associated domain-containing protein
## VIT_06s0061g01110  -  Lecithine cholesterol acyltransferase
## VIT_14s0030g00330  -  Sugar transporter ERD6-like 8
## VIT_08s0007g06140  -  Anthranilate phosphoribosyltransferase
## VIT_18s0001g09430  -  Tetratricopeptide repeat (TPR)-containing
## VIT_05s0029g01140  -  Sucrose-phosphate synthase
## VIT_02s0012g01410  -  TRN2 (TORNADO 2)
## VIT_09s0002g03690  -  Avr9/Cf-9 rapidly elicited protein 146
## VIT_11s0016g05720  -  Protein kinase APK1B
## VIT_00s0199g00070  -  Pleckstriny (PH) domain-containing protein
## VIT_16s0050g01850  -  AAA-type ATPase
## VIT_17s0000g08230  -  Unknown protein
## VIT_00s0407g00010  -  Pleckstriny (PH) domain-containing protein
```

```
## VIT_02s0025g03570  -  DNA binding / hydrolase, acting on ester bonds
## VIT_10s0003g01430  -  MEE62 (maternal effect embryo arrest 62)
## VIT_18s0089g00920  -  RNA binding motif protein 38
## VIT_02s0025g02250  -  Dof zinc finger protein DOF4.6
## VIT_04s0023g02820  -  putative MADS-box Apetala  3b (VviAP3b)
## VIT_09s0002g04120  -  Ethylene-responsive protein
## VIT_16s0098g01420  -  DOF affecting germination 1
## VIT_02s0025g03500  -  Unknown protein
```

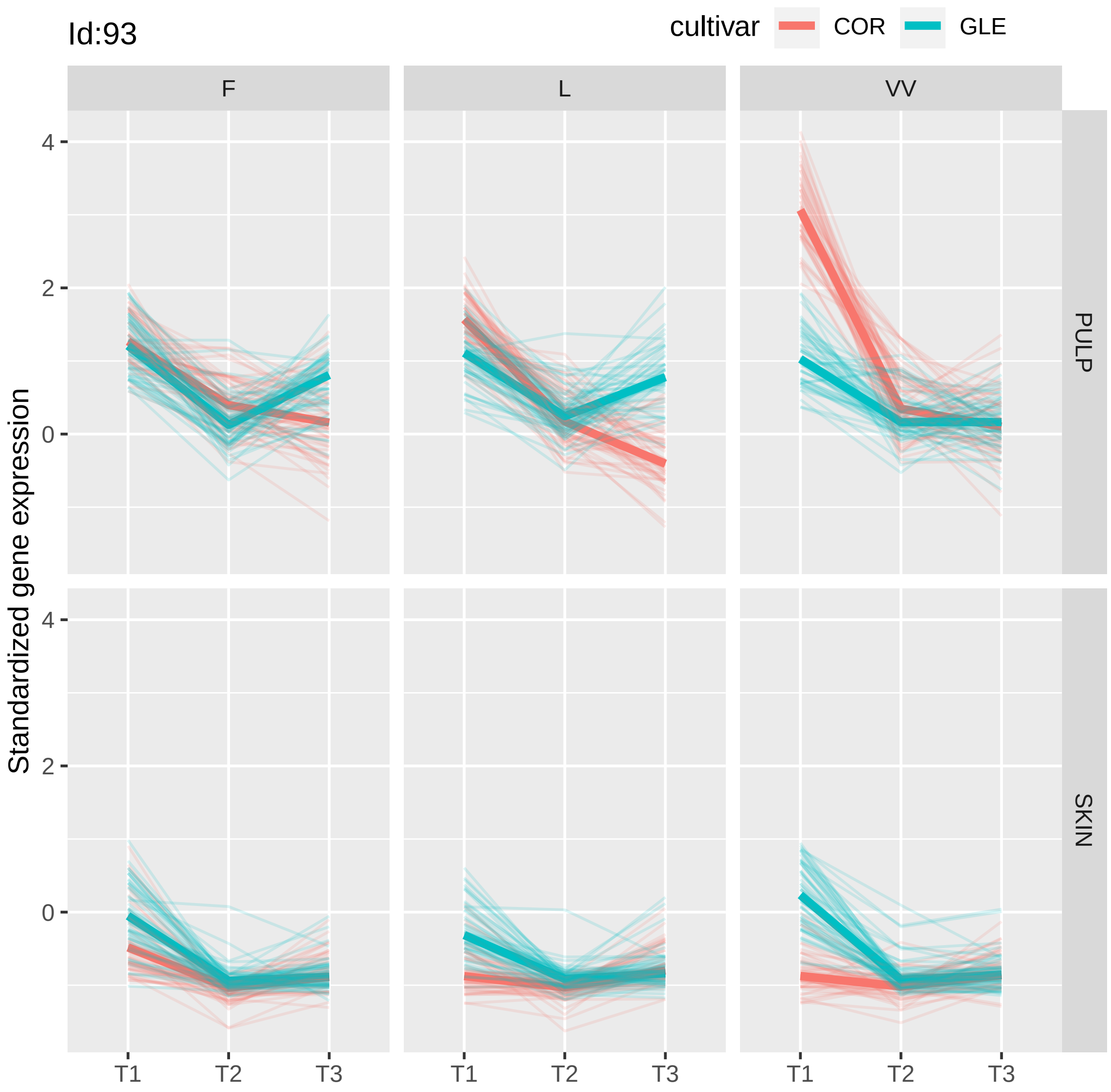
Id:93
cultivar
COR
GLE
F
L
VV
PULP
SKIN
Standardized gene expression
4
2
0
T1
T2
T3

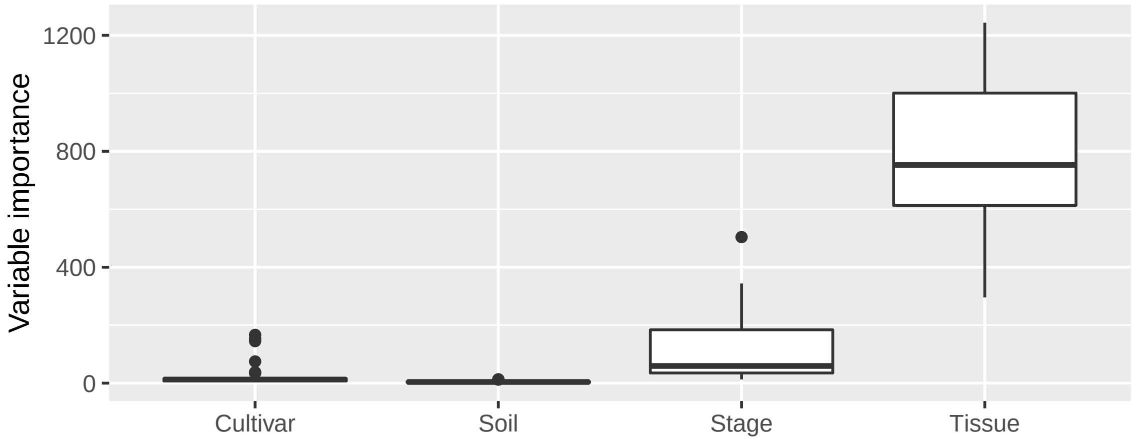
Variable importance
1200
800
400
0
Cultivar
Soil
Stage
Tissue

Cluster no. 74

```
## Number of genes in the cluster: 57
## Homogeneity Index:    0.84
## Variable importance for Stage:     Rank =  74  - Median =   56.3
## Variable importance for Cultivar:  Rank =  82  - Median =  11.05
## Variable importance for Tissue:    Rank =  10  - Median =  802.9
## Variable importance for Soil:      Rank =  84  - Median =   3.64
##
## Gene ID               Gene Annotation
## VIT_19s0090g00960  -  Unknown
## VIT_00s0198g00160  -  AAA-type ATPase
## VIT_00s0229g00060  -  Nodulin MtN21 family
## VIT_15s0046g01540  -  F-box family protein
## VIT_09s0054g00610  -  F-box domain containing protein
## VIT_01s0146g00270  -  Delta3,5-delta2,4-dienoyl-CoA isomerase
## VIT_08s0007g02340  -  Exocyst subunit EXO70 A2
## VIT_14s0171g00090  -  Uracil phosphoribosyltransferase.
## VIT_14s0060g01530  -  DNA polymerase III subunit epsilon
## VIT_18s0089g00410  -  Sucrose-phosphate synthase 1
## VIT_13s0019g00340  -  Glutaredoxin
## VIT_18s0001g12120  -  Basic Leucine Zipper Transcription Factor (VvbZIP46)
## VIT_07s0005g00290  -  Universal stress protein (USP) family protein
## VIT_01s0127g00710  -  Mov34 STAM-binding protein
## VIT_07s0005g01570  -  No hit
## VIT_19s0085g01200  -  CTP synthase
## VIT_01s0026g01400  -  Unknown protein
## VIT_18s0001g13150  -  BEL1 homeotic protein 3
## VIT_04s0023g03780  -  Cell cycle checkpoint protein RAD17 (AtRAD17)
## VIT_14s0108g00840  -  Unknown protein
## VIT_01s0011g01420  -  UDP-N-acetylglucosamine pyrophosphorylase
## VIT_13s0019g05370  -  Histone H2B
## VIT_08s0007g07320  -  Unknown protein
## VIT_00s0186g00020  -  SUM2 (small ubiquitin-like modifier 2)
## VIT_11s0016g04200  -  MEE55 (maternal effect embryo arrest 55)
## VIT_10s0071g01030  -  Transducin family protein / WD-40 repeat
## VIT_11s0016g05710  -  UDP-glucuronic acid/UDP-N-acetylgalactosamine transporter
## VIT_13s0101g00460  -  No hit
## VIT_04s0008g00520  -  6-phosphofructokinase
## VIT_03s0063g00200  -  Histidinol-phosphatase
## VIT_17s0000g01870  -  Indeterminate(ID)-domain 16
## VIT_13s0064g00540  -  R protein MLA10
## VIT_18s0041g01340  -  R protein L6
## VIT_02s0087g00930  -  (9,10) (9`,10`) cleavage dioxygenase (CCD4) (VvCCD4b)
## VIT_06s0004g01640  -  UDP-glucoronosyl and UDP-glucosyl transferase
## VIT_09s0002g05940  -  Ras GTP-binding protein (RAN3)
## VIT_14s0060g02270  -  Translationally-controlled tumor protein
## VIT_14s0060g00170  -  Nuclear pore complex protein Nup98-Nup96
## VIT_10s0003g02720  -  Beta-1,3 glucanase
## VIT_13s0067g03550  -  Centromere protein
## VIT_19s0015g01430  -  BTB/POZ domain-containing protein
## VIT_01s0113g00500  -  Argonaute
## VIT_08s0007g00180  -  OBP3 (OBF-binding protein 3)
## VIT_05s0049g01410  -  Phosphate transporter 1
## VIT_13s0067g02050  -  Unknown protein
## VIT_07s0191g00250  -  Exo-1,3-beta-glucanase
```

```
## VIT_00s2466g00010  -  Exo-1,3-beta-glucanase
## VIT_08s0040g01230  -  Auxin transport protein (PIN3)
## VIT_02s0241g00020  -  Unknown
## VIT_01s0011g06400  -  Indeterminate(ID)-domain 16
## VIT_01s0150g00360  -  Unknown
## VIT_17s0000g02420  -  Auxin transport protein (PIN3)
## VIT_17s0000g01210  -  Unknown protein
## VIT_11s0118g00390  -  Oxidoreductase, 2OG-Fe(II) oxygenase
## VIT_14s0060g00160  -  Nuclear pore complex protein Nup98-Nup96
## VIT_07s0005g01710  -  WRKY Transcription Factor (VvWRKY19)
## VIT_08s0056g00730  -  SEC14 cytosolic factor
```

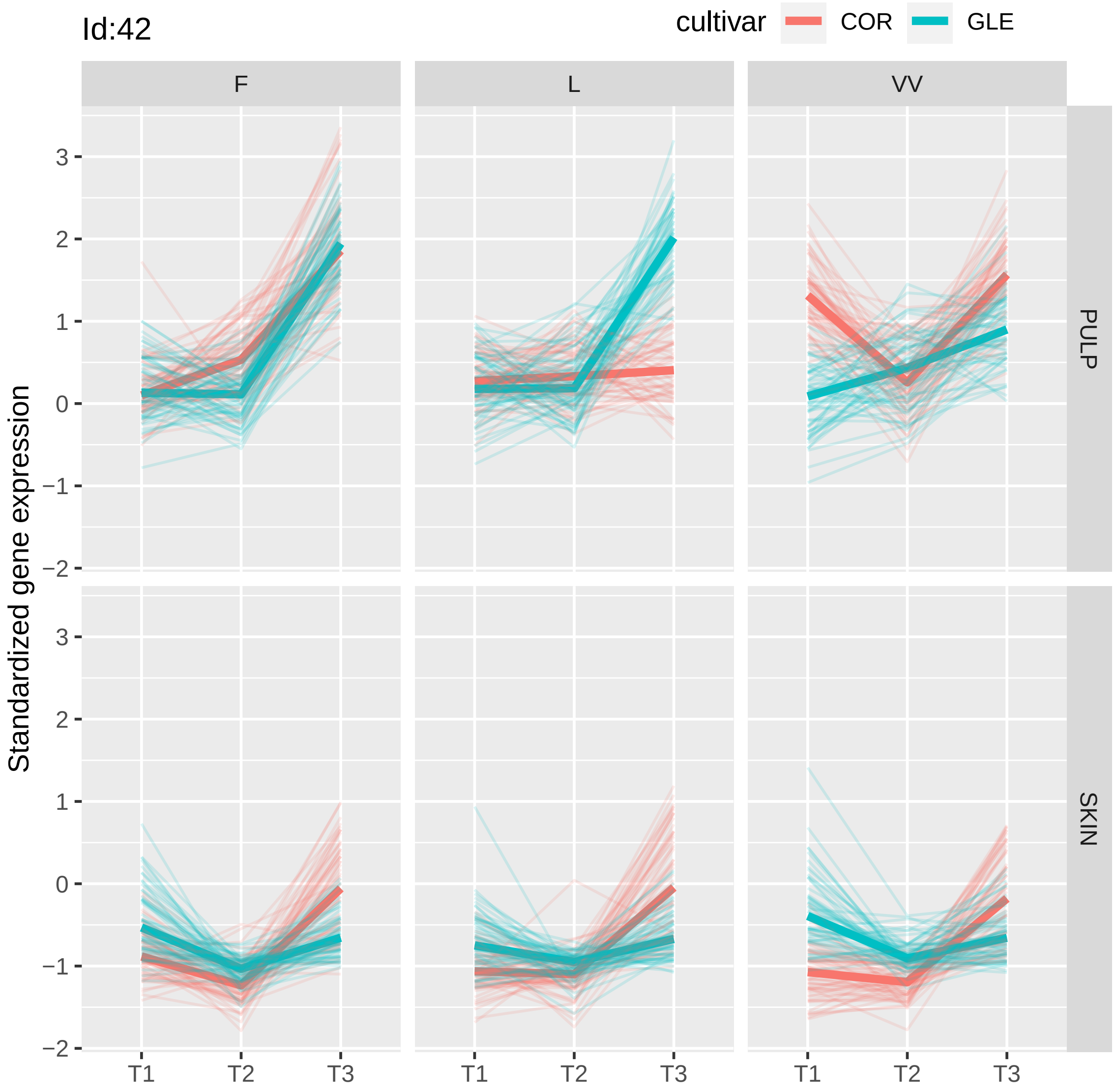

Id:42
cultivar
COR
GLE
F
L
VV
PULP
SKIN
Standardized gene expression
3
2
1
0
−1
−2
T1
T2
T3

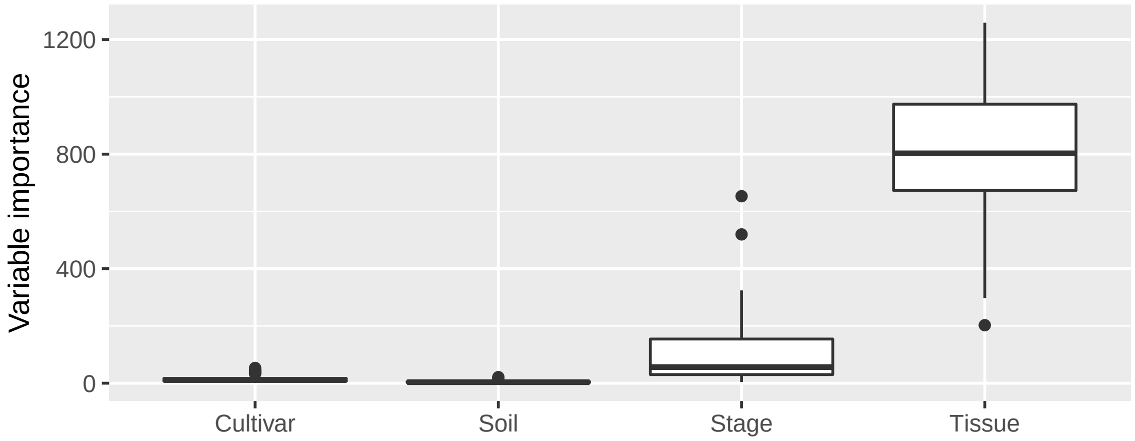

Variable importance
1200
800
400
0
Cultivar
Soil
Stage
Tissue

Cluster no. 75

```
## Number of genes in the cluster: 43
## Homogeneity Index:    0.72
## Variable importance for Stage:     Rank =  75  - Median =  56.23
## Variable importance for Cultivar:  Rank =  34  - Median =  93.66
## Variable importance for Tissue:    Rank =  20  - Median =  537.2
## Variable importance for Soil:      Rank =  56  - Median =   5.42
##
## Gene ID               Gene Annotation
## VIT_14s0006g01040  -  No hit
## VIT_18s0001g08840  -  Serine carboxypeptidase S10
## VIT_16s0039g02400  -  Protein kinase FU (FUSED)
## VIT_18s0001g08910  -  No hit
## VIT_10s0071g00500  -  GRAM domain-containing protein / ABA-responsive
## VIT_14s0060g02440  -  Indeterminate(ID)-domain 2
## VIT_07s0104g00270  -  Isopentenyltransferase 5
## VIT_16s0098g01050  -  RNA binding / nucleic acid binding / nucleotide binding [Arabidopsis t
## VIT_13s0067g03630  -  No hit
## VIT_16s0098g00640  -  Thaumatin
## VIT_18s0001g03430  -  Flavonol synthase
## VIT_12s0055g00370  -  Harpin-induced protein
## VIT_16s0115g00200  -  DNA-directed RNA Polymerase II subunit K
## VIT_15s0021g00270  -  Ent-kaurenoic acid oxidase
## VIT_10s0071g00060  -  No hit
## VIT_16s0050g00850  -  C2 domain-containing protein
## VIT_17s0000g03080  -  R protein MLA10
## VIT_18s0001g11120  -  Ternary complex factor MIP1
## VIT_00s0259g00210  -  DNA-binding protein
## VIT_19s0027g00790  -  Transglycosylase SLT domain containing protein
## VIT_11s0016g00330  -  Pectinesterase family
## VIT_03s0091g00680  -  NPY2 (naked pins in yuc mutants 2)
## VIT_03s0091g01040  -  No hit
## VIT_02s0087g00400  -  Pectinacetylesterase
## VIT_07s0031g03040  -  Calmodulin-binding protein
## VIT_01s0010g01840  -  GEM-like protein 5
## VIT_04s0023g03530  -  No hit
## VIT_07s0031g03030  -  Calmodulin-binding protein
## VIT_04s0023g03540  -  Thaumatin SCUTL1
## VIT_12s0034g02370  -  Unknown protein
## VIT_07s0151g00450  -  basic helix-loop-helix (bHLH) family
## VIT_14s0006g00180  -  Aspartyl protease
## VIT_13s0019g02460  -  Proton-dependent oligopeptide transport (POT) family protein
## VIT_15s0048g01270  -  putative MADS-box Agamous-like 6a (VviAGL6a)
## VIT_08s0040g00800  -  Nodulin MtN21
## VIT_00s0956g00030  -  DNA-binding protein
## VIT_01s0137g00400  -  Unknown protein
## VIT_11s0016g03540  -  Auxin-responsive protein IAA27
## VIT_08s0040g02680  -  Unknown protein
## VIT_10s0071g00770  -  Aspartic Protease (VvAP29)
## VIT_04s0023g02360  -  Chaperone BCS1 mitochondrial
## VIT_10s0116g00590  -  Pectinesterase family
## VIT_04s0023g01480  -  Dimethylaniline monooxygenase, N-oxide-forming
```

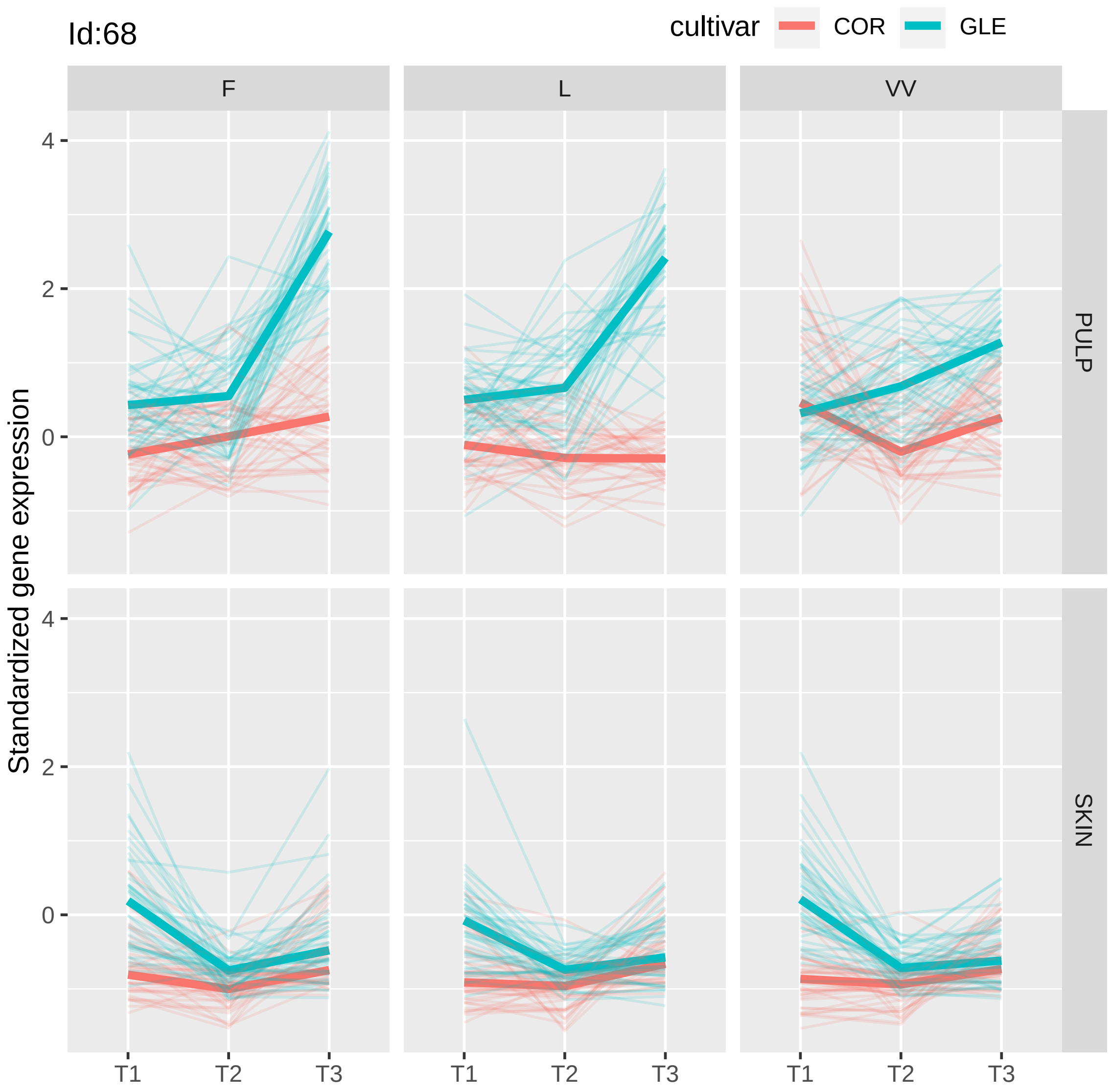
Id:68
cultivar
COR
GLE
F
L
VV
PULP
SKIN
Standardized gene expression
4
2
0
T1
T2
T3

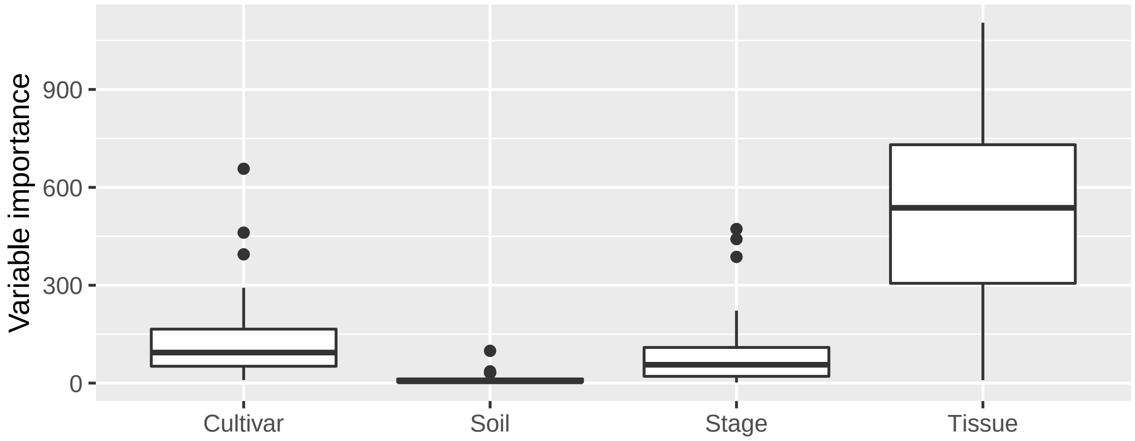
Variable importance
900
600
300
0
Cultivar
Soil
Stage
Tissue

Cluster no. 76

```
## Number of genes in the cluster: 42
## Homogeneity Index:     0.9
## Variable importance for Stage:     Rank =  76  - Median =  56.14
## Variable importance for Cultivar:  Rank =  46  - Median =  50.32
## Variable importance for Tissue:    Rank =  12  - Median =  750.1
## Variable importance for Soil:      Rank =  97  - Median =   2.83
##
## Gene ID               Gene Annotation
## VIT_12s0028g01720  -  Unknown
## VIT_09s0002g03260  -  No hit
## VIT_00s0304g00080  -  Trehalose-6-phosphate phosphatase
## VIT_08s0040g00480  -  Unknown
## VIT_17s0000g03670  -  Unknown protein
## VIT_06s0004g01050  -  Calcineurin phosphoesterase
## VIT_13s0019g02430  -  LIM domain containing protein
## VIT_05s0049g00570  -  Unknown
## VIT_18s0001g15410  -  Alcohol dehydrogenase 1
## VIT_08s0007g05720  -  No hit
## VIT_00s0662g00040  -  Dehydration Responsive Element-Binding Transcription Factor (VvDREB38)
## VIT_00s0601g00040  -  Unknown protein
## VIT_14s0083g01050  -  putative MADS-box sepallata 1 (VviSEP1)
## VIT_09s0002g03250  -  Profilin 4 (PRO4) (PFN4)
## VIT_08s0007g04740  -  RAB GTPase RAB_ALPHA
## VIT_04s0069g00250  -  Unknown
## VIT_12s0028g01570  -  Zinc finger (C3HC4-type ring finger)
## VIT_03s0091g00410  -  Unknown protein
## VIT_18s0001g05370  -  Unknown protein
## VIT_08s0040g03360  -  Cofilin
## VIT_05s0077g01700  -  Copper-binding family protein
## VIT_01s0026g01250  -  Unknown protein
## VIT_07s0104g00640  -  No hit
## VIT_05s0049g00850  -  Retrotransposon protein, Unclassified
## VIT_17s0000g06960  -  UDP-glucose 6-dehydrogenase
## VIT_02s0025g01790  -  Cellulose synthase CSLG3
## VIT_19s0138g00120  -  1,3-beta-glucan synthase
## VIT_18s0089g00980  -  No hit
## VIT_08s0040g02700  -  Heavy-metal-associated domain-containing protein
## VIT_19s0014g03290  -  NAC domain-containing protein (VvNAC17)
## VIT_13s0320g00070  -  Myosin-like protein XIF
## VIT_19s0015g00320  -  KCO1 (two pore K channel)
## VIT_03s0063g02120  -  Minor allergen
## VIT_06s0004g01180  -  Peroxidase
## VIT_00s0324g00030  -  Unknown
## VIT_06s0080g00970  -  NAC domain-containing protein (VvNAC67)
## VIT_00s0259g00080  -  Metal ion binding
## VIT_19s0014g05180  -  RPS2 (resistant to p. syringae 2)
## VIT_18s0001g06340  -  RPS4 (resistant to p. syringae 4)
## VIT_14s0066g01440  -  Homeodomain leucine zipper protein HB-1 (VvATHB-4)
## VIT_01s0010g02210  -  FRK1 (FLG22-induced receptor-like kinase 1)
## VIT_04s0008g03250  -  Homeobox gene 8
```

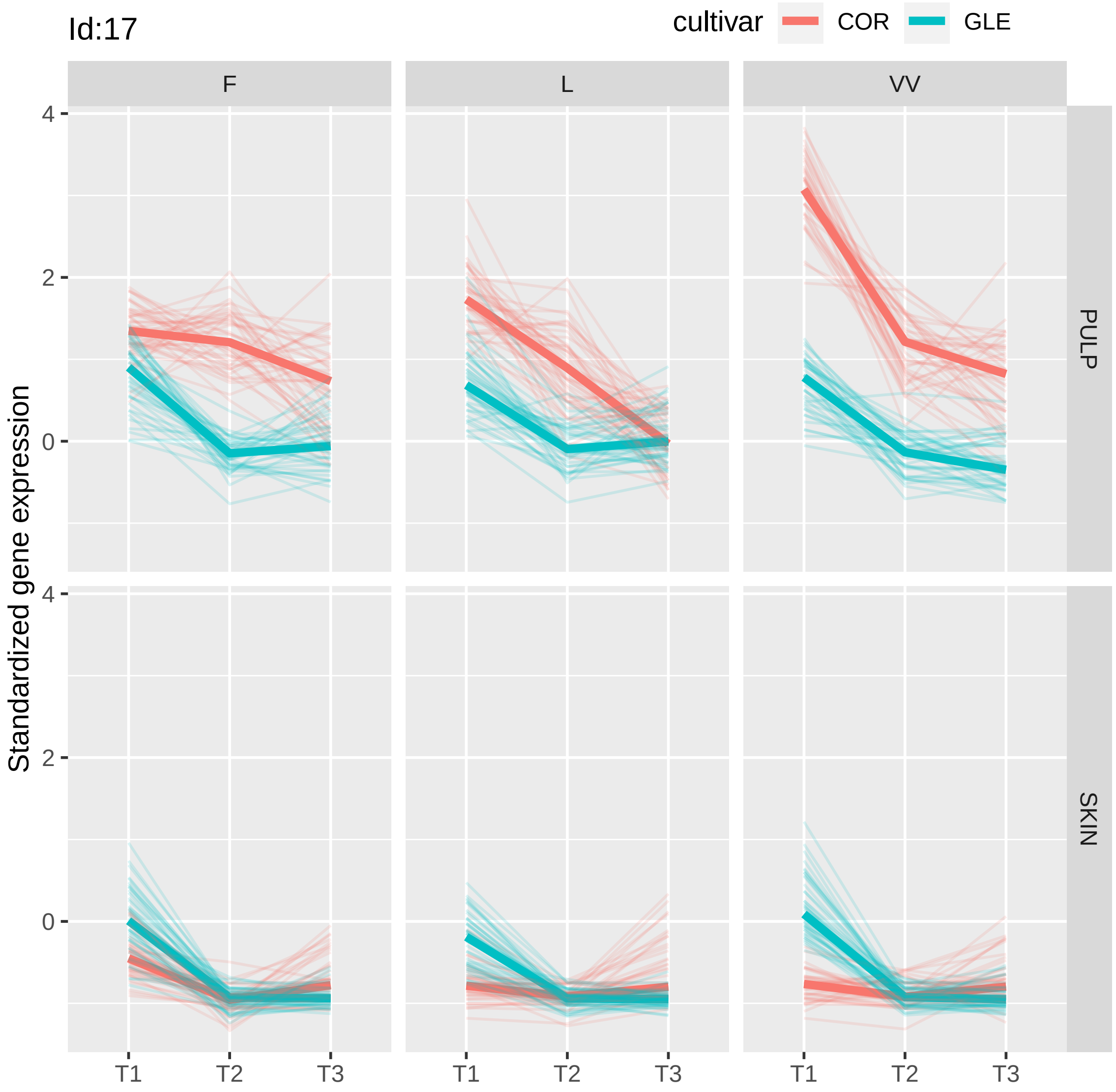
Id:17
cultivar
COR
GLE
F
L
VV
PULP
SKIN
Standardized gene expression
4
2
0
T1
T2
T3

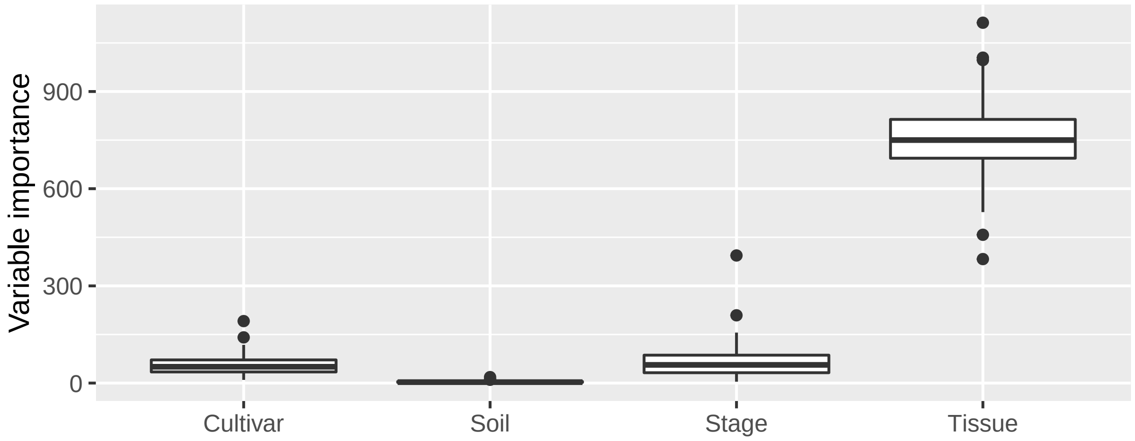
Variable importance
900
600
300
0
Cultivar
Soil
Stage
Tissue

Cluster no. 77

```
## Number of genes in the cluster: 64
## Homogeneity Index:    0.92
## Variable importance for Stage:     Rank =  77  - Median =  55.77
## Variable importance for Cultivar:  Rank =  42  - Median =  56.95
## Variable importance for Tissue:    Rank =  13  - Median =  742.5
## Variable importance for Soil:      Rank =  77  - Median =    4.1
##
## Gene ID                Gene Annotation
## VIT_07s0104g00730  -  Unknown protein
## VIT_10s0003g00890  -  Magnesium-chelatase subunit chlI, chloroplast precursor
## VIT_00s0265g00010  -  Phosphoglycerate mutase
## VIT_07s0151g00860  -  Hydrolase, alpha/beta fold
## VIT_16s0100g00410  -  TraB protein
## VIT_13s0064g00200  -  Unknown protein
## VIT_13s0019g05250  -  Malate dehydrogenase [NADP], chloroplast precursor (NADP-MDH)
## VIT_17s0000g06450  -  Ribulose-phosphate 3-epimerase, chloroplast precursor
## VIT_07s0104g00510  -  MRNA-binding protein precursor
## VIT_13s0084g00160  -  Proline-rich family protein
## VIT_04s0008g05490  -  L-ascorbate peroxidase.
## VIT_13s0067g02450  -  Uroporphyrinogen decarboxylase
## VIT_17s0000g09910  -  Haloacid dehalogenase hydrolase
## VIT_12s0028g01100  -  Aldo/keto reductase
## VIT_14s0060g00820  -  3-beta hydroxysteroid dehydrogenase
## VIT_00s0391g00020  -  Protein cbbY
## VIT_02s0109g00080  -  Phosphoribulokinase
## VIT_00s0533g00020  -  Zeaxanthin epoxidase, chloroplast precursor
## VIT_06s0004g06170  -  Thylakoid soluble phosphoprotein
## VIT_15s0024g00390  -  Ribosomal protein L3
## VIT_05s0049g00420  -  E8 protein
## VIT_01s0026g02480  -  CP12-2
## VIT_03s0038g00740  -  GC1 (GIANT chloroplast 1)
## VIT_09s0018g01780  -  ACT domain repeat 1 (ACR1) uridylyltransferase
## VIT_15s0024g00640  -  SIGB (SIGMA factor B)
## VIT_00s0454g00030  -  Subtilisin protease
## VIT_01s0244g00030  -  ACP4 (Acyl carrier protein 4)
## VIT_11s0016g05570  -  Thylakoid lumenal 16.5 kDa protein, chloroplast precursor
## VIT_18s0001g11590  -  Dual-specific kinase DSK1
## VIT_01s0026g02680  -  Oxygen evolving enhancer 3 (PsbQ)
## VIT_03s0088g01190  -  Malate dehydrogenase, glyoxysomal precursor
## VIT_19s0093g00510  -  S-2-hydroxy-acid oxidase, peroxisomal
## VIT_18s0122g00960  -  Glyceraldehyde-3-phosphate dehydrogenase B, chloroplast precursor
## VIT_13s0064g00190  -  RNA polymerase sigma subunit SigD
## VIT_11s0016g03700  -  Pentatricopeptide (PPR) repeat-containing protein
## VIT_01s0011g03590  -  Ribosomal protein L15, chloroplast (CL15) 50S
## VIT_19s0085g01190  -  ABC Transporter (VvPMP1 - VvABCD1)
## VIT_13s0019g03350  -  Sedoheptulose-1,7-bisphosphatase (SBPase), Chloroplast
## VIT_06s0009g03740  -  Serine-glyoxylate aminotransferase
## VIT_18s0001g10510  -  Thioredoxin family
## VIT_10s0116g01740  -  ATP synthase gamma chain 1t (ATPC1)
## VIT_08s0007g08290  -  GTP-binding protein TypA/BipA
## VIT_10s0003g00300  -  Acclimation of photosynthesis to environment
## VIT_01s0011g04190  -  Unknown
## VIT_18s0001g02700  -  Chlorophyll a oxygenase (CAO)
## VIT_14s0060g01030  -  Haloacid dehalogenase hydrolase
```

```
## VIT_13s0064g00170  -  Ribosomal protein L1
## VIT_06s0080g00030  -  Aldo/keto reductase
## VIT_01s0010g01140  -  Unknown
## VIT_01s0011g02150  -  Photosystem II stability/assembly factor, chloroplast (HCF136)
## VIT_08s0007g02970  -  Unknown protein
## VIT_04s0023g00720  -  Unknown protein
## VIT_00s0207g00030  -  No hit
## VIT_16s0098g01650  -  Protein phosphatase 2C PPH1
## VIT_04s0008g06690  -  Proton gradient regulation 3
## VIT_04s0023g03510  -  Ferredoxin:nadp+ Oxidoreductase PETH
## VIT_18s0001g15650  -  Pathogenesis related protein
## VIT_09s0002g05200  -  Phosphoribulokinase/uridine kinase
## VIT_02s0025g01470  -  Unknown protein
## VIT_00s0323g00040  -  Unknown protein
## VIT_11s0016g04250  -  HCF101 (high-chlorophyll-fluorescence 101)
## VIT_01s0011g03010  -  Serine/threonine-protein kinase SNT7, chloroplast precursor
## VIT_06s0061g01340  -  Phosphoglycerate mutase
## VIT_11s0065g00170  -  Squamosa promoter-binding protein (VvSBP10)
```

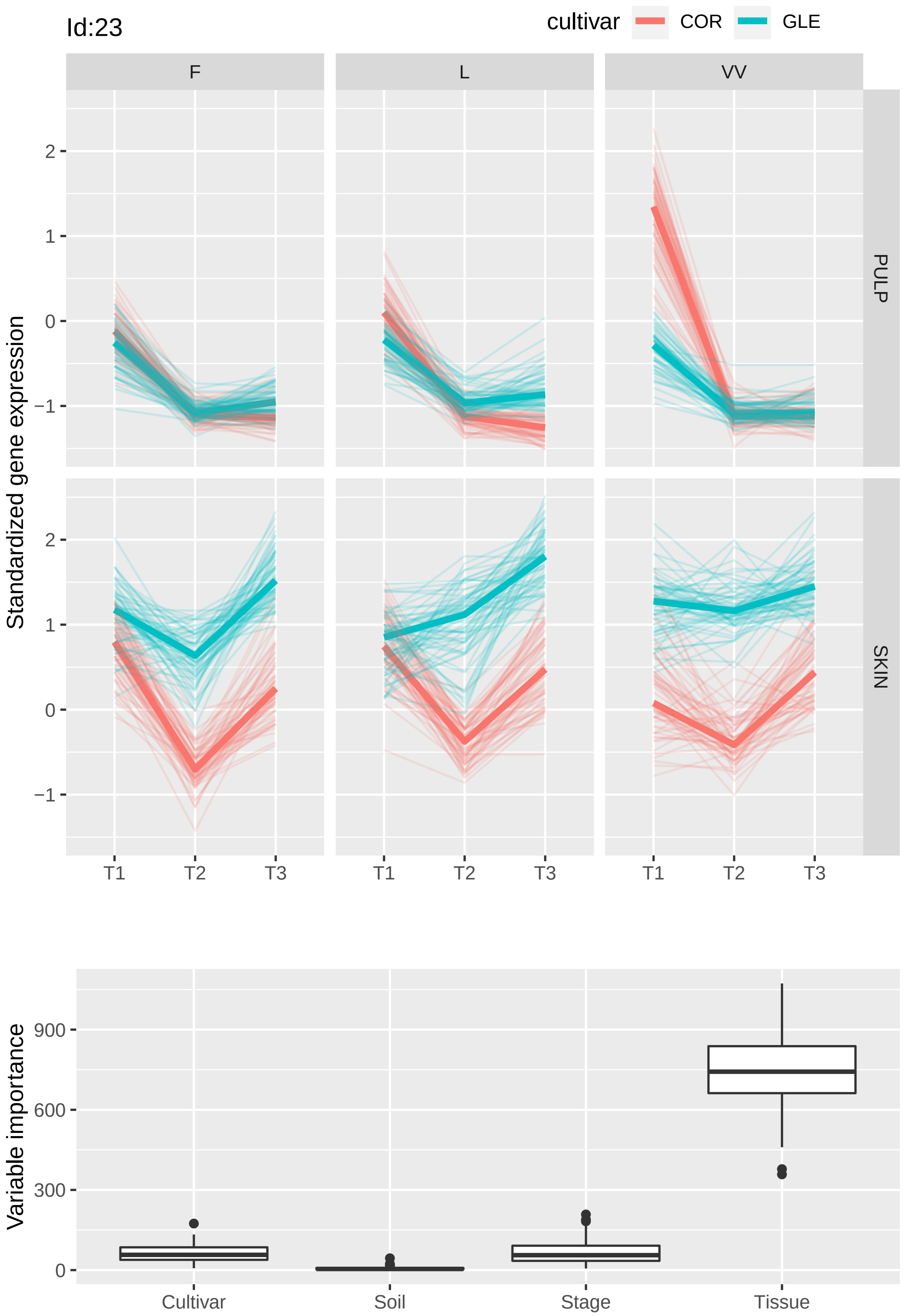
Id:23
cultivar
COR
GLE
F
L
VV
PULP
SKIN
Standardized gene expression
2
1
0
−1
T1
T2
T3
Variable importance
900
600
300
0
Cultivar
Soil
Stage
Tissue

Cluster no. 78

```
## Number of genes in the cluster: 83
## Homogeneity Index:    0.75
## Variable importance for Stage:     Rank =  78  - Median =  52.63
## Variable importance for Cultivar:  Rank =   5  - Median =  843.6
## Variable importance for Tissue:    Rank =  85  - Median =  17.87
## Variable importance for Soil:      Rank =  86  - Median =   3.57
##
## Gene ID                Gene Annotation
## VIT_04s0008g04620  -  Expansin family protein (EXPR3)
## VIT_09s0018g02120  -  No hit
## VIT_04s0008g02840  -  Avirulence induced gene (AIG)
## VIT_08s0007g05480  -  Kinesin motor protein
## VIT_16s0050g01190  -  Metal ion binding
## VIT_14s0006g02170  -  Jasmonate O-methyltransferase
## VIT_17s0000g00940  -  F-box protein
## VIT_04s0008g02370  -  Pentatricopeptide (PPR) repeat-containing protein
## VIT_15s0021g02650  -  Lipase GDSL
## VIT_08s0040g01610  -  2-phosphoglycerate kinase
## VIT_09s0002g04570  -  Unknown
## VIT_00s0294g00080  -  Receptor kinase homolog LRK10
## VIT_12s0034g01350  -  R protein L6
## VIT_09s0002g06360  -  Unknown protein
## VIT_14s0030g00970  -  Oxidoreductase, zinc-binding dehydrogenase family
## VIT_13s0064g01380  -  Anthranilate phosphoribosyltransferase
## VIT_04s0008g02580  -  V-type H+-transporting ATPase subunit A
## VIT_16s0148g00020  -  Ser/Thr receptor-like kinase1
## VIT_15s0046g01230  -  Leaf senescence protein-like
## VIT_13s0019g02890  -  Glucosyltransferase-2
## VIT_04s0008g04020  -  RD22 [Vitis vinifera]
## VIT_17s0053g00990  -  Expansin  (VvEXPA18)
## VIT_05s0062g01070  -  Receptor protein kinase
## VIT_10s0071g00550  -  GRAM domain-containing protein / ABA-responsive
## VIT_01s0011g01750  -  No hit
## VIT_02s0012g01270  -  Abscisic acid receptor PYL1  RCAR12
## VIT_15s0046g00080  -  Yippee
## VIT_13s0019g04890  -  Shugoshin-1
## VIT_03s0091g00960  -  No hit
## VIT_00s0260g00070  -  FK506-binding protein genes family (VvFKBPa)
## VIT_16s0050g02700  -  Receptor kinase homolog LRK10
## VIT_16s0148g00110  -  Receptor kinase homolog LRK10
## VIT_15s0024g01630  -  Glutathione S-transferase 8 GSTU8
## VIT_15s0024g01650  -  Glutathione S-transferase 8 GSTU8
## VIT_09s0002g02840  -  Light repressible receptor protein kinase
## VIT_12s0034g02270  -  Cc-nbs-lrr resistance protein
## VIT_07s0095g00680  -  Serine/threonine kinase BRLK
## VIT_16s0148g00010  -  Receptor kinase homolog LRK10
## VIT_18s0122g00630  -  Cinnamoyl-CoA reductase
## VIT_19s0014g03610  -  Unknown
## VIT_13s0067g03590  -  R protein MLA10
## VIT_00s0425g00030  -  Receptor serine/threonine kinase
## VIT_05s0049g00290  -  No hit
## VIT_15s0046g00400  -  Seven in absentia SINA4
## VIT_11s0052g01360  -  No hit
## VIT_16s0148g00070  -  Receptor-like protein kinase
```

```
## VIT_16s0050g00190  -  DTA2 (downstream target of AGL15 2)
## VIT_13s0047g00720  -  Unknown
## VIT_15s0046g00870  -  Cleavage and polyadenylation specificity factor (CPSF) A subunit
## VIT_19s0090g00100  -  Unknown protein
## VIT_06s0004g02300  -  Calcium Dependent Protein Kinase (VvCPK5)
## VIT_12s0034g01100  -  Mucin-related
## VIT_02s0033g01260  -  No hit
## VIT_06s0080g00530  -  Aspartyl-tRNA synthetase
## VIT_16s0098g00090  -  Zinc finger (C3HC4-type ring finger)
## VIT_01s0011g00060  -  No hit
## VIT_00s0425g00040  -  Receptor serine/threonine kinase
## VIT_00s0669g00010  -  E3 ubiquitin-protein ligase CHIP
## VIT_19s0014g00900  -  Brassinosteroid Signaling positive regulator (BZR1)
## VIT_00s0582g00020  -  Acyl-coenzyme A thioesterase 9.
## VIT_18s0001g13190  -  MAPK (MPK9)
## VIT_16s0148g00120  -  Receptor kinase homolog LRK10
## VIT_13s0101g00290  -  No hit
## VIT_16s0039g01260  -  Ser/Thr receptor-like kinase1
## VIT_01s0146g00010  -  Calreticulin
## VIT_00s0449g00010  -  Membrane bound O-acyl transferase (MBOAT)
## VIT_09s0002g02440  -  ABC Transporter (VvMRP9 - VvABCC9)
## VIT_09s0002g07710  -  Disease resistance protein
## VIT_11s0206g00100  -  Unknown protein
## VIT_09s0018g00620  -  Co-chaperone-curved DNA binding protein A
## VIT_07s0005g02670  -  3-N-debenzoyl-2-deoxytaxol N-benzoyltransferase
## VIT_13s0047g00550  -  Cinnamyl alcohol dehydrogenase
## VIT_12s0035g00420  -  Disease resistance protein
## VIT_16s0050g02710  -  RPK1 (receptor-like protein kinase 1)
## VIT_00s0199g00270  -  Unknown protein
## VIT_13s0047g00620  -  No hit
## VIT_18s0041g02390  -  Abscisic acid 8` hydroxylase (CYP707A2) (VvA8H-CYP707A2.5)
## VIT_08s0007g02720  -  Secoisolariciresinol dehydrogenase
## VIT_00s0400g00040  -  Dynamin-2A
## VIT_00s0160g00310  -  Disease resistance protein (TIR-NBS-LRR class
## VIT_12s0034g00380  -  R protein L6
## VIT_11s0065g01080  -  DEAD/DEAH box helicase (RH10)
## VIT_07s0005g06690  -  Transcription initiation factor TFIIF beta subunit (TFIIF-beta)
```

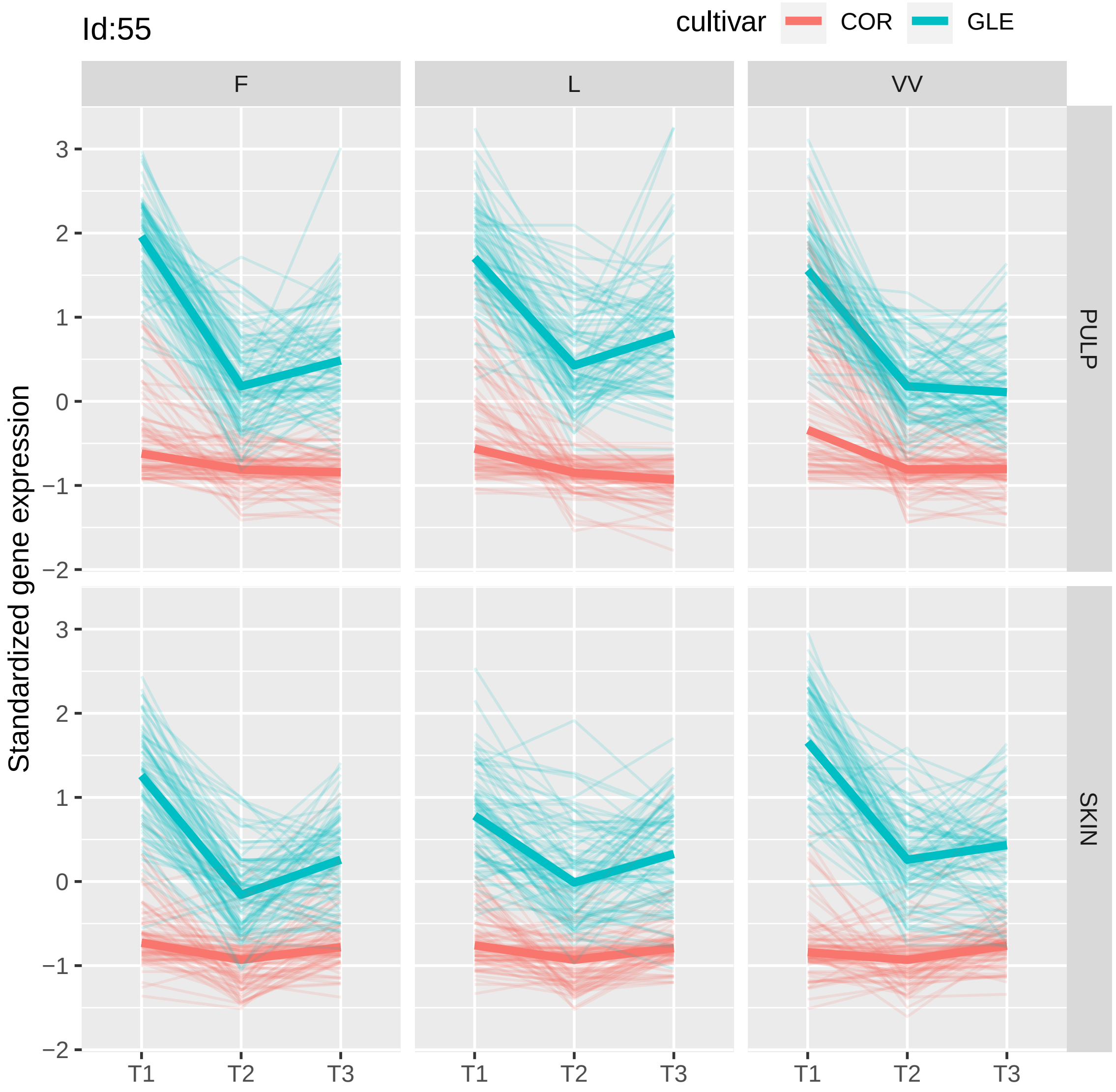
Id:55
cultivar
COR
GLE
F
L
VV
PULP
SKIN
Standardized gene expression
3
2
1
0
−1
−2
T1
T2
T3

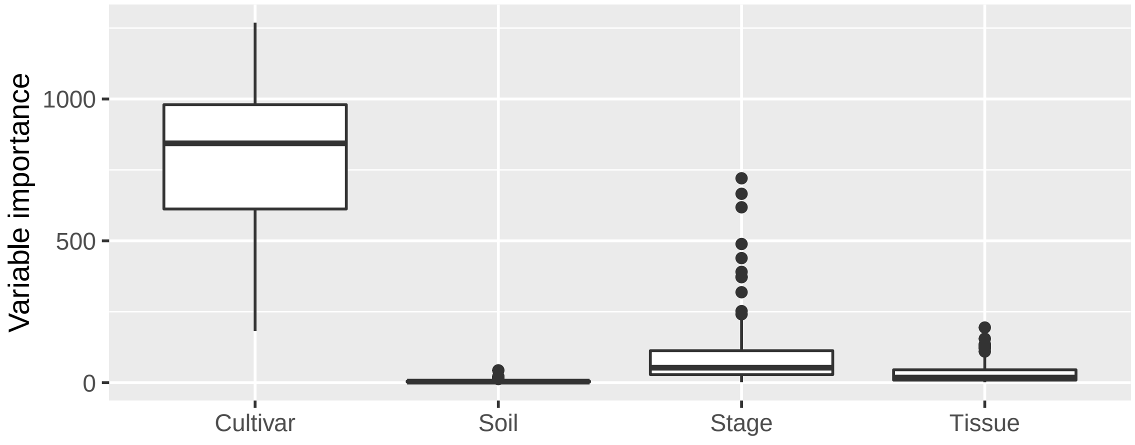
Variable importance
1000
500
0
Cultivar
Soil
Stage
Tissue

Cluster no. 79

```
## Number of genes in the cluster: 72
## Homogeneity Index:    0.71
## Variable importance for Stage:     Rank =  79  - Median =  49.05
## Variable importance for Cultivar:  Rank =  15  - Median =  301.6
## Variable importance for Tissue:    Rank =  38  - Median =  223.4
## Variable importance for Soil:      Rank =  40  - Median =   5.96
##
## Gene ID               Gene Annotation
## VIT_01s0137g00780  -  Unknown protein
## VIT_13s0019g02710  -  Rho guanyl-nucleotide exchange factor ROPGEF5
## VIT_06s0004g05020  -  Haloacid dehalogenase hydrolase
## VIT_06s0004g05790  -  Unknown protein
## VIT_13s0067g01570  -  Lectin-like receptor kinase 7;2
## VIT_09s0002g03750  -  GATA transcription factor 11
## VIT_05s0020g04940  -  NBS-LRR type disease resistance protein
## VIT_02s0025g02560  -  O-succinylhomoserine sulfhydrylase
## VIT_16s0098g00990  -  No hit
## VIT_13s0047g00140  -  RKF1 (receptor-like kinase in flowers 1)
## VIT_00s0341g00070  -  ERF/AP2 Gene Family (VvERF031),Dehydration Responsive Element-Binding
## VIT_13s0019g00480  -  Zinc finger (C2H2 type) family
## VIT_11s0016g02790  -  Zinc finger (CCCH-type) family protein
## VIT_08s0007g03800  -  Unknown protein
## VIT_00s0632g00010  -  Dehydration Responsive Element-Binding Transcription Factor (VvDREB36)
## VIT_16s0022g01330  -  R protein  PRF  disease resistance protein
## VIT_05s0020g04930  -  RPM1 (resistance to p. syringae pv maculicola 1)
## VIT_03s0063g00880  -  Calmodulin-binding protein
## VIT_08s0040g02600  -  F-box protein
## VIT_00s0203g00160  -  Cyclin D-type
## VIT_02s0025g05090  -  Microtubule motor PAK (phosphatidic acid kinase) KHC
## VIT_18s0041g00160  -  Disease resistance protein (NBS class)
## VIT_14s0066g01570  -  Unknown protein
## VIT_08s0007g08250  -  Nuclear transcription factor Y subunit A-9
## VIT_00s0220g00070  -  Latex cyanogenic beta glucosidase
## VIT_12s0059g00960  -  Cellulose synthase CSLB04
## VIT_01s0026g02710  -  NAC domain-containing protein (VvNAC26)
## VIT_05s0051g00180  -  Glutathione S-transferase 8 GSTU8
## VIT_06s0080g00040  -  ABC Transporter (VvPDR33 - VvABCG63)
## VIT_16s0148g00280  -  Receptor kinase homolog LRK10
## VIT_12s0035g02120  -  Unknown
## VIT_17s0119g00120  -  Alpha-amylase/subtilisin inhibitor
## VIT_08s0007g00270  -  No hit
## VIT_16s0039g00930  -  CYP89A5
## VIT_08s0056g00610  -  Transposon protein, Mutator sub-class
## VIT_02s0012g00400  -  1-aminocyclopropane-1-carboxylate oxidase
## VIT_05s0020g00830  -  CBL-interacting protein kinase 1 (CIPK1)
## VIT_16s0148g00380  -  Receptor kinase LRK10
## VIT_05s0094g01120  -  NADP-dependent oxidoreductase
## VIT_05s0077g01250  -  Endochitinase A2 precursor
## VIT_16s0039g01310  -  Receptor serine/threonine kinase PR5K
## VIT_07s0005g00850  -  ERS type ethylene receptor
## VIT_17s0000g03330  -  Receptor serine/threonine kinase PR5K
## VIT_02s0025g01500  -  Nucleoside diphosphate kinase 3, (NDK3)
## VIT_16s0039g01330  -  Receptor kinase homolog LRK10
## VIT_06s0004g00310  -  Hcr2-p4.1
```

```
## VIT_03s0063g00710  -  Carboxyesterase 12; CXE12
## VIT_16s0050g00900  -  Anthocyanin permease (VvAnthoMATE3 - VvAM3)
## VIT_08s0007g05580  -  Embryo-abundant protein
## VIT_06s0009g02920  -  Flavonoid 3',5'-hydroxylase (F3'5'H)
## VIT_13s0084g00490  -  No hit
## VIT_11s0016g01650  -  Ankyrin repeat
## VIT_06s0004g01450  -  Lipoxygenase (LOX2)
## VIT_00s0400g00020  -  HcrVf1 protein
## VIT_00s0294g00020  -  Kinase
## VIT_14s0128g00510  -  Protein kinase Xa21
## VIT_11s0016g01620  -  Unknown
## VIT_09s0002g05240  -  Cyclin A2;3
## VIT_02s0012g01570  -  Cinnamoyl-CoA reductase
## VIT_00s0216g00060  -  Nuclear transport factor 2 (NTF2)
## VIT_10s0003g05690  -  Ribulose bisphosphate carboxylase, large chain
## VIT_06s0009g01990  -  Anthocyanin 3-O-galactosyltransferase
## VIT_08s0007g00810  -  Lectin receptor kinase 7
## VIT_18s0001g06750  -  Steroleosin-B
## VIT_16s0039g00850  -  F-box family protein
## VIT_12s0142g00240  -  PDF1 (protodermal factor1)
## VIT_11s0016g01670  -  Ankyrin repeat
## VIT_00s0211g00090  -  Serine hydroxymethyltransferase 2
## VIT_01s0011g05950  -  NSL1 (necrotic spotted lesions 1)
## VIT_03s0063g00730  -  CXE carboxylesterase CXE10
## VIT_11s0016g01530  -  Ankyrin repeat family protein
## VIT_17s0000g02700  -  Alpha-dioxygenase 2
```

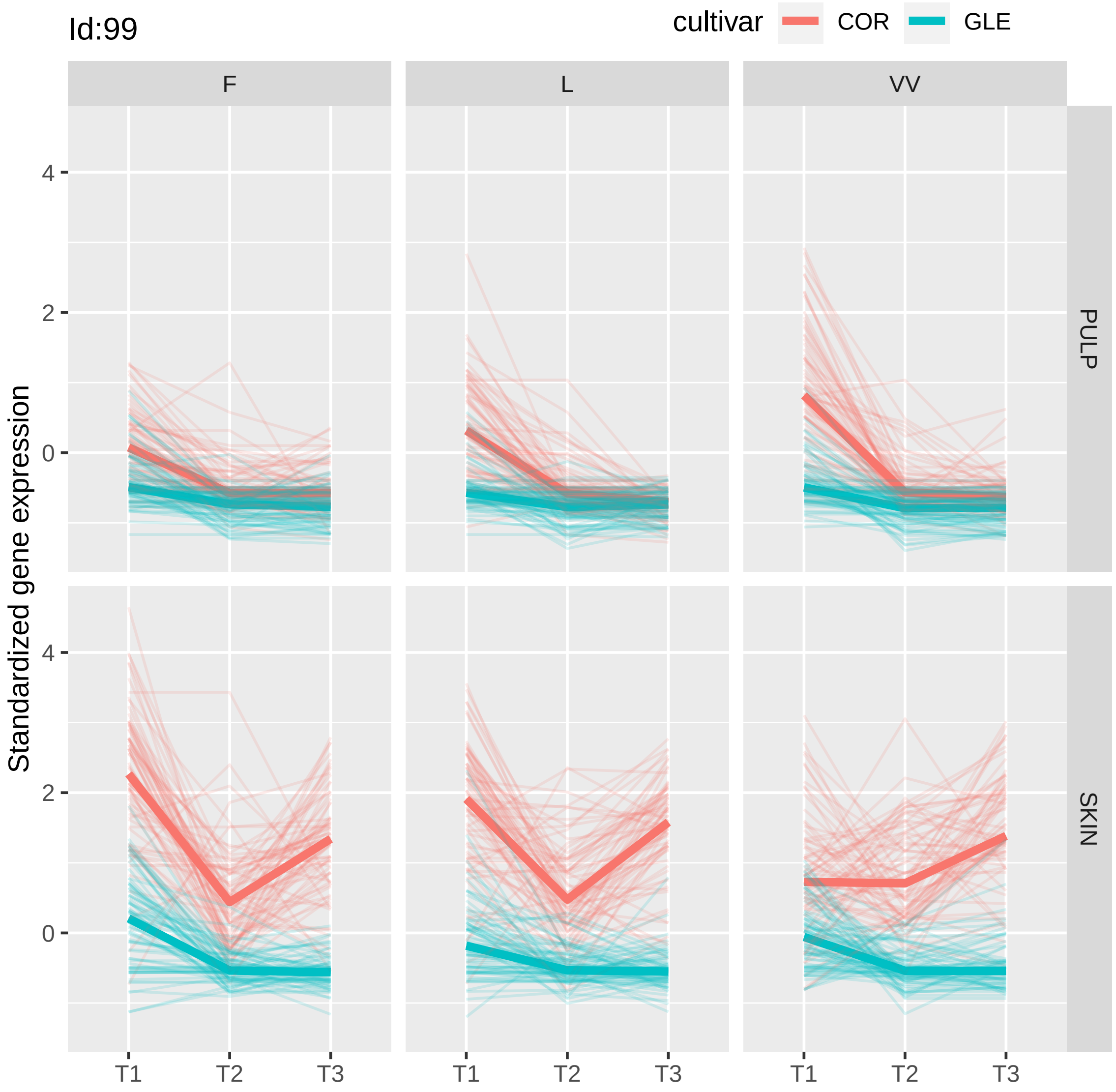

Id:99
cultivar
COR
GLE
F
L
VV
PULP
SKIN
Standardized gene expression
4
2
0
T1
T2
T3

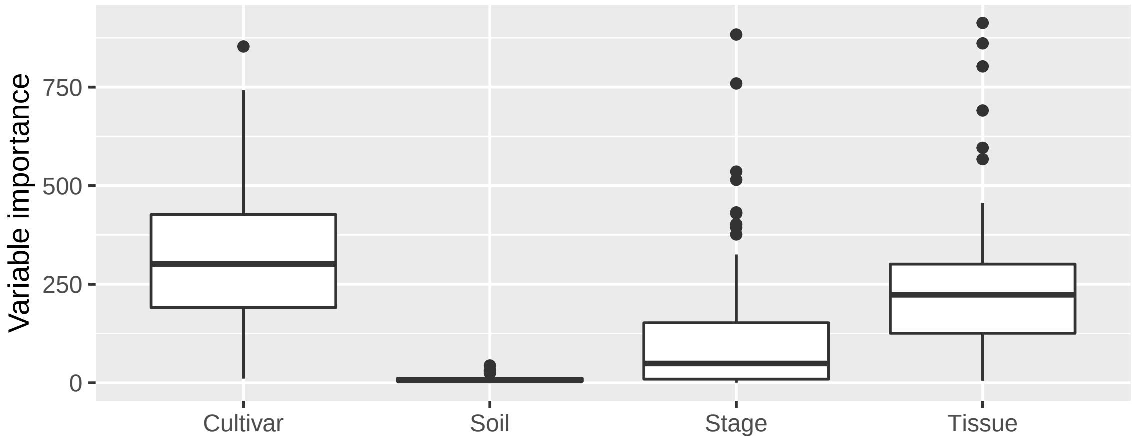

Variable importance
750
500
250
0
Cultivar
Soil
Stage
Tissue

Cluster no. 80

```
## Number of genes in the cluster: 55
## Homogeneity Index:    0.81
## Variable importance for Stage:     Rank =  80  - Median =  48.82
## Variable importance for Cultivar:  Rank =  29  - Median =  122.9
## Variable importance for Tissue:    Rank =  26  - Median =  420.7
## Variable importance for Soil:      Rank =  61  - Median =   5.13
##
## Gene ID                Gene Annotation
## VIT_00s0187g00160  -  Ripening-related protein
## VIT_00s1764g00020  -  Leucine-rich repeat transmembrane protein kinase
## VIT_07s0005g04390  -  Clavata1 receptor kinase (CLV1)
## VIT_00s0203g00050  -  Receptor-interacting protein
## VIT_00s0441g00020  -  Squalene monooxygenase
## VIT_01s0011g00670  -  SNAP25A protein
## VIT_13s0067g01980  -  Proteinase inhibitor (LUTI)
## VIT_08s0007g07690  -  Polygalacturonase inhibiting protein PGIP1
## VIT_01s0150g00210  -  CYP51A
## VIT_08s0007g06520  -  Actin beta/gamma 1
## VIT_06s0009g00370  -  U-box domain-containing protein
## VIT_12s0034g01160  -  Blue (type 1) copper domain
## VIT_08s0040g02500  -  Nodulin MtN21 family
## VIT_12s0059g00670  -  NADP-dependent oxidoreductase
## VIT_09s0002g06430  -  Lactoylglutathione lyase
## VIT_12s0059g00770  -  Monocopper oxidase SKS5 (SKU5 Similar 5)
## VIT_09s0002g06160  -  Ser/thr specific protein kinase
## VIT_18s0072g00660  -  Nodulin MtN21 family
## VIT_16s0050g02680  -  Protein phosphatase 2C
## VIT_17s0000g09150  -  ATP binding
## VIT_00s0322g00010  -  Cyclin-D like protein
## VIT_01s0011g01780  -  F-box protein PP2-B12 (Protein phloem protein 2-like B12)
## VIT_14s0066g00250  -  Lipase GDSL
## VIT_08s0058g01480  -  1,4-beta-mannan endohydrolase
## VIT_11s0016g04880  -  Salt-inducible protein kinase
## VIT_18s0001g10160  -  Wuschel homeobox 4
## VIT_16s0098g01640  -  No hit
## VIT_08s0040g00380  -  Zinc finger (DHHC type) family
## VIT_00s0301g00060  -  No hit
## VIT_03s0038g03940  -  Tonoplast monosaccharide transporter2
## VIT_05s0051g00700  -  No hit
## VIT_04s0008g01700  -  Unknown protein
## VIT_05s0049g00610  -  No hit
## VIT_10s0003g03750  -  9-cis-epoxycarotenoid dioxygenase 2 (NCED2) (VvNCED2)
## VIT_12s0057g00190  -  Unknown
## VIT_18s0001g05300  -  Trehalose-6-phosphate phosphatase
## VIT_15s0024g00400  -  R protein MLA10
## VIT_06s0061g01120  -  UDP-D-apiose/UDP-D-xylose synthase
## VIT_06s0061g01290  -  No hit
## VIT_18s0001g09500  -  CYP81B2v1
## VIT_06s0009g01320  -  L-ascorbate oxidase
## VIT_11s0016g02940  -  Profilin 1
## VIT_08s0058g01460  -  1,4-beta-mannan endohydrolase
## VIT_16s0050g02310  -  No hit
## VIT_05s0029g00140  -  ERF/AP2 Gene Family (VvERF005),Dehydration Responsive Element-Binding
## VIT_05s0077g01460  -  No hit
```

```
## VIT_08s0007g03260  -  Unknown protein
## VIT_17s0000g01010  -  Lysophospholipase homolog
## VIT_02s0025g04890  -  CYP76B1
## VIT_05s0102g00450  -  MSS3 (multicopy suppressors of snf4 deficiency in yeast 3)
## VIT_13s0106g00690  -  No hit
## VIT_06s0009g01340  -  Ascorbate oxidase precursor
## VIT_02s0025g04520  -  Calcium-dependent protein kinase 1 CDPK protein kinase
## VIT_05s0094g00770  -  Short-chain dehydrogenase/reductase (SDR)
## VIT_15s0048g01420  -  Plastocyanin domain-containing protein
```

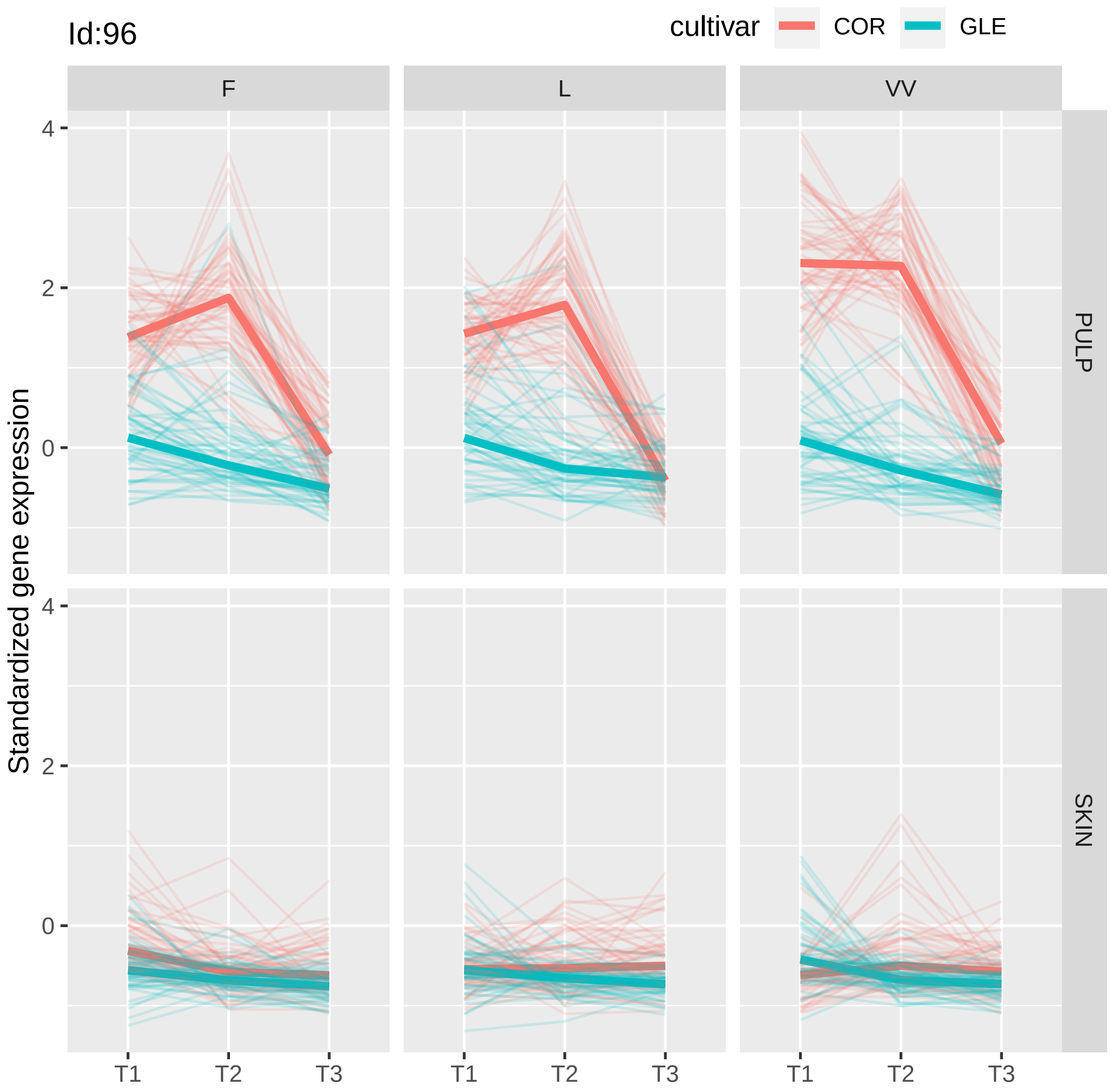
Id:96
cultivar
COR
GLE
F
L
VV
PULP
SKIN
Standardized gene expression
4
2
0
T1
T2
T3

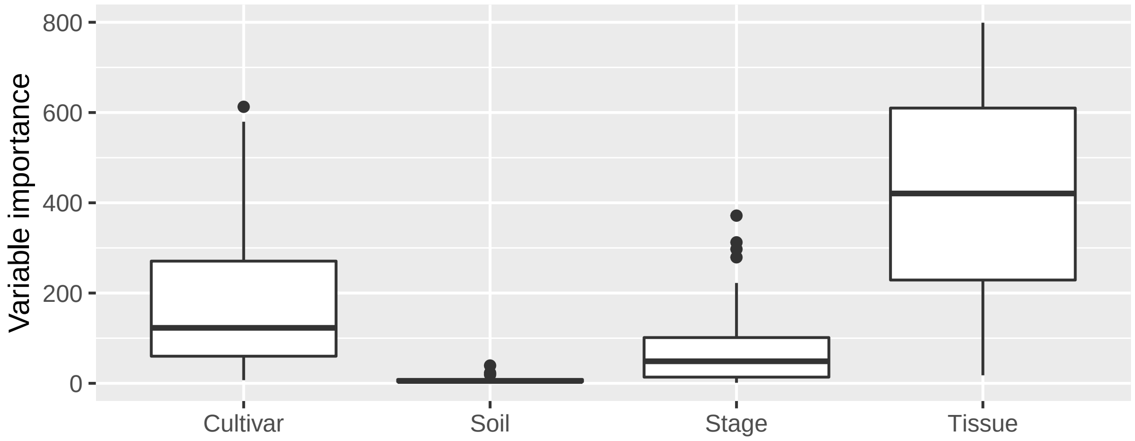
Variable importance
800
600
400
200
0
Cultivar
Soil
Stage
Tissue

Cluster no. 81

```
## Number of genes in the cluster: 78
## Homogeneity Index:    0.84
## Variable importance for Stage:     Rank =  81  - Median =  45.64
## Variable importance for Cultivar:  Rank =  60  - Median =  26.11
## Variable importance for Tissue:    Rank =   7  - Median =  916.3
## Variable importance for Soil:      Rank =  85  - Median =   3.61
##
## Gene ID               Gene Annotation
## VIT_04s0008g05210  -  Basic Leucine Zipper Transcription Factor (VvbZIP10)
## VIT_05s0020g03670  -  MARD1 (mediator of ABA-regulated dormancy 1)
## VIT_00s0398g00020  -  S-receptor kinase KIK1 precursor
## VIT_03s0063g01590  -  CYP82C4
## VIT_09s0002g04260  -  Unknown protein
## VIT_08s0007g00920  -  Tropinone reductase
## VIT_02s0012g00380  -  Norcoclaurine synthase
## VIT_14s0068g00070  -  feronia receptor-like kinase
## VIT_03s0063g00550  -  Fw2.2 ORFX
## VIT_13s0019g04260  -  Monoglyceride lipase
## VIT_03s0063g02240  -  PLATZ transcription factor
## VIT_16s0098g00410  -  Receptor kinase homolog LRK10
## VIT_18s0001g09800  -  S-receptor kinase
## VIT_05s0077g00030  -  Ammonium transporter 1, member 1 (AMT1.1)
## VIT_17s0000g02530  -  FK506-binding protein genes family (VvFKBP17-2)
## VIT_10s0116g01860  -  MATE efflux family protein
## VIT_04s0008g03940  -  BURP domain-containing protein
## VIT_07s0129g00470  -  Unknown protein
## VIT_17s0000g00130  -  SEC14 cytosolic factor
## VIT_12s0142g00640  -  FLS2 (flagellin-sensitive 2)
## VIT_00s0194g00190  -  No hit
## VIT_18s0041g00220  -  TIR-NBS-LRR disease resistance
## VIT_00s0286g00140  -  Serine/threonine-protein kinase receptor ARK3
## VIT_03s0088g01150  -  Squalene monooxygenase
## VIT_18s0001g14770  -  Lipase 3 (EXL3) family II extracellular
## VIT_18s0001g11170  -  Myb domain protein 73
## VIT_14s0066g00900  -  Cysteine-rich receptor-like protein kinase 42
## VIT_17s0000g03690  -  Ribulose-1,5-bisphophate carboxylase/oxygenase small subunit
## VIT_13s0074g00660  -  ABC Transporter (VvPDR24 - VvABCG54)
## VIT_00s0194g00250  -  KCO1 (two pore K channel)
## VIT_05s0094g00990  -  UDP-glucose:salicylic acid glucosyltransferase
## VIT_18s0089g00510  -  Isopentenyltransferase 1
## VIT_14s0068g00100  -  feronia receptor-like kinase
## VIT_14s0108g01080  -  myb domain protein 106
## VIT_03s0063g01890  -  Ring-H2 finger protein ATL3G
## VIT_17s0000g09540  -  CYP71A26
## VIT_16s0039g01670  -  Cinnamoyl-CoA reductase
## VIT_09s0002g01090  -  flavonoid 3-monooxygenase
## VIT_03s0017g01040  -  Cis-zeatin O-beta-D-glucosyltransferase
## VIT_18s0001g14280  -  ER lumen protein retaining receptor
## VIT_08s0007g07480  -  Aldose 1-epimerase
## VIT_17s0000g09550  -  CYP71A26
## VIT_13s0106g00060  -  Ankyrin repeat
## VIT_07s0031g02950  -  Ammonium transporter 2
## VIT_05s0102g00370  -  Receptor protein kinase (ACR4)
## VIT_13s0106g00070  -  Ankyrin repeat protein
```

```
## VIT_06s0004g06890  -  Potassium transporter (KUP1)
## VIT_18s0001g11570  -  CYP82C1p
## VIT_11s0016g01060  -  Avr9/Cf-9 induced kinase 1
## VIT_18s0001g15310  -  Thioredoxin M-type
## VIT_11s0016g04540  -  ABC Transporter (VvPDR1 - VvABCG31)
## VIT_18s0041g00210  -  TIR-NBS-LRR disease resistance
## VIT_02s0025g04550  -  Glucose-methanol-choline (GMC) oxidoreductase
## VIT_14s0068g00110  -  feronia receptor-like kinase
## VIT_05s0020g04540  -  Beta-ketoacyl-CoA synthase
## VIT_09s0002g04010  -  Ferredoxin
## VIT_04s0023g01550  -  Unknown
## VIT_19s0014g04150  -  S-receptor protein kinase
## VIT_07s0104g01730  -  HAK5 (High affinity K+ transporter 5)
## VIT_09s0002g02980  -  FRK1 (FLG22-induced receptor-like kinase 1)
## VIT_12s0057g01450  -  Subtilisin protease
## VIT_12s0028g01360  -  PMR5 (powdery mildew resistant 5)
## VIT_00s0532g00070  -  CRK10 (cysteine-rich RLK10); kinase
## VIT_15s0048g01670  -  CYP704G7
## VIT_08s0007g05030  -  myb domain protein 36
## VIT_13s0067g03890  -  Beta-ketoacyl-CoA synthase
## VIT_18s0001g15640  -  Pathogenesis-related
## VIT_01s0011g02330  -  Unknown protein
## VIT_11s0118g00180  -  HHP1 (heptahelical protein 1)
## VIT_19s0014g04130  -  Serine/threonine-protein kinase receptor ARK3
## VIT_17s0053g00400  -  S-receptor protein kinase
## VIT_15s0107g00210  -  Cinnamyl alcohol dehydrogenase
## VIT_09s0018g01650  -  ERF/AP2 Gene Family (VvAP2-06)
## VIT_05s0020g01820  -  Integral membrane family protein UPF0497
## VIT_15s0048g01680  -  CYP704G7
## VIT_13s0067g03220  -  Unknown protein
## VIT_17s0000g03750  -  Chitin elicitor-binding CEBIP LysM domain-containing
## VIT_02s0012g01190  -  Zinc finger (C2H2 type) family
```

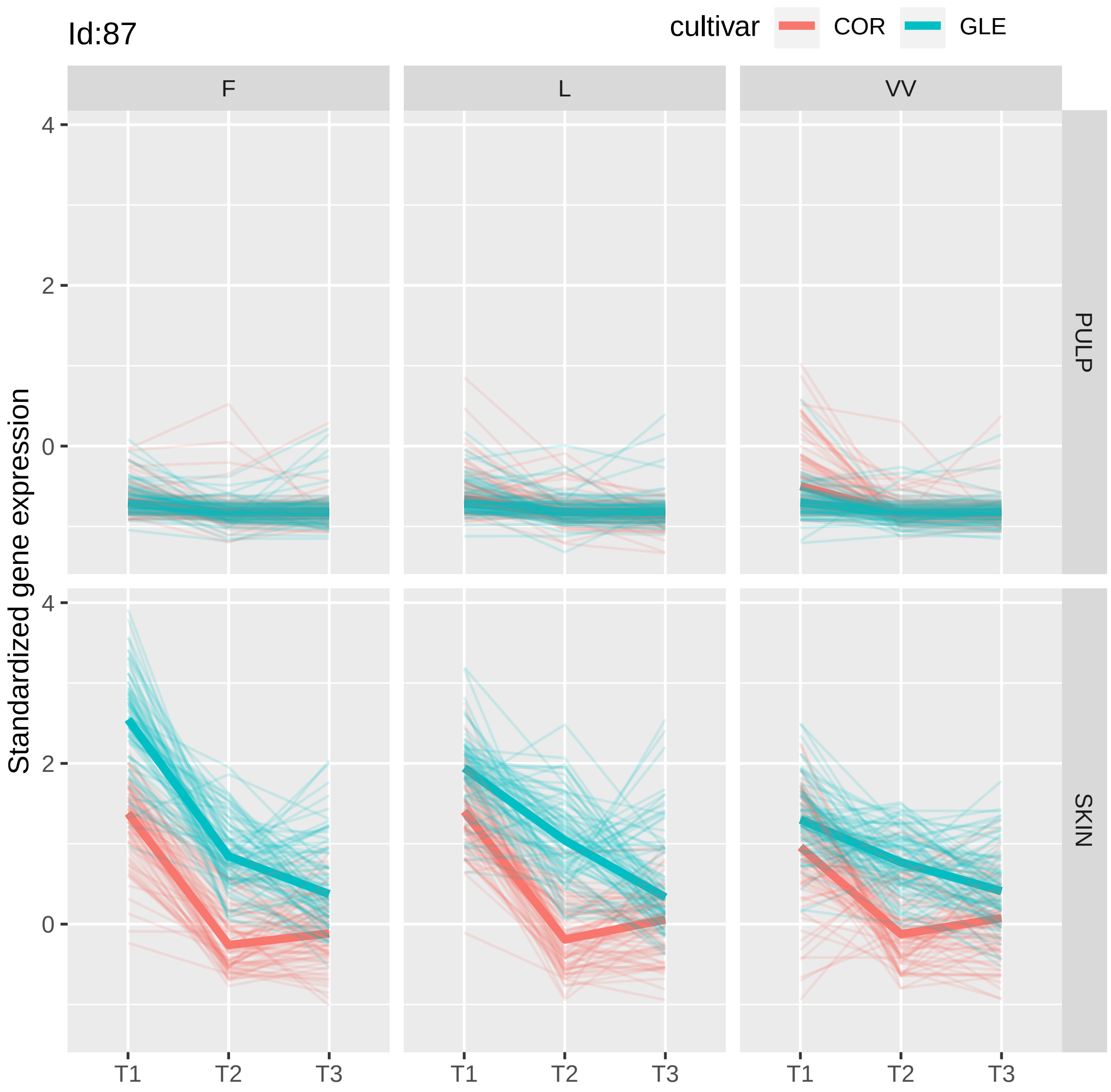
Id:87
cultivar
COR
GLE
F
L
VV
PULP
SKIN
Standardized gene expression
4
2
0
T1
T2
T3

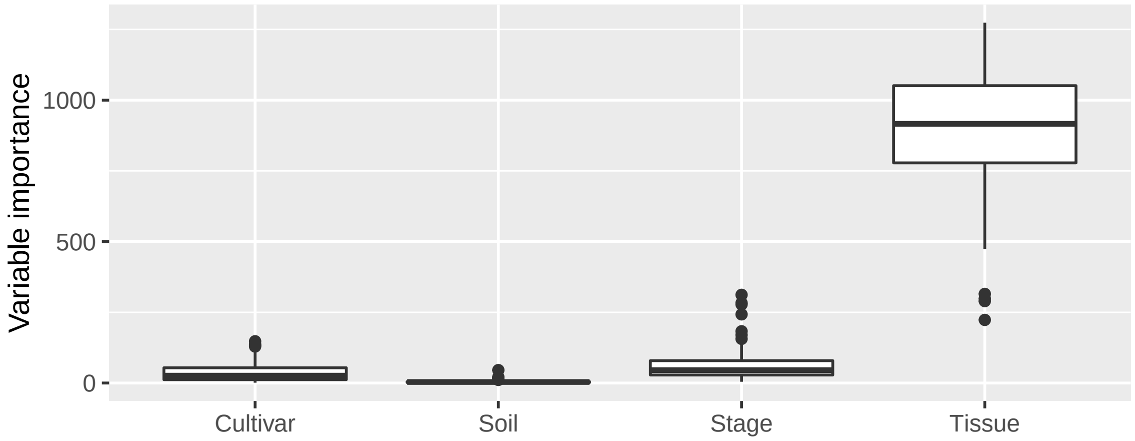
Variable importance
1000
500
0
Cultivar
Soil
Stage
Tissue

Cluster no. 82

```
## Number of genes in the cluster: 48
## Homogeneity Index:    0.82
## Variable importance for Stage:     Rank =  82  - Median =  37.24
## Variable importance for Cultivar:  Rank =  59  - Median =  26.86
## Variable importance for Tissue:    Rank =   8  - Median =  897.5
## Variable importance for Soil:      Rank =  78  - Median =   4.03
##
## Gene ID                Gene Annotation
## VIT_03s0038g02190  -  Nodulin
## VIT_18s0001g13580  -  Kinesin motor protein
## VIT_14s0083g00520  -  Proline oxidase
## VIT_19s0014g01370  -  Curculin (mannose-binding) lectin
## VIT_03s0180g00290  -  Gibberellin-regulated protein 4 (GASA4)
## VIT_00s0266g00020  -  Linalool synthase (VvTPS64)
## VIT_05s0049g01450  -  No hit
## VIT_16s0148g00090  -  GASA4
## VIT_04s0023g00530  -  Auxin responsive SAUR protein
## VIT_18s0001g13080  -  Unknown protein
## VIT_19s0090g00080  -  Ubiquitin-conjugating enzyme E2 variant
## VIT_09s0054g01080  -  Polygalacturonase QRT3
## VIT_19s0090g00120  -  Ubiquitin-conjugating enzyme E2 variant
## VIT_00s0271g00010  -  Linalool synthase (VvTPS55)
## VIT_00s0317g00140  -  Exocyst subunit EXO70 H2
## VIT_00s0385g00020  -  Linalool synthase (VvTPS60)
## VIT_02s0025g02620  -  BDG1 (BODYGUARD1) hydrolase
## VIT_14s0108g00740  -  GASA4
## VIT_06s0004g06390  -  UDP-glucose glucosyltransferase
## VIT_11s0052g01630  -  Flavonoid 3-O-glucosyltransferase
## VIT_00s0253g00110  -  Methyl jasmonate esterase
## VIT_03s0038g03950  -  Phosphate carrier protein
## VIT_01s0026g01340  -  Glutathione S-transferase 29 GSTU18
## VIT_12s0057g01060  -  Jasmonate O-methyltransferase
## VIT_00s1317g00010  -  Gibberellin-regulated protein 4 (GASA4)
## VIT_03s0088g00270  -  No hit
## VIT_06s0004g05350  -  Tropinone reductase
## VIT_01s0026g02620  -  Expansin  (VvEXPA1)
## VIT_05s0020g04110  -  ELIP1 (early light-inducible protein)
## VIT_08s0040g03280  -  HCF152 (high chlorophyll fluorescence 152)
## VIT_12s0134g00450  -  S-locus lectin protein kinase
## VIT_03s0091g01090  -  Unknown
## VIT_06s0004g05400  -  Tropinone reductase
## VIT_00s0379g00050  -  Short-chain dehydrogenase/reductase (SDR)
## VIT_13s0074g00550  -  Amino acid transporter family
## VIT_01s0010g02910  -  ATP binding protein
## VIT_06s0004g05320  -  Tropinone reductase
## VIT_13s0047g00010  -  ZIFL2 (zinc induced facilitator-like 1)
## VIT_04s0008g02250  -  Beta-ketoacyl-CoA synthase
## VIT_03s0091g00230  -  Unknown protein
## VIT_15s0048g01490  -  Geraniol 10-hydroxylase
## VIT_13s0074g00390  -  CYP77A2
## VIT_11s0052g01600  -  UDP-glucose flavonoid 3-O-glucosyltransferase 7
## VIT_06s0004g05380  -  Tropinone reductase
## VIT_18s0001g12490  -  O-methyltransferase
## VIT_03s0063g01880  -  Acyl-CoA synthetase long-chain member 2
```

```
## VIT_19s0014g00080  -  Steroid 5alpha-reductase
## VIT_04s0069g00540  -  Glutamate receptor protein
```

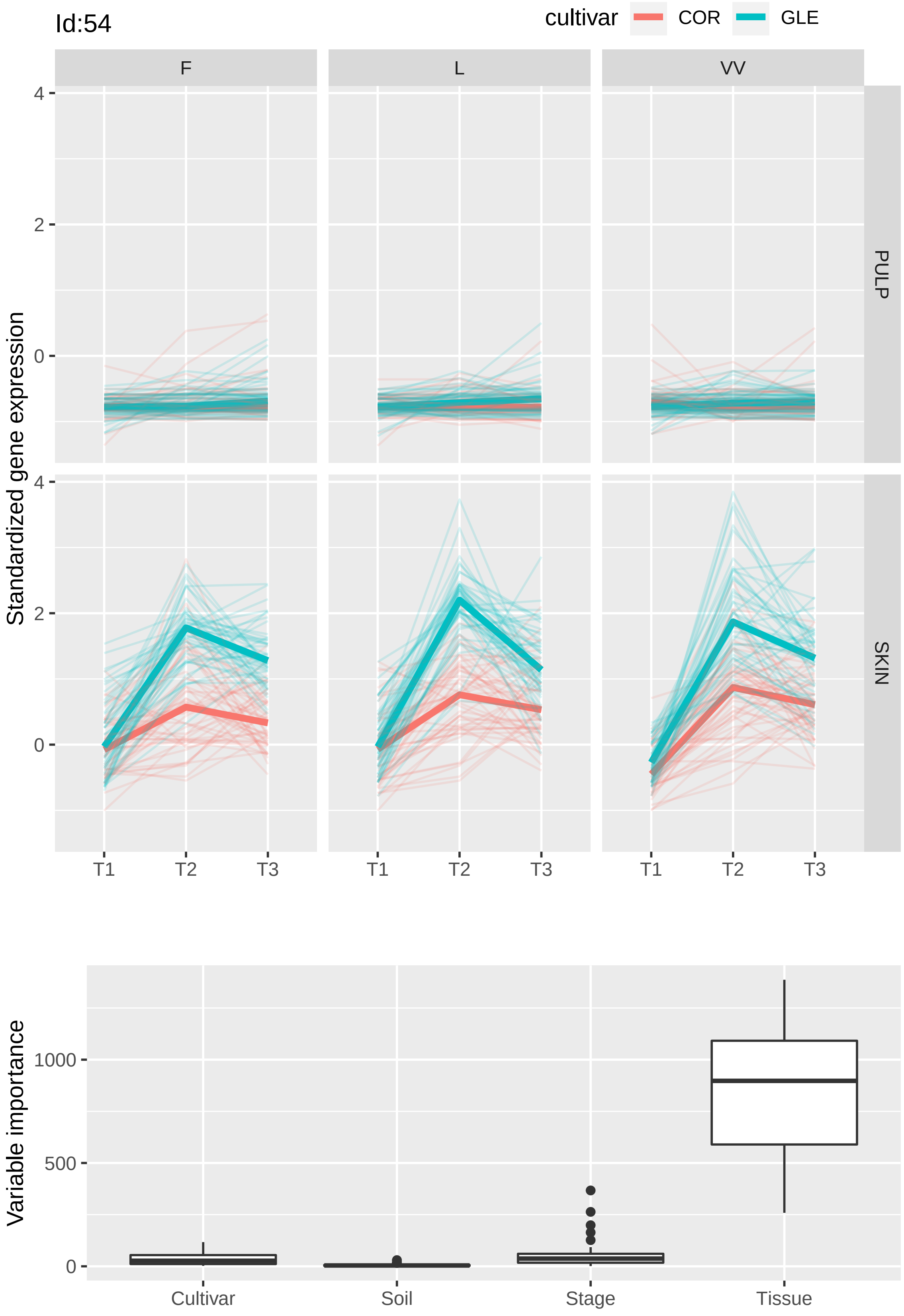

Id:54
cultivar
COR
GLE
F
L
VV
PULP
SKIN
Standardized gene expression
4
2
0
T1
T2
T3
Variable importance
1000
500
0
Cultivar
Soil
Stage
Tissue

Cluster no. 83

```
## Number of genes in the cluster: 69
## Homogeneity Index:    0.75
## Variable importance for Stage:     Rank =  83  - Median =  29.82
## Variable importance for Cultivar:  Rank =  64  - Median =  20.88
## Variable importance for Tissue:    Rank =   9  - Median =  886.9
## Variable importance for Soil:      Rank =  80  - Median =   3.82
##
## Gene ID               Gene Annotation
## VIT_18s0001g02790  -  Unknown protein
## VIT_13s0156g00630  -  S-receptor kinase
## VIT_19s0090g01360  -  No hit
## VIT_04s0023g02280  -  S-adenosyl-L-methionine:salicylic acid carboxyl methyltransferase
## VIT_05s0049g00770  -  No hit
## VIT_09s0002g05750  -  Bromo adjacent region
## VIT_16s0039g00880  -  CYP89H3
## VIT_08s0040g01780  -  Lysine decarboxylase
## VIT_04s0008g07330  -  No hit
## VIT_05s0049g00800  -  Unknown protein
## VIT_07s0005g01700  -  Transcription regulator
## VIT_01s0010g02030  -  Gamma-thionin precursor
## VIT_03s0063g00860  -  Serine carboxypeptidase
## VIT_18s0086g00680  -  No hit
## VIT_01s0011g05140  -  Major latex-like protein ripening protein [grip61]
## VIT_04s0023g00050  -  Sterol glucosyltransferase
## ENSRNA049469475  -
## VIT_07s0031g02840  -  Diacylglycerol kinase 2
## VIT_13s0158g00360  -  Calcium-transporting ATPase 11 ACA11
## VIT_01s0011g05070  -  Major latex-like protein 34
## VIT_16s0098g01150  -  Auxin-responsive SAUR29
## VIT_01s0011g05120  -  Major latex
## VIT_10s0071g00300  -  Unknown protein
## VIT_08s0007g07330  -  No hit
## VIT_12s0055g00810  -  Cationic peroxidase
## VIT_10s0003g01460  -  Nucleobase-ascorbate transporter 2 (NAT2)
## VIT_01s0011g05110  -  Major latex protein 22
## VIT_12s0057g00180  -  Wound-induced
## VIT_17s0000g02370  -  Receptor protein kinase
## VIT_11s0052g00320  -  Ca2+-ATPase 10 ACA10, plasma membrane
## VIT_02s0025g04870  -  No hit
## VIT_04s0044g01130  -  Alcohol dehydrogenase 2 [Vitis vinifera]
## VIT_00s0225g00060  -  MATE efflux family protein
## VIT_10s0071g00390  -  GRAM domain-containing protein / ABA-responsive
## VIT_10s0071g00330  -  GRAM domain-containing protein / ABA-responsive
## VIT_03s0038g03570  -  Monocopper oxidase SKS5 (SKU5 Similar 5)
## VIT_01s0011g03910  -  Protein phosphatase 2C
## VIT_04s0044g01110  -  Alcohol dehydrogenase 6
## VIT_03s0038g04680  -  Isoflavone reductase Bet v 6.0101
## VIT_17s0000g06200  -  Mini zinc finger 1 MIF1
## VIT_06s0009g01920  -  No hit
## VIT_18s0001g05990  -  UDP-glycosyltransferase 85A1
## VIT_13s0158g00340  -  Glycerol-3-phosphate dehydrogenase
## VIT_04s0008g06390  -  TPR1 (topless-related 1)
## VIT_15s0046g01920  -  ferric reduction oxidase 2
## VIT_14s0060g00280  -  RARE-cold-inducible 2B
```

```
## VIT_19s0014g01770  -  Glutamate receptor protein
## VIT_18s0086g00520  -  No hit
## VIT_04s0008g06400  -  TPR1 (topless-related 1)
## VIT_06s0061g00550  -  Xyloglucan endotransglucosylase/hydrolase 32
## VIT_12s0057g00700  -  Glucan endo-1,3-beta-glucosidase 3 precursor
## VIT_04s0023g03550  -  Thaumatin
## VIT_03s0038g04690  -  Isoflavone reductase protein 6
## VIT_03s0038g03430  -  Expansin  (VvEXLA1)
## VIT_19s0014g01700  -  Unknown
## VIT_13s0067g02140  -  Heavy-metal-associated domain-containing protein
## VIT_01s0011g02790  -  IDA (inflorescence deficient in abscission)
## VIT_03s0038g04700  -  Isoflavone reductase
## VIT_15s0046g03550  -  (+)-neomenthol dehydrogenase
## VIT_05s0049g00330  -  E8 protein
## VIT_01s0010g00240  -  CTP-synthetase
## VIT_18s0001g09080  -  No hit
## VIT_03s0038g03580  -  Lipase GDSL
## VIT_17s0000g01080  -  HVA22E (HVA22-like protein E)
## VIT_17s0000g06300  -  No hit
## VIT_07s0005g06210  -  RPM1 (resistance to p. syringae pv maculicola 1)
## VIT_04s0008g06380  -  TPR1 (topless-related 1)
## VIT_08s0007g00540  -  Haloacid dehalogenase hydrolase
## VIT_14s0030g01880  -  Calmodulin-binding region IQD26
```

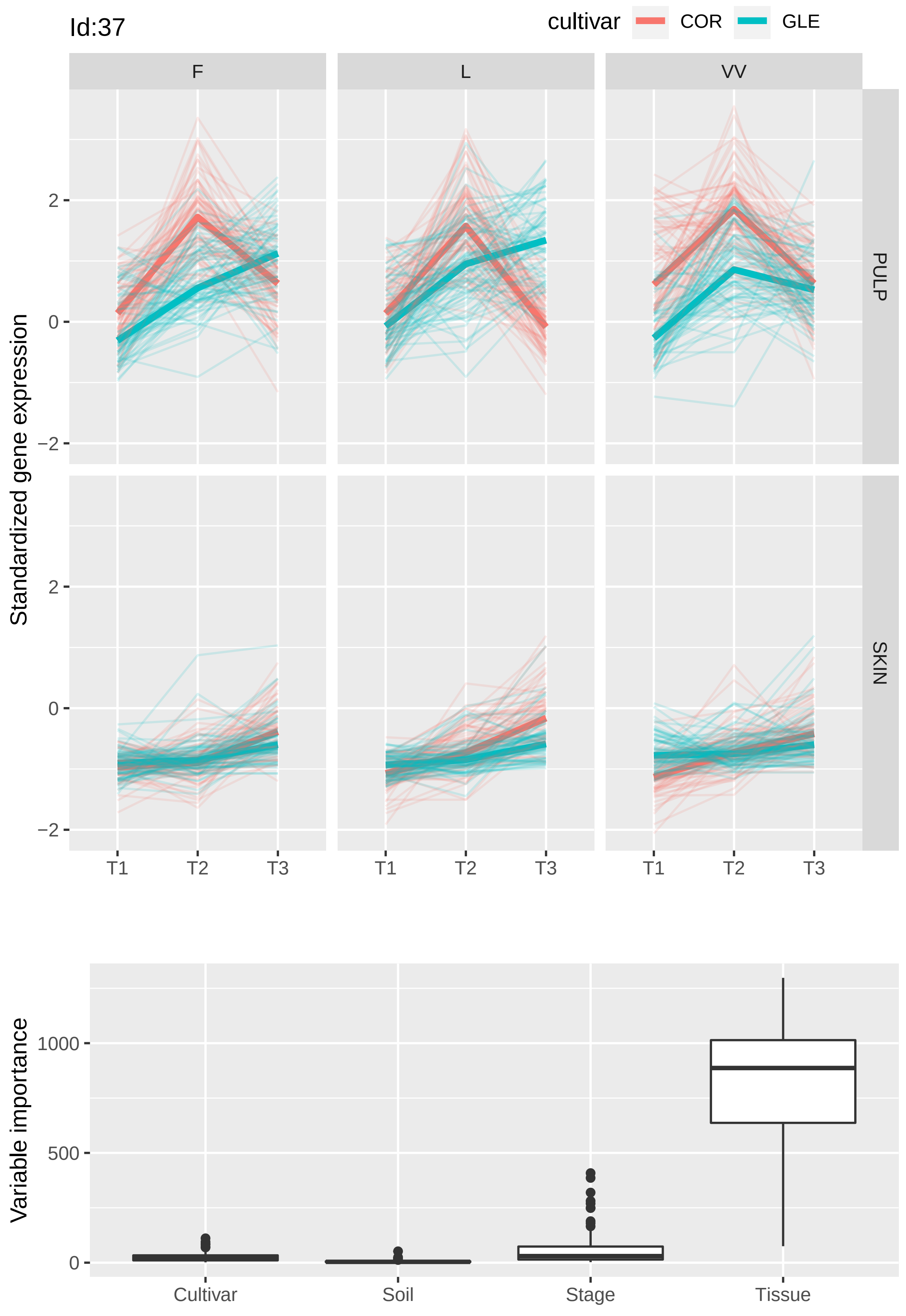

Id:37
cultivar
COR
GLE
F
L
VV
PULP
SKIN
Standardized gene expression
2
0
−2
T1
T2
T3
Variable importance
1000
500
0
Cultivar
Soil
Stage
Tissue

Cluster no. 84

```
## Number of genes in the cluster: 84
## Homogeneity Index:    0.77
## Variable importance for Stage:     Rank =  84  - Median =  27.17
## Variable importance for Cultivar:  Rank =  26  - Median =  154.4
## Variable importance for Tissue:    Rank =  29  - Median =  404.9
## Variable importance for Soil:      Rank =  38  - Median =   6.03
##
## Gene ID               Gene Annotation
## VIT_12s0134g00030  -  E-beta-ocimene synthase
## VIT_00s1389g00010  -  Cinnamyl alcohol dehydrogenase
## VIT_11s0016g03840  -  No hit
## VIT_00s0323g00070  -  Pectin methylesterase inhibitor
## VIT_19s0140g00230  -  No hit
## VIT_10s0092g00110  -  Ribose-5-phosphate isomerase
## VIT_07s0104g00430  -  Endo-1,3;1,4-beta-D-glucanase precursor
## VIT_16s0050g00110  -  Unknown protein
## VIT_05s0020g03680  -  GCN5 N-acetyltransferase (GNAT)
## VIT_18s0041g02430  -  abscisic aldehyde oxidase (AAO3) (VvAAO3)
## VIT_17s0000g06610  -  Zinc finger (C3HC4-type ring finger)
## VIT_07s0141g00700  -  R protein MLA10
## VIT_11s0016g01290  -  CYP72A1
## VIT_17s0000g05360  -  Germin
## ENSRNA049466094  -
## VIT_05s0077g01660  -  s4_Pathogenesis protein 10 [Vitis vinifera]
## VIT_00s0372g00030  -  Linalool synthase (VvTPS66)
## VIT_00s1553g00010  -  Subtilisin-like proteinase AIR3
## VIT_07s0151g00260  -  Quinone-oxidoreductase, Chloroplast
## VIT_05s0077g00520  -  Gibberellin 2-oxidase
## VIT_03s0038g04510  -  Chaperone BCS1 mitochondrial
## VIT_05s0077g01650  -  s5_Pathogenesis protein 10 [Vitis vinifera]
## VIT_00s0266g00040  -  (+)-delta-cadinene synthase isozyme A
## VIT_08s0007g03760  -  Growth-regulating factor 4 AtGRF4
## VIT_06s0004g03700  -  Phototropin-1
## VIT_05s0020g03710  -  GCN5 N-acetyltransferase (GNAT)
## VIT_12s0034g01630  -  No hit
## VIT_18s0089g00450  -  R protein L6
## VIT_10s0003g02840  -  Purine permease 10 PUP10
## VIT_04s0008g01470  -  WRKY Transcription Factor (VvWRKY06)
## VIT_18s0089g00460  -  TIR-NBS-TIR type disease resistance protein
## VIT_19s0014g02290  -  Ribosomal protein L11 methyltransferase
## VIT_13s0067g02560  -  Unknown protein
## VIT_09s0070g00160  -  CBL-interacting protein kinase 6 (VvCIPK02)
## VIT_04s0008g00330  -  Clavata1 receptor kinase (CLV1)
## VIT_19s0015g02900  -  CYP72A1
## VIT_11s0016g03830  -  Protein kinase
## VIT_10s0003g01920  -  RKF1 (receptor-like kinase in flowers 1)
## VIT_05s0049g01550  -  Peptide transporter protein 3
## VIT_00s0187g00020  -  No hit
## VIT_04s0008g00310  -  Clavata1 receptor kinase (CLV1)
## VIT_18s0072g01230  -  R protein L6
## VIT_18s0089g00650  -  CF4
## VIT_14s0060g02450  -  Unknown
## VIT_16s0050g00860  -  Ribosomal protein L31
## VIT_12s0028g00960  -  phytoene synthase (PSY) (VvPSY2)
```

```
## VIT_04s0008g03530  -  Ankyrin repeat
## VIT_04s0079g00790  -  Acyl-CoA synthetases (Acyl-activating enzyme 11)
## VIT_00s0372g00060  -  Linalool synthase (VvTPS58), E,E)-Geranyl linalool syn
## VIT_06s0004g03120  -  MLO-like protein 3
## VIT_17s0000g07800  -  Unknown protein
## VIT_18s0001g06260  -  R protein L6
## VIT_11s0016g00600  -  Beta-ketoacyl ACP synthase, plastidic
## VIT_09s0002g08230  -  PTAC17 (plastid transcriptionally active17)
## VIT_17s0000g08450  -  Carbonic anhydrase chloroplast
## VIT_14s0128g00780  -  Lipoxygenase (VvLOXC)
## VIT_07s0031g01370  -  flavonoid 3-monooxygenase
## VIT_16s0013g00070  -  Unknown
## VIT_13s0047g00500  -  3-hydroxyisobutyryl-CoA hydrolase
## VIT_18s0041g01440  -  R protein L6
## VIT_06s0004g05450  -  Tropinone reductase
## VIT_13s0019g02050  -  Ribulose bisphosphate carboxylase/oxygenase activase, chloroplast
## VIT_19s0014g04590  -  S-locus protein kinase
## VIT_16s0013g00210  -  Metacaspase 1
## VIT_03s0038g00410  -  Enhanced disease susceptibility 5 EDS5
## VIT_13s0067g01880  -  Other LOB domain-containing protein  ASL5
## VIT_12s0142g00690  -  No hit
## VIT_00s0615g00010  -  Cinnamyl alcohol dehydrogenase
## VIT_16s0013g01780  -  Derlin-1
## VIT_13s0067g00490  -  Protein kinase Xa21
## VIT_19s0014g04580  -  S-locus protein kinase
## VIT_12s0035g01080  -  carotenoid isomerase (CISO1) (VvCISO2)
## VIT_13s0074g00800  -  CCHC-type integrase
## VIT_09s0002g06900  -  NtEIG-A1
## VIT_01s0010g00530  -  UDP-glucoronosyl and UDP-glucosyl transferase
## VIT_19s0014g04530  -  S-locus receptor kinase
## VIT_03s0063g01180  -  Gibberellin 20 oxidase 2
## VIT_12s0055g00180  -  UDP-glucose glucosyltransferase
## VIT_09s0002g06890  -  Dicyanin blue copper protein
## VIT_16s0148g00260  -  Ser/Thr receptor-like kinase1
## VIT_08s0040g02100  -  Zinc finger (C2H2 type) JACKDAW
## VIT_00s0258g00040  -  Ser/Thr receptor-like kinase1
## VIT_06s0004g05370  -  Tropinone reductase
## VIT_05s0049g01080  -  Glutathione S-transferase 25 GSTU7
```

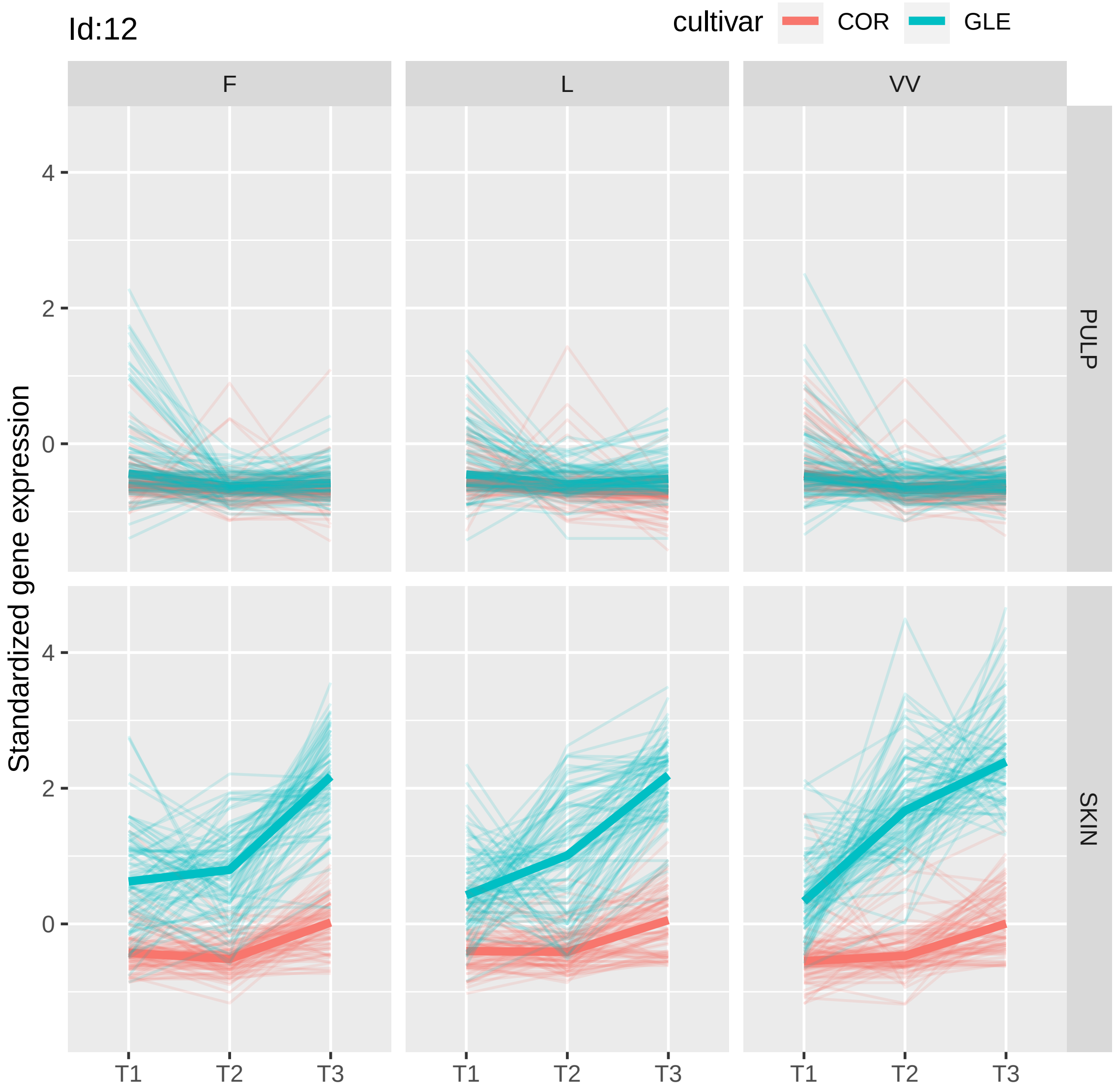
Id:12
cultivar
COR
GLE
F
L
VV
PULP
SKIN
Standardized gene expression
4
2
0
T1
T2
T3

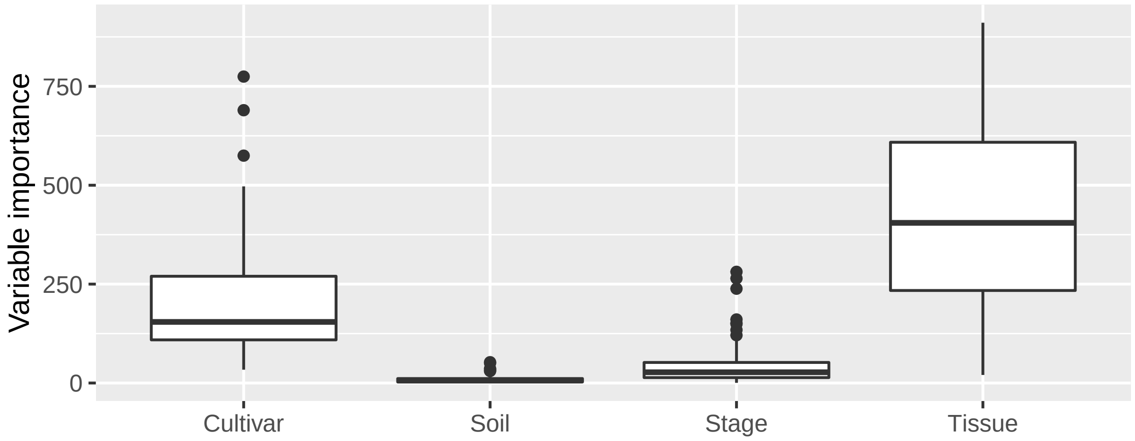
Variable importance
750
500
250
0
Cultivar
Soil
Stage
Tissue

Cluster no. 85

```
## Number of genes in the cluster: 67
## Homogeneity Index:    0.85
## Variable importance for Stage:     Rank =  85  - Median =  25.58
## Variable importance for Cultivar:  Rank =  89  - Median =   9.06
## Variable importance for Tissue:    Rank =   4  - Median =  984.1
## Variable importance for Soil:      Rank =  90  - Median =   3.39
##
## Gene ID                Gene Annotation
## VIT_03s0038g04100  -  3-hydroxy-3-methylglutaryl-coenzyme A reductase 3
## VIT_01s0011g03110  -  myb family
## VIT_14s0066g00470  -  Unknown protein
## VIT_09s0002g00700  -  Dormancy/auxin associated protein
## VIT_01s0011g04590  -  Zinc finger (C3HC4-type ring finger)
## VIT_15s0021g02470  -  Epoxide hydrolase-like protein
## VIT_06s0061g01550  -  No hit
## VIT_19s0014g02360  -  Phytosulfokine receptor precursor
## VIT_10s0003g04390  -  ABC Transporter (VvMRP17 - VvABCC17)
## VIT_17s0053g00710  -  Calcium-dependent protein kinase
## VIT_15s0024g01440  -  Glucose 6 phosphate/phosphate translocator-like protein
## VIT_06s0004g00790  -  Amino acid permease
## VIT_18s0001g04520  -  No hit
## VIT_19s0014g05150  -  RPS2 (resistant to p. syringae 2)
## VIT_14s0068g00280  -  Unknown protein
## VIT_06s0009g00440  -  LHW (lonesome highway)
## VIT_05s0020g04210  -  Sulfate adenylyltransferase 3
## VIT_00s0218g00150  -  UDP-rhamnose/rhamnosyltransferase
## VIT_07s0104g01120  -  Rac GTPase activating protein
## VIT_08s0040g03070  -  WRKY Transcription Factor (VvWRKY26) - (VvPH3)
## VIT_06s0080g00150  -  Subtilisin-like proteinase AIR3
## VIT_05s0049g00350  -  Desacetoxyvindoline 4-hydroxylase
## VIT_06s0009g03630  -  CYP86A2
## VIT_00s0389g00030  -  CYP72A54
## VIT_18s0001g15120  -  Unknown protein
## VIT_14s0081g00200  -  AN3 (ANGUSITFOLIA3)
## VIT_05s0020g04840  -  H1flk, putative
## VIT_10s0116g00400  -  SEC14 cytosolic factor
## VIT_06s0004g03210  -  Unknown
## VIT_18s0001g12340  -  Unknown protein
## VIT_01s0011g04160  -  Phosphatidic acid phosphatase alpha
## VIT_09s0002g03360  -  Calmodulin binding protein
## VIT_14s0068g01960  -  Acetolactate synthase III, chloroplast precursor
## VIT_04s0008g01070  -  Calmodulin-binding region IQD6
## VIT_05s0102g00750  -  Zinc finger (B-box type)
## VIT_17s0000g09710  -  Leucine-rich repeat transmembrane protein kinase
## VIT_09s0002g02420  -  Dual-specific kinase DSK1
## VIT_18s0001g00660  -  High mobility group (HMG1/2) family protein
## VIT_02s0033g00270  -  Pinoresinol-lariciresinol reductase
## VIT_13s0074g00680  -  ABC Transporter (VvPDR25 - VvABCG55)
## VIT_16s0039g01060  -  CYP89A2
## VIT_18s0001g11160  -  Unknown protein
## VIT_09s0002g00420  -  Senescence-associated protein
## VIT_00s0317g00110  -  Esterase/lipase/thioesterase
## VIT_07s0129g00380  -  Phototropic-responsive NPH3
## VIT_18s0001g02420  -  Aspartic Protease (VvAP40)
```

```
## VIT_17s0000g04470  -  No hit
## VIT_04s0069g00720  -  Glutamate receptor protein
## VIT_11s0016g00890  -  Unknown protein
## VIT_05s0062g00740  -  UDP-glucoronosyl/UDP-glucosyl transferase UGT75C1
## VIT_03s0063g00140  -  Caffeoyl-CoA O-methyltransferase (CCoAOMT2)
## VIT_17s0000g02950  -  Glutathione S-transferase 25 GSTU7
## VIT_17s0000g02940  -  Glutathione S-transferase 8 GSTU8
## VIT_06s0004g02750  -  Auxin response factor 16
## VIT_02s0012g01390  -  Unknown
## VIT_18s0166g00190  -  U-box domain-containing protein
## VIT_09s0002g00220  -  Avr9/Cf-9 rapidly elicited protein 132
## VIT_17s0000g03510  -  Serine/threonine kinase BRLK
## VIT_17s0000g05610  -  Isopiperitenol dehydrogenase
## VIT_08s0007g04030  -  Unknown protein
## VIT_18s0001g08380  -  Homeobox protein knotted-1 like 1 (KNAT1)
## VIT_07s0005g01520  -  WRKY Transcription Factor (VvWRKY18)
## VIT_12s0057g01080  -  Kelch repeat-containing protein
## VIT_03s0088g00300  -  Unknown
## VIT_05s0124g00240  -  basic helix-loop-helix (bHLH) family
## VIT_16s0039g01410  -  Tocopherol O-methyltransferase, chloroplast precursor
## VIT_16s0098g00420  -  Ser/Thr receptor-like kinase1
```

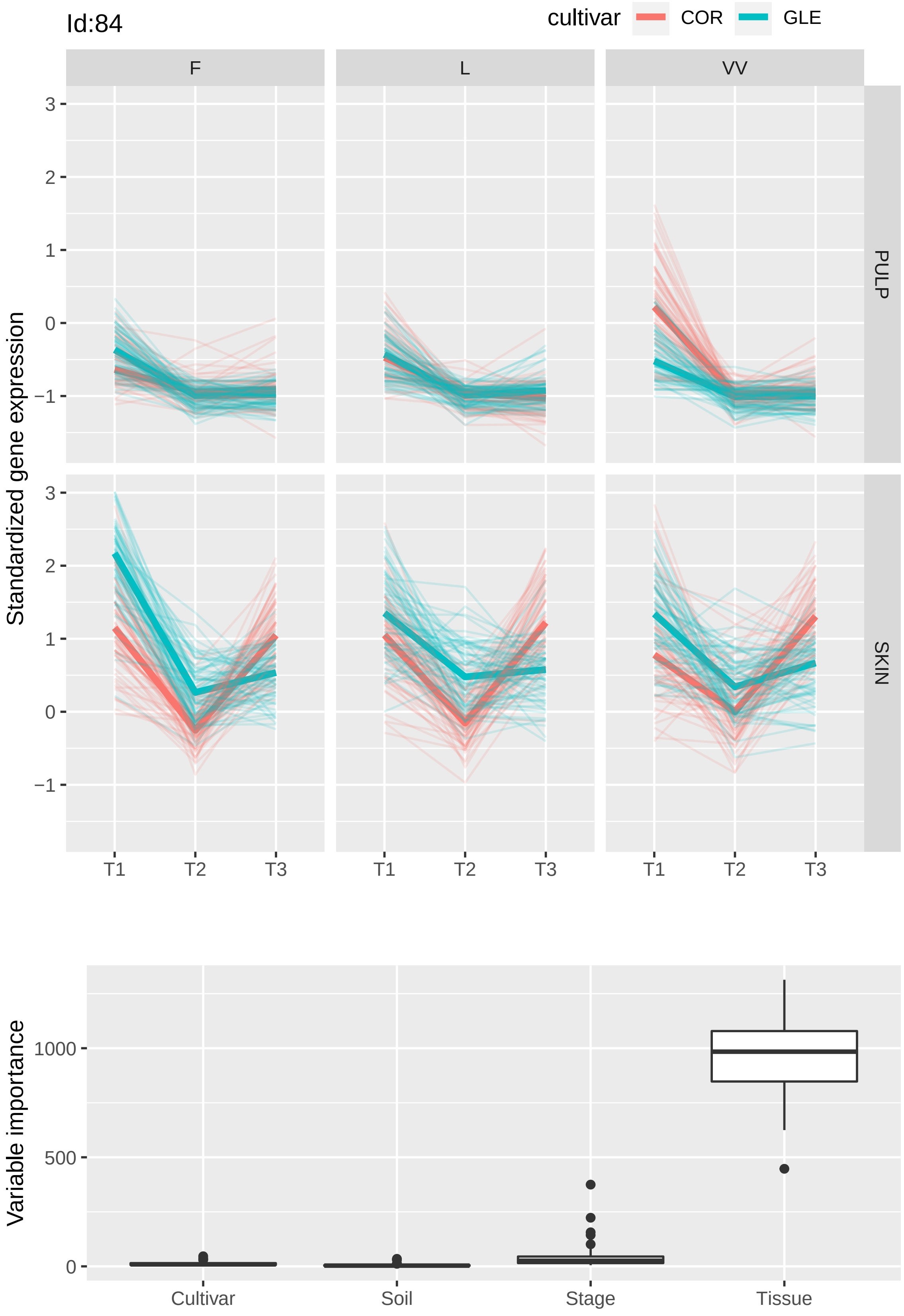
Id:84
cultivar
COR
GLE
F
L
VV
PULP
SKIN
Standardized gene expression
3
2
1
0
−1
T1
T2
T3
Variable importance
1000
500
0
Cultivar
Soil
Stage
Tissue

Cluster no. 86

```
## Number of genes in the cluster: 74
## Homogeneity Index:     0.8
## Variable importance for Stage:     Rank =  86  - Median =  23.89
## Variable importance for Cultivar:  Rank =  23  - Median =  181.7
## Variable importance for Tissue:    Rank =  23  - Median =  481.9
## Variable importance for Soil:      Rank =  74  - Median =   4.54
##
## Gene ID               Gene Annotation
## VIT_07s0104g01320  -  Transport inhibitor response 1
## VIT_08s0007g08420  -  R protein MLA10
## VIT_08s0040g02900  -  Aluminum sensitive 3
## VIT_10s0071g00930  -  Prolyl oligopeptidase
## VIT_11s0016g05380  -  Acidic leucine-rich nuclear phosphoprotein 32 member E
## VIT_15s0046g00980  -  Cyclic nucleotide-regulated ion channel 1
## VIT_10s0003g05030  -  Leucine-rich repeat family protein
## VIT_15s0048g01860  -  No hit
## VIT_18s0072g00990  -  Leucine-rich repeat protein kinase
## VIT_04s0023g00940  -  Phospholipase A1
## VIT_03s0091g00100  -  Methylthioribose kinase
## VIT_06s0004g06080  -  Proton-dependent oligopeptide transport (POT) family protein
## VIT_05s0020g03270  -  S-receptor kinase
## VIT_04s0044g00720  -  S-receptor kinase
## VIT_18s0089g01210  -  Cupin super
## VIT_06s0009g01070  -  Unknown protein
## VIT_05s0020g03000  -  2-Hydroxyisoflavanone dehydratase
## VIT_00s0469g00020  -  Cellulose synthase CSLE1
## VIT_18s0001g01660  -  NADH dehydrogenase I subunit M
## VIT_10s0042g00240  -  Rpp4 candidate 1
## VIT_06s0004g05670  -  Glutathione S-transferase 25 GSTU7
## VIT_08s0007g02800  -  Pro-X carboxypeptidase Lysosomal
## VIT_08s0007g05630  -  No hit
## VIT_04s0023g01210  -  Geranylgeranyl pyrophosphate synthetase 1, chloroplast precursor
## VIT_10s0003g04460  -  ABC Transporter (VvMRP19 - VvABCC19)
## VIT_02s0025g02850  -  Subtilisin protease
## VIT_00s0591g00020  -  3-deoxy-D-arabino-heptulosonate 7-phosphate synthase
## VIT_03s0038g02400  -  Protein kinase
## VIT_01s0011g03070  -  ERF/AP2 Gene Family (VvRAV1)
## VIT_00s0469g00040  -  Cellulose synthase CSLE1
## VIT_15s0046g01650  -  Unknown
## VIT_05s0094g00810  -  Ion channel DMI1, chloroplast precursor
## VIT_10s0092g00570  -  Leucine-rich repeat family
## VIT_16s0039g01910  -  Vegetative storage protein win4.5
## VIT_01s0010g03870  -  Unknown protein
## VIT_01s0011g04760  -  myb domain protein 4 (VvMybC2-L1)
## VIT_06s0009g01350  -  R protein  PRF  disease resistance protein
## VIT_18s0041g00250  -  R protein L6
## VIT_18s0001g01310  -  Wall-associated receptor kinase 5
## VIT_16s0050g02100  -  Disease resistance
## VIT_17s0000g04110  -  No hit
## VIT_10s0116g01870  -  MATE efflux family protein
## VIT_04s0008g02510  -  Receptor kinase homolog LRK10
## VIT_15s0048g01700  -  CYP86A1
## VIT_19s0090g00180  -  5-alpha-taxadienol-10-beta-hydroxylase
## VIT_03s0167g00190  -  CYP714A1
```

```
## VIT_01s0011g06500  -  NADPH-dependent codeinone reductase
## ENSRNA049996164  -
## VIT_05s0029g01540  -  Ankyrin repeat
## VIT_18s0089g00310  -  Unknown
## VIT_03s0017g01660  -  Scarecrow transcription factor 23 (SCL23)
## VIT_13s0067g01350  -  basic helix-loop-helix (bHLH) family
## VIT_09s0002g02080  -  CYP90D2
## VIT_13s0084g00140  -  Zinc finger (C3HC4-type ring finger)
## VIT_03s0017g01370  -  UDP-glycosyltransferase 85A8
## VIT_15s0024g00980  -  Amidase
## VIT_11s0206g00050  -  Esterase/lipase/thioesterase
## VIT_12s0028g00860  -  NAC domain-containing protein (VvNAC36)
## VIT_00s1349g00010  -  Cellulose synthase CSLE1
## VIT_15s0048g00200  -  Gag-pol polyprotein
## VIT_06s0061g00240  -  ABC transporter G member 7
## VIT_11s0037g00680  -  Leucine Rich Repeat receptor-like kinase
## VIT_00s0194g00210  -  KCO1 (two pore K channel)
## VIT_05s0094g01230  -  Allyl alcohol dehydrogenase
## VIT_19s0015g02420  -  Serine/threonine-protein kinase receptor ARK3
## VIT_10s0003g02850  -  Purine permease 10 PUP10
## VIT_15s0048g01050  -  Unknown
## VIT_08s0007g01200  -  S-locus receptor protein kinase
## VIT_10s0003g03330  -  Receptor-like serine-threonine protein kinase
## VIT_01s0182g00130  -  PHO1-like protein
## VIT_12s0034g01250  -  TIR-NBS-LRR disease resistance
## VIT_13s0106g00010  -  Brassinosteroid insensitive 1-associated receptor kinase 1
## VIT_13s0156g00570  -  S-receptor kinase
## VIT_00s0484g00020  -  Receptor kinase ARK3
```

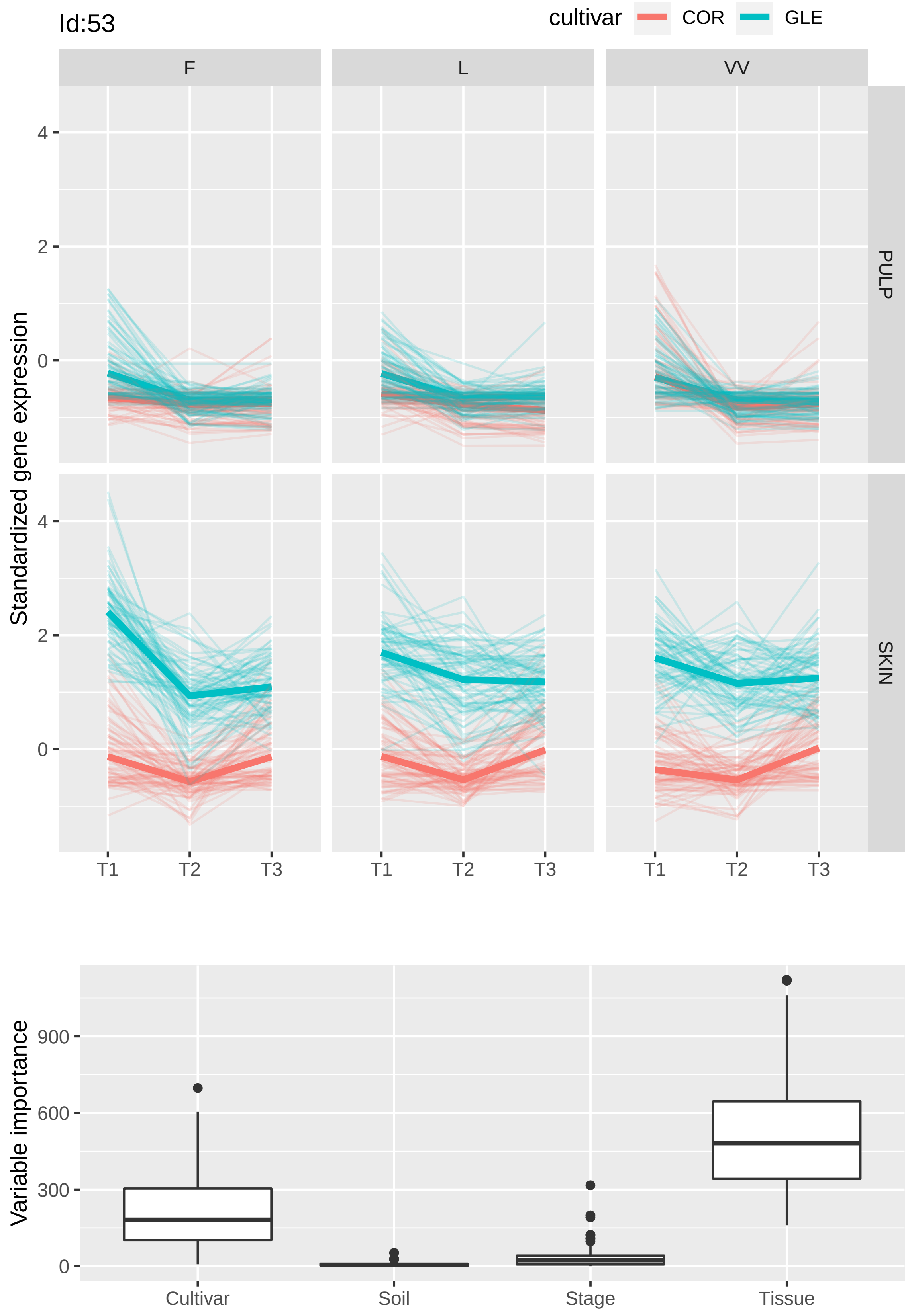
Id:53
cultivar
COR
GLE
F
L
VV
PULP
SKIN
Standardized gene expression
4
2
0
T1
T2
T3
Variable importance
900
600
300
0
Cultivar
Soil
Stage
Tissue

Cluster no. 87

```
## Number of genes in the cluster: 73
## Homogeneity Index:    0.75
## Variable importance for Stage:     Rank =  87  - Median =  23.13
## Variable importance for Cultivar:  Rank =  32  - Median =  107.3
## Variable importance for Tissue:    Rank =  21  - Median =  514.8
## Variable importance for Soil:      Rank =  52  - Median =   5.53
##
## Gene ID                Gene Annotation
## VIT_05s0020g05040  -  Proteinase inhibitor 1 PPI3B2
## VIT_01s0150g00410  -  Unknown protein
## VIT_14s0219g00160  -  Nitrate-responsive NOI protein
## VIT_02s0241g00190  -  No hit
## VIT_13s0019g02100  -  No hit
## VIT_00s0385g00010  -  Linalool synthase (VvTPS59)
## VIT_04s0044g00040  -  Dirigent
## VIT_02s0033g00390  -  VvMybA2
## VIT_07s0005g06000  -  Pore-forming toxin-like protein Hfr-2
## VIT_13s0101g00220  -  Ribosomal RNA 16S
## VIT_02s0033g00380  -  VvMybA2 (C-term)
## VIT_18s0166g00250  -  No hit
## VIT_18s0001g14310  -  flavanone-3-hydroxylase  2 (F3H2) [Vitis vinifera]
## VIT_03s0097g00460  -  Geraniol 10-hydroxylase
## VIT_13s0019g04460  -  Phenylalanine ammonia-lyase 2 (PAL2)
## VIT_01s0011g06650  -  Beta-Ig-H3/fasciclin
## VIT_13s0019g02110  -  fringe-related protein
## VIT_07s0005g01290  -  DnaJ homolog, subfamily C, member 7
## VIT_02s0012g02810  -  CYP76C4
## VIT_07s0005g00480  -  Pectinesterase inhibitor
## VIT_04s0079g00690  -  Glutathione S-transferase (VvGST4)
## VIT_03s0038g00950  -  Auxin-responsive SAUR9
## VIT_08s0007g07930  -  Clavata1 receptor kinase (CLV1)
## VIT_13s0074g00570  -  Gamma-aminobutyric acid transporter
## VIT_13s0067g03820  -  chalcone isomerase  1 [Vitis vinifera] (VvCHI1)
## VIT_16s0039g02230  -  UDP glucose:flavonoid 3-o-glucosyltransferase (VvUFGT) GeneID: 1002330
## VIT_03s0038g00570  -  Unknown protein
## VIT_01s0026g00380  -  CBS domain containing protein
## VIT_03s0017g00870  -  Transferase family / Anthocyanin Acyl-transferase (VvAnAT)
## VIT_07s0005g06090  -  Pore-forming toxin-like protein Hfr-2
## VIT_12s0059g01960  -  Unknown protein
## VIT_01s0026g02400  -  Glutathione S-transferase 10 GSTU10
## VIT_04s0023g00520  -  Auxin responsive SAUR protein
## VIT_01s0010g01260  -  23S ribosomal RNA
## VIT_16s0050g00910  -  MATE efflux family protein (VvAnthoMATE2)
## VIT_19s0015g00040  -  ABC Transporter (VvMRP4 - VvABCC4)
## VIT_19s0090g00400  -  Zinc finger (C3HC4-type ring finger)
## VIT_02s0025g04720  -  Leucoanthocyanidin dioxygenase (VvLDOX) [Vitis vinifera]
## VIT_09s0018g01190  -  Anthranilate N-benzoyltransferase
## VIT_10s0523g00050  -  Extracellular ligand-gated ion channel
## VIT_13s0067g00240  -  Binding
## VIT_09s0054g01520  -  Beta-amyrin synthase
## VIT_07s0005g06110  -  Pore-forming toxin-like protein Hfr-2
## VIT_00s0266g00070  -  Linalool synthase (VvTPS54), (3S)- Linalool syn
## VIT_01s0010g03510  -  Anthocyanin  O-methyltransferase (VvAOMT1)
## VIT_06s0004g01960  -  No hit
```

```
## VIT_14s0068g00920  -  chalcone synthase 2 (CHS2)
## VIT_05s0124g00080  -  No hit
## VIT_06s0004g08150  -  trans-cinnamate 4-monooxygenase (VvC4H)
## VIT_01s0010g03490  -  Caffeoyl-CoA O-methyltransferase (VvAMOT2)
## VIT_06s0004g02620  -  Phenylalanine ammonia-lyase
## VIT_02s0033g00450  -  VvMybA3
## VIT_01s0010g03470  -  Caffeoyl-CoA O-methyltransferase (VvAMOT3)
## VIT_13s0019g02940  -  Cis-zeatin O-beta-D-glucosyltransferase
## VIT_10s0116g00530  -  Thiazole biosynthetic enzyme, chloroplast (ARA6)
## VIT_10s0003g05420  -  S-2-hydroxy-acid oxidase, peroxisomal
## VIT_14s0081g00810  -  No hit
## VIT_05s0136g00260  -  chalcone synthase 3 (CHS3)
## VIT_07s0005g06060  -  Pore-forming toxin-like protein Hfr-2
## VIT_18s0001g09400  -  Cytochrome b5 DIF-F (VvCytoB5)
## VIT_14s0060g01420  -  Unknown protein
## VIT_18s0001g03470  -  Flavonol synthase
## VIT_06s0009g02830  -  Flavonoid 3',5'-hydroxylase
## VIT_17s0000g02680  -  ABA glucosidase
## VIT_01s0010g03460  -  Caffeoyl-CoA O-methyltransferase
## VIT_02s0033g00410  -  VvMybA1
## VIT_00s0388g00010  -  Serine/threonine kinase
## VIT_18s0001g06840  -  Peroxidase GvPx2b class III
## VIT_14s0068g00930  -  chalcone synthase 1 (CHS1)
## VIT_13s0067g02870  -  chalcone isomerase  2 [Vitis vinifera] (VvCHI2)
## VIT_01s0011g03490  -  3-ketoacyl-CoA synthase
## VIT_14s0030g00040  -  Disease resistance protein (CC-NBS-LRR class)
## VIT_18s0072g00160  -  Peroxidase 12
```

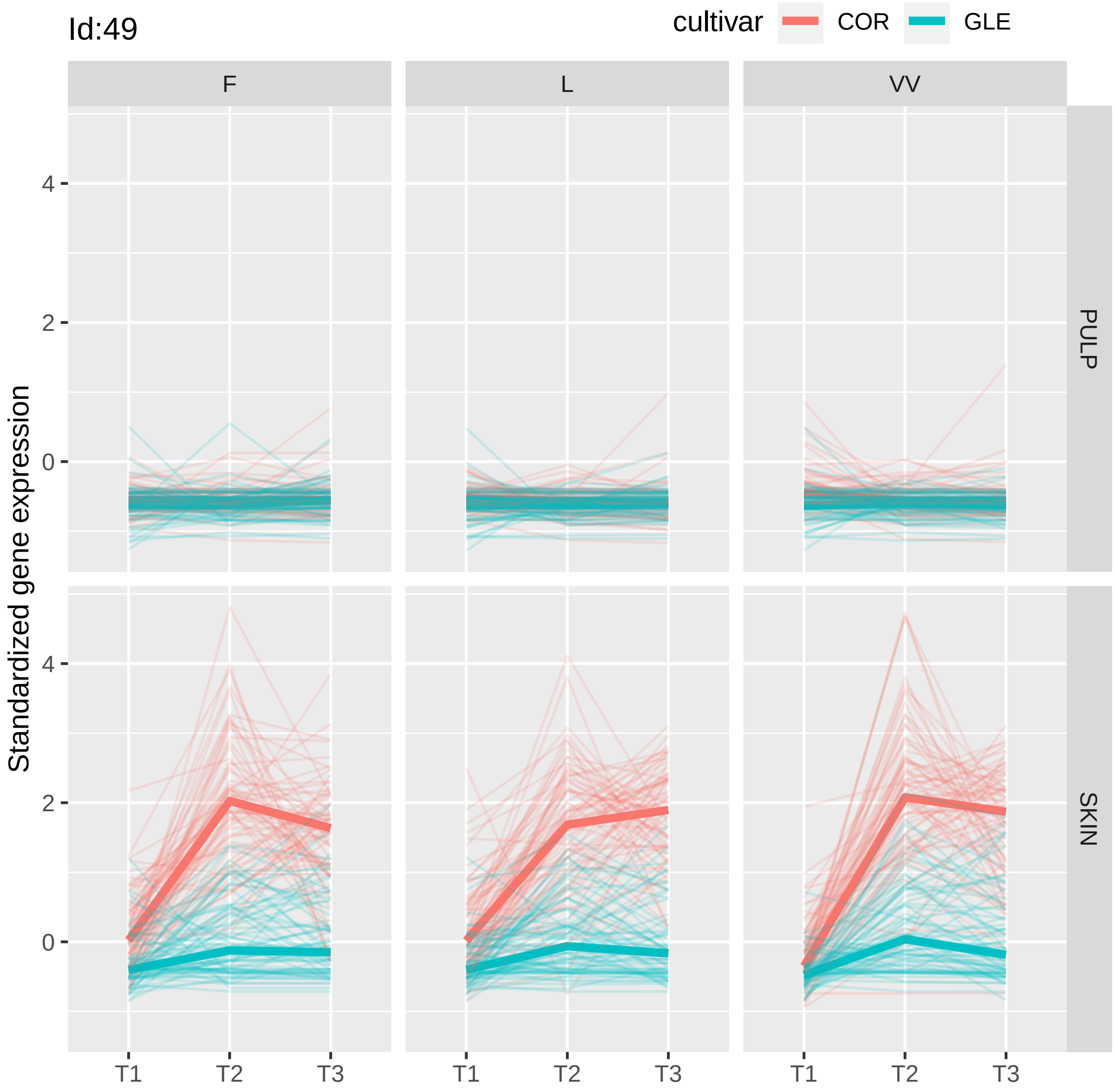
Id:49
cultivar
COR
GLE
F
L
VV
PULP
SKIN
Standardized gene expression
4
2
0
T1
T2
T3

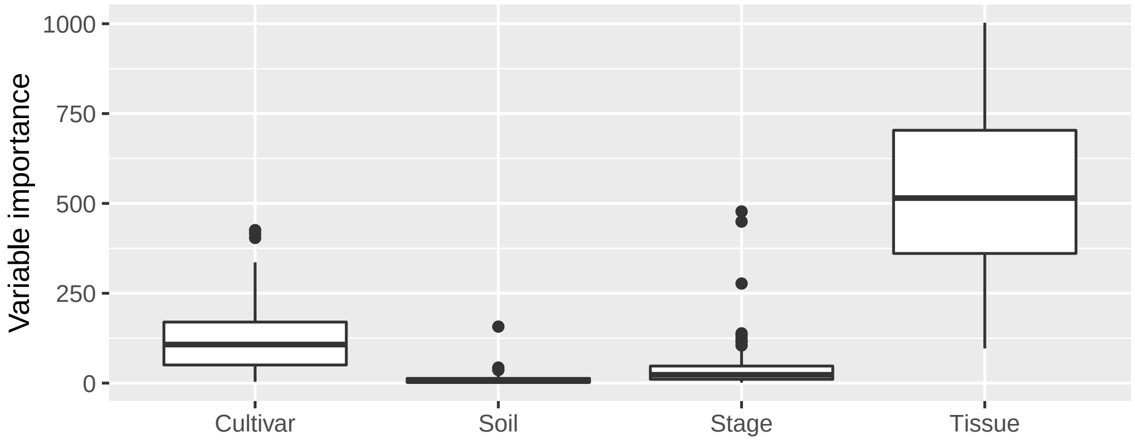
Variable importance
1000
750
500
250
0
Cultivar
Soil
Stage
Tissue

Cluster no. 88

```
## Number of genes in the cluster: 64
## Homogeneity Index:    0.72
## Variable importance for Stage:     Rank =  88  - Median =  21.32
## Variable importance for Cultivar:  Rank =  20  - Median =  198.6
## Variable importance for Tissue:    Rank =  41  - Median =  191.2
## Variable importance for Soil:      Rank =  13  - Median =   8.71
##
## Gene ID                Gene Annotation
## VIT_14s0066g01880  -  Zf AN1 domain-containing stress-associated protein 12 (SAP12)
## VIT_11s0016g05360  -  Phospholipase D alpha 1 precursor (PLD 1) (Choline phosphatase 1)
## VIT_04s0008g00180  -  NADP-dependent malic enzyme
## VIT_11s0037g00910  -  No hit
## VIT_08s0007g04200  -  Late meristem identity1 HB51/LMI1 (VvATHB-5)
## ENSRNA049465017  -
## VIT_18s0001g12740  -  No hit
## VIT_05s0049g00600  -  No hit
## VIT_03s0063g01330  -  Nodulin 1A, senescence-associated
## VIT_05s0049g00680  -  No hit
## VIT_06s0004g06770  -  No hit
## VIT_04s0023g03220  -  Myosin-related
## VIT_03s0063g01310  -  Oxidoreductase, 2OG-Fe(II) oxygenase family
## VIT_05s0049g00620  -  No hit
## VIT_05s0049g00690  -  No hit
## VIT_08s0007g04180  -  Retrotransposon protein, Ty1-copia subclass
## VIT_07s0005g02040  -  Alpha-L-fucosidase 2 precursor
## VIT_18s0001g05380  -  TTL1 (tetratricopetide-repeat thioredoxin-like 1)
## VIT_00s0324g00060  -  UDP-glycosyltransferase 85A8
## VIT_05s0049g00730  -  No hit
## VIT_12s0057g00060  -  No hit
## VIT_19s0093g00590  -  Myosin heavy chain-like protein
## VIT_05s0062g00480  -  Xyloglucan endo-transglycosylase, C-terminal
## VIT_00s0203g00020  -  No hit
## VIT_10s0003g00410  -  MLO6 (mildew resistance locus O 6)
## VIT_18s0001g11630  -  Allene oxide synthase (jasmonates from fatty acids)
## VIT_12s0057g00170  -  Wound-induced
## VIT_04s0008g04600  -  Expansin family protein (EXPR3)
## VIT_18s0164g00100  -  Laccase
## VIT_12s0057g00080  -  Wound-induced
## VIT_02s0025g00240  -  ß-carotene hydroxylase (BCH1) (VvBCH1)
## VIT_03s0063g01290  -  Gibberellin 20 oxidase 2
## VIT_19s0027g01400  -  R protein  PRF  disease resistance protein
## VIT_17s0000g01280  -  WRKY Transcription Factor (VvWRKY52)
## VIT_12s0035g00350  -  AAA-type ATPase
## VIT_12s0057g01510  -  Unknown protein
## VIT_12s0057g00120  -  Wound-induced
## VIT_01s0010g00380  -  FLS2 (flagellin-sensitive 2)
## VIT_07s0031g01160  -  Hyperosmotically inducible periplasmic protein
## VIT_12s0057g00090  -  Wound-induced
## VIT_12s0059g00490  -  Unknown
## VIT_05s0049g01780  -  Caleosin
## VIT_05s0049g00550  -  No hit
## VIT_14s0006g02950  -  Lateral organ boundaries protein 41
## VIT_13s0067g02840  -  Phosphatidylinositol-4-phosphate 5-kinase
## VIT_14s0066g00700  -  Oleosin H-isoform
```

```
## VIT_12s0057g00100  -  Wound-responsive
## VIT_11s0016g02770  -  Meiotic nuclear divisions 1
## VIT_12s0035g00330  -  AAA-type ATPase
## VIT_02s0025g02490  -  Unknown protein
## VIT_18s0001g08200  -  MATE efflux family protein ZF14
## VIT_04s0008g00220  -  IAA6
## VIT_03s0063g01220  -  Nodulin 1A, senescence-associated
## VIT_10s0116g01690  -  No hit
## VIT_12s0057g00140  -  Wound-induced
## VIT_05s0051g00690  -  No hit
## VIT_12s0057g00070  -  Wound-induced
## VIT_02s0234g00110  -  Oleosin OLE-4
## VIT_12s0057g00150  -  Wound-induced
## VIT_14s0006g02970  -  CYP735A1
## VIT_07s0129g00510  -  Unknown
## VIT_09s0096g00960  -  Binding
## VIT_08s0007g04240  -  Late embryogenesis abundant protein
## VIT_00s0231g00080  -  No hit
```

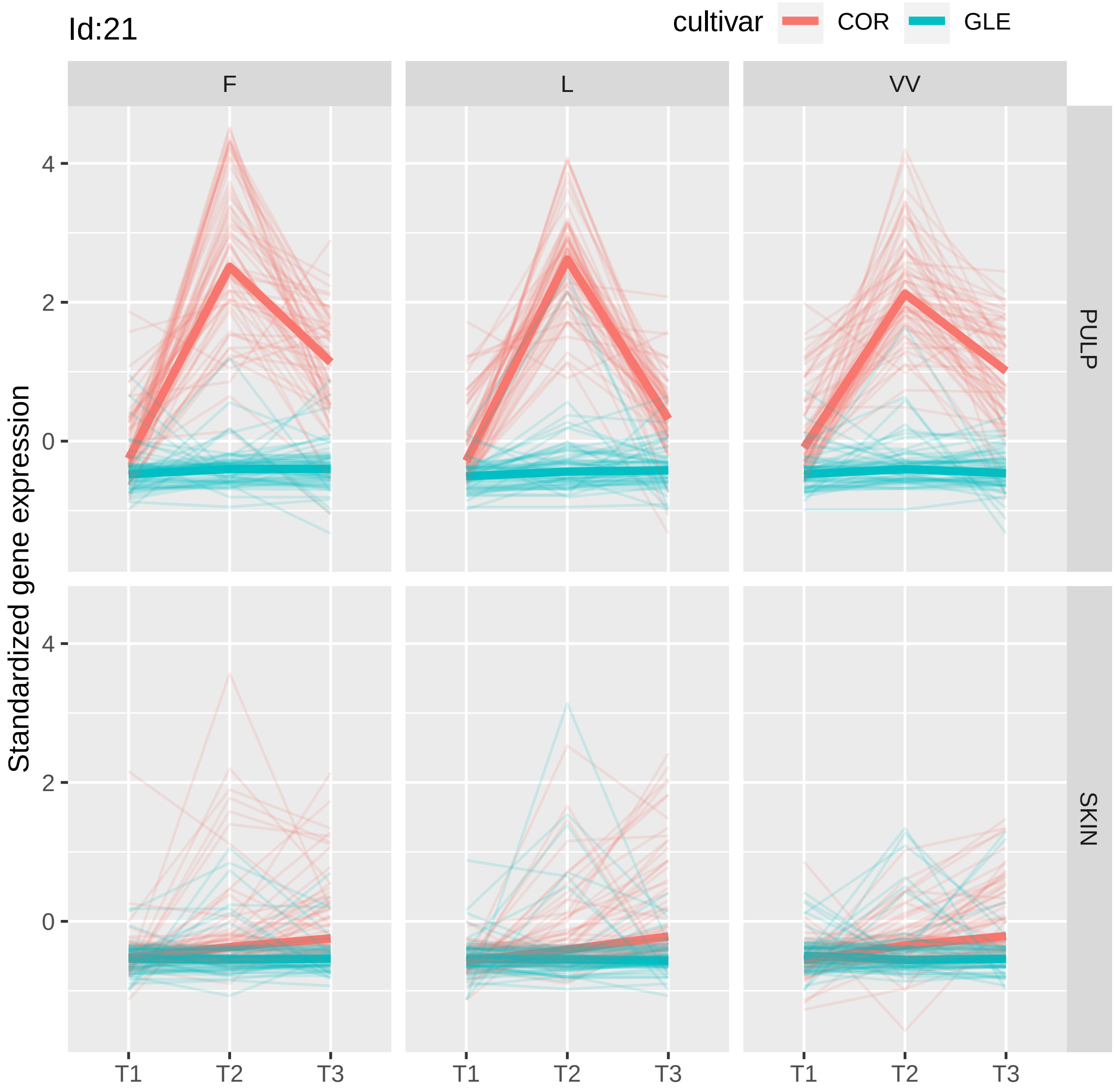
Id:21
cultivar
COR
GLE
F
L
VV
PULP
SKIN
Standardized gene expression
4
2
0
T1
T2
T3

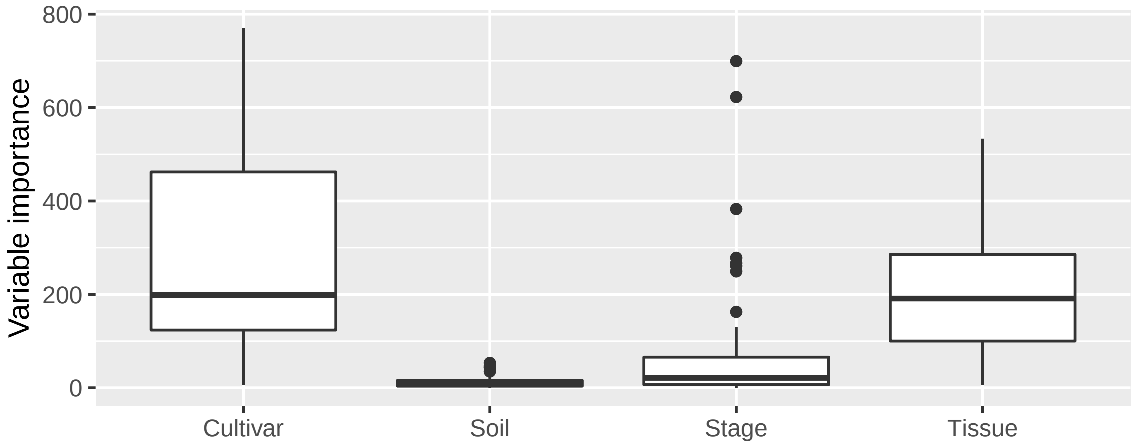
Variable importance
800
600
400
200
0
Cultivar
Soil
Stage
Tissue

Cluster no. 89

```
## Number of genes in the cluster: 138
## Homogeneity Index:    0.82
## Variable importance for Stage:     Rank =  89  - Median =  18.87
## Variable importance for Cultivar:  Rank =  57  - Median =  30.49
## Variable importance for Tissue:    Rank =   3  - Median =  984.5
## Variable importance for Soil:      Rank = 101  - Median =   2.46
##
## Gene ID                Gene Annotation
## VIT_07s0141g00620  -  Bile acid:sodium symporter
## VIT_11s0118g00360  -  Leucoanthocyanidin dioxygenase
## VIT_05s0049g01290  -  Protein phosphatase 2C
## VIT_02s0012g03140  -  Ribosomal protein S7 30S
## VIT_04s0008g04180  -  Arsenite transport protein (ArsB)
## VIT_19s0015g00710  -  Cellulose synthase CSLE1
## VIT_14s0060g01450  -  3'-5' exoribonuclease
## VIT_19s0014g00580  -  Disease resistance protein (NBS-LRR class)
## VIT_09s0002g03370  -  No hit
## VIT_08s0007g01890  -  Unknown protein
## VIT_07s0031g01380  -  ferulate 5-hydroxylase
## VIT_17s0053g00780  -  Homeodomain GLABROUS11
## VIT_03s0038g01320  -  Crp1 protein
## VIT_05s0049g01420  -  Peroxisomal membrane protein PMP22
## VIT_08s0058g00140  -  Ammonium transporter 2
## VIT_09s0002g08460  -  8-amino-7-oxononanoate synthase
## VIT_00s0125g00140  -  SHR5-receptor-like kinase
## VIT_04s0043g00700  -  PTAC3 (plastid transcriptionally active3)
## VIT_05s0051g00670  -  11,12 9-cis epoxycarotenoid dioxygenase (NCED1) (VvNCED1)
## VIT_19s0014g00070  -  Solanesyl diphosphate synthase
## VIT_19s0014g04540  -  S-locus protein kinase
## VIT_07s0005g03230  -  ERF/AP2 Gene Family (VvERF099)
## VIT_12s0142g00330  -  Copper-transporting ATPase PAA1
## VIT_03s0038g03440  -  Alanyl-tRNA synthetase
## VIT_13s0019g04060  -  MLO1
## VIT_17s0000g09270  -  MATE efflux family protein
## VIT_13s0019g04610  -  Salt stress-inducible protein kinase
## VIT_03s0038g04260  -  Chloride channel protein CLC-e
## VIT_13s0084g00560  -  CF2
## VIT_01s0127g00890  -  Dual-specific kinase DSK1
## VIT_00s0477g00040  -  Clathrin assembly protein 16
## VIT_09s0002g03550  -  ABC Transporter (VvPDR3 - VvABCG33)
## VIT_08s0007g05260  -  Glutamate synthase (GLU1), ferredoxin-dependent
## VIT_14s0108g01350  -  Unknown protein
## VIT_14s0108g00540  -  Phosphofructokinase
## VIT_00s1674g00010  -  4-nitrophenylphosphatase
## VIT_05s0077g02270  -  Unknown protein
## VIT_03s0063g02040  -  Caffeine synthase
## VIT_11s0118g00630  -  Unknown protein
## VIT_00s0454g00010  -  Subtilase
## VIT_12s0142g00400  -  Copper-transporting ATPase PAA1
## VIT_06s0004g02670  -  Cyclic nucleotide-gated ion channel 15
## VIT_08s0007g01400  -  Glutathione S-transferase 8 GSTU8
## VIT_14s0068g02240  -  HcrVf1 protein
## VIT_13s0019g01080  -  Protein kinase APK1B
## VIT_19s0014g02770  -  Unknown protein
```

```
## VIT_16s0050g01780  -  Unknown protein
## VIT_06s0004g02290  -  Unknown
## VIT_00s2547g00010  -  WRKY DNA-binding protein 21
## VIT_13s0067g02090  -  Unknown protein
## VIT_13s0067g01120  -  Omega-3 fatty acid desaturase, chloroplast, temperature-sensitive (FAD
## VIT_00s0374g00020  -  S-locus lectin protein kinase
## VIT_00s0194g00270  -  RNA-binding protein P67, Chloroplast
## VIT_18s0001g10180  -  N-hydroxycinnamoyl/benzoyltransferase 3
## VIT_11s0016g03510  -  Elongation factor Ts
## VIT_14s0219g00200  -  Pentatricopeptide (PPR) repeat-containing protein
## VIT_05s0062g00720  -  UDP-glucoronosyl/UDP-glucosyl transferase UGT75C1
## VIT_10s0405g00050  -  Inositol transporter 4
## VIT_15s0046g02390  -  ANTR2 (anion transporter 2)
## VIT_18s0001g12000  -  Geranylgeranyl pyrophosphate synthetase 1, chloroplast precursor
## VIT_19s0027g00020  -  WAG1
## VIT_01s0011g05310  -  CRR3 (chlororespiratory reduction 3)
## VIT_17s0000g07500  -  PTAC2 (plastid transcriptionally active2); binding
## VIT_01s0010g03330  -  Unknown
## VIT_19s0014g00600  -  BED finger-nbs-lrr resistance protein [Populus trichocarpa]
## VIT_18s0166g00260  -  No hit
## VIT_01s0011g00660  -  Pentatricopeptide (PPR) repeat
## VIT_17s0000g05620  -  Integral membrane HPP family protein
## VIT_18s0122g00240  -  Lys Motif-Type Receptor-Like Kinase LYK8
## VIT_00s0231g00040  -  RPM1-interacting protein 4
## VIT_03s0091g00560  -  Leucine-rich repeat protein kinase
## VIT_13s0064g00860  -  Nuclear transcription factor Y subunit A-1
## VIT_19s0014g05080  -  Oxygen evolving enhancer 3 (PsbQ)
## VIT_06s0061g00080  -  Saccharopine dehydrogenase
## VIT_11s0149g00050  -  Hexose transporter 7
## VIT_00s0324g00070  -  Cis-zeatin O-beta-D-glucosyltransferase
## VIT_06s0009g02700  -  F-box family protein
## VIT_00s0992g00020  -  Heat shock protein (HSP26.5-P) 26.5 kDa class P
## VIT_10s0003g03930  -  Inositol transporter 2
## VIT_10s0116g00430  -  Unknown protein
## VIT_19s0014g02270  -  Ribosomal protein L11 methyltransferase
## VIT_19s0090g01870  -  ABC Transporter (VvMRP26 - VvABCC26)
## VIT_05s0020g03290  -  S-receptor kinase
## VIT_16s0148g00240  -  Receptor kinase homolog LRK14
## VIT_16s0039g01900  -  Myb KAN2 (KANADI 2)
## VIT_04s0044g00660  -  S-receptor kinase
## VIT_04s0008g03220  -  Lysophospholipase
## VIT_13s0084g00570  -  CF2
## VIT_00s0207g00070  -  HAD-superfamily hydrolase, subfamily IIA
## VIT_17s0000g09470  -  Nitrate transporter3.1
## VIT_00s0194g00310  -  RNA-binding protein P67, Chloroplast
## VIT_07s0130g00160  -  No hit
## VIT_11s0103g00530  -  Oxysterol binding protein
## VIT_10s0003g04450  -  ABC Transporter (VvMRP18 - VvABCC18)
## VIT_12s0142g00800  -  Leucine-rich repeat protein kinase
## VIT_09s0002g00080  -  Phytosulfokine receptor
## VIT_09s0018g01150  -  No hit
## VIT_13s0019g01110  -  R protein  PRF  disease resistance protein
## VIT_19s0027g00450  -  Pentatricopeptide (PPR) repeat-containing protein
## VIT_02s0025g04320  -  Thaumatin
## VIT_06s0004g01680  -  Unknown protein
```

```
## VIT_05s0062g00820  -  Unknown protein
## VIT_09s0002g03640  -  ABC Transporter (VvPDR7 - VvABCG37)
## VIT_08s0058g00090  -  Hcr9-Avr4-par1
## VIT_19s0093g00050  -  R protein  PRF  disease resistance protein
## VIT_00s0456g00040  -  S-locus protein kinase
## VIT_01s0011g00720  -  WRKY Transcription Factor (VvWRKY01)
## VIT_14s0006g01290  -  myb domain protein 113 (VvMYBA6)
## VIT_13s0067g01270  -  Avr9/Cf-9 rapidly elicited protein 284
## VIT_02s0012g00180  -  Serine protease
## VIT_04s0044g01610  -  CYP82C1p
## VIT_19s0015g00250  -  Zinc transporter ZIP11
## VIT_00s2485g00010  -  CRK10 (cysteine-rich RLK10)
## VIT_11s0016g02310  -  Thioredoxin 2
## VIT_02s0025g01710  -  Unknown
## VIT_14s0030g00680  -  Ribosomal protein S4 [Vitis vinifera]
## VIT_01s0010g03320  -  Kelch repeat-containing F-box protein
## VIT_13s0067g03170  -  Transcription elongation factor S-II
## VIT_14s0108g01250  -  Unknown
## VIT_00s0484g00030  -  ARK3 (Arabidopsis Receptor Kinase 3)
## VIT_11s0016g00040  -  BRI1 (brassinosteroid insensitive 1)
## VIT_01s0127g00070  -  Nitrate transporter2.5
## VIT_06s0004g03590  -  TOE1 (target of eat1 1)  related to apetala2 7
## VIT_15s0046g03090  -  DTA2 (downstream target of AGL15 2)
## VIT_16s0022g00890  -  Invertase/pectin methylesterase inhibitor
## VIT_00s0484g00010  -  ARK3 (Arabidopsis Receptor Kinase 3)
## VIT_05s0020g01090  -  Basic Leucine Zipper Transcription Factor (VvbZIP15)
## VIT_00s0262g00090  -  Receptor kinase RK20-1
## VIT_13s0156g00580  -  S-receptor kinase
## VIT_08s0007g05100  -  Glycosyl hydrolase family 31 protein
## VIT_13s0067g00440  -  Protein kinase Xa21
## VIT_13s0067g00420  -  Protein kinase Xa21
## VIT_08s0056g00800  -  myb domain protein 60 (VvMYB60)
## VIT_18s0001g03910  -  Nitrate reductase 2 (NR2)
## VIT_00s0218g00200  -  UDP-glucuronosyl/UDP-glucosyltransferase
## VIT_15s0046g01280  -  JAR1-like protein
## VIT_00s0253g00090  -  Methyl jasmonate esterase
## VIT_00s0456g00020  -  S-locus lectin protein kinase
```

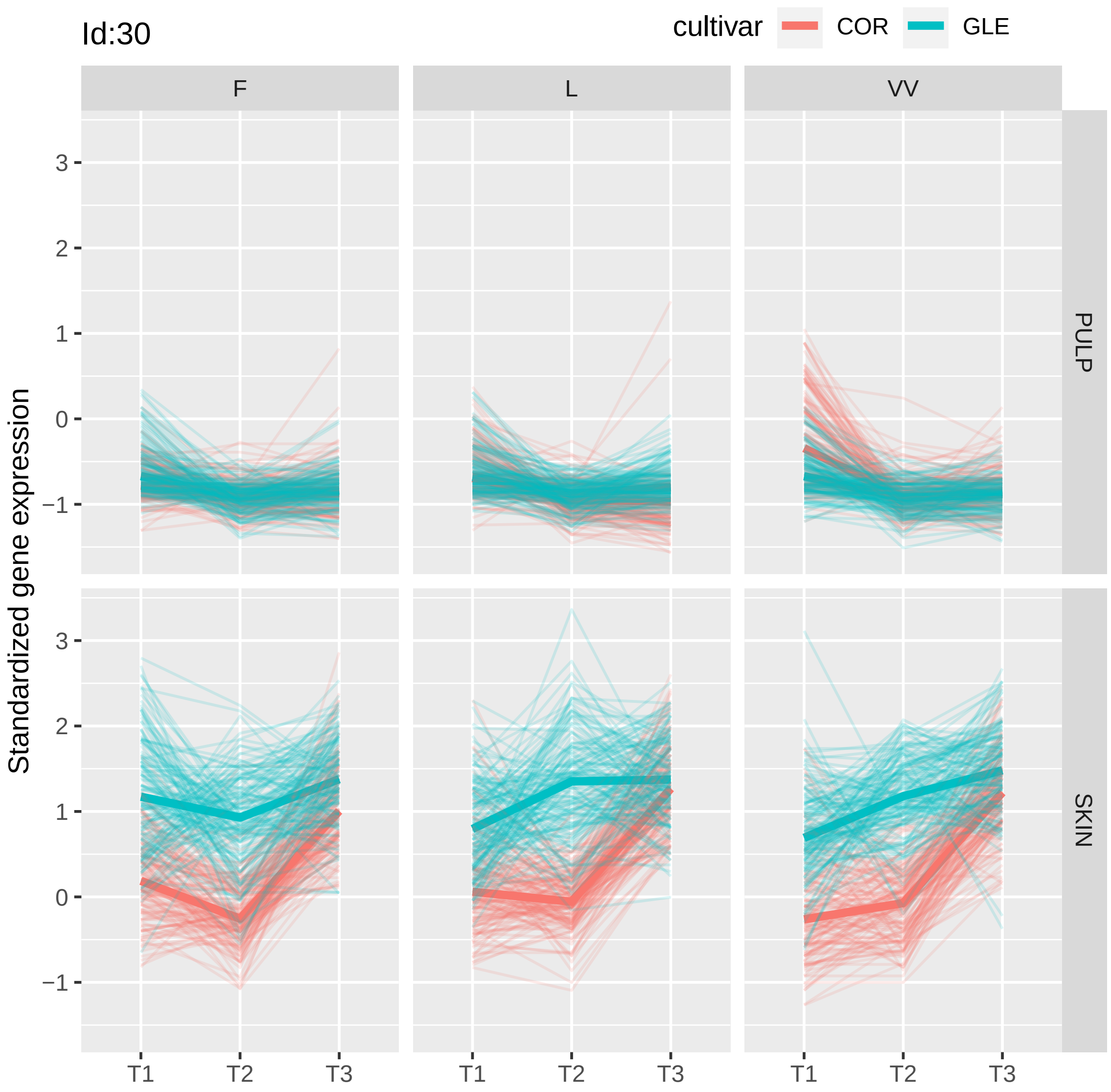

Id:30
cultivar
COR
GLE
F
L
VV
PULP
SKIN
Standardized gene expression
3
2
1
0
−1
T1
T2
T3

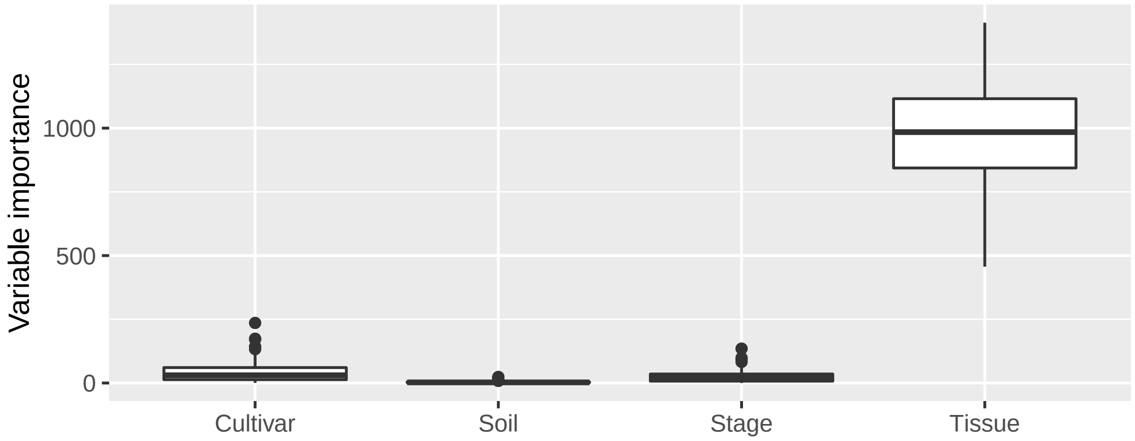

Variable importance
1000
500
0
Cultivar
Soil
Stage
Tissue

Cluster no. 90

```
## Number of genes in the cluster: 71
## Homogeneity Index:    0.73
## Variable importance for Stage:     Rank =  90  - Median =  18.53
## Variable importance for Cultivar:  Rank =  71  - Median =   14.8
## Variable importance for Tissue:    Rank =   1  - Median =  999.9
## Variable importance for Soil:      Rank =  88  - Median =   3.53
##
## Gene ID               Gene Annotation
## VIT_18s0122g00300  -  Unknown protein
## VIT_16s0039g02040  -  4-coumarate-CoA ligase 3
## VIT_00s0504g00010  -  Photosystem II PsbD
## VIT_19s0085g00110  -  ABC transporter B member 11
## VIT_01s0026g00200  -  Xyloglucan endotransglucosylase/hydrolase 28
## VIT_05s0049g00300  -  1-aminocyclopropane-1-carboxylate oxidase homolog 1
## VIT_05s0049g01620  -  Cyclopropane fatty acid synthase
## VIT_12s0059g01870  -  GH3-like protein
## VIT_14s0006g02990  -  3-ketoacyl-CoA synthase
## VIT_17s0000g10060  -  Lipase GDSL
## VIT_10s0003g00620  -  Lipase GDSL
## VIT_01s0011g04840  -  No hit
## VIT_00s0267g00030  -  Unknown
## VIT_12s0059g01590  -  Lipase GDSL
## VIT_03s0038g01830  -  Proline-rich protein 4
## VIT_04s0043g00560  -  DD1A protein
## VIT_01s0011g02360  -  Unknown
## VIT_08s0007g07140  -  Esterase/lipase/thioesterase family protein
## VIT_19s0085g00030  -  Unknown protein
## VIT_11s0103g00760  -  CIA2 (chloroplast import apparatus 2)
## VIT_10s0405g00040  -  Auxin-responsive protein
## VIT_15s0046g02400  -  Glycerol-3-phosphate acyltransferase 8
## VIT_00s0286g00130  -  S-locus lectin protein kinase
## VIT_13s0019g01260  -  Enoyl reductase
## VIT_10s0003g00330  -  HSL1 (HAESA-like 1)
## VIT_15s0046g02070  -  Zinc finger (C3HC4-type ring finger)
## VIT_09s0002g01080  -  Lipoxygenase (VvLOXO)
## VIT_09s0002g03140  -  Lipase GDSL
## VIT_04s0023g00490  -  Auxin responsive SAUR protein
## VIT_18s0122g00780  -  Inorganic phosphate transporter
## VIT_08s0056g00260  -  Harpin-induced protein
## VIT_08s0040g00780  -  P-coumaroyl shikimate 3'-hydroxylase isoform 1
## VIT_09s0002g03130  -  FRK1 (FLG22-induced receptor-like kinase 1)
## VIT_11s0065g00130  -  Taxane 10-beta-hydroxylase
## VIT_14s0128g00550  -  Protein kinase Xa21
## VIT_18s0001g14790  -  Lipase 3 (EXL3) family II extracellular
## VIT_19s0090g01750  -  Unknown protein
## VIT_07s0005g01670  -  No hit
## VIT_00s0218g00170  -  UDP-rhamnose/rhamnosyltransferase
## VIT_04s0008g03930  -  RD22
## VIT_18s0041g00520  -  Proton-dependent oligopeptide transport (POT) family protein
## VIT_18s0001g15130  -  WD40 repeat protein
## VIT_08s0056g01070  -  MATE efflux family protein
## VIT_18s0001g09060  -  Unknown protein
## VIT_17s0000g09360  -  No hit
## VIT_18s0122g01260  -  Protein kinase 1B
```

```
## VIT_15s0046g00490  -  Wax synthase
## VIT_12s0028g00270  -  WRKY Transcription Factor (VvWRKY35)
## VIT_03s0038g04210  -  Phototropin-2
## VIT_14s0030g01950  -  Transferase
## VIT_17s0000g06190  -  myb domain protein 94 (VvMYB30)
## VIT_08s0007g03370  -  Unknown protein
## VIT_02s0012g01660  -  ABC Transporter (VvMDR5 - VvABCB5)
## VIT_14s0006g02600  -  Wall-associated kinase 4
## VIT_08s0007g03990  -  Cellulose synthase CSLA09
## VIT_14s0068g00400  -  Hydrolase, alpha/beta fold family
## VIT_18s0122g01400  -  Exostosin family protein
## VIT_11s0016g04270  -  Hydrolase, alpha/beta fold
## VIT_11s0037g01210  -  Eceriferum 1 (CER1 protein) Sterol desaturase
## VIT_13s0067g03180  -  Unknown protein
## VIT_04s0008g06290  -  Receptor Like Protein 15 RLP15
## VIT_06s0004g01510  -  Lipoxygenase (LOX2) (VvLOXA)
## VIT_17s0000g04480  -  ERF/AP2 Gene Family (VvERF048)
## VIT_08s0058g01210  -  Non-specific lipid transfer protein LTP1
## VIT_12s0057g01440  -  Heavy-metal-associated domain-containing protein
## VIT_14s0066g01210  -  Carbonic anhydrase, chloroplast precursor
## VIT_16s0039g00010  -  ABC Transporter (VvWBC19 - VvABCG19)
## VIT_06s0061g00230  -  ABC Transporter (VvWBC26 - VvABCG26)
## VIT_07s0095g00520  -  S-receptor kinase 1
## VIT_01s0150g00060  -  SOUL heme-binding
## VIT_03s0038g03450  -  Auxin-induced SAUR
```

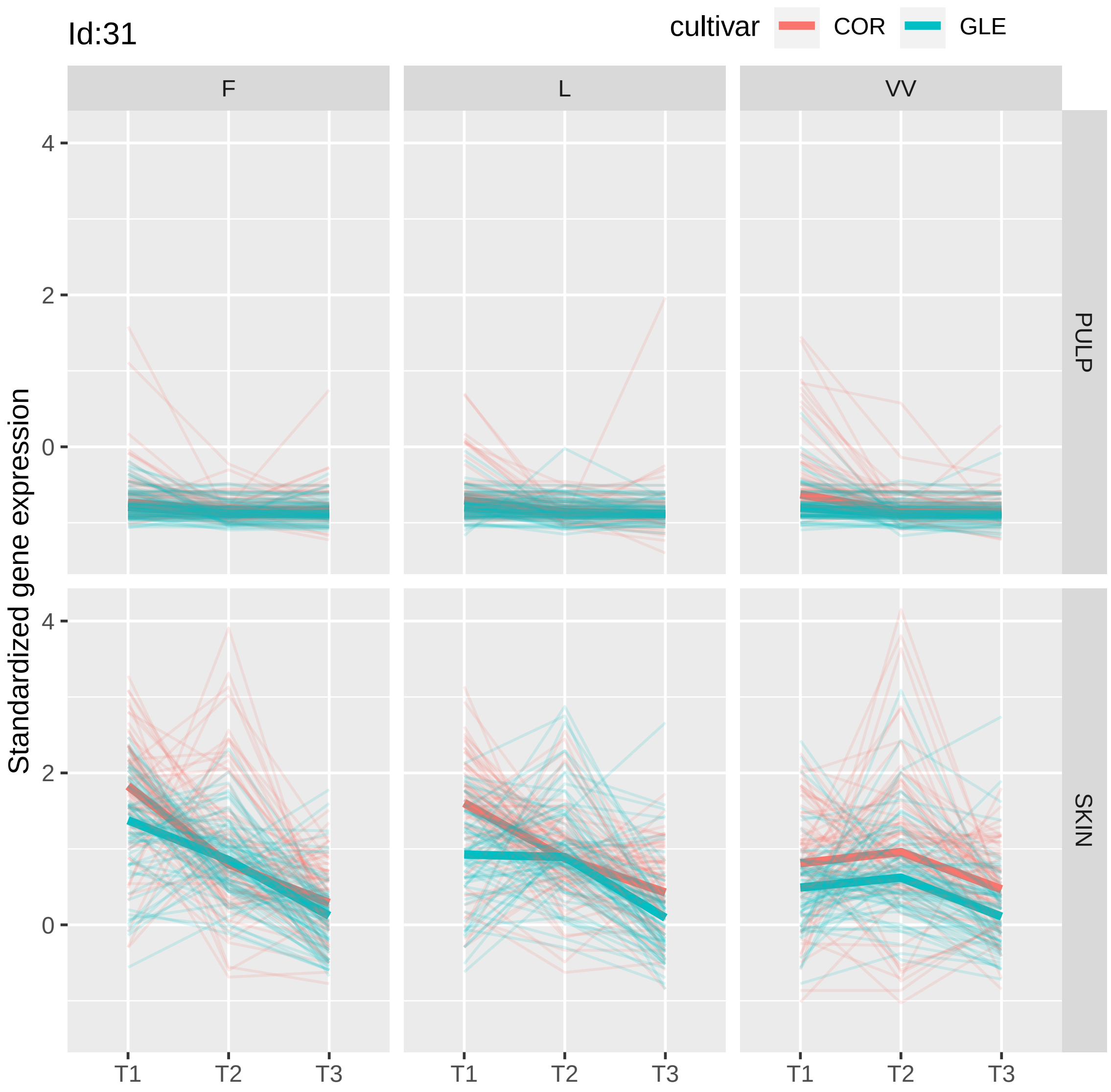
Id:31
cultivar
COR
GLE
F
L
VV
PULP
SKIN
Standardized gene expression
4
2
0
T1
T2
T3

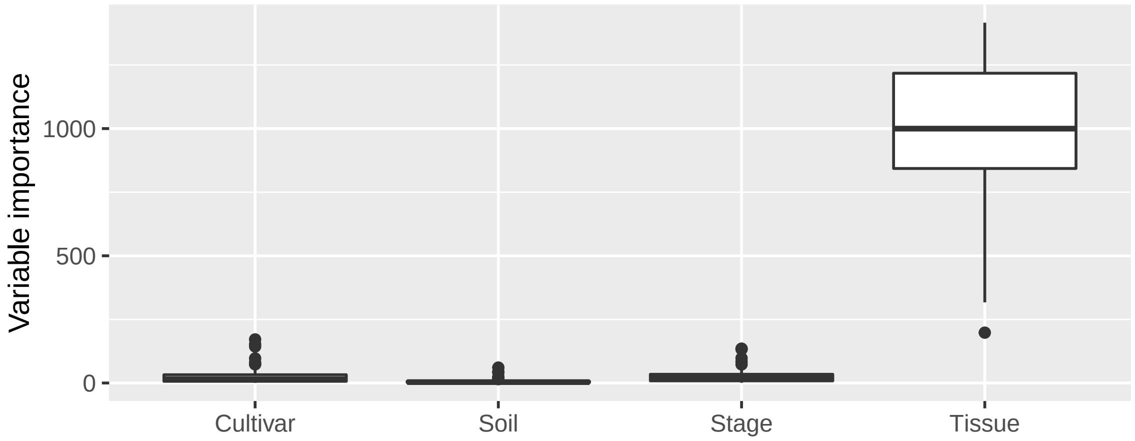
Variable importance
1000
500
0
Cultivar
Soil
Stage
Tissue

Cluster no. 91

```
## Number of genes in the cluster: 77
## Homogeneity Index:    0.71
## Variable importance for Stage:     Rank =  91  - Median =  18.39
## Variable importance for Cultivar:  Rank =  39  - Median =  67.76
## Variable importance for Tissue:    Rank =  14  - Median =  707.8
## Variable importance for Soil:      Rank =  44  - Median =   5.78
##
## Gene ID                Gene Annotation
## VIT_05s0049g01970  -  IAA13
## VIT_03s0038g01410  -  Aquaporin PIP PIP1A
## VIT_15s0048g02970  -  Extensin
## VIT_12s0134g00160  -  Xyloglucan endotransglycosylase/hydrolase 16
## VIT_14s0006g01810  -  Unknown
## VIT_08s0056g01190  -  myb domain protein 105
## VIT_17s0000g08530  -  Boron transporter-like protein 1
## VIT_01s0011g02730  -  ABC Transporter (VvWBC8 - VvABCG8)
## VIT_12s0028g03030  -  Zinc finger (C2H2 type) MAGPIE
## VIT_19s0014g05090  -  Thioredoxin h
## VIT_14s0108g00560  -  Ankyrin repeat
## VIT_18s0001g02220  -  Beta-galactosidase / lactase
## VIT_09s0002g02450  -  Phosphatase
## VIT_18s0001g13210  -  Lectin jacalin
## VIT_00s0762g00020  -  S-locus lectin protein kinase
## VIT_03s0038g04760  -  basic helix-loop-helix (bHLH) family
## VIT_18s0001g10270  -  Basic helix-loop-helix protein SPATULA
## VIT_03s0038g03080  -  Catechol O-methyltransferase (VvOMT4)
## VIT_07s0104g00650  -  No hit
## VIT_05s0020g04260  -  Calcium-transporting ATPase 13 ACA13
## VIT_14s0108g00550  -  No hit
## VIT_15s0024g00730  -  Ankyrin
## VIT_14s0171g00320  -  Kelch repeat-containing protein
## VIT_08s0040g02400  -  Myosin heavy chain
## VIT_11s0016g00070  -  Zinc finger (C3HC4-type ring finger)
## VIT_08s0040g00060  -  Receptor protein kinase
## VIT_04s0079g00280  -  NAC domain-containing protein (VvNAC64)
## VIT_18s0001g14710  -  Ketol-acid reductoisomerase precursor
## VIT_14s0060g02390  -  SRC2/SRC2 (soybean gene regulated BY cold-2)
## VIT_04s0023g01700  -  No hit
## VIT_09s0002g03870  -  RPS5 (resistant to p. syringae 5)
## VIT_04s0079g00610  -  Early nodulin ENOD18 protein
## VIT_07s0005g01090  -  ATP binding protein
## VIT_02s0154g00300  -  Small nuclear ribonucleoprotein Sm D3
## VIT_16s0098g01080  -  Growth-regulating factor 7
## VIT_09s0002g01540  -  Unknown protein
## VIT_01s0011g06600  -  Glutamate decarboxylase
## VIT_11s0016g03570  -  No hit
## VIT_10s0071g00580  -  Zfwd2 protein (ZFWD2)
## VIT_08s0007g04330  -  CHUP1 (chloroplast unusual positioning 1)
## VIT_11s0103g00310  -  Unknown protein
## VIT_01s0011g05870  -  RDR1 (RNA-dependent RNA polymerase 1)
## VIT_13s0067g02070  -  Kelch repeat-containing protein
## VIT_12s0035g00680  -  ACR4 (Arabidopsis CRINKLY4)
## VIT_09s0002g02610  -  Lectin jacalin
## VIT_01s0011g03480  -  Cinnamoyl CoA reductase
```

```
## VIT_00s0131g00050  -  Annexin ANN3
## VIT_18s0001g06160  -  TIR-NBS-LRR-TIR disease resistance protein
## VIT_05s0049g00360  -  2-oxoglutarate-dependent dioxygenase
## VIT_10s0003g05090  -  Wall-associated kinase 1 (WAK1)
## VIT_11s0016g03560  -  Ethylene-responsive protein
## VIT_09s0002g07540  -  VQ motif-containing protein
## VIT_18s0001g10670  -  EF hand
## VIT_00s0199g00080  -  Unknown protein
## VIT_06s0004g04220  -  No hit
## VIT_03s0038g03920  -  Glutamate receptor 2 GLUR2
## VIT_18s0001g07410  -  basic helix-loop-helix (bHLH) family
## VIT_13s0067g01630  -  myb domain protein 65
## VIT_03s0063g01470  -  Steroid nuclear receptor, ligand-binding
## VIT_11s0016g04360  -  Glycosyl hydrolase family 17 protein
## VIT_11s0103g00460  -  No hit
## VIT_07s0005g01680  -  Stachyose synthase
## VIT_05s0051g00540  -  LRR receptor-like kinase 2
## VIT_01s0137g00360  -  Transducin family protein / WD-40 repeat
## VIT_17s0000g07710  -  No hit
## VIT_14s0060g00310  -  No hit
## VIT_19s0090g01650  -  LOP1 TRN1 (LOPPED 1, TORNADO 1)
## VIT_02s0025g01780  -  Cellulose synthase CSLG3
## VIT_09s0002g04840  -  Unknown
## VIT_04s0008g03340  -  No hit
## VIT_18s0001g03260  -  R protein L6
## VIT_06s0004g00960  -  Unknown
## VIT_06s0061g01440  -  Unknown protein
## VIT_15s0024g00440  -  R protein  PRF  disease resistance protein
## VIT_17s0000g03900  -  No hit
## VIT_07s0130g00220  -  Peroxidase ATP32, class III
## VIT_05s0020g03200  -  Spermine synthase
```

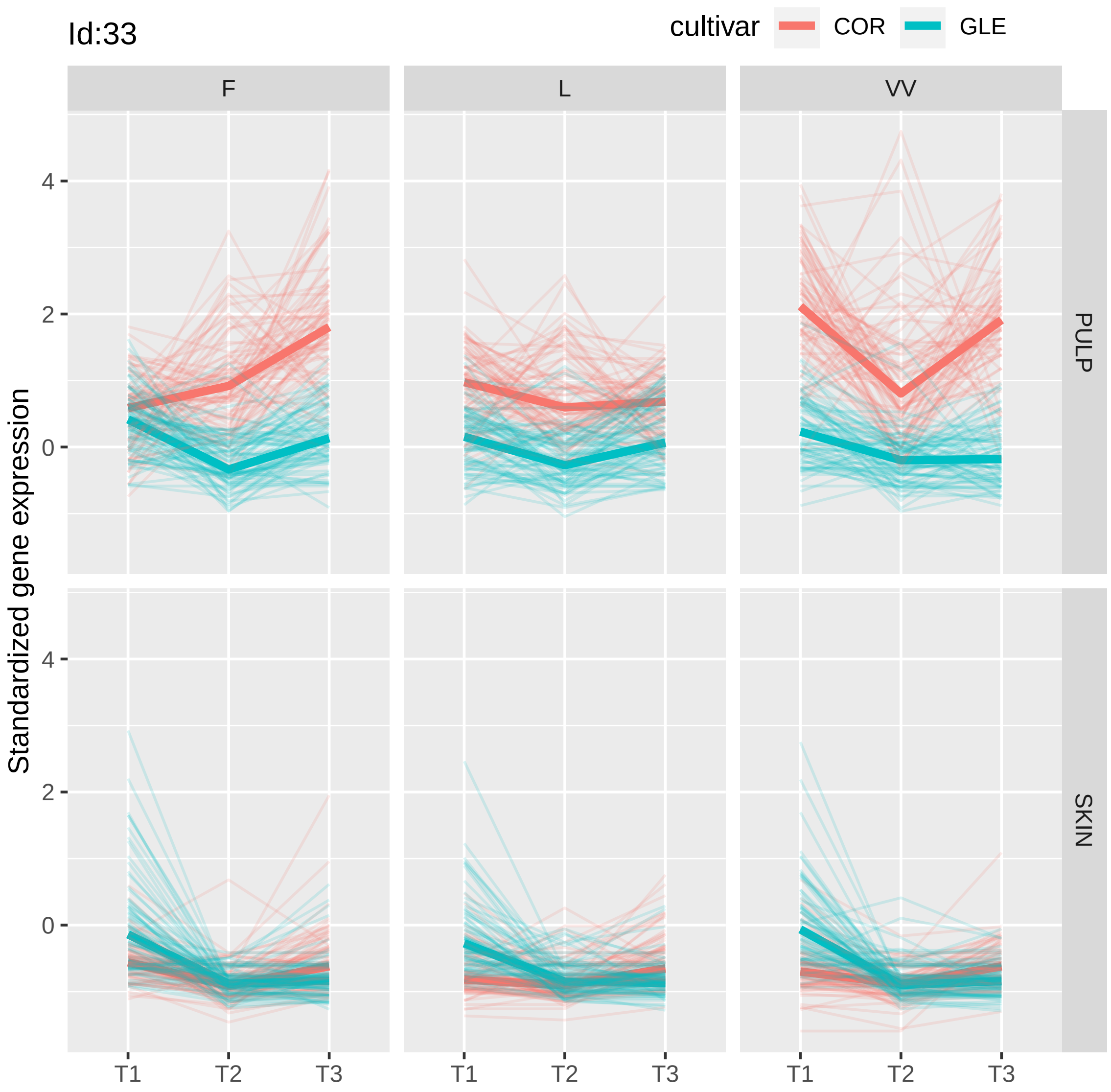
Id:33
cultivar
COR
GLE
F
L
VV
PULP
SKIN
Standardized gene expression
4
2
0
T1
T2
T3

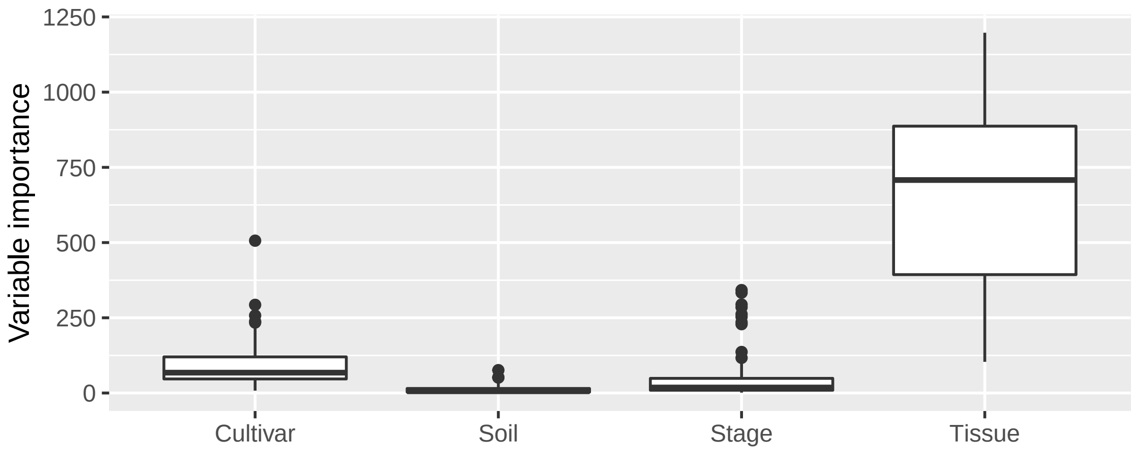
Variable importance
1250
1000
750
500
250
0
Cultivar
Soil
Stage
Tissue

Cluster no. 92

```
## Number of genes in the cluster: 61
## Homogeneity Index:    0.62
## Variable importance for Stage:     Rank =  92  - Median =  18.08
## Variable importance for Cultivar:  Rank =   6  - Median =  824.6
## Variable importance for Tissue:    Rank =  78  - Median =  28.58
## Variable importance for Soil:      Rank =  49  - Median =   5.65
##
## Gene ID                Gene Annotation
## VIT_13s0019g02820  -  Heat shock protein 18.2 kDa class I
## VIT_13s0019g02760  -  Heat shock protein 17.6 kDa class I
## VIT_02s0154g00490  -  Heat shock 22 kDa protein mitochondrial
## VIT_03s0038g02020  -  Amidase
## VIT_12s0055g00930  -  Heavy-metal-associated domain-containing protein
## VIT_16s0022g01640  -  Receptor serine/threonine kinase
## VIT_14s0108g00630  -  Amino acid permease
## VIT_04s0069g00860  -  Sarcosine oxidase
## VIT_02s0241g00180  -  UDP-D- glcucuronate  4-epimerase 5 GAE5
## VIT_07s0031g01660  -  CYP86A2
## VIT_13s0158g00250  -  No hit
## VIT_03s0017g01440  -  Berberine bridge enzyme
## VIT_00s0160g00090  -  TIR-NBS-LRR-TIR disease resistance protein
## VIT_12s0035g01930  -  No hit
## VIT_00s0184g00230  -  No hit
## VIT_03s0038g03600  -  Serine/threonine kinase
## VIT_03s0063g00820  -  Carboxyesterase 12; CXE12
## VIT_04s0008g01560  -  Small molecular heat shock protein 17.5
## VIT_00s0184g00120  -  R protein L6
## VIT_09s0002g06790  -  Small heat shock protein (HSP26.5-P) 26.5 kDa class I
## VIT_08s0105g00380  -  Leucoanthocyanidin dioxygenase
## VIT_00s1955g00010  -  No hit
## VIT_04s0008g01580  -  Heat shock protein 17.6 kDa class II
## VIT_05s0165g00160  -  Ankyrin repeat
## VIT_15s0046g00070  -  PBP1 (pinoid-binding protein 1)
## VIT_09s0002g04280  -  Dynein light chain LC6, flagellar outer arm
## VIT_18s0001g06490  -  Aquaporin PIP1B
## VIT_01s0026g01410  -  Inorganic pyrophosphatase
## VIT_15s0046g02380  -  CYP86A8
## VIT_09s0070g00260  -  No hit
## VIT_09s0002g05840  -  RPS5 (resistant to p. syringae 5)
## VIT_15s0046g02670  -  No hit
## VIT_12s0059g02240  -  Unknown
## VIT_00s0367g00010  -  No hit
## VIT_14s0030g02280  -  Pentatricopeptide (PPR) repeat-containing protein
## VIT_04s0023g02150  -  Phosphatidic acid phosphatase / PAP2
## VIT_19s0014g04190  -  ARK3 (Arabidopsis Receptor Kinase 3)
## VIT_13s0047g00860  -  Replication protein
## VIT_00s0335g00010  -  R protein L6
## VIT_06s0009g03080  -  Histone deacetylase (HD2A)
## VIT_00s0335g00020  -  TIR-NBS-LRR disease resistance
## VIT_14s0060g02460  -  Unknown
## VIT_14s0060g01990  -  ATPP2-B15 (Phloem protein 2-B15)
## VIT_17s0000g01160  -  Nodulin
## VIT_18s0001g06480  -  Aquaporin PIP PIP1;4
## VIT_19s0090g00570  -  Salicylic acid-induced fragment 1 protein
```

```
## VIT_00s0532g00020  -  Kinesin family member C1
## VIT_04s0008g04590  -  CXE carboxylesterase
## VIT_19s0015g01450  -  DJ-1 family protein
## VIT_12s0142g00530  -  Gamma-glutamyltranspeptidase
## VIT_02s0109g00440  -  No hit
## VIT_09s0002g05990  -  6-4 photolyase
## VIT_18s0001g00510  -  Prolyl endopeptidase
## VIT_11s0016g04900  -  Unknown
## VIT_04s0044g00190  -  Mannitol dehydrogenase
## VIT_07s0255g00090  -  CYP82C1p
## VIT_09s0002g05360  -  ABC transporter g family pleiotropic drug resistance 12 PDR12
## VIT_19s0027g01480  -  Phenylalanyl-tRNA synthetase class IIc
## VIT_08s0007g03740  -  DEAD box RNA helicase (PRH75)
## VIT_18s0089g00600  -  R protein L6
## VIT_11s0016g03250  -  Lachrymatory factor synthase
```

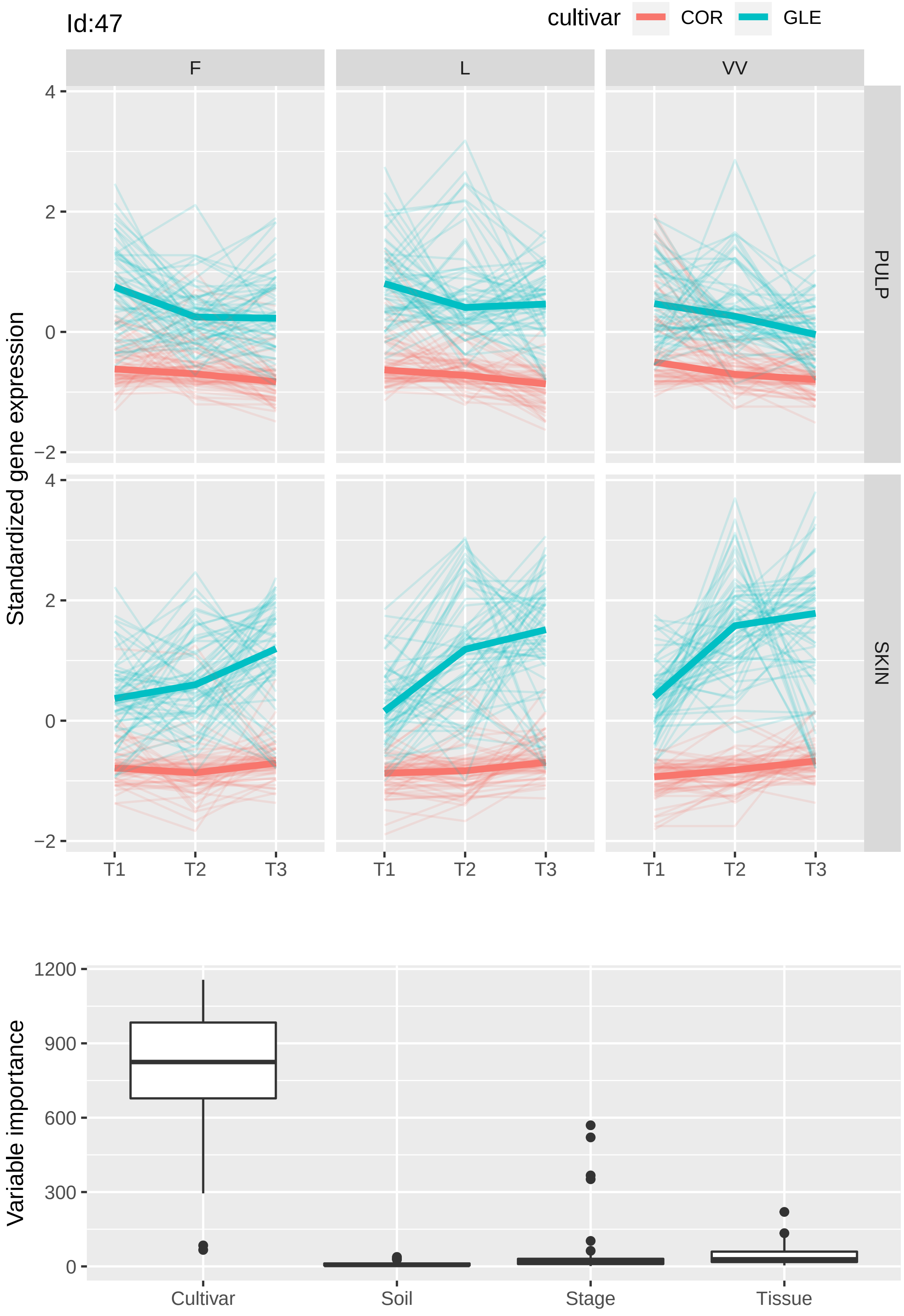

Id:47
cultivar
COR
GLE
F
L
VV
PULP
SKIN
Standardized gene expression
4
2
0
−2
T1
T2
T3
Variable importance
1200
900
600
300
0
Cultivar
Soil
Stage
Tissue

Cluster no. 93

```
## Number of genes in the cluster: 70
## Homogeneity Index:    0.75
## Variable importance for Stage:     Rank =  93  - Median =  16.75
## Variable importance for Cultivar:  Rank =   7  - Median =  743.7
## Variable importance for Tissue:    Rank =  58  - Median =  92.69
## Variable importance for Soil:      Rank =  91  - Median =   3.38
##
## Gene ID               Gene Annotation
## VIT_04s0008g05130  -  TSO1 (chinese for 'ugly')
## VIT_19s0090g00260  -  R protein disease resistance protein
## VIT_06s0004g06320  -  Anthranilate synthase component I-1, chloroplast precursor
## VIT_00s0173g00260  -  ATP binding
## VIT_09s0002g01170  -  Hydrolase, alpha/beta fold family protein
## VIT_10s0116g01340  -  Copper-binding family protein
## VIT_04s0008g05140  -  TSO1 (chinese for 'ugly')
## VIT_11s0037g00950  -  S-adenosylmethionine decarboxylase proenzyme
## VIT_17s0000g06630  -  Unknown protein
## VIT_18s0001g13240  -  Thioredoxin H-type 1
## VIT_11s0037g00940  -  No hit
## VIT_13s0019g02910  -  Cis-zeatin O-beta-D-glucosyltransferase
## VIT_08s0032g01100  -  Acyl-CoA oxidase ACX6
## VIT_19s0014g03720  -  Unknown protein
## VIT_05s0020g01080  -  Unknown
## VIT_09s0002g03980  -  RPS5 (resistant to p. syringae 5)
## VIT_07s0005g03140  -  Aspartic Protease (VvAP15)
## VIT_01s0150g00510  -  Protein phosphatase 2C
## VIT_12s0035g01770  -  DNA (cytosine-5)-methyltransferase AthI
## VIT_15s0024g01750  -  Protein kinase
## VIT_07s0005g01110  -  Aminotransferases class I pyridoxal-phosphate-binding site
## VIT_13s0158g00260  -  R protein MLA10
## VIT_04s0023g03040  -  Lactoylglutathione lyase
## VIT_00s0279g00100  -  No hit
## VIT_16s0050g00870  -  Acyltransferase
## VIT_08s0058g01030  -  Saposin B domain-containing protein
## VIT_03s0038g02880  -  Unknown
## VIT_02s0025g00500  -  Unknown protein
## VIT_16s0013g01180  -  No hit
## VIT_13s0019g05200  -  MATE efflux family protein
## VIT_00s0531g00040  -  Alkylated DNA repair protein
## VIT_14s0006g02140  -  Cis-zeatin O-beta-D-glucosyltransferase
## VIT_04s0043g00960  -  Tetratricopeptide repeat (TPR)-containing
## VIT_00s0269g00020  -  Unknown protein
## VIT_00s0188g00190  -  Auxilin
## VIT_09s0002g02010  -  Myosin-like protein XIK
## VIT_01s0011g01740  -  Lectin
## VIT_05s0094g00180  -  No hit
## VIT_00s0160g00210  -  Myosin heavy chain
## VIT_13s0019g04900  -  Pectate lyase
## VIT_18s0041g02180  -  LIM domain containing protein-like
## VIT_07s0005g01030  -  Cellulose synthase CSLD5
## VIT_01s0011g05060  -  Major latex-like protein 34
## VIT_19s0015g02680  -  Glutathione S-transferase 25 GSTU25
## VIT_11s0016g05680  -  RAB GTPase activator
## VIT_06s0004g02130  -  No hit
```

```
## VIT_18s0089g01140  -  Wall-associated kinase
## VIT_03s0132g00310  -  Myosin-like protein XIK
## VIT_12s0035g01760  -  DNA (cytosine-5)-methyltransferase AthI
## VIT_13s0073g00050  -  Cationic amino acid transporter 6
## VIT_13s0074g00640  -  Unknown protein
## VIT_18s0041g00570  -  Proton-dependent oligopeptide transport (POT) family protein
## VIT_11s0037g01390  -  Acyl-CoA oxidase ACX3
## VIT_04s0079g00810  -  Cold induced protein
## VIT_09s0002g04520  -  No hit
## VIT_15s0046g03600  -  (+)-neomenthol dehydrogenase
## VIT_18s0122g01180  -  Unknown
## VIT_18s0117g00400  -  TIR-NBS-LRR-TIR disease resistance protein
## VIT_14s0219g00130  -  PQ-loop repeat / transmembrane
## VIT_18s0122g00550  -  Cyclin-dependent kinase B2;1
## VIT_01s0026g01570  -  Nitrate transporter 1:2
## VIT_07s0191g00150  -  LMBR1 integral membrane protein
## VIT_13s0139g00200  -  Orotate phosphoribosyltransferase
## VIT_09s0002g03800  -  RPS5 (resistant to p. syringae 5)
## VIT_00s0269g00100  -  Ankyrin
## VIT_14s0006g02850  -  BEE1 (BR ENHANCED EXPRESSION 1)
## VIT_18s0041g00560  -  Proton-dependent oligopeptide transport (POT) family protein
## VIT_03s0038g00220  -  Purple acid phosphatase 10 ATPAP10/PAP10
## VIT_14s0006g02820  -  Mannose-6-phosphate isomerase
## VIT_16s0022g00940  -  Pectinesterase PME3
```

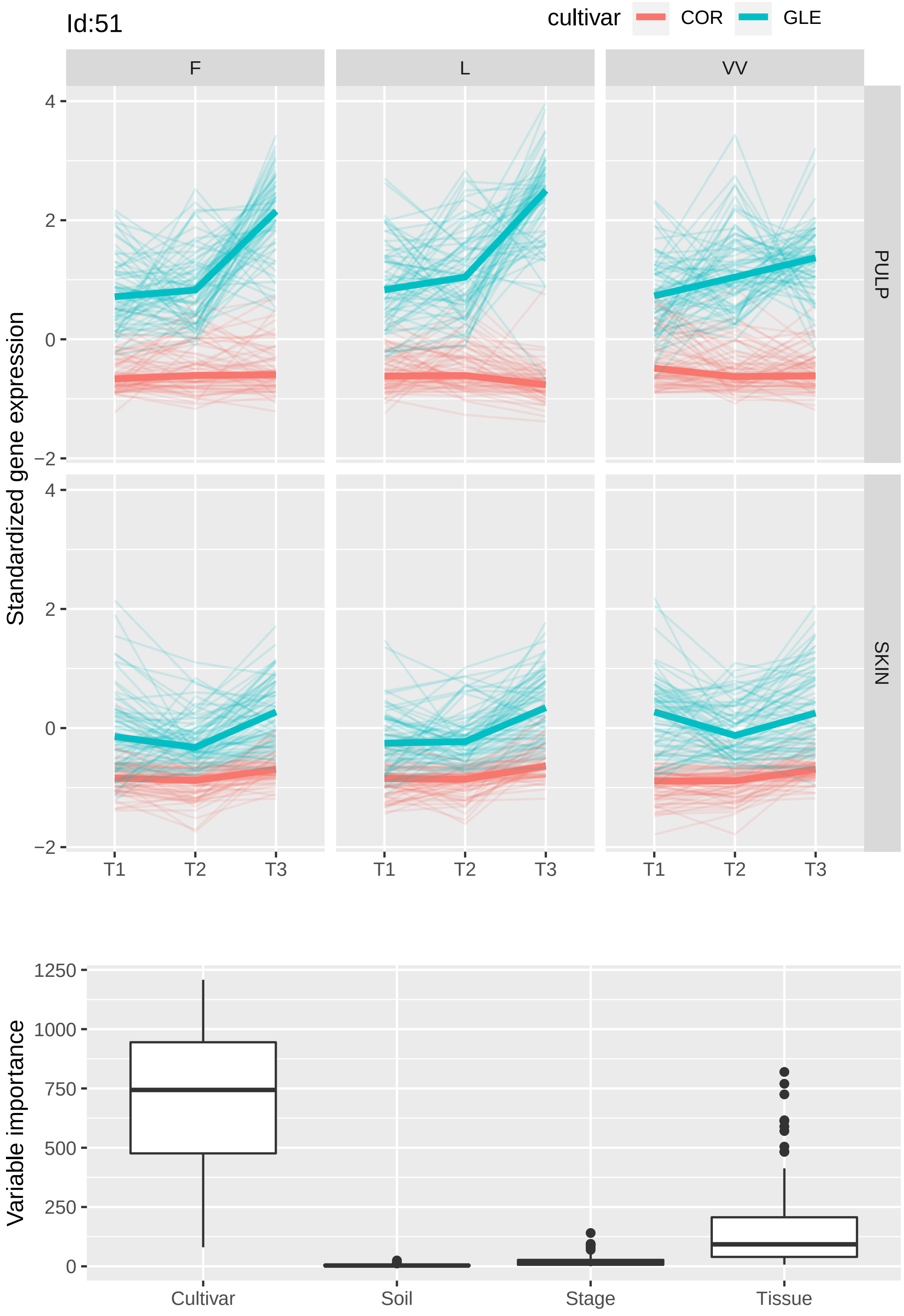
Id:51
cultivar
COR
GLE
F
L
VV
PULP
SKIN
Standardized gene expression
4
2
0
−2
T1
T2
T3
Variable importance
1250
1000
750
500
250
0
Cultivar
Soil
Stage
Tissue

Cluster no. 94

```
## Number of genes in the cluster: 95
## Homogeneity Index:    0.81
## Variable importance for Stage:     Rank =  94  - Median =  15.96
## Variable importance for Cultivar:  Rank =  11  - Median =    516
## Variable importance for Tissue:    Rank =  40  - Median =  192.1
## Variable importance for Soil:      Rank =  50  - Median =   5.58
##
## Gene ID                Gene Annotation
## VIT_01s0011g02020  -  2-oxo acid dehydrogenase, lipoyl-binding site
## VIT_04s0043g01000  -  Oligouridylate binding protein 1B UBP1B
## VIT_08s0007g06680  -  PMR5 (powdery mildew resistant 5)
## VIT_15s0046g01530  -  Intracellular protein transport protein USO1
## VIT_14s0066g02550  -  H(+)-ATPase 4 AHA4
## VIT_15s0048g01090  -  SPX (SYG1/Pho81/XPR1) domain-containing protein
## VIT_04s0079g00660  -  SET Domain group 40
## VIT_04s0079g00650  -  SET Domain group 40
## VIT_18s0122g00590  -  DNA-binding protein
## VIT_17s0000g06360  -  Expansin  (VvEXPA17)
## VIT_04s0023g01720  -  SEC14 cytosolic factor, putative
## VIT_04s0008g03350  -  Unknown protein
## VIT_14s0081g00560  -  WRKY Transcription Factor (VvWRKY42)
## VIT_00s0222g00030  -  No hit
## VIT_06s0004g04440  -  Osmotin
## VIT_05s0020g00050  -  No hit
## VIT_03s0097g00470  -  ATHVA22A (Arabidopsis thaliana HVA22 homologue A)
## VIT_19s0090g00690  -  Unknown protein
## VIT_11s0065g01160  -  Translation initiation factor eIF-2B epsilon subunit
## VIT_18s0075g00080  -  Anaphase-promoting complex component APC1
## VIT_12s0055g00680  -  Leucine-rich repeat protein kinase
## VIT_12s0034g02250  -  Disease resistance protein (NBS class)
## VIT_00s0378g00010  -  Disease resistance protein (NBS class)
## VIT_15s0048g00120  -  Unknown
## VIT_06s0080g01060  -  Calcium-binding EF hand
## VIT_16s0013g01930  -  Acid phosphatase
## VIT_17s0000g01230  -  putative MADS-box TM8a (VviTM8a)
## VIT_18s0001g00780  -  No hit
## VIT_13s0067g01750  -  Receptor protein kinase
## VIT_14s0060g01980  -  Unknown protein
## VIT_05s0049g00150  -  SWI/SNF matrix-associated regulator of chromatin sbfamily A mber 3 3
## VIT_18s0041g01940  -  Cation exchanger (CAX10)
## VIT_08s0007g08330  -  Polygalacturonase PG1
## VIT_08s0007g00170  -  DPB-1 transcription factor (DPB)
## VIT_12s0059g01990  -  No hit
## ENSRNA049996344  -
## VIT_17s0000g05100  -  No hit
## VIT_16s0098g00270  -  No hit
## VIT_11s0016g02760  -  No hit
## VIT_07s0141g00470  -  RNA polymerase Rpb7 N-terminal domain-containing
## VIT_18s0001g06230  -  No hit
## VIT_04s0008g01220  -  RKL1 (Receptor-like kinase 1)
## VIT_12s0059g01080  -  Acid phosphatase/vanadium-dependent haloperoxidase
## VIT_00s0231g00070  -  Auxin-responsive protein
## VIT_05s0020g00060  -  Unknown
## VIT_14s0006g00060  -  No hit
```

```
## VIT_17s0000g03090  -  R protein disease resistance protein
## VIT_06s0004g06500  -  Villin
## VIT_09s0002g05080  -  Kelch repeat-containing F-box family protein
## VIT_01s0127g00590  -  Protein disulfide isomerase
## VIT_05s0020g00210  -  No hit
## VIT_08s0040g02610  -  Unknown
## VIT_05s0049g00210  -  Lesion inducing protein
## VIT_07s0031g02380  -  No hit
## VIT_11s0016g04490  -  IAA16
## VIT_16s0039g00720  -  Folate-biopterin transporter
## VIT_09s0002g05040  -  RPS5 (resistant to p. syringae 5)
## VIT_03s0038g04480  -  SEC14 cytosolic factor
## VIT_03s0180g00210  -  Myb domain protein R1
## VIT_01s0026g01020  -  Binding
## VIT_07s0104g00350  -  Circadian clock coupling factor ZGT
## VIT_18s0075g00090  -  Unknown
## VIT_04s0044g00250  -  Beta-cyanoalanine synthase 1
## VIT_15s0048g02300  -  NAC domain-containing protein (VvNAC53)
## VIT_03s0038g04390  -  Dehydrin 1
## VIT_12s0142g00590  -  R protein L6
## VIT_03s0038g04710  -  Isoflavone reductase (synthesis of phytoalexines)
## VIT_04s0008g03490  -  Ankyrin repeat protein
## VIT_02s0012g01680  -  Trehalose phosphatase 5
## VIT_13s0067g01740  -  Receptor protein kinase
## VIT_00s0207g00170  -  Allergen V5/Tpx-1
## VIT_16s0098g01350  -  Oligopeptide transporter 6
## ENSRNA049470318  -
## VIT_13s0084g00080  -  MAP65/ASE1; t-snare
## VIT_07s0031g00700  -  Caltractin / centrin
## VIT_17s0000g07670  -  Betaine-aldehyde dehydrogenase
## VIT_13s0073g00670  -  Unknown protein
## VIT_09s0054g00640  -  N-acetyltransferase ESCO1
## VIT_04s0023g01680  -  No hit
## VIT_00s0222g00100  -  CC-NBS-LRR class
## VIT_06s0004g06240  -  Zinc finger (C2H2 type) family
## VIT_17s0000g01620  -  Uridine/cytidine kinase 1
## VIT_12s0034g00770  -  TIR-NBS-LRR-TIR disease resistance protein
## VIT_14s0171g00150  -  Avr9/Cf-9 rapidly elicited protein 57
## VIT_13s0106g00770  -  SEC13
## VIT_14s0006g01930  -  Unknown protein
## VIT_09s0002g05820  -  Unknown
## VIT_16s0022g02520  -  ZFWD1 (zinc finger WD40 repeat protein 1)
## VIT_08s0007g02570  -  CCR4-NOT transcription complex subunit 7
## VIT_15s0046g01870  -  Proline-rich family protein
## VIT_06s0004g06880  -  Ran-binding protein 1 RanBP1
## VIT_15s0046g00190  -  Laccase
## VIT_12s0028g00560  -  Unknown
## VIT_11s0206g00040  -  Diadenosine tetraphosphatase
## VIT_18s0089g01030  -  ERF/AP2 Gene Family (VvERF017),Dehydration Responsive Element-Binding
```

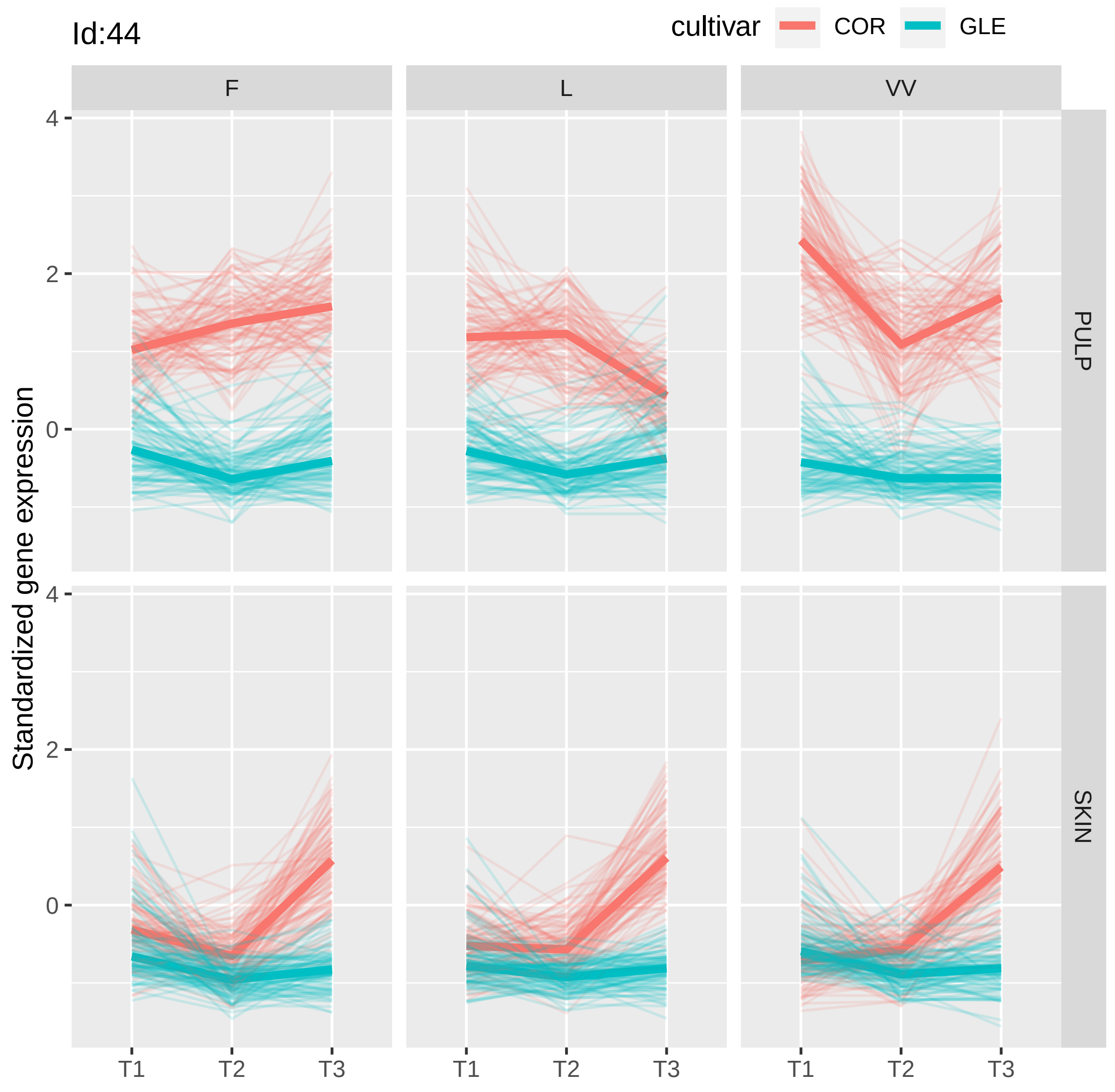
Id:44
cultivar
COR
GLE
F
L
VV
PULP
SKIN
Standardized gene expression
4
2
0
T1
T2
T3

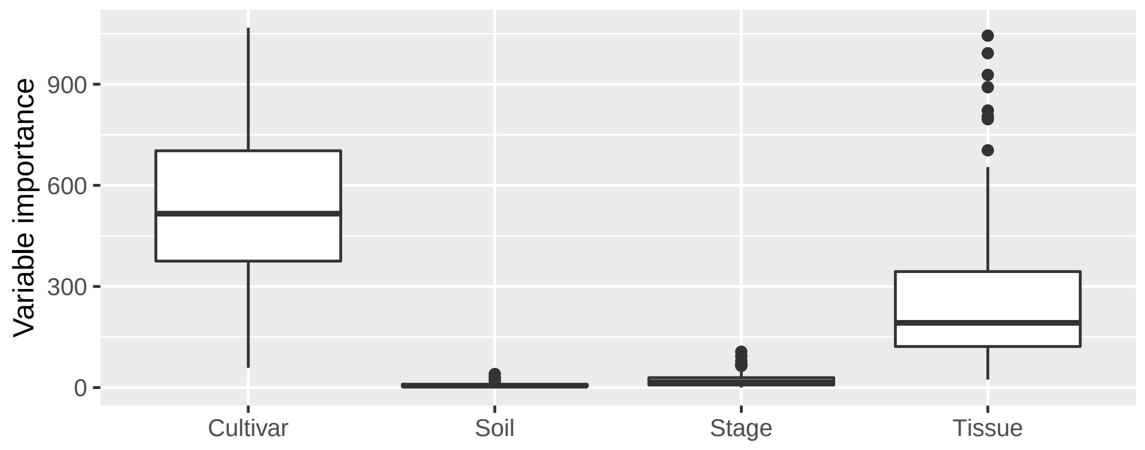
Variable importance
900
600
300
0
Cultivar
Soil
Stage
Tissue

Cluster no. 95

```
## Number of genes in the cluster: 50
## Homogeneity Index:    0.74
## Variable importance for Stage:     Rank =  95  - Median =  15.84
## Variable importance for Cultivar:  Rank =  68  - Median =  17.85
## Variable importance for Tissue:    Rank =   5  - Median =  952.2
## Variable importance for Soil:      Rank =  75  - Median =   4.44
##
## Gene ID                Gene Annotation
## VIT_13s0064g00470  -  Disease resistance protein (NBS-LRR class) RGH1
## VIT_01s0011g04250  -  Zinc finger (B-box type)
## VIT_19s0093g00150  -  Glutathione S-transferase 25 GSTU25
## VIT_06s0061g01070  -  Oligopeptidase A
## VIT_19s0085g00920  -  Organic cation transport protein OCT2
## VIT_05s0020g03360  -  Unknown
## VIT_01s0026g02590  -  Calcium-binding protein CML
## VIT_13s0156g00150  -  Unknown
## VIT_07s0005g02070  -  No hit
## VIT_08s0007g08070  -  No transmitting tract
## VIT_05s0049g00720  -  No hit
## VIT_08s0007g01900  -  Proton-dependent oligopeptide transport (POT) family protein
## VIT_12s0059g00290  -  Calmodulin-binding region IQD33
## VIT_19s0015g02820  -  Potassium transporter 1
## VIT_12s0057g00460  -  Auxin-responsive protein
## VIT_02s0025g04880  -  Geraniol 10-hydroxylase
## VIT_00s0207g00220  -  Cold acclimation protein
## VIT_04s0008g04390  -  No hit
## VIT_10s0003g02620  -  AWPM19
## VIT_15s0024g01540  -  Glutathione S-transferase 8 GSTU8
## VIT_10s0003g03190  -  RNA recognition motif (RRM)-containing
## VIT_17s0000g03200  -  Unknown protein
## VIT_18s0001g13530  -  SH3 domain-containing protein 2
## VIT_11s0103g00370  -  Cd2+-exporting ATPase HMA2 (Heavy metal ATPase 2)
## VIT_08s0007g07350  -  Pyridoxamine 5'-phosphate oxidase
## VIT_07s0005g05610  -  3-ketoacyl-CoA thiolase PED1
## VIT_17s0000g07260  -  Glucan endo-1,3-beta-glucosidase 3 precursor
## VIT_03s0038g02160  -  Thaumatin
## VIT_03s0091g01140  -  Meprin and TRAF homology domain-containing protein
## VIT_14s0006g01030  -  Calmodulin-binding heat-shock protein
## VIT_14s0006g00250  -  Cysteine-rich repeat secretory protein 60
## VIT_08s0007g01020  -  Aldo-keto reductase
## VIT_17s0000g06400  -  NAC domain-containing protein (VvNAC05)
## VIT_10s0071g00890  -  Unknown protein
## VIT_13s0067g00140  -  Proline-rich family protein
## VIT_00s0174g00170  -  Myb family
## VIT_01s0026g02100  -  Unknown protein
## VIT_17s0000g02480  -  Calcium-binding protein
## VIT_11s0103g00380  -  Cd2+-exporting ATPase HMA2 (Heavy metal ATPase 2)
## VIT_14s0030g01220  -  No hit
## VIT_12s0059g01920  -  Strubbelig-receptor family 3
## VIT_08s0040g01290  -  F-box family protein
## VIT_00s0214g00150  -  F-box protein PP2-B1 (Protein phloem protein 2-like B1)
## VIT_00s0268g00020  -  Phytase
## VIT_04s0008g04490  -  MARD1 (mediator of ABA-regulated dormancy 1)
## VIT_14s0066g02410  -  Aldehyde Dehydrogenase (VvALDH10B1)
```

```
## VIT_01s0026g02520  -  Ribonuclease T2
## VIT_01s0010g01000  -  NIK1 (NSP- interacting  kinase 1)
## VIT_01s0011g01660  -  VAMP726 (VESICLE-associated membrane protein)
## VIT_01s0011g06000  -  Vacuolar iron transporter 1
```

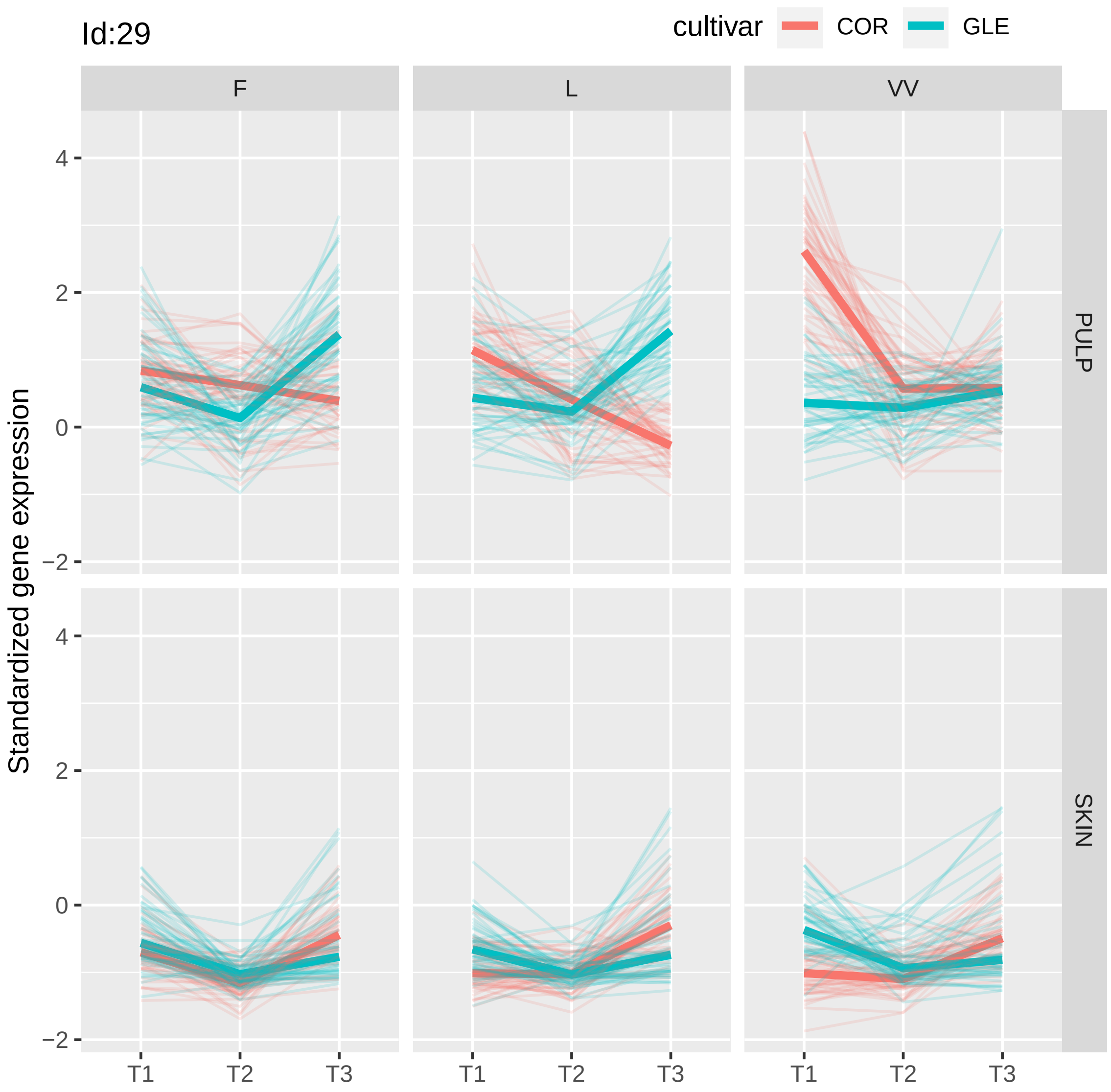
Id:29
cultivar
COR
GLE
F
L
VV
PULP
SKIN
Standardized gene expression
4
2
0
−2
T1
T2
T3

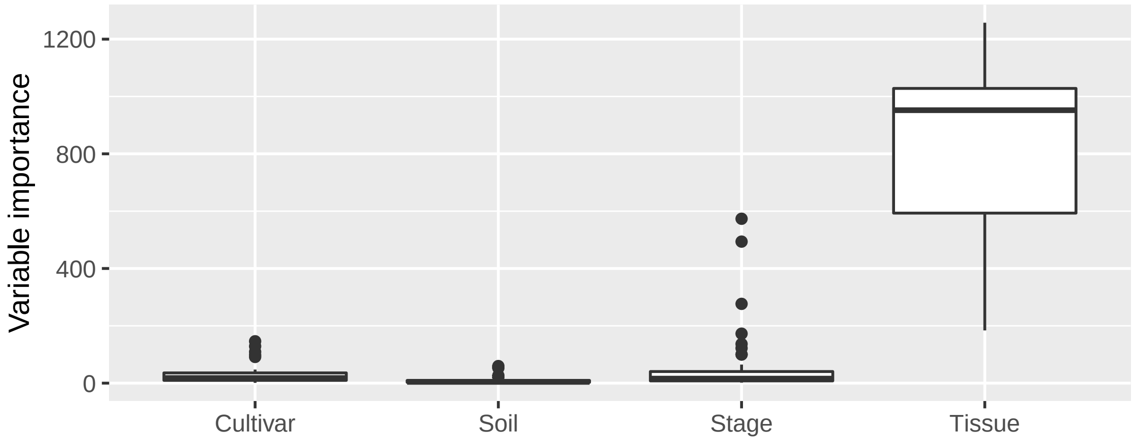
Variable importance
1200
800
400
0
Cultivar
Soil
Stage
Tissue

Cluster no. 96

```
## Number of genes in the cluster: 63
## Homogeneity Index:     0.8
## Variable importance for Stage:     Rank =  96  - Median =  13.37
## Variable importance for Cultivar:  Rank =   4  - Median =  848.6
## Variable importance for Tissue:    Rank =  56  - Median =  102.8
## Variable importance for Soil:      Rank = 100  - Median =   2.48
##
## Gene ID                Gene Annotation
## VIT_04s0008g01450  -  Cyclopropane-fatty-acyl-phospholipid synthase
## VIT_16s0039g02290  -  Unknown
## VIT_09s0002g03840  -  RPS5 (resistant to p. syringae 5)
## VIT_19s0014g00500  -  Receptor kinase 2
## VIT_18s0001g01250  -  Senescence-inducible chloroplast stay-green protein 2
## VIT_09s0002g02890  -  FRK1 (FLG22-induced receptor-like kinase 1)
## VIT_10s0003g01010  -  Lipase GDSL
## VIT_16s0148g00060  -  Receptor kinase homolog LRK10
## VIT_07s0104g00220  -  Phenazine biosynthesis PhzC/PhzF
## VIT_00s0187g00210  -  Rho GDP-dissociation inhibitor 2
## VIT_01s0011g04280  -  Receptor-like kinase ARK1AS
## VIT_06s0004g04810  -  Protein kinase APK1A
## VIT_18s0001g15560  -  Unknown protein
## VIT_15s0046g01300  -  No hit
## VIT_19s0015g01750  -  WD-40 repeat
## VIT_15s0046g03770  -  Anaphase-promoting complex component APC11
## VIT_00s0582g00010  -  Phosphoglucomutase/phosphomannomutase C terminal
## VIT_18s0001g05830  -  Unknown
## VIT_19s0090g01180  -  Light induced protein like
## VIT_00s0199g00110  -  Threonine endopeptidase
## VIT_03s0091g00010  -  No hit
## VIT_16s0013g01610  -  Ribosomal protein P1  acidic 60S
## VIT_09s0002g08500  -  No hit
## VIT_18s0122g01290  -  RWP-RK domain-containing protein
## VIT_03s0088g01060  -  Aminotransferase AGD2
## VIT_19s0093g00400  -  Glutathione S-transferase 25 GSTU25
## VIT_14s0036g00070  -  R protein  PRF  disease resistance protein
## VIT_15s0024g01420  -  DEAD (Asp-Glu-Ala-Asp) box polypeptide 18
## VIT_09s0002g02880  -  FRK1 (FLG22-induced receptor-like kinase 1)
## VIT_09s0018g00410  -  Cyclic nucleotide gated channel 5
## VIT_03s0017g00660  -  Nematode resistance-like protein
## VIT_08s0007g05180  -  Unknown
## VIT_05s0102g01090  -  Unknown
## VIT_04s0008g05160  -  Unknown protein
## VIT_04s0023g02350  -  CYP722A1
## VIT_12s0034g01750  -  R protein MLA10
## VIT_05s0102g01080  -  Disease resistance protein (CC-NBS-LRR class)
## VIT_01s0011g06450  -  Deoxymugineic acid synthase
## VIT_03s0017g00940  -  Cytochrome B6-F complex iron-sulfur subunit, PETC
## VIT_03s0063g00780  -  CXE carboxylesterase
## VIT_17s0000g07560  -  EDS1 (Enhanced disease susceptibility 1)
## VIT_03s0017g01740  -  Annexin ANN3
## VIT_06s0009g00980  -  CXE carboxylesterase
## VIT_10s0003g01130  -  Armadillo/beta-catenin repeat
## VIT_09s0002g07630  -  Beta-galactosidase
## VIT_18s0041g00190  -  R protein L6
```

```
## VIT_19s0085g00830  -  Ent-kaur-16-ene synthase (VvTPS69)
## VIT_09s0002g08020  -  R protein MLA10
## VIT_14s0066g00760  -  Disease resistance protein (NBS-LRR class)
## VIT_11s0118g00620  -  Methyltransferase type 11
## VIT_19s0027g01580  -  Disease resistance protein (NBS-LRR class)
## VIT_00s0207g00260  -  No hit
## VIT_06s0080g00980  -  Secoisolariciresinol dehydrogenase
## VIT_13s0073g00140  -  Ovate family protein 13 OFP13
## VIT_09s0002g08290  -  R protein MLA10
## VIT_15s0024g01020  -  Amidase
## VIT_14s0036g00100  -  Disease resistance protein (CC-NBS-LRR class)
## VIT_13s0019g02130  -  Tropinone reductase
## VIT_05s0020g02870  -  DEAD/DEAH box helicase
## VIT_03s0088g00340  -  No hit
## VIT_01s0026g00070  -  No hit
## VIT_04s0008g05550  -  SHR5-receptor-like kinase
## VIT_10s0071g01120  -  Alpha-galactosidase
```

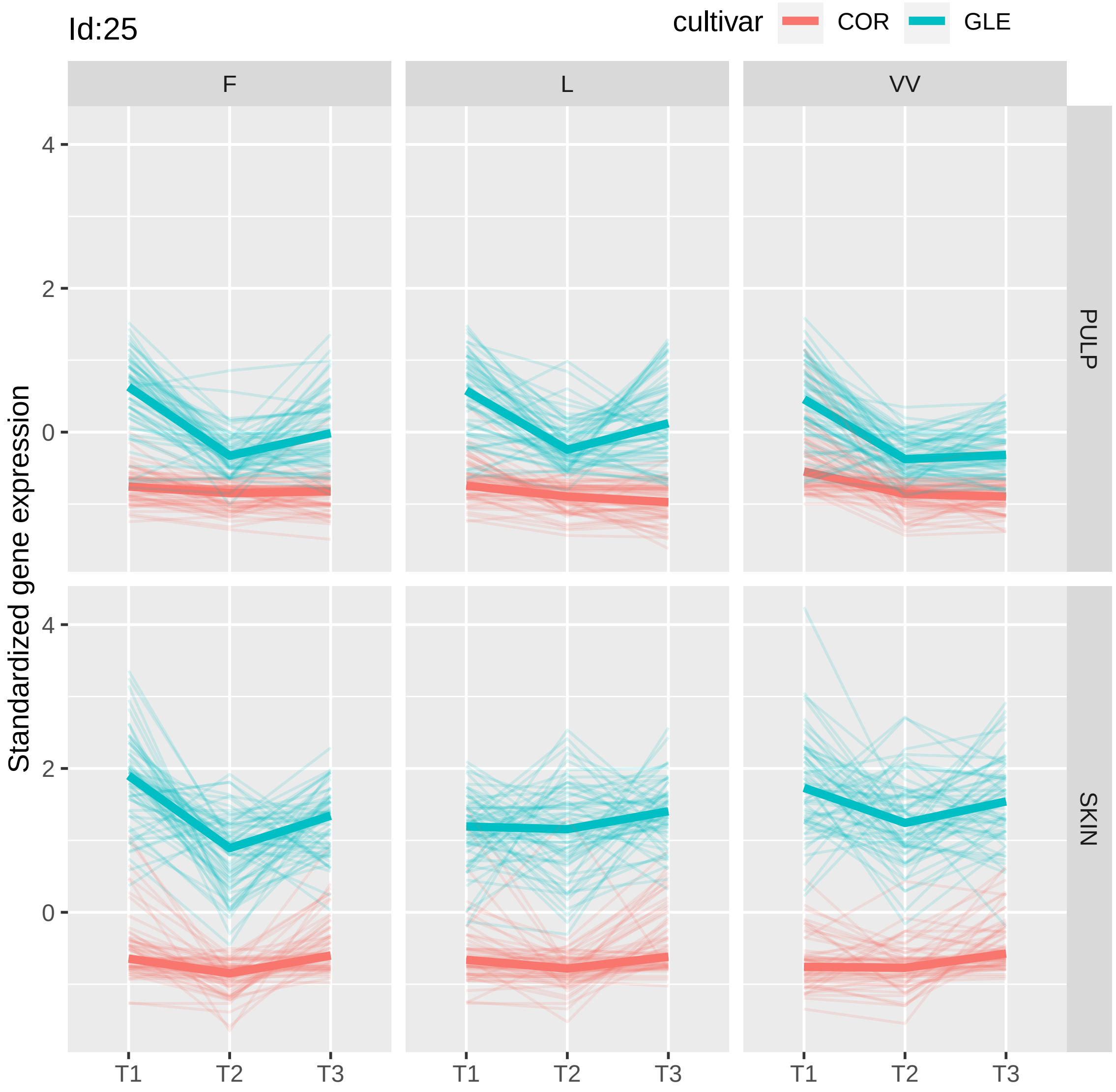
Id:25
cultivar
COR
GLE
F
L
VV
PULP
SKIN
Standardized gene expression
4
2
0
T1
T2
T3

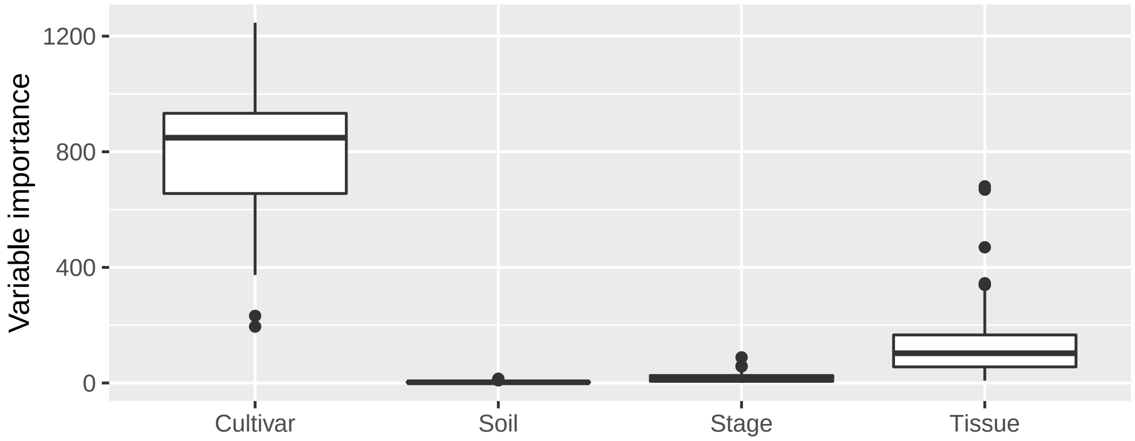
Variable importance
1200
800
400
0
Cultivar
Soil
Stage
Tissue

Cluster no. 97

```
## Number of genes in the cluster: 112
## Homogeneity Index:    0.85
## Variable importance for Stage:     Rank =  97  - Median =   12.1
## Variable importance for Cultivar:  Rank =  35  - Median =  89.54
## Variable importance for Tissue:    Rank =   6  - Median =  935.3
## Variable importance for Soil:      Rank =  96  - Median =   2.85
##
## Gene ID               Gene Annotation
## VIT_14s0081g00800  -  R protein  PRF  disease resistance protein
## VIT_12s0028g03570  -  SPA3 (SPA1-related 3)
## VIT_08s0007g04550  -  NCS1 nucleoside transporter family protein
## VIT_00s0220g00110  -  Acetolactate synthase small subunit
## VIT_07s0130g00340  -  Esterase/lipase/thioesterase
## VIT_04s0023g00410  -  Photosystem I subunit XI (PSAL)
## VIT_01s0011g00730  -  Alpha-1,4-glucan phosphorylase type H
## VIT_17s0000g01750  -  Auxin-independent growth promoter
## VIT_08s0105g00430  -  Omega-3 fatty acid desaturase, chloroplast precursor
## VIT_13s0064g00650  -  ATAB2
## VIT_07s0031g01550  -  No hit
## VIT_01s0010g03910  -  Squamosa promoter-binding protein (VvSBP3)
## VIT_06s0009g01360  -  R protein  PRF  disease resistance protein
## VIT_19s0014g04160  -  ARK3 (Arabidopsis Receptor Kinase 3)
## VIT_15s0046g01990  -  UDP-glucosyltransferase
## VIT_08s0058g01450  -  STN8 (state transition 8)
## VIT_08s0007g05010  -  Unknown protein
## VIT_07s0031g01560  -  Unknown protein
## VIT_17s0000g06280  -  Geranylgeranyl reductase
## VIT_11s0037g00840  -  Tetratricopeptide repeat (TPR)-containing
## VIT_08s0007g09000  -  Glycerol-3-phosphate dehydrogenase
## VIT_17s0000g09370  -  Elongation factor EF-Tu RABE1B
## VIT_00s0332g00060  -  ATP synthase CF1 beta subunit
## VIT_02s0025g01670  -  Unknown protein
## VIT_09s0002g06830  -  Curculin (mannose-binding) lectin
## VIT_08s0007g08540  -  Mg-chelatase subunit XANTHA-F
## VIT_13s0067g00730  -  Carboxylic ester hydrolase
## VIT_18s0001g13260  -  ATP-dependent protease La (LON) domain-containing protein
## VIT_00s0323g00080  -  Elongation factor G, chloroplast precursor
## VIT_06s0004g00260  -  Shoot1 protein
## VIT_07s0005g03090  -  Cyclin-dependent protein kinase CDT1A
## VIT_06s0080g01100  -  Unknown protein
## VIT_08s0040g00790  -  FK506-binding protein genes family (VvFKBPc)
## VIT_13s0019g00320  -  Photosystem II protein P PsbP
## VIT_04s0008g01300  -  Glycine dehydrogenase
## VIT_01s0011g00850  -  Unknown protein
## VIT_19s0027g00130  -  Translation initiation factor IF-2, chloroplast
## VIT_04s0008g03560  -  Lactoylglutathione lyase
## VIT_01s0011g00130  -  Squamosa promoter-binding protein (VvSBP1)
## VIT_16s0039g02540  -  Rubredoxin
## VIT_02s0012g01120  -  Glycine cleavage system H protein, mitochondrial
## VIT_00s0225g00130  -  Alanine transaminase.
## VIT_18s0041g01220  -  GCN5 N-acetyltransferase (GNAT)
## VIT_18s0001g01210  -  Senescence-inducible chloroplast stay-green protein 2
## VIT_18s0001g14910  -  Mannitol dehydrogenase
## VIT_14s0066g00750  -  Disease resistance protein (NBS-LRR class)
```

```
## VIT_16s0050g01710  -  Receptor serine/threonine kinase PR5K
## VIT_16s0039g02530  -  No hit
## VIT_18s0001g14660  -  ABC Transporter (VvMDR3 - VvABCB3)
## VIT_04s0023g03010  -  fructose-bisphosphate aldolase, chloroplast precursor
## VIT_18s0122g00830  -  PMI1 (plastid movement impaired1)
## VIT_09s0018g00290  -  Unknown protein
## VIT_13s0019g04080  -  Protein transport protein Sec61 subunit beta
## VIT_01s0146g00400  -  Humj1
## VIT_11s0016g01220  -  Auxin-binding protein ABP19a precursor
## VIT_03s0038g03250  -  Vestitone reductase
## VIT_00s0211g00160  -  Serine hydroxymethyltransferase 1
## VIT_16s0050g00710  -  Unknown protein
## VIT_18s0001g08800  -  Lecithine cholesterol acyltransferase-like protein
## VIT_13s0019g04250  -  Unknown protein
## VIT_11s0037g00850  -  Tetratricopeptide repeat (TPR)-containing
## VIT_04s0043g01010  -  violaxanthin de-epoxidase (VDE1) (VvVDE1)
## VIT_19s0015g02910  -  CYP72A1
## VIT_16s0022g00470  -  Peroxisomal biogenesis factor 11 (PEX11)
## VIT_00s0211g00120  -  Glycine hydroxymethyltransferase
## VIT_00s0371g00050  -  Mannitol dehydrogenase
## VIT_01s0011g02800  -  RIC7 (ROP-interactive crib motif-containing protein 7)
## VIT_15s0046g00480  -  Wax synthase
## VIT_13s0084g00070  -  CYP72A53v1
## VIT_16s0050g01720  -  Receptor serine/threonine kinase PR5K
## VIT_01s0127g00740  -  Quinone oxidoreductase, chloroplast precursor
## VIT_01s0010g01870  -  Salicylic acid-induced fragment 1 protein
## VIT_02s0025g01960  -  No hit
## VIT_02s0025g02640  -  Unknown protein
## VIT_12s0034g00130  -  Anthocyanidin 3-O-glucosyltransferase
## VIT_00s0475g00020  -  Zinc finger (C3HC4-type ring finger)
## VIT_00s0346g00110  -  Mannitol dehydrogenase
## VIT_06s0004g05310  -  Tropinone reductase
## VIT_02s0241g00040  -  Cyclobutane pyrimidine dimer photolyase
## VIT_09s0002g01450  -  Unknown protein
## VIT_00s0207g00010  -  Anthranilate N-benzoyltransferase protein 1
## VIT_07s0129g00530  -  Hydrolase, alpha/beta fold family
## VIT_13s0067g03260  -  Pheophorbidase
## VIT_11s0037g01400  -  Zinc finger (C3HC4-type ring finger)
## VIT_00s0211g00080  -  Serine hydroxymethyltransferase 2
## VIT_00s1206g00010  -  Aspartic proteinase nepenthesin-1 precursor
## VIT_02s0154g00040  -  Thylakoid lumenal protein
## VIT_08s0007g00530  -  3-beta hydroxysteroid dehydrogenase/isomerase protein
## VIT_14s0066g00400  -  Chalcone isomerase
## VIT_03s0038g01330  -  Anthranilate N-benzoyltransferase protein 1
## VIT_05s0020g04560  -  Armadillo/beta-catenin repeat protein / U-box domain-containing protei
## VIT_07s0129g00990  -  Protein kinase
## VIT_06s0004g07770  -  Peroxidase
## VIT_16s0100g00200  -  Unknown protein
## VIT_03s0063g01560  -  CYP82C1p
## VIT_18s0001g15330  -  Nodulin MtN3 family
## VIT_09s0054g01410  -  Beta-amyrin synthase
## VIT_00s0259g00120  -  Oligopeptide transporter OPT3
## VIT_04s0008g06300  -  Leucine-rich repeat family
## VIT_14s0060g00480  -  S-adenosylmethionine synthetase 1 (SAM1)
```

```
## VIT_00s0262g00010  -  Receptor kinase RK20-1
## VIT_19s0177g00030  -  Gibberellin 2-beta-dioxygenase 7
## VIT_01s0011g02860  -  CHUP1 (chloroplast unusual positioning 1)
## VIT_18s0001g00690  -  Glutathione S-transferase 8 GSTF8
## VIT_00s2377g00010  -  Unknown protein
## VIT_10s0116g01880  -  MATE efflux family protein
## VIT_00s0429g00040  -  Aspartic Protease (VvAP47)
## VIT_11s0016g02350  -  Ubiquinone/menaquinone biosynthesis methyltransferase UbiE
## VIT_11s0037g00690  -  CF5
## VIT_05s0062g01120  -  PIN1
## VIT_06s0009g02640  -  Hydroxymethylglutaryl-CoA lyase
## VIT_07s0205g00030  -  Zinc finger (C3HC4-type ring finger)
```

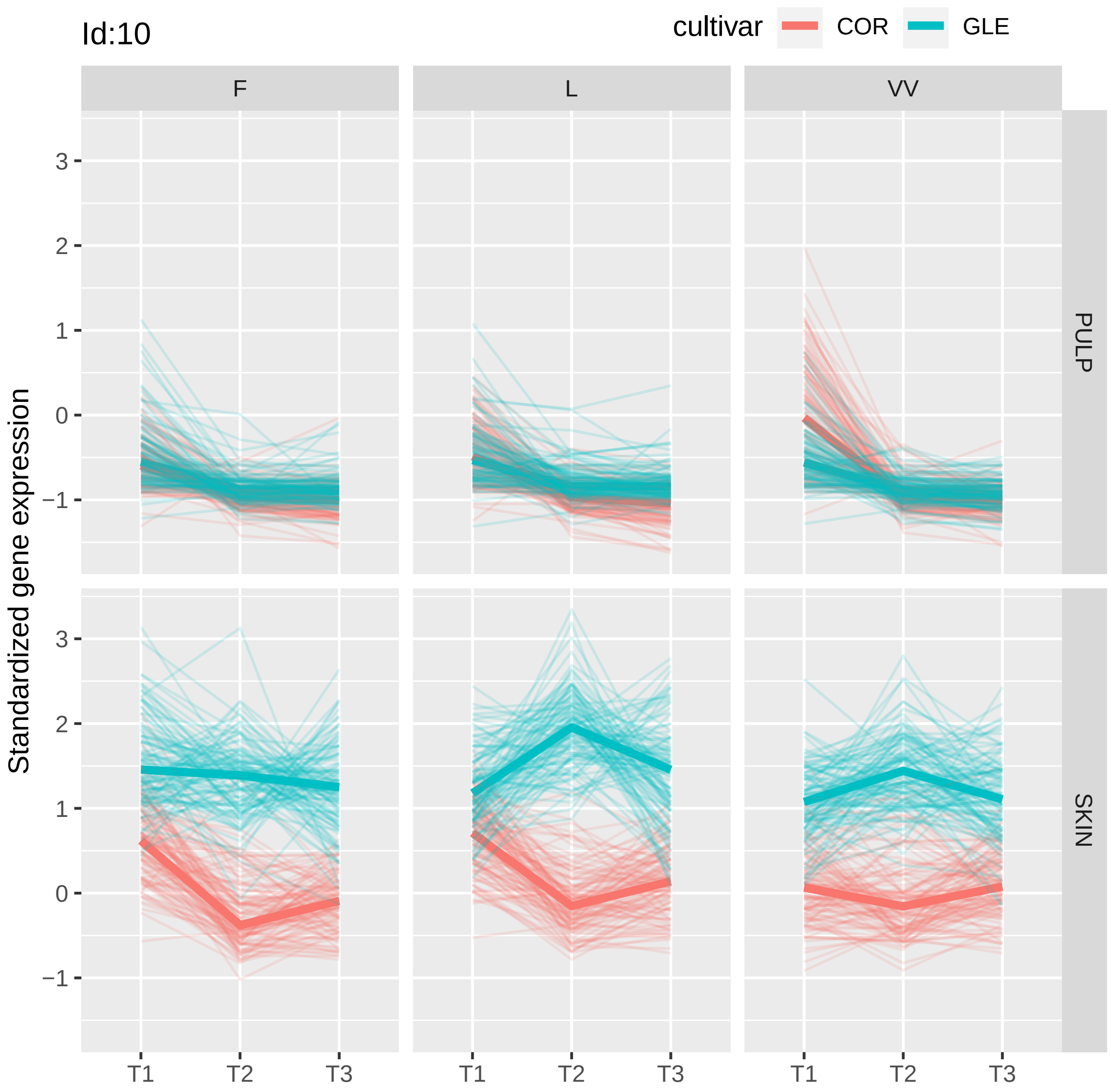

Id:10
cultivar
COR
GLE
F
L
VV
PULP
SKIN
Standardized gene expression
3
2
1
0
−1
T1
T2
T3

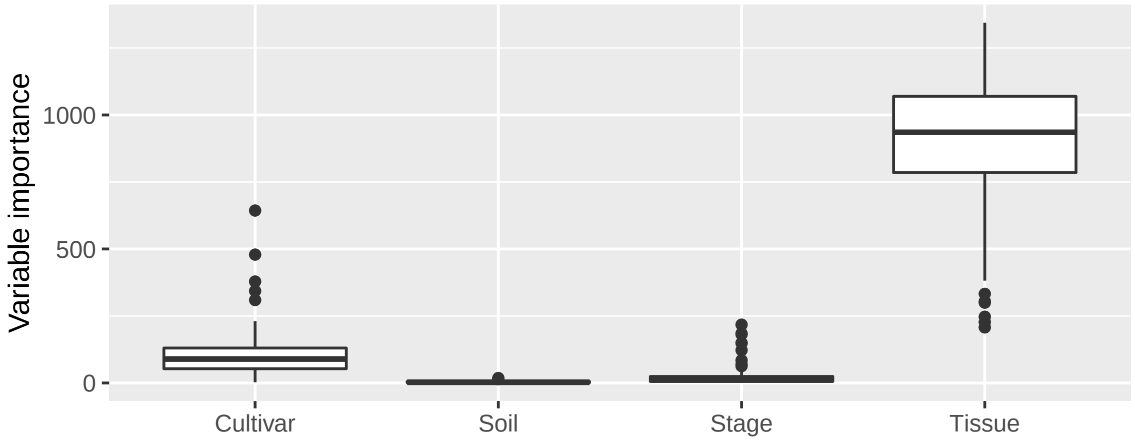

Variable importance
1000
500
0
Cultivar
Soil
Stage
Tissue

Cluster no. 98

```
## Number of genes in the cluster: 44
## Homogeneity Index:    0.85
## Variable importance for Stage:     Rank =  98  - Median =  10.27
## Variable importance for Cultivar:  Rank =  21  - Median =  191.6
## Variable importance for Tissue:    Rank =  30  - Median =  381.8
## Variable importance for Soil:      Rank =  62  - Median =   5.12
##
## Gene ID               Gene Annotation
## VIT_00s0271g00110  -  flavodoxin-like quinone reductase 1
## VIT_06s0061g00590  -  Nodulin family protein
## VIT_12s0059g00750  -  Prephenate dehydratase
## VIT_11s0016g04160  -  Sulfate transporter 3.5
## VIT_13s0064g00460  -  Unknown protein
## VIT_09s0018g01980  -  Unknown
## VIT_14s0066g01500  -  UDP-glucoronosyl and UDP-glucosyl transferase
## VIT_09s0070g00710  -  Isopentenyltransferase
## VIT_14s0066g01490  -  UDP-glucoronosyl and UDP-glucosyl transferase
## VIT_11s0016g02110  -  FAD linked oxidase, N-terminal
## VIT_13s0106g00250  -  Carboxylic ester hydrolase
## VIT_01s0010g02440  -  ABC Transporter (VvMRP1 - VvABCC1)
## VIT_19s0140g00210  -  SOUL heme-binding
## VIT_16s0098g00950  -  Subtilase
## VIT_16s0098g01160  -  Subtilisin protease
## VIT_12s0134g00500  -  Cyclin D6
## VIT_18s0001g00300  -  Auxin responsive SAUR protein
## VIT_01s0011g03450  -  Alpha-glucosidase
## VIT_04s0023g02010  -  Hydrolase, alpha/beta fold
## VIT_02s0025g03710  -  DTA2 (downstream target of AGL15 2)
## VIT_00s1286g00020  -  Elongation factor EF-G
## VIT_17s0000g09800  -  No hit
## VIT_17s0000g09810  -  Pectate lyase
## VIT_13s0019g02120  -  Zinc finger (C2H2 type) family
## VIT_05s0077g01670  -  s3_Pathogenesis protein 10 [Vitis vinifera]
## VIT_06s0004g07970  -  Expansin  (VvEXPA7)
## VIT_16s0050g02520  -  RNA polymerase sigma subunit SigE (sigE)
## VIT_10s0003g02120  -  Lipase GDSL
## VIT_04s0008g04460  -  Molybdate transporter 1
## VIT_06s0004g05330  -  Tropinone reductase
## VIT_05s0049g01100  -  Glutathione S-transferase 25 GSTU7
## VIT_18s0001g14800  -  Lipase 3 (EXL3) family II extracellular
## VIT_06s0004g04000  -  Beta-ketoacyl-CoA synthase
## VIT_11s0118g00770  -  Unknown protein
## VIT_13s0047g00030  -  ZIFL1 (Zinc induced facilitator 1)
## VIT_14s0108g01020  -  Expansin  (VvEXPA16)
## VIT_15s0046g01980  -  UDP-glucosyltransferase
## VIT_06s0004g01750  -  Polygalacturonase inhibitor protein
## VIT_01s0011g06580  -  Unknown protein
## VIT_02s0109g00310  -  flavonoid 3-monooxygenase
## VIT_05s0049g01070  -  Glutathione S-transferase 19 GSTU1
## VIT_13s0047g00040  -  ZIFL1 (Zinc induced facilitator 1)
## VIT_10s0116g00410  -  Gibberellin 2-beta-dioxygenase 7
## VIT_19s0090g00710  -  No hit
```

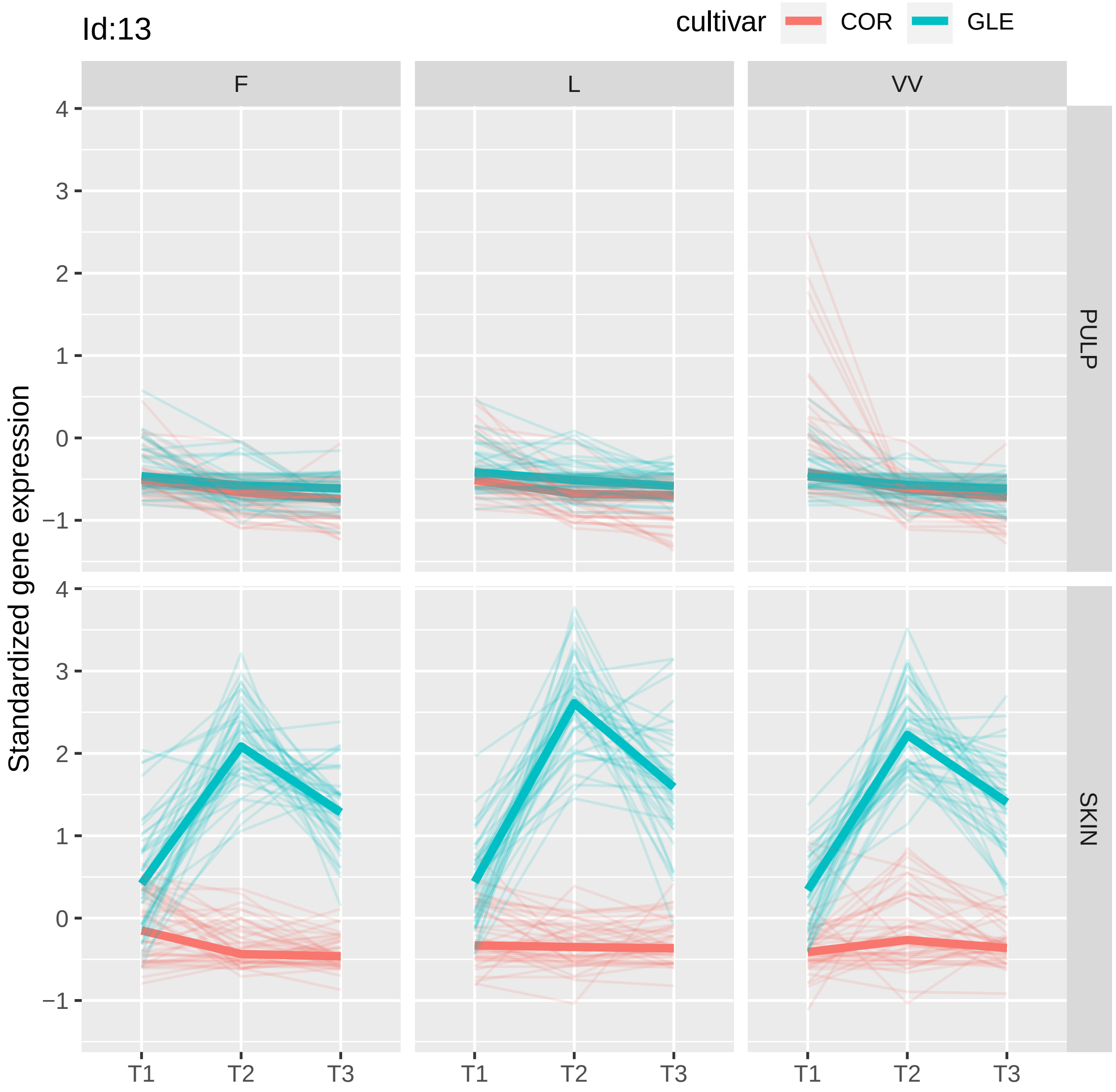
Id:13
cultivar
COR
GLE
F
L
VV
PULP
SKIN
Standardized gene expression
4
3
2
1
0
−1
T1
T2
T3

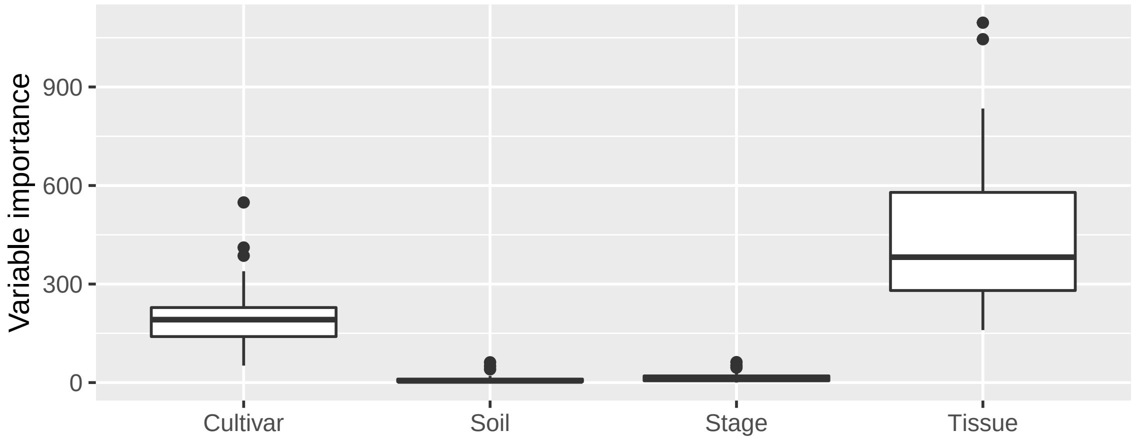
Variable importance
900
600
300
0
Cultivar
Soil
Stage
Tissue

Cluster no. 99

```
## Number of genes in the cluster: 31
## Homogeneity Index:    0.83
## Variable importance for Stage:     Rank =  99  - Median =  10.06
## Variable importance for Cultivar:  Rank =   2  - Median =   1059
## Variable importance for Tissue:    Rank =  97  - Median =  10.96
## Variable importance for Soil:      Rank =  93  - Median =   3.31
##
## Gene ID               Gene Annotation
## VIT_08s0007g06340  -  Unknown protein
## VIT_00s0372g00010  -  Zinc-binding protein
## VIT_13s0019g02180  -  Tropinone reductase
## VIT_19s0014g04060  -  ARK3 (Arabidopsis Receptor Kinase 3)
## VIT_06s0080g00630  -  No hit
## VIT_08s0007g03750  -  1,4-alpha-glucan branching enzyme IIB, chloroplast precursor
## VIT_18s0001g06700  -  No hit
## VIT_19s0014g03380  -  3'(2') 5'-bisphosphate nucleotidase
## VIT_09s0096g00390  -  Disease resistance protein (NBS-LRR class)
## VIT_13s0019g03990  -  Unknown protein
## VIT_11s0037g00440  -  Hydroxycinnamoyl-CoA shikimate/quinate hydroxycinnamoyltransferase
## VIT_01s0010g01130  -  6-phosphogluconate dehydrogenase
## VIT_03s0017g02290  -  Regulator of chromosome condensation (RCC1)
## VIT_17s0000g04410  -  TGH (tough); RNA binding
## VIT_09s0002g02730  -  FRK1 (FLG22-induced receptor-like kinase 1)
## VIT_09s0054g00360  -  Cation efflux family protein MTPc4
## VIT_00s0199g00130  -  Cysteine desulfurase
## VIT_11s0149g00300  -  Chitinase, class V
## VIT_03s0063g01250  -  Nodulin 1A, Senescence-associated
## VIT_09s0002g06260  -  RPS5 (resistant to p. syringae 5)
## VIT_02s0154g00130  -  Exostosin (Xyloglucan galactosyltransferase KATAMARI 1)
## VIT_14s0068g00490  -  UDP-glucuronosyl/UDP-glucosyltransferase
## VIT_12s0034g01710  -  No hit
## VIT_04s0008g04610  -  PDE247 (pigment defective 247)
## VIT_19s0015g02760  -  Ring-H2 finger protein ATL1B
## VIT_16s0039g02300  -  EIX receptor 2
## VIT_18s0001g09290  -  Unknown protein
## VIT_07s0031g03220  -  Patellin-1
## VIT_14s0036g00810  -  HMG-CoA synthase 2
## VIT_17s0000g02800  -  Hydroxymethylglutaryl coenzyme A synthase
## VIT_07s0005g01340  -  Ribosomal protein L29e
```

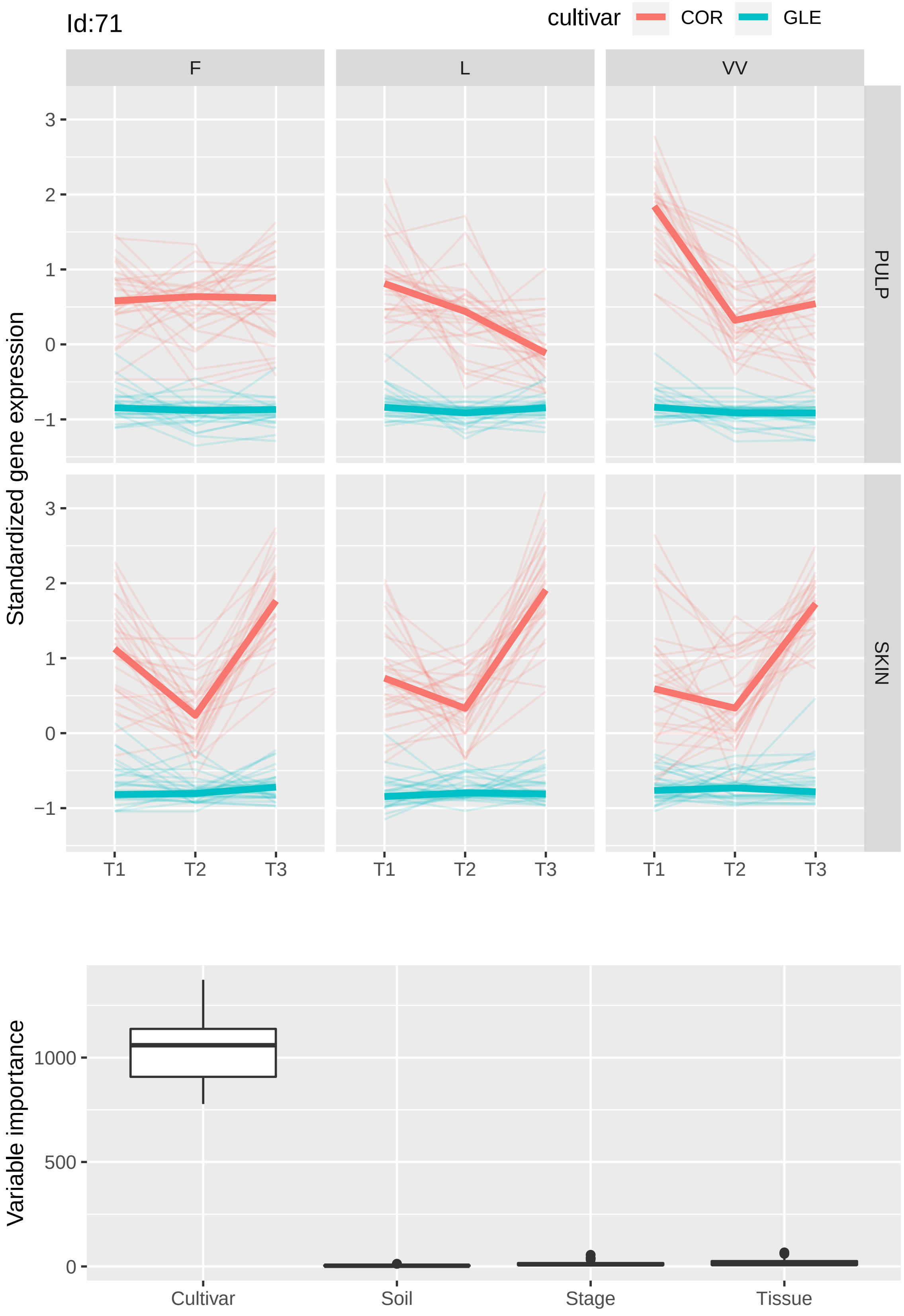

Id:71
cultivar
COR
GLE
F
L
VV
PULP
SKIN
Standardized gene expression
3
2
1
0
−1
T1
T2
T3
Variable importance
1000
500
0
Cultivar
Soil
Stage
Tissue

Cluster no. 100

```
## Number of genes in the cluster: 106
## Homogeneity Index:     0.8
## Variable importance for Stage:     Rank = 100  - Median =   9.67
## Variable importance for Cultivar:  Rank =  67  - Median =  18.81
## Variable importance for Tissue:    Rank =   2  - Median =  999.2
## Variable importance for Soil:      Rank =  99  - Median =   2.53
##
## Gene ID                Gene Annotation
## VIT_08s0007g00240  -  WD-40 repeat protein
## VIT_02s0025g04590  -  TCP family transcription factor TCP9
## VIT_16s0100g00670  -  Homeodomain GLABROUS1
## VIT_00s0125g00180  -  Zinc finger (C3HC4-type ring finger)
## VIT_00s0409g00050  -  Receptor-like serine-threonine protein kinase
## VIT_18s0001g08040  -  basic helix-loop-helix (bHLH) family
## ENSRNA049996612  -
## VIT_03s0038g01180  -  Auxin responsive SAUR protein
## VIT_11s0016g05740  -  Calmodulin-5 (CAM5)
## VIT_16s0050g02430  -  Receptor protein kinase
## VIT_07s0005g02570  -  WRKY Transcription Factor (VvWRKY20)
## VIT_00s0225g00090  -  Copper amine oxidase
## VIT_03s0038g01760  -  Disease resistance protein (CC-NBS class)
## VIT_10s0003g00580  -  ERF/AP2 Gene Family (VvERF075)
## VIT_18s0001g08770  -  CF9
## VIT_12s0055g01280  -  Brassinosteroid insensitive 1-associated receptor kinase 1
## VIT_05s0102g00710  -  fructokinase-1
## VIT_17s0000g00330  -  Inducer of CBF expression 1 ICE1
## VIT_16s0098g00010  -  Receptor-like protein kinase
## VIT_14s0030g00650  -  Shikimate dehydrogenase
## VIT_12s0035g01260  -  NBS-LRR type disease resistance protein
## VIT_10s0071g01200  -  Vacuolar pyrophosphatase [Vitis vinifera]
## VIT_13s0019g01710  -  Scarecrow transcription factor 14 (SCL14)
## VIT_14s0108g00270  -  Aldose 1-epimerase
## VIT_12s0028g00610  -  Aspartic Protease (VvAP31)
## VIT_12s0055g00020  -  UDP-glucose glucosyltransferase
## VIT_07s0005g04380  -  IAA12
## VIT_16s0050g00930  -  anthocyanin permease (VvAnthoMATE1 - VvAM1)
## VIT_12s0059g02280  -  Spg1p (septum-promoting GTPase)
## VIT_18s0001g05030  -  Glucan 1,3-beta-glucosidase precursor
## VIT_17s0000g02100  -  Wall-associated kinase 2 (WAK2)
## VIT_18s0041g00710  -  UDP-glucose: anthocyanidin 5,3-O-glucosyltransferase
## VIT_16s0148g00040  -  Receptor serine/threonine kinase
## VIT_09s0002g02700  -  basic helix-loop-helix (bHLH) family
## VIT_15s0048g02230  -  Calcineurin phosphoesterase
## VIT_03s0017g01940  -  Unknown
## VIT_00s0424g00020  -  Receptor serine/threonine kinase
## VIT_01s0011g00380  -  SP1L1 (SPIRAL1-like1)
## VIT_14s0128g00160  -  Protein kinase CDG1
## VIT_02s0241g00060  -  Unknown protein
## VIT_15s0046g02060  -  ATRAD3 (Arabidopsis thaliana Ras Associated with Diabetes protein 3
## VIT_09s0002g03570  -  ABC transporter G member 15
## VIT_18s0001g07700  -  Ethylene-inducible CTR1
## VIT_07s0141g00920  -  CYP94A1
## VIT_00s0225g00050  -  MATE efflux family protein
## VIT_12s0034g00060  -  UDP-glucose glucosyltransferase
```

```
## VIT_15s0048g02240  -  Acid phosphatase PAP14
## VIT_08s0007g03970  -  Peroxiredoxin type 2
## VIT_11s0016g01480  -  Nuclear transcription factor Y subunit A-3
## VIT_13s0074g00700  -  ABC Transporter (VvPDR27 - VvABCG57)
## VIT_10s0116g01830  -  Unknown protein
## VIT_13s0139g00100  -  Disease resistance protein (CC-NBS-LRR class)
## VIT_08s0105g00420  -  Unknown
## VIT_01s0026g01550  -  Homeodomain leucine zipper protein HB-1
## VIT_19s0014g05320  -  Leucine-rich repeat transmembrane protein kinase, putative
## VIT_16s0148g00030  -  Receptor-like protein kinase LRK14
## VIT_06s0004g05240  -  Ethylene receptor (ETR2)
## VIT_00s0707g00010  -  Heat shock protein (HSP26.5-P) 26.5 kDa class P
## VIT_14s0060g01940  -  Epoxide hydrolase
## VIT_15s0048g02000  -  Homeodomain GLABROUS1
## VIT_07s0031g02610  -  NAC domain-containing protein (VvNAC39)
## VIT_07s0005g04020  -  Leucine Rich Repeat receptor-like kinase
## VIT_02s0025g00140  -  Ring-H2 zinc finger protein RHA1b
## VIT_06s0004g02640  -  Receptor protein kinase PERK1
## VIT_12s0034g01140  -  Plastocyanin domain-containing protein
## VIT_06s0004g08250  -  Leucine-rich repeat transmembrane protein kinase, putative
## VIT_12s0059g02310  -  PDF2 (protodermal factor2)
## VIT_04s0008g03160  -  G protein protein gamma subunit (AGG2)
## VIT_15s0046g01810  -  S-locus lectin protein kinase
## VIT_14s0006g02510  -  Plastid movement impaired 15 (PMI15)
## VIT_00s0477g00030  -  MATE efflux family protein ripening responsive
## VIT_16s0013g01950  -  Hexose transporter [Vitis vinifera]
## VIT_18s0001g05580  -  Unknown protein
## VIT_06s0080g00070  -  S-receptor kinase
## VIT_09s0054g01230  -  Beta-amyrin synthase
## VIT_17s0000g04450  -  Phospholipid-transporting ATPase
## VIT_00s0301g00040  -  Glycine-rich protein
## VIT_13s0156g00550  -  S-receptor kinase
## VIT_00s0366g00020  -  CRK10 (cysteine-rich RLK10)
## VIT_12s0057g01410  -  Exostosin
## VIT_00s0259g00140  -  Oligopeptide transporter OPT3
## VIT_10s0003g00900  -  Anthranilate N-hydroxycinnamoyl/benzoyltransferase
## VIT_14s0060g00900  -  Unknown protein
## VIT_10s0003g04090  -  Remorin
## VIT_00s0388g00050  -  Receptor kinase homolog LRK14
## VIT_08s0058g01270  -  Zinc finger (C3HC4-type ring finger)
## VIT_19s0090g00190  -  Taxane 10-beta-hydroxylase
## VIT_18s0122g01160  -  12-oxophytodienoate reductase 2
## VIT_03s0017g01280  -  ABC Transporter (VvWBC12 - VvABCG12)
## VIT_00s0388g00060  -  Receptor serine/threonine kinase
## VIT_04s0008g03840  -  Ankyrin repeat
## VIT_04s0008g05500  -  S-receptor kinase
## VIT_19s0014g03390  -  Unknown protein
## VIT_08s0058g01230  -  Non-specific lipid-transfer protein
## VIT_18s0001g10810  -  Carboxylesterase CXE
## VIT_00s0388g00020  -  Receptor serine/threonine kinase PR5K
## VIT_05s0049g00940  -  Inorganic phosphate transporter 1-4
## VIT_08s0058g00590  -  Macrophage migration inhibitory factor
## VIT_03s0038g04730  -  Polyphenol oxidase
## VIT_08s0105g00300  -  Phospholipase C
## VIT_16s0050g00020  -  Cullin-4
```

```
## VIT_08s0032g01190  -  Nuclear transcription factor Y subunit A-10
## VIT_04s0023g02100  -  Unknown
## VIT_05s0029g01420  -  Ankyrin repeat
## VIT_02s0025g00420  -  WRKY Transcription Factor (VvWRKY04)
## VIT_15s0048g02720  -  fatty acid elongase
```

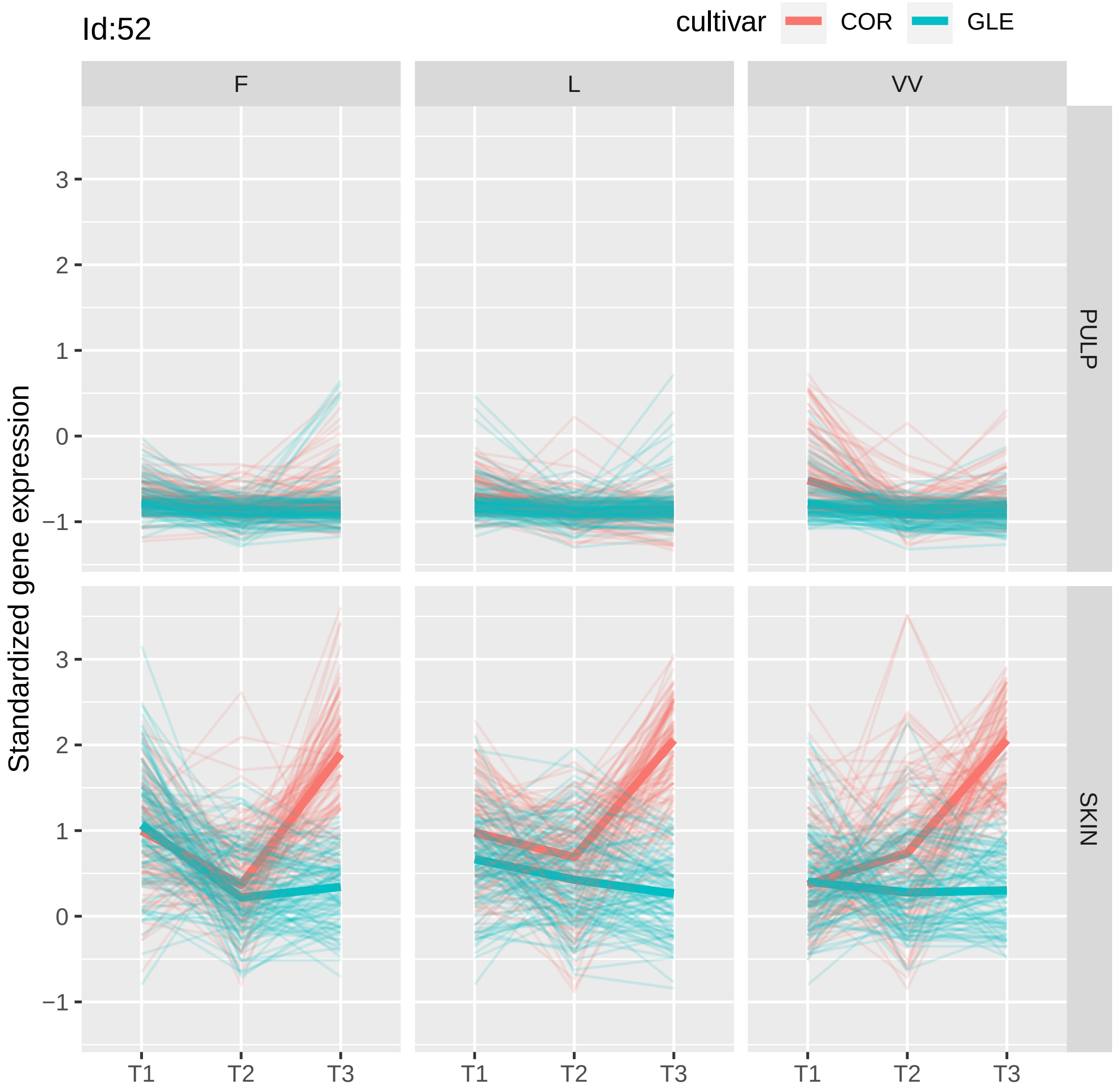

Id:52
cultivar
COR
GLE
F
L
VV
PULP
SKIN
Standardized gene expression
3
2
1
0
−1
T1
T2
T3

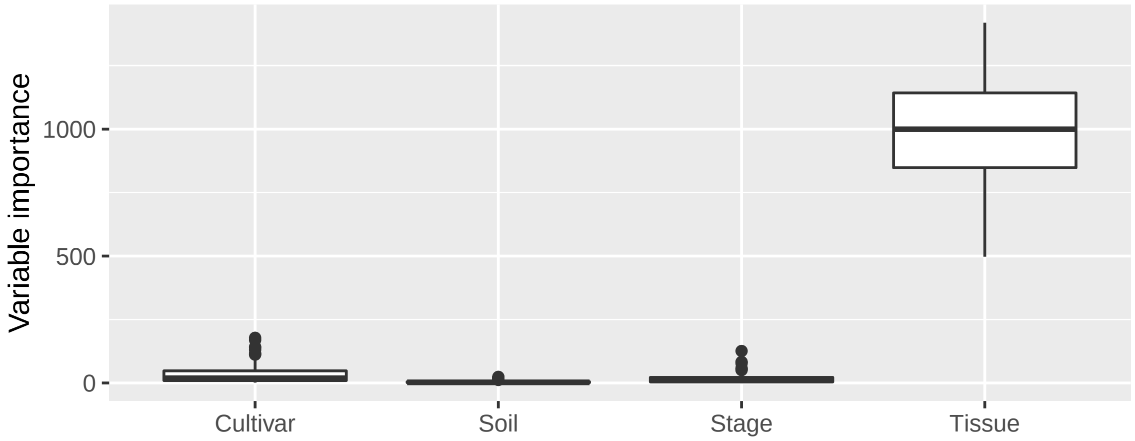

Variable importance
1000
500
0
Cultivar
Soil
Stage
Tissue

Cluster no. 101

```
## Number of genes in the cluster: 101
## Homogeneity Index:    0.85
## Variable importance for Stage:     Rank = 101  - Median =   6.55
## Variable importance for Cultivar:  Rank =   3  - Median =  933.5
## Variable importance for Tissue:    Rank =  68  - Median =   39.3
## Variable importance for Soil:      Rank =  95  - Median =   3.09
##
## Gene ID                Gene Annotation
## VIT_13s0067g00560  -  Cinnamyl alcohol dehydrogenase
## VIT_00s1684g00010  -  No hit
## VIT_13s0019g00010  -  Sulfate transporter 4.2
## VIT_02s0025g03620  -  Glutathione S-transferase 1 GSTT1
## VIT_00s0612g00010  -  Phospholipase/carboxylesterase
## VIT_09s0002g03290  -  Peroxiredoxin (alkyl hydroperoxide reductase subunit C)
## VIT_02s0087g00590  -  Ribosomal protein L30
## VIT_13s0064g00680  -  Retroelement pol polyprotein
## VIT_00s1613g00010  -  Syntaxin 52
## VIT_00s0642g00010  -  E3 ubiquitin-protein ligase CHIP
## VIT_10s0003g00910  -  Unknown protein
## VIT_10s0042g00210  -  Homeobox protein 23
## VIT_04s0023g02430  -  Basic Leucine Zipper Transcription Factor (VvbZIP13)
## VIT_09s0002g07920  -  Vacuolar proton-inorganic pyrophosphatase
## VIT_18s0041g02380  -  Disease resistance protein (TIR-NBS-LRR class)
## VIT_01s0011g03810  -  Receptor protein kinase
## VIT_00s0407g00040  -  Thymidylate synthase
## VIT_11s0016g03630  -  Peroxiredoxin-5
## VIT_07s0129g00100  -  Voltage dependent anion channel 2
## VIT_00s0524g00010  -  Armadillo/beta-catenin repeat protein / U-box domain-containing protei
## VIT_19s0014g05380  -  Monodehydroascorbate reductase
## VIT_09s0002g01130  -  Acylamino-acid-releasing enzyme
## VIT_02s0025g03910  -  VIP3 (vernalization independence 3); nucleotide binding
## VIT_19s0014g05370  -  No hit
## VIT_11s0016g02600  -  caffeoyl-CoA O-methyltransferase
## VIT_09s0096g00040  -  Ribonuclease P/MRP protein subunit POP1
## VIT_05s0062g00670  -  No hit
## VIT_15s0021g02850  -  Unknown
## VIT_04s0008g02220  -  Sterile alpha motif (SAM) domain-containing protein
## VIT_03s0088g00210  -  STT3B (staurosporin and temperature sensitive 3-like B)
## VIT_18s0076g00100  -  Pentatricopeptide repeat-containing protein
## VIT_00s0173g00250  -  RKF1 (receptor-like kinase in flowers 1)
## VIT_16s0022g00500  -  ABC transporter C member 12
## VIT_19s0093g00190  -  Glutathione S-transferase 25 GSTU25
## VIT_03s0017g01480  -  Integral membrane Yip1 family protein
## VIT_15s0107g00360  -  Unknown protein
## VIT_09s0070g00970  -  RKF1 (receptor-like kinase in flowers 1)
## VIT_14s0083g00420  -  TRN2 (TORNADO 2)
## VIT_00s0483g00030  -  Isochorismatase hydrolase
## VIT_01s0127g00520  -  Protein disulfide isomerase
## VIT_18s0041g02310  -  No hit
## VIT_07s0031g02700  -  Amidase
## VIT_09s0002g04930  -  Kelch repeat-containing F-box family protein
## VIT_11s0149g00070  -  Ribosomal protein S6
## VIT_06s0061g01500  -  No hit
## VIT_00s0174g00230  -  Zinc finger (FYVE type)
```

```
## VIT_00s0921g00020  -  Alpha-1,3-mannosyl-glycoprotein beta-1,2-N-acetylglucosaminyltransfera
## VIT_05s0020g00130  -  Unknown protein
## VIT_19s0014g05400  -  C-myc binding protein
## VIT_00s0203g00210  -  Zinc finger (B-box type)
## VIT_02s0012g02920  -  Acyl-CoA oxidase ACX3
## VIT_16s0050g01300  -  C2 domain-containing protein
## VIT_17s0000g06050  -  Ankyrin protein kinase
## VIT_14s0066g00770  -  No hit
## VIT_00s0352g00020  -  Glutamate carboxypeptidase (AMP1)
## VIT_19s0014g05360  -  Replication protein A 70 kDa DNA-binding subunit
## VIT_01s0026g01180  -  No hit
## VIT_18s0001g04950  -  No hit
## VIT_03s0017g00650  -  GTP cyclohydrolase II
## VIT_01s0127g00300  -  CRS2-associated factor 2, chloroplastic
## VIT_04s0008g04910  -  WD-40 repeat protein (LEUNIG)
## VIT_00s0187g00330  -  IQ calmodulin-binding region; Apoptosis regulator Bcl-2 protein, BAG 
## VIT_19s0014g05330  -  Ankyrin repeat
## VIT_00s0471g00040  -  BSD domain-containing protein
## VIT_18s0041g00340  -  CF4
## VIT_02s0025g03850  -  YDA (YODA)
## VIT_00s0921g00030  -  SRD2 (Shoot redifferentiation defective 2)
## VIT_17s0000g03070  -  R protein MLA10
## VIT_05s0020g00080  -  Unknown
## VIT_08s0007g03090  -  Kinesin family member 11
## VIT_00s0174g00090  -  far-red impaired responsive family protein
## VIT_06s0009g03290  -  Pre-mRNA branch site protein p14
## VIT_00s0513g00020  -  Peptidyl-prolyl cis-trans isomerase-like 2
## VIT_15s0046g02680  -  No hit
## VIT_04s0023g02060  -  Gamma carbonic anhydrase-like 1 (gamma CAL1)
## VIT_01s0010g01240  -  Argonaute (AGO1)
## VIT_16s0039g02380  -  Disease resistance family protein
## VIT_07s0141g01000  -  No hit
## VIT_18s0072g01030  -  Calcium-dependent protein kinase (VvCPK16)
## VIT_07s0197g00230  -  Disease resistance protein (CC-NBS-LRR class)
## VIT_19s0015g00810  -  SEC13
## VIT_00s0173g00240  -  No hit
## VIT_12s0035g00440  -  R protein disease resistance protein
## VIT_02s0025g04090  -  GCS1/HAP2 (generative cell-specific 1)
## VIT_00s0187g00220  -  Partial optokinetic response b
## VIT_00s0288g00060  -  No hit
## VIT_00s0187g00240  -  SWIB complex BAF60b domain-containing protein
## VIT_00s0174g00100  -  far-red impaired responsive family protein
## VIT_01s0127g00310  -  Unknown protein
## VIT_06s0004g03510  -  TIR-NBS-TIR type disease resistance protein
## VIT_12s0028g03230  -  RNA helicase
## VIT_14s0006g00070  -  Acyl-[acyl-carrier-protein] desaturase
## VIT_00s0432g00020  -  Unknown
## VIT_00s2564g00010  -  Unknown
## VIT_00s0227g00170  -  Syntaxin 52
## VIT_00s2574g00010  -  Protein transport protein Sec61 subunit alpha
## VIT_00s0187g00320  -  Cellulase
## VIT_01s0011g02480  -  Phospholipid-transporting ATPase
## VIT_01s0026g01170  -  Unknown
## VIT_04s0023g02550  -  Unknown protein
## VIT_17s0053g00460  -  Pinoresinol-lariciresinol reductase
```

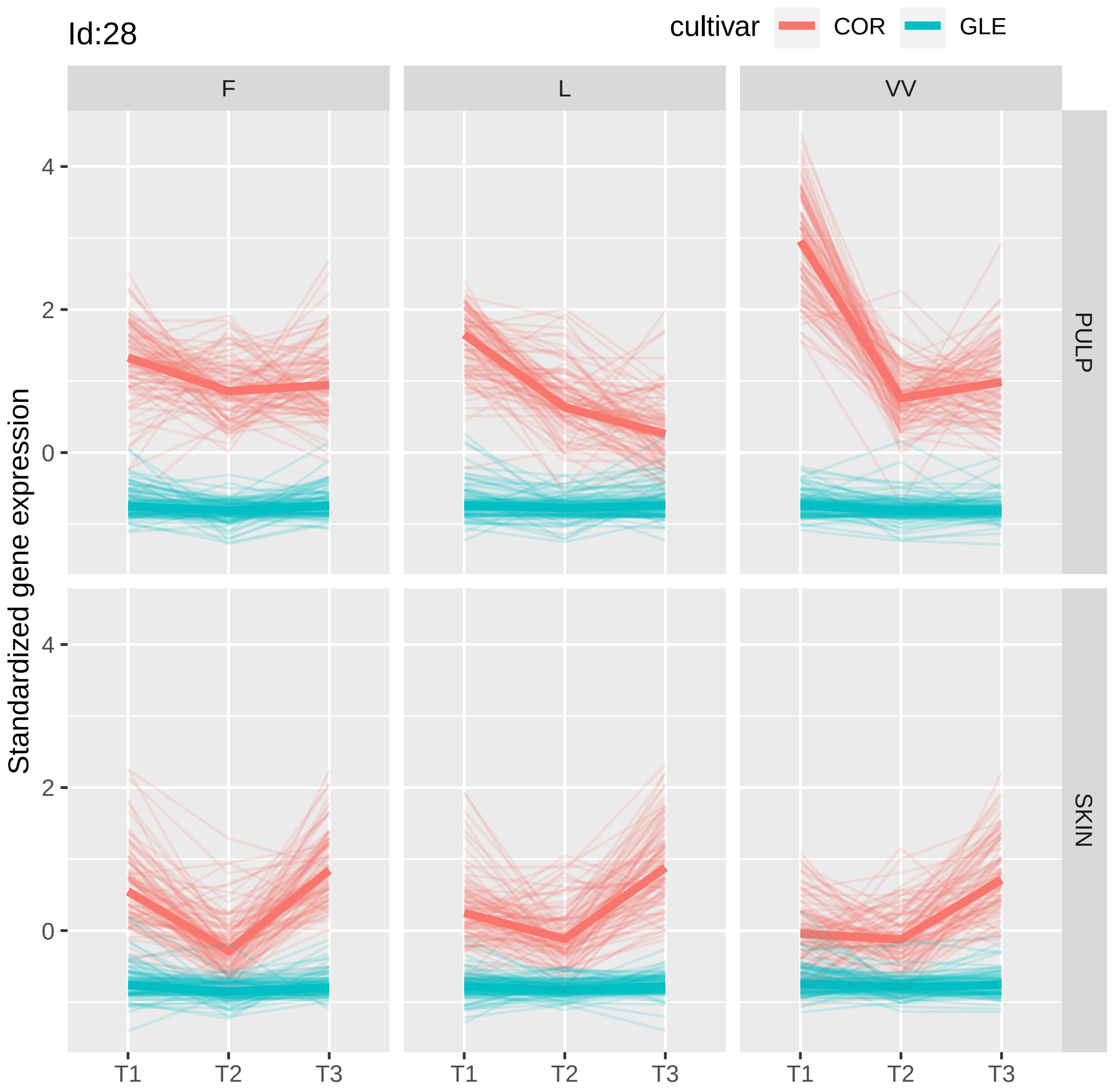

Id:28
cultivar
COR
GLE
F
L
VV
PULP
SKIN
Standardized gene expression
4
2
0
T1
T2
T3

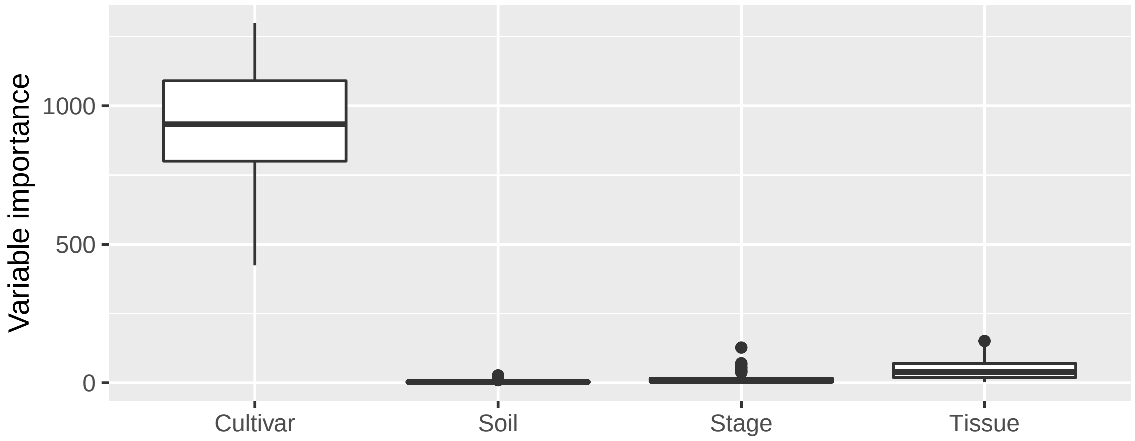

Variable importance
1000
500
0
Cultivar
Soil
Stage
Tissue

Cluster no. 102

```
## Number of genes in the cluster: 80
## Homogeneity Index:    0.87
## Variable importance for Stage:     Rank = 102  - Median =   6.34
## Variable importance for Cultivar:  Rank =   1  - Median =   1282
## Variable importance for Tissue:    Rank = 102  - Median =   5.57
## Variable importance for Soil:      Rank = 102  - Median =   1.84
##
## Gene ID                Gene Annotation
## VIT_09s0002g03330  -  Disease resistance protein (NBS-LRR class)
## VIT_03s0017g01430  -  Integral membrane Yip1 family protein
## VIT_13s0067g02370  -  SAC3/GANP
## VIT_00s2313g00010  -  Coiled-coil protein
## VIT_00s2037g00010  -  Unknown
## VIT_09s0002g07690  -  NADPH quinone oxidoreductase-like protein
## VIT_09s0002g07620  -  NADPH quinone oxidoreductase
## VIT_00s0672g00010  -  Translation initiation factor eIF-2B gamma subunit
## VIT_19s0176g00090  -  Agenet domain-containing protein
## VIT_00s0650g00030  -  Unknown
## VIT_01s0011g01020  -  RPM1 (resistance to p. syringae pv maculicola 1)
## VIT_13s0067g00790  -  R protein  PRF  disease resistance protein
## VIT_09s0002g07820  -  Quinone oxidoreductase
## VIT_09s0002g07840  -  NADPH quinone oxidoreductase-like protein
## VIT_01s0011g01000  -  RPM1 (resistance to p. syringae pv maculicola 1)
## VIT_19s0014g02960  -  No hit
## VIT_18s0041g02240  -  ABC Transporter (VvATH3 - VvABCA4)
## VIT_09s0002g07760  -  NADPH quinone oxidoreductase
## VIT_00s0524g00020  -  Cellulose synthase CSLC06
## VIT_00s0193g00160  -  Ankyrin
## VIT_17s0000g10080  -  Transcriptional factor B3
## VIT_19s0014g00880  -  Seven in absentia SINA5
## VIT_11s0052g01770  -  20S proteasome subunit alpha 2
## VIT_18s0089g00570  -  Acetyl-CoA acetyltransferase, cytosolic 2
## VIT_00s0207g00020  -  Unknown protein
## VIT_00s0163g00050  -  Argininosuccinate synthase
## VIT_18s0041g00440  -  Unknown
## VIT_00s0160g00280  -  No hit
## VIT_00s0163g00060  -  Zinc finger (C3HC4-type ring finger)
## VIT_16s0039g02360  -  Disease resistance protein (NBS-LRR class)
## VIT_09s0096g00930  -  Disease resistance protein (TIR-NBS-LRR class)
## VIT_00s0555g00050  -  CHCH domain containing protein
## VIT_16s0115g00470  -  Lucose inhibited division protein A
## VIT_02s0025g04660  -  Senescence-inducible chloroplast stay-green protein 1
## VIT_09s0002g04910  -  R protein  PRF  disease resistance protein
## VIT_19s0014g04000  -  Curculin (mannose-binding) lectin
## VIT_15s0048g00930  -  ATP-dependent DNA helicase 2 subunit 2 ku80
## VIT_03s0038g01730  -  KOW domain-containing transcription factor family protein
## VIT_12s0035g01250  -  Disease resistance protein (NBS-LRR class)
## VIT_01s0011g00950  -  RPM1 (resistance to p. syringae pv maculicola 1)
## VIT_12s0034g02350  -  R protein disease resistance protein
## VIT_00s2439g00010  -  No hit
## VIT_09s0002g07580  -  NADPH quinone oxidoreductase-like protein
## VIT_03s0110g00230  -  Seed maturation protein PM23
## VIT_05s0077g00200  -  Unknown protein
## VIT_12s0034g00750  -  MEKK1
```

```
## VIT_06s0004g08030  -  Alanyl-tRNA synthetase
## VIT_11s0206g00090  -  Calmodulin-binding protein
## VIT_16s0039g02350  -  Dihydroflavonol 4-reductase
## VIT_07s0130g00250  -  4,5-DOPA dioxygenase extradiol
## VIT_18s0089g00590  -  Glyceraldehyde-3-phosphate dehydrogenase, cytosolic
## VIT_09s0002g05050  -  RPS5 (resistant to p. syringae 5)
## VIT_03s0017g01090  -  Alanyl-tRNA synthetase
## VIT_00s0961g00020  -  Transducin protein
## VIT_00s0847g00010  -  Phosphoinositide binding
## VIT_12s0034g02310  -  R protein MLA10
## VIT_00s0160g00320  -  TIR-NBS-LRR type R protein 7
## VIT_09s0002g03270  -  RPS5 (resistant to p. syringae 5)
## VIT_16s0115g00460  -  Auxilin
## VIT_03s0091g00070  -  ACT domain-containing protein (ACR6)
## VIT_00s2472g00010  -  Enhancer of mRNA-decapping protein 4
## VIT_13s0064g00300  -  Separase
## VIT_12s0035g00410  -  Disease resistance protein
## VIT_03s0091g00060  -  ACT domain-containing protein (ACR6)
## VIT_03s0091g00530  -  N-acetyltransferase ESCO2
## VIT_02s0025g04020  -  S-N-methylcoclaurine 3'-hydroxylase
## VIT_16s0039g01930  -  No hit
## VIT_13s0101g00380  -  Vacuolar protein sorting 36 / VPS36
## VIT_19s0176g00050  -  No hit
## VIT_07s0031g02080  -  Alginate_lyase2
## VIT_18s0041g02400  -  Aldehyde oxidase
## VIT_09s0070g00450  -  No hit
## VIT_19s0176g00060  -  Unknown
## VIT_04s0008g03680  -  Unknown protein
## VIT_00s0269g00080  -  Peptidase C50, separase
## VIT_11s0037g01370  -  Ankyrin
## VIT_12s0055g00730  -  ATATH2 (ABC2 homolog 2)
## VIT_00s0233g00020  -  Transaldolase
## VIT_19s0093g00100  -  Clp protease proteolytic subunit 5
## VIT_16s0039g01920  -  myb domain protein 93
```

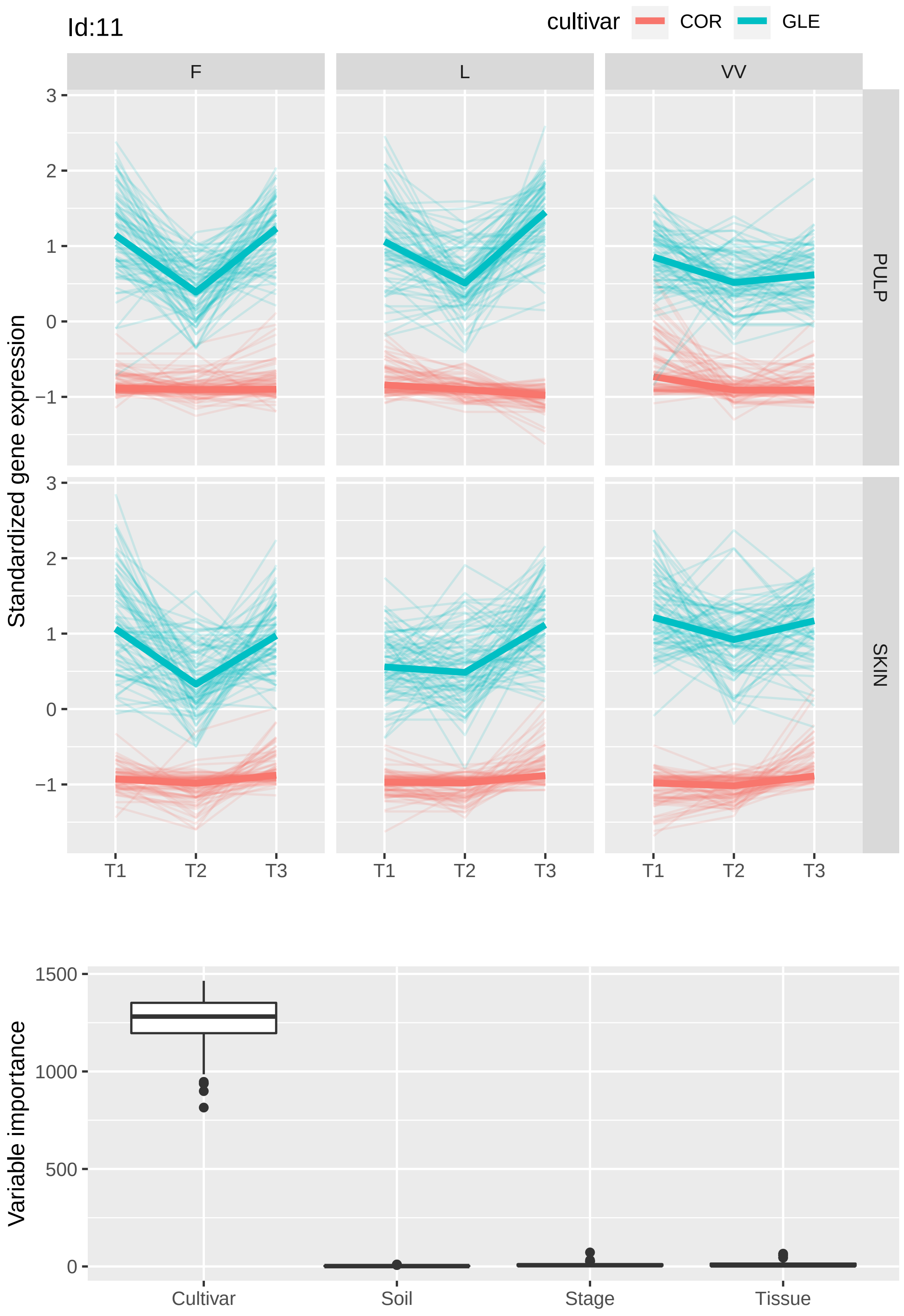
Id:11
cultivar
COR
GLE
F
L
VV
PULP
SKIN
Standardized gene expression
3
2
1
0
−1
T1
T2
T3
Variable importance
1500
1000
500
0
Cultivar
Soil
Stage
Tissue
